# Relationship between gene expression and lung function in Idiopathic Interstitial Pneumonias

**DOI:** 10.1186/s12864-015-2102-3

**Published:** 2015-10-26

**Authors:** Mark P. Steele, Leah G. Luna, Christopher D. Coldren, Elissa Murphy, Corinne E. Hennessy, David Heinz, Christopher M. Evans, Steve Groshong, Carlyne Cool, Gregory P. Cosgrove, Kevin K. Brown, Tasha E. Fingerlin, Marvin I. Schwarz, David A. Schwartz, Ivana V. Yang

**Affiliations:** Department of Medicine, Vanderbilt University, Nashville, TN USA; Center for Genes, Environment and Health, National Jewish Health, Denver, CO USA; Department of Medicine, University of Colorado School of Medicine, Aurora, CO USA; Department of Medicine, National Jewish Health, Denver, CO USA; Department of Biostatistics and Bioinformatics, Colorado School of Public Health, Aurora, CO USA; Department of Epidemiology, Colorado School of Public Health, Aurora, CO USA

## Abstract

**Background:**

Idiopathic interstitial pneumonias (IIPs) are a group of heterogeneous, somewhat unpredictable diseases characterized by progressive scarring of the interstitium. Since lung function is a key determinant of survival, we reasoned that the transcriptional profile in IIP lung tissue would be associated with measures of lung function, and could enhance prognostic approaches to IIPs.

**Results:**

Using gene expression profiling of 167 lung tissue specimens with IIP diagnosis and 50 control lungs, we identified genes whose expression is associated with changes in lung function (% predicted FVC and % predicted D_L_CO) modeled as categorical (severe vs mild disease) or continuous variables while adjusting for smoking status and IIP subtype; false discovery rate (FDR) approach was used to correct for multiple comparisons. This analysis identified 58 transcripts that are associated with mild vs severe disease (categorical analysis), including those with established role in fibrosis (*ADAMTS4*, *ADAMTS9, AGER*, *HIF-1α*, *SERPINA3*, *SERPINE2*, and *SELE*) as well as novel IIP candidate genes such as rhotekin 2 (*RTKN2*) and peptidase inhibitor 15 (*PI15*). Protein-protein interactome analysis of 553 genes whose expression is significantly associated with lung function when modeled as continuous variables demonstrates that more severe presentation of IIPs is characterized by an increase in cell cycle progression and apoptosis, increased hypoxia, and dampened innate immune response. Our findings were validated in an independent cohort of 131 IIPs and 40 controls at the mRNA level and for one gene (RTKN2) at the protein level by immunohistochemistry in a subset of samples.

**Conclusions:**

We identified commonalities and differences in gene expression among different subtypes of IIPs. Disease progression, as characterized by lower measures of FVC and D_L_CO, results in marked changes in expression of novel and established genes and pathways involved in IIPs. These genes and pathways represent strong candidates for biomarker studies and potential therapeutic targets for IIP severity.

**Electronic supplementary material:**

The online version of this article (doi:10.1186/s12864-015-2102-3) contains supplementary material, which is available to authorized users.

## Background

There is substantial clinical heterogeneity in the clinical, radiologic, and histopathologic features within each subtype of idiopathic interstitial pneumonias (IIPs). For instance, all forms of IIP have a somewhat unpredictable prognosis and many but not all patients can progress to end stage lung disease [1–3]. While subtypes of IIPs differ in clinical, radiographic, and histopathologic presentation [1–3], the type of IIP often cannot be determined and many of the subtypes of IIP have overlapping clinical and laboratory features indicating that the current definitions remain too broad.

Idiopathic pulmonary fibrosis (IPF), by far the most common form of IIP, is histopathologically defined by the presence of the prototypical form of pulmonary fibrosis, usual interstitial pneumonia (UIP), a fibrosing interstitial pneumonia characterized by a pattern of heterogeneous, subpleural regions of fibrotic, and remodeled lung that often results in death within 2–3 years of diagnosis [4]. Other IIPs, such as respiratory bronchiolitis-associated interstitial lung disease (RB-ILD), are more cellular, occur earlier in life, and have a considerably lower mortality [5]. By contrast, idiopathic nonspecific interstitial pneumonia (iNSIP), a pattern of IIP that is more likely a syndrome than a disease, is most commonly characterized by interstitial fibrosis but in a more uniform pattern than UIP, and carries a better prognosis than IPF/UIP [6, 7]. These differences within the subtype and overlaps among subtypes create a distinct challenge to achieve an accurate diagnosis for an individual patient with this potentially life-threatening diagnosis.

The molecular mechanisms that account for the extreme heterogeneity in clinical presentation, radiologic patterns, histopathologic variation, and disease progression are largely unknown. We hypothesize that biological heterogeneity in IIPs will be reflected by gene expression patterns in lung tissue from patients with IIP, and gene expression patterns will change as a function of disease activity, progression, and severity. To test this hypothesis, we measured gene expression in lung tissue of patients with IIP, and correlated gene expression patterns with diffusing capacity of the lung for carbon monoxide (D_L_CO) and forced vital capacity (FVC).

## Results

### Demographic characteristics of the Lung Tissue Research Consortium (LTRC) cohort

Table 1 summarizes demographic and clinical characteristics of the LTRC IIPs cohort and by subtypes of IIPs that include at least 10 individuals. Included in the table is the portion of the non-diseased control cohort used together with the LTRC cohort. Overall, the IIPs cohort is older than the controls. Within the IIP cohort, individuals within IPF/UIP group are the oldest followed by iNSIP, uncharacterized fibrosis, and RB-ILD. There are no significant differences across IIP subtypes in gender or racial distribution. Comparison of smoking histories reveals that approximately half of the individuals with IIP are former smokers, as compared to controls that are almost 50 % current smokers. Subjects with IPF/UIP and RB-ILD diagnosis reported higher pack-years compared to subjects with iNSIP, uncharacterized fibrosis, and controls; however, there is variability within each group and therefore there are no statistically significant differences among groups. We also compared St. George’s score, an indicator of overall lung health [8] in subtypes of IIP (no data available for controls) and did not find significant differences among groups. Finally, variables associated with lung function, % predicted pre-bronchodilator FVC and D_L_CO reveal better lung function in the RB-ILD group compared to other IIPs.

**Table 1:** Subject demographics and clinical characteristics of the derivation (LTRC) cohort by IIP subcategory

Disease group^a^	Control	All IIPs	IPF	INSIP	UF	RB-ILD	*P* value (IIP vs control)	*P* value (IIP subtypes)
Number	50	167	119	17	13	11		
Age - mean (std dev)	47.5 (16.4)	60.9 (10.2)	62.6 (8.7)	57.2 (12.2)	56.7 (16.4)	52.2 (10.9)	<0.0001*	0.0016***
Gender – % male	54	61	65	53	62	36	0.41**	0.27**
Race - % Caucasian	82	87	94	82	85	82	0.067**	0.18**
Smoker –							<0.0001**	0.12**
Current	21 (42 %)	3 (3 %)	0 (0 %)	1 (6 %)	0 (0 %)	1 (9 %)		
Former	7 (14 %)	96 (55 %)	70 (59 %)	11 (65 %)	6 (46 %)	6 (55 %)		
Never	20 (40 %)	58 (35 %)	41 (34 %)	4 (24 %)	7 (54 %)	4 (36 %)		
Unknown	2 (4 %)	10 (7 %)	8 (7 %)	1 (6 %)	0 (0 %)	0 (0 %)		
Pack years - mean (std dev)^b^	22.1 (19.5)	58.6 (103)	69.9 (115)	20.5 (23.8)	21.8 (14.1)	53.3 (30.8)	0.080*	0.37***
St George’s score - mean (std dev)	NA	48.3 (21.0)	46.6 (22.2)	50.9 (16.6)	54.5 (20.6)	37.8 (17.0)	NA	0.24***
Pre-BD FVC, %predicted - mean (std dev)	NA	63.1 (19.1)	61.3 (16.8)	69.9 (22.2)	59.7 (27.6)	85.7 (13.1)	NA	0.0005***
D_L_CO, %predicted - mean (std dev)	NA	48.4 (21.9)	45.9 (20.3)	58.7 (22.1)	48.7 (16.6)	77.1 (21.3)	NA	<0.0001***

^a^IIP = idiopathic interstitial pneumonia, IPF = idiopathic pulmonary fibrosis, UIP = usual interstitial pneumonia, iNSIP = idiopathic nonspecific interstitial pneumonia, UF = uncharacterized fibrosis, RB-ILD = respiratory bronchiolitis associated interstitial lung disease. Data not reported individually for cryptogenic organizing pneumonia (COP) (*n* = 3) and desquamative interstitial pneumonia (DIP) (*n* = 4) categories

^b^Average for current and former smokers

*Two-tailed student’s *t*-test

**Fisher’s exact test

***One-way ANOVA

### Identification of genes associated with the IIPs clinical subtype

To establish whether there are significant differences in expression in clinical subtypes of IIP, we first identified molecular profiles associated with clinically defined subtypes of IIP. For this initial analysis, we used an ANCOVA model that incorporates clinical subtype with at least 10 individuals per group (including controls), age, gender and smoking status as factors. Venn diagrams in Additional file 1: Figure S1 illustrate overlap of differentially expressed mRNAs amongst IIP categories versus controls using the 5 % FDR criterion alone or combined 5 % FDR and 2-fold change criteria. While the statistical interpretation of intersection-union testing among these groups is compromised by differences in the power of individual contrasts and by the common comparator group, a large proportion of the differentially expressed mRNA transcripts exhibit differences between control lung and two or more of the IIP subgroups. However, we also observed genes unique to each IIP subtype, especially in the IPF/UIP group which has the most differentially expressed transcripts overall as well as the most unique transcripts. This result is likely a combination of IPF/UIP being the largest group of IIPs examined in our study and the fact that IPF/UIP has the most remodeled lung of all IIPs. Examination of 29 genes in common to all IIPs using the 5 % FDR and 2 fold change criteria reveals genes involved in inflammation (*IL1RL1, IL1R2, IL18R1, IL8RAP*), extracellular matrix (*ITGA10, FCN3*), Wnt signaling (*SFRP2*), coagulation (alpha-1 antichymotrypsin or *SERPINA3*) and host defense (*DEFA3*) (Additional file 1: Table S1). Interestingly, defensin DEFA3 is one of the genes we identified as differentially expressed in peripheral blood of severe vs mild IPF/UIP categorized by differences in the D_L_CO [9]. We used the high degree of overlap among IIPs and biological relevance of genes identified in common to all IIPs as a rationale for inclusion of all IIP subtypes in the analysis of lung function variables as opposed to focusing only on IPF/UIP; however, we adjust for IIP subtype in all further analyses.

### Identification of genes associated with D_L_CO

We next sought to identify genes whose expression is associated with the D_L_CO measurement. For this analysis, we used an ANOVA model that incorporates categories of disease based on predicted D_L_CO (see below), IIP subtype and smoking status, or an ANCOVA model that incorporates the % predicted D_L_CO measurement, IIP subtype and smoking status as factors. Although no significant difference in smoking status exist among IIPs due to high variability in each IIP subtype, differences in smoking histories in these individuals may influence gene expression and we therefore included them in the model. We did not include age and gender because they are accounted for in the calculation of % predicted lung function variables. By categorical analysis, 91 unique transcripts differentiate mild disease (D_L_CO ≥65 %; *n* = 33) from severe disease (D_L_CO ≤35 %; *n* = 40) at 5 % FDR (Additional file 1: Table S2). When D_L_CO is treated as a continuous variable (135 subjects with D_L_CO measurement available), 706 genes correlate with changes in % predicted D_L_CO at 5 % FDR (Additional file 1: Table S3). Six hundred fourteen genes not identified in the categorical analysis were found to be associated with changes in D_L_CO in the continuous analysis.

### Identification of genes associated with FVC

We also identified gene expression changes associated with the FVC measurement, in an analogous manner to the analysis of D_L_CO. By categorical analysis, 681 genes differentiate mild disease (FVC ≥75 %; *n* = 50) from severe disease (FVC ≤40 %; *n* = 40) when categorized by % predicted FVC at 5 % FDR (Additional file 1: Table S4). Two thousand four hundred sixty seven genes correlate with changes in % predicted FVC in 164 subjects with available FVC data at 5 % FDR (Additional file 1: Table S5) with 1794 of these transcripts not overlapping with categorical analysis.

### Transcriptional changes in common to decline in D_L_CO and FVC

**Fig. 1:**
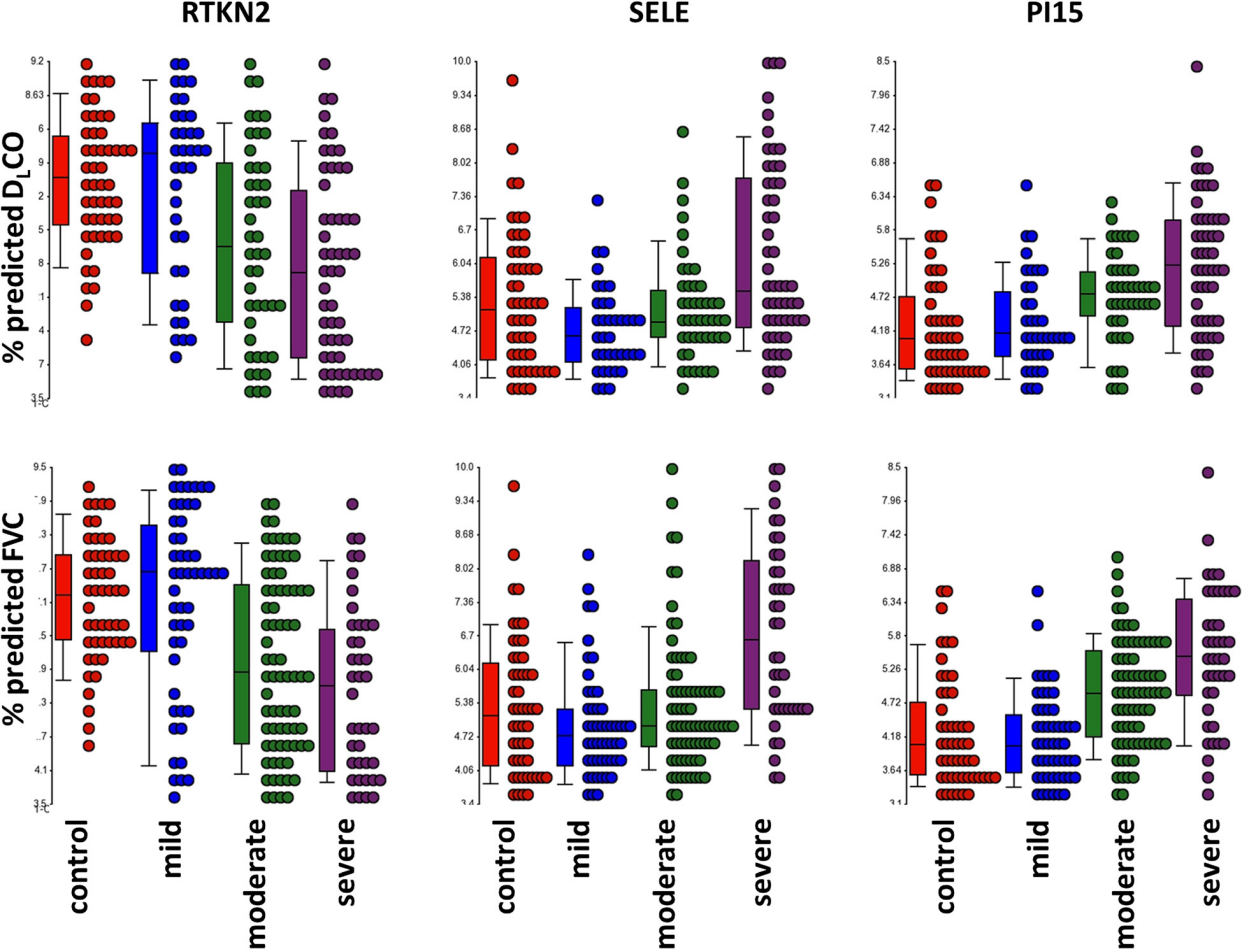
Categorical analysis of changes in gene expression based on lung disease severity categorized by % predicted D_L_CO (top) or % predicted FVC (bottom) for novel IIP candidate genes rhotekin 2 (RTKN2 - left), selectin E (SELE - middle), and peptidase inhibitor 15 (PI15 – right). Mild, moderate, and severe are defined as percent predicted D_L_CO (mild ≥65 %, moderate <65 % and >35 %, and severe ≤35 %), or FVC (mild ≥75 %, moderate <75 % and >40 %, and severe ≤40 %). *p* values for severe vs mild groups are shown in Table 2

**Fig. 2:**
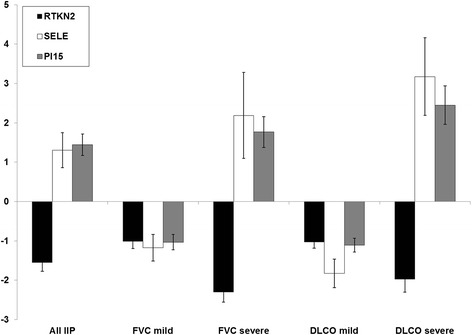
qRT-PCR validation of microarray data. Fold changes relative to control lungs for rhotekin 2 (black bars), selecting E (white bars) and PI15 (gray bars) in all IIPs and mild and severe disease based on either FVC or D_L_CO. Error bars represent standard deviations

To focus on genes that are the most likely to be involved in the pathogenesis of IIP, we identified differentially expressed transcripts in common to lower measures of D_L_CO and FVC. We first intersected 91 transcripts identified as significant in the categorical analysis of disease severity based on D_L_CO and 681 from the analogous analysis of FVC. Fifty eight transcripts that are in common to the two analyses are listed in Table 2. While a number of these transcripts, including ADAMTS family members (*ADAMTS4*, *ADAMTS9*) [10], *AGER* [11], *HIF-1α* [12, 13], serpin family members (*SERPINA3*, *SERPINE2*) [14], and selectin E (*SELE*) [15] have an established role in lung fibrosis, expression of novel IIP candidate genes such as rhotekin 2 (*RTKN2*) and peptidase inhibitor 15 (*PI15*) is also significantly altered in severe compared to mild disease. Dot plots shown in Fig. 1 demonstrate decrease in *RTKN2* but an increase in *SELE* and *PI15* with a decrease in lung function. We confirmed expression levels of these three genes in the same set of LTRC samples by qRT-PCR (Fig. 2).

**Table 2:** Differentially expressed genes (5 % FDR) in severe vs mild disease as characterized both by a decline in % predicted D_L_CO and FVC

Transcript ID	Gene symbol	*p*-value (Severe vs Mild D_L_CO)	Severe/Mild Fold Change (D_L_CO)	*p*-value (Severe vs Mild FVC)	Severe/Mild Fold Change (FVC)
7922229	SELE	9.4E-05	2.87	2.9E-05	2.80
7921821	ADAMTS4	5.0E-05	2.22	2.8E-06	2.41
8146957	PI15	1.9E-05	2.04	1.7E-06	2.16
8043981	IL1R2	0.00035	1.86	2.5E-05	2.00
7976496	SERPINA3	9.5E-06	1.81	3.3E-05	1.76
8088560	ADAMTS9	1.4E-05	1.84	8.5E-05	1.75
8162276	NFIL3	0.00011	1.59	6.9E-06	1.63
7974851	HIF1A	6.8E-07	1.52	2.7E-07	1.57
8145122	SLC39A14	0.00012	1.51	1.9E-05	1.51
8113220	ELL2	8.2E-06	1.52	2.6E-05	1.51
8106743	VCAN	8.3E-05	1.48	0.00014	1.49
7921344	ELL2	1.6E-05	1.56	3.0E-05	1.49
7902227	GADD45A	2.6E-05	1.33	6.4E-08	1.48
8059376	SERPINE2	0.00031	1.57	0.00013	1.45
8043909	NPAS2	5.2E-05	1.56	0.00067	1.45
7897449	SPSB1	0.00011	1.36	4.3E-06	1.44
8044391	MERTK	0.00015	1.46	1.5E-05	1.43
8168749	SRPX2	1.9E-06	1.57	3.0E-05	1.43
8156043	PSAT1	0.00017	1.40	9.0E-06	1.43
8115814	SH3PXD2B	3.0E-05	1.47	3.0E-05	1.42
8106098	MAP1B	3.4E-05	1.48	0.00019	1.42
8157216	UGCG	3.8E-05	1.39	5.3E-06	1.41
8099685	LGI2	0.00032	1.45	0.00018	1.40
8007931	ITGB3	4.4E-05	1.37	5.2E-05	1.40
7986446	ALDH1A3	3.1E-05	1.49	0.0020	1.37
8105040	OSMR	0.00012	1.33	3.00E-05	1.35
8043995	IL1R1	3.4E-05	1.34	3.8E-05	1.35
7922610	ABL2	0.00034	1.39	3.8E-05	1.33
7915787	PIK3R3	2.9E-05	1.26	4.9E-05	1.33
8006123	CPD	0.00017	1.24	3.2E-05	1.27
8066939	B4GALT5	0.00026	1.25	3.2E-05	1.27
8133155	TPST1	0.00021	1.32	1.1E-05	1.25
8041168	SNORD53	0.00019	1.30	0.0018	1.25
8102482	SEC24D	1.0E-05	1.31	5.5E-05	1.24
8041149	WDR43	0.00010	1.29	0.00079	1.24
7985934	SEMA4B	0.00012	1.21	8.8E-05	1.23
8076515	ARFGAP3	0.00013	1.26	0.00035	1.21
8117128	E2F3	3.5E-06	1.27	0.00022	1.20
7966839	VSIG10	0.00021	−1.17	0.0026	−1.15
8109086	ADRB2	0.00015	−1.24	0.0013	−1.17
8035201	CPAMD8	5.5E-05	−1.30	0.0010	−1.22
8041206	LBH	0.00036	−1.33	0.0015	−1.26
7940530	C11orf9	3.2E-05	−1.24	0.0019	−1.27
8149885	ADRA1A	1.7E-05	−1.33	4.1E-05	−1.27
8082465	CCDC48	9.8E-05	−1.28	8.1E-06	−1.28
8038117	DBP	0.00019	−1.35	0.00016	−1.31
8057677	SLC40A1	0.00038	−1.41	0.0023	−1.32
7968650	C13orf36	7.1E-06	−1.38	0.00019	−1.34
8089467	ZBED2	3.5E-05	−1.45	0.00021	−1.45
8171248	KAL1	5.3E-05	−1.67	0.00077	−1.50
8155734	FAM189A2	2.0E-05	−1.56	4.0E-05	−1.54
8156569	MIR23B	0.00024	−1.71	3.0E-06	−1.66
8179967	AGER	6.22E-05	−1.81	0.0023	−1.74
8125341	AGER	7.5E-05	−1.42	0.0026	−1.77
8178771	AGER	7.1E-05	−1.67	0.0025	−1.78
8109383	GRIA1	4.9E-06	−1.86	3.4E-05	−1.81
8171427	FIGF	0.00028	−2.07	0.0011	−1.90
7933855	RTKN2	0.00020	−2.57	0.0016	−2.34

**Fig. 3:**
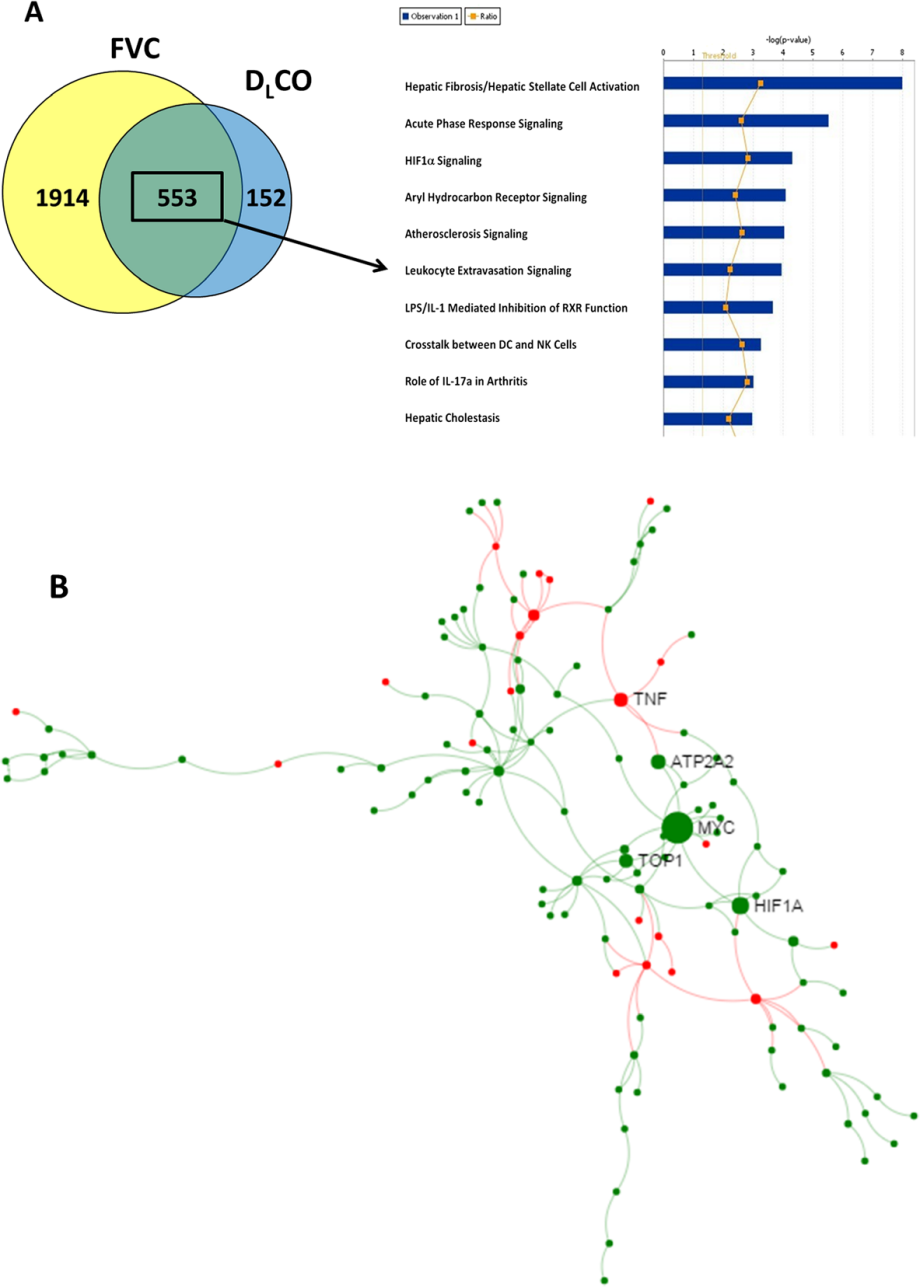
553 differentially expressed genes common to FVC and DLCO by continuous analysis were evaluated by (**a**) canonical pathways and (**b**) protein-protein interactome analysis. Pathways and their associated –log *P* value, as determined by the Fisher Exact Test in Ingenuity Pathway Analysis, are shown as the blue bars. The ratio of the number of genes mapping to these pathways relative to the total number of genes in the pathway is plotted as orange squares. The interactome was created using NetworkAnalyst [33] and the InnateDB PPI dataset by first importing all 553 genes and then reducing the network to zero-order interactome. The nodes are colored based on their correlation of expression and lung function variables (green are negative and red are positive correlations). The sizes of nodes are proportional to their betweenness centrality values

Given that analysis of expression changes as a function of continuous decrease in lung function variables identified large number of additional genes, we use the overlap between transcripts associated with a decline in D_L_CO or FVC in the continuous analysis (553 unique transcripts) to identify canonical pathways and transcriptional networks that are likely to be important predictors of severity of IIPs. Canonical Pathways Analysis of the 553 genes identified hepatic fibrosis and hepatic stellate cell activation (*P* <1 × < 10^−7^), acute phase response signaling (*P* <1 × 10 ^−5^), and HIF-1α signaling (*P* <1 × 10^−4^) as the most significantly enriched (Fig. 3a). A number of other pathways were identified (*P* <0.001) including IL17a, DC and NK crosstalk, LPS/IL-1 mediated inhibition of RXR function, leukocyte extravasation signaling, atherosclerosis signaling, and aryl hydrocarbon receptor signaling. Protein-protein interactome analysis using the InnateDB database of interactions suggests that more severe presentation of IIPs is characterized by an increase in cell cycle progression and apoptosis (genes centered around MYC), increased hypoxia (genes centered around HIF-1α) and dampened innate immune response (genes centered around TNF-α) (Fig. 3b). Network analysis in Ingenuity Pathway Analysis identified seven high scoring networks (score >25; Additional file 1: Figure S2) that largely recapitulate findings from InnateDB database.

### Validation of gene expression changes in an independent cohort

To test the generalizability of our findings, we examined expression of the candidate genes with the strongest associations with DLCO and FVC in an independent cohort of IIPs. Additional file 1: Table S6 provides characteristics of the replication (National Jewish Health or NJH) cohort. We primarily focused this analysis on the most pronounced transcriptional changes, 4 genes with >2 fold change from the categorical analysis of both FVC and D_L_CO (*RTKN2, PI15, ADAMTS4, and SELE*; Table 2). We used the same ANOVA models as in the derivation cohort, incorporating lung function variable, IIP subtype, and smoking, on these 4 genes to assess whether there are significant differences in expression between mild and severe disease, as defined by percent predicted D_L_CO (22 mild and 17 severe) or FVC (36 mild and 23 severe). The results of this analysis (Table 3) demonstrate that extent of transcriptional changes associated with lung function are dampened in this cohort compared to the LTRC cohort. However, all genes have *p* <0.05 for association with either FVC or DLCO. We also performed an analysis to validate 58 genes from Table 2 in the replication cohort in the same manner as previous analysis. 44/58 (76 %) genes have nominal *p* <0.05 in at least one of the analyses (Additional file 1: Table S7). We also considered lack of RB-ILD samples in the replication cohort as a counfounder of our validation analysis. This subtype of IIPs is characterized by better lung function than IPF and iNSIP; presence of RB-ILD cases with better lung function creates a wider range of values for FVC and D_L_CO in the LTRC cohort and may potentially allow for identification of more associations despite controlling for IIP subtype in the model. 49/58 (85 %) genes identified as significant in the categorical analysis of FVC and D_L_CO (Table 2) remain significant after removal of RB-ILD samples from the analysis. Similarly, of the 533 genes identified in common to D_L_CO and FVC when considered as continuous variables, 401 genes (75 %) remain significant in the analysis without RB-ILD. Therefore, RB-ILD samples do not significantly influence the results of our analysis beyond loss of power with fewer samples.

**Table 3:** Validation of gene expression changes from the LTRC IIP cohort in an independent cohort of IIPs (NJH cohort)

	D_L_CO categorical		FVC categorical	
Gene symbol	*p*-value	Severe/mild fold	*p*-value	Severe/mild fold
ADAMTS4	0.001531	1.70	0.337822	1.12
PI15	0.046174	1.33	0.181961	1.27
RTKN2	0.252356	−1.27	0.045309	−2.32
SELE	0.000903	2.01	0.425210	−1.06

### Localization of gene expression changes

**Fig. 4:**
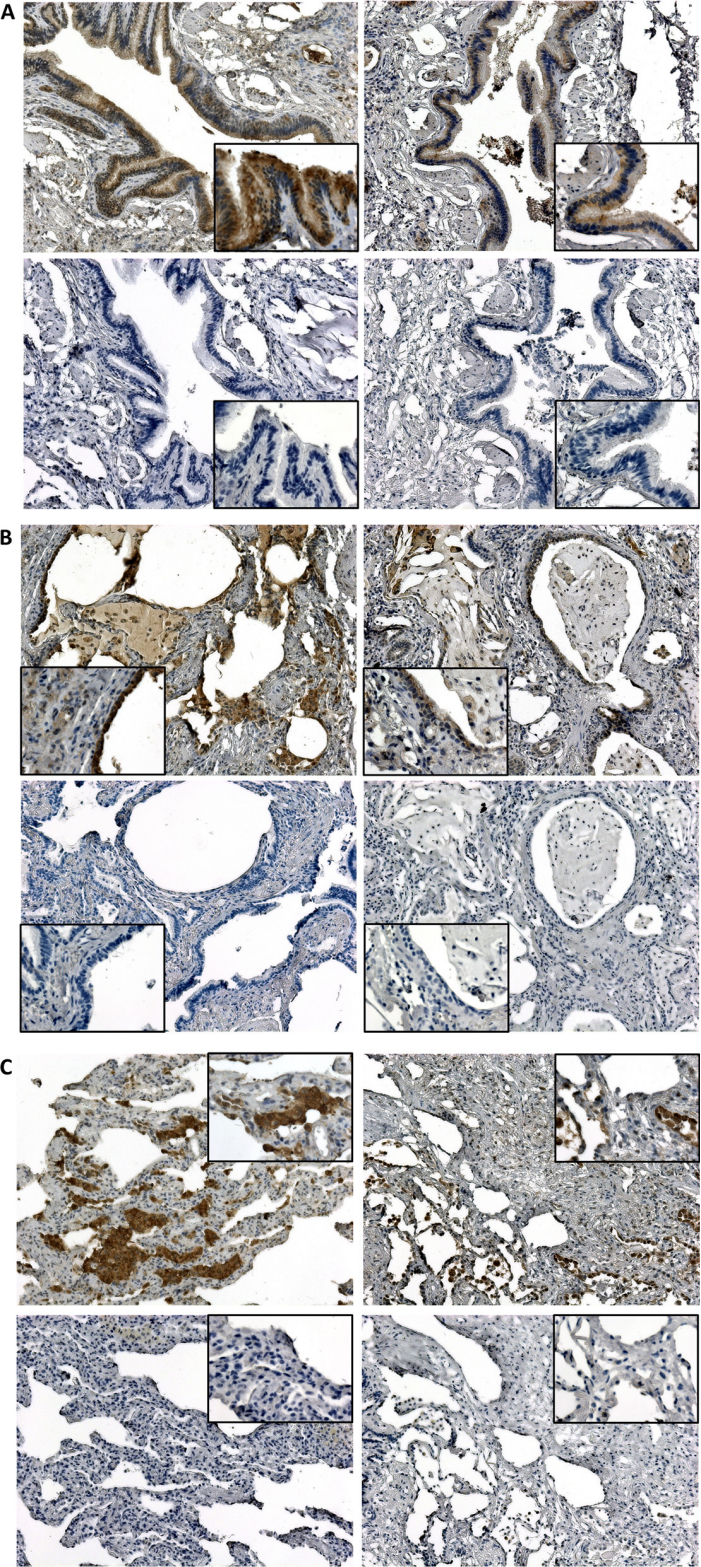
Localization of rhotekin 2 expression in IPF lung of patients with mild (left panels) and severe (right panels) disease. Immunohistochemical staining of airways (**a**), honeycomb cysts (**b**), and alveolar cysts (**c**) lung tissue reveals some decrease in expression of rhotekin 2 in airway epithelial cells in patients with severe compared to mild disease accompanied by an increase in expression in hyperplastic alveolar type II cells in alveolar cysts. Top panels represent rhotekin 2 stained sections and bottom panels are corresponding tissue sections incubated with non-immune serum (negative primary antibody controls). Tissue sections were counterstained with hematoxylin. Images were taken at 10× and 40× (inset) magnifications

To partially address the issue of cell specificity of gene expression profiles identified in whole lung tissue, we performed immunohistochemistry to localize expression of rhotekin 2, one of the genes with most prominent expression change associated with diseases progression and severity, in normal and IIP lung. In histologically normal lung tissue, rhotekin 2 is expressed in airway/bronchial epithelia, alveolar epithelia, alveolar macrophages, and submucosal glands (Additional file 1: Figure S3). We next examined different histopathological features (airways, honeycomb cysts and alveolar cysts) in lung tissue of IPF subjects with more compared to less severe disease. While we did not observe consistent differences across all subjects we examined, we observed a trend of lower expression of rhotekin 2 in IPF airways (Fig. 4a), honeycomb cysts (Fig. 4b), and alveolar cystic areas (Fig. 4c) of individuals with more severe disease. Based on our data, reduced mRNA levels of rhotekin 2 in severe IPF are most likely a combination of reduced expression in specific cells and loss of healthy airway epithelia that are replaced by honeycomb cysts [16]. Localization of rhotekin 2 in iNSIP lung tissue also demonstrated a decrease in expression in more severe disease (Additional file 1: Figure S4). These findings corroborate the findings of reduced rhotekin 2 expression at the mRNA level in more severe IIPs.

## Discussion

Results of our investigation of global gene expression changes in lungs of patients with IIPs demonstrate that, at the molecular level, there are more commonalities than differences among different subtypes of IIPs. On the other hand, the extent of disease, as characterized by lower measures of FVC and D_L_CO, is associated with marked changes in expression of novel and established genes and pathways involved in IIPs.

Our findings that lung tissue from IPF, iNSIP, RB-ILD, and uncharacterized fibrosis share many common expression patterns supports the concept that, despite differences in clinical presentation, these diseases may be related etiologically and pathogenically. Three publications [17–19] indicate that IPF/UIP and iNSIP, presumed distinct clinical-pathologic processes, may be related etiologically and pathogenically. Two of these studies [17, 19] have used transcriptional profiles of affected lung tissue to create IIP molecular signatures, and their findings suggest that dissimilar histological patterns may be related biologically.

In addition to some differences in expression among different IIP subtypes, we identified more pronounced changes in gene expression associated with more advanced disease. This conclusion is supported by previous reports [20, 21]. Selman et al*.* identified 437 differentially expressed genes in rapid compared to slow progressors, including overexpression of genes involved in morphogenesis, oxidative stress, migration/proliferation, and genes from fibroblasts/smooth muscle cells [20]. Our group also demonstrated differential expression (*p* <0.05 and >5 fold change) of 191 transcripts in individuals with progressive IPF (based on changes in D_L_CO and FVC over 12 months) compared to slow progressors [21]. While only less than 10 % of the genes in Table 2 (*AGER*, *C11orf9*, *NFIL3*, *PSAT1*, *RTKN2*, and *SERPINE2*) were identified by these earlier studies, additional overlap was observed when we considered gene families (for example *GADD45A* in the present study and *GADD45B* in our earlier publication), suggesting that the present study was successful at confirming published observations but also identifying novel gene transcripts. Two potential explanations for limited overlap are the fact that technologies and genome annotations have changed (our earlier study used the Serial analysis of gene expression [SAGE] as opposed to microarrays) and sample size (our current study has considerably larger sample size than previous publications).

Among the gene transcripts most strongly associated with disease severity are rhotekin 2 (*RTKN2*), selecting E (*SELE*), and peptidase inhibitor 15 (*PI15*). Rhotekin 2 is a member of a family of proteins containing a Rho-binding domain that are target peptides for the Rho-GTPases and are important in lymphocyte development and function [22]; however rhotekin 2 is ubiquitously expressed [23]. Genetic variants in rhotekin 2 have been associated with rheumatoid arthritis (RA) and activation of the NF-κB pathway in Japanese [24]. These known roles for rhotekin 2, the observation of decreased expression of rhotekin 2 in more severe disease, both in our earlier publication [21] and in the current study, and localization of expression to the lung epithelium in the current study, point to this gene as a candidate that warrants further functional investigation in IIPs. Selectin E is a member of the selectin family of cell adhesion molecules and plays a central role in adhesion of leukocytes to the endothelium [25]. Its expression inhibits bleomycin-induced lung fibrosis [15] but the soluble form of the protein product is increased in serum of patients with pulmonary fibrosis [26]. Finally, *PI15* is a trypsin inhibitor that is developmentally regulated in the lung mesenchyme [27] but otherwise is not well characterized; its role in development is consistent with recapitulation of developmental pathways in lung injury that is a hallmark of IIPs [28].

One of the strengths of the present study is the use of two independent cohorts of IIP for derivation and validation. However, we were only able to validate the most pronounced transcriptional changes identified in the derivation cohort. One possible explanation for this is that overall the NJH cohort has milder disease, an observation we have previously made [29]. We also considered the possibility that lack of RB-ILD cases in the replication cohort contributed to limited validation but ruled this out by showing that exclusion of RB-ILD cases from the derivation cohort did not substantially influence the results. A notable weaknesses of the present study in that, analogous to earlier publications, we have used whole lung tissue with the mixture of cells. Immunohistochemistry analysis of rhotekin 2, one of the genes with strongest correlation with disease severity in our study, demonstrates that reduced expression may be a combination of reduced expression in specific cells and loss of healthy airway epithelia that are replaced by honeycomb cysts. Further future studies will be needed to determine which of the genes identified by our analysis are differentially regulated in specific cell populations. Another weakness of our study is cross-sectional design in which we identify gene expression changes associated with a decline in lung function across a cohort of different individuals. A study of longitudinal design with measurements of gene expression and lung function over time in same individuals will be needed to validate these findings. However, the finding that rhotekin 2 expression was associated with more rapid disease progression in our earlier study [21] supports the notion that genes identified in our study have direct relevance to disease progression.

## Conclusions

In summary, we identified commonalities and differences in gene among different subtypes of IIP. Disease progression, as characterized by lower measures of FVC and D_L_CO, results in marked changes in expression of novel and established genes and pathways involved in IIP. These genes and pathways represent strong candidates for biomarker studies and potential therapeutic targets for IIP severity.

## Methods

### Subjects and tissue samples

All human tissue was collected with appropriate ethical review for the protection of human subjects. Written informed consent was obtained for all subjects’ participation in research by the Lung Tissue Research Consortium and National Jewish Health ILD Research Program. The current study only used de-identified information and was determined to be non-human subject research by both National Jewish Health and Colorado Multiple Institution IRBs. ATS/ERS guidelines were followed for diagnosis of IIP subtypes. The LTRC IIP cohort was used to derive gene expression signatures. NJH IIP cohort was used to validate gene expression signatures. The control tissue cohort was split to provide control lung expression profiles for both derivation and validation stages.

Lung tissue specimens from lower (*n* = 121), upper (*n* = 31), and middle/lingula (*n* = 15) lobes from subjects with IIP (119 IPF, 17 iNSIP, 13 uncharacterized fibrosis, 11 RB-ILD, 4 DIP, and 3 COP) were obtained from the LTRC. The LTRC is a resource created by the NHLBI to provide human lung tissues and DNA to qualified investigators for use in research. The program enrolls donor subjects who are anticipating lung surgery, collects blood and extensive phenotypic data from the prospective donors, and then processes their surgical waste tissues for research use. Most donor subjects have fibrotic interstitial lung disease or COPD. Clinical data include clinical and pathological diagnoses, chest CT images, pulmonary function tests (spirometry, D_L_CO, and ABG), exposure (including cigarette smoking history) and symptom questionnaires (including Borg dyspnea scale), and family history of lung disease.

The NJH ILD cohort consists of 131 patients with biopsy-proven IIP (111 IPF/UIP, 12 iNSIP, and 8 uncharacterized fibrosis) that were clinically evaluated by investigators at National Jewish Health. All subjects in this cohort have undergone a standardized evaluation designed to provide a specific diagnosis. The evaluation included a standardized history focused on the presence of current or previous systemic disease; medications; tobacco and recreational drug use; familial lung disease; avocational, occupational, environmental, and accidental exposures. Additional testing includes serologic evaluation for evidence of systemic disease, chest radiography, pulmonary physiology (including lung volumes by body plethysmography, spirometry before and after inhaled bronchodilator, and diffusing capacity), pressure volume curves, and gas exchange with exercise (formal six-minute walk testing and/or cardiopulmonary exercise testing). Video assisted thorascopic (VAT) or open surgical lung biopsy was performed as clinically indicated. The diagnosis of IIP was established using the criteria defined in the ATS/ERS consensus statement [1, 2].

Control, non-diseased lung tissue from lower (*n* = 86), and middle (*n* = 4) lobes was obtained from International Institute for Advancement of Medicine, formerly Tissue Transformation Technologies (Ediston, NJ). All individuals had suffered brain death and were evaluated for organ transplantation before research consent. Informed consent was obtained at the time of transplant evaluation. All specimens failed regional lung selection criteria for transplantation. Subjects had to demonstrate no evidence of active infection or chest radiographic abnormalities, mechanical ventilation <48 h, PaO_2_/FiO_2_ ratio >200, and no past medical history of underlying lung disease or systemic disease that involves the lungs (e.g., rheumatoid arthritis). Lung samples were procured within 34 h after brain death (mean, 16.2 h; range, 4.5–33.25 h). The control cohort was divided to proportionally provide the same percentage controls to LTRC and NJH IIP cohorts; 50 controls were used with the LTRC cohort and 40 with the NJH cohort.

### Microarray data generation

Total RNA including small RNA species was isolated from approximately 100 mg of snap-frozen lung tissue using the mirVana kit (AB/Ambion, Austin TX). RNA purity and concentration were determined by spectrophotometry, and RNA integrity was determined using the Bioanalyzer (Agilent, Santa Clara, CA). mRNA microarray target labeling was conducted using 300 ng of total RNA and the Message Amp II kit (AB/Ambion, Austin TX), hybridized to the Human Gene 1.0 ST Array (Affymetrix, Santa Clara, CA) and processed according to the manufacturer’s instructions. All microarray data met the quality control criteria established by the Tumor Analysis Best Practices Working Group [30] and are available in the Gene Expression Omnibus repository as GSE31962.

### Microarray data analysis

Expression data from 217 mRNA arrays (LTRC cohort; 167 IIPs and 50 controls) were analyzed using ANOVA implemented in Partek (St Louis, MO). Intensity data were imported, log_2_-transformed, and quantile normalized using RMA [31], and expression levels were summarized on a transcript level using the mean value of all probesets mapping to a transcript. Non-expressed and invariant transcripts were removed using a median variance filter, corrected by a Benjamini-Hochberg false discovery rate (FDR) of 0.10 [32], resulting in a final dataset of 11950 transcript measurements across 217 samples. Differential expression of individual transcripts was identified using an ANOVA (for categorical analysis) or ANCOVA (for continuous analysis) model incorporating lung function variable (%predicted FVC or D_L_CO), the final clinical diagnosis of each subject and smoking status. We included age and smoking status in the model as there are significant differences in these variables between IIP and control groups. We considered the impact of several technical variables including array batch, RNA preservative, RNA quality (RIN) and anatomic location of the lung biopsy; minimal expression changes were associated with these variables and we therefore did not include them in the final model. NJH cohort mRNA expression profiles were collected and processed in the same manner as the LTRC cohort data, with the exception of the final filtering step; in this case, 11950 transcripts from the LTRC dataset were retained in the dataset. Pathway analysis was performed using the Ingenuity Pathway Analysis (IPA) database and software (www.ingenuity.com) and the Fisher Exact Test to determine significant enrichments. We used the Network Analyst tool to generate the protein-protein interactome. NetworkAnalyst uses a comprehensive high-quality protein-protein interaction (PPI) database based on InnateDB [33]. The database contains manually curated protein interaction data from published literature as well as experimental data from several PPI databases including IntAct, MINT, DIP, BIND, and BioGRID. The database currently contains 14755 proteins and 145955 interactions for human, and 5657 proteins and 14491 interactions for mouse.

### Quantitative RT-PCR

Primers for mRNA expression were designed using Primer-BLAST and are listed in Additional file 1: Table S8. RNA was normalized to a concentration of 100 ng/μL and reverse transcribed to cDNA using the Applied Biosystems High Capacity cDNA Reverse Transcription Kit. Each 20-μL PCR contained 15 ng cDNA, 0.5 μM final concentration of forward and reverse primers and 1× final concentration of the Power SYBR Green master mix. Real-time PCR was performed on an Applied Biosystems Viia 7 instrument using the following profile: 50 °C for 2 min, 95 °C for 10 min, and 40 cycles of 95 °C for 15 s, and 60 °C for 1 min. Dissociation curves were collected at the end of each run. ΔCT values were calculated relative to GAPDH, and ΔΔCT values were calculated by comparison among different groups of samples.

### Immunohistochemistry

Standard immunohistochemical staining protocols were followed. Briefly, histological sections of normal and IPF lung were deparaffinized, blocked with hydrogen peroxide, followed by antigen retrieval in citrate buffer, and non-immune serum block. RTKN2 anti-rabbit antibody (Sigma, St. Louis MO; product number HPA037946) was added to histological sections at 1:200 final dilution and incubated overnight. Secondary antibody staining was performed with 3, 3′-diaminobenzidine (DAB) using the ImmPRESS kit (Vector Laboratories, Burlingame CA) and RTKN2 was visualized using the peroxidase substrate (ImmPact DAB kit). The sections were counterstained with hematoxylin. The primary antibody was replaced by non-immune serum for negative control slides.

## Additional file

Additional file 1:
**Supplemental Figures and Tables.** (PDF 2199 kb)
