# Supplementary material for: Relationship between gene expression and lung function in Idiopathic Interstitial Pneumonias
Source: BMC Genomics. 2015 Oct 26;16:869. doi: 10.1186/s12864-015-2102-3 (PMC4621862; doi:10.1186/s12864-015-2102-3)
Supplement: Additional file 1: — Supplemental Figures and Tables. (PDF 2199 kb) [file 12864_2015_2102_MOESM1_ESM.pdf]

(A)

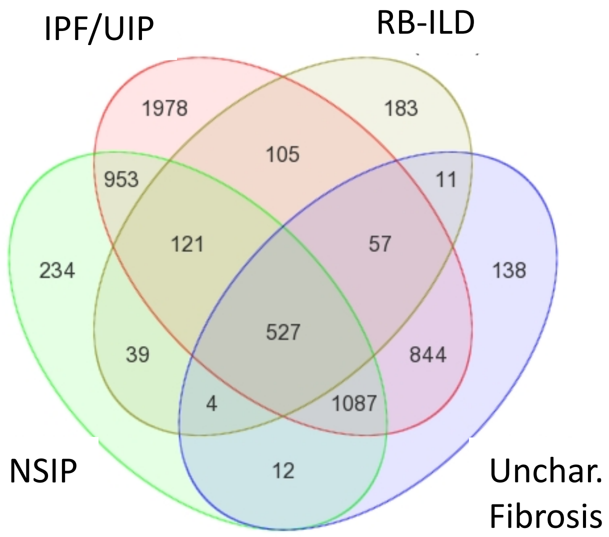

(B)

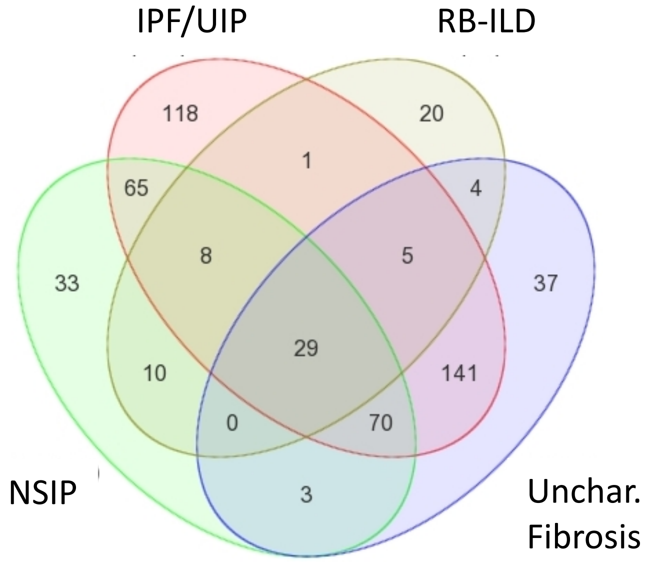

Supplemental Figure S1. Gene expression differences between control lung and IIP subcategories overlap significantly. **(A)** mRNA expression differences meeting the 5% FDR criteria by clinical subcategory. **(B)** mRNA expression differences meeting the 5% FDR and 2-fold change criteria by clinical subcategory.

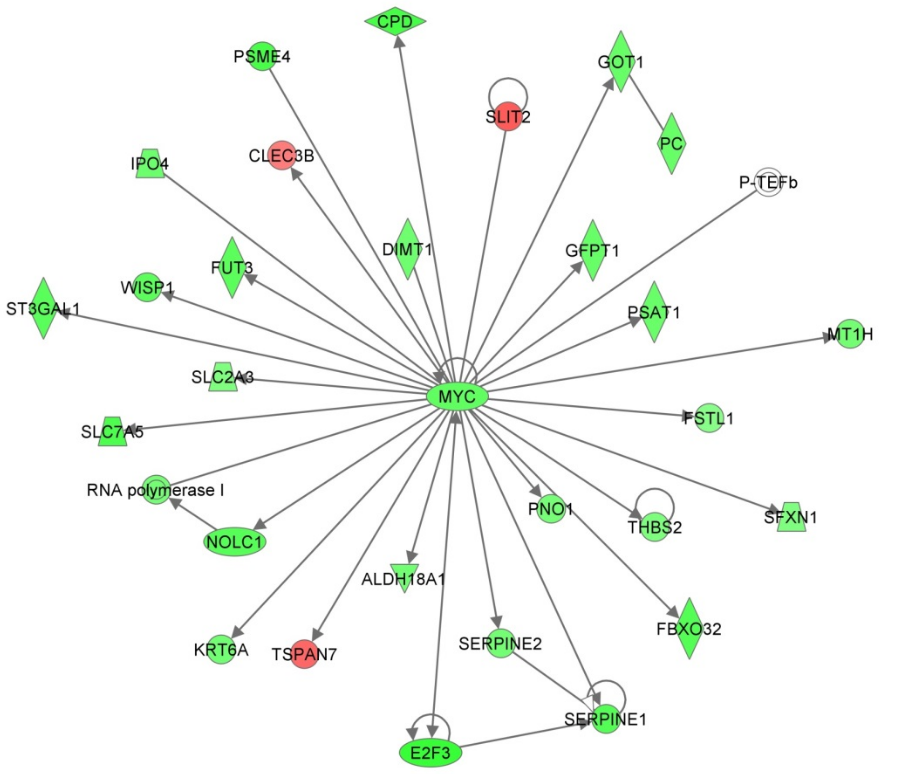

Network 1 (51)

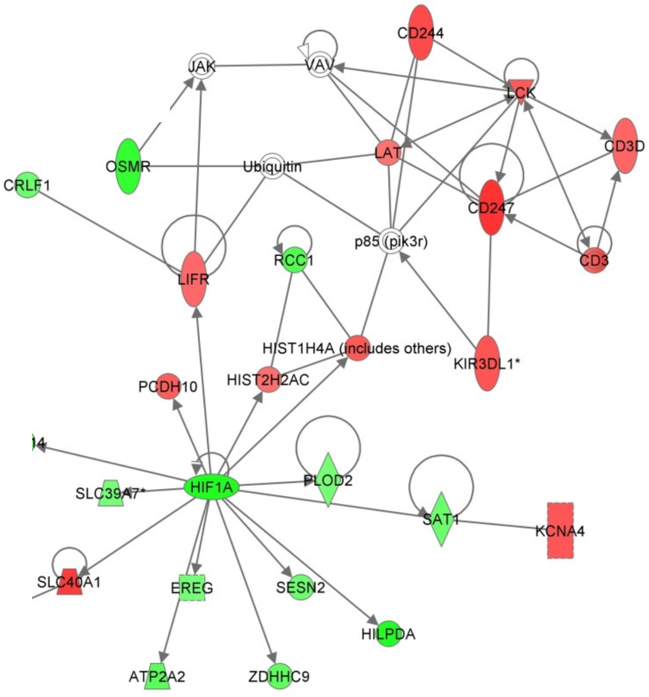

Network 2 (43)

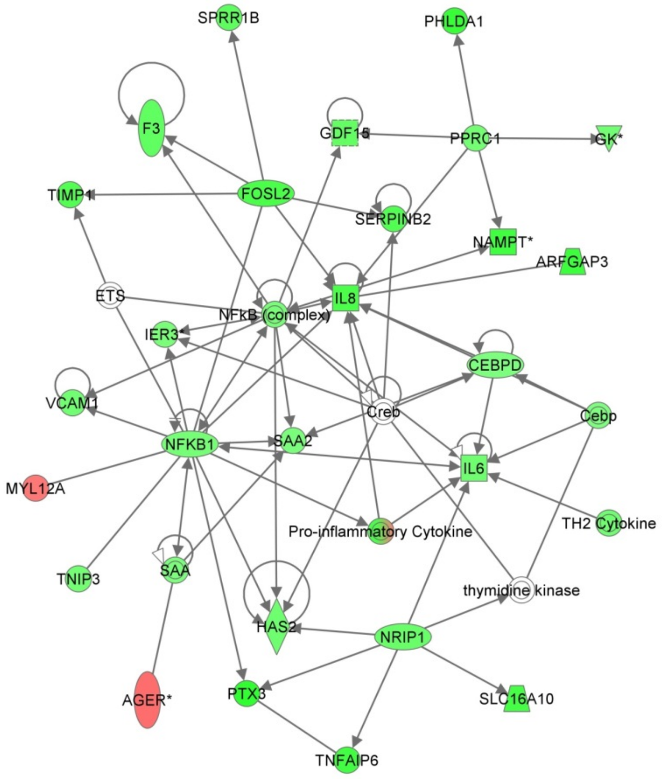

Network 3 (36)

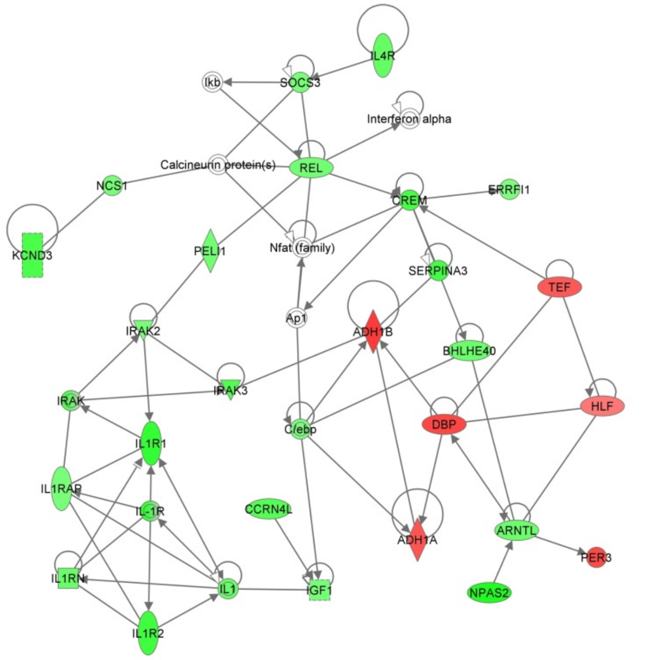

Network 4 (34)

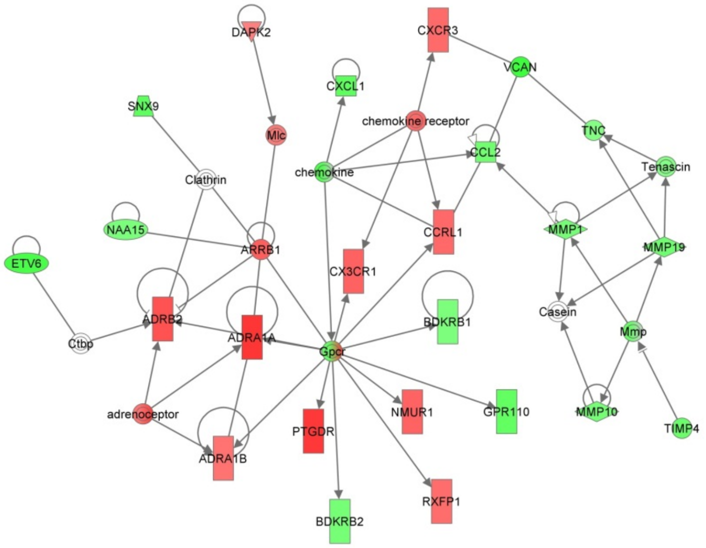

Network 5 (32)

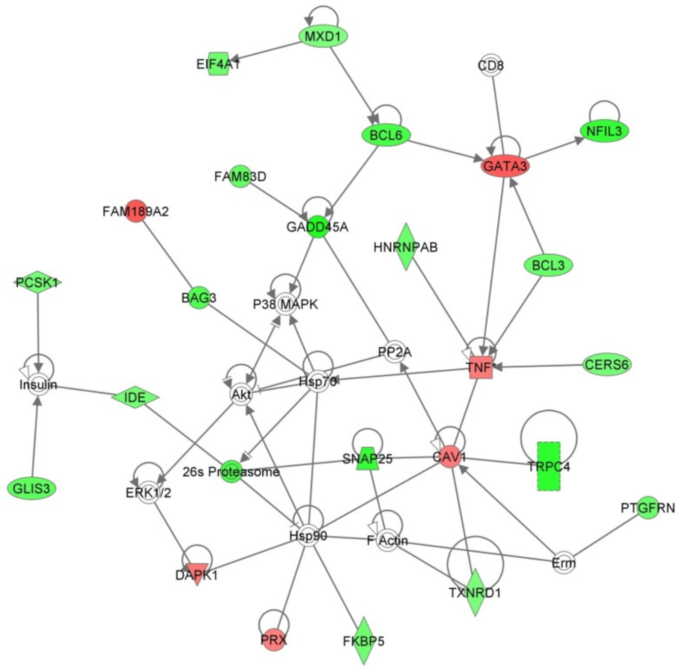

Network 6 (30)

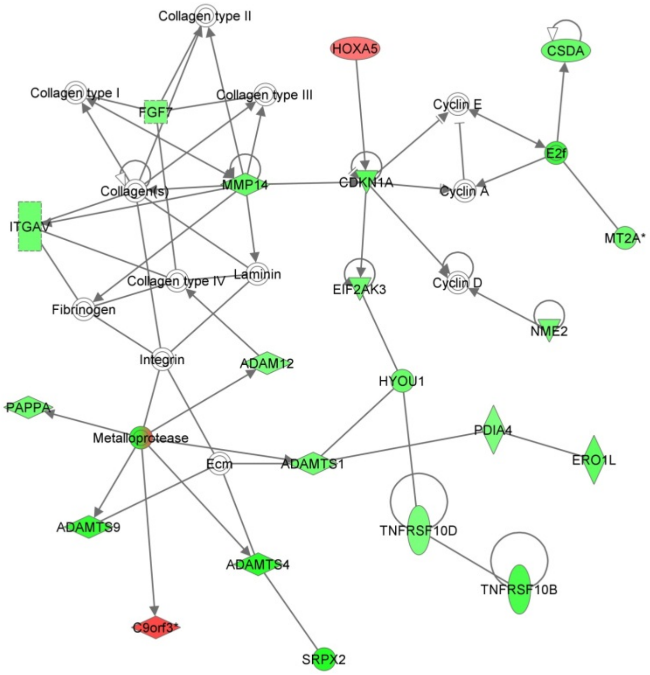

Network 7 (25)

Supplemental Figure S2. Networks from Ingenuity Pathway Analysis of 553 genes in common to continuous analysis of FVC and DLCO. Network scores are shown in parenthesis. Genes in networks are colored according to direction and magnitude of correlation with lung function where green indicates inverse (higher gene expression with lower lung function or worse disease) and red direct (lower gene expression with lower lung function or worse disease) correlation

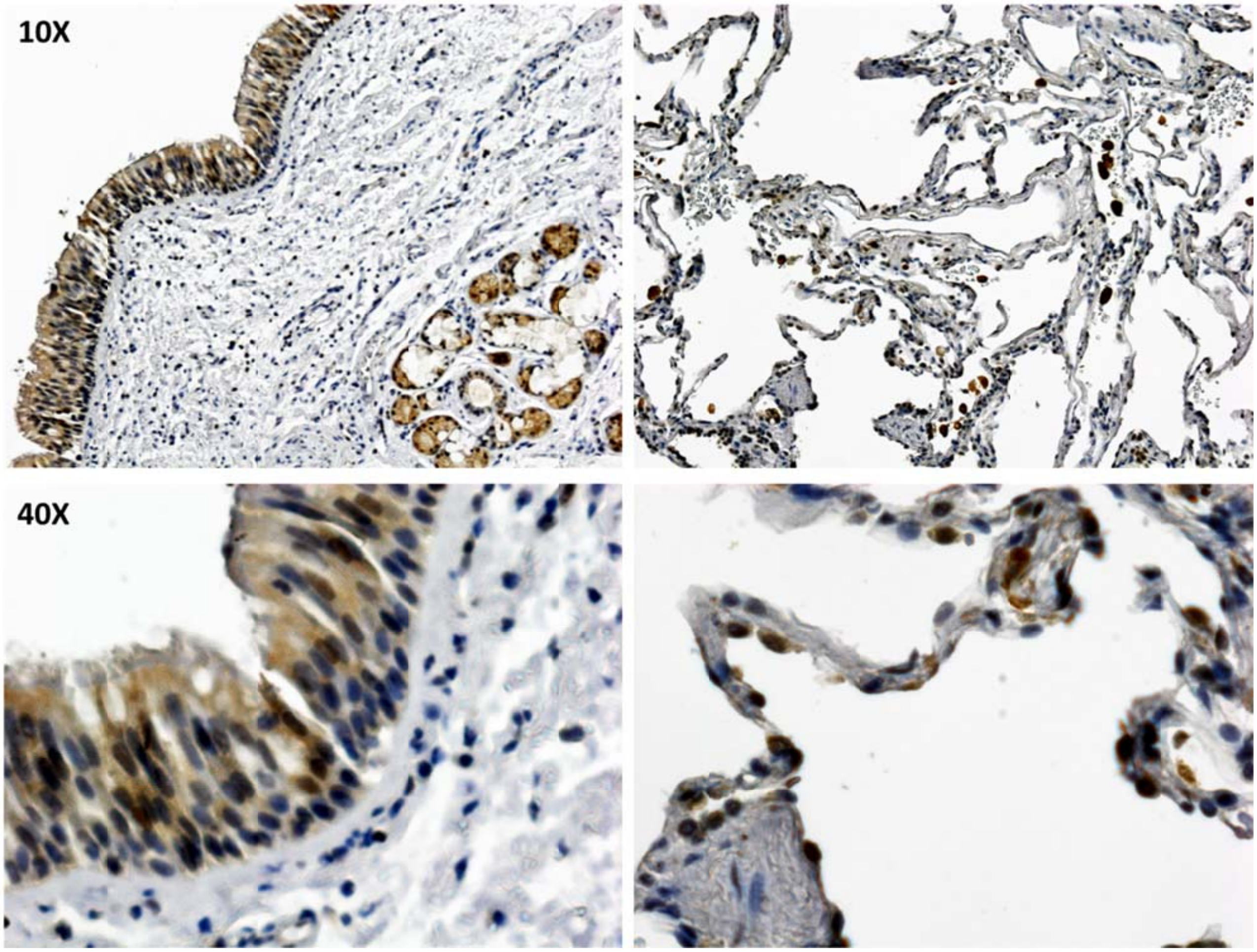

Supplemental Figure S3. Immunohistochemical staining of histologically normal trachea (left) and distal lung (right) tissue for rhotekin 2. Tissue sections were counterstained with hematoxylin. Images were taken at 10X (top) and 40X (bottom) magnification.

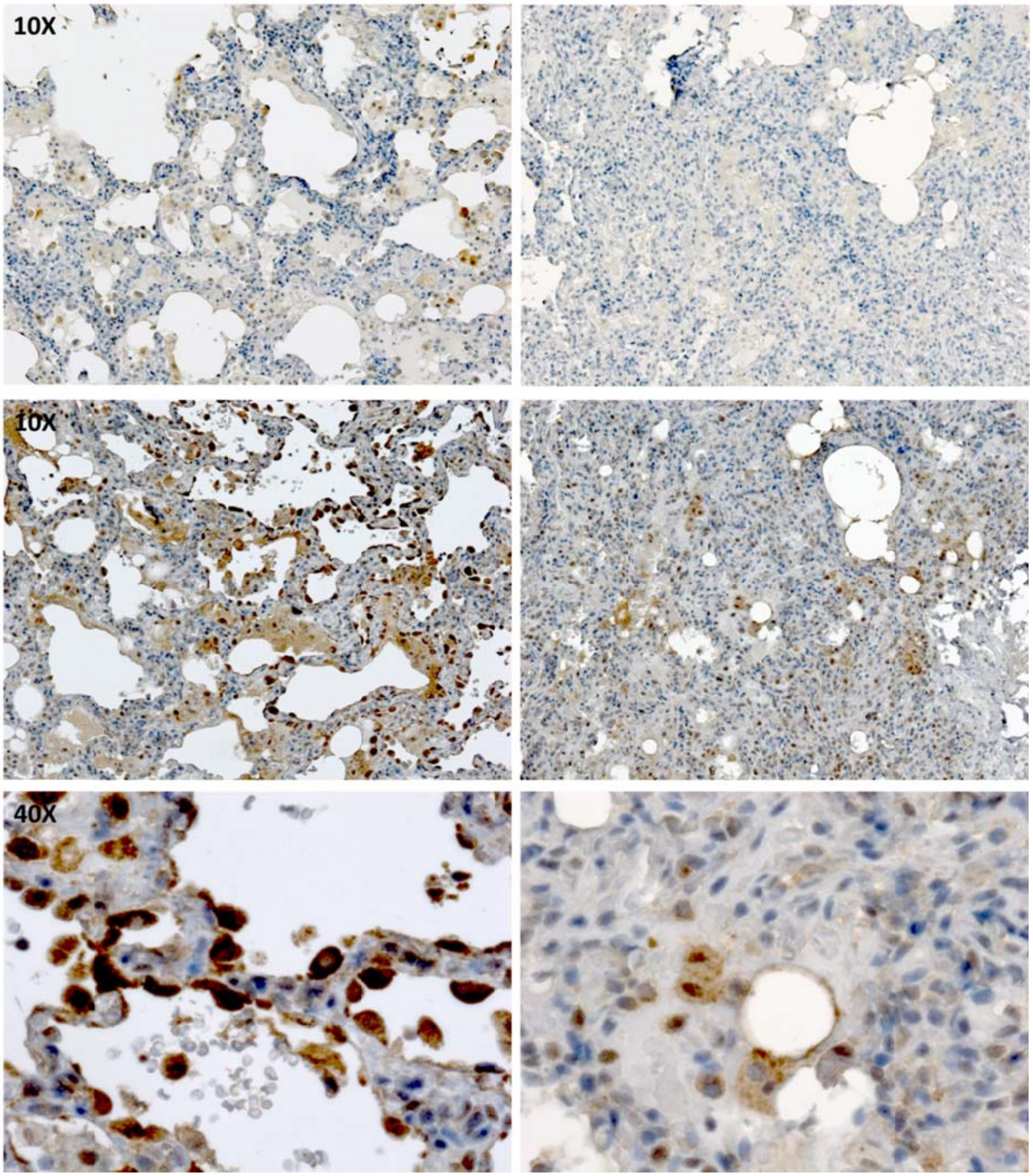

Supplemental Figure S4. Immunohistochemical staining of NSIP tissue for rhotekin 2. Left panels are tissue from a subject with mild disease while those on the right are from a patient with severe disease. Top row represents tissue sections incubated with non-immune serum (negative primary antibody controls), middle and bottom rows depict tissue sections stained with the primary rhotekin 2 antibodu at 10X and 40X magnification, respectively. Tissue sections were counterstained with hematoxylin.

Supplemental Table S1. Union of genes differentially expressed in different subtypes of IIP compared to controls using 5%FDR and 2 fold change criterion (corresponding to Venn diagram in Supplemental Figure S1B).

Transcript ID	Gene Symbol	RefSeq	p-value (IPF/UIP vs. Normal)	Fold-Change (IPF/UIP vs. Normal)	p-value (UF vs. Normal)	Fold-Change (UF vs. Normal)	p-value (NSIP vs. Normal)	Fold-Change (NSIP vs. Normal)	p-value (RB-ILD vs. Normal)	Fold-Change (RB-ILD vs. Normal)
8083415	AADAC	NM_001086	0.0270338	1.44533	0.117016	1.46983	0.0016473	2.0531	0.0065037	2.01497
8142554	AASS	NM_005763	3.42E-19	-1.99056	2.63E-10	-1.97902	2.85E-13	-2.09459	7.52E-13	-2.26682
8132743	ABCA13	NM_152701	8.10E-07	2.4554	0.0004609	2.53344	0.500747	1.17625	0.649468	1.13176
7998784	ABCA3	NM_001089	6.96E-11	-2.71568	0.0002948	-2.20501	0.020776	-1.58577	0.178253	-1.35259
8058591	ACADL	NM_001608	1.67E-08	-2.11805	0.0164797	-1.57899	0.036029	-1.44406	0.574329	1.11704
8103951	ACSL1	NM_001995	6.54E-17	-2.0507	1.81E-07	-1.87503	2.29E-07	-1.77563	5.33E-06	-1.76195
8042788	ACTG2	NM_001615	8.38E-12	2.44405	0.0005804	1.89158	0.0641485	1.36729	0.633273	1.095
8069676	ADAMTS1	NM_006988	5.39E-06	-1.91339	0.0638282	-1.4665	6.67E-07	-2.64224	1.96E-06	-2.85193
7921821	ADAMTS4	NM_005099	0.0430295	-1.41664	0.822994	1.05832	0.0125435	-1.7994	9.77E-05	-2.85031
8088560	ADAMTS9	NM_182920	0.998773	-1.00021	0.756948	-1.06511	0.648354	-1.08946	0.0007226	-2.07023
8101904	ADH7	NM_000673	1.01E-07	2.15155	0.0146819	1.65731	0.111291	1.35336	0.735025	1.07514
7938390	ADM	NM_001124	1.02E-11	-2.18533	0.0005399	-1.75682	2.71E-07	-2.19432	1.69E-07	-2.46992
8149885	ADRA1A	NM_000680	2.99E-28	-2.12973	2.55E-16	-2.16834	4.06E-14	-1.91512	4.92E-06	-1.52785
8054254	AFF3	NM_002285	2.18E-16	-2.40488	5.73E-06	-1.96566	1.51E-05	-1.80913	0.54195	-1.09669
7985741	AGBL1	NM_152336	0.869984	1.01703	0.650087	1.07172	0.0016436	1.56572	3.79E-06	2.12653
7947947	AGBL2	NM_024783	7.48E-08	2.12335	0.0022055	1.8565	0.18572	1.27663	0.229704	1.28451
8125341	AGER	NM_001136	7.14E-11	-3.07311	0.0208027	-1.75491	0.0004227	-2.22002	0.630315	1.12884
8178771	AGER	NM_001136	7.31E-11	-3.08209	0.0204303	-1.76103	0.0003914	-2.2363	0.616704	1.13478
8179967	AGER	NM_001136	6.66E-11	-2.92887	0.0201096	-1.71666	0.0003587	-2.16335	0.618647	1.12723
8138381	AGR2	NM_006408	4.77E-07	2.08975	0.0028673	1.88249	0.0460845	1.47358	0.553554	1.1383
8138392	AGR3	NM_176813	3.58E-09	2.02218	2.36E-05	2.07629	5.07E-05	1.90454	0.0029868	1.69615
8011680	ALOX15	NM_001140	0.0001045	2.07816	0.006036	2.13568	0.218587	1.36485	0.0062691	2.19585
8004784	ALOX15B	NM_001141	8.87E-07	-1.89692	0.0001001	-2.09868	0.0034627	-1.66406	0.0233018	-1.55996
7918794	AMPD1	NM_000036	2.46E-08	2.1864	0.0169369	1.61645	1.55E-05	2.25675	0.0142583	1.67102
7934979	ANKRD1	NM_014391	1.82E-06	-3.15104	0.240374	-1.50201	9.73E-05	-3.54643	0.298947	-1.45452
8154823	ANKRD18B	ENST0000029094	5.05E-10	2.08424	0.0236272	1.46127	0.0038756	1.56503	0.352135	1.17535
8047300	AOX1	NM_001159	2.25E-06	-1.84928	3.31E-06	-2.44384	0.0006343	-1.81782	5.60E-07	-2.73393

7948167	APLNR	NM_005161	1.01E-14	2.07522	2.58E-12	2.62054	5.21E-10	2.17686	5.17E-07	2.01054
8122058	ARG1	NM_000045	2.74E-21	-1.95955	1.95E-10	-1.87333	2.69E-14	-2.02879	4.55E-10	-1.89444
8171313	ARHGAP6	NM_013427	2.64E-22	-1.94795	7.45E-11	-1.85588	1.36E-15	-2.05156	0.0055969	-1.30004
7926638	ARMC3	NM_173081	1.52E-08	3.12798	0.0002442	2.91118	0.137228	1.4822	0.287774	1.37387
7932744	ARMC4	NM_018076	4.87E-08	2.55014	0.0012149	2.22955	0.157111	1.37689	0.286584	1.31246
8162394	ASPN	NM_017680	1.63E-26	5.33155	1.26E-14	5.30624	3.48E-08	2.88835	2.89E-06	2.7343
8110631	BTNL9	NM_152547	1.93E-20	-3.40661	6.94E-09	-2.88661	4.51E-10	-2.88287	0.0009833	-1.84148
7927723	C10orf107	NM_173554	8.30E-07	2.31827	0.0040691	2.03572	0.445621	1.18806	0.114766	1.49714
7936201	C10orf79	NM_025145	3.96E-09	3.94307	0.0005138	3.20465	0.0945934	1.6665	0.139313	1.66526
7930593	C10orf81	NM_024889	5.34E-09	3.56893	0.0012988	2.73764	0.0736372	1.66821	0.487706	1.25054
7943387	C11orf70	BC006128	2.12E-07	2.08478	0.0049065	1.77811	0.176943	1.28752	0.571237	1.12697
7943740	C11orf88	NM_207430	4.87E-07	2.47655	0.0011657	2.33997	0.286105	1.28974	0.20069	1.41212
7957673	C12orf55	ENST0000029895	2.75E-11	3.24291	0.000462	2.4091	0.0332974	1.62888	0.135623	1.47024
7957688	C12orf63	NM_198520	1.78E-09	2.84677	0.0013812	2.21807	0.117417	1.4276	0.265032	1.331
7958253	C12orf75	NM_001145199	3.56E-18	2.00671	2.99E-11	2.13174	1.34E-05	1.55677	0.0003021	1.5101
7968789	C13orf15	NM_014059	7.48E-16	-2.10248	0.0023044	-1.47322	1.68E-10	-2.17819	0.150382	-1.2077
7968866	C13orf30	NM_182508	5.97E-10	4.23018	8.62E-05	3.72465	0.0878969	1.6799	0.102498	1.7518
7921909	C1orf110	NM_178550	3.50E-07	2.10955	0.001596	1.95595	0.116811	1.35579	0.269013	1.27384
7907232	C1orf129	NM_025063	1.39E-07	2.21504	0.0046762	1.85275	0.169864	1.31489	0.232965	1.30818
7917019	C1orf173	NM_001002912	1.09E-08	2.97579	0.0004391	2.63103	0.0980008	1.51395	0.170869	1.47318
7921862	C1orf192	NM_001013625	3.21E-08	2.68857	0.0006441	2.41551	0.167286	1.38409	0.0878528	1.57538
7918294	C1orf194	NM_001122961	6.17E-08	3.57928	0.0002228	3.53097	0.204201	1.48295	0.183117	1.59506
7916629	C1orf87	BC027878	1.83E-06	2.13995	0.0031634	1.982	0.481861	1.16038	0.337534	1.25773
7903959	C1orf88	NM_181643	5.38E-07	2.04608	0.0006812	2.02558	0.247561	1.24444	0.358476	1.21667
8060940	C20orf103	NM_012261	6.45E-19	2.15368	7.27E-14	2.52977	4.20E-07	1.74477	0.0144493	1.34548
8061894	C20orf114	NM_033197	4.80E-10	10.6265	1.90E-05	10.3883	0.0200888	3.17212	0.0587658	2.88083
8061272	C20orf26	NM_015585	2.74E-07	2.2356	0.005845	1.86542	0.233821	1.27933	0.291768	1.27939
8063601	C20orf85	NM_178456	3.15E-08	3.0225	0.0001588	2.98754	0.195234	1.40582	0.144581	1.54305
8040672	C2orf39	NM_145038	3.93E-07	2.35025	0.00104	2.23067	0.170633	1.35744	0.152172	1.43467
8056710	C2orf77	NM_001085447	2.59E-08	2.51322	0.0016595	2.11771	0.119344	1.40402	0.145721	1.43047
8104758	C5orf23	BC022250	3.69E-09	-2.0824	0.0336034	-1.45726	2.00E-10	-2.96409	0.047686	-1.4408
8111864	C6	NM_001115131	2.71E-06	2.27029	0.0157837	1.8436	0.164346	1.38162	0.355252	1.27443
8122561	C6orf103	NM_024694	1.07E-07	2.35507	0.0058657	1.89732	0.252814	1.27521	0.3229	1.2679
8123951	C6orf105	NM_001143948	0.773967	1.03234	0.0291756	1.43276	0.11829	1.26704	1.26E-05	2.14501

8130664	C6orf118	NM_144980	4.20E-08	2.41828	0.0025838	2.01301	0.170419	1.33746	0.408129	1.21892
8126853	C6orf138	NM_001013732	1.22E-16	2.468	1.04E-11	2.90589	4.24E-07	2.03888	0.0008309	1.68628
8121015	C6orf165	NM_001031743	1.84E-08	2.65143	0.0003452	2.45058	0.168678	1.36822	0.286807	1.31504
8122827	C6orf97	NM_025059	7.46E-11	2.60739	7.45E-05	2.30317	0.0180555	1.57344	0.0458435	1.53983
8134051	C7orf63	NM_001039706	1.77E-13	2.19178	1.06E-05	1.94357	0.0148619	1.39529	0.0768496	1.31319
8158081	C9orf117	BC141809	1.64E-07	2.23743	0.0005823	2.15479	0.224239	1.27997	0.277655	1.2828
8155747	C9orf135	NM_001010940	1.65E-07	2.45682	0.0014634	2.20582	0.178351	1.357	0.304713	1.30059
8147123	CA3	NM_005181	0.0048561	-1.47268	0.0035707	-1.80914	0.0010728	-1.84969	1.15E-06	-2.85811
8051275	CAPN13	NM_144575	5.23E-08	2.36975	0.0064554	1.86139	0.170977	1.33095	0.223971	1.33215
8025004	CAPS	NM_004058	1.50E-07	2.1973	0.0002039	2.24678	0.176588	1.30668	0.123072	1.41217
8111506	CAPSL	NM_144647	6.25E-08	2.30771	0.0021098	1.98526	0.135217	1.35581	0.375221	1.22606
7961844	CASC1	NM_018272	5.03E-08	2.24804	0.0029059	1.89269	0.124808	1.35082	0.264567	1.27972
8070632	CBS	NM_001178008	2.80E-45	-2.19739	8.27E-25	-2.11072	6.66E-31	-2.23276	2.16E-22	-2.0661
8023314	CCDC11	NM_145020	6.72E-08	2.67796	0.0018874	2.2677	0.145324	1.41963	0.244434	1.37185
7996198	CCDC113	NM_014157	1.55E-08	2.46079	0.0003584	2.27196	0.119415	1.38522	0.364753	1.23848
8057377	CCDC141	NM_173648	4.44E-06	-1.92119	0.0004496	-2.07635	0.156921	-1.30762	0.793943	-1.05737
8133770	CCDC146	NM_020879	1.10E-11	3.3356	2.64E-05	2.8975	0.0146834	1.75263	0.0514923	1.65642
8092295	CCDC39	NM_181426	6.07E-07	2.00457	0.010454	1.67557	0.242252	1.24097	0.364703	1.208
7959108	CCDC60	NM_178499	3.21E-07	2.02868	0.0061764	1.72963	0.254929	1.23166	0.47895	1.15751
8089544	CCDC80	NM_199511	5.75E-15	3.41573	1.71E-08	3.54126	5.77E-09	3.34274	0.000464	2.22092
8006459	CCL13	NM_005408	3.79E-14	2.00758	0.0009356	1.53014	2.33E-09	2.07444	0.0001264	1.67451
8006594	CCL18	NM_002988	1.33E-07	2.12143	0.0094096	1.70505	2.51E-05	2.24567	0.0036471	1.86531
8006433	CCL2	NM_002982	0.0050545	1.53123	0.0007271	2.14487	0.254028	1.26423	0.473471	-1.18098
7960794	CD163	NM_004244	4.56E-14	-2.42559	2.58E-08	-2.55049	2.81E-06	-2.05004	7.48E-08	-2.55895
7906348	CD1C	NM_001765	1.07E-13	2.16625	3.80E-06	1.97683	9.38E-07	1.95172	8.24E-07	2.13774
8177222	CD24	NM_013230	4.92E-20	3.64844	1.64E-11	3.81405	5.06E-08	2.66124	0.0171464	1.59934
8154233	CD274	NM_014143	9.75E-11	-2.23948	0.0012847	-1.76922	0.0002943	-1.81001	0.0009074	-1.84563
7997504	CDH13	NM_001257	9.82E-14	-2.10485	0.0001699	-1.69569	1.05E-09	-2.25465	0.117697	-1.25304
8022674	CDH2	NM_001792	9.31E-17	2.33073	4.17E-06	1.91982	7.35E-05	1.67252	0.448694	1.11517
7996819	CDH3	NM_001793	6.27E-25	3.33687	1.92E-09	2.58038	2.96E-10	2.51195	0.234078	1.20613
8135341	CDHR3	NM_152750	1.54E-10	4.35523	3.40E-05	3.92805	0.0242687	1.96441	0.0237952	2.15012
8057463	CERKL	NM_201548	4.64E-08	2.0633	0.0007845	1.90312	0.11212	1.31994	0.262552	1.24713
7903920	CHI3L2	NM_001025199	8.77E-09	-2.08862	0.0035219	-1.70925	0.0054912	-1.59945	0.0208709	-1.55312
7923562	CHIT1	NM_003465	0.0251036	1.53955	0.0037843	2.29026	0.572066	1.15901	0.537339	1.19967

8022666	CHST9	NM_031422	2.70E-09	2.59483	0.00019	2.36695	0.11252	1.39512	0.18043	1.37338
7902702	CLCA2	NM_006536	1.36E-07	2.3679	0.0002057	2.41749	0.156589	1.35804	0.64602	1.11837
8092726	CLDN1	NM_021101	1.40E-16	3.2509	4.88E-07	2.73286	1.67E-06	2.41013	0.595297	1.11313
8082928	CLDN18	NM_001002026	4.67E-06	-2.09593	0.146762	-1.40346	0.0578004	-1.5056	0.77525	1.07174
7953749	CLEC4D	NM_080387	1.21E-08	-1.81527	6.22E-05	-1.83552	1.01E-05	-1.85848	2.40E-06	-2.11862
7960900	CLEC4E	NM_014358	9.41E-16	-3.18654	8.38E-08	-2.9824	1.97E-07	-2.65323	0.0002371	-2.15115
8068383	CLIC6	NM_053277	3.13E-10	2.34286	0.0006932	1.92635	0.0011464	1.78355	0.0510923	1.47541
8088866	CNTN3	NM_020872	1.77E-20	2.22765	1.39E-06	1.76667	2.75E-06	1.66323	0.0716821	1.24083
8148070	COL14A1	NM_021110	6.20E-28	3.99839	4.48E-14	3.66989	3.32E-14	3.33805	0.0062144	1.58636
8156783	COL15A1	NM_001855	9.83E-20	2.46827	9.86E-10	2.33489	1.25E-12	2.51697	0.0211328	1.37731
7936144	COL17A1	NM_000494	2.48E-16	2.32674	2.47E-06	1.97036	0.0008976	1.54438	0.423527	1.12396
8016646	COL1A1	NM_000088	1.26E-18	3.36927	2.08E-09	3.18579	4.93E-11	3.26233	0.328563	1.20735
8134263	COL1A2	NM_000089	3.02E-13	1.99258	3.24E-08	2.11972	3.58E-08	1.99384	0.39592	1.12277
8046922	COL3A1	NM_000090	1.64E-17	3.00617	2.02E-09	2.98643	4.71E-10	2.86105	0.576682	1.10682
8059905	COL6A3	NM_004369	7.98E-13	2.07158	1.94E-05	1.85267	3.35E-07	1.98454	0.561972	1.08901
8035517	COMP	NM_000095	6.10E-16	2.22033	2.04E-05	1.7961	0.0003089	1.57487	0.171818	1.21133
8091385	CP	NM_000096	7.76E-16	5.94355	1.63E-06	4.42676	1.51E-06	3.95875	0.0633685	1.79621
8083260	CPA3	NM_001870	5.37E-10	2.16116	0.000443	1.86723	0.0004541	1.77611	0.0104343	1.60117
7971444	CPB2	NM_001872	9.24E-10	-3.57655	0.0048663	-2.30674	0.0001441	-2.85283	0.0045135	-2.40623
7977409	CRIP1	NM_001311	6.07E-23	1.90668	1.56E-11	1.84121	7.43E-18	2.10678	3.17E-23	2.72526
8146967	CRISPLD1	NM_031461	3.03E-14	2.1714	1.50E-07	2.14553	0.0027195	1.48059	0.304646	1.16227
7997642	CRISPLD2	NM_031476	0.0007202	-1.40215	0.079165	-1.29296	9.78E-06	-1.83806	4.99E-10	-2.69345
7935535	CRTAC1	NM_018058	5.31E-18	-2.55718	0.0001446	-1.76048	0.0115171	-1.40939	0.835054	-1.03222
7914950	CSF3R	NM_156039	3.25E-22	-2.45324	7.72E-09	-2.07859	7.02E-09	-1.96682	9.64E-07	-1.89451
8086330	CSRNP1	NM_033027	1.74E-10	-2.06065	0.0657018	-1.34238	3.16E-08	-2.32503	0.0030095	-1.64476
7902290	CTH	NM_001902	2.38E-19	-1.90585	2.26E-09	-1.8195	2.04E-13	-2.00125	1.66E-09	-1.87572
8095005	CWH43	AK300495	5.84E-05	-2.26944	0.0003569	-2.92231	0.0037891	-2.2194	0.178038	-1.51542
7933194	CXCL12	NM_000609	1.19E-18	1.91833	6.44E-10	1.90075	1.40E-13	2.06173	0.0007012	1.42595
8114249	CXCL14	NM_004887	1.69E-19	2.30664	2.54E-10	2.27245	3.35E-11	2.21914	0.0337214	1.31602
8058905	CXCR1	NM_000634	2.63E-13	-1.82196	0.0001019	-1.56749	8.93E-11	-2.04402	5.91E-07	-1.83854
8048227	CXCR2	NM_001557	3.31E-09	-1.90052	0.008206	-1.50681	2.65E-07	-2.12485	0.0190832	-1.45921
8166671	CXorf22	NM_152632	7.05E-08	2.49244	0.0020533	2.12581	0.203106	1.32875	0.388803	1.24264
8166690	CXorf59	NM_173695	9.45E-08	3.01825	0.0013432	2.61156	0.196427	1.42288	0.162376	1.53902
8067140	CYP24A1	NM_000782	4.52E-10	3.12768	6.47E-05	2.85876	0.053421	1.58578	0.66579	1.123

8141328	CYP3A5	NM_000777	8.01E-12	-2.17486	0.0502291	-1.3661	1.15E-06	-2.07918	0.471221	-1.12644
7970954	DCLK1	NM_004734	1.33E-19	2.64015	1.70E-08	2.31322	5.62E-09	2.22741	0.188657	1.21667
8149116	DEFA3	NM_005217	6.70E-16	-4.13675	1.45E-07	-3.6844	3.20E-08	-3.55646	0.0006729	-2.3653
8149126	DEFA3	NM_005217	6.70E-16	-4.13675	1.45E-07	-3.6844	3.20E-08	-3.55646	0.0006729	-2.3653
8149137	DEFA3	NM_005217	1.46E-15	-3.74414	1.62E-07	-3.40255	5.96E-08	-3.22383	0.0009236	-2.20375
8048541	DES	NM_001927	9.87E-09	2.08729	0.0470996	1.43867	0.277133	1.20067	0.802795	1.04856
7980485	DIO2	NM_013989	2.14E-23	2.46275	3.68E-08	1.96198	8.91E-07	1.73363	0.0581708	1.26321
7959696	DNAH10	NM_207437	4.96E-07	2.12319	0.0037779	1.87451	0.284166	1.23648	0.321599	1.24848
7959681	DNAH10	NM_207437	6.61E-08	2.93364	0.0017236	2.46436	0.222621	1.37738	0.307624	1.35321
8131719	DNAH11	NM_003777	1.39E-07	2.50543	0.0046296	2.04016	0.161452	1.38064	0.243427	1.35445
8088299	DNAH12	NM_178504	5.47E-09	3.78701	0.0007398	3.02862	0.121599	1.58948	0.153686	1.61983
8088315	DNAH12	NM_178504	5.75E-09	3.28666	0.0011393	2.59944	0.116682	1.52246	0.290673	1.37624
8088322	DNAH12	NM_198564	3.49E-09	2.89415	0.0012271	2.3039	0.107499	1.46131	0.206124	1.39973
8000034	DNAH3	NM_017539	1.54E-07	2.61203	0.0018169	2.28323	0.176808	1.38592	0.455719	1.2255
8111019	DNAH5	NM_001369	0.0001319	2.01012	0.0118559	1.96012	0.798068	1.06458	0.746031	-1.09365
8043055	DNAH6	NM_001370	2.11E-09	2.45157	0.005036	1.82216	0.154047	1.32187	0.159766	1.36474
8043043	DNAH6	NM_001370	4.29E-11	3.23941	0.0001508	2.62129	0.0452791	1.58927	0.0598615	1.63518
8043059	DNAH6	NM_001370	1.64E-09	3.25712	0.0006796	2.59718	0.0339148	1.7236	0.138826	1.53429
8043071	DNAH6	NM_001370	7.33E-09	2.76411	0.0025714	2.13984	0.0731798	1.51239	0.129055	1.48521
8057821	DNAH7	NM_018897	6.83E-10	2.73173	0.0003928	2.28741	0.0864724	1.43973	0.175856	1.38365
8004957	DNAH9	NM_001372	2.66E-08	2.7902	0.0006916	2.4692	0.188909	1.37539	0.240326	1.37952
8154892	DNAI1	NM_012144	3.93E-07	2.30305	0.0024746	2.05724	0.301419	1.25205	0.391019	1.23459
7985147	DNAJA4	NM_018602	3.87E-09	2.07157	0.0001932	1.94354	0.103216	1.30218	0.374373	1.17618
8118061	DPCR1	NM_080870	1.50E-12	-2.04767	0.0187036	-1.3956	0.0003782	-1.59765	0.252687	-1.18293
8022692	DSC3	NM_024423	4.44E-08	2.96852	0.0487761	1.7528	0.0049772	2.09771	0.263786	1.3915
8094533	DTHD1	NM_001136536	2.33E-10	3.64557	0.0002697	2.89676	0.0358126	1.74844	0.0702814	1.7223
7983413	DUOX1	NM_017434	1.82E-15	-2.14839	1.26E-08	-2.17846	8.13E-07	-1.85075	2.49E-05	-1.80358
8115831	DUSP1	NM_004417	2.05E-06	-1.81492	0.177461	-1.27648	1.62E-06	-2.27337	0.459493	-1.14937
8081503	DZIP3	NM_014648	7.05E-15	2.40857	7.69E-05	1.8668	0.0001513	1.73403	0.0100745	1.51954
8162404	ECM2	NM_001393	9.95E-16	2.09508	1.29E-10	2.34158	1.17E-08	1.99001	1.56E-07	2.03517
7972157	EDNRB	NM_001122659	9.73E-09	-1.80399	0.0157292	-1.42676	4.04E-07	-2.02248	0.233057	-1.19934
8150691	EFCAB1	NM_024593	3.16E-08	3.62207	0.000575	3.18281	0.151578	1.5513	0.169089	1.60913
8085732	EFHB	NM_144715	3.19E-06	2.19999	0.0108277	1.87047	0.308529	1.25747	0.310229	1.29428
8120222	EFHC1	NR_033327	2.56E-09	2.02497	0.0009163	1.75654	0.047874	1.35961	0.0870454	1.34966

8172225	EFHC2	NM_025184	1.47E-10	2.34084	0.0002165	2.01733	0.0302117	1.45471	0.129778	1.34335
7932598	ENKUR	NM_145010	3.58E-07	2.86659	0.0010466	2.67659	0.324768	1.30913	0.465474	1.25292
8081081	EPHA3	NM_005233	7.54E-16	2.17029	4.00E-10	2.36425	3.19E-05	1.6707	0.0514952	1.3061
8026631	F2RL3	NM_003950	4.00E-19	-2.31791	1.76E-09	-2.208	1.90E-13	-2.49075	3.36E-07	-1.99453
8176193	F8	NM_000132	2.89E-06	-1.8937	1.79E-05	-2.36875	0.0189317	-1.53423	0.216083	-1.28876
8104570	FAM105A	NM_019018	2.56E-19	-2.05775	7.56E-07	-1.72799	1.12E-08	-1.80058	0.0598295	-1.23516
8049990	FAM150B	NM_001002919	9.28E-12	-1.93838	2.39E-06	-1.92941	1.56E-09	-2.20162	1.38E-07	-2.15891
7985398	FAM154B	AK304339	9.12E-09	3.11023	0.0003726	2.75555	0.0799151	1.57508	0.117161	1.58241
7900635	FAM183A	NM_001101376	1.56E-06	2.11866	0.0020792	2.01361	0.244155	1.27331	0.161024	1.38931
8106950	FAM81B	NM_152548	8.43E-09	2.59203	0.0004295	2.31513	0.159789	1.35656	0.157325	1.4143
8062571	FAM83D	NM_030919	2.47E-12	2.35278	0.0062362	1.59871	0.0648088	1.3371	0.885481	1.02582
7931281	FANK1	NM_145235	1.64E-09	2.52066	9.50E-05	2.36834	0.106557	1.38198	0.301149	1.26335
8056257	FAP	NM_004460	2.63E-15	2.77207	2.38E-10	3.23612	2.79E-07	2.36982	0.012826	1.58502
8019392	FASN	NM_004104	5.37E-19	-2.73024	1.86E-09	-2.58279	4.13E-06	-1.93043	0.000285	-1.78583
7914075	FCN3	NM_003665	2.36E-16	-4.60575	2.96E-05	-2.94556	8.37E-11	-4.93693	0.0016091	-2.3206
8103311	FGA	NM_000508	4.35E-06	-2.0059	0.0384067	-1.57551	0.0001129	-2.20692	0.0012783	-2.10005
7972650	FGF14	NM_175929	8.70E-16	2.47398	8.21E-05	1.8526	0.0015315	1.57487	0.0072004	1.54302
7939052	FIBIN	NM_203371	2.52E-19	-2.57495	3.38E-10	-2.52767	1.31E-11	-2.53028	0.0123631	-1.44643
8171427	FIGF	NM_004469	2.54E-08	-2.80055	0.135342	-1.48305	4.72E-05	-2.73827	0.823325	1.06311
8125919	FKBP5	NM_001145775	4.51E-10	-2.47929	8.44E-07	-2.83396	1.24E-06	-2.57163	3.56E-06	-2.7667
7970763	FLT1	NM_002019	2.62E-16	-2.04427	6.92E-05	-1.61891	5.18E-07	-1.76237	6.02E-06	-1.77524
7919314	FMO5	NM_001461	3.66E-17	-3.03422	1.33E-07	-2.64726	2.58E-06	-2.21236	0.0024307	-1.76715
8123104	FNDC1	NM_032532	2.93E-25	2.89275	1.31E-12	2.70777	1.01E-10	2.28847	1.38E-05	1.84254
8029693	FOSB	NM_006732	0.426336	1.19142	0.009573	2.34024	0.270313	-1.39261	0.052776	1.93386
8038899	FPR1	NM_002029	1.17E-15	-2.34165	4.93E-05	-1.82358	1.42E-08	-2.20103	5.04E-05	-1.86789
8167560	GAGE12C	NM_001098408	0.0003403	-1.75852	0.0012406	-2.11813	0.0032551	-1.87561	0.379854	-1.23373
8078155	GALNTL2	NM_054110	5.56E-08	-2.13966	2.02E-06	-2.65334	4.88E-05	-2.14455	3.98E-09	-3.5886
7946641	GALNTL4	NM_198516	4.70E-25	-2.09232	8.26E-10	-1.80954	1.30E-12	-1.89989	1.43E-05	-1.53159
7917503	GBP3	NM_018284	0.0002265	1.52765	0.0018814	1.69192	0.0002474	1.77558	8.51E-05	2.0083
8027002	GDF15	NM_004864	1.39E-06	1.72632	9.13E-06	2.09404	0.0191764	1.42383	0.640103	-1.08241
8151816	GEM	NM_005261	1.42E-11	2.14062	2.20E-11	3.04717	0.0099087	1.45844	0.229473	1.21826
7983704	GLDN	NM_181789	2.11E-07	-2.10123	0.0014369	-1.93623	9.88E-05	-2.11395	0.0633473	-1.48823
7959834	GLT1D1	NM_144669	2.00E-19	-1.92494	5.10E-09	-1.80658	3.98E-15	-2.13584	2.73E-10	-1.95471
8162117	GOLM1	NM_016548	2.68E-18	2.23931	1.45E-09	2.19516	4.31E-08	1.91703	0.0318816	1.32199

8126820	GPR110	NM_153840	1.03E-10	2.33813	0.0039703	1.71122	0.00075	1.78883	0.802228	1.04934
8037614	GPR4	NM_005282	2.39E-18	-2.17372	2.80E-06	-1.77668	2.95E-11	-2.16658	7.46E-07	-1.88578
7996100	GPR97	NM_170776	1.28E-17	-2.0123	4.01E-06	-1.68665	2.11E-13	-2.22407	8.23E-12	-2.3005
8109333	GPX3	NM_002084	7.72E-34	-2.70404	2.86E-16	-2.44117	1.18E-23	-2.86732	1.26E-09	-1.94289
7955578	GRASP	NM_181711	2.68E-23	-2.43188	4.45E-08	-1.93882	9.37E-16	-2.55041	9.82E-08	-1.95487
7982377	GREM1	NM_013372	2.09E-10	1.87814	8.46E-07	2.03012	0.0002024	1.62602	0.689204	1.05986
8109383	GRIA1	NM_000827	2.63E-13	-2.64426	2.86E-06	-2.42304	1.05E-06	-2.34595	0.289564	-1.22538
8127072	GSTA1	NM_145740	2.16E-10	3.24978	4.70E-06	3.41067	0.0727759	1.54284	0.125266	1.51922
8127065	GSTA2	NM_000846	1.59E-06	2.05958	0.0015821	1.9984	0.0873707	1.40819	0.210037	1.3273
8080964	GXYLT2	NM_001080393	8.22E-22	2.37262	2.93E-11	2.2994	7.09E-12	2.21216	2.20E-08	2.04998
8152617	HAS2	NM_005328	1.35E-11	2.9618	3.03E-09	4.01446	0.0001592	2.21392	0.210505	1.34067
7991766	HBA1	NM_000558	0.108406	1.27915	0.0001147	2.43134	0.037186	1.54751	1.60E-08	3.99258
7991762	HBA2	NM_000517	0.108406	1.27915	0.0001147	2.43134	0.037186	1.54751	1.60E-08	3.99258
7946033	HBB	NM_000518	0.0669491	1.36367	0.0001587	2.60865	0.010696	1.80677	7.37E-09	4.78569
8057898	HECW2	NM_020760	9.10E-09	-2.33873	0.156199	-1.34776	5.73E-05	-2.21292	0.575933	-1.13015
8097628	HHIP	NM_022475	9.60E-09	-2.37527	0.0218946	-1.63893	0.0163508	-1.61164	0.4344	-1.19061
8081488	HHLA2	NM_007072	2.25E-14	3.04448	5.28E-05	2.28703	0.0001932	2.01553	0.0913131	1.42459
8029784	HIF3A	NM_152794	1.76E-21	-2.09919	1.75E-11	-2.0774	4.20E-13	-2.08154	0.0003118	-1.48
7908204	HMCN1	NM_031935	4.38E-25	2.43463	6.85E-12	2.24247	1.81E-13	2.23893	4.15E-05	1.62212
7997188	HP	NM_005143	0.0085911	-2.0413	0.0855758	-1.98834	0.515865	-1.26955	0.632463	-1.21952
8103769	HPGD	NM_000860	0.0005442	-1.84329	0.769863	1.07835	0.0012452	-2.17493	0.407974	1.24902
7935613	HPSE2	NM_021828	9.08E-14	2.05202	3.54E-05	1.75446	0.0020381	1.46647	0.0206532	1.38114
8097335	HSPA4L	NM_014278	2.39E-18	3.07047	2.34E-07	2.51401	3.77E-05	1.95252	0.382558	1.17004
8002446	HYDIN	NM_032821	5.28E-08	2.481	0.0012756	2.17494	0.124924	1.40182	0.191876	1.38312
8002470	HYDIN	NM_032821	2.17E-08	2.47312	0.0012721	2.11956	0.132368	1.37732	0.255017	1.31429
8002481	HYDIN	NM_032821	6.49E-09	2.48629	0.0004552	2.20905	0.102938	1.39905	0.183373	1.36235
8002492	HYDIN	NM_032821	2.15E-10	3.07695	0.000131	2.63748	0.0424564	1.59698	0.131511	1.48035
8061564	ID1	NM_181353	1.34E-19	-2.37465	0.0002841	-1.59971	1.40E-11	-2.31584	3.81E-06	-1.87641
7902541	IFI44L	NM_006820	0.0006987	1.65169	0.0345584	1.58223	0.0001155	2.18455	0.0022544	2.00318
7965873	IGF1	NM_001111283	2.73E-07	2.16342	0.0118061	1.72519	0.0002293	2.10026	0.409877	-1.20274
7981722	IGHA1	AK128476	1.01E-09	2.35004	0.0103211	1.66669	4.51E-07	2.58114	0.0813615	1.43323
7981740	IGHA1	BC073771	5.47E-09	2.30775	0.0130654	1.66296	8.43E-07	2.58892	0.209491	1.30515
7995263	IGHA1	AF067420	4.28E-09	2.52278	0.0073316	1.82974	2.20E-06	2.72338	0.124678	1.4307
7981724	IGHD	BC021276	6.47E-10	2.42413	0.0122945	1.66553	3.20E-07	2.67461	0.0671516	1.47269

7981718	IGHM	BC020240	8.26E-10	2.26744	0.0248715	1.52946	3.43E-07	2.49272	0.100212	1.38172
7981601	IGHV4-31	AK301335	2.02E-09	2.24615	0.0049809	1.7185	5.05E-07	2.48995	0.0964651	1.3933
8100827	IGJ	NM_144646	3.62E-11	2.39741	0.0069696	1.65511	1.43E-07	2.53101	0.0062602	1.70197
8053690	IGK@	BC032451	2.66E-09	2.65413	0.0072548	1.87583	7.04E-07	2.98738	0.0719822	1.54786
8043360	IGK@	BC030813	1.50E-09	2.59876	0.0010916	2.09407	2.22E-07	3.0112	0.0553435	1.56481
8043449	IGK@	BC030813	9.36E-10	2.9163	0.0014324	2.21949	2.30E-07	3.37804	0.109672	1.51097
8043431	IGKC	AF113887	1.22E-09	2.73304	0.0038663	1.97848	8.87E-07	2.9777	0.0239433	1.73884
8043433	IGKC	BC110394	1.51E-07	2.55036	0.0145549	1.87277	0.0001001	2.53757	0.197746	1.40866
8043436	IGKC	BC073772	1.75E-06	2.78428	0.0399949	1.8889	8.69E-05	3.11316	0.222944	1.47953
8043438	IGKC	BC093097	1.65E-08	3.2505	0.013769	2.08971	1.99E-06	3.81277	0.0962303	1.67571
8043459	IGKC	ENST0000039027	2.63E-10	2.14225	0.0029772	1.66462	5.06E-07	2.24973	0.0306604	1.46852
8043476	IGKC	ENST0000039027	1.38E-09	2.86859	0.004848	2.01274	6.87E-07	3.1903	0.0592037	1.62464
8043470	IGKV3D-11	ENST0000039025	1.88E-09	3.08537	0.0027713	2.23117	7.18E-07	3.48753	0.0797957	1.62553
8043423	IGKV4-1	ENST0000039024	7.99E-08	1.94801	0.006918	1.62145	7.21E-06	2.12125	0.173073	1.28688
7981730	IGLJ3	AB001736	7.96E-10	2.36989	0.0106981	1.66517	3.50E-07	2.61399	0.0621743	1.47196
8071642	IGLV6-57	ENST0000039028	8.86E-07	1.91957	0.0083006	1.66056	5.71E-05	2.05618	0.420262	1.17354
8174598	IL13RA2	NM_000640	6.23E-19	3.19361	6.60E-06	2.24418	9.08E-08	2.44167	0.440941	1.15085
8044035	IL18R1	NM_003855	7.11E-19	-2.54531	3.30E-09	-2.39022	5.79E-15	-2.99091	1.38E-10	-2.6971
8044049	IL18RAP	NM_003853	2.69E-18	-2.78915	4.75E-08	-2.44835	9.44E-11	-2.70132	6.76E-07	-2.32341
8043981	IL1R2	NM_004633	1.93E-21	-5.17368	3.53E-11	-4.92756	2.65E-16	-6.50183	2.51E-14	-7.00314
8044021	IL1RL1	NM_016232	8.26E-12	-2.80872	0.0005897	-2.08635	1.33E-11	-4.02387	2.76E-06	-2.881
8085062	IL5RA	NM_000564	5.02E-11	2.48474	4.25E-05	2.25157	0.0222777	1.5095	0.0235239	1.58566
8104901	IL7R	NM_002185	2.27E-07	-2.10851	0.113915	-1.38698	0.0003691	-1.98928	0.441598	-1.17992
8132092	INMT	NM_006774	1.01E-18	-2.96836	1.54E-06	-2.26256	5.72E-11	-2.86218	0.001323	-1.74926
8142646	IQUB	NM_178827	4.95E-08	2.38971	0.0048919	1.91044	0.175479	1.3299	0.274309	1.29659
7904761	ITGA10	NM_003637	9.89E-25	-2.31749	2.67E-12	-2.19701	1.72E-18	-2.56898	2.96E-11	-2.16963
8131666	ITGB8	NM_002214	1.10E-13	2.06009	8.27E-06	1.84741	3.89E-05	1.68221	0.0063107	1.47058
7921690	ITLN1	NM_017625	0.0005353	-2.40835	0.250457	-1.53108	0.898572	-1.04443	0.700926	1.15956
7921702	ITLN2	NM_080878	1.36E-19	-2.2831	6.57E-05	-1.64045	1.30E-06	-1.74765	0.257119	1.15459
7969166	KCNRG	NM_173605	9.80E-09	2.03109	0.0012405	1.77554	0.0472735	1.38053	0.162669	1.2915
7903592	KIAA1324	NM_020775	2.24E-05	2.06872	0.0251586	1.7489	0.0140287	1.76116	0.072719	1.59294
7943376	KIAA1377	NM_020802	9.29E-14	2.37149	0.000208	1.82842	0.0011114	1.62754	0.0013322	1.71841
8163002	KLF4	NM_004235	1.42E-07	-1.85394	0.571252	-1.09966	1.48E-06	-2.15049	0.829419	1.03835
8174654	KLHL13	NM_033495	1.30E-23	2.47122	1.51E-09	2.10403	3.52E-05	1.58102	0.0107899	1.37

8015337	KRT15	NM_002275	1.27E-10	2.91815	0.0020435	2.07536	0.0788566	1.46298	0.863971	1.04264
8015387	KRT17	NM_000422	9.52E-11	2.08986	2.06E-07	2.35981	0.0438423	1.34781	0.544152	1.10632
7963427	KRT5	NM_000424	4.25E-13	5.56048	0.0001588	3.52798	0.0117026	2.1556	0.369968	1.35872
7963410	KRT6C	NM_173086	2.90E-07	2.08169	0.141513	1.35217	0.267131	1.23325	0.900964	1.02688
8121949	LAMA2	NM_000426	8.41E-06	-1.50746	0.0009722	-1.55994	3.54E-06	-1.79266	4.93E-09	-2.32895
8158167	LCN2	NM_005564	1.72E-07	2.15061	0.0022266	1.91196	0.264299	1.24096	0.507891	-1.15552
8092707	LEPREL1	NM_018192	4.29E-10	-2.15521	0.0296649	-1.4611	0.0100048	-1.51426	0.841556	-1.03675
8112476	LOC100133280	ENST0000036079	2.34E-13	-2.02631	1.46E-07	-2.06548	6.67E-08	-1.98963	9.88E-07	-2.01368
8105935	LOC100133280	ENST0000036079	7.67E-13	-2.19524	3.73E-07	-2.22283	2.18E-07	-2.1209	8.47E-07	-2.23539
8105987	LOC100133280	ENST0000036079	2.09E-13	-2.19353	3.11E-07	-2.18563	8.39E-09	-2.26716	1.45E-08	-2.48116
8105989	LOC100133280	ENST0000036079	4.24E-13	-2.19525	2.38E-07	-2.23143	1.18E-07	-2.13774	6.24E-07	-2.23543
8112519	LOC100133280	ENST0000036079	3.19E-13	-2.18525	2.13E-07	-2.21808	9.23E-09	-2.26883	2.86E-08	-2.43976
8001104	LOC100290146	AK289373	4.21E-09	2.33828	0.0095664	1.70804	6.62E-07	2.63309	0.115965	1.4
7981737	LOC100291056	ENST0000039063	2.01E-07	2.56185	0.0162305	1.87163	4.20E-05	2.71252	0.285915	1.33397
7981728	LOC100293211	ENST0000039060	9.57E-10	2.53649	0.0113392	1.73062	4.43E-07	2.8073	0.0769024	1.48802
8001102	LOC440248	ENST0000038303	2.05E-09	2.00441	0.0074195	1.55798	9.95E-07	2.14349	0.13086	1.29562
8053735	LOC642838	XM_001714030	6.54E-08	1.90197	0.0090825	1.56305	7.30E-06	2.05312	0.0436119	1.43125
8076113	LOC646851	NM_001013647	4.97E-09	2.27013	0.0016212	1.88564	0.0887332	1.36747	0.254042	1.26681
8043468	LOC652493	ENST0000039024	4.29E-08	2.95271	0.0173485	1.96391	1.22E-05	3.19818	0.075015	1.6894
8043474	LOC652493	ENST0000039024	6.97E-10	2.64851	0.0009776	2.10583	1.05E-06	2.80857	0.0510425	1.5762
8077499	LOH3CR2A	AF086709	1.60E-14	-2.52797	0.0016847	-1.69482	4.22E-10	-2.72555	0.0013866	-1.74994
7903214	LPPR4	NM_014839	1.95E-26	2.49477	1.13E-14	2.49787	2.01E-13	2.21858	1.86E-05	1.65137
8056611	LRP2	NM_004525	8.34E-10	-2.35987	0.0022793	-1.83946	0.048626	-1.43421	0.453426	-1.16683
8135218	LRRC17	NM_005824	2.13E-22	2.69413	1.25E-09	2.33749	7.43E-10	2.21273	0.0178107	1.39348
8008040	LRRC46	NM_033413	1.10E-07	2.89672	0.0003356	2.83647	0.131392	1.49034	0.224509	1.43711
8005289	LRRC48	NM_001130090	7.96E-07	2.40992	0.0013602	2.29261	0.198581	1.35487	0.15346	1.46405
7997556	LRRC50	NM_178452	1.60E-07	3.03222	0.001315	2.67746	0.215016	1.4142	0.31678	1.37159
7957433	LRRIQ1	NM_032165	1.27E-09	3.2258	0.0001904	2.81462	0.146406	1.44177	0.1781	1.46694
8077366	LRRN1	NM_020873	1.53E-16	2.44432	1.68E-07	2.21622	0.0009945	1.57171	0.139588	1.25464
8064894	LRRN4	NM_152611	2.13E-12	-2.11703	0.0002213	-1.74678	0.0004419	-1.62952	0.0112093	-1.48489
7980152	LTBP2	NM_000428	1.93E-08	1.62533	5.27E-08	2.00236	0.0043154	1.38652	0.0012051	1.5221
7965403	LUM	NM_002345	5.48E-15	1.94396	2.03E-09	2.07663	1.19E-06	1.71142	0.0001019	1.61699
8138466	MACC1	NM_182762	2.83E-14	2.00495	1.66E-07	1.97661	1.16E-06	1.78736	0.0002744	1.62372
8058462	MDH1B	NM_001039845	1.02E-07	2.51139	0.0014967	2.21303	0.176043	1.36183	0.354838	1.26914

7900365	MFSD2A	NM_001136493	1.32E-16	-2.44527	7.68E-07	-2.11205	2.86E-06	-1.91645	0.0007494	-1.6861
8136662	MGAM	NM_004668	7.69E-29	-4.16624	6.48E-12	-3.24361	1.12E-21	-4.94918	8.58E-15	-4.08525
7971661	MIR15A	NR_029485	9.80E-06	1.75827	0.0997764	1.3558	2.06E-06	2.29163	0.0033572	1.76648
7911339	MIR1977	NR_031741	0.0201214	-1.45125	0.0035175	-2.00277	0.0179999	-1.677	0.722512	1.09101
8165698	MIR1977	NR_031741	0.0201214	-1.45125	0.0035175	-2.00277	0.0179999	-1.677	0.722512	1.09101
8083494	MME	NM_007288	2.36E-10	-2.20134	0.0013311	-1.76773	6.53E-05	-1.93065	0.647961	-1.08695
7951271	MMP1	NM_002421	1.70E-11	6.59658	0.002169	3.3732	0.0057304	2.73901	0.838719	1.08665
7951259	MMP10	NM_002425	2.79E-07	1.96916	0.0001207	2.09487	0.413064	1.15327	0.825486	1.04433
7951309	MMP13	NM_002427	2.88E-13	3.18882	0.0001159	2.37088	0.000337	2.09208	0.723996	1.08424
8151684	MMP16	AL136588	1.24E-13	2.10702	1.03E-07	2.14941	9.87E-06	1.78516	0.220338	1.19432
7951217	MMP7	NM_002423	3.79E-19	7.27509	1.09E-06	4.43702	5.27E-07	4.11785	0.137699	1.58411
7989146	MNS1	NM_018365	4.88E-11	2.6079	0.0001077	2.23961	0.0212446	1.54776	0.0366524	1.56393
8157632	MORN5	NM_198469	8.27E-07	2.10271	0.0013089	2.02321	0.268876	1.24726	0.181579	1.35236
7940333	MS4A15	NM_001098835	1.73E-12	-2.55932	0.0001114	-2.07297	2.97E-05	-2.07162	0.268301	-1.23843
7940226	MS4A2	NM_000139	6.38E-12	2.24578	0.0019302	1.67423	1.52E-05	1.95472	0.0149166	1.52114
7940323	MS4A8B	NM_031457	5.53E-09	4.23663	0.0001436	3.89362	0.110687	1.67865	0.117074	1.7771
7927529	MSMB	NM_002443	7.53E-07	3.41975	0.0343841	2.13587	0.267049	1.44122	0.0583449	2.02677
7995806	MT1A	NM_005946	0.0001851	-1.61307	0.048335	-1.44623	0.0001135	-1.96177	0.0003918	-2.00856
7995803	MT1JP	AF348994	5.81E-09	-2.00188	0.0005379	-1.81124	1.34E-07	-2.34122	1.32E-05	-2.1932
7995787	MT1M	NM_176870	2.45E-10	-3.01874	8.57E-05	-2.6736	2.46E-08	-3.71361	6.92E-06	-3.25228
7995838	MT1X	NM_005952	1.31E-06	-1.94985	0.0006346	-1.98885	5.23E-07	-2.57645	6.36E-07	-2.88895
7995783	MT2A	NM_005953	1.77E-05	-1.72178	0.038636	-1.46318	8.08E-05	-1.96998	0.0001473	-2.08885
8095362	MT2A	NM_005953	7.69E-06	-1.70875	0.0215281	-1.49167	5.74E-05	-1.92292	6.62E-05	-2.08004
8090180	MUC13	NM_033049	0.0015151	1.44154	2.18E-07	2.46633	0.54187	1.0994	0.987622	-1.00273
8033674	MUC16	NM_024690	8.15E-06	2.034	0.0419441	1.59951	0.107018	1.40829	0.297979	1.28319
8092978	MUC4	NM_018406	4.43E-07	2.25259	0.0087197	1.84014	0.0476479	1.52635	0.855417	1.04472
7937612	MUC5B	NM_002458	1.03E-07	2.99236	0.0009073	2.68943	0.0910191	1.58384	0.854218	1.05791
8171172	MXRA5	NM_015419	1.04E-20	2.36413	5.58E-12	2.44243	6.41E-12	2.27146	0.002503	1.47542
8122202	MYB	NM_001130173	2.47E-10	2.0056	0.0033904	1.58136	0.001977	1.56289	0.0132305	1.49536
7933084	NAMPT	NM_005746	8.88E-06	-1.64922	0.0403322	-1.39806	7.83E-06	-1.98678	4.94E-05	-2.01629
8147244	NECAB1	NM_022351	3.96E-25	-3.34315	1.79E-07	-2.25219	5.74E-08	-2.18167	0.192061	-1.2272
8085867	NEK10	NM_199347	1.95E-08	2.55979	0.0012219	2.18128	0.162208	1.35997	0.261614	1.32154
8082643	NEK11	NM_024800	2.57E-12	2.2829	4.22E-06	2.1706	0.0027029	1.58216	0.006698	1.59625
7971757	NEK5	NM_199289	1.37E-07	2.22315	0.0018535	1.97932	0.203541	1.28984	0.295167	1.26696

7962455	NELL2	NM_006159	6.69E-09	2.22315	0.0003623	2.03039	0.0742757	1.38113	0.0375236	1.53079
7925320	NID1	NM_002508	2.96E-09	-1.68673	0.0098756	-1.38344	5.30E-07	-1.81293	5.06E-08	-2.08461
8114354	NME5	NM_003551	5.78E-09	2.17223	0.0009025	1.88858	0.0747845	1.36555	0.27686	1.23902
7905606	NPR1	NM_000906	4.65E-23	-2.35882	2.15E-06	-1.73734	6.83E-09	-1.88009	0.0001456	-1.57861
8104746	NPR3	NM_000908	3.89E-09	-2.03438	0.02404	-1.47412	3.13E-09	-2.6505	0.0202238	-1.51597
7955589	NR4A1	NM_002135	0.00674	-1.46562	0.992142	-1.00204	0.0001931	-2.0606	0.272615	-1.26705
8055952	NR4A2	NM_006186	0.121198	-1.30196	0.410547	1.2298	0.0026164	-2.02268	0.483013	-1.20151
8162373	OGN	NM_033014	2.55E-13	2.23358	1.69E-06	2.11383	5.56E-07	2.06121	0.0012443	1.67803
7961142	OLR1	NM_002543	0.948361	1.01104	0.0491035	1.6422	0.184583	1.35998	0.0037578	2.14902
7953873	OVOS	BX647938	7.63E-12	1.9717	0.0030058	1.51456	7.34E-08	2.03742	0.0011417	1.60805
7961026	OVOS	BX647938	6.22E-12	2.02654	0.0058234	1.49018	1.44E-07	2.05229	0.0011368	1.63568
8083447	P2RY1	NM_002563	1.26E-27	-2.7066	8.18E-12	-2.32026	1.33E-14	-2.43129	2.76E-05	-1.67926
8123303	PACRG	NM_152410	9.37E-08	2.06665	0.0010798	1.90254	0.185075	1.26835	0.202331	1.29481
7947512	PAMR1	NM_015430	2.38E-12	2.07106	1.27E-09	2.50519	7.30E-11	2.49608	7.61E-05	1.835
7928944	PAPSS2	NM_004670	7.57E-21	-2.21184	1.36E-05	-1.64819	3.62E-10	-1.97562	0.0052418	-1.39015
8122222	PDE7B	NM_018945	3.67E-21	2.12102	4.36E-08	1.81852	1.74E-12	2.07091	0.0049625	1.36466
8095080	PDGFRA	NM_006206	2.28E-18	-2.1206	2.46E-08	-1.95313	1.02E-11	-2.1542	1.36E-09	-2.14481
8149725	PEBP4	NM_144962	6.24E-12	-2.75171	0.0002016	-2.17391	0.0059685	-1.69104	0.347535	-1.22281
8126324	PGC	NM_002630	2.35E-05	-2.30002	8.00E-05	-3.14525	0.0110368	-1.95999	0.131306	-1.56697
8146957	PI15	NM_015886	1.00E-07	2.19765	0.0059621	1.79701	0.0219865	1.5663	0.205656	-1.32166
7951672	PIH1D2	NM_138789	1.66E-06	2.0188	0.0051346	1.81459	0.133395	1.33981	0.156956	1.36563
8136839	PIP	NM_002652	2.57E-05	2.08388	0.005502	2.02947	0.361072	1.23716	0.495822	1.19631
8147891	PKHD1L1	NM_177531	4.53E-07	-1.84643	1.68E-06	-2.3594	0.0019432	-1.65466	0.0487048	-1.43252
8081890	PLA1A	NM_015900	3.55E-10	-2.08671	0.0042113	-1.61219	1.84E-06	-2.11081	7.31E-06	-2.20553
7967034	PLA2G1B	NM_000928	3.33E-13	-2.98296	0.0004729	-2.09095	0.000231	-2.04903	0.176347	-1.34092
7987815	PLA2G4F	NM_213600	2.44E-09	-2.21362	5.74E-05	-2.16813	0.0321085	-1.45429	0.396656	-1.18126
7957514	PLEKHG7	NM_001004330	8.64E-09	2.02836	0.0012328	1.77013	0.114791	1.28966	0.350523	1.18509
8001547	PLLP	NM_015993	1.81E-20	-2.16636	6.73E-05	-1.56771	8.91E-09	-1.84192	0.177608	-1.16844
8061883	PLUNC	NM_130852	2.99E-05	3.71962	0.0185815	2.94434	0.400373	1.42403	0.607994	1.2757
7971077	POSTN	NM_006475	4.61E-09	2.52239	0.0009297	2.11935	0.0206136	1.61721	0.0613126	-1.54968
8088813	PROK2	NM_001126128	1.80E-16	-2.31316	5.28E-07	-2.04603	2.44E-13	-2.71403	3.10E-08	-2.28991
8099476	PROM1	NM_006017	1.06E-08	4.14336	6.18E-05	4.23058	0.056572	1.86479	0.330813	1.43041
8102468	PRSS12	NM_003619	7.17E-15	2.69892	1.75E-07	2.57385	0.0002608	1.81956	3.19E-05	2.16907
8136807	PRSS2	NM_002770	0.001109	1.6105	0.0002425	2.21698	0.746333	-1.06569	0.869786	1.0371

8149551	PSD3	NM_015310	1.97E-25	2.22369	4.94E-11	1.98368	1.16E-09	1.78607	3.05E-06	1.63748
7904293	PTGFRN	NM_020440	1.01E-23	2.1093	2.53E-11	1.97815	8.69E-11	1.83963	0.0634371	1.20658
7922976	PTGS2	NM_000963	0.0080758	1.39216	2.08E-05	2.21957	0.30621	1.18885	0.0550889	1.44429
7964872	PTPRB	NM_001109754	7.44E-17	-2.53825	3.30E-05	-1.90038	1.40E-10	-2.56591	0.0251237	-1.4266
8083594	PTX3	NM_002852	0.0057748	-1.60822	0.0862783	-1.5439	0.055679	-1.5635	0.0015122	-2.32524
8138504	RAPGEF5	NM_012294	7.91E-12	-2.02209	0.0002095	-1.71894	2.04E-07	-2.03666	0.0322503	-1.38015
8165672	RFC1	L23320	6.91E-05	-1.91617	5.75E-05	-2.64604	0.0122677	-1.73589	0.0819749	-1.53895
8151999	RGS22	NM_015668	2.20E-10	2.5235	0.0001047	2.25019	0.0569739	1.4357	0.1048	1.41594
8142079	RINT1	NM_021930	9.08E-08	2.3996	0.0007299	2.22833	0.160033	1.35473	0.351634	1.25498
7973110	RNASE2	NM_002934	2.12E-16	-2.34604	1.21E-07	-2.16594	4.40E-07	-1.9695	1.49E-07	-2.22251
8081001	ROBO2	NM_002942	1.03E-06	1.80186	1.35E-05	2.1626	0.0650278	1.34403	0.0003745	1.91847
8104492	ROPN1L	NM_031916	6.23E-05	2.10606	0.0149037	1.9381	0.938354	1.01942	0.768963	1.08605
8146468	RP1	NM_006269	1.67E-06	2.27918	0.0342796	1.6929	0.339517	1.24356	0.0725463	1.59101
8001423	RPGRIP1L	NM_015272	2.36E-11	2.08144	0.0001497	1.80854	0.0063032	1.47712	0.349961	1.16138
8001918	RRAD	NM_001128850	0.0001768	1.34511	1.42E-09	2.07121	0.259529	1.1271	0.493914	1.08539
8070603	RSPH1	NM_080860	5.21E-08	2.53699	0.0003823	2.41048	0.188488	1.34479	0.232237	1.35521
8131452	RSPH10B	NM_173565	8.60E-07	2.62018	0.0034107	2.29701	0.352549	1.2726	0.302077	1.35316
8138009	RSPH10B	NM_173565	9.71E-07	2.71934	0.0031308	2.4016	0.331021	1.30101	0.328564	1.34831
8121622	RSPH4A	NM_001010892	2.97E-07	2.7475	0.001082	2.54777	0.351912	1.27452	0.441102	1.2545
7933855	RTKN2	NM_145307	2.13E-05	-2.75037	0.34662	-1.38322	0.0197939	-2.10525	0.0514792	2.01694
8098060	RXFP1	NM_021634	4.55E-11	-2.27383	0.0012881	-1.7683	5.26E-06	-2.12324	0.242271	-1.23776
7920238	S100A12	NM_005621	6.16E-23	-3.79728	6.63E-10	-3.14356	3.05E-15	-4.01773	3.11E-12	-3.91553
7920244	S100A8	NM_002964	8.96E-18	-2.85138	1.24E-07	-2.46086	6.19E-10	-2.67406	0.0001476	-1.93848
7905571	S100A9	NM_002965	2.22E-18	-2.31026	2.35E-09	-2.23185	6.63E-12	-2.37069	1.33E-06	-1.94766
7903393	S1PR1	NM_001400	9.08E-18	-2.00475	9.21E-06	-1.64267	9.06E-12	-2.07023	0.0002842	-1.52137
7940654	SCGB1A1	NM_003357	4.29E-05	2.71679	0.0003744	3.59381	0.326823	1.37758	0.160183	1.68048
8116439	SCGB3A1	NM_052863	2.20E-07	2.50354	0.0001087	2.71643	0.0611131	1.55177	0.0956208	1.55492
8108995	SCGB3A2	NM_054023	4.37E-05	2.14585	0.330225	1.30205	0.0013379	2.24947	0.004752	2.23441
8056457	SCN1A	NM_001165963	7.10E-16	-2.27411	4.82E-06	-1.91814	0.000303	-1.59955	0.0415333	-1.3447
8056518	SCN7A	NM_002976	3.10E-13	-2.36321	3.18E-09	-2.74617	1.10E-12	-3.13066	0.0002328	-1.88991
8150889	SDR16C5	NM_138969	1.87E-16	-2.51855	1.22E-06	-2.14213	7.49E-08	-2.1893	7.39E-05	-1.89983
7922229	SELE	NM_000450	0.0143209	1.75602	0.0044325	2.63881	0.750388	-1.10407	0.0563708	-1.96192
7976496	SERPINA3	NM_001085	7.86E-11	-2.30894	1.59E-06	-2.4395	7.29E-13	-3.57287	3.02E-15	-4.97522
8023696	SERPINB3	NM_006919	1.10E-05	2.51578	0.0007561	2.81598	0.314826	1.32466	0.382259	1.31794

8021584	SERPINB5	NM_002639	4.80E-08	2.13518	0.0002787	2.07905	0.0278373	1.49794	0.795297	1.055
8071420	SERPIND1	NM_000185	5.48E-15	2.74097	0.000274	1.92307	1.39E-08	2.61777	0.0070176	1.65016
8135069	SERPINE1	NM_000602	0.0080394	-1.67772	0.636323	1.14503	0.0039355	-2.15705	0.006115	-2.28216
8091910	SERPINI2	NM_006217	8.99E-12	2.16633	0.0015509	1.66015	0.0005737	1.66448	0.0251394	1.44949
8103254	SFRP2	NM_003013	1.33E-31	6.86189	7.65E-15	5.54003	1.77E-16	5.4083	1.39E-06	2.87547
8139087	SFRP4	NM_003014	7.60E-13	2.6919	2.79E-09	3.28804	0.0003411	1.90192	0.0020314	1.86489
8070665	SIK1	NM_173354	9.28E-08	-1.84277	0.137277	-1.2758	6.55E-07	-2.16562	0.0047591	-1.62466
7979505	SIX1	NM_005982	6.22E-09	2.06405	0.0090286	1.5934	0.0404343	1.39924	0.556429	1.11452
7979510	SIX4	NM_017420	9.46E-12	2.03842	4.35E-05	1.83849	0.0042211	1.47504	0.301342	1.17032
7906613	SLAMF7	NM_021181	1.36E-10	2.4511	0.0003621	2.03587	6.28E-09	2.99106	0.0213722	1.60548
8059538	SLC19A3	NM_025243	4.34E-14	-2.24715	0.0027222	-1.56474	0.0002184	-1.6684	0.0297674	-1.39973
8154135	SLC1A1	NM_004170	4.39E-11	-2.13965	0.0262776	-1.43569	5.08E-06	-2.00928	0.251271	-1.2136
8104930	SLC1A3	NM_004172	0.0037099	-1.44878	0.0208476	-1.54508	0.0064145	-1.6068	2.15E-05	-2.33089
7983650	SLC27A2	NM_003645	9.03E-10	3.2027	3.71E-05	3.09618	0.0302803	1.71404	0.79997	1.07338
8101992	SLC39A8	NM_022154	3.58E-12	-2.4313	0.0026135	-1.7189	0.0015811	-1.68986	0.274762	-1.22474
8125149	SLC44A4	NM_025257	7.19E-08	2.00801	5.74E-05	2.1343	0.0871751	1.33946	0.289754	1.22623
8178653	SLC44A4	NM_025257	7.19E-08	2.00801	5.74E-05	2.1343	0.0871751	1.33946	0.289754	1.22623
8179861	SLC44A4	NM_025257	7.19E-08	2.00801	5.74E-05	2.1343	0.0871751	1.33946	0.289754	1.22623
8013989	SLC6A4	NM_001045	9.55E-08	-4.37844	0.225662	-1.6158	1.07E-05	-5.16848	0.577315	-1.25801
8090823	SLCO2A1	NM_005630	1.04E-11	-2.25228	0.0005642	-1.79214	1.32E-09	-2.65262	0.0002474	-1.90978
8063923	SLCO4A1	NM_016354	3.46E-25	-3.5452	1.50E-12	-3.2726	1.41E-16	-3.69381	2.53E-13	-3.60653
8113369	SLCO4C1	NM_180991	1.35E-10	-2.58511	0.0004944	-2.08573	0.0006127	-1.94637	0.0470958	-1.54024
8094301	SLIT2	NM_004787	4.99E-12	-2.02756	7.36E-05	-1.78015	1.50E-07	-2.04365	0.124493	-1.25726
7972239	SLITRK6	NM_032229	1.26E-12	2.54795	4.15E-05	2.1511	0.0028995	1.66192	0.0024327	1.79409
7951479	SLN	NM_003063	3.21E-09	2.25441	0.0144597	1.6147	2.67E-05	2.15843	0.0408503	1.51641
7984353	SMAD6	NR_027654	5.49E-12	-2.3879	0.0413398	-1.43488	5.47E-06	-2.13155	0.142554	-1.30946
7976812	SNORD113-4	NR_003232	2.90E-07	1.7766	7.20E-06	2.09191	0.0017124	1.59894	0.517587	1.11423
8080863	SNTN	NM_001080537	2.64E-08	4.04443	0.0001827	3.90141	0.147448	1.61395	0.136185	1.74452
8138363	SOSTDC1	NM_015464	0.0010349	-2.03279	0.521979	-1.22413	0.0118946	-2.09076	0.428826	1.29734
8084165	SOX2	NM_003106	2.03E-10	2.70114	0.0004117	2.20024	0.0154592	1.63912	0.216893	1.32742
7944869	SPA17	NM_017425	1.87E-08	2.06313	0.001184	1.82544	0.0202158	1.48401	0.0939503	1.37806
7918973	SPAG17	NM_206996	2.43E-08	2.8029	0.002124	2.26481	0.152994	1.41523	0.289135	1.33744
7926622	SPAG6	NM_012443	2.56E-09	3.90572	7.38E-05	3.69991	0.118204	1.59614	0.0955995	1.75738
7909768	SPATA17	NM_138796	2.41E-11	2.84177	6.54E-05	2.43793	0.0081273	1.71346	0.0843362	1.48419

8095021	SPATA18	NM_145263	3.33E-15	3.67997	6.74E-07	3.1885	0.0011596	1.98699	0.0414684	1.62108
8096301	SPP1	NM_001040058	3.95E-07	4.23686	0.0036154	3.31912	0.226239	1.57758	0.518481	-1.31589
8114797	SPRY4	NM_030964	7.24E-12	-2.04774	0.0128996	-1.44144	3.80E-06	-1.89323	0.001376	-1.63632
8168749	SRPX2	NM_014467	5.50E-07	-1.56247	0.0007587	-1.54717	1.54E-07	-1.89557	1.66E-10	-2.44693
8018774	ST6GALNAC1	NM_018414	1.57E-10	2.20288	6.69E-05	2.02518	0.0080047	1.53399	0.501421	1.12927
7902425	ST6GALNAC3	NM_152996	8.88E-24	-2.04681	1.11E-13	-2.09083	5.02E-15	-2.05831	6.96E-09	-1.79104
7946365	STK33	NM_030906	3.64E-08	2.13635	0.007151	1.70395	0.0685844	1.39234	0.320953	1.22537
7971126	STOML3	NM_145286	1.95E-07	2.88873	0.0001954	3.01904	0.242326	1.36996	0.187542	1.49347
7927915	STOX1	NM_152709	5.44E-07	2.04348	0.0079088	1.72997	0.251579	1.24178	0.65812	1.0989
7978376	STXBP6	NM_014178	1.83E-10	-2.04283	0.0381739	-1.38883	2.85E-07	-2.1606	0.422535	-1.14083
8146863	SULF1	NM_001128205	6.91E-14	2.40361	1.25E-08	2.60402	1.91E-06	2.07303	0.0273122	1.45269
8100798	SULT1B1	NM_014465	2.78E-17	-2.42921	0.0007849	-1.62138	7.55E-10	-2.32524	2.66E-10	-2.66787
8163202	SVEP1	NM_153366	6.26E-11	-2.15468	0.0015088	-1.69752	3.10E-05	-1.90817	0.0012083	-1.75502
8097080	SYNPO2	NM_133477	3.99E-11	2.42316	0.0018436	1.80733	0.0030774	1.6784	0.308751	1.22057
8097991	TDO2	NM_005651	3.25E-16	3.72511	4.74E-06	2.79894	1.89E-07	2.96947	0.253065	1.29864
8154692	TEK	NM_000459	3.81E-10	-2.03252	0.0024759	-1.62989	1.85E-05	-1.90475	0.036424	-1.41858
8011990	TEKT1	NM_053285	2.61E-07	2.59854	0.0005	2.55468	0.313589	1.28003	0.212719	1.41225
7983828	TEX9	NM_198524	3.28E-13	2.83721	9.94E-05	2.19505	0.0024152	1.75189	0.168425	1.32983
7980316	TGFB3	NM_003239	4.81E-16	2.05821	1.14E-08	2.05456	1.30E-09	2.032	0.171415	1.18879
7917649	TGFBR3	NM_003243	6.04E-16	-1.84787	2.21E-07	-1.74039	1.29E-12	-2.05537	3.58E-06	-1.66979
7982597	THBS1	NM_003246	7.68E-06	-1.83231	0.10298	-1.37658	4.72E-05	-2.1122	2.54E-06	-2.67442
8130867	THBS2	NM_003247	2.67E-09	1.90903	2.75E-06	2.09825	0.0006524	1.6328	0.291849	-1.18425
7952268	THY1	NM_006288	1.87E-24	3.44363	2.56E-14	3.61825	1.62E-15	3.48377	0.0021557	1.66088
8075635	TIMP3	NM_000362	4.71E-09	-1.92715	0.0001653	-1.8381	1.14E-07	-2.23339	0.203026	-1.23485
8085360	TIMP4	NM_003256	7.00E-14	-1.82702	1.70E-08	-1.918	3.31E-13	-2.21656	7.80E-13	-2.4184
8016841	TMEM100	NM_001099640	1.29E-12	-3.68558	0.0015295	-2.26917	8.93E-07	-3.29264	0.464013	-1.21518
8083897	TMEM212	NM_001164436	8.83E-07	2.94166	0.0015208	2.75011	0.217979	1.43117	0.25125	1.45814
8113483	TMEM232	NM_001039763	3.15E-09	3.34723	0.0001972	2.98598	0.0848336	1.58492	0.208854	1.46059
8081288	TMEM45A	NM_018004	2.34E-11	2.5648	0.0012169	1.90845	0.0178342	1.54285	0.851757	1.03913
8147313	TMEM67	NM_153704	1.70E-08	2.04201	0.0007713	1.84765	0.090427	1.3257	0.478436	1.14223
7944164	TMPRSS4	NM_019894	2.45E-11	2.79158	5.70E-05	2.41979	0.0085791	1.6918	0.554082	1.14187
7962058	TMTC1	NM_175861	2.82E-11	-1.79514	8.33E-08	-1.98085	1.72E-06	-1.74764	5.31E-08	-2.06139
8163637	TNC	NM_002160	5.41E-15	3.11943	0.000217	2.11727	7.52E-06	2.32524	0.646808	1.09987
8014974	TOP2A	NM_001067	1.02E-12	1.92415	0.0007706	1.54459	9.54E-09	2.01856	0.272209	1.15719

8084766	TP63	NM_003722	1.73E-14	2.68431	1.84E-05	2.17115	0.0027484	1.63856	0.202964	1.26503
8002020	TPPP3	NM_016140	3.27E-21	2.58254	8.19E-11	2.47891	5.04E-08	1.99546	2.47E-08	2.22568
7952290	TRIM29	NM_012101	2.20E-10	2.30986	0.0002539	1.99442	0.0242278	1.47396	0.127688	1.34355
8054166	TSGA10	NM_025244	5.70E-08	2.05825	0.0052682	1.70637	0.104444	1.3295	0.444377	1.16324
7901175	TSPAN1	NM_005727	6.47E-10	3.45457	5.09E-06	3.76105	0.0406495	1.71065	0.0762359	1.68999
7954653	TSPAN11	NM_001080509	4.44E-17	2.09214	2.75E-08	1.98728	0.0001524	1.52595	0.0047001	1.42429
7964927	TSPAN8	NM_004616	0.0495231	1.32667	0.0004974	2.11486	0.253877	-1.25008	0.708074	-1.08616
7934334	TTC18	NM_145170	1.25E-07	2.14925	0.007337	1.74987	0.164992	1.30389	0.179942	1.33579
8007176	TTC25	NM_031421	1.01E-07	2.39665	0.0017987	2.09743	0.073911	1.47362	0.166453	1.40307
8057056	TTN	NM_133378	1.18E-07	-2.16188	1.53E-05	-2.50994	0.0275734	-1.52939	0.55991	-1.13465
8019842	TYMS	NM_001071	2.15E-15	2.0734	4.19E-05	1.69157	7.64E-09	2.00838	0.0162468	1.37273
7898623	UBXN10	NM_152376	1.51E-12	2.25706	4.21E-06	2.12846	0.0187624	1.41745	0.0041074	1.62083
8049349	UGT1A1	NM_000463	3.96E-12	2.28548	0.0014105	1.71008	0.0292063	1.39869	0.760799	1.05404
7911343	UIMC1	AF284753	0.0004821	-1.50735	4.63E-05	-2.03884	0.007624	-1.52969	0.457452	1.14188
8165703	UIMC1	AF284753	0.0004821	-1.50735	4.63E-05	-2.03884	0.007624	-1.52969	0.457452	1.14188
8144397	USP17L2	NM_201402	0.0002522	-1.81164	0.0011343	-2.17953	0.0012816	-2.03439	0.410659	-1.2246
8149241	USP17L2	NM_201402	0.0002522	-1.81164	0.0011343	-2.17953	0.0012816	-2.03439	0.410659	-1.2246
8094134	USP17L6P	NR_027279	0.0003724	-2.15787	0.0016532	-2.72509	0.0006613	-2.7239	0.260728	-1.44666
8097098	USP53	NM_019050	1.16E-16	-1.96487	3.89E-09	-1.97384	5.87E-12	-2.10504	9.40E-08	-1.89072
7903358	VCAM1	NM_001078	8.84E-10	2.61305	3.14E-06	2.885	2.97E-05	2.38727	0.490726	1.17223
8088979	VGLL3	NM_016206	3.19E-20	-2.02912	1.15E-08	-1.83412	4.68E-13	-2.06785	1.87E-05	-1.59245
8079060	VIPR1	NM_004624	1.66E-13	-3.42265	0.0020857	-2.05079	8.53E-09	-3.58004	0.0791823	-1.52698
8129618	VNN1	NM_004666	1.25E-07	-1.8307	0.0016793	-1.68199	8.06E-06	-1.99254	3.37E-06	-2.25378
8129637	VNN2	NM_004665	2.10E-10	-2.21097	0.0017204	-1.74624	2.82E-07	-2.3611	1.56E-05	-2.24569
8169263	VSIG1	NM_001170553	4.99E-11	3.91914	0.0003357	2.89138	1.28E-07	4.34209	0.0507915	1.81411
7981732	VSIG6	ENST0000033856	5.16E-10	3.23899	0.0015639	2.3487	6.00E-07	3.54363	0.0260005	1.86281
7986639	VSIG6	ENST0000033856	8.19E-09	2.81425	0.000811	2.37567	7.08E-06	2.94889	0.171454	1.43874
7993898	VWA3A	NM_173615	1.28E-07	2.00182	0.0039155	1.7288	0.137607	1.29399	0.0915599	1.39263
8043747	VWA3B	NM_144992	9.59E-08	2.46138	0.0010861	2.22188	0.356522	1.22772	0.105953	1.50282
8004889	WDR16	NM_145054	3.90E-08	2.47492	0.0005585	2.27733	0.188983	1.32995	0.163304	1.40778
8091922	WDR49	NM_178824	1.53E-13	3.80933	3.17E-05	2.89772	0.0004202	2.28461	0.0052406	2.08493
7902660	WDR63	NM_145172	1.95E-09	2.48415	0.0003679	2.17275	0.227994	1.26964	0.338218	1.23881
7900639	WDR65	NR_030778	3.21E-07	2.24354	0.0023481	2.00758	0.248196	1.2731	0.313371	1.26884
7959330	WDR66	NM_144668	6.48E-07	2.07224	0.0010179	2.01268	0.362264	1.19314	0.45101	1.17943

8048870	WDR69	NM_178821	5.27E-06	2.18114	0.006555	1.96901	0.576948	1.13545	0.501526	1.18882
7916789	WDR78	NM_024763	6.68E-09	2.7477	0.0001977	2.55077	0.198196	1.34168	0.0999243	1.5299
8055361	YSK4	NM_025052	2.50E-07	2.1069	0.0055921	1.78335	0.197293	1.27925	0.288649	1.25718
8091887	ZBBX	NM_024687	1.22E-08	4.14006	0.0004894	3.505	0.149971	1.60229	0.169965	1.6614
7943984	ZBTB16	NM_006006	4.30E-10	-2.54018	9.81E-06	-2.59429	2.83E-07	-2.79837	0.003905	-1.89469
8057418	ZNF385B	NM_152520	1.84E-14	-2.28646	7.48E-06	-1.97653	0.0044418	-1.48137	0.379986	-1.14545
8085774	ZNF385D	NM_024697	5.77E-11	2.31652	0.0050287	1.66453	0.0002027	1.87127	0.874397	-1.03006
7930915		---	1.50E-19	-2.04013	6.78E-10	-1.9748	2.07E-10	-1.91148	2.40E-06	-1.70068
8047763		---	2.35E-15	2.03915	0.0003051	1.57042	2.56E-07	1.82885	0.0329283	1.31622
8173924		---	5.21E-08	-2.14853	0.0009592	-1.95529	0.0169535	-1.55895	0.414255	-1.18589
7911335		---	0.0040902	-1.59167	0.0047288	-1.96663	0.0004527	-2.17719	0.636254	-1.1239
8043456		---	5.98E-09	1.98834	0.0089305	1.55574	1.74E-06	2.13787	0.0735831	1.36827
8043480		---	9.24E-08	1.89129	0.0082012	1.57513	1.02E-05	2.03392	0.0381426	1.44752
8053715		---	1.10E-08	1.94236	0.0144103	1.50202	1.16E-06	2.1417	0.0684434	1.36952
8053718		---	0.0001817	1.86556	0.187969	1.37659	0.0003454	2.25162	0.243342	1.34287
8102592		---	1.52E-06	1.69842	0.501297	1.11245	1.69E-07	2.20309	0.0216223	1.46422
8151629		---	9.00E-06	-1.93034	0.0024177	-1.92742	1.99E-05	-2.36336	0.0089068	-1.79954
8165694		---	0.0040902	-1.59167	0.0047288	-1.96663	0.0004527	-2.17719	0.636254	-1.1239
7981720		---	1.32E-09	2.10062	0.0090568	1.57654	8.31E-07	2.24689	0.0953852	1.3518
8043441		---	4.15E-08	3.51787	0.0146637	2.2338	2.28E-05	3.68699	0.0802484	1.81808
8043443		---	1.16E-06	2.37746	0.0216915	1.8065	5.78E-05	2.63205	0.61071	1.14538
8043446		---	2.42E-07	2.72126	0.0275232	1.85022	3.55E-05	2.94515	0.209083	1.43869
8053713		---	1.11E-07	2.54664	0.0107583	1.90975	3.00E-05	2.68837	0.190062	1.4108
7965587		---	0.0244825	1.52564	0.812492	1.06769	6.27E-05	2.82554	7.41E-06	3.74668
8162531		---	8.80E-06	-1.79483	0.0088113	-1.65179	5.32E-05	-2.0585	4.50E-05	-2.27973
7896752		---	1.57E-06	-1.66941	4.34E-08	-2.39204	0.0007025	-1.62588	0.290572	-1.18427
8043375		---	2.84E-06	-1.61544	5.74E-08	-2.29373	0.0007738	-1.58957	0.325317	-1.16332
8045533		---	0.0083598	-1.60399	0.0008585	-2.43033	0.0090845	-1.89121	0.673978	1.12206
8045804		---	0.0068299	-1.56438	0.0025128	-2.09844	0.0084662	-1.81051	0.755935	-1.08169
8098193		---	7.09E-05	-1.53853	6.20E-06	-2.07337	0.0001287	-1.75998	0.0009632	-1.73006
8100308		---	4.81E-05	-1.85113	0.0017742	-2.00313	0.0009422	-1.97087	0.135343	-1.40844
8145083		---	4.45E-07	-1.88976	9.25E-05	-2.05438	0.0851086	-1.33355	0.338313	-1.19772
8156358		---	0.0063667	-1.56447	0.0020822	-2.11469	0.0103811	-1.77292	0.900961	1.03166
8165667		---	1.57E-06	-1.66941	4.34E-08	-2.39204	0.0007025	-1.62588	0.290572	-1.18427

8017619	---	2.01E-11	-2.24621	7.23E-07	-2.36859	0.0003004	-1.77109	0.0005911	-1.84573
8165709	---	0.0002786	-1.733	9.70E-07	-3.03709	2.76E-05	-2.3858	0.716531	-1.08685
7896748	---	1.48E-08	-2.69461	2.60E-07	-3.75984	3.71E-07	-3.33227	0.0492117	-1.66884
7970392	---	0.0013309	-2.17709	0.002707	-2.92676	0.0001986	-3.43931	0.408227	-1.35698
7986637	---	1.91E-06	2.61584	0.0099887	2.12629	0.0012811	2.39428	0.0998843	1.64767
8100310	---	2.01E-05	-2.51517	0.000327	-3.13454	0.000174	-3.00966	0.211088	-1.5044
8165684	---	2.53E-05	-2.0327	3.37E-06	-3.2006	9.30E-05	-2.4466	0.385831	-1.24652

Supplemental Table S2. Differentially expressed genes at 5%FDR between severe and mild IIP, as defined by percent predicted DLCO.

Transcript ID	gene_assignment	Gene Symbol	RefSeq	p-value(Severe vs. Mild; DLCO)	Fold-Change(Severe vs. Mild; DLCO)
7922229	NM_000450 // SELE // selectin E // 1q22-q25 // 6401 /// ENST00000333360 // SELE // sele	SELE	NM_000450	9.39E-05	2.87225
7921821	NM_005099 // ADAMTS4 // ADAM metallopeptidase with thrombospondin type 1 motif, 4 // 1q	ADAMTS4	NM_005099	5.82E-05	2.22395
8146957	NM_015886 // PI15 // peptidase inhibitor 15 // 8q21.11 // 51050 /// ENST00000260113 //	PI15	NM_015886	1.93E-05	2.03628
8043981	NM_004633 // IL1R2 // interleukin 1 receptor, type II // 2q12 // 7850 /// NM_173343 //	IL1R2	NM_004633	0.000352	1.86409
8088560	NM_182920 // ADAMTS9 // ADAM metallopeptidase with thrombospondin type 1 motif, 9 // 3p	ADAMTS9	NM_182920	1.37E-05	1.83912
7976496	NM_001085 // SERPINA3 // serpin peptidase inhibitor, clade A (alpha-1 antiproteinase, a	SERPINA3	NM_001085	9.45E-06	1.81059
8162276	NM_005384 // NFIL3 // nuclear factor, interleukin 3 regulated // 9q22 // 4783 /// ENST0	NFIL3	NM_005384	0.000105	1.58707
8020762	NM_001944 // DSG3 // desmoglein 3 // 18q12.1 // 1830 /// ENST00000257189 // DSG3 // des	DSG3	NM_001944	1.46E-04	1.58328
7982377	NM_013372 // GREM1 // gremlin 1 // 15q13.3 // 26585 /// NM_001191323 // GREM1 // gremli	GREM1	NM_013372	5.50E-05	1.57037

8168749	NM_014467 // SRPX2 // sushi-repeat-containing protein, X-linked 2 // Xq21.33-q23 // 272	SRPX2	NM_014467	1.89E-06	1.56646
8106743	NM_004385 // VCAN // versican // 5q14.3 // 1462 /// NM_001164097 // VCAN // versican //	VCAN	NM_004385	8.28E-05	1.56367
8043909	NM_002518 // NPAS2 // neuronal PAS domain protein 2 // 2q11.2 // 4862 /// ENST000003356	NPAS2	NM_002518	5.21E-05	1.56153
8145122	NM_001128431 // SLC39A14 // solute carrier family 39 (zinc transporter), member 14 // 8	SLC39A14	NM_001128431	1.16E-04	1.52486
7974851	NM_001530 // HIF1A // hypoxia inducible factor 1, alpha subunit (basic helix-loop-helix	HIF1A	NM_001530	6.77E-07	1.52214
8113220	NM_012081 // ELL2 // elongation factor, RNA polymerase II, 2 // 5q15 // 22936 /// ENST0	ELL2	NM_012081	8.18E-06	1.50817
7986446	NM_000693 // ALDH1A3 // aldehyde dehydrogenase 1 family, member A3 // 15q26.3 // 220 //	ALDH1A3	NM_000693	3.10E-05	1.48845
8115814	NM_001017995 // SH3PXD2B // SH3 and PX domains 2B // 5q35.1 // 285590 /// ENST000003116	SH3PXD2B	NM_001017995	2.99E-05	1.48396
7921344	NM_012081 // ELL2 // elongation factor, RNA polymerase II, 2 // 5q15 // 22936 /// ENST0	ELL2	NM_012081	1.58E-05	1.47836
8106098	NM_005909 // MAP1B // microtubule-associated protein 1B // 5q13 // 4131 /// ENST0000029	MAP1B	NM_005909	3.41E-05	1.4715
7971104	NM_016179 // TRPC4 // transient receptor potential cation channel, subfamily C, member	TRPC4	NM_016179	2.27E-04	1.46009

8156043	NM_058179 // PSAT1 // phosphoserine aminotransferase 1 // 9q21.2 // 29968 /// NM_021154	PSAT1	NM_058179	1.62E-04	1.45883
8044391	NM_006343 // MERTK // c-mer proto-oncogene tyrosine kinase // 2q14.1 // 10461 /// ENST0	MERTK	NM_006343	0.00015	1.39906
8157216	NM_003358 // UGCG // UDP-glucose ceramide glucosyltransferase // 9q31 // 7357 /// ENST0	UGCG	NM_003358	3.79E-05	1.39282
7915787	NM_003629 // PIK3R3 // phosphoinositide-3-kinase, regulatory subunit 3 (gamma) // 1p34.	PIK3R3	NM_003629	2.89E-05	1.39272
8007931	NM_000212 // ITGB3 // integrin, beta 3 (platelet glycoprotein IIIa, antigen CD61) // 17	ITGB3	NM_000212	4.42E-05	1.36675
7897449	NM_025106 // SPSB1 // splA/ryanodine receptor domain and SOCS box containing 1 // 1p36.	SPSB1	NM_025106	1.08E-04	1.35934
8105040	NM_003999 // OSMR // oncostatin M receptor // 5p13.1 // 9180 /// NM_001168355 // OSMR /	OSMR	NM_003999	0.000122	1.3379
7902227	NM_001924 // GADD45A // growth arrest and DNA-damage-inducible, alpha // 1p31.2 // 1647	GADD45A	NM_001924	2.57E-05	1.33243
8085914	NM_003615 // SLC4A7 // solute carrier family 4, sodium bicarbonate cotransporter, membe	SLC4A7	NM_003615	3.32E-05	1.33186
8043995	NM_000877 // IL1R1 // interleukin 1 receptor, type I // 2q12 // 3554 /// ENST0000023394	IL1R1	NM_000877	3.37E-05	1.32769
8060963	NM_003081 // SNAP25 // synaptosomal-associated protein, 25kDa // 20p12-p11.2 // 6616 //	SNAP25	NM_003081	5.83E-05	1.32478

8041168	NR_002741 // SNORD53 // small nucleolar RNA, C/D box 53 // 2p23.2 // 26796	SNORD53	NR_002741	1.86E-04	1.32352
8041149	NM_015131 // WDR43 // WD repeat domain 43 // 2p23.2 // 23160 /// ENST00000407426 // WDR	WDR43	NM_015131	1.01E-04	1.31493
8113130	NM_024717 // MCTP1 // multiple C2 domains, transmembrane 1 // 5q15 // 79772 /// NM_0010	MCTP1	NM_024717	5.45E-05	1.3083
8106986	NM_014899 // RHOBTB3 // Rho-related BTB domain containing 3 // 5q15 // 22836 /// ENST00	RHOBTB3	NM_014899	8.06E-05	1.29922
8133155	NM_003596 // TPST1 // tyrosylprotein sulfotransferase 1 // 7q11.21 // 8460 /// ENST0000	TPST1	NM_003596	0.000213	1.29666
8102482	NM_014822 // SEC24D // SEC24 family, member D (S. cerevisiae) // 4q26 // 9871 /// ENST0	SEC24D	NM_014822	1.04E-05	1.28533
8117128	NM_001949 // E2F3 // E2F transcription factor 3 // 6p22 // 1871 /// ENST00000346618 //	E2F3	NM_001949	3.51E-06	1.27104
7922610	NM_007314 // ABL2 // v-abl Abelson murine leukemia viral oncogene homolog 2 // 1q25.2 /	ABL2	NM_007314	3.36E-04	1.25995
8076515	NM_014570 // ARFGAP3 // ADP-ribosylation factor GTPase activating protein 3 // 22q13.2	ARFGAP3	NM_014570	0.000135	1.25794
8006123	NM_001304 // CPD // carboxypeptidase D // 17q11.2 // 1362 /// ENST00000225719 // CPD //	CPD	NM_001304	1.67E-04	1.24884
7966462	NM_024953 // NAA25 // N(alpha)-acetyltransferase 25, NatB auxiliary subunit // 12q24.13	NAA25	NM_024953	2.65E-05	1.24057

8066939	NM_004776 // B4GALT5 // UDP-Gal:betaGlcNAc beta 1,4- galactosyltransferase, polypeptide	B4GALT5	NM_004776	2.58E-04	1.23765
7939376	NM_174902 // LDLRAD3 // low density lipoprotein receptor class A domain containing 3 //	LDLRAD3	NM_174902	0.000129	1.21772
7985934	NM_020210 // SEMA4B // sema domain, immunoglobulin domain (Ig), transmembrane domain (T	SEMA4B	NM_020210	1.21E-04	1.20655
8123562	NM_001500 // GMDS // GDP-mannose 4,6-dehydratase // 6p25 // 2762 /// ENST00000380815 //	GMDS	NM_001500	0.000101	1.19484
7966839	NM_019086 // VSIG10 // V-set and immunoglobulin domain containing 10 // 12q24.23 // 546	VSIG10	NM_019086	2.09E-04	-1.17326
8070912	NM_194255 // SLC19A1 // solute carrier family 19 (folate transporter), member 1 // 21q2	SLC19A1	NM_194255	2.93E-05	-1.21638
8168408	---		---	1.10E-04	-1.23095
8149885	NM_000680 // ADRA1A // adrenergic, alpha-1A-, receptor // 8p21.2 // 148 /// NM_033303 /	ADRA1A	NM_000680	1.67E-05	-1.23507
8109086	NM_000024 // ADRB2 // adrenergic, beta-2-, receptor, surface // 5q31-q32 // 154 /// ENS	ADRB2	NM_000024	0.000145	-1.24028
7950473	NM_004041 // ARRB1 // arrestin, beta 1 // 11q13 // 408 /// NM_020251 // ARRB1 // arrest	ARRB1	NM_004041	0.000236	-1.25118
8074780	NM_013313 // YPEL1 // yippee-like 1 (Drosophila) // 22q11.2 // 29799 /// NM_148175 // P	YPEL1	NM_013313	3.40E-05	-1.25594
8082465	NM_024768 // CCDC48 // coiled-coil domain containing 48 // 3q21.3 // 79825 /// AK022119	CCDC48	NM_024768	9.77E-05	-1.27811

8035201	NM_015692 // CPAMD8 // C3 and PZP-like, alpha-2-macroglobulin domain containing 8 // 19	CPAMD8	NM_015692	5.51E-05	-1.30016
7920082	NM_005060 // RORC // RAR-related orphan receptor C // 1q21 // 6097 /// NM_001001523 //	RORC	NM_005060	1.31E-05	-1.30477
8041206	NM_030915 // LBH // limb bud and heart development homolog (mouse) // 2p23.1 // 81606 /	LBH	NM_030915	3.60E-04	-1.32539
8009096	NM_000789 // ACE // angiotensin I converting enzyme (peptidyl-dipeptidase A) 1 // 17q23	ACE	NM_000789	1.29E-05	-1.32854
7940530	NM_001127392 // C11orf9 // chromosome 11 open reading frame 9 // 11q12-q13.1 // 745 ///	C11orf9	NM_001127392	3.24E-05	-1.3326
7929065	NM_001548 // IFIT1 // interferon-induced protein with tetratricopeptide repeats 1 // 10	IFIT1	NM_001548	6.95E-05	-1.35309
7968650	NM_203451 // C13orf36 // chromosome 13 open reading frame 36 // 13q13.3 // 400120 /// E	C13orf36	NM_203451	7.07E-06	-1.37536
8125341	NM_001136 // AGER // advanced glycosylation end product-specific receptor // 6p21.3 //	AGER	NM_001136	7.53E-05	-1.42019
8089467	NM_024508 // ZBED2 // zinc finger, BED-type containing 2 // 3q13.2 // 79413 /// ENST000	ZBED2	NM_024508	3.47E-05	-1.44758
7921702	NM_080878 // ITLN2 // intelectin 2 // 1q22-q23 // 142683 /// ENST00000368029 // ITLN2 /	ITLN2	NM_080878	1.57E-05	-1.47918
8155734	NM_004816 // FAM189A2 // family with sequence similarity 189, member A2 // 9q21.11 // 9	FAM189A2	NM_004816	1.98E-05	-1.56097

8171248	NM_000216 // KAL1 // Kallmann syndrome 1 sequence // Xp22.32 // 3730 /// ENST0000026264	KAL1	NM_000216	5.27E-05	-1.67256
8156569	NR_029664 // MIR23B // microRNA 23b // 9q22.32 // 407011 /// AF043897 // C9orf3 // chro	MIR23B	NR_029664	0.000239	-1.71053
8109383	NM_000827 // GRIA1 // glutamate receptor, ionotropic, AMPA 1 // 5q33|5q31.1 // 2890 ///	GRIA1	NM_000827	4.94E-06	-1.86131
8171427	NM_004469 // FIGF // c-fos induced growth factor (vascular endothelial growth factor D)	FIGF	NM_004469	0.000279	-2.06932
7933855	NM_145307 // RTKN2 // rhotekin 2 // 10q21.2 // 219790 /// ENST00000373789 // RTKN2 // r	RTKN2	NM_145307	0.000203	-2.57427
7897378	NM_016831 // PER3 // period homolog 3 (Drosophila) // 1p36.23 // 8863 /// ENST000003619	PER3	NM_016831	3.72E-04	
7905553	NM_003125 // SPRR1B // small proline-rich protein 1B // 1q21-q22 // 6699 /// ENST000003	SPRR1B	NM_003125	5.35E-05	
7944082	NM_001001522 // TAGLN // transgelin // 11q23.2 // 6876 /// NM_003186 // TAGLN // transg	TAGLN	NM_001001522	8.96E-05	
7963880	NM_001144996 // ITGA7 // integrin, alpha 7 // 12q13 // 3679 /// NM_002206 // ITGA7 // i	ITGA7	NM_001144996	3.04E-04	
7970441	NM_004004 // GJB2 // gap junction protein, beta 2, 26kDa // 13q11-q12 // 2706 /// ENST0	GJB2	NM_004004	0.000364	
7970569	NM_014363 // SACS // spastic ataxia of Charlevoix-Saguenay (sacsin) // 13q12 // 26278 /	SACS	NM_014363	1.47E-04	

7994603	NM_001030288 // SPN // sialophorin // 16p11.2 // 6693 /// NM_003123 // SPN // sialophor	SPN	NM_001030288	9.01E-05
8016980	NR_029683 // MIR142 // microRNA 142 // 17q22 // 406934	MIR142	NR_029683	0.00029
8038117	NM_001352 // DBP // D site of albumin promoter (albumin D-box) binding protein // 19q13	DBP	NM_001352	0.000189
8040080	NM_080657 // RSAD2 // radical S-adenosyl methionine domain containing 2 // 2p25.2 // 91	RSAD2	NM_080657	0.000329
8040587	NM_014971 // EFR3B // EFR3 homolog B (S. cerevisiae) // 2p23.3 // 22979 /// ENST0000040	EFR3B	NM_014971	0.000128
8050102	NM_207315 // CMPK2 // cytidine monophosphate (UMP-CMP) kinase 2, mitochondrial // 2p25.	CMPK2	NM_207315	1.56E-04
8057677	NM_014585 // SLC40A1 // solute carrier family 40 (iron-regulated transporter), member 1	SLC40A1	NM_014585	3.75E-04
8059376	NM_006216 // SERPINE2 // serpin peptidase inhibitor, clade E (nexin, plasminogen activa	SERPINE2	NM_006216	0.000309
8082585	NR_022012 // COL6A5 // collagen, type VI, alpha 5 // 3q22.1 // 256076 /// NM_153264 //	COL6A5	NR_022012	0.000222
8092578	NM_004454 // ETV5 // ets variant 5 // 3q28 // 2119 /// ENST00000306376 // ETV5 // ets v	ETV5	NM_004454	3.79E-04
8099685	NM_018176 // LGI2 // leucine-rich repeat LGI family, member 2 // 4p15.2 // 55203 /// EN	LGI2	NM_018176	3.24E-04

8126729	NM_001114086 // CLIC5 // chloride intracellular channel 5 // 6p12.3 // 53405 /// NM_016	CLIC5	NM_001114086	5.70E-05
8133741	NM_030570 // UPK3B // uroplakin 3B // 7q11.2 // 80761 /// NM_182684 // UPK3B // uroplak	UPK3B	NM_030570	1.26E-05
8178771	NM_001136 // AGER // advanced glycosylation end product-specific receptor // 6p21.3 //	AGER	NM_001136	7.08E-05
8179967	NM_001136 // AGER // advanced glycosylation end product-specific receptor // 6p21.3 //	AGER	NM_001136	6.22E-05

Supplemental Table S3. Genes whose expression correlates with percent predicted DLCO in all IIPs at 5%FDR.

Transcript ID	RefSeq	Gene Symbol	gene_assignment	p-value(DLCO)	PartialCorr(DLCO)
8045776	NM_052917	GALNT13	NM_052917 // GALNT13 // UDP-N-acetyl-alpha-D-galactosamine:polypeptide N-acetylgalactos	4.45E-05	0.261029
7966839	NM_019086	VSIG10	NM_019086 // VSIG10 // V-set and immunoglobulin domain containing 10 // 12q24.23 // 546	1.66E-05	0.292799
7940530	NM_001127392	C11orf9	NM_001127392 // C11orf9 // chromosome 11 open reading frame 9 // 11q12-q13.1 // 745 ///	6.06E-05	0.286701
7933298	NM_031912	SYT15	NM_031912 // SYT15 // synaptotagmin XV // 10q11.1 // 83849 /// NM_181519 // SYT15 // sy	0.000298475	0.262407
8008900	NM_000717	CA4	NM_000717 // CA4 // carbonic anhydrase IV // 17q23 // 762 /// ENST00000300900 // CA4 //	2.98E-06	0.314848
7971077	NM_006475	POSTN	NM_006475 // POSTN // periostin, osteoblast specific factor // 13q13.3 // 10631 /// NM_	0.000552048	-0.243928
7926677	NM_001145373	OTUD1	NM_001145373 // OTUD1 // OTU domain containing 1 // 10p12.2 // 220213 /// ENST000003764	0.00082402	0.235498
7968650	NM_203451	C13orf36	NM_203451 // C13orf36 // chromosome 13 open reading frame 36 // 13q13.3 // 400120 /// E	2.00E-05	0.295458
8147244	NM_022351	NECAB1	NM_022351 // NECAB1 // N-terminal EF-hand calcium binding protein 1 // 8q21.3 // 64168	0.00066345	0.248169
7921702	NM_080878	ITLN2	NM_080878 // ITLN2 // intelectin 2 // 1q22-q23 // 142683 /// ENST00000368029 // ITLN2 /	1.52E-07	0.360355

8062571	NM_030919	FAM83D	NM_030919 // FAM83D // family with sequence similarity 83, member D // 20q11.22-q12 //	3.10E-05	-0.30395
7951363	NM_001191016	CASP12	NM_001191016 // CASP12 // caspase 12 (gene/pseudogene) // 11q22.3 // 120329 /// NR_0340	0.000122099	0.275097
7996819	NM_001793	CDH3	NM_001793 // CDH3 // cadherin 3, type 1, P-cadherin (placental) // 16q22.1 // 1001 ///	0.000389168	-0.258007
7950473	NM_004041	ARRB1	NM_004041 // ARRB1 // arrestin, beta 1 // 11q13 // 408 /// NM_020251 // ARRB1 // arrest	2.19E-05	0.299536
8104079	NM_005245	FAT1	NM_005245 // FAT1 // FAT tumor suppressor homolog 1 (Drosophila) // 4q35 // 2195 /// EN	0.000877193	-0.242269
8097335	NM_014278	HSPA4L	NM_014278 // HSPA4L // heat shock 70kDa protein 4-like // 4q28 // 22824 /// ENST0000029	0.00100447	-0.244037
8022674	NM_001792	CDH2	NM_001792 // CDH2 // cadherin 2, type 1, N-cadherin (neuronal) // 18q11.2 // 1000 /// E	0.000325148	-0.262728
7924996	NM_032800	C1orf198	NM_032800 // C1orf198 // chromosome 1 open reading frame 198 // 1q42.2 // 84886 /// NM_	0.000992121	0.246873
8111892	NM_000436	OXCT1	NM_000436 // OXCT1 // 3-oxoacid CoA transferase 1 // 5p13.1 // 5019 /// ENST00000196371	0.00210193	-0.234006
7976443	NM_001130080	IFI27	NM_001130080 // IFI27 // interferon, alpha-inducible protein 27 // 14q32 // 3429 /// NM	0.00110118	0.249108
8126839	NM_014452	TNFRSF21	NM_014452 // TNFRSF21 // tumor necrosis factor receptor superfamily, member 21 // 6p21.	0.000687146	-0.252525
8042788	NM_001615	ACTG2	NM_001615 // ACTG2 // actin, gamma 2, smooth muscle, enteric // 2p13.1 // 72 /// ENST00	8.55E-05	-0.294057

8054377	NM_201555	FHL2	NM_201555 // FHL2 // four and a half LIM domains 2 // 2q12.2 // 2274 /// NM_001450 // F	0.000437891	-0.254778
8112841	NM_004272	HOMER1	NM_004272 // HOMER1 // homer homolog 1 (Drosophila) // 5q14.2 // 9456 /// ENST000003340	0.00293604	-0.232576
7950810	NM_206927	SYTL2	NM_206927 // SYTL2 // synaptotagmin-like 2 // 11q14 // 54843 /// NM_206928 // SYTL2 //	0.00217167	-0.230883
7904293	NM_020440	PTGFRN	NM_020440 // PTGFRN // prostaglandin F2 receptor negative regulator // 1p13.1 // 5738 /	7.84E-05	-0.289563
8087925	NM_003280	TNNC1	NM_003280 // TNNC1 // troponin C type 1 (slow) // 3p21.1 // 7134 /// ENST00000232975 //	0.000999954	0.24508
8133741	NM_030570	UPK3B	NM_030570 // UPK3B // uroplakin 3B // 7q11.2 // 80761 /// NM_182684 // UPK3B // uroplak	5.89E-05	0.293385
8048541	NM_001927	DES	NM_001927 // DES // desmin // 2q35 // 1674 /// ENST00000373960 // DES // desmin // 2q35	0.000210589	-0.279664
8161884	NM_015225	PRUNE2	NM_015225 // PRUNE2 // prune homolog 2 (Drosophila) // 9q21.2 // 158471 /// AB535152 //	0.00115703	-0.249017
8144228	AK094159	FLJ36840	AK094159 // FLJ36840 // hypothetical LOC645524 // --- // 645524	0.000425807	0.28404
7927277	NM_014696	GPRIN2	NM_014696 // GPRIN2 // G protein regulated inducer of neurite outgrowth 2 // 10q11.22 /	0.00254565	0.228663
8161865	NM_015225	PRUNE2	NM_015225 // PRUNE2 // prune homolog 2 (Drosophila) // 9q21.2 // 158471 /// AB050197 //	0.00153566	-0.248816

8040587	NM_014971	EFR3B	NM_014971 // EFR3B // EFR3 homolog B (S. cerevisiae) // 2p23.3 // 22979 /// ENST0000040	0.000539973	0.250059
8163637	NM_002160	TNC	NM_002160 // TNC // tenascin C // 9q33 // 3371 /// ENST00000350763 // TNC // tenascin C	0.00160887	-0.236213
7956658	NM_004731	SLC16A7	NM_004731 // SLC16A7 // solute carrier family 16, member 7 (monocarboxylic acid transpo	0.000223354	-0.279997
8151816	NM_005261	GEM	NM_005261 // GEM // GTP binding protein overexpressed in skeletal muscle // 8q13-q21 //	0.00154459	-0.24634
8036890	NM_020956	PRX	NM_020956 // PRX // periaxin // 19q13.2 // 57716 /// NM_181882 // PRX // periaxin // 19	0.0019896	0.23616
8117890	NM_005516	HLA-E	NM_005516 // HLA-E // major histocompatibility complex, class I, E // 6p21.3 // 3133 //	0.000384472	0.268167
7912157	NM_018948	ERRFI1	NM_018948 // ERRFI1 // ERBB receptor feedback inhibitor 1 // 1p36 // 54206 /// ENST0000	0.00199398	-0.252994
8057797	NM_004657	SDPR	NM_004657 // SDPR // serum deprivation response // 2q32-q33 // 8436 /// ENST00000304141	0.000117817	0.289611
8128472	NM_006828	ASCC3	NM_006828 // ASCC3 // activating signal cointegrator 1 complex subunit 3 // 6q16 // 109	0.000388364	-0.271917
8141328	NR_033807	CYP3A5	NR_033807 // CYP3A5 // cytochrome P450, family 3, subfamily A, polypeptide 5 // 7q21.1	0.002468	0.244045
8103789	NM_005277	GPM6A	NM_005277 // GPM6A // glycoprotein M6A // 4q34 // 2823 /// NM_201591 // GPM6A // glycop	0.000201548	0.282399

8130867	NM_003247	THBS2	NM_003247 // THBS2 // thrombospondin 2 // 6q27 // 7058 /// ENST00000366787 // THBS2 //	0.000778706	-0.253643
8085774	NM_024697	ZNF385D	NM_024697 // ZNF385D // zinc finger protein 385D // 3p24.3 // 79750 /// ENST00000281523	0.00278995	-0.225922
8110932	NM_003966	SEMA5A	NM_003966 // SEMA5A // sema domain, seven thrombospondin repeats (type 1 and type 1-lik	0.00138858	0.2446
8044813	NM_183240	TMEM37	NM_183240 // TMEM37 // transmembrane protein 37 // 2q14.2 // 140738 /// ENST00000306406	0.00205521	0.247425
8147777	NM_138455	CTHRC1	NM_138455 // CTHRC1 // collagen triple helix repeat containing 1 // 8q22.3 // 115908 //	0.000565318	-0.258538
7989596	NM_014326	DAPK2	NM_014326 // DAPK2 // death-associated protein kinase 2 // 15q22.31 // 23604 /// ENST00	0.00195303	0.23666
8063437	NM_173485	TSHZ2	NM_173485 // TSHZ2 // teashirt zinc finger homeobox 2 // 20q13.2 // 128553 /// NM_00119	0.00256771	-0.229965
8073960	NM_001001852	PIM3	NM_001001852 // PIM3 // pim-3 oncogene // 22q13 // 415116 /// ENST00000360612 // PIM3 /	0.000253166	-0.305792
7980616	NM_007039	PTPN21	NM_007039 // PTPN21 // protein tyrosine phosphatase, non-receptor type 21 // 14q31.3 //	0.0022826	0.235879
7951271	NM_002421	MMP1	NM_002421 // MMP1 // matrix metallopeptidase 1 (interstitial collagenase) // 11q22.3 //	0.000529782	-0.268878
7999387	NM_001424	EMP2	NM_001424 // EMP2 // epithelial membrane protein 2 // 16p13.2 // 2013 /// ENST000003595	0.00109973	0.250442

7922326	NR_029627	MIR214	NR_029627 // MIR214 // microRNA 214 // 1q24.3 // 406996 /// NR_036066 // MIR3120 // mic	0.00118532	0.25955
8177788	NM_005516	HLA-E	NM_005516 // HLA-E // major histocompatibility complex, class I, E // 6p21.3 // 3133 //	0.000462825	0.268933
8179103	NM_005516	HLA-E	NM_005516 // HLA-E // major histocompatibility complex, class I, E // 6p21.3 // 3133 //	0.000462825	0.268933
7952268	NM_006288	THY1	NM_006288 // THY1 // Thy-1 cell surface antigen // 11q23.3 // 7070 /// ENST00000284240	0.000622791	-0.257734
8046861	NM_002210	ITGAV	NM_002210 // ITGAV // integrin, alpha V (vitronectin receptor, alpha polypeptide, antig	0.000215164	-0.281781
8179967	NM_001136	AGER	NM_001136 // AGER // advanced glycosylation end product-specific receptor // 6p21.3 //	0.000362724	0.26996
8178771	NM_001136	AGER	NM_001136 // AGER // advanced glycosylation end product-specific receptor // 6p21.3 //	0.000422072	0.267481
8125341	NM_001136	AGER	NM_001136 // AGER // advanced glycosylation end product-specific receptor // 6p21.3 //	0.000470111	0.265645
8044882	NM_020909	EPB41L5	NM_020909 // EPB41L5 // erythrocyte membrane protein band 4.1 like 5 // 2q14.2 // 57669	0.00105644	0.242345
7934215	NM_014767	SPOCK2	NM_014767 // SPOCK2 // sparc/osteonectin, cwcv and kazal-like domains proteoglycan (tes	0.00154334	0.247496
7958644	NM_170665	ATP2A2	NM_170665 // ATP2A2 // ATPase, Ca++ transporting, cardiac muscle, slow twitch 2 // 12q2	1.78E-05	-0.321805

7935230	NM_002860	ALDH18A1	NM_002860 // ALDH18A1 // aldehyde dehydrogenase 18 family, member A1 // 10q24.3 // 5832	0.000240798	-0.273776
7963410	NM_173086	KRT6C	NM_173086 // KRT6C // keratin 6C // 12q13.13 // 286887 /// NM_005555 // KRT6B // kerati	0.000718763	-0.266933
7900488	NR_029846	MIR30E	NR_029846 // MIR30E // microRNA 30e // 1p34.2 // 407034	0.00181628	0.254873
7960865	NM_006931	SLC2A3	NM_006931 // SLC2A3 // solute carrier family 2 (facilitated glucose transporter), membe	0.00268265	-0.248238
8095986	NM_005139	ANXA3	NM_005139 // ANXA3 // annexin A3 // 4q21.21 // 306 /// ENST00000264908 // ANXA3 // anne	0.000960189	0.256338
7954527	NM_020183	ARNTL2	NM_020183 // ARNTL2 // aryl hydrocarbon receptor nuclear translocator-like 2 // 12p12.2	0.000151168	-0.288009
8089835	NM_007085	FSTL1	NM_007085 // FSTL1 // follistatin-like 1 // 3q13.33 // 11167 /// NR_029584 // MIR198 //	0.0028294	-0.231796
8092594	---		---	0.00269191	0.239206
8171427	NM_004469	FIGF	NM_004469 // FIGF // c-fos induced growth factor (vascular endothelial growth factor D)	2.56E-05	0.322772
8135774	NM_002851	PTPRZ1	NM_002851 // PTPRZ1 // protein tyrosine phosphatase, receptor-type, Z polypeptide 1 //	0.00063792	-0.271025
8052654	NM_020651	PELI1	NM_020651 // PELI1 // pellino homolog 1 (Drosophila) // 2p13.3 // 57162 /// ENST0000035	0.00100256	-0.275416
8156043	NM_058179	PSAT1	NM_058179 // PSAT1 // phosphoserine aminotransferase 1 // 9q21.2 // 29968 /// NM_021154	0.000125772	-0.291864

7937892	NR_027015	PGAP2	NR_027015 // PGAP2 // post-GPI attachment to proteins 2 // 11p15.5 // 27315 /// NM_0144	0.00263208	0.236923
8092230	NM_022470	ZMAT3	NM_022470 // ZMAT3 // zinc finger, matrin-type 3 // 3q26.32 // 64393 /// NM_152240 // Z	0.00108321	-0.254478
8158627	NM_014286	NCS1	NM_014286 // NCS1 // neuronal calcium sensor 1 // 9q34 // 23413 /// NM_001128826 // NCS	0.000486609	-0.264829
8038861	NM_001245	SIGLEC6	NM_001245 // SIGLEC6 // sialic acid binding Ig-like lectin 6 // 19q13.3 // 946 /// NM_1	0.00126293	0.271276
8035304	NM_004335	BST2	NM_004335 // BST2 // bone marrow stromal cell antigen 2 // 19p13.1 // 684 /// ENST00000	0.00211412	0.243922
8149555	NM_015310	PSD3	NM_015310 // PSD3 // pleckstrin and Sec7 domain containing 3 // 8p21.3 // 23362 /// NM_	0.000270021	-0.279328
8089467	NM_024508	ZBED2	NM_024508 // ZBED2 // zinc finger, BED-type containing 2 // 3q13.2 // 79413 /// ENST000	6.15E-05	0.302006
8015706	NR_024461	LOC100190938	NR_024461 // LOC100190938 // hypothetical LOC100190938 // 17q21.31 // 100190938 /// NR_	0.00177332	0.241136
8122334	NM_178445	CCRL1	NM_178445 // CCRL1 // chemokine (C-C motif) receptor-like 1 // 3q22 // 51554 /// NM_016	0.000419508	0.280401
7967117	NM_003733	OASL	NM_003733 // OASL // 2'-5'-oligoadenylate synthetase-like // 12q24.2 // 8638 /// NM_198	0.00234097	0.242485
7935027	NM_004969	IDE	NM_004969 // IDE // insulin-degrading enzyme // 10q23-q25 // 3416 /// NM_001165946 // I	0.000437007	-0.270618

7952426	NM_014312	VSIG2	NM_014312 // VSIG2 // V-set and immunoglobulin domain containing 2 // 11q24 // 23584 //	0.00156307	0.252508
8157804	NM_182487	OLFML2A	NM_182487 // OLFML2A // olfactomedin-like 2A // 9q33.3 // 169611 /// ENST00000373580 //	0.000151314	0.288776
8124848	NM_003897	IER3	NM_003897 // IER3 // immediate early response 3 // 6p21.3 // 8870 /// ENST00000259874 /	0.00261237	-0.248831
8133518	NM_000265	NCF1	NM_000265 // NCF1 // neutrophil cytosolic factor 1 // 7q11.23 // 653361 /// NR_003187 /	0.00137734	0.249236
7939492	NM_001145033	C11orf96	NM_001145033 // C11orf96 // chromosome 11 open reading frame 96 // 11p11.2 // 387763 //	3.88E-05	-0.34021
7918900	---		---	0.000396898	0.293798
7981142	NM_024734	CLMN	NM_024734 // CLMN // calmin (calponin-like, transmembrane) // 14q32.13 // 79789 /// ENS	0.00124437	-0.258975
8126729	NM_001114086	CLIC5	NM_001114086 // CLIC5 // chloride intracellular channel 5 // 6p12.3 // 53405 /// NM_016	0.000120336	0.296392
8178435	NM_003897	IER3	NM_003897 // IER3 // immediate early response 3 // 6p21.3 // 8870 /// ENST00000259874 /	0.00174133	-0.25867
7931930	NM_006257	PRKCQ	NM_006257 // PRKCQ // protein kinase C, theta // 10p15 // 5588 /// ENST00000263125 // P	0.00273002	0.240638
7996883	NM_005329	HAS3	NM_005329 // HAS3 // hyaluronan synthase 3 // 16q22.1 // 3038 /// NM_138612 // HAS3 //	0.00139894	0.248241
8079305	NM_003278	CLEC3B	NM_003278 // CLEC3B // C-type lectin domain family 3, member B // 3p22-p21.3 // 7123 //	0.00253068	0.241644

7994826	NM_002209	ITGAL	NM_002209 // ITGAL // integrin, alpha L (antigen CD11A (p180), lymphocyte function-asso	0.00210123	0.240629
8092578	NM_004454	ETV5	NM_004454 // ETV5 // ets variant 5 // 3q28 // 2119 /// ENST00000306376 // ETV5 // ets v	0.000782195	0.262142
8009096	NM_000789	ACE	NM_000789 // ACE // angiotensin I converting enzyme (peptidyl-dipeptidase A) 1 // 17q23	0.000154348	0.280617
8067944	NR_029480	MIRLET7C	NR_029480 // MIRLET7C // microRNA let-7c // 21q21.1 // 406885	0.00132609	0.264268
8077441	NM_003670	BHLHE40	NM_003670 // BHLHE40 // basic helix-loop-helix family, member e40 // 3p26 // 8553 /// E	0.000515028	-0.28755
8135069	NM_000602	SERPINE1	NM_000602 // SERPINE1 // serpin peptidase inhibitor, clade E (nexin, plasminogen activa	5.49E-05	-0.331487
7933855	NM_145307	RTKN2	NM_145307 // RTKN2 // rhotekin 2 // 10q21.2 // 219790 /// ENST00000373789 // RTKN2 // r	4.09E-05	0.311878
8129392	NM_001012279	C6orf174	NM_001012279 // C6orf174 // chromosome 6 open reading frame 174 // 6q22.33 // 387104 //	0.00272528	0.230169
8171297	NM_000381	MID1	NM_000381 // MID1 // midline 1 (Opitz/BBB syndrome) // Xp22 // 4281 /// NM_033290 // MI	0.00039496	-0.285547
7907861	NM_004736	XPR1	NM_004736 // XPR1 // xenotropic and polytropic retrovirus receptor 1 // 1q25.1 // 9213	0.00184215	-0.250638
8067942	NR_029514	MIR99A	NR_029514 // MIR99A // microRNA 99a // 21q21.1 // 407055	0.0022402	0.251106
7965873	NM_001111283	IGF1	NM_001111283 // IGF1 // insulin-like growth factor 1 (somatomedin C) // 12q23.2 // 3479	0.00047787	-0.266635

8085914	NM_003615	SLC4A7	NM_003615 // SLC4A7 // solute carrier family 4, sodium bicarbonate cotransporter, membe	6.21E-05	-0.308091
7917875	NM_001993	F3	NM_001993 // F3 // coagulation factor III (thromboplastin, tissue factor) // 1p22-p21 /	0.000257189	-0.308386
7955110	AK125945	DKFZP779L1853	AK125945 // DKFZP779L1853 // hypothetical LOC643162 // 12q13.11 // 643162	0.000389745	0.286033
8051413	NM_015475	FAM98A	NM_015475 // FAM98A // family with sequence similarity 98, member A // 2p22.3 // 25940	0.00162795	-0.250007
7902441	NM_030965	ST6GALNAC5	NM_030965 // ST6GALNAC5 // ST6 (alpha-N-acetyl-neuraminyl-2,3-beta-galactosyl-1,3)-N-ac	0.00125185	0.250509
8043504	NM_002371	MAL	NM_002371 // MAL // mal, T-cell differentiation protein // 2cen-q13 // 4118 /// NM_0224	0.000341416	0.291943
8162313	NM_013417	IARS	NM_013417 // IARS // isoleucyl-tRNA synthetase // 9q21 // 3376 /// NM_002161 // IARS //	0.000395191	-0.275857
8166607	---		---	0.000539995	0.291993
7946589	NM_130385	MRVI1	NM_130385 // MRVI1 // murine retrovirus integration site 1 homolog // 11p15 // 10335 //	0.000107686	-0.30252
8082597	NM_001102608	COL6A6	NM_001102608 // COL6A6 // collagen, type VI, alpha 6 // 3q22.1 // 131873 /// ENST000003	0.000784517	0.27143
8113130	NM_024717	MCTP1	NM_024717 // MCTP1 // multiple C2 domains, transmembrane 1 // 5q15 // 79772 /// NM_0010	1.44E-05	-0.344142
7962183	NM_001005353	AK4	NM_001005353 // AK4 // adenylate kinase 4 // 1p31.3 // 205 /// NM_013410 // AK4 // aden	0.00179646	-0.250694

8018864	NM_003955	SOCS3	NM_003955 // SOCS3 // suppressor of cytokine signaling 3 // 17q25.3 // 9021 /// ENST000	0.0014383	-0.266743
8082465	NM_024768	CCDC48	NM_024768 // CCDC48 // coiled-coil domain containing 48 // 3q21.3 // 79825 /// AK022119	3.29E-06	0.355735
8102482	NM_014822	SEC24D	NM_014822 // SEC24D // SEC24 family, member D (S. cerevisiae) // 4q26 // 9871 /// ENST0	1.98E-08	-0.411078
8140227	NM_000265	NCF1	NM_000265 // NCF1 // neutrophil cytosolic factor 1 // 7q11.23 // 653361 /// NR_003187 /	0.00262209	0.238236
8133314	NM_000265	NCF1	NM_000265 // NCF1 // neutrophil cytosolic factor 1 // 7q11.23 // 653361 /// NR_003187 /	0.00286301	0.236128
7952341	NM_024769	ASAM	NM_024769 // ASAM // adipocyte-specific adhesion molecule // 11q24.1 // 79827 /// ENST0	0.000482086	-0.277805
8103951	NM_001995	ACSL1	NM_001995 // ACSL1 // acyl-CoA synthetase long-chain family member 1 // 4q35 // 2180 //	0.00150285	-0.260757
7916654	NM_181712	KANK4	NM_181712 // KANK4 // KN motif and ankyrin repeat domains 4 // 1p31.3 // 163782 /// ENS	0.000662024	0.278147
8110112	---		---	0.00148315	-0.267704
7934161	NM_005041	PRF1	NM_005041 // PRF1 // perforin 1 (pore forming protein) // 10q22 // 5551 /// NM_00108311	2.10E-05	0.327731
8139488	NM_001013398	IGFBP3	NM_001013398 // IGFBP3 // insulin-like growth factor binding protein 3 // 7p13-p12 // 3	0.00246445	-0.237491
7968004	NM_000231	SGCG	NM_000231 // SGCG // sarcoglycan, gamma (35kDa dystrophin-associated glycoprotein) // 1	3.34E-06	0.35247

8150592	NM_005195	CEBPD	NM_005195 // CEBPD // CCAAT/enhancer binding protein (C/EBP), delta // 8p11.2-p11.1 //	0.00224346	-0.251751
8046086	NM_203463	LASS6	NM_203463 // LASS6 // LAG1 homolog, ceramide synthase 6 // 2q24.3 // 253782 /// ENST000	0.000860696	-0.26574
8067140	NM_000782	CYP24A1	NM_000782 // CYP24A1 // cytochrome P450, family 24, subfamily A, polypeptide 1 // 20q13	0.00278731	-0.240082
8123562	NM_001500	GMDS	NM_001500 // GMDS // GDP-mannose 4,6-dehydratase // 6p25 // 2762 /// ENST00000380815 //	5.11E-05	-0.311059
8109086	NM_000024	ADRB2	NM_000024 // ADRB2 // adrenergic, beta-2-, receptor, surface // 5q31-q32 // 154 /// ENS	3.14E-05	0.323073
8035506	NM_004750	CRLF1	NM_004750 // CRLF1 // cytokine receptor-like factor 1 // 19p12 // 9244 /// ENST00000392	0.000288094	-0.285676
8123006	NM_003898	SYNJ2	NM_003898 // SYNJ2 // synaptojanin 2 // 6q25.3 // 8871 /// NM_001178088 // SYNJ2 // syn	0.00104916	-0.254684
7996546	NM_018296	LRRC36	NM_018296 // LRRC36 // leucine rich repeat containing 36 // 16q22.1 // 55282 /// NM_001	0.000541393	0.271425
8097388	NM_018078	LARP1B	NM_018078 // LARP1B // La ribonucleoprotein domain family, member 1B // 4q28.2 // 55132	0.000359857	-0.285535
7994280	NM_000418	IL4R	NM_000418 // IL4R // interleukin 4 receptor // 16p12.1-p11.2 // 3566 /// NM_001008699 /	0.000337711	-0.302
8132725	NM_003364	UPP1	NM_003364 // UPP1 // uridine phosphorylase 1 // 7p12.3 // 7378 /// NM_181597 // UPP1 //	0.000583072	-0.284567

8091780	NM_001038628	B3GALNT1	NM_001038628 // B3GALNT1 // beta-1,3-N-acetylgalactosaminyltransferase 1 (globoside blo	0.00255892	0.239901
8074991	NM_001099781	GGT5	NM_001099781 // GGT5 // gamma-glutamyltransferase 5 // 22q11.23 // 2687 /// NM_004121 /	0.000487875	-0.266031
8114898	---		---	0.000843124	0.279177
8077786	NM_001570	IRAK2	NM_001570 // IRAK2 // interleukin-1 receptor-associated kinase 2 // 3p25.3 // 3656 ///	0.00198745	-0.259936
8157487	NM_002581	PAPPA	NM_002581 // PAPPA // pregnancy-associated plasma protein A, pappalysin 1 // 9q33.2 //	0.000252545	-0.282187
7945894	---		---	0.00258757	0.248633
8157905	NM_033446	FAM125B	NM_033446 // FAM125B // family with sequence similarity 125, member B // 9q33.3 // 8985	0.000414497	0.280311
8148184	NM_032899	FAM83A	NM_032899 // FAM83A // family with sequence similarity 83, member A // 8q24.13 // 84985	0.00230854	-0.247923
7984174	NM_024798	SNX22	NM_024798 // SNX22 // sorting nexin 22 // 15q22.31 // 79856 /// NM_000942 // PPIB // pe	0.00156406	0.251353
8038809	NM_005601	NKG7	NM_005601 // NKG7 // natural killer cell group 7 sequence // 19q13.41 // 4818 /// ENST0	1.88E-05	0.332348
8096489	NM_006457	PDLIM5	NM_006457 // PDLIM5 // PDZ and LIM domain 5 // 4q22 // 10611 /// NM_001011513 // PDLIM5	0.00084022	-0.261218
7910427	NM_004481	GALNT2	NM_004481 // GALNT2 // UDP-N-acetyl-alpha-D-galactosamine:polypeptide N-acetylgalactosa	0.000712025	-0.277953

8171248	NM_000216	KAL1	NM_000216 // KAL1 // Kallmann syndrome 1 sequence // Xp22.32 // 3730 /// ENST0000026264	0.000263792	0.284232
8162117	NM_016548	GOLM1	NM_016548 // GOLM1 // golgi membrane protein 1 // 9q21.33 // 51280 /// NM_177937 // GOL	0.00115362	-0.256602
8146863	NM_001128205	SULF1	NM_001128205 // SULF1 // sulfatase 1 // 8q13.1 // 23213 /// NM_015170 // SULF1 // sulfa	0.000190996	-0.288174
8099685	NM_018176	LGI2	NM_018176 // LGI2 // leucine-rich repeat LGI family, member 2 // 4p15.2 // 55203 /// EN	9.48E-06	-0.343787
8059905	NM_004369	COL6A3	NM_004369 // COL6A3 // collagen, type VI, alpha 3 // 2q37 // 1293 /// NM_057167 // COL6	0.00131942	-0.253563
7971661	NR_029485	MIR15A	NR_029485 // MIR15A // microRNA 15a // 13q14.2 // 406948	2.46E-05	0.343131
7929750	NM_020354	ENTPD7	NM_020354 // ENTPD7 // ectonucleoside triphosphate diphosphohydrolase 7 // --- // 57089	2.20E-06	-0.363916
7966462	NM_024953	NAA25	NM_024953 // NAA25 // N(alpha)-acetyltransferase 25, NatB auxiliary subunit // 12q24.13	3.75E-05	-0.329831
8028908	NM_025194	ITPKC	NM_025194 // ITPKC // inositol 1,4,5-trisphosphate 3-kinase C // 19q13.1 // 80271 /// E	0.000147448	-0.312134
7929145	NM_182765	HECTD2	NM_182765 // HECTD2 // HECT domain containing 2 // 10q23.32 // 143279 /// NM_173497 //	0.000835001	-0.270244
8095907	NM_025074	FRAS1	NM_025074 // FRAS1 // Fraser syndrome 1 // 4q21.21 // 80144 /// NM_001166133 // FRAS1 /	0.000492066	0.285532

8159900	NM_152629	GLIS3	NM_152629 // GLIS3 // GLIS family zinc finger 3 // 9p24.2 // 169792 /// NM_001042413 //	9.38E-05	-0.31159
7936968	NM_003474	ADAM12	NM_003474 // ADAM12 // ADAM metallopeptidase domain 12 // 10q26.3 // 8038 /// NM_021641	0.00108009	-0.257309
8138363	NM_015464	SOSTDC1	NM_015464 // SOSTDC1 // sclerostin domain containing 1 // 7p21.1 // 25928 /// ENST00000	0.00236928	0.24927
8170468	NM_005342	HMGB3	NM_005342 // HMGB3 // high-mobility group box 3 // Xq28 // 3149 /// ENST00000325307 //	0.000720424	-0.269649
8025918	NM_001299	CNN1	NM_001299 // CNN1 // calponin 1, basic, smooth muscle // 19p13.2-p13.1 // 1264 /// ENST	0.000407021	-0.283195
8136889	---		---	0.000959313	0.269639
8140668	NM_006080	SEMA3A	NM_006080 // SEMA3A // sema domain, immunoglobulin domain (Ig), short basic domain, sec	1.43E-06	-0.374731
8121569	AK091822	FLJ34503	AK091822 // FLJ34503 // hypothetical FLJ34503 // 6q21 // 285759	0.002157	0.242584
8057486	NM_005019	PDE1A	NM_005019 // PDE1A // phosphodiesterase 1A, calmodulin-dependent // 2q32.1 // 5136 ///	0.00216009	-0.245637
8155327	NM_000692	ALDH1B1	NM_000692 // ALDH1B1 // aldehyde dehydrogenase 1 family, member B1 // 9p11.1 // 219 ///	0.00292653	-0.238251
8061653	NM_080625	C20orf160	NM_080625 // C20orf160 // chromosome 20 open reading frame 160 // 20q11.2 // 140706 ///	0.000678161	0.269206
8035517	NM_000095	COMP	NM_000095 // COMP // cartilage oligomeric matrix protein // 19p13.1 // 1311 /// ENST000	1.38E-05	-0.333924

7911114	NM_018012	KIF26B	NM_018012 // KIF26B // kinesin family member 26B // 1q44 // 55083 /// ENST00000407071 /	0.000144978	-0.302725
7989277	NM_004998	MYO1E	NM_004998 // MYO1E // myosin IE // 15q21-q22 // 4643 /// ENST00000288235 // MYO1E // my	9.17E-07	-0.375024
7969640	NM_182848	CLDN10	NM_182848 // CLDN10 // claudin 10 // 13q31-q34 // 9071 /// NM_001160100 // CLDN10 // cl	0.000377939	-0.284696
8156373	NM_001083536	FGD3	NM_001083536 // FGD3 // FYVE, RhoGEF and PH domain containing 3 // --- // 89846 /// NM_	0.000132647	0.306715
8061416	NM_003650	CST7	NM_003650 // CST7 // cystatin F (leukocystatin) // 20p11.21 // 8530 /// ENST00000480798	0.00227373	0.250699
7965110	---		---	0.00189073	0.244538
8057620	NM_000393	COL5A2	NM_000393 // COL5A2 // collagen, type V, alpha 2 // 2q14-q32 // 1290 /// ENST0000037486	0.000626425	-0.270043
8089261	NM_170662	CBLB	NM_170662 // CBLB // Cas-Br-M (murine) ecotropic retroviral transforming sequence b //	0.00250579	-0.243716
7897460	NM_032315	SLC25A33	NM_032315 // SLC25A33 // solute carrier family 25, member 33 // 1p36.22 // 84275 /// EN	0.00231951	-0.261238
8151496	NM_001033723	ZNF704	NM_001033723 // ZNF704 // zinc finger protein 704 // 8q21.13 // 619279 /// ENST00000327	0.000226957	0.295641
8086799	NM_006574	CSPG5	NM_006574 // CSPG5 // chondroitin sulfate proteoglycan 5 (neuroglycan C) // 3p21.3 // 1	0.000749517	-0.285575
7948088	NM_032315	SLC25A33	NM_032315 // SLC25A33 // solute carrier family 25, member 33 // 1p36.22 // 84275 /// EN	0.00248472	-0.25939

8118571	NM_002800	PSMB9	NM_002800 // PSMB9 // proteasome (prosome, macropain) subunit, beta type, 9 (large mult	0.0012615	0.263092
8178211	NM_002800	PSMB9	NM_002800 // PSMB9 // proteasome (prosome, macropain) subunit, beta type, 9 (large mult	0.0012615	0.263092
8179495	NM_002800	PSMB9	NM_002800 // PSMB9 // proteasome (prosome, macropain) subunit, beta type, 9 (large mult	0.0012615	0.263092
8023575	NM_133459	CCBE1	NM_133459 // CCBE1 // collagen and calcium binding EGF domains 1 // 18q21.32 // 147372	0.00169092	0.245448
8152617	NM_005328	HAS2	NM_005328 // HAS2 // hyaluronan synthase 2 // 8q24.12 // 3037 /// ENST00000303924 // HA	0.000392508	-0.279723
7951259	NM_002425	MMP10	NM_002425 // MMP10 // matrix metallopeptidase 10 (stromelysin 2) // 11q22.3 // 4319 ///	0.000904432	-0.268305
8070912	NM_194255	SLC19A1	NM_194255 // SLC19A1 // solute carrier family 19 (folate transporter), member 1 // 21q2	0.00196746	0.249219
7961546	NM_004447	EPS8	NM_004447 // EPS8 // epidermal growth factor receptor pathway substrate 8 // 12p12.3 //	0.000201413	-0.298178
7921677	NM_016382	CD244	NM_016382 // CD244 // CD244 molecule, natural killer cell receptor 2B4 // 1q23.3 // 517	1.74E-05	0.336344
8162276	NM_005384	NFIL3	NM_005384 // NFIL3 // nuclear factor, interleukin 3 regulated // 9q22 // 4783 /// ENST0	1.21E-06	-0.397085
7963946	NM_002429	MMP19	NM_002429 // MMP19 // matrix metallopeptidase 19 // 12q14 // 4327 /// ENST00000322569 /	0.000744577	-0.279714

8116980	NM_001165032	RNF182	NM_001165032 // RNF182 // ring finger protein 182 // 6p23 // 221687 /// NM_152737 // RN	0.00200264	0.240724
7929047	NM_001547	IFIT2	NM_001547 // IFIT2 // interferon-induced protein with tetratricopeptide repeats 2 // 10	0.000101789	0.302631
8175492	NM_173694	ATP11C	NM_173694 // ATP11C // ATPase, class VI, type 11C // Xq27.1 // 286410 /// NM_001010986	0.00231586	-0.245677
8050007	NM_012293	PXDN	NM_012293 // PXDN // peroxidasin homolog (Drosophila) // 2p25 // 7837 /// ENST000002528	0.000268739	-0.305385
8002342	---		---	0.00192884	-0.254444
8146957	NM_015886	PI15	NM_015886 // PI15 // peptidase inhibitor 15 // 8q21.11 // 51050 /// ENST00000260113 //	6.24E-08	-0.403008
7918558	NM_004980	KCND3	NM_004980 // KCND3 // potassium voltage-gated channel, Shal-related subfamily, member 3	4.32E-06	-0.352876
8156278	NM_005226	S1PR3	NM_005226 // S1PR3 // sphingosine-1-phosphate receptor 3 // 9q22.1-q22.2 // 1903 /// NM	0.00208521	-0.247005
8015366	NM_000526	KRT14	NM_000526 // KRT14 // keratin 14 // 17q12-q21 // 3861 /// ENST00000167586 // KRT14 // k	0.0024598	-0.242795
8098060	NM_021634	RXFP1	NM_021634 // RXFP1 // relaxin/insulin-like family peptide receptor 1 // 4q32.1 // 59350	0.000669755	0.271999
8044793	NM_182915	STEAP3	NM_182915 // STEAP3 // STEAP family member 3 // 2q14.2 // 55240 /// NM_018234 // STEAP3	0.000795565	-0.270359
7983630	NM_002009	FGF7	NM_002009 // FGF7 // fibroblast growth factor 7 // 15q21.2 // 2252 /// M60828 // FGF7 /	0.00227895	-0.248447

8034712	NM_024825	PODNL1	NM_024825 // PODNL1 // podocan-like 1 // 19p13.12 // 79883 /// NM_001146254 // PODNL1 /	0.00123853	-0.257311
7970441	NM_004004	GJB2	NM_004004 // GJB2 // gap junction protein, beta 2, 26kDa // 13q11-q12 // 2706 /// ENST0	0.00240853	-0.244621
7976496	NM_001085	SERPINA3	NM_001085 // SERPINA3 // serpin peptidase inhibitor, clade A (alpha-1 antiproteinase, a	1.39E-06	-0.369357
7935188	NM_001034954	SORBS1	NM_001034954 // SORBS1 // sorbin and SH3 domain containing 1 // 10q23.33 // 10580 /// N	0.00121627	-0.269096
7970569	NM_014363	SACS	NM_014363 // SACS // spastic ataxia of Charlevoix-Saguenay (sacsin) // 13q12 // 26278 /	0.000212353	-0.296031
8082408	NM_013336	SEC61A1	NM_013336 // SEC61A1 // Sec61 alpha 1 subunit (S. cerevisiae) // 3q21.3 // 29927 /// EN	0.00143566	-0.259662
8138834	NM_017946	FKBP14	NM_017946 // FKBP14 // FK506 binding protein 14, 22 kDa // 7p14.3 // 55033 /// ENST0000	0.00154757	-0.254099
8065416	NM_001322	CST2	NM_001322 // CST2 // cystatin SA // 20p11.21 // 1470 /// ENST00000304725 // CST2 // cys	0.00117818	-0.264104
8106098	NM_005909	MAP1B	NM_005909 // MAP1B // microtubule-associated protein 1B // 5q13 // 4131 /// ENST0000029	7.16E-07	-0.376291
8154962	NM_001135004	DNAJB5	NM_001135004 // DNAJB5 // DnaJ (Hsp40) homolog, subfamily B, member 5 // 9p13.3 // 2582	6.36E-05	-0.313455
8039842	NM_012312	KIR2DS2	NM_012312 // KIR2DS2 // killer cell immunoglobulin-like receptor, two domains, short cy	0.00103645	0.264629

7956737	NR_029661	MIRLET7I	NR_029661 // MIRLET7I // microRNA let-7i // 12q14.1 // 406891	0.000267715	0.299934
7996759	---		---	0.000852839	0.271518
8105340	NM_006144	GZMA	NM_006144 // GZMA // granzyme A (granzyme 1, cytotoxic T-lymphocyte-associated serine e	0.000137452	0.311011
7905553	NM_003125	SPRR1B	NM_003125 // SPRR1B // small proline-rich protein 1B // 1q21-q22 // 6699 /// ENST000003	0.000110605	-0.313609
7935425	NM_015179	RRP12	NM_015179 // RRP12 // ribosomal RNA processing 12 homolog (S. cerevisiae) // 10q24.1 //	0.00212594	-0.260076
8077299	NM_014461	CNTN6	NM_014461 // CNTN6 // contactin 6 // 3p26-p25 // 27255 /// ENST00000446702 // CNTN6 //	0.000148472	0.294236
7982366	NM_001144757	SCG5	NM_001144757 // SCG5 // secretogranin V (7B2 protein) // 15q13-q14 // 6447 /// NM_00302	0.000225286	-0.288052
8162283	NM_004560	ROR2	NM_004560 // ROR2 // receptor tyrosine kinase-like orphan receptor 2 // 9q22 // 4920 //	0.000190702	-0.29639
8052762	NM_002056	GFPT1	NM_002056 // GFPT1 // glutamine--fructose-6-phosphate transaminase 1 // 2p13 // 2673 //	0.000160243	-0.296772
8110265	NM_213647	FGFR4	NM_213647 // FGFR4 // fibroblast growth factor receptor 4 // 5q35.1-qter // 2264 /// NM	0.000567285	0.279238
8179049	NR_024240	HLA-J	NR_024240 // HLA-J // major histocompatibility complex, class I, J (pseudogene) // 6p21	0.00248514	0.24653
8061414	---		---	0.0026489	0.247818
7983922	---		---	0.00220381	-0.256677
8099471	NM_031950	FGFBP2	NM_031950 // FGFBP2 // fibroblast growth factor binding protein 2 // 4p16 // 83888 ///	0.000187621	0.298412

7939052	NM_203371	FIBIN	NM_203371 // FIBIN // fin bud initiation factor homolog (zebrafish) // 11p14.2 // 38775	7.22E-05	0.315529
8061013	NM_080826	ISM1	NM_080826 // ISM1 // isthmin 1 homolog (zebrafish) // 20p12.1 // 140862 /// ENST0000026	0.00199784	-0.243417
7973336	NM_004995	MMP14	NM_004995 // MMP14 // matrix metallopeptidase 14 (membrane-inserted) // 14q11-q12 // 43	0.000197532	-0.30214
7963421	NM_005554	KRT6A	NM_005554 // KRT6A // keratin 6A // 12q12-q13 // 3853 /// ENST00000330722 // KRT6A // k	0.000798665	-0.273965
7922162	NM_006996	SLC19A2	NM_006996 // SLC19A2 // solute carrier family 19 (thiamine transporter), member 2 // 1q	0.00200062	-0.261085
7951030	NR_003036	SNORD6	NR_003036 // SNORD6 // small nucleolar RNA, C/D box 6 // 11q21 // 692075	0.000508494	-0.287256
8173493	NM_001142797	CXCR3	NM_001142797 // CXCR3 // chemokine (C-X-C motif) receptor 3 // Xq13 // 2833 /// NM_0015	0.00085304	0.277785
7956878	NM_007199	IRAK3	NM_007199 // IRAK3 // interleukin-1 receptor-associated kinase 3 // 12q14.3 // 11213 //	5.20E-05	-0.343175
7991047	AK097109	LOC100131860	AK097109 // LOC100131860 // hypothetical protein LOC100131860 // 15q25.2 // 100131860	0.000810673	0.273873
8042503	NM_002357	MXD1	NM_002357 // MXD1 // MAX dimerization protein 1 // 2p13-p12 // 4084 /// ENST00000264444	0.00258198	-0.250036
8161044	NM_003289	TPM2	NM_003289 // TPM2 // tropomyosin 2 (beta) // 9p13 // 7169 /// NM_213674 // TPM2 // trop	1.88E-05	-0.34197

8122986	NM_016224	SNX9	NM_016224 // SNX9 // sorting nexin 9 // 6q25.1-q26 // 51429 /// ENST00000392185 // SNX9	5.66E-05	-0.316009
8041048	NM_005253	FOSL2	NM_005253 // FOSL2 // FOS-like antigen 2 // 2p23.3 // 2355 /// ENST00000264716 // FOSL2	1.41E-05	-0.352753
8000649	---		---	0.000842796	-0.274507
8000690	---		---	0.000842796	-0.274507
8135990	NM_001458	FLNC	NM_001458 // FLNC // filamin C, gamma // 7q32-q35 // 2318 /// NM_001127487 // FLNC // f	0.000152736	-0.301041
7903461	NM_001113226	NTNG1	NM_001113226 // NTNG1 // netrin G1 // 1p13.3 // 22854 /// NM_001113228 // NTNG1 // netr	0.000992464	0.273919
8004497	NM_001416	EIF4A1	NM_001416 // EIF4A1 // eukaryotic translation initiation factor 4A1 // 17p13 // 1973 //	0.000762021	-0.284082
8020806	NM_017831	RNF125	NM_017831 // RNF125 // ring finger protein 125 // 18q12.1 // 54941 /// ENST00000217740	0.00201709	0.248332
8022145	NM_173464	L3MBTL4	NM_173464 // L3MBTL4 // l(3)mbt-like 4 (Drosophila) // 18p11.31 // 91133 /// ENST000002	0.00185367	0.255132
8063923	NM_016354	SLCO4A1	NM_016354 // SLCO4A1 // solute carrier organic anion transporter family, member 4A1 //	0.000191427	-0.317534
7954645	NM_001080509	TSPAN11	NM_001080509 // TSPAN11 // tetraspanin 11 // 12p11.21 // 441631 /// ENST00000261177 //	0.00170444	-0.252233
7908496	NM_005666	CFHR2	NM_005666 // CFHR2 // complement factor H-related 2 // 1q31.3 // 3080 /// ENST000003674	0.0013109	-0.254943

8135594	NM_001753	CAV1	NM_001753 // CAV1 // caveolin 1, caveolae protein, 22kDa // 7q31.1 // 857 /// NM_001172	0.0019459	0.248898
8059720	NM_006056	NMUR1	NM_006056 // NMUR1 // neuromedin U receptor 1 // 2q37.1 // 10316 /// ENST00000305141 //	0.000274327	0.290387
8087881	NR_029660	MIRLET7G	NR_029660 // MIRLET7G // microRNA let-7g // 3p21.1 // 406890	0.000136716	0.314942
7974851	NM_001530	HIF1A	NM_001530 // HIF1A // hypoxia inducible factor 1, alpha subunit (basic helix-loop-helix	2.85E-09	-0.438835
8069943	NM_014825	URB1	NM_014825 // URB1 // URB1 ribosome biogenesis 1 homolog (S. cerevisiae) // 21q22.11 //	6.17E-05	-0.317545
8070557	NM_001098402	ZNF295	NM_001098402 // ZNF295 // zinc finger protein 295 // 21q22.3 // 49854 /// NM_020727 //	0.00261522	-0.246924
7928882	NM_006829	C10orf116	NM_006829 // C10orf116 // chromosome 10 open reading frame 116 // 10q23.2 // 10974 ///	4.83E-06	0.365418
8102594	NM_024873	TNIP3	NM_024873 // TNIP3 // TNFAIP3 interacting protein 3 // 4q27 // 79931 /// NM_001128843 /	0.00109899	-0.271431
8135955	NM_001219	CALU	NM_001219 // CALU // calumenin // 7q32.1 // 813 /// NM_001130674 // CALU // calumenin /	7.37E-06	-0.349454
7922610	NM_007314	ABL2	NM_007314 // ABL2 // v-abl Abelson murine leukemia viral oncogene homolog 2 // 1q25.2 /	1.19E-07	-0.425858
8031328	NM_006737	KIR3DL2	NM_006737 // KIR3DL2 // killer cell immunoglobulin-like receptor, three domains, long c	0.000965674	0.267131

8065136	NM_001042576	RRBP1	NM_001042576 // RRBP1 // ribosome binding protein 1 homolog 180kDa (dog) // 20p12 // 62	4.55E-05	-0.32906
7986446	NM_000693	ALDH1A3	NM_000693 // ALDH1A3 // aldehyde dehydrogenase 1 family, member A3 // 15q26.3 // 220 //	3.89E-07	-0.392473
7967993	NM_002010	FGF9	NM_002010 // FGF9 // fibroblast growth factor 9 (glia-activating factor) // 13q11-q12 /	0.00131048	0.262902
7961059	NM_002258	KLRB1	NM_002258 // KLRB1 // killer cell lectin-like receptor subfamily B, member 1 // 12p13 /	0.00015973	0.303893
8174767	NM_017938	FAM70A	NM_017938 // FAM70A // family with sequence similarity 70, member A // Xq24 // 55026 //	0.000414165	0.286329
8075569	NM_014227	SLC5A4	NM_014227 // SLC5A4 // solute carrier family 5 (low affinity glucose cotransporter), me	0.00277446	0.242237
8063458	NM_018431	DOK5	NM_018431 // DOK5 // docking protein 5 // 20q13.2 // 55816 /// ENST00000262593 // DOK5	0.00103443	-0.263655
7944082	NM_001001522	TAGLN	NM_001001522 // TAGLN // transgelin // 11q23.2 // 6876 /// NM_003186 // TAGLN // transg	4.22E-05	-0.328926
8083677	NM_014575	SCHIP1	NM_014575 // SCHIP1 // schwannomin interacting protein 1 // 3q25.32-q25.33 // 29970 ///	0.00245463	-0.256114
8153021	NM_003033	ST3GAL1	NM_003033 // ST3GAL1 // ST3 beta-galactoside alpha-2,3-sialyltransferase 1 // 8q24.22 /	0.000136873	-0.321266
8023043	NM_024430	PSTPIP2	NM_024430 // PSTPIP2 // proline-serine-threonine phosphatase interacting protein 2 // 1	0.000189365	-0.303496

8031297	NM_014218	KIR2DL1	NM_014218 // KIR2DL1 // killer cell immunoglobulin-like receptor, two domains, long cyt	0.000424371	0.286743
7978595	NM_013448	BAZ1A	NM_013448 // BAZ1A // bromodomain adjacent to zinc finger domain, 1A // 14q13.2 // 1117	0.000330035	-0.29777
8094870	NM_001080505	SHISA3	NM_001080505 // SHISA3 // shisa homolog 3 (Xenopus laevis) // 4p13 // 152573 /// ENST00	0.000692736	0.282984
8131803	NM_000600	IL6	NM_000600 // IL6 // interleukin 6 (interferon, beta 2) // 7p21 // 3569 /// ENST00000404	0.000832571	-0.280628
8040753	NM_017727	TMEM214	NM_017727 // TMEM214 // transmembrane protein 214 // 2p23.3 // 54867 /// NM_001083590 /	0.00210758	-0.255765
8118142	NM_000594	TNF	NM_000594 // TNF // tumor necrosis factor // 6p21.3 // 7124 /// ENST00000376122 // TNF	0.00254593	0.248522
8177983	NM_000594	TNF	NM_000594 // TNF // tumor necrosis factor // 6p21.3 // 7124 /// ENST00000376122 // TNF	0.00254593	0.248522
8179263	NM_000594	TNF	NM_000594 // TNF // tumor necrosis factor // 6p21.3 // 7124 /// ENST00000376122 // TNF	0.00254593	0.248522
7963880	NM_001144996	ITGA7	NM_001144996 // ITGA7 // integrin, alpha 7 // 12q13 // 3679 /// NM_002206 // ITGA7 // i	0.000101006	-0.309579
7950391	NM_173582	PGM2L1	NM_173582 // PGM2L1 // phosphoglucomutase 2-like 1 // 11q13.4 // 283209 /// ENST0000029	0.00037885	-0.285712
8080419	NM_206825	GNL3	NM_206825 // GNL3 // guanine nucleotide binding protein-like 3 (nucleolar) // 3p21.1 //	0.00060278	-0.278767

8043725	NM_001079	ZAP70	NM_001079 // ZAP70 // zeta-chain (TCR) associated protein kinase 70kDa // 2q12 // 7535	0.00236666	0.25005
7941879	NM_198517	TBC1D10C	NM_198517 // TBC1D10C // TBC1 domain family, member 10C // 11q13.2 // 374403 /// ENST00	0.00257042	0.249176
7982597	NM_003246	THBS1	NM_003246 // THBS1 // thrombospondin 1 // 15q15 // 7057 /// ENST00000260356 // THBS1 //	2.03E-06	-0.394499
8049044	NM_025139	ARMC9	NM_025139 // ARMC9 // armadillo repeat containing 9 // 2q37.1 // 80210 /// ENST00000349	0.00168211	-0.258957
7903777	NM_000851	GSTM5	NM_000851 // GSTM5 // glutathione S-transferase mu 5 // 1p13.3 // 2949 /// ENST00000256	0.00213531	0.260076
8167185	NM_003254	TIMP1	NM_003254 // TIMP1 // TIMP metallopeptidase inhibitor 1 // Xp11.3-p11.23 // 7076 /// EN	2.06E-05	-0.33716
7899462	NM_001048194	RCC1	NM_001048194 // RCC1 // regulator of chromosome condensation 1 // 1p36.1 // 1104 /// NR	5.54E-05	-0.338622
8109612	NM_000679	ADRA1B	NM_000679 // ADRA1B // adrenergic, alpha-1B-, receptor // 5q33.3 // 147 /// ENST0000030	0.00144993	0.261429
7933672	NM_001142763	PCDH15	NM_001142763 // PCDH15 // protocadherin-related 15 // 10q21.1 // 65217 /// NM_001142764	0.00068211	0.286638
8035201	NM_015692	CPAMD8	NM_015692 // CPAMD8 // C3 and PZP-like, alpha-2-macroglobulin domain containing 8 // 19	0.000215574	0.290056
8110090	NM_022754	SFXN1	NM_022754 // SFXN1 // sideroflexin 1 // --- // 94081 /// ENST00000321442 // SFXN1 // si	0.00257309	-0.247808

8032157	NM_014963	SBNO2	NM_014963 // SBNO2 // strawberry notch homolog 2 (Drosophila) // 19p13.3 // 22904 /// N	0.000970005	-0.282272
8101881	NM_000668	ADH1B	NM_000668 // ADH1B // alcohol dehydrogenase 1B (class I), beta polypeptide // 4q23 // 1	1.32E-05	0.3663
8126855	NM_207499	C6orf138	NM_207499 // C6orf138 // chromosome 6 open reading frame 138 // 6p12.3 // 442213 /// NM	0.000295217	-0.289503
8103975	NR_003542	SLED1	NR_003542 // SLED1 // proteoglycan 3 pseudogene // 4q35.1 // 643036 /// AY358224 // SLE	0.00102349	-0.281308
7928589	NM_005729	PPIF	NM_005729 // PPIF // peptidylprolyl isomerase F // 10q22-q23 // 10105 /// ENST000002251	8.40E-05	-0.326279
8063000	NM_006103	WFDC2	NM_006103 // WFDC2 // WAP four-disulfide core domain 2 // 20q12-q13.2 // 10406 /// ENST	0.00258053	-0.249767
7922040	NM_198053	CD247	NM_198053 // CD247 // CD247 molecule // 1q22-q23 // 919 /// NM_000734 // CD247 // CD247	1.26E-06	0.386843
7926105	NM_001002295	GATA3	NM_001002295 // GATA3 // GATA binding protein 3 // 10p15 // 2625 /// NM_002051 // GATA3	0.000155636	0.302281
8143684	NM_004911	PDIA4	NM_004911 // PDIA4 // protein disulfide isomerase family A, member 4 // 7q35 // 9601 //	0.00178502	-0.252556
8106986	NM_014899	RHOBTB3	NM_014899 // RHOBTB3 // Rho-related BTB domain containing 3 // 5q15 // 22836 /// ENST00	0.000316966	-0.302903
8094301	NM_004787	SLIT2	NM_004787 // SLIT2 // slit homolog 2 (Drosophila) // 4p15.2 // 9353 /// ENST00000504154	0.000216404	0.303662

8156199	NM_004938	DAPK1	NM_004938 // DAPK1 // death-associated protein kinase 1 // 9q34.1 // 1612 /// ENST00000	0.00154624	0.254708
8159854	NM_014878	KIAA0020	NM_014878 // KIAA0020 // KIAA0020 // 9p24.2 // 9933 /// ENST00000397885 // KIAA0020 //	0.000149766	-0.316603
7929990	NM_015062	PPRC1	NM_015062 // PPRC1 // peroxisome proliferator-activated receptor gamma, coactivator-rel	0.00128878	-0.273306
8175195	NM_001077188	HS6ST2	NM_001077188 // HS6ST2 // heparan sulfate 6-O-sulfotransferase 2 // Xq26.2 // 90161 ///	0.00126989	-0.260431
7963614	NM_000889	ITGB7	NM_000889 // ITGB7 // integrin, beta 7 // 12q13.13 // 3695 /// ENST00000422257 // ITGB7	0.00165073	0.266003
7903358	NM_001078	VCAM1	NM_001078 // VCAM1 // vascular cell adhesion molecule 1 // 1p32-p31 // 7412 /// NM_0806	0.000580732	-0.274302
8084742	NM_005578	LPP	NM_005578 // LPP // LIM domain containing preferred translocation partner in lipoma //	0.000900195	-0.271582
7953892	NM_016523	KLRF1	NM_016523 // KLRF1 // killer cell lectin-like receptor subfamily F, member 1 // 12p13.3	1.02E-06	0.381999
7905088	NM_003517	HIST2H2AC	NM_003517 // HIST2H2AC // histone cluster 2, H2ac // 1q21.2 // 8338 /// ENST00000331380	0.000994585	0.26504
8006433	NM_002982	CCL2	NM_002982 // CCL2 // chemokine (C-C motif) ligand 2 // 17q11.2-q12 // 6347 /// ENST0000	0.000579406	-0.278058
8074780	NM_013313	YPEL1	NM_013313 // YPEL1 // yippee-like 1 (Drosophila) // 22q11.2 // 29799 /// NM_148175 // P	1.95E-06	0.364776
7927732	NM_032199	ARID5B	NM_032199 // ARID5B // AT rich interactive domain 5B (MRF1-like) // 10q21.2 // 84159 //	0.00053755	-0.26653

7921900	NM_053282	SH2D1B	NM_053282 // SH2D1B // SH2 domain containing 1B // 1q23.3 // 117157 /// ENST00000367929	0.000216556	0.29827
8020762	NM_001944	DSG3	NM_001944 // DSG3 // desmoglein 3 // 18q12.1 // 1830 /// ENST00000257189 // DSG3 // des	0.00127446	-0.265747
8126820	NM_153840	GPR110	NM_153840 // GPR110 // G protein-coupled receptor 110 // 6p12.3 // 266977 /// NM_025048	0.000177247	-0.305504
7951977	NM_001164836	FXYD6	NM_001164836 // FXYD6 // FXYD domain containing ion transport regulator 6 // 11q23.3 //	0.00191762	0.258512
7902227	NM_001924	GADD45A	NM_001924 // GADD45A // growth arrest and DNA-damage-inducible, alpha // 1p31.2 // 1647	2.37E-09	-0.442895
7925622	NM_015446	AHCTF1	NM_015446 // AHCTF1 // AT hook containing transcription factor 1 // 1q44 // 25909 /// A	0.00115499	-0.27152
7902541	NM_006820	IFI44L	NM_006820 // IFI44L // interferon-induced protein 44-like // 1p31.1 // 10964 /// ENST00	0.00180164	0.256792
8166469	NR_027783	SAT1	NR_027783 // SAT1 // spermidine/spermine N1-acetyltransferase 1 // Xp22.1 // 6303 /// N	0.000562293	-0.284737
8148317	NM_002467	MYC	NM_002467 // MYC // v-myc myelocytomatosis viral oncogene homolog (avian) // 8q24.21 //	0.000135092	-0.310471
8065758	AK096092	FLJ38773	AK096092 // FLJ38773 // hypothetical protein FLJ38773 // 20q11.22 // 284808	0.00291319	0.251891
8149885	NM_000680	ADRA1A	NM_000680 // ADRA1A // adrenergic, alpha-1A-, receptor // 8p21.2 // 148 /// NM_033303 /	1.12E-06	0.374822
7979031	---		---	0.0014996	-0.268857

8039871	NM_001083539	KIR3DS1	NM_001083539 // KIR3DS1 // killer cell immunoglobulin-like receptor, three domains, sho	0.000296144	0.296133
8117476	NM_006994	BTN3A3	NM_006994 // BTN3A3 // butyrophilin, subfamily 3, member A3 // 6p21.3 // 10384 /// NM_1	0.000241437	0.298598
8069142	NM_198687	KRTAP10-4	NM_198687 // KRTAP10-4 // keratin associated protein 10-4 // 21q22.3 // 386672 /// ENST	0.000696987	0.284595
7988260	NM_032892	FRMD5	NM_032892 // FRMD5 // FERM domain containing 5 // 15q15.3 // 84978 /// ENST00000417257	0.00012247	-0.309469
8111677	NM_002310	LIFR	NM_002310 // LIFR // leukemia inhibitory factor receptor alpha // 5p13-p12 // 3977 ///	0.000738444	0.27987
8020903	NM_020474	GALNT1	NM_020474 // GALNT1 // UDP-N-acetyl-alpha-D-galactosamine:polypeptide N-acetylgalactosa	0.00224766	-0.253312
8106743	NM_004385	VCAN	NM_004385 // VCAN // versican // 5q14.3 // 1462 /// NM_001164097 // VCAN // versican //	1.01E-06	-0.381242
8102988	NM_198682	GYPE	NM_198682 // GYPE // glycophorin E (MNS blood group) // 4q31.1 // 2996 /// NM_002102 //	0.000145034	0.305701
7930921	NM_004281	BAG3	NM_004281 // BAG3 // BCL2-associated athanogene 3 // 10q25.2-q26.2 // 9531 /// ENST0000	2.25E-05	-0.343579
7995803	NR_036677	MT1JP	NR_036677 // MT1JP // metallothionein 1J (pseudogene) // 16q13 // 4498 /// AF348994 //	0.000833042	-0.282629
7962146	NM_001135811	FAM60A	NM_001135811 // FAM60A // family with sequence similarity 60, member A // 12p11 // 5851	0.00205712	-0.256185

8050102	NM_207315	CMPK2	NM_207315 // CMPK2 // cytidine monophosphate (UMP-CMP) kinase 2, mitochondrial // 2p25.	0.000196186	0.302905
7973067	NM_000270	PNP	NM_000270 // PNP // purine nucleoside phosphorylase // 14q13.1 // 4860 /// ENST00000361	0.000166047	-0.316064
8097461	NM_012118	CCRN4L	NM_012118 // CCRN4L // CCR4 carbon catabolite repression 4-like (S. cerevisiae) // 4q31	6.38E-05	-0.333297
7970810	NM_003045	SLC7A1	NM_003045 // SLC7A1 // solute carrier family 7 (cationic amino acid transporter, y+ sys	0.000140675	-0.305806
7932407	NM_001004470	ST8SIA6	NM_001004470 // ST8SIA6 // ST8 alpha-N-acetyl-neuraminide alpha-2,8-sialyltransferase 6	6.63E-05	0.317533
7923976	NR_029518	MIR29B2	NR_029518 // MIR29B2 // microRNA 29b-2 // 1q32.2 // 407025	0.00215938	0.246685
8118613	NM_006979	SLC39A7	NM_006979 // SLC39A7 // solute carrier family 39 (zinc transporter), member 7 // 6p21.3	0.00184421	-0.263857
8178225	NM_006979	SLC39A7	NM_006979 // SLC39A7 // solute carrier family 39 (zinc transporter), member 7 // 6p21.3	0.00184421	-0.263857
8179525	NM_006979	SLC39A7	NM_006979 // SLC39A7 // solute carrier family 39 (zinc transporter), member 7 // 6p21.3	0.00184421	-0.263857
8098177	NM_007246	KLHL2	NM_007246 // KLHL2 // kelch-like 2, Mayven (Drosophila) // 4q21.2 // 11275 /// NM_00116	0.00213785	-0.263379
8062603	NM_003286	TOP1	NM_003286 // TOP1 // topoisomerase (DNA) I // 20q12-q13.1 // 7150 /// ENST00000361337 /	0.00144162	-0.259642

7937404	NM_173573	C11orf35	NM_173573 // C11orf35 // chromosome 11 open reading frame 35 // 11p15.5 // 256329 /// E	0.00156481	0.255588
8115907	NM_016391	NOP16	NM_016391 // NOP16 // NOP16 nucleolar protein homolog (yeast) // 5q35.2 // 51491 /// EN	0.000349225	-0.295595
7995783	NM_005953	MT2A	NM_005953 // MT2A // metallothionein 2A // 16q13 // 4502 /// ENST00000245185 // MT2A //	0.00155062	-0.266942
8108981	NM_001112724	STK32A	NM_001112724 // STK32A // serine/threonine kinase 32A // 5q32 // 202374 /// NM_145001 /	0.00277387	-0.252607
8108905	NM_020768	KCTD16	NM_020768 // KCTD16 // potassium channel tetramerisation domain containing 16 // 5q31.3	0.00118213	0.267139
7952145	NM_006389	HYOU1	NM_006389 // HYOU1 // hypoxia up-regulated 1 // 11q23.1-q23.3 // 10525 /// NM_001130991	0.000235462	-0.295002
8136727	BC030533	TRBC1	BC030533 // TRBC1 // T cell receptor beta constant 1 // 7q34 // 28639 /// AK093303 // T	0.000153611	0.317431
8016980	NR_029683	MIR142	NR_029683 // MIR142 // microRNA 142 // 17q22 // 406934	0.00128103	0.262865
7956009	NM_152637	METTL7B	NM_152637 // METTL7B // methyltransferase like 7B // 12q13.2 // 196410 /// ENST00000394	0.00225486	-0.262221
7982377	NM_013372	GREM1	NM_013372 // GREM1 // gremlin 1 // 15q13.3 // 26585 /// NM_001191323 // GREM1 // gremli	5.07E-05	-0.322739
8060963	NM_003081	SNAP25	NM_003081 // SNAP25 // synaptosomal-associated protein, 25kDa // 20p12-p11.2 // 6616 //	4.97E-07	-0.393078

7980861	NM_024764	CATSPERB	NM_024764 // CATSPERB // cation channel, sperm-associated, beta // 14q32.12 // 79820 //	0.000245622	-0.301314
7922523	---		---	0.00280684	-0.252475
8041206	NM_030915	LBH	NM_030915 // LBH // limb bud and heart development homolog (mouse) // 2p23.1 // 81606 /	9.08E-06	0.36049
7997642	NM_031476	CRISPLD2	NM_031476 // CRISPLD2 // cysteine-rich secretory protein LCCL domain containing 2 // 16	7.48E-05	-0.315588
8100603	NM_001812	CENPC1	NM_001812 // CENPC1 // centromere protein C 1 // 4q13.2 // 1060 /// ENST00000273853 //	0.00249368	0.256683
8133876	NM_001001548	CD36	NM_001001548 // CD36 // CD36 molecule (thrombospondin receptor) // 7q11.2 // 948 /// NM	0.000513116	0.277225
7917322	NM_032184	SYDE2	NM_032184 // SYDE2 // synapse defective 1, Rho GTPase, homolog 2 (C. elegans) // 1p22.3	0.00135223	0.255228
7951036	NR_003033	SNORD5	NR_003033 // SNORD5 // small nucleolar RNA, C/D box 5 // 11q21 // 692072 /// AK128061 /	0.00133366	-0.267448
7915787	NM_003629	PIK3R3	NM_003629 // PIK3R3 // phosphoinositide-3-kinase, regulatory subunit 3 (gamma) // 1p34.	1.30E-07	-0.427223
8097480	NM_057175	NAA15	NM_057175 // NAA15 // N(alpha)-acetyltransferase 15, NatA auxiliary subunit // 4q31.1 /	0.00182159	-0.257085
8086344	NM_001337	CX3CR1	NM_001337 // CX3CR1 // chemokine (C-X3-C motif) receptor 1 // 3p21|3p21.3 // 1524 /// N	0.000353892	0.292719
8117128	NM_001949	E2F3	NM_001949 // E2F3 // E2F transcription factor 3 // 6p22 // 1871 /// ENST00000346618 //	1.70E-06	-0.389439

7965979	NM_001034173	ALDH1L2	NM_001034173 // ALDH1L2 // aldehyde dehydrogenase 1 family, member L2 // 12q23.3 // 160	0.00193123	-0.254434
8137863	---		---	0.000316691	0.300977
8091283	NM_182943	PLOD2	NM_182943 // PLOD2 // procollagen-lysine, 2-oxoglutarate 5-dioxygenase 2 // 3q24 // 535	0.00132564	-0.264484
8157216	NM_003358	UGCG	NM_003358 // UGCG // UDP-glucose ceramide glucosyltransferase // 9q31 // 7357 /// ENST0	3.06E-07	-0.39387
8155734	NM_004816	FAM189A2	NM_004816 // FAM189A2 // family with sequence similarity 189, member A2 // 9q21.11 // 9	0.000116536	0.312584
7905329	NM_006818	MLLT11	NM_006818 // MLLT11 // myeloid/lymphoid or mixed-lineage leukemia (trithorax homolog, D	0.00290112	-0.239769
7953385	NM_002046	GAPDH	NM_002046 // GAPDH // glyceraldehyde-3-phosphate dehydrogenase // 12p13 // 2597 /// ENS	0.0028116	-0.246684
8040080	NM_080657	RSAD2	NM_080657 // RSAD2 // radical S-adenosyl methionine domain containing 2 // 2p25.2 // 91	0.00235606	0.253257
8168408	---		---	7.40E-05	0.331238
8096079	---		---	0.00267918	0.249979
8063382	NM_005985	SNAI1	NM_005985 // SNAI1 // snail homolog 1 (Drosophila) // 20q13.2 // 6615 /// ENST000002440	0.00045466	-0.296034
8149733	NM_003842	TNFRSF10B	NM_003842 // TNFRSF10B // tumor necrosis factor receptor superfamily, member 10b // 8p2	1.95E-05	-0.351944
8031311	NM_013289	KIR3DL1	NM_013289 // KIR3DL1 // killer cell immunoglobulin-like receptor, three domains, long c	0.000101133	0.318284

7939158	NM_001076786	QSER1	NM_001076786 // QSER1 // glutamine and serine rich 1 // 11p13 // 79832 /// ENST00000399	0.0025618	-0.257556
8103563	NM_017631	DDX60	NM_017631 // DDX60 // DEAD (Asp-Glu-Ala-Asp) box polypeptide 60 // 4q32.3 // 55601 ///	0.00236091	0.251816
8105040	NM_003999	OSMR	NM_003999 // OSMR // oncostatin M receptor // 5p13.1 // 9180 /// NM_001168355 // OSMR /	2.53E-07	-0.40053
8152828	NM_031415	GSDMC	NM_031415 // GSDMC // gasdermin C // 8q24.21 // 56169 /// ENST00000276708 // GSDMC // g	0.00103695	-0.270547
7954293	NM_000921	PDE3A	NM_000921 // PDE3A // phosphodiesterase 3A, cGMP-inhibited // 12p12 // 5139 /// ENST000	0.000905424	-0.267705
7906475	NM_001004310	FCRL6	NM_001004310 // FCRL6 // Fc receptor-like 6 // 1q23.2 // 343413 /// ENST00000368106 //	0.000619484	0.283408
8128934	---		---	0.00213382	-0.256248
8095362	NM_005953	MT2A	NM_005953 // MT2A // metallothionein 2A // 16q13 // 4502 /// ENST00000245185 // MT2A //	0.00165211	-0.266457
8056363	NM_173512	SLC38A11	NM_173512 // SLC38A11 // solute carrier family 38, member 11 // 2q24.3 // 151258 /// EN	5.77E-05	-0.328635
8117458	NM_001145009	BTN3A1	NM_001145009 // BTN3A1 // butyrophilin, subfamily 3, member A1 // 6p22.1 // 11119 /// N	8.64E-05	0.319473
8106660	NM_006909	RASGRF2	NM_006909 // RASGRF2 // Ras protein-specific guanine nucleotide-releasing factor 2 // 5	0.00255866	-0.259042
8101874	NM_000667	ADH1A	NM_000667 // ADH1A // alcohol dehydrogenase 1A (class I), alpha polypeptide // 4q23 //	0.000141672	0.318197

8109383	NM_000827	GRIA1	NM_000827 // GRIA1 // glutamate receptor, ionotropic, AMPA 1 // 5q33|5q31.1 // 2890 ///	2.24E-07	0.405
8094789	NM_014988	LIMCH1	NM_014988 // LIMCH1 // LIM and calponin homology domains 1 // 4p13 // 22998 /// NM_0011	0.000920984	0.274928
8096335	NM_017912	HERC6	NM_017912 // HERC6 // hect domain and RLD 6 // 4q22.1 // 55008 /// NM_001165136 // HERC	0.00216604	0.259628
8072413	NM_134269	SMTN	NM_134269 // SMTN // smoothelin // 22q12.2 // 6525 /// NM_134270 // SMTN // smoothelin	0.0015507	-0.263736
7954985	NM_032256	TMEM117	NM_032256 // TMEM117 // transmembrane protein 117 // 12q12 // 84216 /// ENST00000266534	0.00102755	-0.268724
7975268	NM_001172	ARG2	NM_001172 // ARG2 // arginase, type II // 14q24.1 // 384 /// NM_006370 // VTI1B // vesi	0.000868047	-0.280589
7914648	NM_198040	PHC2	NM_198040 // PHC2 // polyhomeotic homolog 2 (Drosophila) // 1p34.3 // 1912 /// NM_00442	0.000361404	-0.306988
8018095	---		---	0.000718414	-0.287372
7921690	NM_017625	ITLN1	NM_017625 // ITLN1 // intelectin 1 (galactofuranose binding) // 1q21.3 // 55600 /// ENS	0.00204236	0.259003
7976560	NM_000623	BDKRB2	NM_000623 // BDKRB2 // bradykinin receptor B2 // 14q32.1-q32.2 // 624 /// ENST000003060	0.00106457	-0.266688
8029465	NM_005178	BCL3	NM_005178 // BCL3 // B-cell CLL/lymphoma 3 // 19q13.1-q13.2 // 602 /// BC064993 // BCL3	0.000397591	-0.29686
8177046	---		---	0.00126499	-0.279032

8083594	NM_002852	PTX3	NM_002852 // PTX3 // pentraxin 3, long // 3q25 // 5806 /// ENST00000295927 // PTX3 // p	9.90E-07	-0.402639
8168749	NM_014467	SRPX2	NM_014467 // SRPX2 // sushi-repeat-containing protein, X-linked 2 // Xq21.33-q23 // 272	5.44E-08	-0.42953
8166948	---		---	0.00168582	0.264742
8042416	NM_014882	ARHGAP25	NM_014882 // ARHGAP25 // Rho GTPase activating protein 25 // 2p13.3 // 9938 /// NM_0011	0.00148559	0.265929
7933084	NM_005746	NAMPT	NM_005746 // NAMPT // nicotinamide phosphoribosyltransferase // 7q22.3 // 10135 /// ENS	1.13E-05	-0.362981
8123644	NM_001069	TUBB2A	NM_001069 // TUBB2A // tubulin, beta 2A // 6p25 // 7280 /// ENST00000333628 // TUBB2A /	4.78E-05	-0.344594
8152946	NM_004519	KCNQ3	NM_004519 // KCNQ3 // potassium voltage-gated channel, KQT-like subfamily, member 3 //	2.63E-06	-0.389882
7979548	NM_145171	GPHB5	NM_145171 // GPHB5 // glycoprotein hormone beta 5 // 14q23.2 // 122876 /// ENST00000314	0.00115044	0.27657
8146645	NM_152414	BHLHE22	NM_152414 // BHLHE22 // basic helix-loop-helix family, member e22 // 8q13 // 27319 ///	0.00237588	-0.243161
7943218	NM_015368	PANX1	NM_015368 // PANX1 // pannexin 1 // 11q21 // 24145 /// ENST00000227638 // PANX1 // pann	3.73E-05	-0.342671
8133155	NM_003596	TPST1	NM_003596 // TPST1 // tyrosylprotein sulfotransferase 1 // 7q11.21 // 8460 /// ENST0000	8.44E-06	-0.352026
8095376	NM_005953	MT2A	NM_005953 // MT2A // metallothionein 2A // 16q13 // 4502 /// ENST00000245185 // MT2A //	0.0010068	-0.279622

7953981	NM_001987	ETV6	NM_001987 // ETV6 // ets variant 6 // 12p13 // 2120 /// ENST00000266427 // ETV6 // ets	1.31E-05	-0.35611
8041149	NM_015131	WDR43	NM_015131 // WDR43 // WD repeat domain 43 // 2p23.2 // 23160 /// ENST00000407426 // WDR	5.13E-06	-0.365523
8043995	NM_000877	IL1R1	NM_000877 // IL1R1 // interleukin 1 receptor, type I // 2q12 // 3554 /// ENST0000023394	4.97E-07	-0.397848
7926934	---		---	0.00231136	0.256493
8085360	NM_003256	TIMP4	NM_003256 // TIMP4 // TIMP metallopeptidase inhibitor 4 // 3p25 // 7079 /// ENST0000028	0.000492824	-0.287743
8099897	NM_003359	UGDH	NM_003359 // UGDH // UDP-glucose 6-dehydrogenase // 4p15.1 // 7358 /// NM_001184700 //	0.00235877	-0.249982
7952056	NM_000732	CD3D	NM_000732 // CD3D // CD3d molecule, delta (CD3-TCR complex) // 11q23 // 915 /// NM_0010	0.000643061	0.285197
8156569	NR_029664	MIR23B	NR_029664 // MIR23B // microRNA 23b // 9q22.32 // 407011 /// AF043897 // C9orf3 // chro	2.90E-05	0.341154
8105302	NM_006350	FST	NM_006350 // FST // follistatin // 5q11.2 // 10468 /// NM_013409 // FST // follistatin	0.000132661	-0.30867
7921821	NM_005099	ADAMTS4	NM_005099 // ADAMTS4 // ADAM metallopeptidase with thrombospondin type 1 motif, 4 // 1q	2.43E-07	-0.418749
8069676	NM_006988	ADAMTS1	NM_006988 // ADAMTS1 // ADAM metallopeptidase with thrombospondin type 1 motif, 1 // 21	0.000753164	-0.281984
8059279	NM_004438	EPHA4	NM_004438 // EPHA4 // EPH receptor A4 // 2q36.1 // 2043 /// ENST00000281821 // EPHA4 //	0.00115882	-0.271904
8142136	NM_175884	FLJ36031	NM_175884 // FLJ36031 // hypothetical protein FLJ36031 // 7q22.3 // 168455	0.00124638	-0.265959

8179228	NM_007109	TCF19	NM_007109 // TCF19 // transcription factor 19 // 6p21.3 // 6941 /// NM_001077511 // TCF	0.00239102	0.264762
7944185	NM_000073	CD3G	NM_000073 // CD3G // CD3g molecule, gamma (CD3-TCR complex) // 11q23 // 917 /// ENST000	0.000313732	0.30768
7978718	NM_006364	SEC23A	NM_006364 // SEC23A // Sec23 homolog A (S. cerevisiae) // 14q21.1 // 10484 /// ENST0000	0.000880445	-0.274294
7899753	NM_005356	LCK	NM_005356 // LCK // lymphocyte-specific protein tyrosine kinase // 1p34.3 // 3932 /// N	0.00020395	0.307877
7956826	NM_015279	TBC1D30	NM_015279 // TBC1D30 // TBC1 domain family, member 30 // 12q14.3 // 23329 /// AB449914	1.64E-05	-0.36009
7952046	NM_144765	MPZL2	NM_144765 // MPZL2 // myelin protein zero-like 2 // 11q24 // 10205 /// NM_005797 // MPZ	0.000537763	-0.283695
8001531	NM_005950	MT1G	NM_005950 // MT1G // metallothionein 1G // 16q13 // 4495 /// BC020757 // MT1G // metall	0.000716997	-0.28914
8017850	NM_017983	WIPI1	NM_017983 // WIPI1 // WD repeat domain, phosphoinositide interacting 1 // 17q24.2 // 55	2.48E-06	-0.382853
8177669	NR_034021	SMA5	NR_034021 // SMA5 // glucuronidase, beta pseudogene // 5q13 // 11042 /// NR_027386 // G	0.00287095	0.246024
7921076	NM_182679	GPATCH4	NM_182679 // GPATCH4 // G patch domain containing 4 // 1q22 // 54865 /// NM_015590 // G	0.000633602	-0.285451
8103812	NM_080874	ASB5	NM_080874 // ASB5 // ankyrin repeat and SOCS box-containing 5 // 4q34.2 // 140458 /// E	0.00132536	-0.268219

8143781	NM_024711	GIMAP6	NM_024711 // GIMAP6 // GTPase, IMAP family member 6 // --- // 474344 /// NR_024115 // G	0.000396826	0.289367
7907430	NM_000639	FASLG	NM_000639 // FASLG // Fas ligand (TNF superfamily, member 6) // 1q23 // 356 /// ENST000	0.000375431	0.293977
8004184	NM_017523	XAF1	NM_017523 // XAF1 // XIAP associated factor 1 // 17p13.1 // 54739 /// NM_199139 // XAF1	0.000249128	0.303314
8175393	NM_004840	ARHGEF6	NM_004840 // ARHGEF6 // Rac/Cdc42 guanine nucleotide exchange factor (GEF) 6 // Xq26.3	5.96E-05	0.323093
7922229	NM_000450	SELE	NM_000450 // SELE // selectin E // 1q22-q25 // 6401 /// ENST00000333360 // SELE // sele	7.76E-08	-0.422184
7943998	NM_006169	NNMT	NM_006169 // NNMT // nicotinamide N-methyltransferase // 11q23.1 // 4837 /// ENST000002	4.11E-05	-0.334099
7928291	NM_004273	CHST3	NM_004273 // CHST3 // carbohydrate (chondroitin 6) sulfotransferase 3 // 10q22.1 // 946	2.57E-05	-0.349158
8094609	NM_138389	FAM114A1	NM_138389 // FAM114A1 // family with sequence similarity 114, member A1 // 4p14 // 9268	0.000159609	-0.311561
8048717	NM_152386	SGPP2	NM_152386 // SGPP2 // sphingosine-1-phosphate phosphatase 2 // 2q36.1 // 130367 /// ENS	0.00138951	-0.261331
8075886	NM_000878	IL2RB	NM_000878 // IL2RB // interleukin 2 receptor, beta // 22q13|22q13.1 // 3560 /// ENST000	7.44E-05	0.324519
7953749	NM_080387	CLEC4D	NM_080387 // CLEC4D // C-type lectin domain family 4, member D // 12p13.31 // 338339 //	0.00249631	-0.255105

7906863	NM_003115	UAP1	NM_003115 // UAP1 // UDP-N-acteylglucosamine pyrophosphorylase 1 // 1q23.3 // 6675 ///	4.89E-05	-0.324802
8157092	NM_018112	TMEM38B	NM_018112 // TMEM38B // transmembrane protein 38B // 9q31.2 // 55151 /// ENST0000037469	0.000904909	-0.284526
7920082	NM_005060	RORC	NM_005060 // RORC // RAR-related orphan receptor C // 1q21 // 6097 /// NM_001001523 //	1.86E-06	0.383197
7910611	NM_002245	KCNK1	NM_002245 // KCNK1 // potassium channel, subfamily K, member 1 // 1q42-q43 // 3775 ///	0.000815208	-0.276805
8145122	NM_001128431	SLC39A14	NM_001128431 // SLC39A14 // solute carrier family 39 (zinc transporter), member 14 // 8	1.23E-06	-0.38835
8173755	NM_004867	ITM2A	NM_004867 // ITM2A // integral membrane protein 2A // Xq13.3-Xq21.2 // 9452 /// NM_0011	7.48E-05	0.315915
8138857	NM_024051	GGCT	NM_024051 // GGCT // gamma-glutamylcyclotransferase // 7p15-p14 // 79017 /// ENST000002	0.000761806	-0.27659
8171723	---		---	0.000452306	0.286697
8091537	NM_178822	IGSF10	NM_178822 // IGSF10 // immunoglobulin superfamily, member 10 // 3q25.1 // 285313 /// NM	9.20E-05	0.331188
8053668	NM_004836	EIF2AK3	NM_004836 // EIF2AK3 // eukaryotic translation initiation factor 2-alpha kinase 3 // 2p	0.000888405	-0.276923
8171449	NM_021804	ACE2	NM_021804 // ACE2 // angiotensin I converting enzyme (peptidyl-dipeptidase A) 2 // Xp22	0.000192322	-0.317449
8103535	NR_026575	GK3P	NR_026575 // GK3P // glycerol kinase 3 pseudogene // 4q32.1 // 2713 /// AK292282 // GK3	0.000188394	-0.305804

8027002	NM_004864	GDF15	NM_004864 // GDF15 // growth differentiation factor 15 // 19p13.11 // 9518 /// ENST0000	0.000237379	-0.301861
8109001	NM_001127698	SPINK5	NM_001127698 // SPINK5 // serine peptidase inhibitor, Kazal type 5 // 5q32 // 11005 ///	0.00252222	-0.247948
8001455	---		---	0.00103864	0.275366
8051187	NM_022823	FNDC4	NM_022823 // FNDC4 // fibronectin type III domain containing 4 // 2p23.3 // 64838 /// E	0.000995797	-0.277563
8161423	NR_003674	KGFLP1	NR_003674 // KGFLP1 // keratinocyte growth factor-like protein 1 // 9p11.2 // 387628 //	0.00203767	-0.260683
8161455	NR_003674	KGFLP1	NR_003674 // KGFLP1 // keratinocyte growth factor-like protein 1 // 9p11.2 // 387628 //	0.00203767	-0.260683
8155487	NR_003674	KGFLP1	NR_003674 // KGFLP1 // keratinocyte growth factor-like protein 1 // 9p11.2 // 387628 //	0.00203767	-0.260683
7974870	NM_003082	SNAPC1	NM_003082 // SNAPC1 // small nuclear RNA activating complex, polypeptide 1, 43kDa // 14	0.00187072	-0.260226
7995834	NR_003669	MT1IP	NR_003669 // MT1IP // metallothionein 1I (pseudogene) // 16q13 // 644314 /// AF348997 /	0.000925946	-0.282063
7979179	NM_014584	ERO1L	NM_014584 // ERO1L // ERO1-like (S. cerevisiae) // 14q22.1 // 30001 /// ENST00000395686	0.000155207	-0.312642
8069541	NM_022136	SAMSN1	NM_022136 // SAMSN1 // SAM domain, SH3 domain and nuclear localization signals 1 // 21q	0.00162596	-0.259588
8176624	NM_001122665	DDX3Y	NM_001122665 // DDX3Y // DEAD (Asp-Glu-Ala-Asp) box polypeptide 3, Y-linked // Yq11 //	0.00261332	-0.250567

8011245	NM_018128	TSR1	NM_018128 // TSR1 // TSR1, 20S rRNA accumulation, homolog (S. cerevisiae) // 17p13.3 //	0.000437635	-0.292823
7961230	NM_003651	CSDA	NM_003651 // CSDA // cold shock domain protein A // 12p13.1 // 8531 /// NM_001145426 //	0.000488401	-0.292765
8148435	NM_003882	WISP1	NM_003882 // WISP1 // WNT1 inducible signaling pathway protein 1 // 8q24.22 // 8840 ///	8.40E-05	-0.316977
8144786	NM_003046	SLC7A2	NM_003046 // SLC7A2 // solute carrier family 7 (cationic amino acid transporter, y+ sys	0.000316506	-0.298578
7964660	NM_000706	AVPR1A	NM_000706 // AVPR1A // arginine vasopressin receptor 1A // 12q14-q15 // 552 /// ENST000	0.00282341	-0.248236
8097449	NM_032961	PCDH10	NM_032961 // PCDH10 // protocadherin 10 // 4q28.3 // 57575 /// NM_020815 // PCDH10 // p	0.000372542	0.300853
8152280	NM_013437	LRP12	NM_013437 // LRP12 // low density lipoprotein receptor-related protein 12 // 8q22.2 //	0.000592741	-0.286688
8066247	NR_027241	LOC388796	NR_027241 // LOC388796 // hypothetical LOC388796 // 20q11.23 // 388796 /// NR_015366 //	0.0001524	-0.319484
7948058	NM_001193471	FOLH1	NM_001193471 // FOLH1 // folate hydrolase (prostate-specific membrane antigen) 1 // 11p	0.000687812	0.286995
8112564	NR_027386	GUSBP3	NR_027386 // GUSBP3 // glucuronidase, beta pseudogene 3 // 5q13.2 // 653188 /// NR_0340	0.00282326	0.245883
8152703	NM_058229	FBXO32	NM_058229 // FBXO32 // F-box protein 32 // 8q24.13 // 114907 /// NM_148177 // FBXO32 //	3.47E-05	-0.333871

8096635	NM_003998	NFKB1	NM_003998 // NFKB1 // nuclear factor of kappa light polypeptide gene enhancer in B-cell	0.00138359	-0.266039
7939056	NM_003986	BBOX1	NM_003986 // BBOX1 // butyrobetaine (gamma), 2-oxoglutarate dioxygenase (gamma-butyrobe	0.00188879	-0.259643
8042144	NM_002908	REL	NM_002908 // REL // v-rel reticuloendotheliosis viral oncogene homolog (avian) // 2p13-	0.00120934	-0.274564
8095680	NM_000584	IL8	NM_000584 // IL8 // interleukin 8 // 4q13-q21 // 3576 /// ENST00000307407 // IL8 // int	1.15E-05	-0.353577
8156571	NR_029665	MIR27B	NR_029665 // MIR27B // microRNA 27b // 9q22.32 // 407019 /// AF043897 // C9orf3 // chro	0.00186457	0.257139
8137252	NM_130759	GIMAP1	NM_130759 // GIMAP1 // GTPase, IMAP family member 1 // 7q36.1 // 170575 /// ENST0000030	0.000163224	0.311628
8113120	BC028919	TOB2	BC028919 // TOB2 // transducer of ERBB2, 2 // 22q13.2 // 10766	0.00259485	0.252066
8165707	BC028919	TOB2	BC028919 // TOB2 // transducer of ERBB2, 2 // 22q13.2 // 10766	0.00259485	0.252066
8107897	NM_003687	PDLIM4	NM_003687 // PDLIM4 // PDZ and LIM domain 4 // 5q31.1 // 8572 /// NM_001131027 // PDLIM	0.00201103	-0.257082
8005957	NR_000009	SNORD4B	NR_000009 // SNORD4B // small nucleolar RNA, C/D box 4B // 17q11 // 26772	0.00178435	-0.264376
8121949	NM_000426	LAMA2	NM_000426 // LAMA2 // laminin, alpha 2 // 6q22-q23 // 3908 /// NM_001079823 // LAMA2 //	0.000714514	-0.274988
8006123	NM_001304	CPD	NM_001304 // CPD // carboxypeptidase D // 17q11.2 // 1362 /// ENST00000225719 // CPD //	1.33E-05	-0.359144

8137485	NM_001039350	DPP6	NM_001039350 // DPP6 // dipeptidyl-peptidase 6 // 7q36.2 // 1804 /// NM_001936 // DPP6	7.31E-05	0.315948
8061094	NM_002594	PCSK2	NM_002594 // PCSK2 // proprotein convertase subtilisin/kexin type 2 // 20p11.2 // 5126	0.00210631	-0.256427
8159992	NM_024896	ERMP1	NM_024896 // ERMP1 // endoplasmic reticulum metallopeptidase 1 // 9p24 // 79956 /// ENS	0.00262155	0.245568
8126853	NM_001013732	C6orf138	NM_001013732 // C6orf138 // chromosome 6 open reading frame 138 // 6p12.3 // 442213 ///	0.00291361	-0.246512
8174134	NM_001006938	TCEAL6	NM_001006938 // TCEAL6 // transcription elongation factor A (SII)-like 6 // Xq22.1 // 1	0.00173432	-0.25883
8133209	NR_003666	SPDYE7P	NR_003666 // SPDYE7P // speedy homolog E7 (Xenopus laevis), pseudogene // 7q11.23 // 44	0.00278055	0.246485
7899436	NM_031459	SESN2	NM_031459 // SESN2 // sestrin 2 // 1p35.3 // 83667 /// ENST00000253063 // SESN2 // sest	0.000624986	-0.285137
8129482	NM_001017373	SAMD3	NM_001017373 // SAMD3 // sterile alpha motif domain containing 3 // 6q23.1 // 154075 //	0.000209877	0.311522
7929065	NM_001548	IFIT1	NM_001548 // IFIT1 // interferon-induced protein with tetratricopeptide repeats 1 // 10	4.85E-05	0.334914
8007537	NM_145273	CD300LG	NM_145273 // CD300LG // CD300 molecule-like family member g // 17q21.31 // 146894 /// N	0.000943928	0.267138
8104788	NM_001145525	RAI14	NM_001145525 // RAI14 // retinoic acid induced 14 // 5p13.3-p13.2 // 26064 /// NM_00114	0.000528517	-0.289072

8019924	NM_006471	MYL12A	NM_006471 // MYL12A // myosin, light chain 12A, regulatory, non-sarcomeric // 18p11.31	0.00295252	0.249662
7947270	NM_002233	KCNA4	NM_002233 // KCNA4 // potassium voltage-gated channel, shaker-related subfamily, member	0.000165063	0.313274
8043861	NM_015904	EIF5B	NM_015904 // EIF5B // eukaryotic translation initiation factor 5B // 2q11.2 // 9669 ///	0.00131358	-0.267277
8051030	NM_021095	SLC5A6	NM_021095 // SLC5A6 // solute carrier family 5 (sodium-dependent vitamin transporter),	0.00108058	-0.27894
8095697	NM_001511	CXCL1	NM_001511 // CXCL1 // chemokine (C-X-C motif) ligand 1 (melanoma growth stimulating act	0.000132134	-0.312412
7960575	NM_001033714	NOP2	NM_001033714 // NOP2 // NOP2 nucleolar protein homolog (yeast) // 12p13 // 4839 /// NM_	9.39E-05	-0.32438
8003298	NM_003486	SLC7A5	NM_003486 // SLC7A5 // solute carrier family 7 (cationic amino acid transporter, y+ sys	3.24E-05	-0.34458
8069208	NM_030582	COL18A1	NM_030582 // COL18A1 // collagen, type XVIII, alpha 1 // 21q22.3 // 80781 /// NM_130444	0.00284907	-0.246798
8127502	NR_026807	C6orf155	NR_026807 // C6orf155 // chromosome 6 open reading frame 155 // 6q13 // 79940	0.00239037	0.256592
8110450	NM_031266	HNRNPAB	NM_031266 // HNRNPAB // heterogeneous nuclear ribonucleoprotein A/B // 5q35.3 // 3182 /	0.000744025	-0.289773
8156897	BC008993	C9orf30	BC008993 // C9orf30 // chromosome 9 open reading frame 30 // 9q31.1 // 91283 /// AY5983	0.000143646	-0.321588

7965918	NM_001031701	NT5DC3	NM_001031701 // NT5DC3 // 5'-nucleotidase domain containing 3 // 12q22-q23.1 // 51559 /	0.000528961	-0.288599
7938563	NM_001178	ARNTL	NM_001178 // ARNTL // aryl hydrocarbon receptor nuclear translocator-like // 11p15 // 4	0.000858898	-0.289962
8113234	NM_000439	PCSK1	NM_000439 // PCSK1 // proprotein convertase subtilisin/kexin type 1 // 5q15-q21 // 5122	0.00139697	-0.27091
8042381	NM_020143	PNO1	NM_020143 // PNO1 // partner of NOB1 homolog (S. cerevisiae) // 2p14 // 56902 /// ENST0	0.00186923	-0.267173
8150862	---		---	0.000378295	-0.299674
7939376	NM_174902	LDLRAD3	NM_174902 // LDLRAD3 // low density lipoprotein receptor class A domain containing 3 //	6.59E-05	-0.334598
7994541	NM_014387	LAT	NM_014387 // LAT // linker for activation of T cells // 16p11.2 // 27040 /// NM_0010149	0.00208883	0.260606
7971104	NM_016179	TRPC4	NM_016179 // TRPC4 // transient receptor potential cation channel, subfamily C, member	4.04E-07	-0.407372
8142120	NM_005746	NAMPT	NM_005746 // NAMPT // nicotinamide phosphoribosyltransferase // 7q22.3 // 10135 /// ENS	5.65E-06	-0.372399
8166632	NM_001128127	GK	NM_001128127 // GK // glycerol kinase // Xp21.3 // 2710 /// NM_203391 // GK // glycerol	0.00100192	-0.274093
8162531	---		---	0.0011222	-0.273545
7899615	NM_178865	SERINC2	NM_178865 // SERINC2 // serine incorporator 2 // 1p35.1 // 347735 /// ENST00000373709 /	0.000159209	-0.312582
8059376	NM_006216	SERPINE2	NM_006216 // SERPINE2 // serpin peptidase inhibitor, clade E (nexin, plasminogen activa	0.00123744	-0.270362

8000028	NM_173475	DCUN1D3	NM_173475 // DCUN1D3 // DCN1, defective in cullin neddylation 1, domain containing 3 (S	6.60E-05	-0.325255
8043993	AY358263	LOC100131131	AY358263 // LOC100131131 // AHPA9419 // 2q12.1 // 100131131	0.00202151	-0.258495
7996891	NM_032830	CIRH1A	NM_032830 // CIRH1A // cirrhosis, autosomal recessive 1A (cirhin) // 16q22.1 // 84916 /	0.000132804	-0.313421
7979824	NM_001130004	ACTN1	NM_001130004 // ACTN1 // actinin, alpha 1 // 14q24.1-q24.2|14q24|14q22-q24 // 87 /// NM	1.79E-05	-0.35065
8147206	NM_003821	RIPK2	NM_003821 // RIPK2 // receptor-interacting serine-threonine kinase 2 // 8q21 // 8767 //	0.00196448	-0.258264
7976567	NM_000710	BDKRB1	NM_000710 // BDKRB1 // bradykinin receptor B1 // 14q32.1-q32.2 // 623 /// ENST000002166	0.00261417	-0.253882
7904287	NM_001767	CD2	NM_001767 // CD2 // CD2 molecule // 1p13.1 // 914 /// ENST00000369478 // CD2 // CD2 mol	0.000520055	0.295811
8095728	NM_001432	EREG	NM_001432 // EREG // epiregulin // 4q13.3 // 2069 /// ENST00000244869 // EREG // epireg	0.00132346	-0.265822
8069553	NM_003489	NRIP1	NM_003489 // NRIP1 // nuclear receptor interacting protein 1 // 21q11.2 // 8204 /// ENS	0.000498408	-0.292267
8080980	NM_018029	EBLN2	NM_018029 // EBLN2 // endogenous Borna-like N element-2 // 3p13 // 55096 /// AY036895 /	0.00108346	-0.278759
8007931	NM_000212	ITGB3	NM_000212 // ITGB3 // integrin, beta 3 (platelet glycoprotein IIIa, antigen CD61) // 17	3.28E-08	-0.429066
7962375	NM_153026	PRICKLE1	NM_153026 // PRICKLE1 // prickle homolog 1 (Drosophila) // 12q12 // 144165 /// NM_00114	0.000388891	0.296152

8149749	NM_003840	TNFRSF10D	NM_003840 // TNFRSF10D // tumor necrosis factor receptor superfamily, member 10d, decoy	0.00280852	-0.25604
8112312	NM_014473	DIMT1L	NM_014473 // DIMT1L // DIM1 dimethyladenosine transferase 1-like (S. cerevisiae) // 5q1	0.000845073	-0.281203
8008588	NM_002126	HLF	NM_002126 // HLF // hepatic leukemia factor // 17q22 // 3131 /// ENST00000226067 // HLF	0.00286825	0.252064
7897449	NM_025106	SPSB1	NM_025106 // SPSB1 // splA/ryanodine receptor domain and SOCS box containing 1 // 1p36.	6.23E-07	-0.384404
8092849	NM_024524	ATP13A3	NM_024524 // ATP13A3 // ATPase type 13A3 // 3q29 // 79572 /// ENST00000256031 // ATP13A	2.12E-07	-0.406284
8044574	NM_173842	IL1RN	NM_173842 // IL1RN // interleukin 1 receptor antagonist // 2q14.2 // 3557 /// NM_173841	0.000271028	-0.294298
7968351	NM_032849	C13orf33	NM_032849 // C13orf33 // chromosome 13 open reading frame 33 // 13q12.3 // 84935 /// EN	5.81E-05	-0.327075
8084794	NM_002182	IL1RAP	NM_002182 // IL1RAP // interleukin 1 receptor accessory protein // 3q28 // 3556 /// NM_	0.00189581	-0.261382
8033054	NM_000149	FUT3	NM_000149 // FUT3 // fucosyltransferase 3 (galactoside 3(4)-L-fucosyltransferase, Lewis	0.000183492	-0.320038
8068202	NM_058187	C21orf63	NM_058187 // C21orf63 // chromosome 21 open reading frame 63 // 21q22.11 // 59271 /// E	0.000579951	-0.281069
8092691	NM_001706	BCL6	NM_001706 // BCL6 // B-cell CLL/lymphoma 6 // 3q27 // 604 /// NM_001130845 // BCL6 // B	5.43E-06	-0.362441

7943162	NM_014039	C11orf54	NM_014039 // C11orf54 // chromosome 11 open reading frame 54 // 11q21 // 28970 /// ENST	0.000488696	0.281645
7921916	NM_003617	RGS5	NM_003617 // RGS5 // regulator of G-protein signaling 5 // 1q23.1 // 8490 /// NM_001195	0.00179346	-0.2636
7956759	NM_020762	SRGAP1	NM_020762 // SRGAP1 // SLIT-ROBO Rho GTPase activating protein 1 // 12q14.2 // 57522 //	6.17E-05	-0.329903
7905220	NM_004425	ECM1	NM_004425 // ECM1 // extracellular matrix protein 1 // 1q21 // 1893 /// NM_022664 // EC	0.00274647	-0.251406
7961540	NM_032918	RERG	NM_032918 // RERG // RAS-like, estrogen-regulated, growth inhibitor // 12p12.3 // 85004	0.00250982	0.254948
8082246	---		---	0.00102403	0.272887
8174103	NM_001128127	GK	NM_001128127 // GK // glycerol kinase // Xp21.3 // 2710 /// NM_000167 // GK // glycerol	0.00207865	-0.258823
7911341	---		---	0.00222485	0.258697
7965040	NM_007350	PHLDA1	NM_007350 // PHLDA1 // pleckstrin homology-like domain, family A, member 1 // 12q15 //	1.67E-06	-0.389623
7904429	NR_033781	HSD3BP4	NR_033781 // HSD3BP4 // hydroxy-delta-5-steroid dehydrogenase, 3 beta, pseudogene 4 //	8.49E-06	0.371805
8140463	NM_006682	FGL2	NM_006682 // FGL2 // fibrinogen-like 2 // 7q11.23 // 10875 /// ENST00000248598 // FGL2	0.00130181	0.279326
7987145	NM_001103184	FMN1	NM_001103184 // FMN1 // formin 1 // 15q13.3 // 342184 /// ENST00000334528 // FMN1 // fo	0.00192912	-0.265578

8084064	NM_006636	MTHFD2	NM_006636 // MTHFD2 // methylenetetrahydrofolate dehydrogenase (NADP+ dependent) 2, met	9.70E-05	-0.329524
7958174	NM_003330	TXNRD1	NM_003330 // TXNRD1 // thioredoxin reductase 1 // 12q23-q24.1 // 7296 /// NM_001093771	0.00285085	-0.254859
8142061	NM_019042	PUS7	NM_019042 // PUS7 // pseudouridylate synthase 7 homolog (S. cerevisiae) // 7q22.3 // 54	0.00255099	-0.2563
8068651	NM_006198	PCP4	NM_006198 // PCP4 // Purkinje cell protein 4 // 21q22.2 // 5121 /// ENST00000328619 //	0.00118274	-0.269242
8085081	NM_016302	CRBN	NM_016302 // CRBN // cereblon // 3p26.2 // 51185 /// NM_001173482 // CRBN // cereblon /	0.00227419	0.25702
8138735	NM_019102	HOXA5	NM_019102 // HOXA5 // homeobox A5 // 7p15.2 // 3202 /// ENST00000222726 // HOXA5 // hom	0.00236429	0.259123
7973221	AK301287	TRAJ17	AK301287 // TRAJ17 // T cell receptor alpha joining 17 // 14q11 // 28738 /// BC035680 /	3.21E-05	0.35007
8003611	NM_024792	FAM57A	NM_024792 // FAM57A // family with sequence similarity 57, member A // 17p13.3 // 79850	0.000866056	-0.27564
8043945	NM_145686	MAP4K4	NM_145686 // MAP4K4 // mitogen-activated protein kinase kinase kinase kinase 4 // 2q11.	0.00100983	-0.280393
8088560	NM_182920	ADAMTS9	NM_182920 // ADAMTS9 // ADAM metallopeptidase with thrombospondin type 1 motif, 9 // 3p	4.43E-07	-0.400681
8052149	NM_014614	PSME4	NM_014614 // PSME4 // proteasome (prosome, macropain) activator subunit 4 // 2p16.2 //	9.27E-06	-0.358069
7978132	NM_024658	IPO4	NM_024658 // IPO4 // importin 4 // 14q12 // 79711 /// ENST00000354464 // IPO4 // import	0.000589274	-0.284819

8175217	NM_001448	GPC4	NM_001448 // GPC4 // glypican 4 // Xq26.1 // 2239 /// ENST00000370828 // GPC4 // glypic	0.00214173	-0.249256
8044391	NM_006343	MERTK	NM_006343 // MERTK // c-mer proto-oncogene tyrosine kinase // 2q14.1 // 10461 /// ENST0	6.34E-06	-0.370535
8125887	NM_003214	TEAD3	NM_003214 // TEAD3 // TEA domain family member 3 // 6p21.2 // 7005 /// ENST00000506863	3.72E-05	-0.336946
8072461	NM_016733	LIMK2	NM_016733 // LIMK2 // LIM domain kinase 2 // 22q12.2 // 3985 /// NM_001031801 // LIMK2	0.000634	-0.291617
8140840	NM_024636	STEAP4	NM_024636 // STEAP4 // STEAP family member 4 // 7q21.12 // 79689 /// ENST00000380079 //	0.00028056	-0.291399
7976812	NR_003232	SNORD113-4	NR_003232 // SNORD113-4 // small nucleolar RNA, C/D box 113-4 // 14q32.31 // 767564	0.00213144	-0.256167
7990452	---		---	0.000988182	0.284507
8008517	NM_198175	NME1	NM_198175 // NME1 // non-metastatic cells 1, protein (NM23A) expressed in // 17q21.3 //	0.00120277	-0.268657
8057677	NM_014585	SLC40A1	NM_014585 // SLC40A1 // solute carrier family 40 (iron-regulated transporter), member 1	1.29E-05	0.364065
8038117	NM_001352	DBP	NM_001352 // DBP // D site of albumin promoter (albumin D-box) binding protein // 19q13	5.76E-05	0.34278
8130556	NM_001024465	SOD2	NM_001024465 // SOD2 // superoxide dismutase 2, mitochondrial // 6q25.3 // 6648 /// NM_	0.00218869	-0.266281
7995838	NM_005952	MT1X	NM_005952 // MT1X // metallothionein 1X // 16q13 // 4501 /// ENST00000394485 // MT1X //	0.000277545	-0.302698

8121515	NM_018593	SLC16A10	NM_018593 // SLC16A10 // solute carrier family 16, member 10 (aromatic amino acid trans	7.28E-06	-0.363853
8042830	NR_027405	MTHFD2	NR_027405 // MTHFD2 // methylenetetrahydrofolate dehydrogenase (NADP+ dependent) 2, met	4.75E-05	-0.341671
8111915	NM_005410	SEPP1	NM_005410 // SEPP1 // selenoprotein P, plasma, 1 // 5q31 // 6414 /// NM_001085486 // SE	0.000544434	0.290732
7923974	NR_029832	MIR29C	NR_029832 // MIR29C // microRNA 29c // 1q32.2 // 407026	4.28E-05	0.32385
7921344	NM_012081	ELL2	NM_012081 // ELL2 // elongation factor, RNA polymerase II, 2 // 5q15 // 22936 /// ENST0	1.57E-06	-0.397338
8032839	NM_032108	SEMA6B	NM_032108 // SEMA6B // sema domain, transmembrane domain (TM), and cytoplasmic domain,	0.00139772	-0.276187
7995829	NM_005951	MT1H	NM_005951 // MT1H // metallothionein 1H // 16q13 // 4496 /// ENST00000332374 // MT1H //	0.00137304	-0.27105
8060734	NR_029519	MIR103-2	NR_029519 // MIR103-2 // microRNA 103-2 // 20p13 // 406896 /// NR_031722 // MIR103-2AS	0.00282783	0.250683
8102440	NM_024590	ARSJ	NM_024590 // ARSJ // arylsulfatase family, member J // 4q26 // 79642 /// ENST0000031536	0.000628346	-0.285226
8125775	AY568085	C6orf125	AY568085 // C6orf125 // chromosome 6 open reading frame 125 // 6p21.31 // 84300 /// AF0	4.61E-05	-0.345458
7991234	NM_005928	MFGE8	NM_005928 // MFGE8 // milk fat globule-EGF factor 8 protein // 15q25 // 4240 /// NM_001	0.0014984	-0.270713

8043981	NM_004633	IL1R2	NM_004633 // IL1R2 // interleukin 1 receptor, type II // 2q12 // 7850 /// NM_173343 //	6.95E-06	-0.374177
8082585	NR_022012	COL6A5	NR_022012 // COL6A5 // collagen, type VI, alpha 5 // 3q22.1 // 256076 /// NM_153264 //	0.000981195	0.284938
8026490	NR_015379	UCA1	NR_015379 // UCA1 // urothelial cancer associated 1 (non-protein coding) // 19p13.12 //	0.00165467	0.273827
8126058	NM_016059	PPIL1	NM_016059 // PPIL1 // peptidylprolyl isomerase (cyclophilin)-like 1 // 6p21.1 // 51645	0.000360447	-0.299133
7949719	NM_001040716	PC	NM_001040716 // PC // pyruvate carboxylase // 11q13.4-q13.5 // 5091 /// NM_000920 // PC	0.000452526	-0.29527
8148501	NM_032611	PTP4A3	NM_032611 // PTP4A3 // protein tyrosine phosphatase type IVA, member 3 // 8q24.3 // 111	0.00252039	-0.260468
8094340	NR_029631	MIR218-1	NR_029631 // MIR218-1 // microRNA 218-1 // 4p15.31 // 407000	5.57E-05	0.327039
8061035	NM_080676	MACROD2	NM_080676 // MACROD2 // MACRO domain containing 2 // 20p12.1 // 140733 /// NM_001033087	0.000413353	-0.299527
7950990	NM_152313	SLC36A4	NM_152313 // SLC36A4 // solute carrier family 36 (proton/amino acid symporter), member	0.00109897	-0.272252
7986503	NR_003260	C15orf51	NR_003260 // C15orf51 // dynamin 1 pseudogene // 15q26.3 // 196968 /// AK302717 // C15o	0.00284934	0.248351
7926983	NM_183013	CREM	NM_183013 // CREM // cAMP responsive element modulator // 10p11.21 // 1390 /// NM_18301	6.53E-06	-0.367283

8028656	NM_022835	PLEKHG2	NM_022835 // PLEKHG2 // pleckstrin homology domain containing, family G (with RhoGef do	0.00246447	-0.255003
8176730	NM_001039567	RPS4Y2	NM_001039567 // RPS4Y2 // ribosomal protein S4, Y-linked 2 // Yq11.223 // 140032 /// EN	0.00234795	-0.254802
8133176	NM_014504	RABGEF1	NM_014504 // RABGEF1 // RAB guanine nucleotide exchange factor (GEF) 1 // 7q11.21 // 27	0.00154634	-0.272023
7938702	NR_026750	DKFZp686O2416(	NR_026750 // DKFZp686O24166 // hypothetical protein DKFZp686O24166 // 11p15.1 // 374383	2.11E-05	-0.348108
8045688	NM_007115	TNFAIP6	NM_007115 // TNFAIP6 // tumor necrosis factor, alpha-induced protein 6 // 2q23.3 // 713	7.20E-06	-0.36829
7897378	NM_016831	PER3	NM_016831 // PER3 // period homolog 3 (Drosophila) // 1p36.23 // 8863 /// ENST000003619	8.10E-05	0.33833
8119842	NM_203290	POLR1C	NM_203290 // POLR1C // polymerase (RNA) I polypeptide C, 30kDa // 6p21.1 // 9533 /// NM	0.00139565	-0.273311
8165682	---		---	0.0027278	0.252261
7945232	NM_139055	ADAMTS15	NM_139055 // ADAMTS15 // ADAM metallopeptidase with thrombospondin type 1 motif, 15 //	0.00113911	-0.278955
8100154	NM_006587	CORIN	NM_006587 // CORIN // corin, serine peptidase // 4p13-p12 // 10699 /// ENST00000273857	0.000368877	-0.304675
8051583	NM_000104	CYP1B1	NM_000104 // CYP1B1 // cytochrome P450, family 1, subfamily B, polypeptide 1 // 2p21 //	5.32E-05	-0.323537
7951034	NR_002920	SNORA8	NR_002920 // SNORA8 // small nucleolar RNA, H/ACA box 8 // 11q21 // 654320 /// AK128061	0.000295525	-0.298253

8066027	---		---	0.00183095	-0.26902
7975459	NM_015556	SIPA1L1	NM_015556 // SIPA1L1 // signal-induced proliferation-associated 1 like 1 // 14q24.2 //	0.00226585	-0.255596
7974363	NM_000953	PTGDR	NM_000953 // PTGDR // prostaglandin D2 receptor (DP) // 14q22.1 // 5729 /// ENST0000030	4.23E-06	0.368914
8170187	NM_000074	CD40LG	NM_000074 // CD40LG // CD40 ligand // Xq26 // 959 /// ENST00000370629 // CD40LG // CD40	0.00226892	0.262924
8113305	NM_001270	CHD1	NM_001270 // CHD1 // chromodomain helicase DNA binding protein 1 // 5q15-q21 // 1105 //	9.36E-05	-0.325717
8090637	---		---	0.00104795	0.277756
7961757	NM_003034	ST8SIA1	NM_003034 // ST8SIA1 // ST8 alpha-N-acetyl-neuraminide alpha-2,8-sialyltransferase 1 //	9.48E-06	0.368661
7910416	NM_014777	URB2	NM_014777 // URB2 // URB2 ribosome biogenesis 2 homolog (S. cerevisiae) // 1q42.13 // 9	0.000838981	-0.284915
8052908	NM_173535	CLEC4F	NM_173535 // CLEC4F // C-type lectin domain family 4, member F // 2p13.3 // 165530 ///	0.00226527	0.258436
8156521	NR_029483	MIRLET7F1	NR_029483 // MIRLET7F1 // microRNA let-7f-1 // 9q22.32 // 406888	0.000785035	0.280891
7937876	---		---	0.00260099	0.253401
8130993	NM_020223	FAM20C	NM_020223 // FAM20C // family with sequence similarity 20, member C // 7p22.3 // 56975	0.000907401	-0.283743
7985934	NM_020210	SEMA4B	NM_020210 // SEMA4B // sema domain, immunoglobulin domain (Ig), transmembrane domain (T	2.78E-05	-0.345407

8066939	NM_004776	B4GALT5	NM_004776 // B4GALT5 // UDP-Gal:betaGlcNAc beta 1,4- galactosyltransferase, polypeptide	1.07E-06	-0.400192
8166784	NM_004615	TSPAN7	NM_004615 // TSPAN7 // tetraspanin 7 // Xp11.4 // 7102 /// ENST00000378482 // TSPAN7 //	0.000734355	0.281124
7915015	NM_013285	GNL2	NM_013285 // GNL2 // guanine nucleotide binding protein-like 2 (nucleolar) // 1p34.3 //	0.000592695	-0.292204
7946983	NM_030754	SAA2	NM_030754 // SAA2 // serum amyloid A2 // 11p15.1-p14 // 6289 /// BC020795 // SAA2 // se	0.00242764	-0.25745
8175023	NM_016032	ZDHHC9	NM_016032 // ZDHHC9 // zinc finger, DHHC-type containing 9 // Xq26.1 // 51114 /// NM_00	0.000125292	-0.314983
7951032	NR_003026	SNORA1	NR_003026 // SNORA1 // small nucleolar RNA, H/ACA box 1 // 11q21 // 677792	7.89E-05	-0.333782
8119088	NR_037151	CDKN1A	NR_037151 // CDKN1A // cyclin-dependent kinase inhibitor 1A (p21, Cip1) // 6p21.2 // 10	7.01E-05	-0.324163
8040655	NM_033505	EPT1	NM_033505 // EPT1 // ethanolaminephosphotransferase 1 (CDP-ethanolamine-specific) // 2p	0.00238793	-0.26078
8146921	NM_172037	RDH10	NM_172037 // RDH10 // retinol dehydrogenase 10 (all-trans) // 8q21.11 // 157506 /// ENS	1.21E-05	-0.355207
7988687	NM_005254	GABPB1	NM_005254 // GABPB1 // GA binding protein transcription factor, beta subunit 1 // 15q21	0.00164743	-0.267345
7930008	NM_004741	NOLC1	NM_004741 // NOLC1 // nucleolar and coiled-body phosphoprotein 1 // 10q24.32 // 9221 //	5.71E-05	-0.327982

8036787	NM_003890	FCGBP	NM_003890 // FCGBP // Fc fragment of IgG binding protein // 19q13.1 // 8857 /// ENST000	0.0024457	-0.257996
8043909	NM_002518	NPAS2	NM_002518 // NPAS2 // neuronal PAS domain protein 2 // 2q11.2 // 4862 /// ENST000003356	5.54E-08	-0.441609
7925823	NM_012341	GTPBP4	NM_012341 // GTPBP4 // GTP binding protein 4 // 10p15-p14 // 23560 /// ENST00000360803	5.21E-05	-0.336846
8044499	NM_005415	SLC20A1	NM_005415 // SLC20A1 // solute carrier family 20 (phosphate transporter), member 1 // 2	0.000391018	-0.297893
8116760	NM_031480	RIOK1	NM_031480 // RIOK1 // RIO kinase 1 (yeast) // 6p24.3 // 83732 /// NM_153005 // RIOK1 //	0.00253614	-0.259847
7922889	NM_006469	IVNS1ABP	NM_006469 // IVNS1ABP // influenza virus NS1A binding protein // 1q25.1-q31.1 // 10625	0.000293235	-0.307467
8076515	NM_014570	ARFGAP3	NM_014570 // ARFGAP3 // ADP-ribosylation factor GTPase activating protein 3 // 22q13.2	4.21E-06	-0.381406
7934553	NM_032772	ZNF503	NM_032772 // ZNF503 // zinc finger protein 503 // 10q22.2 // 84858 /// ENST00000372524	0.00229809	0.254918
7963986	NM_002870	RAB13	NM_002870 // RAB13 // RAB13, member RAS oncogene family // 1q21.2 // 5872 /// ENST00000	0.00103411	-0.284633
8073422	NM_003216	TEF	NM_003216 // TEF // thyrotrophic embryonic factor // 22q13|22q13.2 // 7008 /// NM_00114	0.000231745	0.31071
8117368	NM_003542	HIST1H4C	NM_003542 // HIST1H4C // histone cluster 1, H4c // 6p21.3 // 8364 /// BC130558 // HIST1	0.000294468	0.308084

8135915	NM_013332	C7orf68	NM_013332 // C7orf68 // chromosome 7 open reading frame 68 // 7q32.1 // 29923 /// NM_00	1.95E-07	-0.43306
8041168	NR_002741	SNORD53	NR_002741 // SNORD53 // small nucleolar RNA, C/D box 53 // 2p23.2 // 26796	7.16E-06	-0.359973
8130422	NM_173515	CNKSR3	NM_173515 // CNKSR3 // CNKSR family member 3 // 6q25.2 // 154043 /// ENST00000367213 //	3.43E-05	-0.346386
8113220	NM_012081	ELL2	NM_012081 // ELL2 // elongation factor, RNA polymerase II, 2 // 5q15 // 22936 /// ENST0	1.20E-06	-0.4007
7927936	NM_004728	DDX21	NM_004728 // DDX21 // DEAD (Asp-Glu-Ala-Asp) box polypeptide 21 // 10q21 // 9188 /// EN	4.34E-06	-0.373966
7935627	NM_002079	GOT1	NM_002079 // GOT1 // glutamic-oxaloacetic transaminase 1, soluble (aspartate aminotrans	0.000531448	-0.295758
7991581	NM_014918	CHSY1	NM_014918 // CHSY1 // chondroitin sulfate synthase 1 // 15q26.3 // 22856 /// ENST000002	8.07E-06	-0.364946
8125919	NM_001145775	FKBP5	NM_001145775 // FKBP5 // FK506 binding protein 5 // 6p21.31 // 2289 /// NM_004117 // FK	0.00126047	-0.278665
8166157	NM_203281	BMX	NM_203281 // BMX // BMX non-receptor tyrosine kinase // Xp22.2 // 660 /// NM_001721 //	0.000879576	-0.280169
8091523	NM_176894	P2RY13	NM_176894 // P2RY13 // purinergic receptor P2Y, G-protein coupled, 13 // 3q24 // 53829	0.000755305	0.288024
8084732	NM_022147	RTP4	NM_022147 // RTP4 // receptor (chemosensory) transporter protein 4 // 3q27.3 // 64108 /	3.52E-05	0.345681

8010287	NM_030968	C1QTNF1	NM_030968 // C1QTNF1 // C1q and tumor necrosis factor related protein 1 // 17q25.3 // 1	0.00180178	-0.260385
8103725	---		---	0.00123208	-0.278376
7906995	NM_012474	UCK2	NM_012474 // UCK2 // uridine-cytidine kinase 2 // 1q23 // 7371 /// ENST00000367879 // U	0.00289131	-0.251541
8115814	NM_001017995	SH3PXD2B	NM_001017995 // SH3PXD2B // SH3 and PX domains 2B // 5q35.1 // 285590 /// ENST000003116	3.97E-07	-0.417609
8166096	NM_152634	TCEANC	NM_152634 // TCEANC // transcription elongation factor A (SII) N-terminal and central d	0.00261015	0.253357
8021635	NM_001143818	SERPINB2	NM_001143818 // SERPINB2 // serpin peptidase inhibitor, clade B (ovalbumin), member 2 /	6.00E-05	-0.336096
7927146	NM_018590	CSGALNACT2	NM_018590 // CSGALNACT2 // chondroitin sulfate N-acetylgalactosaminyltransferase 2 // 1	0.000303526	-0.304161
8160637	NM_001497	B4GALT1	NM_001497 // B4GALT1 // UDP-Gal:betaGlcNAc beta 1,4- galactosyltransferase, polypeptide	0.000659324	-0.289386
8048733	NM_004457	ACSL3	NM_004457 // ACSL3 // acyl-CoA synthetase long-chain family member 3 // 2q34-q35 // 218	0.00264728	-0.257174
8041170	---		---	3.83E-05	-0.344616
8085628	NM_015199	ANKRD28	NM_015199 // ANKRD28 // ankyrin repeat domain 28 // 3p25.1 // 23243 /// NM_001195098 //	0.000592192	-0.288562

Supplemental Table S4. Differentially expressed genes at 5%FDR between severe and mild IIP, as defined by percent predicted FVC.

Transcript ID	gene_assignment	Gene Symbol	RefSeq	p-value(Severe vs. Mild; FVC)	Fold-Change(Severe vs. Mild; FVC)
7922229	NM_000450 // SELE // selectin E // 1q22-q25 // 6401 /// ENST00000333360 // SELE // sele	SELE	NM_000450	2.86E-05	2.79961
7951271	NM_002421 // MMP1 // matrix metallopeptidase 1 (interstitial collagenase) // 11q22.3 //	MMP1	NM_002421	0.00214136	2.614
8135069	NM_000602 // SERPINE1 // serpin peptidase inhibitor, clade E (nexin, plasminogen activa	SERPINE1	NM_000602	8.16E-06	2.60569
7921821	NM_005099 // ADAMTS4 // ADAM metallopeptidase with thrombospondin type 1 motif, 4 // 1q	ADAMTS4	NM_005099	2.81E-06	2.41167
8131803	NM_000600 // IL6 // interleukin 6 (interferon, beta 2) // 7p21 // 3569 /// ENST00000404	IL6	NM_000600	0.000870402	2.3245
7997188	NM_005143 // HP // haptoglobin // 16q22.1 // 3240 /// NM_001126102 // HP // haptoglobin	HP	NM_005143	0.00250618	2.29351
8146957	NM_015886 // PI15 // peptidase inhibitor 15 // 8q21.11 // 51050 /// ENST00000260113 //	PI15	NM_015886	1.73E-06	2.15761
8083594	NM_002852 // PTX3 // pentraxin 3, long // 3q25 // 5806 /// ENST00000295927 // PTX3 // p	PTX3	NM_002852	4.42E-05	2.04807
8043981	NM_004633 // IL1R2 // interleukin 1 receptor, type II // 2q12 // 7850 /// NM_173343 //	IL1R2	NM_004633	2.45E-05	1.99816

7982597	NM_003246 // THBS1 // thrombospondin 1 // 15q15 // 7057 /// ENST00000260356 // THBS1 //	THBS1	NM_003246	8.31E-06	1.99534
8169504	NM_007231 // SLC6A14 // solute carrier family 6 (amino acid transporter), member 14 //	SLC6A14	NM_007231	0.000176811	1.95371
8095744	NM_001657 // AREG // amphiregulin // 4q13-q21 // 374 /// BC009799 // AREG // amphiregul	AREG	NM_001657	0.00184122	1.94836
7968351	NM_032849 // C13orf33 // chromosome 13 open reading frame 33 // 13q12.3 // 84935 /// EN	C13orf33	NM_032849	5.64E-06	1.93964
8103725	---		---	6.40E-05	1.84996
8156848	NM_006981 // NR4A3 // nuclear receptor subfamily 4, group A, member 3 // 9q22 // 8013 /	NR4A3	NM_006981	0.000459052	1.84106
8095736	NM_001657 // AREG // amphiregulin // 4q13-q21 // 374 /// BC009799 // AREG // amphiregul	AREG	NM_001657	0.00195252	1.84018
7933084	NM_005746 // NAMPT // nicotinamide phosphoribosyltransferase // 7q22.3 // 10135 /// ENS	NAMPT	NM_005746	1.99E-06	1.84005
8095680	NM_000584 // IL8 // interleukin 8 // 4q13-q21 // 3576 /// ENST00000307407 // IL8 // int	IL8	NM_000584	0.000178481	1.82821
8045688	NM_007115 // TNFAIP6 // tumor necrosis factor, alpha-induced protein 6 // 2q23.3 // 713	TNFAIP6	NM_007115	0.000441078	1.79088
7995838	NM_005952 // MT1X // metallothionein 1X // 16q13 // 4501 /// ENST00000394485 // MT1X //	MT1X	NM_005952	0.000183149	1.78544
8152617	NM_005328 // HAS2 // hyaluronan synthase 2 // 8q24.12 // 3037 /// ENST00000303924 // HA	HAS2	NM_005328	0.000191787	1.78279

7976496	NM_001085 // SERPINA3 // serpin peptidase inhibitor, clade A (alpha-1 antiproteinase, a	SERPINA3	NM_001085	3.28E-05	1.76344
8162531	---		---	0.000256061	1.75877
8088560	NM_182920 // ADAMTS9 // ADAM metallopeptidase with thrombospondin type 1 motif, 9 // 3p	ADAMTS9	NM_182920	8.49E-05	1.75415
7995806	NM_005946 // MT1A // metallothionein 1A // 16q13 // 4489 /// ENST00000443255 // MT1A //	MT1A	NM_005946	0.000177745	1.74656
8126784	NM_001168357 // PLA2G7 // phospholipase A2, group VII (platelet-activating factor acety	PLA2G7	NM_001168357	0.000565478	1.72507
7995783	NM_005953 // MT2A // metallothionein 2A // 16q13 // 4502 /// ENST00000245185 // MT2A //	MT2A	NM_005953	0.000349741	1.71211
8048864	NM_004591 // CCL20 // chemokine (C-C motif) ligand 20 // 2q33-q37 // 6364 /// NM_001130	CCL20	NM_004591	0.00138135	1.70949
8021635	NM_001143818 // SERPINB2 // serpin peptidase inhibitor, clade B (ovalbumin), member 2 /	SERPINB2	NM_001143818	0.00103298	1.70884
8006433	NM_002982 // CCL2 // chemokine (C-C motif) ligand 2 // 17q11.2-q12 // 6347 /// ENST0000	CCL2	NM_002982	0.000340707	1.69989
8069676	NM_006988 // ADAMTS1 // ADAM metallopeptidase with thrombospondin type 1 motif, 1 // 21	ADAMTS1	NM_006988	0.00115356	1.68584
8095697	NM_001511 // CXCL1 // chemokine (C-X-C motif) ligand 1 (melanoma growth stimulating act	CXCL1	NM_001511	0.000250869	1.68378
8051583	NM_000104 // CYP1B1 // cytochrome P450, family 1, subfamily B, polypeptide 1 // 2p21 //	CYP1B1	NM_000104	7.47E-05	1.67789

8095362	NM_005953 // MT2A // metallothionein 2A // 16q13 // 4502 /// ENST00000245185 // MT2A //	MT2A	NM_005953	0.00020243	1.67655
8018864	NM_003955 // SOCS3 // suppressor of cytokine signaling 3 // 17q25.3 // 9021 /// ENST000	SOCS3	NM_003955	0.000953972	1.65805
8122265	NM_006290 // TNFAIP3 // tumor necrosis factor, alpha-induced protein 3 // 6q23 // 7128	TNFAIP3	NM_006290	0.000587197	1.65626
7951259	NM_002425 // MMP10 // matrix metallopeptidase 10 (stromelysin 2) // 11q22.3 // 4319 ///	MMP10	NM_002425	0.000946768	1.65575
8142120	NM_005746 // NAMPT // nicotinamide phosphoribosyltransferase // 7q22.3 // 10135 /// ENS	NAMPT	NM_005746	3.91E-06	1.65364
7963410	NM_173086 // KRT6C // keratin 6C // 12q13.13 // 286887 /// NM_005555 // KRT6B // kerati	KRT6C	NM_173086	0.0021413	1.65322
8103311	NM_000508 // FGA // fibrinogen alpha chain // 4q28 // 2243 /// NM_021871 // FGA // fibr	FGA	NM_000508	0.000635348	1.63688
8162276	NM_005384 // NFIL3 // nuclear factor, interleukin 3 regulated // 9q22 // 4783 /// ENST0	NFIL3	NM_005384	6.86E-06	1.63076
7903358	NM_001078 // VCAM1 // vascular cell adhesion molecule 1 // 1p32-p31 // 7412 /// NM_0806	VCAM1	NM_001078	0.00131073	1.60612
7965873	NM_001111283 // IGF1 // insulin-like growth factor 1 (somatomedin C) // 12q23.2 // 3479	IGF1	NM_001111283	0.00141048	1.60551
8001531	NM_005950 // MT1G // metallothionein 1G // 16q13 // 4495 /// BC020757 // MT1G // metall	MT1G	NM_005950	0.000421606	1.59631

8148317	NM_002467 // MYC // v-myc myelocytomatosis viral oncogene homolog (avian) // 8q24.21 //	MYC	NM_002467	0.0002345	1.59469
7997642	NM_031476 // CRISPLD2 // cysteine-rich secretory protein LCCL domain containing 2 // 16	CRISPLD2	NM_031476	7.12E-05	1.59428
7974851	NM_001530 // HIF1A // hypoxia inducible factor 1, alpha subunit (basic helix-loop-helix	HIF1A	NM_001530	2.65E-07	1.56659
7939492	NM_001145033 // C11orf96 // chromosome 11 open reading frame 96 // 11p11.2 // 387763 //	C11orf96	NM_001145033	2.82E-05	1.5573
7920238	NM_005621 // S100A12 // S100 calcium binding protein A12 // 1q21 // 6283 /// ENST000003	S100A12	NM_005621	0.00122495	1.55463
8104930	NM_004172 // SLC1A3 // solute carrier family 1 (glial high affinity glutamate transport	SLC1A3	NM_004172	0.00115795	1.55063
8146921	NM_172037 // RDH10 // retinol dehydrogenase 10 (all-trans) // 8q21.11 // 157506 /// ENS	RDH10	NM_172037	6.56E-07	1.5493
8003298	NM_003486 // SLC7A5 // solute carrier family 7 (cationic amino acid transporter, y+ sys	SLC7A5	NM_003486	3.31E-05	1.54019
7995803	NR_036677 // MT1JP // metallothionein 1J (pseudogene) // 16q13 // 4498 /// AF348994 //	MT1JP	NR_036677	0.00103898	1.5389
7922162	NM_006996 // SLC19A2 // solute carrier family 19 (thiamine transporter), member 2 // 1q	SLC19A2	NM_006996	0.000172398	1.5335
8077270	NM_006614 // CHL1 // cell adhesion molecule with homology to L1CAM (close homolog of L1	CHL1	NM_006614	0.000169042	1.5324

7963421	NM_005554 // KRT6A // keratin 6A // 12q12-q13 // 3853 /// ENST00000330722 // KRT6A // k	KRT6A	NM_005554	0.000368401	1.52875
7923547	NM_001276 // CHI3L1 // chitinase 3-like 1 (cartilage glycoprotein-39) // 1q32.1 // 1116	CHI3L1	NM_001276	0.00199224	1.52814
7953749	NM_080387 // CLEC4D // C-type lectin domain family 4, member D // 12p13.31 // 338339 //	CLEC4D	NM_080387	0.000151709	1.52499
8121515	NM_018593 // SLC16A10 // solute carrier family 16, member 10 (aromatic amino acid trans	SLC16A10	NM_018593	0.000480183	1.52153
7912157	NM_018948 // ERRFI1 // ERBB receptor feedback inhibitor 1 // 1p36 // 54206 /// ENST0000	ERRFI1	NM_018948	0.000299596	1.51926
8113220	NM_012081 // ELL2 // elongation factor, RNA polymerase II, 2 // 5q15 // 22936 /// ENST0	ELL2	NM_012081	2.62E-05	1.51199
8145122	NM_001128431 // SLC39A14 // solute carrier family 39 (zinc transporter), member 14 // 8	SLC39A14	NM_001128431	1.88E-05	1.50678
8135915	NM_013332 // C7orf68 // chromosome 7 open reading frame 68 // 7q32.1 // 29923 /// NM_00	C7orf68	NM_013332	0.00100409	1.49553
8062571	NM_030919 // FAM83D // family with sequence similarity 83, member D // 20q11.22-q12 //	FAM83D	NM_030919	0.00203347	1.49451
8129618	NM_004666 // VNN1 // vanin 1 // 6q23-q24 // 8876 /// ENST00000367928 // VNN1 // vanin 1	VNN1	NM_004666	0.00071095	1.49379
7921344	NM_012081 // ELL2 // elongation factor, RNA polymerase II, 2 // 5q15 // 22936 /// ENST0	ELL2	NM_012081	3.03E-05	1.4933

8063923	NM_016354 // SLCO4A1 // solute carrier organic anion transporter family, member 4A1 //	SLCO4A1	NM_016354	0.000793926	1.48976
8106743	NM_004385 // VCAN // versican // 5q14.3 // 1462 /// NM_001164097 // VCAN // versican //	VCAN	NM_004385	0.000136297	1.48638
8103951	NM_001995 // ACSL1 // acyl-CoA synthetase long-chain family member 1 // 4q35 // 2180 //	ACSL1	NM_001995	1.31E-05	1.48422
8102800	NM_014331 // SLC7A11 // solute carrier family 7, (cationic amino acid transporter, y+ s	SLC7A11	NM_014331	0.000736783	1.48069
7902227	NM_001924 // GADD45A // growth arrest and DNA-damage-inducible, alpha // 1p31.2 // 1647	GADD45A	NM_001924	6.41E-08	1.48043
8021169	NM_006033 // LIPG // lipase, endothelial // 18q21.1 // 9388 /// ENST00000261292 // LIPG	LIPG	NM_006033	0.000704718	1.48012
8041048	NM_005253 // FOSL2 // FOS-like antigen 2 // 2p23.3 // 2355 /// ENST00000264716 // FOSL2	FOSL2	NM_005253	4.78E-05	1.47885
8070665	NM_173354 // SIK1 // salt-inducible kinase 1 // 21q22.3 // 150094 /// ENST00000270162 /	SIK1	NM_173354	0.0026759	1.47781
8095376	NM_005953 // MT2A // metallothionein 2A // 16q13 // 4502 /// ENST00000245185 // MT2A //	MT2A	NM_005953	0.000304752	1.47366
8048717	NM_152386 // SGPP2 // sphingosine-1-phosphate phosphatase 2 // 2q36.1 // 130367 /// ENS	SGPP2	NM_152386	0.000234149	1.47342
8166632	NM_001128127 // GK // glycerol kinase // Xp21.3 // 2710 /// NM_203391 // GK // glycerol	GK	NM_001128127	5.25E-05	1.47125

8028908	NM_025194 // ITPKC // inositol 1,4,5-trisphosphate 3-kinase C // 19q13.1 // 80271 /// E	ITPKC	NM_025194	0.000391718	1.47075
8174103	NM_001128127 // GK // glycerol kinase // Xp21.3 // 2710 /// NM_000167 // GK // glycerol	GK	NM_001128127	2.99E-05	1.46511
8097903	NM_003264 // TLR2 // toll-like receptor 2 // 4q32 // 7097 /// ENST00000260010 // TLR2 /	TLR2	NM_003264	0.000106178	1.46181
8093104	NM_138461 // TM4SF19 // transmembrane 4 L six family member 19 // 3q29 // 116211 /// EN	TM4SF19	NM_138461	0.00114711	1.45906
7963946	NM_002429 // MMP19 // matrix metallopeptidase 19 // 12q14 // 4327 /// ENST00000322569 /	MMP19	NM_002429	0.00166156	1.45571
7917875	NM_001993 // F3 // coagulation factor III (thromboplastin, tissue factor) // 1p22-p21 /	F3	NM_001993	4.38E-07	1.45561
7969640	NM_182848 // CLDN10 // claudin 10 // 13q31-q34 // 9071 /// NM_001160100 // CLDN10 // cl	CLDN10	NM_182848	0.000434035	1.44747
8043909	NM_002518 // NPAS2 // neuronal PAS domain protein 2 // 2q11.2 // 4862 /// ENST000003356	NPAS2	NM_002518	0.000670363	1.44708
8059376	NM_006216 // SERPINE2 // serpin peptidase inhibitor, clade E (nexin, plasminogen activa	SERPINE2	NM_006216	0.000134419	1.44631
7965040	NM_007350 // PHLDA1 // pleckstrin homology-like domain, family A, member 1 // 12q15 //	PHLDA1	NM_007350	3.54E-07	1.44238
8069541	NM_022136 // SAMSN1 // SAM domain, SH3 domain and nuclear localization signals 1 // 21q	SAMSN1	NM_022136	0.00050701	1.43655

7943998	NM_006169 // NNMT // nicotinamide N-methyltransferase // 11q23.1 // 4837 /// ENST000002	NNMT	NM_006169	4.68E-05	1.43635
7897449	NM_025106 // SPSB1 // splA/ryanodine receptor domain and SOCS box containing 1 // 1p36.	SPSB1	NM_025106	4.25E-06	1.4359
8083569	NM_015508 // TIPARP // TCDD-inducible poly(ADP-ribose) polymerase // 3q25.31 // 25976 /	TIPARP	NM_015508	0.000208445	1.43405
8156043	NM_058179 // PSAT1 // phosphoserine aminotransferase 1 // 9q21.2 // 29968 /// NM_021154	PSAT1	NM_058179	9.00E-06	1.43389
8168749	NM_014467 // SRPX2 // sushi-repeat-containing protein, X-linked 2 // Xq21.33-q23 // 272	SRPX2	NM_014467	2.98E-05	1.42655
8044391	NM_006343 // MERTK // c-mer proto-oncogene tyrosine kinase // 2q14.1 // 10461 /// ENST0	MERTK	NM_006343	1.48E-05	1.42606
7946983	NM_030754 // SAA2 // serum amyloid A2 // 11p15.1-p14 // 6289 /// BC020795 // SAA2 // se	SAA2	NM_030754	0.00277929	1.42568
8044574	NM_173842 // IL1RN // interleukin 1 receptor antagonist // 2q14.2 // 3557 /// NM_173841	IL1RN	NM_173842	0.00158304	1.42563
8106098	NM_005909 // MAP1B // microtubule-associated protein 1B // 5q13 // 4131 /// ENST0000029	MAP1B	NM_005909	0.000193657	1.419
8115814	NM_001017995 // SH3PXD2B // SH3 and PX domains 2B // 5q35.1 // 285590 /// ENST000003116	SH3PXD2B	NM_001017995	2.96E-05	1.4157
8105302	NM_006350 // FST // follistatin // 5q11.2 // 10468 /// NM_013409 // FST // follistatin	FST	NM_006350	0.000473313	1.40945

8157216	NM_003358 // UGCG // UDP-glucose ceramide glucosyltransferase // 9q31 // 7357 /// ENST0	UGCG	NM_003358	5.26E-06	1.40642
8157487	NM_002581 // PAPPA // pregnancy-associated plasma protein A, pappalysin 1 // 9q33.2 //	PAPPA	NM_002581	0.00180753	1.40515
8151816	NM_005261 // GEM // GTP binding protein overexpressed in skeletal muscle // 8q13-q21 //	GEM	NM_005261	0.00230396	1.40081
7911178	NM_004895 // NLRP3 // NLR family, pyrin domain containing 3 // 1q44 // 114548 /// NM_00	NLRP3	NM_004895	0.00172952	1.40079
8007931	NM_000212 // ITGB3 // integrin, beta 3 (platelet glycoprotein IIIa, antigen CD61) // 17	ITGB3	NM_000212	5.20E-05	1.40032
8063382	NM_005985 // SNAI1 // snail homolog 1 (Drosophila) // 20q13.2 // 6615 /// ENST000002440	SNAI1	NM_005985	0.000215646	1.39962
8042830	NR_027405 // MTHFD2 // methylenetetrahydrofolate dehydrogenase (NADP+ dependent) 2, met	MTHFD2	NR_027405	2.45E-05	1.39882
8099685	NM_018176 // LGI2 // leucine-rich repeat LGI family, member 2 // 4p15.2 // 55203 /// EN	LGI2	NM_018176	0.000178722	1.39537
7906863	NM_003115 // UAP1 // UDP-N-acteylglucosamine pyrophosphorylase 1 // 1q23.3 // 6675 ///	UAP1	NM_003115	6.69E-05	1.39372
8144786	NM_003046 // SLC7A2 // solute carrier family 7 (cationic amino acid transporter, y+ sys	SLC7A2	NM_003046	0.000426259	1.38698
8038899	NM_001193306 // FPR1 // formyl peptide receptor 1 // 19q13.4 // 2357 /// NM_002029 // F	FPR1	NM_001193306	0.00279367	1.38566

7927936	NM_004728 // DDX21 // DEAD (Asp-Glu-Ala-Asp) box polypeptide 21 // 10q21 // 9188 /// EN	DDX21	NM_004728	4.37E-05	1.38336
8130556	NM_001024465 // SOD2 // superoxide dismutase 2, mitochondrial // 6q25.3 // 6648 /// NM_	SOD2	NM_001024465	0.000766136	1.38184
8113709	NM_002317 // LOX // lysyl oxidase // 5q23.2 // 4015 /// NM_001178102 // LOX // lysyl ox	LOX	NM_002317	0.000637521	1.38174
8042503	NM_002357 // MXD1 // MAX dimerization protein 1 // 2p13-p12 // 4084 /// ENST00000264444	MXD1	NM_002357	0.000822332	1.38139
7995834	NR_003669 // MT1IP // metallothionein 1I (pseudogene) // 16q13 // 644314 /// AF348997 /	MT1IP	NR_003669	0.000566904	1.37194
8114249	NM_004887 // CXCL14 // chemokine (C-X-C motif) ligand 14 // 5q31 // 9547 /// ENST000003	CXCL14	NM_004887	0.00122124	1.36932
7986446	NM_000693 // ALDH1A3 // aldehyde dehydrogenase 1 family, member A3 // 15q26.3 // 220 //	ALDH1A3	NM_000693	0.0019769	1.36629
8092849	NM_024524 // ATP13A3 // ATPase type 13A3 // 3q29 // 79572 /// ENST00000256031 // ATP13A	ATP13A3	NM_024524	1.98E-07	1.36516
8148435	NM_003882 // WISP1 // WNT1 inducible signaling pathway protein 1 // 8q24.22 // 8840 ///	WISP1	NM_003882	0.000713894	1.36219
8150592	NM_005195 // CEBPD // CCAAT/enhancer binding protein (C/EBP), delta // 8p11.2-p11.1 //	CEBPD	NM_005195	0.000102255	1.3609
8140840	NM_024636 // STEAP4 // STEAP family member 4 // 7q21.12 // 79689 /// ENST00000380079 //	STEAP4	NM_024636	0.000754938	1.35605

8140668	NM_006080 // SEMA3A // sema domain, immunoglobulin domain (Ig), short basic domain, sec	SEMA3A	NM_006080	0.00192107	1.35498
8043995	NM_000877 // IL1R1 // interleukin 1 receptor, type I // 2q12 // 3554 /// ENST0000023394	IL1R1	NM_000877	3.84E-05	1.35003
8105040	NM_003999 // OSMR // oncostatin M receptor // 5p13.1 // 9180 /// NM_001168355 // OSMR /	OSMR	NM_003999	2.97E-05	1.34891
8052654	NM_020651 // PELI1 // pellino homolog 1 (Drosophila) // 2p13.3 // 57162 /// ENST0000035	PELI1	NM_020651	0.000644227	1.34487
7995829	NM_005951 // MT1H // metallothionein 1H // 16q13 // 4496 /// ENST00000332374 // MT1H //	MT1H	NM_005951	6.43E-05	1.3397
7973067	NM_000270 // PNP // purine nucleoside phosphorylase // 14q13.1 // 4860 /// ENST00000361	PNP	NM_000270	0.000258098	1.33954
7915787	NM_003629 // PIK3R3 // phosphoinositide-3-kinase, regulatory subunit 3 (gamma) // 1p34.	PIK3R3	NM_003629	4.85E-05	1.33346
7940253	NM_139249 // MS4A6E // membrane-spanning 4-domains, subfamily A, member 6E // 11q12.2 /	MS4A6E	NM_139249	0.00168643	1.33343
7996100	NM_170776 // GPR97 // G protein-coupled receptor 97 // 16q21 // 222487 /// ENST00000333	GPR97	NM_170776	0.000638492	1.33084
7922610	NM_007314 // ABL2 // v-abl Abelson murine leukemia viral oncogene homolog 2 // 1q25.2 /	ABL2	NM_007314	3.84E-05	1.32693
8148304	NM_025195 // TRIB1 // tribbles homolog 1 (Drosophila) // 8q24.13 // 10221 /// ENST00000	TRIB1	NM_025195	0.00261694	1.32643

7956878	NM_007199 // IRAK3 // interleukin-1 receptor-associated kinase 3 // 12q14.3 // 11213 //	IRAK3	NM_007199	0.000616331	1.32591
8084064	NM_006636 // MTHFD2 // methylenetetrahydrofolate dehydrogenase (NADP+ dependent) 2, met	MTHFD2	NM_006636	0.00106444	1.32506
8070557	NM_001098402 // ZNF295 // zinc finger protein 295 // 21q22.3 // 49854 /// NM_020727 //	ZNF295	NM_001098402	0.000118174	1.32347
8161648	NM_001206 // KLF9 // Kruppel-like factor 9 // 9q13 // 687 /// ENST00000377126 // KLF9 /	KLF9	NM_001206	0.00197735	1.32042
8084794	NM_002182 // IL1RAP // interleukin 1 receptor accessory protein // 3q28 // 3556 /// NM_	IL1RAP	NM_002182	0.000503375	1.32016
8029465	NM_005178 // BCL3 // B-cell CLL/lymphoma 3 // 19q13.1-q13.2 // 602 /// BC064993 // BCL3	BCL3	NM_005178	0.000151707	1.31999
8171449	NM_021804 // ACE2 // angiotensin I converting enzyme (peptidyl-dipeptidase A) 2 // Xp22	ACE2	NM_021804	1.80E-05	1.31976
8077441	NM_003670 // BHLHE40 // basic helix-loop-helix family, member e40 // 3p26 // 8553 /// E	BHLHE40	NM_003670	0.000980003	1.31853
8085360	NM_003256 // TIMP4 // TIMP metallopeptidase inhibitor 4 // 3p25 // 7079 /// ENST0000028	TIMP4	NM_003256	0.000627755	1.31841
8092691	NM_001706 // BCL6 // B-cell CLL/lymphoma 6 // 3q27 // 604 /// NM_001130845 // BCL6 // B	BCL6	NM_001706	0.000103058	1.31683
7910611	NM_002245 // KCNK1 // potassium channel, subfamily K, member 1 // 1q42-q43 // 3775 ///	KCNK1	NM_002245	0.000138249	1.31636

8026631	NM_003950 // F2RL3 // coagulation factor II (thrombin) receptor-like 3 // 19p12 // 9002	F2RL3	NM_003950	0.00273179	1.31477
7995813	NR_027781 // MT1DP // metallothionein 1D (pseudogene) // 16q13 // 326343 /// NR_003658	MT1DP	NR_027781	0.0018875	1.30961
7900426	NM_022733 // SMAP2 // small ArfGAP2 // 1p35.3-p34.1 // 64744 /// ENST00000372718 // SMA	SMAP2	NM_022733	0.0002194	1.3024
8174474	NM_022977 // ACSL4 // acyl-CoA synthetase long-chain family member 4 // Xq22.3-q23 // 2	ACSL4	NM_022977	0.000821816	1.30061
8160297	NM_001122 // PLIN2 // perilipin 2 // 9p22.1 // 123 /// ENST00000276914 // PLIN2 // peri	PLIN2	NM_001122	0.00231143	1.29842
7963406	NM_005555 // KRT6B // keratin 6B // 12q12-q13 // 3854 /// ENST00000252252 // KRT6B // k	KRT6B	NM_005555	0.000816907	1.29611
7974366	NM_000956 // PTGER2 // prostaglandin E receptor 2 (subtype EP2), 53kDa // 14q22 // 5732	PTGER2	NM_000956	0.00087961	1.2953
7957551	NM_003877 // SOCS2 // suppressor of cytokine signaling 2 // 12q // 8835 /// ENST0000034	SOCS2	NM_003877	0.00203023	1.29336
8097461	NM_012118 // CCRN4L // CCR4 carbon catabolite repression 4-like (S. cerevisiae) // 4q31	CCRN4L	NM_012118	0.000304188	1.29187
8175023	NM_016032 // ZDHHC9 // zinc finger, DHHC-type containing 9 // Xq26.1 // 51114 /// NM_00	ZDHHC9	NM_016032	0.000468169	1.29145
7929750	NM_020354 // ENTPD7 // ectonucleoside triphosphate diphosphohydrolase 7 // --- // 57089	ENTPD7	NM_020354	6.93E-05	1.28812

8003667	NM_002615 // SERPINF1 // serpin peptidase inhibitor, clade F (alpha-2 antiplasmin, pigm	SERPINF1	NM_002615	0.00241231	1.28651
8006999	NR_033662 // CSF3 // colony stimulating factor 3 (granulocyte) // 17q11.2-q12 // 1440 /	CSF3	NR_033662	0.000352025	1.28582
7930921	NM_004281 // BAG3 // BCL2-associated athanogene 3 // 10q25.2-q26.2 // 9531 /// ENST0000	BAG3	NM_004281	1.71E-06	1.28359
7975268	NM_001172 // ARG2 // arginase, type II // 14q24.1 // 384 /// NM_006370 // VTI1B // vesi	ARG2	NM_001172	0.000888735	1.28345
7904293	NM_020440 // PTGFRN // prostaglandin F2 receptor negative regulator // 1p13.1 // 5738 /	PTGFRN	NM_020440	0.00185755	1.28147
7943218	NM_015368 // PANX1 // pannexin 1 // 11q21 // 24145 /// ENST00000227638 // PANX1 // pann	PANX1	NM_015368	6.22E-05	1.28052
7945232	NM_139055 // ADAMTS15 // ADAM metallopeptidase with thrombospondin type 1 motif, 15 //	ADAMTS15	NM_139055	0.00104612	1.27963
8119161	NM_002648 // PIM1 // pim-1 oncogene // 6p21.2 // 5292 /// ENST00000373509 // PIM1 // pi	PIM1	NM_002648	0.00196744	1.27695
8099897	NM_003359 // UGDH // UDP-glucose 6-dehydrogenase // 4p15.1 // 7358 /// NM_001184700 //	UGDH	NM_003359	0.00221063	1.27609
8031374	NM_002000 // FCAR // Fc fragment of IgA, receptor for // 19q13.2-q13.4 // 2204 /// NM_1	FCAR	NM_002000	0.000962173	1.27459
7928589	NM_005729 // PPIF // peptidylprolyl isomerase F // 10q22-q23 // 10105 /// ENST000002251	PPIF	NM_005729	0.000324422	1.27363

7938702	NR_026750 // DKFZp686O24166 // hypothetical protein DKFZp686O24166 // 11p15.1 // 374383	DKFZp686O24166	NR_026750	0.000189602	1.27332
8167185	NM_003254 // TIMP1 // TIMP metallopeptidase inhibitor 1 // Xp11.3-p11.23 // 7076 /// EN	TIMP1	NM_003254	0.00124537	1.27328
7930008	NM_004741 // NOLC1 // nucleolar and coiled-body phosphoprotein 1 // 10q24.32 // 9221 //	NOLC1	NM_004741	0.000298766	1.27043
8066939	NM_004776 // B4GALT5 // UDP-Gal:betaGlcNAc beta 1,4- galactosyltransferase, polypeptide	B4GALT5	NM_004776	3.23E-05	1.26943
7970810	NM_003045 // SLC7A1 // solute carrier family 7 (cationic amino acid transporter, y+ sys	SLC7A1	NM_003045	0.000416714	1.26897
8034873	NM_013447 // EMR2 // egf-like module containing, mucin-like, hormone receptor-like 2 //	EMR2	NM_013447	0.00194942	1.26875
8080419	NM_206825 // GNL3 // guanine nucleotide binding protein-like 3 (nucleolar) // 3p21.1 //	GNL3	NM_206825	0.000253241	1.26785
8006123	NM_001304 // CPD // carboxypeptidase D // 17q11.2 // 1362 /// ENST00000225719 // CPD //	CPD	NM_001304	3.24E-05	1.26659
7938777	NM_005566 // LDHA // lactate dehydrogenase A // 11p15.4 // 3939 /// NR_028500 // LDHA /	LDHA	NM_005566	2.60E-05	1.26531
7958174	NM_003330 // TXNRD1 // thioredoxin reductase 1 // 12q23-q24.1 // 7296 /// NM_001093771	TXNRD1	NM_003330	1.41E-05	1.26456
8147206	NM_003821 // RIPK2 // receptor-interacting serine-threonine kinase 2 // 8q21 // 8767 //	RIPK2	NM_003821	0.00123154	1.26407

8152946	NM_004519 // KCNQ3 // potassium voltage-gated channel, KQT-like subfamily, member 3 //	KCNQ3	NM_004519	0.00124087	1.26315
8107706	NM_005573 // LMNB1 // lamin B1 // 5q23.2 // 4001 /// NM_001198557 // LMNB1 // lamin B1	LMNB1	NM_005573	0.00114733	1.26251
7939056	NM_003986 // BBOX1 // butyrobetaine (gamma), 2-oxoglutarate dioxygenase (gamma-butyrobe	BBOX1	NM_003986	0.00201594	1.26044
8169115	NM_198465 // NRK // Nik related kinase // Xq22.3 // 203447 /// BX538345 // NRK // Nik r	NRK	NM_198465	0.00205432	1.25804
8142136	NM_175884 // FLJ36031 // hypothetical protein FLJ36031 // 7q22.3 // 168455	FLJ36031	NM_175884	0.00103372	1.25692
7908672	NM_000299 // PKP1 // plakophilin 1 (ectodermal dysplasia/skin fragility syndrome) // 1q	PKP1	NM_000299	0.00132354	1.25677
8009417	NM_002266 // KPNA2 // karyopherin alpha 2 (RAG cohort 1, importin alpha 1) // 17q24.2 /	KPNA2	NM_002266	0.000126841	1.25604
8135955	NM_001219 // CALU // calumenin // 7q32.1 // 813 /// NM_001130674 // CALU // calumenin /	CALU	NM_001219	0.000397568	1.2555
8000028	NM_173475 // DCUN1D3 // DCN1, defective in cullin neddylation 1, domain containing 3 (S	DCUN1D3	NM_173475	0.00129169	1.25384
8062927	NM_002638 // PI3 // peptidase inhibitor 3, skin-derived // 20q13.12 // 5266 /// ENST000	PI3	NM_002638	0.00245467	1.25084
7952341	NM_024769 // ASAM // adipocyte-specific adhesion molecule // 11q24.1 // 79827 /// ENST0	ASAM	NM_024769	0.000692004	1.24871

8035506	NM_004750 // CRLF1 // cytokine receptor-like factor 1 // 19p12 // 9244 /// ENST00000392	CRLF1	NM_004750	0.00191819	1.2487
8041168	NR_002741 // SNORD53 // small nucleolar RNA, C/D box 53 // 2p23.2 // 26796	SNORD53	NR_002741	0.00180678	1.24838
8160637	NM_001497 // B4GALT1 // UDP-Gal:betaGlcNAc beta 1,4- galactosyltransferase, polypeptide	B4GALT1	NM_001497	2.01E-05	1.24791
7926983	NM_183013 // CREM // cAMP responsive element modulator // 10p11.21 // 1390 /// NM_18301	CREM	NM_183013	6.34E-05	1.24531
8133155	NM_003596 // TPST1 // tyrosylprotein sulfotransferase 1 // 7q11.21 // 8460 /// ENST0000	TPST1	NM_003596	1.09E-05	1.24501
7948088	NM_032315 // SLC25A33 // solute carrier family 25, member 33 // 1p36.22 // 84275 /// EN	SLC25A33	NM_032315	0.00126907	1.24493
8019737	NM_002266 // KPNA2 // karyopherin alpha 2 (RAG cohort 1, importin alpha 1) // 17q24.2 /	KPNA2	NM_002266	3.94E-05	1.24425
7966052	NM_004075 // CRY1 // cryptochrome 1 (photolyase-like) // 12q23-q24.1 // 1407 /// ENST00	CRY1	NM_004075	0.001967	1.24332
7996891	NM_032830 // CIRH1A // cirrhosis, autosomal recessive 1A (cirhin) // 16q22.1 // 84916 /	CIRH1A	NM_032830	0.000116778	1.24319
8042144	NM_002908 // REL // v-rel reticuloendotheliosis viral oncogene homolog (avian) // 2p13-	REL	NM_002908	0.00152168	1.24302
8082816	NM_021203 // SRPRB // signal recognition particle receptor, B subunit // 3q22.1 // 5847	SRPRB	NM_021203	9.31E-05	1.24109

8121277	NM_001624 // AIM1 // absent in melanoma 1 // 6q21 // 202 /// ENST00000369066 // AIM1 //	AIM1	NM_001624	0.00107918	1.24055
8065416	NM_001322 // CST2 // cystatin SA // 20p11.21 // 1470 /// ENST00000304725 // CST2 // cys	CST2	NM_001322	0.00185742	1.24037
8123644	NM_001069 // TUBB2A // tubulin, beta 2A // 6p25 // 7280 /// ENST00000333628 // TUBB2A /	TUBB2A	NM_001069	0.00211043	1.24027
7926531	NM_178815 // ARL5B // ADP-ribosylation factor-like 5B // 10p12.31 // 221079 /// ENST000	ARL5B	NM_178815	0.00157859	1.23949
8041149	NM_015131 // WDR43 // WD repeat domain 43 // 2p23.2 // 23160 /// ENST00000407426 // WDR	WDR43	NM_015131	0.000786157	1.23897
8048733	NM_004457 // ACSL3 // acyl-CoA synthetase long-chain family member 3 // 2q34-q35 // 218	ACSL3	NM_004457	0.000240756	1.23874
7936242	NM_033397 // ITPRIP // inositol 1,4,5-triphosphate receptor interacting protein // 10q2	ITPRIP	NM_033397	0.00129144	1.2381
7995825	NM_005949 // MT1F // metallothionein 1F // 16q13 // 4494 /// ENST00000334350 // MT1F //	MT1F	NM_005949	0.00166821	1.23805
8102482	NM_014822 // SEC24D // SEC24 family, member D (S. cerevisiae) // 4q26 // 9871 /// ENST0	SEC24D	NM_014822	5.53E-05	1.23625
8047217	NM_025147 // COQ10B // coenzyme Q10 homolog B (S. cerevisiae) // 2q33.1 // 80219 /// EN	COQ10B	NM_025147	0.0010711	1.23517
7995820	NM_005947 // MT1B // metallothionein 1B // 16q13 // 4490 /// ENST00000334346 // MT1B //	MT1B	NM_005947	0.000345137	1.23391

8032157	NM_014963 // SBNO2 // strawberry notch homolog 2 (Drosophila) // 19p13.3 // 22904 /// N	SBNO2	NM_014963	0.00118581	1.23352
8052762	NM_002056 // GFPT1 // glutamine--fructose-6-phosphate transaminase 1 // 2p13 // 2673 //	GFPT1	NM_002056	3.32E-06	1.23298
8024572	NM_002068 // GNA15 // guanine nucleotide binding protein (G protein), alpha 15 (Gq clas	GNA15	NM_002068	0.000338197	1.23258
7985934	NM_020210 // SEMA4B // sema domain, immunoglobulin domain (Ig), transmembrane domain (T	SEMA4B	NM_020210	8.83E-05	1.23248
8044499	NM_005415 // SLC20A1 // solute carrier family 20 (phosphate transporter), member 1 // 2	SLC20A1	NM_005415	0.000125552	1.23233
7929990	NM_015062 // PPRC1 // peroxisome proliferator-activated receptor gamma, coactivator-rel	PPRC1	NM_015062	0.0024005	1.23225
7956826	NM_015279 // TBC1D30 // TBC1 domain family, member 30 // 12q14.3 // 23329 /// AB449914	TBC1D30	NM_015279	2.93E-05	1.23193
8156761	NM_018946 // NANS // N-acetylneuraminic acid synthase // 9p24.1-p23 // 54187 /// ENST00	NANS	NM_018946	0.00227362	1.23139
8089835	NM_007085 // FSTL1 // follistatin-like 1 // 3q13.33 // 11167 /// NR_029584 // MIR198 //	FSTL1	NM_007085	0.000867018	1.22955
8042381	NM_020143 // PNO1 // partner of NOB1 homolog (S. cerevisiae) // 2p14 // 56902 /// ENST0	PNO1	NM_020143	0.000976526	1.2288
8153021	NM_003033 // ST3GAL1 // ST3 beta-galactoside alpha-2,3-sialyltransferase 1 // 8q24.22 /	ST3GAL1	NM_003033	0.000212248	1.22834

8132725	NM_003364 // UPP1 // uridine phosphorylase 1 // 7p12.3 // 7378 /// NM_181597 // UPP1 //	UPP1	NM_003364	0.000310416	1.22821
8062603	NM_003286 // TOP1 // topoisomerase (DNA) I // 20q12-q13.1 // 7150 /// ENST00000361337 /	TOP1	NM_003286	0.000662493	1.2278
7952046	NM_144765 // MPZL2 // myelin protein zero-like 2 // 11q24 // 10205 /// NM_005797 // MPZ	MPZL2	NM_144765	0.000886834	1.22706
8128850	NM_033125 // SLC22A16 // solute carrier family 22 (organic cation/carnitine transporter	SLC22A16	NM_033125	0.000449521	1.22632
8061186	NM_006363 // SEC23B // Sec23 homolog B (S. cerevisiae) // 20p11.23 // 10483 /// NM_0329	SEC23B	NM_006363	0.000298052	1.22565
7923659	NM_032833 // PPP1R15B // protein phosphatase 1, regulatory (inhibitor) subunit 15B // 1	PPP1R15B	NM_032833	0.00150878	1.22543
7935230	NM_002860 // ALDH18A1 // aldehyde dehydrogenase 18 family, member A1 // 10q24.3 // 5832	ALDH18A1	NM_002860	0.00045563	1.22494
7962327	NM_052885 // SLC2A13 // solute carrier family 2 (facilitated glucose transporter), memb	SLC2A13	NM_052885	4.80E-05	1.22375
8157092	NM_018112 // TMEM38B // transmembrane protein 38B // 9q31.2 // 55151 /// ENST0000037469	TMEM38B	NM_018112	0.00159696	1.22345
8107897	NM_003687 // PDLIM4 // PDZ and LIM domain 4 // 5q31.1 // 8572 /// NM_001131027 // PDLIM	PDLIM4	NM_003687	0.000392916	1.22339
8113073	NM_020801 // ARRDC3 // arrestin domain containing 3 // 5q14.3 // 57561 /// ENST00000265	ARRDC3	NM_020801	0.000516907	1.22332

7994280	NM_000418 // IL4R // interleukin 4 receptor // 16p12.1-p11.2 // 3566 /// NM_001008699 /	IL4R	NM_000418	0.000486945	1.2228
7952145	NM_006389 // HYOU1 // hypoxia up-regulated 1 // 11q23.1-q23.3 // 10525 /// NM_001130991	HYOU1	NM_006389	0.000751554	1.2225
7922889	NM_006469 // IVNS1ABP // influenza virus NS1A binding protein // 1q25.1-q31.1 // 10625	IVNS1ABP	NM_006469	9.32E-05	1.22229
7928291	NM_004273 // CHST3 // carbohydrate (chondroitin 6) sulfotransferase 3 // 10q22.1 // 946	CHST3	NM_004273	0.000282085	1.22215
8024228	NM_177401 // MIDN // midnolin // 19p13.3 // 90007 /// ENST00000300952 // MIDN // midnol	MIDN	NM_177401	0.00099954	1.22205
7925413	AF333388 // MT1P2 // metallothionein 1 pseudogene 2 // 1q43 // 645745	MT1P2	AF333388	0.00143544	1.22173
8052149	NM_014614 // PSME4 // proteasome (prosome, macropain) activator subunit 4 // 2p16.2 //	PSME4	NM_014614	5.85E-05	1.22082
8077786	NM_001570 // IRAK2 // interleukin-1 receptor-associated kinase 2 // 3p25.3 // 3656 ///	IRAK2	NM_001570	0.000931424	1.22006
7979085	NM_002863 // PYGL // phosphorylase, glycogen, liver // 14q21-q22 // 5836 /// NM_0011639	PYGL	NM_002863	0.000323792	1.21938
7978132	NM_024658 // IPO4 // importin 4 // 14q12 // 79711 /// ENST00000354464 // IPO4 // import	IPO4	NM_024658	0.000436413	1.21906
8123598	NM_030666 // SERPINB1 // serpin peptidase inhibitor, clade B (ovalbumin), member 1 // 6	SERPINB1	NM_030666	0.00163876	1.21874

7985134	NM_005530 // IDH3A // isocitrate dehydrogenase 3 (NAD+) alpha // 15q25.1-q25.2 // 3419	IDH3A	NM_005530	0.000209161	1.21852
8175217	NM_001448 // GPC4 // glypican 4 // Xq26.1 // 2239 /// ENST00000370828 // GPC4 // glypic	GPC4	NM_001448	0.00111047	1.21815
7949532	NM_005438 // FOSL1 // FOS-like antigen 1 // 11q13 // 8061 /// ENST00000312562 // FOSL1	FOSL1	NM_005438	0.00199367	1.21792
8029907	NM_001736 // C5AR1 // complement component 5a receptor 1 // 19q13.3-q13.4 // 728 /// EN	C5AR1	NM_001736	0.00188639	1.21658
7950990	NM_152313 // SLC36A4 // solute carrier family 36 (proton/amino acid symporter), member	SLC36A4	NM_152313	0.00158527	1.21643
8050278	NM_005742 // PDIA6 // protein disulfide isomerase family A, member 6 // 2p25.1 // 10130	PDIA6	NM_005742	1.53E-05	1.21514
7970655	NM_004685 // MTMR6 // myotubularin related protein 6 // 13q12 // 9107 /// ENST000003818	MTMR6	NM_004685	0.000161933	1.21448
7989277	NM_004998 // MYO1E // myosin IE // 15q21-q22 // 4643 /// ENST00000288235 // MYO1E // my	MYO1E	NM_004998	0.000168942	1.21416
8115851	NM_003714 // STC2 // stanniocalcin 2 // 5q35.1 // 8614 /// ENST00000265087 // STC2 // s	STC2	NM_003714	0.00197797	1.21408
8104449	NM_012073 // CCT5 // chaperonin containing TCP1, subunit 5 (epsilon) // 5p15.2 // 22948	CCT5	NM_012073	0.000383484	1.21387
8104601	NM_006317 // BASP1 // brain abundant, membrane attached signal protein 1 // 5p15.1 // 1	BASP1	NM_006317	0.000994948	1.21381

8063386	NM_005194 // CEBPB // CCAAT/enhancer binding protein (C/EBP), beta // 20q13.1 // 1051 /	CEBPB	NM_005194	0.000266483	1.21366
8076515	NM_014570 // ARFGAP3 // ADP-ribosylation factor GTPase activating protein 3 // 22q13.2	ARFGAP3	NM_014570	0.00035261	1.21224
7910427	NM_004481 // GALNT2 // UDP-N-acetyl-alpha-D-galactosamine:polypeptide N-acetylgalactosa	GALNT2	NM_004481	0.000405546	1.21219
7914603	NM_153341 // RNF19B // ring finger protein 19B // 1p35.1 // 127544 /// NM_001127361 //	RNF19B	NM_153341	0.00167844	1.211
8016708	NM_018509 // LRRC59 // leucine rich repeat containing 59 // 17q21.33 // 55379 /// ENST0	LRRC59	NM_018509	0.00143013	1.21007
8152828	NM_031415 // GSDMC // gasdermin C // 8q24.21 // 56169 /// ENST00000276708 // GSDMC // g	GSDMC	NM_031415	0.00108017	1.20975
8119492	NM_004053 // BYSL // bystin-like // 6p21.1 // 705 /// ENST00000230340 // BYSL // bystin	BYSL	NM_004053	0.000587786	1.20967
8100870	NM_014243 // ADAMTS3 // ADAM metallopeptidase with thrombospondin type 1 motif, 3 // 4q	ADAMTS3	NM_014243	0.00078549	1.20931
8052872	NM_003236 // TGFA // transforming growth factor, alpha // 2p13 // 7039 /// NM_001099691	TGFA	NM_003236	0.000394347	1.20834
7979179	NM_014584 // ERO1L // ERO1-like (S. cerevisiae) // 14q22.1 // 30001 /// ENST00000395686	ERO1L	NM_014584	0.000553896	1.20673
7915015	NM_013285 // GNL2 // guanine nucleotide binding protein-like 2 (nucleolar) // 1p34.3 //	GNL2	NM_013285	0.000161081	1.20555

8010901	NM_003585 // DOC2B // double C2-like domains, beta // 17p13.3 // 8447 /// ENST000003435	DOC2B	NM_003585	0.00148625	1.20392
7935425	NM_015179 // RRP12 // ribosomal RNA processing 12 homolog (S. cerevisiae) // 10q24.1 //	RRP12	NM_015179	0.00282941	1.20336
8117128	NM_001949 // E2F3 // E2F transcription factor 3 // 6p22 // 1871 /// ENST00000346618 //	E2F3	NM_001949	0.000224773	1.20314
8096635	NM_003998 // NFKB1 // nuclear factor of kappa light polypeptide gene enhancer in B-cell	NFKB1	NM_003998	0.000700618	1.20235
7978595	NM_013448 // BAZ1A // bromodomain adjacent to zinc finger domain, 1A // 14q13.2 // 1117	BAZ1A	NM_013448	0.0012086	1.19994
8062211	NM_032194 // RPF2 // ribosome production factor 2 homolog (S. cerevisiae) // 6q21 // 84	RPF2	NM_032194	0.0025293	1.19983
8017555	NM_001433 // ERN1 // endoplasmic reticulum to nucleus signaling 1 // 17q24.2 // 2081 //	ERN1	NM_001433	0.00217632	1.19909
7985213	NM_000745 // CHRNA5 // cholinergic receptor, nicotinic, alpha 5 // 15q24 // 1138 /// EN	CHRNA5	NM_000745	0.00183335	1.19821
8113305	NM_001270 // CHD1 // chromodomain helicase DNA binding protein 1 // 5q15-q21 // 1105 //	CHD1	NM_001270	0.00214054	1.1974
8119842	NM_203290 // POLR1C // polymerase (RNA) I polypeptide C, 30kDa // 6p21.1 // 9533 /// NM	POLR1C	NM_203290	0.000718975	1.19721
7910387	NM_021205 // RHOU // ras homolog gene family, member U // 1q42.11-q42.3 // 58480 /// EN	RHOU	NM_021205	0.00225905	1.19665

7924987	NM_000029 // AGT // angiotensinogen (serpin peptidase inhibitor, clade A, member 8) //	AGT	NM_000029	0.00173234	1.1956
7899462	NM_001048194 // RCC1 // regulator of chromosome condensation 1 // 1p36.1 // 1104 /// NR	RCC1	NM_001048194	0.000349287	1.19461
8008517	NM_198175 // NME1 // non-metastatic cells 1, protein (NM23A) expressed in // 17q21.3 //	NME1	NM_198175	0.00132925	1.19452
8051030	NM_021095 // SLC5A6 // solute carrier family 5 (sodium-dependent vitamin transporter),	SLC5A6	NM_021095	0.00144394	1.19444
7915084	NM_024640 // YRDC // yrdC domain containing (E. coli) // 1p34.3 // 79693 /// ENST000003	YRDC	NM_024640	0.00144254	1.19439
8099246	NM_025196 // GRPEL1 // GrpE-like 1, mitochondrial (E. coli) // 4p16 // 80273 /// ENST00	GRPEL1	NM_025196	0.000133274	1.19422
8020903	NM_020474 // GALNT1 // UDP-N-acetyl-alpha-D-galactosamine:polypeptide N-acetylgalactosa	GALNT1	NM_020474	0.001141	1.19377
7988687	NM_005254 // GABPB1 // GA binding protein transcription factor, beta subunit 1 // 15q21	GABPB1	NM_005254	0.000493944	1.19318
7910416	NM_014777 // URB2 // URB2 ribosome biogenesis 2 homolog (S. cerevisiae) // 1q42.13 // 9	URB2	NM_014777	0.00242352	1.19223
8052413	---		---	0.00243374	1.19222
8169272	NM_052936 // ATG4A // ATG4 autophagy related 4 homolog A (S. cerevisiae) // Xq22.1-q22.	ATG4A	NM_052936	0.00275795	1.19221
8146500	NM_002350 // LYN // v-yes-1 Yamaguchi sarcoma viral related oncogene homolog // 8q13 //	LYN	NM_002350	0.00168208	1.19213

8040655	NM_033505 // EPT1 // ethanolaminephosphotransferase 1 (CDP-ethanolamine-specific) // 2p	EPT1	NM_033505	0.00110669	1.1912
8151549	NM_005536 // IMPA1 // inositol(myo)-1(or 4)-monophosphatase 1 // 8q21.13-q21.3 // 3612	IMPA1	NM_005536	0.000711006	1.19046
8142687	NM_005302 // GPR37 // G protein-coupled receptor 37 (endothelin receptor type B-like) /	GPR37	NM_005302	0.000512291	1.19041
7897460	NM_032315 // SLC25A33 // solute carrier family 25, member 33 // 1p36.22 // 84275 /// EN	SLC25A33	NM_032315	0.00122839	1.19032
7949948	NM_022338 // C11orf24 // chromosome 11 open reading frame 24 // 11q13 // 53838 /// ENST	C11orf24	NM_022338	0.000107427	1.18966
8115907	NM_016391 // NOP16 // NOP16 nucleolar protein homolog (yeast) // 5q35.2 // 51491 /// EN	NOP16	NM_016391	0.00275411	1.18871
7949882	NM_000695 // ALDH3B2 // aldehyde dehydrogenase 3 family, member B2 // 11q13 // 222 ///	ALDH3B2	NM_000695	0.00281735	1.18852
8097480	NM_057175 // NAA15 // N(alpha)-acetyltransferase 15, NatA auxiliary subunit // 4q31.1 /	NAA15	NM_057175	0.00121443	1.18838
7997633	NM_005153 // USP10 // ubiquitin specific peptidase 10 // 16q24.1 // 9100 /// ENST000002	USP10	NM_005153	0.000969664	1.18792
7935027	NM_004969 // IDE // insulin-degrading enzyme // 10q23-q25 // 3416 /// NM_001165946 // I	IDE	NM_004969	0.000413919	1.18759
7935707	NM_001278 // CHUK // conserved helix-loop-helix ubiquitous kinase // 10q24-q25 // 1147	CHUK	NM_001278	0.00166027	1.18742

8053668	NM_004836 // EIF2AK3 // eukaryotic translation initiation factor 2-alpha kinase 3 // 2p	EIF2AK3	NM_004836	0.000378661	1.18701
7941621	NM_005700 // DPP3 // dipeptidyl-peptidase 3 // 11q12-q13.1 // 10072 /// NM_130443 // DP	DPP3	NM_005700	0.00161747	1.18616
7921076	NM_182679 // GPATCH4 // G patch domain containing 4 // 1q22 // 54865 /// NM_015590 // G	GPATCH4	NM_182679	0.00264915	1.18592
8051187	NM_022823 // FNDC4 // fibronectin type III domain containing 4 // 2p23.3 // 64838 /// E	FNDC4	NM_022823	0.00105881	1.18452
7919751	NM_021960 // MCL1 // myeloid cell leukemia sequence 1 (BCL2-related) // 1q21 // 4170 //	MCL1	NM_021960	0.00257406	1.18448
8114287	NM_004598 // SPOCK1 // sparc/osteonectin, cwcv and kazal-like domains proteoglycan (tes	SPOCK1	NM_004598	0.00196579	1.18359
8034712	NM_024825 // PODNL1 // podocan-like 1 // 19p13.12 // 79883 /// NM_001146254 // PODNL1 /	PODNL1	NM_024825	8.83E-05	1.18304
7998637	NM_016332 // SEPX1 // selenoprotein X, 1 // 16p13.3 // 51734 /// ENST00000361871 // SEP	SEPX1	NM_016332	0.000537927	1.18258
8013671	NM_006461 // SPAG5 // sperm associated antigen 5 // 17q11.2 // 10615 /// ENST0000032176	SPAG5	NM_006461	0.00125992	1.18221
7991581	NM_014918 // CHSY1 // chondroitin sulfate synthase 1 // 15q26.3 // 22856 /// ENST000002	CHSY1	NM_014918	0.000467817	1.18136
8108099	NM_021982 // SEC24A // SEC24 family, member A (S. cerevisiae) // 5q31.1 // 10802 /// EN	SEC24A	NM_021982	0.00097288	1.18105

8043187	NM_005911 // MAT2A // methionine adenosyltransferase II, alpha // 2p11.2 // 4144 /// EN	MAT2A	NM_005911	0.00208352	1.18047
8150276	NM_001102559 // PPAPDC1B // phosphatidic acid phosphatase type 2 domain containing 1B /	PPAPDC1B	NM_001102559	0.000976092	1.18022
7905938	NM_018845 // RAG1AP1 // recombination activating gene 1 activating protein 1 // 1q22 //	RAG1AP1	NM_018845	0.0006459	1.17931
8011245	NM_018128 // TSR1 // TSR1, 20S rRNA accumulation, homolog (S. cerevisiae) // 17p13.3 //	TSR1	NM_018128	0.0006692	1.17731
8082408	NM_013336 // SEC61A1 // Sec61 alpha 1 subunit (S. cerevisiae) // 3q21.3 // 29927 /// EN	SEC61A1	NM_013336	0.000100274	1.17729
8003611	NM_024792 // FAM57A // family with sequence similarity 57, member A // 17p13.3 // 79850	FAM57A	NM_024792	0.000815115	1.17721
8143684	NM_004911 // PDIA4 // protein disulfide isomerase family A, member 4 // 7q35 // 9601 //	PDIA4	NM_004911	0.000364122	1.17654
7969651	NM_006260 // DNAJC3 // DnaJ (Hsp40) homolog, subfamily C, member 3 // 13q32.1 // 5611 /	DNAJC3	NM_006260	0.000530695	1.17587
8154962	NM_001135004 // DNAJB5 // DnaJ (Hsp40) homolog, subfamily B, member 5 // 9p13.3 // 2582	DNAJB5	NM_001135004	0.000244173	1.17555
8073960	NM_001001852 // PIM3 // pim-3 oncogene // 22q13 // 415116 /// ENST00000360612 // PIM3 /	PIM3	NM_001001852	0.000379356	1.17554
7934753	NM_001099692 // EIF5AL1 // eukaryotic translation initiation factor 5A-like 1 // 10q22.	EIF5AL1	NM_001099692	0.000549916	1.17492

8046861	NM_002210 // ITGAV // integrin, alpha V (vitronectin receptor, alpha polypeptide, antig	ITGAV	NM_002210	0.00124514	1.17488
7905329	NM_006818 // MLLT11 // myeloid/lymphoid or mixed-lineage leukemia (trithorax homolog, D	MLLT11	NM_006818	0.00247519	1.17473
7942613	NM_032564 // DGAT2 // diacylglycerol O-acyltransferase 2 // 11q13.5 // 84649 /// ENST00	DGAT2	NM_032564	0.00270594	1.17369
7928600	NM_001099692 // EIF5AL1 // eukaryotic translation initiation factor 5A-like 1 // 10q22.	EIF5AL1	NM_001099692	0.000617598	1.17206
8089082	NM_080927 // DCBLD2 // discoidin, CUB and LCCL domain containing 2 // 3q12.1|3 // 13156	DCBLD2	NM_080927	0.00141355	1.17124
8003656	NM_001165920 // SERPINF2 // serpin peptidase inhibitor, clade F (alpha-2 antiplasmin, p	SERPINF2	NM_001165920	0.000123391	1.17078
7914563	NM_003680 // YARS // tyrosyl-tRNA synthetase // 1p35.1 // 8565 /// ENST00000373477 // Y	YARS	NM_003680	0.00181422	1.17028
7928630	NM_001099692 // EIF5AL1 // eukaryotic translation initiation factor 5A-like 1 // 10q22.	EIF5AL1	NM_001099692	0.000850779	1.16961
8118613	NM_006979 // SLC39A7 // solute carrier family 39 (zinc transporter), member 7 // 6p21.3	SLC39A7	NM_006979	0.000482625	1.16906
8178225	NM_006979 // SLC39A7 // solute carrier family 39 (zinc transporter), member 7 // 6p21.3	SLC39A7	NM_006979	0.000482625	1.16906
8179525	NM_006979 // SLC39A7 // solute carrier family 39 (zinc transporter), member 7 // 6p21.3	SLC39A7	NM_006979	0.000482625	1.16906

8058052	NM_002156 // HSPD1 // heat shock 60kDa protein 1 (chaperonin) // 2q33.1 // 3329 /// NM_	HSPD1	NM_002156	0.0017041	1.16886
8126058	NM_016059 // PPIL1 // peptidylprolyl isomerase (cyclophilin)-like 1 // 6p21.1 // 51645	PPIL1	NM_016059	0.00019838	1.16871
8115584	NM_024565 // CCNJL // cyclin J-like // 5q33.3 // 79616 /// ENST00000393977 // CCNJL //	CCNJL	NM_024565	0.000580708	1.16865
8020254	NM_031216 // SEH1L // SEH1-like (S. cerevisiae) // 18p11.21 // 81929 /// NM_001013437 /	SEH1L	NM_031216	0.00212115	1.16827
8010271	NM_024419 // PGS1 // phosphatidylglycerophosphate synthase 1 // 17q25.3 // 9489 /// ENS	PGS1	NM_024419	9.64E-05	1.16699
8095221	NM_001079525 // PAICS // phosphoribosylaminoimidazole carboxylase, phosphoribosylaminoi	PAICS	NM_001079525	0.00222501	1.16674
8017850	NM_017983 // WIPI1 // WD repeat domain, phosphoinositide interacting 1 // 17q24.2 // 55	WIPI1	NM_017983	0.00103909	1.16651
7985240	NM_007364 // TMED3 // transmembrane emp24 protein transport domain containing 3 // 15q2	TMED3	NM_007364	0.00250149	1.16506
7904254	NM_000701 // ATP1A1 // ATPase, Na+/K+ transporting, alpha 1 polypeptide // 1p21 // 476	ATP1A1	NM_000701	0.00188119	1.16412
7925823	NM_012341 // GTPBP4 // GTP binding protein 4 // 10p15-p14 // 23560 /// ENST00000360803	GTPBP4	NM_012341	0.00142255	1.1631
8114455	NM_004134 // HSPA9 // heat shock 70kDa protein 9 (mortalin) // 5q31.1 // 3313 /// ENST0	HSPA9	NM_004134	0.00020527	1.16297

8015460	NM_001096 // ACLY // ATP citrate lyase // 17q21.2 // 47 /// NM_198830 // ACLY // ATP ci	ACLY	NM_001096	0.000263509	1.16114
8088820	NM_012234 // RYBP // RING1 and YY1 binding protein // 3p13 // 23429 /// ENST00000477973	RYBP	NM_012234	0.00196475	1.1605
8112312	NM_014473 // DIMT1L // DIM1 dimethyladenosine transferase 1-like (S. cerevisiae) // 5q1	DIMT1L	NM_014473	0.00222215	1.15926
7909236	NM_004759 // MAPKAPK2 // mitogen-activated protein kinase-activated protein kinase 2 //	MAPKAPK2	NM_004759	0.00203402	1.15848
8098177	NM_007246 // KLHL2 // kelch-like 2, Mayven (Drosophila) // 4q21.2 // 11275 /// NM_00116	KLHL2	NM_007246	0.00254663	1.1583
7970513	NM_145061 // SKA3 // spindle and kinetochore associated complex subunit 3 // 13q12.11 /	SKA3	NM_145061	0.00084445	1.15807
7998233	NM_021259 // TMEM8A // transmembrane protein 8A // 16p13.3 // 58986 /// ENST00000431232	TMEM8A	NM_021259	0.00279996	1.15781
7985253	NR_028330 // C15orf37 // chromosome 15 open reading frame 37 // 15q25.1 // 283687 /// N	C15orf37	NR_028330	0.00164581	1.15647
7914648	NM_198040 // PHC2 // polyhomeotic homolog 2 (Drosophila) // 1p34.3 // 1912 /// NM_00442	PHC2	NM_198040	0.000371885	1.15555
8158424	NM_001127244 // LRRC8A // leucine rich repeat containing 8 family, member A // 9q34.11	LRRC8A	NM_001127244	0.00125407	1.15511
8110450	NM_031266 // HNRNPAB // heterogeneous nuclear ribonucleoprotein A/B // 5q35.3 // 3182 /	HNRNPAB	NM_031266	0.00132535	1.15345

8132070	NM_002047 // GARS // glycyl-tRNA synthetase // 7p15 // 2617 /// ENST00000389266 // GARS	GARS	NM_002047	0.00266274	1.14999
7991374	NM_002168 // IDH2 // isocitrate dehydrogenase 2 (NADP+), mitochondrial // 15q26.1 // 34	IDH2	NM_002168	0.000853252	1.14983
8121588	NM_013352 // DSE // dermatan sulfate epimerase // 6q22 // 29940 /// NM_001080976 // DSE	DSE	NM_013352	0.00163664	1.14974
7988260	NM_032892 // FRMD5 // FERM domain containing 5 // 15q15.3 // 84978 /// ENST00000417257	FRMD5	NM_032892	0.00222733	1.14804
8037835	NM_005628 // SLC1A5 // solute carrier family 1 (neutral amino acid transporter), member	SLC1A5	NM_005628	0.00252039	1.14336
8125775	AY568085 // C6orf125 // chromosome 6 open reading frame 125 // 6p21.31 // 84300 /// AF0	C6orf125	AY568085	0.001876	1.14189
7960124	NM_138575 // PGAM5 // phosphoglycerate mutase family member 5 // 12q24.33 // 192111 ///	PGAM5	NM_138575	0.0027593	1.13686
8048847	NM_001135187 // AGFG1 // ArfGAP with FG repeats 1 // 2q36.3 // 3267 /// NM_004504 // AG	AGFG1	NM_001135187	0.00136157	1.13405
7975224	NM_004094 // EIF2S1 // eukaryotic translation initiation factor 2, subunit 1 alpha, 35k	EIF2S1	NM_004094	0.00262917	1.13319
8045171	NM_033416 // IMP4 // IMP4, U3 small nucleolar ribonucleoprotein, homolog (yeast) // 2q2	IMP4	NM_033416	0.00260982	1.13157
8037913	NM_003827 // NAPA // N-ethylmaleimide-sensitive factor attachment protein, alpha // 19q	NAPA	NM_003827	0.00114606	1.12973

8162313	NM_013417 // IARS // isoleucyl-tRNA synthetase // 9q21 // 3376 /// NM_002161 // IARS //	IARS	NM_013417	0.00275775	1.12528
7912283	NM_020248 // CTNNBIP1 // catenin, beta interacting protein 1 // 1p36.22 // 56998 /// NM	CTNNBIP1	NM_020248	0.00119996	-1.1039
7915032	NM_001038633 // RSPO1 // R-spondin homolog (Xenopus laevis) // 1p34.3 // 284654 /// ENS	RSPO1	NM_001038633	0.00208855	-1.13817
7951429	NM_198439 // KBTBD3 // kelch repeat and BTB (POZ) domain containing 3 // 11q22.3 // 143	KBTBD3	NM_198439	0.00113744	-1.14321
8086627	NR_033815 // ALS2CL // ALS2 C-terminal like // 3p21.31 // 259173 /// NM_147129 // ALS2C	ALS2CL	NR_033815	0.00149928	-1.14434
7902102	---		---	0.000544002	-1.14858
8061529	NM_014012 // REM1 // RAS (RAD and GEM)-like GTP-binding 1 // 20q11.21 // 28954 /// ENST	REM1	NM_014012	0.00122253	-1.14996
7966839	NM_019086 // VSIG10 // V-set and immunoglobulin domain containing 10 // 12q24.23 // 546	VSIG10	NM_019086	0.00259561	-1.15018
8157905	NM_033446 // FAM125B // family with sequence similarity 125, member B // 9q33.3 // 8985	FAM125B	NM_033446	0.00177068	-1.15039
8131573	NM_212460 // ARL4A // ADP-ribosylation factor-like 4A // 7p21.3 // 10124 /// NM_0010371	ARL4A	NM_212460	0.00231416	-1.15134
7935910	NM_012215 // MGEA5 // meningioma expressed antigen 5 (hyaluronidase) // 10q24.1-q24.3 /	MGEA5	NM_012215	0.00123735	-1.15259
8166072	NM_021109 // TMSB4X // thymosin beta 4, X-linked // Xq21.3-q22 // 7114 /// NM_183049 //	TMSB4X	NM_021109	0.00224027	-1.15374

8047036	---		---	0.000986369	-1.1564
8104504	---		---	0.00112441	-1.1596
8059532	NM_020161 // C2orf83 // chromosome 2 open reading frame 83 // 2q36.3 // 56918 /// NM_00	C2orf83	NM_020161	0.00143611	-1.15968
7928367	---		---	0.00262681	-1.16015
8054766	NM_001097 // ACR // acrosin // 22q13-qter|22q13.33 // 49 /// ENST00000216139 // ACR //	ACR	NM_001097	0.0025664	-1.16046
7953341	NM_018009 // TAPBPL // TAP binding protein-like // 12p13.31 // 55080 /// NM_014231 // V	TAPBPL	NM_018009	0.00233053	-1.16133
7982131	NR_033353 // GOLGA8G // golgin A8 family, member G // 15q13.1 // 283768 /// NR_033351 /	GOLGA8G	NR_033353	0.00212581	-1.16159
7986922	NR_033353 // GOLGA8G // golgin A8 family, member G // 15q13.1 // 283768 /// NR_033351 /	GOLGA8G	NR_033353	0.00212588	-1.16159
7982230	NR_024074 // GOLGA8IP // golgin A8 family, member I (pseudogene) // 15q11.2 // 283796 /	GOLGA8IP	NR_024074	0.00196155	-1.16269
7956737	NR_029661 // MIRLET7I // microRNA let-7i // 12q14.1 // 406891	MIRLET7I	NR_029661	0.000285085	-1.16333
7979904	NM_003813 // ADAM21 // ADAM metallopeptidase domain 21 // 14q24.1 // 8747 /// NR_003951	ADAM21	NM_003813	0.00272603	-1.16464
8027674	NM_018443 // ZNF302 // zinc finger protein 302 // 19q13.11 // 55900 /// NM_001012320 //	ZNF302	NM_018443	0.0011318	-1.16485
8171066	NR_028269 // LOC100288778 // WAS protein family homolog 1 pseudogene // 12p13.33 // 100	LOC100288778	NR_028269	0.000775272	-1.16493

8109086	NM_000024 // ADRB2 // adrenergic, beta-2-, receptor, surface // 5q31-q32 // 154 /// ENS	ADRB2	NM_000024	0.00126381	-1.16731
7952426	NM_014312 // VSIG2 // V-set and immunoglobulin domain containing 2 // 11q24 // 23584 //	VSIG2	NM_014312	0.00241265	-1.16771
8007803	NR_026905 // C17orf69 // chromosome 17 open reading frame 69 // 17q21.31 // 147081 ///	C17orf69	NR_026905	0.00138587	-1.16772
7981787	NM_001001413 // GOLGA6L1 // golgin A6 family-like 1 // 15q11.2 // 283767 /// ENST000003	GOLGA6L1	NM_001001413	0.00265499	-1.16824
7991668	NR_033351 // GOLGA8F // golgin A8 family, member F // 15q13.1 // 100132565 /// NR_03335	GOLGA8F	NR_033351	0.00224728	-1.17019
8121112	---		---	0.00139825	-1.17049
7990452	---		---	0.00179816	-1.17152
8114213	---		---	0.000870328	-1.17252
8053171	NM_032779 // CCDC142 // coiled-coil domain containing 142 // 2p13.1 // 84865 /// ENST00	CCDC142	NM_032779	0.000755785	-1.17264
7980940	NR_028459 // ATXN3 // ataxin 3 // 14q21 // 4287 /// NM_004993 // ATXN3 // ataxin 3 // 1	ATXN3	NR_028459	0.0017598	-1.17297
7938388	---		---	0.00116698	-1.17301
8044643	NR_026821 // FAM138B // family with sequence similarity 138, member B // 2q14.1 // 6544	FAM138B	NR_026821	0.00137372	-1.17333
7911854	NR_033711 // KIAA0495 // KIAA0495 // 1p36.32 // 57212 /// NR_033710 // KIAA0495 // KIAA	KIAA0495	NR_033711	0.0019202	-1.17413
7932985	NM_003873 // NRP1 // neuropilin 1 // 10p12 // 8829 /// NM_001024628 // NRP1 // neuropil	NRP1	NM_003873	0.00233027	-1.17491

7971780	NM_002498 // NEK3 // NIMA (never in mitosis gene a)-related kinase 3 // 13q14.13 // 475	NEK3	NM_002498	0.00193439	-1.17706
8123520	BC118988 // NCRNA00266 // non-protein coding RNA 266 // --- // 140849	NCRNA00266	BC118988	0.00236673	-1.1773
8117685	NM_024493 // ZKSCAN3 // zinc finger with KRAB and SCAN domains 3 // 6p22.1 // 80317 ///	ZKSCAN3	NM_024493	0.00183751	-1.17798
8022434	---		---	0.00070946	-1.17943
7907788	---		---	0.000645194	-1.17982
8100603	NM_001812 // CENPC1 // centromere protein C 1 // 4q13.2 // 1060 /// ENST00000273853 //	CENPC1	NM_001812	0.00235047	-1.18192
7971191	NR_003365 // SUGT1P3 // suppressor of G2 allele of SKP1 (S. cerevisiae) pseudogene 3 //	SUGT1P3	NR_003365	0.000365534	-1.18212
7982206	NR_024074 // GOLGA8IP // golgin A8 family, member I (pseudogene) // 15q11.2 // 283796 /	GOLGA8IP	NR_024074	0.00130863	-1.1827
8090852	NM_016201 // AMOTL2 // angiomotin like 2 // 3q21-q22 // 51421 /// ENST00000249883 // AM	AMOTL2	NM_016201	0.00196371	-1.18459
7942783	BC002752 // C11orf67 // chromosome 11 open reading frame 67 // 11q14.1 // 28971 /// BC0	C11orf67	BC002752	0.000177509	-1.18763
8076185	NM_175709 // CBX7 // chromobox homolog 7 // 22q13.1 // 23492 /// ENST00000216133 // CBX	CBX7	NM_175709	0.000353075	-1.18797
7942551	---		---	0.002572	-1.18824
8163255	---		---	0.000658618	-1.18838
7971690	---		---	0.00138429	-1.18865
8074789	---		---	0.00127313	-1.18913
7962829	NR_002951 // SNORA2B // small nucleolar RNA, H/ACA box 2B // 12q13.11 // 677794	SNORA2B	NR_002951	0.00152261	-1.19033

7927305	NM_001144000 // AGAP5 // ArfGAP with GTPase domain, ankyrin repeat and PH domain 5 // 1	AGAP5	NM_001144000	0.00249648	-1.19126
8092621	NM_017541 // CRYGS // crystallin, gamma S // 3q25-qter // 1427 /// NM_001134415 // TBCC	CRYGS	NM_017541	0.000419284	-1.19193
8101701	NM_152542 // PPM1K // protein phosphatase, Mg2+/Mn2+ dependent, 1K // 4q22.1 // 152926	PPM1K	NM_152542	0.00140603	-1.19232
8103094	NM_000901 // NR3C2 // nuclear receptor subfamily 3, group C, member 2 // 4q31.1 // 4306	NR3C2	NM_000901	0.000672791	-1.19284
7981895	NR_027407 // GOLGA8DP // golgin A8 family, member D (pseudogene) // 15q11.2 // 10013297	GOLGA8DP	NR_027407	0.000479239	-1.19354
8122426	NM_014721 // PHACTR2 // phosphatase and actin regulator 2 // 6q24.2 // 9749 /// NM_0011	PHACTR2	NM_014721	0.00124323	-1.19368
8133209	NR_003666 // SPDYE7P // speedy homolog E7 (Xenopus laevis), pseudogene // 7q11.23 // 44	SPDYE7P	NR_003666	0.000563771	-1.19514
8031984	NR_026818 // FAM138A // family with sequence similarity 138, member A // 1p36.33 // 645	FAM138A	NR_026818	0.00246389	-1.19573
7911323	NR_026818 // FAM138A // family with sequence similarity 138, member A // 1p36.33 // 645	FAM138A	NR_026818	0.00246389	-1.19573
8055038	NM_017980 // LIMS2 // LIM and senescent cell antigen-like domains 2 // 2q14.3 // 55679	LIMS2	NM_017980	0.00192531	-1.19594
8031981	NR_003659 // WASH3P // WAS protein family homolog 3 pseudogene // 15q26.3 // 374666 ///	WASH3P	NR_003659	0.00079573	-1.19808

8165676	ENST00000361381 // ND4 // NADH dehydrogenase, subunit 4 (complex I) // --- // 4538 ///	ND4	ENST00000361381	0.000493013	-1.19884
8025488	NM_003451 // ZNF177 // zinc finger protein 177 // 19p13.2 // 7730 /// NM_001172650 // Z	ZNF177	NM_003451	0.00106681	-1.19977
8143772	NM_002889 // RARRES2 // retinoic acid receptor responder (tazarotene induced) 2 // 7q36	RARRES2	NM_002889	0.00264841	-1.19981
8171723	---		---	0.000194248	-1.20129
8140424	NM_175064 // SPDYE1 // speedy homolog E1 (Xenopus laevis) // 7p13 // 285955 /// NR_0036	SPDYE1	NM_175064	0.000870289	-1.2017
8104129	---		---	0.00261996	-1.20188
8141228	NM_001134450 // TMEM130 // transmembrane protein 130 // 7q22.1 // 222865 /// NM_152913	TMEM130	NM_001134450	0.00102331	-1.20193
7934384	NM_001024593 // ZMYND17 // zinc finger, MYND-type containing 17 // 10q22.2 // 118490 //	ZMYND17	NM_001024593	0.000161161	-1.20334
8159959	---		---	0.000609912	-1.20437
8107113	---		---	0.00161925	-1.20517
8135197	NM_001031618 // SPDYE2 // speedy homolog E2 (Xenopus laevis) // 7q22.1 // 441273 /// NM	SPDYE2	NM_001031618	0.00220891	-1.20593
8074734	AK128837 // LOC284861 // hypothetical LOC284861 // 22q11.21 // 284861	LOC284861	AK128837	0.0011424	-1.20677
7971671	NR_003923 // GUCY1B2 // guanylate cyclase 1, soluble, beta 2 // 13q14.3 // 2974 /// AF0	GUCY1B2	NR_003923	0.00188925	-1.20686
7979927	NM_003814 // ADAM20 // ADAM metallopeptidase domain 20 // 14q24.1 // 8748 /// ENST00000	ADAM20	NM_003814	0.0023466	-1.20708

7928882	NM_006829 // C10orf116 // chromosome 10 open reading frame 116 // 10q23.2 // 10974 ///	C10orf116	NM_006829	0.0012456	-1.20777
8136658	---		---	0.000219242	-1.20843
7974117	NM_001017923 // C14orf28 // chromosome 14 open reading frame 28 // 14q21.2 // 122525 //	C14orf28	NM_001017923	0.00173911	-1.20935
8097449	NM_032961 // PCDH10 // protocadherin 10 // 4q28.3 // 57575 /// NM_020815 // PCDH10 // p	PCDH10	NM_032961	0.000318426	-1.20963
7987114	NR_024074 // GOLGA8IP // golgin A8 family, member I (pseudogene) // 15q11.2 // 283796 /	GOLGA8IP	NR_024074	0.000655392	-1.20963
7928890	NM_133447 // AGAP11 // ankyrin repeat and GTPase domain Arf GTPase activating protein 1	AGAP11	NM_133447	0.000373816	-1.20981
7977854	NM_032876 // JUB // jub, ajuba homolog (Xenopus laevis) // 14q11.2 // 84962 /// NM_1980	JUB	NM_032876	0.0009433	-1.21029
8027292	NM_133473 // ZNF431 // zinc finger protein 431 // 19p12 // 170959 /// ENST00000311048 /	ZNF431	NM_133473	0.00235533	-1.21029
7908610	---		---	0.000365332	-1.21051
8015706	NR_024461 // LOC100190938 // hypothetical LOC100190938 // 17q21.31 // 100190938 /// NR_	LOC100190938	NR_024461	0.000298135	-1.21131
7900488	NR_029846 // MIR30E // microRNA 30e // 1p34.2 // 407034	MIR30E	NR_029846	0.00154323	-1.21197
8166096	NM_152634 // TCEANC // transcription elongation factor A (SII) N-terminal and central d	TCEANC	NM_152634	0.00225187	-1.21303
8116534	NM_032765 // TRIM52 // tripartite motif-containing 52 // 5q35.3 // 84851 /// ENST000003	TRIM52	NM_032765	0.0007489	-1.21451

8142345	NM_014705 // DOCK4 // dedicator of cytokinesis 4 // 7q31.1 // 9732 /// ENST00000437633	DOCK4	NM_014705	0.000465525	-1.21461
7900235	NM_012090 // MACF1 // microtubule-actin crosslinking factor 1 // 1p32-p31 // 23499 ///	MACF1	NM_012090	0.00263203	-1.21525
8165644	---		---	0.000543488	-1.21613
7981859	NR_024074 // GOLGA8IP // golgin A8 family, member I (pseudogene) // 15q11.2 // 283796 /	GOLGA8IP	NR_024074	0.000806008	-1.21646
8151074	NM_002603 // PDE7A // phosphodiesterase 7A // 8q13 // 5150 /// NM_002604 // PDE7A // ph	PDE7A	NM_002603	0.00018148	-1.21683
7927231	NM_133446 // AGAP4 // ArfGAP with GTPase domain, ankyrin repeat and PH domain 4 // 10q1	AGAP4	NM_133446	0.00170921	-1.21692
8021372	---		---	0.000528814	-1.21836
8035201	NM_015692 // CPAMD8 // C3 and PZP-like, alpha-2-macroglobulin domain containing 8 // 19	CPAMD8	NM_015692	0.00101312	-1.219
7990636	NR_026813 // C15orf5 // chromosome 15 open reading frame 5 // 15q23-q24 // 81698 /// AF	C15orf5	NR_026813	0.00187248	-1.21947
8151788	NM_203390 // RBM12B // RNA binding motif protein 12B // 8q22.1 // 389677 /// ENST000003	RBM12B	NM_203390	0.00103286	-1.22098
8015796	---		---	0.000960997	-1.22103
8156199	NM_004938 // DAPK1 // death-associated protein kinase 1 // 9q34.1 // 1612 /// ENST00000	DAPK1	NM_004938	0.000558787	-1.22226
8127364	NR_003660 // GUSBP4 // glucuronidase, beta pseudogene 4 // 6p11.2 // 375513 /// BC06554	GUSBP4	NR_003660	0.000572471	-1.22289
7899955	---		---	0.00174666	-1.22305

8121569	AK091822 // FLJ34503 // hypothetical FLJ34503 // 6q21 // 285759	FLJ34503	AK091822	0.00211629	-1.22321
7986947	NR_024074 // GOLGA8IP // golgin A8 family, member I (pseudogene) // 15q11.2 // 283796 /	GOLGA8IP	NR_024074	0.00126462	-1.22359
8097586	NM_207123 // GAB1 // GRB2-associated binding protein 1 // 4q31.21 // 2549 /// NM_002039	GAB1	NM_207123	0.00060385	-1.2271
7903753	NM_000848 // GSTM2 // glutathione S-transferase mu 2 (muscle) // 1p13.3 // 2946 /// NM_	GSTM2	NM_000848	0.000222689	-1.22826
8156599	---		---	0.000888334	-1.22982
8117368	NM_003542 // HIST1H4C // histone cluster 1, H4c // 6p21.3 // 8364 /// BC130558 // HIST1	HIST1H4C	NM_003542	0.00246472	-1.23219
8124691	AY358246 // HCG8 // HLA complex group 8 // 6p21.3 // 100507399	HCG8	AY358246	0.00149031	-1.2327
7987027	ENST00000450802 // GOLGA8H // golgin A8 family, member H // 15q13.2 // 728498 /// ENST0	GOLGA8H	ENST00000450802	0.000517222	-1.23333
7917304	NM_018298 // MCOLN3 // mucolipin 3 // 1p22.3 // 55283 /// ENST00000302814 // MCOLN3 //	MCOLN3	NM_018298	0.00148083	-1.23361
7967025	---		---	0.000365806	-1.23366
8159803	NR_026818 // FAM138A // family with sequence similarity 138, member A // 1p36.33 // 645	FAM138A	NR_026818	0.00259068	-1.23682
8123739	NM_016588 // NRN1 // neuritin 1 // 6p25.1 // 51299 /// ENST00000244766 // NRN1 // neuri	NRN1	NM_016588	0.00207361	-1.23729
7919390	---		---	0.00165738	-1.23767
8005549	NM_001129778 // GRAPL // GRB2-related adaptor protein-like // 17p11.2 // 400581 /// ENS	GRAPL	NM_001129778	0.00197676	-1.23893

8133654	NM_001099435 // SPDYE5 // speedy homolog E5 (Xenopus laevis) // 7q11.23 // 442590 /// N	SPDYE5	NM_001099435	0.00146411	-1.24139
7909990	AK290103 // LOC100287934 // hypothetical LOC100287934 // 1p36.33 // 100287934 /// ENST0	LOC100287934	AK290103	0.00237224	-1.24257
7934553	NM_032772 // ZNF503 // zinc finger protein 503 // 10q22.2 // 84858 /// ENST00000372524	ZNF503	NM_032772	3.67E-05	-1.2431
8156523	NR_029481 // MIRLET7D // microRNA let-7d // 9q22.32 // 406886	MIRLET7D	NR_029481	0.000285263	-1.24386
8112558	NR_003504 // GUSBL1 // glucuronidase, beta-like 1 // 6p21 // 387036 /// AK289851 // SMA	GUSBL1	NR_003504	1.46E-05	-1.24401
8112918	---		---	7.90E-05	-1.24505
8074925	NR_024448 // LOC91316 // glucuronidase, beta/immunoglobulin lambda-like polypeptide 1 p	LOC91316	NR_024448	5.31E-06	-1.24524
8151496	NM_001033723 // ZNF704 // zinc finger protein 704 // 8q21.13 // 619279 /// ENST00000327	ZNF704	NM_001033723	0.000292997	-1.24586
8136652	---		---	0.00146427	-1.2468
7929282	NM_002729 // HHEX // hematopoietically expressed homeobox // 10q23.33 // 3087 /// ENST0	HHEX	NM_002729	0.000494468	-1.2481
8111455	NR_027026 // GUSBP1 // glucuronidase, beta pseudogene 1 // 5p14.3 // 728411 /// NR_0035	GUSBP1	NR_027026	1.13E-05	-1.24855
7986741	NM_001001413 // GOLGA6L1 // golgin A6 family-like 1 // 15q11.2 // 283767 /// ENST000003	GOLGA6L1	NM_001001413	0.001095	-1.25071
8027304	NM_001076678 // ZNF493 // zinc finger protein 493 // 19p12 // 284443 /// NM_175910 // Z	ZNF493	NM_001076678	0.00125304	-1.2528

7986736	NM_001001413 // GOLGA6L1 // golgin A6 family-like 1 // 15q11.2 // 283767 /// ENST000003	GOLGA6L1	NM_001001413	0.00100231	-1.25382
8163107	NR_029506 // MIR32 // microRNA 32 // 9q31.3 // 407036	MIR32	NR_029506	0.000611869	-1.25447
8069511	NR_027270 // C21orf81 // ankyrin repeat domain 20 family, member A3 pseudogene // 21q11	C21orf81	NR_027270	0.000254543	-1.25721
7960438	NM_020373 // ANO2 // anoctamin 2 // 12p13.3 // 57101 /// ENST00000327087 // ANO2 // ano	ANO2	NM_020373	0.00200014	-1.25797
7986598	NM_001001413 // GOLGA6L1 // golgin A6 family-like 1 // 15q11.2 // 283767 /// ENST000003	GOLGA6L1	NM_001001413	0.00101181	-1.25838
8041206	NM_030915 // LBH // limb bud and heart development homolog (mouse) // 2p23.1 // 81606 /	LBH	NM_030915	0.00147143	-1.25867
8162529	---		---	0.00181488	-1.25922
8138728	NM_002141 // HOXA4 // homeobox A4 // 7p15.2 // 3201 /// ENST00000360046 // HOXA4 // hom	HOXA4	NM_002141	2.39E-05	-1.25962
8105995	NR_024054 // LOC100170939 // glucuronidase, beta pseudogene // 5q13 // 100170939 /// BC	LOC100170939	NR_024054	1.52E-05	-1.26005
8133034	---		---	0.00136042	-1.26041
8166784	NM_004615 // TSPAN7 // tetraspanin 7 // Xp11.4 // 7102 /// ENST00000378482 // TSPAN7 //	TSPAN7	NM_004615	0.000723449	-1.26044
7933310	---		---	6.59E-05	-1.26188
8161288	NM_033655 // CNTNAP3 // contactin associated protein-like 3 // 9p13.1 // 79937 /// AF33	CNTNAP3	NM_033655	0.00236832	-1.26372
7996759	---		---	0.0013406	-1.26458

8137008	AK290098 // C7orf11 // chromosome 7 open reading frame 11 // 7p14.1 // 136647	C7orf11	AK290098	0.00176603	-1.26604
8117079	BC047037 // LOC644714 // hypothetical LOC644714 // 3p21.31 // 644714	LOC644714	BC047037	0.000447258	-1.26695
8143441	NM_001080392 // KIAA1147 // KIAA1147 // 7q34 // 57189 /// ENST00000297761 // KIAA1147 /	KIAA1147	NM_001080392	6.45E-05	-1.26705
7974363	NM_000953 // PTGDR // prostaglandin D2 receptor (DP) // 14q22.1 // 5729 /// ENST0000030	PTGDR	NM_000953	0.000350748	-1.26713
8113122	---		---	0.00250198	-1.26776
7930450	---		---	0.000901486	-1.26861
8117458	NM_001145009 // BTN3A1 // butyrophilin, subfamily 3, member A1 // 6p22.1 // 11119 /// N	BTN3A1	NM_001145009	0.00139725	-1.27024
8116980	NM_001165032 // RNF182 // ring finger protein 182 // 6p23 // 221687 /// NM_152737 // RN	RNF182	NM_001165032	0.000576874	-1.27091
8136863	NM_153345 // TMEM139 // transmembrane protein 139 // 7q34 // 135932 /// ENST00000359333	TMEM139	NM_153345	0.00111814	-1.27094
8149885	NM_000680 // ADRA1A // adrenergic, alpha-1A-, receptor // 8p21.2 // 148 /// NM_033303 /	ADRA1A	NM_000680	4.05E-05	-1.27262
7940530	NM_001127392 // C11orf9 // chromosome 11 open reading frame 9 // 11q12-q13.1 // 745 ///	C11orf9	NM_001127392	0.00193206	-1.27415
7903407	NM_020978 // AMY2B // amylase, alpha 2B (pancreatic) // 1p21 // 280 /// NM_017619 // RN	AMY2B	NM_020978	0.00110653	-1.27429
7983145	NM_020759 // STARD9 // StAR-related lipid transfer (START) domain containing 9 // 15q15	STARD9	NM_020759	0.000559039	-1.27641

8155455	AK126863 // NCRNA00268 // non-protein coding RNA 268 // 9p11.2 // 441426	NCRNA00268	AK126863	0.000672996	-1.27688
7933008	---		---	0.00221542	-1.27758
7987139	NR_024074 // GOLGA8IP // golgin A8 family, member I (pseudogene) // 15q11.2 // 283796 /	GOLGA8IP	NR_024074	0.000882824	-1.27816
7982350	NR_024074 // GOLGA8IP // golgin A8 family, member I (pseudogene) // 15q11.2 // 283796 /	GOLGA8IP	NR_024074	0.000882831	-1.27816
8082465	NM_024768 // CCDC48 // coiled-coil domain containing 48 // 3q21.3 // 79825 /// AK022119	CCDC48	NM_024768	8.09E-06	-1.27922
8155460	NM_033655 // CNTNAP3 // contactin associated protein-like 3 // 9p13.1 // 79937 /// AF33	CNTNAP3	NM_033655	0.00215905	-1.27961
7989596	NM_014326 // DAPK2 // death-associated protein kinase 2 // 15q22.31 // 23604 /// ENST00	DAPK2	NM_014326	0.00276919	-1.27974
8165648	AK290098 // C7orf11 // chromosome 7 open reading frame 11 // 7p14.1 // 136647	C7orf11	AK290098	0.000389079	-1.28372
7982248	---		---	0.00281868	-1.28428
8136645	NM_016944 // TAS2R4 // taste receptor, type 2, member 4 // 7q31.3-q32 // 50832 /// ENST	TAS2R4	NM_016944	0.00157353	-1.28569
8137863	---		---	0.00125505	-1.2857
8090637	---		---	0.00138317	-1.28587
8166355	NM_014927 // CNKSR2 // connector enhancer of kinase suppressor of Ras 2 // Xp22.12 // 2	CNKSR2	NM_014927	0.000198705	-1.28679
8138718	NM_006735 // HOXA2 // homeobox A2 // 7p15.2 // 3199 /// ENST00000222718 // HOXA2 // hom	HOXA2	NM_006735	0.00229084	-1.28734

8124562	NM_001135215 // ZNF323 // zinc finger protein 323 // 6p21.31|6p22.3-p22.1 // 64288 ///	ZNF323	NM_001135215	5.78E-05	-1.28796
8102988	NM_198682 // GYPE // glycophorin E (MNS blood group) // 4q31.1 // 2996 /// NM_002102 //	GYPE	NM_198682	0.00181309	-1.28897
8112914	---		---	0.00127373	-1.29121
8089038	---		---	0.00127373	-1.29121
7951363	NM_001191016 // CASP12 // caspase 12 (gene/pseudogene) // 11q22.3 // 120329 /// NR_0340	CASP12	NM_001191016	1.31E-05	-1.29123
7955110	AK125945 // DKFZP779L1853 // hypothetical LOC643162 // 12q13.11 // 643162	DKFZP779L1853	AK125945	0.000195233	-1.29235
7961757	NM_003034 // ST8SIA1 // ST8 alpha-N-acetyl-neuraminide alpha-2,8-sialyltransferase 1 //	ST8SIA1	NM_003034	0.000330361	-1.29442
8035793	NM_001159293 // ZNF737 // zinc finger protein 737 // 19p12 // 100129842 /// ENST0000034	ZNF737	NM_001159293	0.00164034	-1.29786
7922326	NR_029627 // MIR214 // microRNA 214 // 1q24.3 // 406996 /// NR_036066 // MIR3120 // mic	MIR214	NR_029627	0.000414092	-1.29943
7946563	---		---	0.000658118	-1.29989
8177669	NR_034021 // SMA5 // glucuronidase, beta pseudogene // 5q13 // 11042 /// NR_027386 // G	SMA5	NR_034021	0.000258143	-1.30097
7982256	NR_024074 // GOLGA8IP // golgin A8 family, member I (pseudogene) // 15q11.2 // 283796 /	GOLGA8IP	NR_024074	0.000227402	-1.30365
8163063	NM_003798 // CTNNAL1 // catenin (cadherin-associated protein), alpha-like 1 // 9q31.2 /	CTNNAL1	NM_003798	0.00195159	-1.30515

8094789	NM_014988 // LIMCH1 // LIM and calponin homology domains 1 // 4p13 // 22998 /// NM_0011	LIMCH1	NM_014988	0.00219827	-1.30645
8117653	---		---	0.000504138	-1.30869
8137252	NM_130759 // GIMAP1 // GTPase, IMAP family member 1 // 7q36.1 // 170575 /// ENST0000030	GIMAP1	NM_130759	0.000446587	-1.31025
8088371	NM_004944 // DNASE1L3 // deoxyribonuclease I-like 3 // 3p14.3 // 1776 /// ENST000003183	DNASE1L3	NM_004944	1.74E-05	-1.31237
8038117	NM_001352 // DBP // D site of albumin promoter (albumin D-box) binding protein // 19q13	DBP	NM_001352	0.00015665	-1.31264
7904429	NR_033781 // HSD3BP4 // hydroxy-delta-5-steroid dehydrogenase, 3 beta, pseudogene 4 //	HSD3BP4	NR_033781	0.000382801	-1.31271
8086344	NM_001337 // CX3CR1 // chemokine (C-X3-C motif) receptor 1 // 3p21|3p21.3 // 1524 /// N	CX3CR1	NM_001337	0.00228332	-1.31314
8057797	NM_004657 // SDPR // serum deprivation response // 2q32-q33 // 8436 /// ENST00000304141	SDPR	NM_004657	0.0016277	-1.31461
7905088	NM_003517 // HIST2H2AC // histone cluster 2, H2ac // 1q21.2 // 8338 /// ENST00000331380	HIST2H2AC	NM_003517	0.000294427	-1.31618
8135594	NM_001753 // CAV1 // caveolin 1, caveolae protein, 22kDa // 7q31.1 // 857 /// NM_001172	CAV1	NM_001753	0.00253259	-1.31893
8057677	NM_014585 // SLC40A1 // solute carrier family 40 (iron-regulated transporter), member 1	SLC40A1	NM_014585	0.0022617	-1.32095
8087881	NR_029660 // MIRLET7G // microRNA let-7g // 3p21.1 // 406890	MIRLET7G	NR_029660	1.12E-05	-1.32099

8112202	NM_006622 // PLK2 // polo-like kinase 2 // 5q12.1-q13.2 // 10769 /// ENST00000274289 //	PLK2	NM_006622	0.000715885	-1.32243
8137240	NM_153236 // GIMAP7 // GTPase, IMAP family member 7 // 7q36.1 // 168537 /// ENST0000031	GIMAP7	NM_153236	0.00104675	-1.32266
7928489	---		---	0.00194641	-1.3233
8122365	NM_020455 // GPR126 // G protein-coupled receptor 126 // 6q24.1 // 57211 /// NM_0010323	GPR126	NM_020455	0.000457808	-1.32557
8105937	BT006760 // GUSBP3 // glucuronidase, beta pseudogene 3 // 5q13.2 // 653188 /// NR_02405	GUSBP3	BT006760	0.000195503	-1.33319
7906969	---		---	0.000120814	-1.3344
8112564	NR_027386 // GUSBP3 // glucuronidase, beta pseudogene 3 // 5q13.2 // 653188 /// NR_0340	GUSBP3	NR_027386	0.000533827	-1.33606
8112469	NR_027386 // GUSBP3 // glucuronidase, beta pseudogene 3 // 5q13.2 // 653188 /// NR_0340	GUSBP3	NR_027386	0.000173292	-1.33708
8177544	NR_034021 // SMA5 // glucuronidase, beta pseudogene // 5q13 // 11042 /// NR_027386 // G	SMA5	NR_034021	0.000119361	-1.33747
7918900	---		---	7.28E-07	-1.33937
7961540	NM_032918 // RERG // RAS-like, estrogen-regulated, growth inhibitor // 12p12.3 // 85004	RERG	NM_032918	0.00037538	-1.3404
7968650	NM_203451 // C13orf36 // chromosome 13 open reading frame 36 // 13q13.3 // 400120 /// E	C13orf36	NM_203451	0.000191237	-1.34051
7997504	NM_001257 // CDH13 // cadherin 13, H-cadherin (heart) // 16q23.3 // 1012 /// ENST000002	CDH13	NM_001257	0.00237167	-1.34074

8144228	AK094159 // FLJ36840 // hypothetical LOC645524 // --- // 645524	FLJ36840	AK094159	0.00263002	-1.34074
7916984	NR_029707 // MIR186 // microRNA 186 // 1p31.1 // 406962	MIR186	NR_029707	0.00182847	-1.34626
7979412	---		---	0.00139894	-1.35063
7903777	NM_000851 // GSTM5 // glutathione S-transferase mu 5 // 1p13.3 // 2949 /// ENST00000256	GSTM5	NM_000851	0.000197269	-1.35154
8091537	NM_178822 // IGSF10 // immunoglobulin superfamily, member 10 // 3q25.1 // 285313 /// NM	IGSF10	NM_178822	0.000572713	-1.35522
8109157	NR_029684 // MIR143 // microRNA 143 // 5q32 // 406935 /// NR_027180 // LOC728264 // hyp	MIR143	NR_029684	0.000311924	-1.35846
8088634	---		---	0.00128471	-1.36194
8111677	NM_002310 // LIFR // leukemia inhibitory factor receptor alpha // 5p13-p12 // 3977 ///	LIFR	NM_002310	0.00170455	-1.3666
8175234	NM_001164617 // GPC3 // glypican 3 // Xq26.1 // 2719 /// NM_004484 // GPC3 // glypican	GPC3	NM_001164617	0.00107851	-1.37537
8143781	NM_024711 // GIMAP6 // GTPase, IMAP family member 6 // --- // 474344 /// NR_024115 // G	GIMAP6	NM_024711	0.000174634	-1.3799
8141342	NM_000765 // CYP3A7 // cytochrome P450, family 3, subfamily A, polypeptide 7 // 7q21-q2	CYP3A7	NM_000765	0.000160997	-1.38201
8086538	BC047037 // LOC644714 // hypothetical LOC644714 // 3p21.31 // 644714	LOC644714	BC047037	0.000301208	-1.38449
8124469	NR_034021 // SMA5 // glucuronidase, beta pseudogene // 5q13 // 11042 /// NR_027386 // G	SMA5	NR_034021	1.01E-05	-1.38476

8112491	NR_034021 // SMA5 // glucuronidase, beta pseudogene // 5q13 // 11042 /// NR_027386 // G	SMA5	NR_034021	3.51E-05	-1.38495
7983132	NM_020759 // STARD9 // StAR-related lipid transfer (START) domain containing 9 // 15q15	STARD9	NM_020759	0.000276754	-1.3857
8165653	ENST00000361390 // ND1 // NADH dehydrogenase, subunit 1 (complex I) // --- // 4535 ///	ND1	ENST00000361390	0.00146282	-1.38639
7968004	NM_000231 // SGCG // sarcoglycan, gamma (35kDa dystrophin-associated glycoprotein) // 1	SGCG	NM_000231	6.37E-05	-1.38687
8101881	NM_000668 // ADH1B // alcohol dehydrogenase 1B (class I), beta polypeptide // 4q23 // 1	ADH1B	NM_000668	0.000863255	-1.39332
8046099	NM_001039724 // NOSTRIN // nitric oxide synthase trafficker // 2q31.1 // 115677 /// NM_	NOSTRIN	NM_001039724	0.00206649	-1.39942
8175683	NR_029638 // MIR224 // microRNA 224 // Xq28 // 407009 /// U92285 // GABRE // gamma-amin	MIR224	NR_029638	0.00087617	-1.40278
7983143	NM_020759 // STARD9 // StAR-related lipid transfer (START) domain containing 9 // 15q15	STARD9	NM_020759	0.000138456	-1.4031
8111925	NM_001014279 // C5orf39 // chromosome 5 open reading frame 39 // 5p12 // 389289 /// ENS	C5orf39	NM_001014279	1.01E-05	-1.40342
7919139	AK090412 // LOC375010 // ankyrin repeat domain 20 family, member A pseudogene // 1q21.1	LOC375010	AK090412	0.000186737	-1.4035
8094301	NM_004787 // SLIT2 // slit homolog 2 (Drosophila) // 4p15.2 // 9353 /// ENST00000504154	SLIT2	NM_004787	0.00205865	-1.40667

7964872	NM_001109754 // PTPRB // protein tyrosine phosphatase, receptor type, B // 12q15-q21 //	PTPRB	NM_001109754	0.00252271	-1.40996
7972215	---		---	6.05E-05	-1.42772
7988342	---		---	0.000679539	-1.42818
8165658	ENST00000361453 // ND2 // MTND2 // --- // 4536	ND2	ENST00000361453	0.000641454	-1.42874
8087925	NM_003280 // TNNC1 // troponin C type 1 (slow) // 3p21.1 // 7134 /// ENST00000232975 //	TNNC1	NM_003280	0.000517665	-1.4319
8041644	NM_172069 // PLEKHH2 // pleckstrin homology domain containing, family H (with MyTH4 dom	PLEKHH2	NM_172069	0.00153287	-1.43233
7923976	NR_029518 // MIR29B2 // microRNA 29b-2 // 1q32.2 // 407025	MIR29B2	NR_029518	0.000564736	-1.43956
8089467	NM_024508 // ZBED2 // zinc finger, BED-type containing 2 // 3q13.2 // 79413 /// ENST000	ZBED2	NM_024508	0.000208224	-1.44717
8156521	NR_029483 // MIRLET7F1 // microRNA let-7f-1 // 9q22.32 // 406888	MIRLET7F1	NR_029483	0.000263255	-1.45104
7926979	---		---	1.18E-05	-1.45461
8042038	NR_002229 // RPL23AP32 // ribosomal protein L23a pseudogene 32 // 2p16.2 // 56969	RPL23AP32	NR_002229	0.00128559	-1.45625
8127502	NR_026807 // C6orf155 // chromosome 6 open reading frame 155 // 6q13 // 79940	C6orf155	NR_026807	0.00101973	-1.46551
8067944	NR_029480 // MIRLET7C // microRNA let-7c // 21q21.1 // 406885	MIRLET7C	NR_029480	3.99E-07	-1.47131
8067942	NR_029514 // MIR99A // microRNA 99a // 21q21.1 // 407055	MIR99A	NR_029514	1.17E-05	-1.47261
7992893	---		---	0.000967816	-1.47479
7919146	NR_003366 // ANKRD20B // ankyrin repeat domain 20B // 2q11.1 // 729171 /// AK090412 //	ANKRD20B	NR_003366	8.88E-05	-1.48255

8138735	NM_019102 // HOXA5 // homeobox A5 // 7p15.2 // 3202 /// ENST00000222726 // HOXA5 // hom	HOXA5	NM_019102	0.00113323	-1.4833
7963534	NM_002272 // KRT4 // keratin 4 // 12q12-q13 // 3851 /// ENST00000293774 // KRT4 // kera	KRT4	NM_002272	0.000744567	-1.48394
8121416	---		---	0.000562852	-1.48771
7944765	---		---	1.80E-05	-1.49345
8171248	NM_000216 // KAL1 // Kallmann syndrome 1 sequence // Xp22.32 // 3730 /// ENST0000026264	KAL1	NM_000216	0.000767844	-1.49571
8129037	---		---	0.000384933	-1.4974
8147988	---		---	0.00168421	-1.51568
8149243	NM_201402 // USP17L2 // ubiquitin specific peptidase 17-like 2 // 8p23.1 // 377630 ///	USP17L2	NM_201402	0.00153132	-1.51732
8113120	BC028919 // TOB2 // transducer of ERBB2, 2 // 22q13.2 // 10766	TOB2	BC028919	0.000131325	-1.52063
8165707	BC028919 // TOB2 // transducer of ERBB2, 2 // 22q13.2 // 10766	TOB2	BC028919	0.000131325	-1.52063
8101284	NM_006259 // PRKG2 // protein kinase, cGMP-dependent, type II // 4q13.1-q21.1 // 5593 /	PRKG2	NM_006259	1.64E-05	-1.52324
7932407	NM_001004470 // ST8SIA6 // ST8 alpha-N-acetyl-neuraminide alpha-2,8-sialyltransferase 6	ST8SIA6	NM_001004470	0.00257697	-1.52421
8144395	NM_201402 // USP17L2 // ubiquitin specific peptidase 17-like 2 // 8p23.1 // 377630 ///	USP17L2	NM_201402	0.00168663	-1.52593
8155734	NM_004816 // FAM189A2 // family with sequence similarity 189, member A2 // 9q21.11 // 9	FAM189A2	NM_004816	4.02E-05	-1.53633
8094340	NR_029631 // MIR218-1 // microRNA 218-1 // 4p15.31 // 407000	MIR218-1	NR_029631	3.79E-05	-1.53922

8149356	NM_201402 // USP17L2 // ubiquitin specific peptidase 17-like 2 // 8p23.1 // 377630 ///	USP17L2	NM_201402	0.00209629	-1.53952
8127498	NR_029598 // MIR30C2 // microRNA 30c-2 // 6q13 // 407032	MIR30C2	NR_029598	0.000101551	-1.54333
8043375	---		---	0.00100633	-1.54551
8108180	---		---	0.00014242	-1.54752
8133038	---		---	0.00134419	-1.5593
8136654	---		---	0.000685591	-1.56145
8165667	---		---	0.00110894	-1.56199
7896752	---		---	0.00110894	-1.56199
8109159	NR_029686 // MIR145 // microRNA 145 // 5q32 // 406937 /// NR_027180 // LOC728264 // hyp	MIR145	NR_029686	0.00204181	-1.56388
7904478	AF172850 // LOC51152 // melanoma antigen // --- // 51152	LOC51152	AF172850	0.00179087	-1.56981
8077299	NM_014461 // CNTN6 // contactin 6 // 3p26-p25 // 27255 /// ENST00000446702 // CNTN6 //	CNTN6	NM_014461	1.17E-05	-1.57065
8099537	---		---	0.000218124	-1.57115
7981960	NR_003321 // SNORD116-6 // small nucleolar RNA, C/D box 116-6 // 15q11.2 // 100033418	SNORD116-6	NR_003321	0.000264796	-1.59251
8055323	NM_207363 // NCKAP5 // NCK-associated protein 5 // 2q21.2 // 344148 /// NM_207481 // NC	NCKAP5	NM_207363	0.00210188	-1.60007
8018006	NM_080282 // ABCA10 // ATP-binding cassette, sub-family A (ABC1), member 10 // 17q24 //	ABCA10	NM_080282	0.000590666	-1.61571
7911341	---		---	3.87E-05	-1.62143
7923974	NR_029832 // MIR29C // microRNA 29c // 1q32.2 // 407026	MIR29C	NR_029832	3.14E-05	-1.63999
8114211	---		---	0.000112024	-1.65798
8107204	---		---	0.000115269	-1.65924

8156569	NR_029664 // MIR23B // microRNA 23b // 9q22.32 // 407011 /// AF043897 // C9orf3 // chro	MIR23B	NR_029664	2.98E-06	-1.66114
8165700	---		---	4.70E-05	-1.6683
7965110	---		---	4.37E-05	-1.67135
8013521	---		---	0.00170832	-1.67368
8071049	AF172850 // LOC51152 // melanoma antigen // --- // 51152	LOC51152	AF172850	0.000377644	-1.67944
7971661	NR_029485 // MIR15A // microRNA 15a // 13q14.2 // 406948	MIR15A	NR_029485	0.000412491	-1.68069
8045287	---		---	0.00219245	-1.68553
8165703	AF284753 // UIMC1 // ubiquitin interaction motif containing 1 // 5q35.2 // 51720	UIMC1	AF284753	0.000210481	-1.68794
7911343	AF284753 // UIMC1 // ubiquitin interaction motif containing 1 // 5q35.2 // 51720	UIMC1	AF284753	0.000210481	-1.68794
8165682	---		---	7.20E-05	-1.6901
8043502	---		---	0.000207601	-1.71407
8156571	NR_029665 // MIR27B // microRNA 27b // 9q22.32 // 407019 /// AF043897 // C9orf3 // chro	MIR27B	NR_029665	2.24E-05	-1.72647
8179967	NM_001136 // AGER // advanced glycosylation end product-specific receptor // 6p21.3 //	AGER	NM_001136	0.00229807	-1.73509
8144397	NM_201402 // USP17L2 // ubiquitin specific peptidase 17-like 2 // 8p23.1 // 377630 ///	USP17L2	NM_201402	0.00202084	-1.73752
8149241	NM_201402 // USP17L2 // ubiquitin specific peptidase 17-like 2 // 8p23.1 // 377630 ///	USP17L2	NM_201402	0.00202084	-1.73752
7973867	---		---	0.000223817	-1.74462
8165680	---		---	0.000120495	-1.74861
8125341	NM_001136 // AGER // advanced glycosylation end product-specific receptor // 6p21.3 //	AGER	NM_001136	0.00259716	-1.76971

8165709	---		---	0.000582574	-1.77501
8178771	NM_001136 // AGER // advanced glycosylation end product-specific receptor // 6p21.3 //	AGER	NM_001136	0.00246913	-1.77918
8109383	NM_000827 // GRIA1 // glutamate receptor, ionotropic, AMPA 1 // 5q33|5q31.1 // 2890 ///	GRIA1	NM_000827	3.40E-05	-1.80799
8165698	---		---	0.000774162	-1.84644
7911339	---		---	0.000774162	-1.84644
8171427	NM_004469 // FIGF // c-fos induced growth factor (vascular endothelial growth factor D)	FIGF	NM_004469	0.00109922	-1.89623
8165694	---		---	0.000897648	-1.90906
7911335	---		---	0.000897648	-1.90906
7896748	---		---	0.000990859	-1.97715
8100310	---		---	0.00250531	-1.99116
8165684	---		---	0.00025094	-2.13362
7933855	NM_145307 // RTKN2 // rhotekin 2 // 10q21.2 // 219790 /// ENST00000373789 // RTKN2 // r	RTKN2	NM_145307	0.00160748	-2.33775

Supplemental Table S5. Genes whose expression correlates with percent predicted FVC in all IIPs at 5%FDR.

Transcript ID	RefSeq	Gene Symbol	gene_assignment	p-value(FVC)	PartialCorr(FVC)
8105267	NM_002203	ITGA2	NM_002203 // ITGA2 // integrin, alpha 2 (CD49B, alpha 2 subunit of VLA-2 receptor) // 5	0.00369018	-0.197338
7921702	NM_080878	ITLN2	NM_080878 // ITLN2 // intelectin 2 // 1q22-q23 // 142683 /// ENST00000368029 // ITLN2 /	8.31E-05	0.260314
7940530	NM_001127392	C11orf9	NM_001127392 // C11orf9 // chromosome 11 open reading frame 9 // 11q12-q13.1 // 745 ///	5.23E-05	0.276638
8008900	NM_000717	CA4	NM_000717 // CA4 // carbonic anhydrase IV // 17q23 // 762 /// ENST00000300900 // CA4 //	0.0016196	0.20888
7933298	NM_031912	SYT15	NM_031912 // SYT15 // synaptotagmin XV // 10q11.1 // 83849 /// NM_181519 // SYT15 // sy	0.00736955	0.184648
8045776	NM_052917	GALNT13	NM_052917 // GALNT13 // UDP-N-acetyl-alpha-D-galactosamine:polypeptide N-acetylgalactos	0.00201618	0.206387
7966839	NM_019086	VSIG10	NM_019086 // VSIG10 // V-set and immunoglobulin domain containing 10 // 12q24.23 // 546	0.00053743	0.235365
8010271	NM_024419	PGS1	NM_024419 // PGS1 // phosphatidylglycerophosphate synthase 1 // 17q25.3 // 9489 /// ENS	7.50E-06	-0.321332
7968650	NM_203451	C13orf36	NM_203451 // C13orf36 // chromosome 13 open reading frame 36 // 13q13.3 // 400120 /// E	8.45E-07	0.322796
8062571	NM_030919	FAM83D	NM_030919 // FAM83D // family with sequence similarity 83, member D // 20q11.22-q12 //	0.0002652	-0.251945
7924996	NM_032800	C1orf198	NM_032800 // C1orf198 // chromosome 1 open reading frame 198 // 1q42.2 // 84886 /// NM_	0.009483	0.183545
8123678	NM_183373	C6orf145	NM_183373 // C6orf145 // chromosome 6 open reading frame 145 // 6p25.2 // 221749 /// EN	0.00242834	-0.221953
8133741	NM_030570	UPK3B	NM_030570 // UPK3B // uroplakin 3B // 7q11.2 // 80761 /// NM_182684 // UPK3B // uroplak	0.00248763	0.210199
8091556	---		---	0.0101193	-0.188035
7996819	NM_001793	CDH3	NM_001793 // CDH3 // cadherin 3, type 1, P-cadherin (placental) // 16q22.1 // 1001 ///	0.000666658	-0.234294

8163637	NM_002160	TNC	NM_002160 // TNC // tenascin C // 9q33 // 3371 /// ENST00000350763 // TNC // tenascin C	0.00170088	-0.214326
8003667	NM_002615	SERPINF1	NM_002615 // SERPINF1 // serpin peptidase inhibitor, clade F (alpha-2 antiplasmin, pigm	0.000576022	-0.232585
7950473	NM_004041	ARRB1	NM_004041 // ARRB1 // arrestin, beta 1 // 11q13 // 408 /// NM_020251 // ARRB1 // arrest	0.000558788	0.234087
8076128	NM_014876	JOSD1	NM_014876 // JOSD1 // Josephin domain containing 1 // 22q13.1 // 9929 /// ENST000002160	0.000431497	-0.250614
7927277	NM_014696	GPRIN2	NM_014696 // GPRIN2 // G protein regulated inducer of neurite outgrowth 2 // 10q11.22 /	0.00143013	0.224834
8020551	NM_198129	LAMA3	NM_198129 // LAMA3 // laminin, alpha 3 // 18q11.2 // 3909 /// NM_001127717 // LAMA3 //	0.0046785	0.199769
8040587	NM_014971	EFR3B	NM_014971 // EFR3B // EFR3 homolog B (S. cerevisiae) // 2p23.3 // 22979 /// ENST0000040	0.00919116	0.181171
8151816	NM_005261	GEM	NM_005261 // GEM // GTP binding protein overexpressed in skeletal muscle // 8q13-q21 //	0.0011739	-0.226771
8097335	NM_014278	HSPA4L	NM_014278 // HSPA4L // heat shock 70kDa protein 4-like // 4q28 // 22824 /// ENST0000029	0.00345442	-0.203092
7951363	NM_001191016	CASP12	NM_001191016 // CASP12 // caspase 12 (gene/pseudogene) // 11q22.3 // 120329 /// NR_0340	7.42E-10	0.405808
7989596	NM_014326	DAPK2	NM_014326 // DAPK2 // death-associated protein kinase 2 // 15q22.31 // 23604 /// ENST00	0.00147757	0.22495
7904293	NM_020440	PTGFRN	NM_020440 // PTGFRN // prostaglandin F2 receptor negative regulator // 1p13.1 // 5738 /	4.71E-05	-0.280953
8087925	NM_003280	TNNC1	NM_003280 // TNNC1 // troponin C type 1 (slow) // 3p21.1 // 7134 /// ENST00000232975 //	0.000254426	0.256596
7986092	NM_002569	FURIN	NM_002569 // FURIN // furin (paired basic amino acid cleaving enzyme) // 15q26.1 // 504	0.000106407	-0.278992
8022559	NM_173505	ANKRD29	NM_173505 // ANKRD29 // ankyrin repeat domain 29 // 18q11.2 // 147463 /// ENST000003229	0.000214729	0.25997

8148304	NM_025195	TRIB1	NM_025195 // TRIB1 // tribbles homolog 1 (Drosophila) // 8q24.13 // 10221 /// ENST00000	0.000110732	-0.284949
8092726	NM_021101	CLDN1	NM_021101 // CLDN1 // claudin 1 // 3q28-q29 // 9076 /// ENST00000295522 // CLDN1 // cla	0.000547666	-0.240858
8033002	NM_004793	LONP1	NM_004793 // LONP1 // lon peptidase 1, mitochondrial // 19p13.2 // 9361 /// ENST0000036	0.000141344	-0.272892
8044882	NM_020909	EPB41L5	NM_020909 // EPB41L5 // erythrocyte membrane protein band 4.1 like 5 // 2q14.2 // 57669	0.000649705	0.23845
8126839	NM_014452	TNFRSF21	NM_014452 // TNFRSF21 // tumor necrosis factor receptor superfamily, member 21 // 6p21.	1.65E-05	-0.297406
8147777	NM_138455	CTHRC1	NM_138455 // CTHRC1 // collagen triple helix repeat containing 1 // 8q22.3 // 115908 //	0.00383421	-0.204231
8103789	NM_005277	GPM6A	NM_005277 // GPM6A // glycoprotein M6A // 4q34 // 2823 /// NM_201591 // GPM6A // glycop	0.000213525	0.26379
7989073	NM_173814	PRTG	NM_173814 // PRTG // protogenin // 15q21.3 // 283659 /// ENST00000389286 // PRTG // pro	0.0062612	0.191111
7956658	NM_004731	SLC16A7	NM_004731 // SLC16A7 // solute carrier family 16, member 7 (monocarboxylic acid transpo	0.000621932	-0.239495
8046861	NM_002210	ITGAV	NM_002210 // ITGAV // integrin, alpha V (vitronectin receptor, alpha polypeptide, antig	3.07E-05	-0.28759
8088106	NM_001135055	TKT	NM_001135055 // TKT // transketolase // 3p14.3 // 7086 /// NM_001064 // TKT // transket	0.00077497	-0.237439
7913667	NM_000403	GALE	NM_000403 // GALE // UDP-galactose-4-epimerase // 1p36-p35 // 2582 /// NM_001008216 //	0.00112152	-0.237949
8128472	NM_006828	ASCC3	NM_006828 // ASCC3 // activating signal cointegrator 1 complex subunit 3 // 6q16 // 109	0.00434685	-0.203427
8130867	NM_003247	THBS2	NM_003247 // THBS2 // thrombospondin 2 // 6q27 // 7058 /// ENST00000366787 // THBS2 //	0.000228715	-0.258246
8110932	NM_003966	SEMA5A	NM_003966 // SEMA5A // sema domain, seven thrombospondin repeats (type 1 and type 1-lik	0.000116286	0.274825

8112841	NM_004272	HOMER1	NM_004272 // HOMER1 // homer homolog 1 (Drosophila) // 5q14.2 // 9456 /// ENST000003340	0.0029891	-0.220486
8079153	NM_016006	ABHD5	NM_016006 // ABHD5 // abhydrolase domain containing 5 // 3p21 // 51099 /// ENST00000458	0.00377364	-0.211634
8079966	NM_004636	SEMA3B	NM_004636 // SEMA3B // sema domain, immunoglobulin domain (Ig), short basic domain, sec	0.00468377	0.205693
8111892	NM_000436	OXCT1	NM_000436 // OXCT1 // 3-oxoacid CoA transferase 1 // 5p13.1 // 5019 /// ENST00000196371	0.00149165	-0.225772
7952426	NM_014312	VSIG2	NM_014312 // VSIG2 // V-set and immunoglobulin domain containing 2 // 11q24 // 23584 //	0.00980403	0.188754
7926037	NM_004566	PFKFB3	NM_004566 // PFKFB3 // 6-phosphofructo-2-kinase/fructose-2,6-biphosphatase 3 // 10p15.1	0.000215047	-0.2717
8035793	NM_001159293	ZNF737	NM_001159293 // ZNF737 // zinc finger protein 737 // 19p12 // 100129842 /// ENST0000034	0.000396977	0.264356
8025828	NM_000527	LDLR	NM_000527 // LDLR // low density lipoprotein receptor // 19p13.3 // 3949 /// NM_0011957	0.00582831	-0.204167
8156043	NM_058179	PSAT1	NM_058179 // PSAT1 // phosphoserine aminotransferase 1 // 9q21.2 // 29968 /// NM_021154	2.15E-06	-0.32751
7909164	NM_001910	CTSE	NM_001910 // CTSE // cathepsin E // 1q31 // 1510 /// NM_148964 // CTSE // cathepsin E /	0.0042124	-0.202573
8054377	NM_201555	FHL2	NM_201555 // FHL2 // four and a half LIM domains 2 // 2q12.2 // 2274 /// NM_001450 // F	0.00350028	-0.201133
8060660	NM_052970	HSPA12B	NM_052970 // HSPA12B // heat shock 70kD protein 12B // 20p13 // 116835 /// NM_001197327	0.000828394	0.238914
7972215	---		---	1.61E-06	0.347466
8158147	NM_052901	SLC25A25	NM_052901 // SLC25A25 // solute carrier family 25 (mitochondrial carrier; phosphate car	0.000130578	-0.282081

8097841	NM_015271	TRIM2	NM_015271 // TRIM2 // tripartite motif-containing 2 // 4q31.3 // 23321 /// NM_001130067	0.00595182	-0.196797
7912157	NM_018948	ERRFI1	NM_018948 // ERRFI1 // ERBB receptor feedback inhibitor 1 // 1p36 // 54206 /// ENST0000	6.86E-06	-0.32964
8080578	NM_018398	CACNA2D3	NM_018398 // CACNA2D3 // calcium channel, voltage-dependent, alpha 2/delta subunit 3 //	0.000921694	0.237106
8171427	NM_004469	FIGF	NM_004469 // FIGF // c-fos induced growth factor (vascular endothelial growth factor D)	0.000108493	0.277729
8036890	NM_020956	PRX	NM_020956 // PRX // periaxin // 19q13.2 // 57716 /// NM_181882 // PRX // periaxin // 19	0.00584253	0.196595
7983890	NM_001018100	GCOM1	NM_001018100 // GCOM1 // GRINL1A complex locus // 15q21.3 // 145781 /// NM_152451 // GC	0.00445526	0.206431
7958644	NM_170665	ATP2A2	NM_170665 // ATP2A2 // ATPase, Ca++ transporting, cardiac muscle, slow twitch 2 // 12q2	4.51E-05	-0.2868
8136652	---		---	0.000457874	0.245644
7992463	NM_001130012	SLC9A3R2	NM_001130012 // SLC9A3R2 // solute carrier family 9 (sodium/hydrogen exchanger), member	0.00253565	0.217025
8144228	AK094159	FLJ36840	AK094159 // FLJ36840 // hypothetical LOC645524 // --- // 645524	0.000336956	0.26095
8104758	BC022250	C5orf23	BC022250 // C5orf23 // chromosome 5 open reading frame 23 // 5p13.3 // 79614 /// ENST00	0.00152173	0.236782
8171418	NM_002641	PIGA	NM_002641 // PIGA // phosphatidylinositol glycan anchor biosynthesis, class A // Xp22.1	0.0066954	-0.203225
8079334	NM_014240	LIMD1	NM_014240 // LIMD1 // LIM domains containing 1 // 3p21.3 // 8994 /// ENST00000273317 //	0.00540803	0.201656
7962884	NM_014470	RND1	NM_014470 // RND1 // Rho family GTPase 1 // 12q12 // 27289 /// ENST00000309739 // RND1	3.19E-05	-0.306281
8178771	NM_001136	AGER	NM_001136 // AGER // advanced glycosylation end product-specific receptor // 6p21.3 //	0.000942621	0.236232

8179967	NM_001136	AGER	NM_001136 // AGER // advanced glycosylation end product-specific receptor // 6p21.3 //	0.000806612	0.23911
8125341	NM_001136	AGER	NM_001136 // AGER // advanced glycosylation end product-specific receptor // 6p21.3 //	0.000984304	0.235468
7980616	NM_007039	PTPN21	NM_007039 // PTPN21 // protein tyrosine phosphatase, non-receptor type 21 // 14q31.3 //	0.000365869	0.257666
8133034	---		---	0.000326952	0.251184
8098924	NM_012318	LETM1	NM_012318 // LETM1 // leucine zipper-EF-hand containing transmembrane protein 1 // 4p16	7.61E-05	-0.288633
7935230	NM_002860	ALDH18A1	NM_002860 // ALDH18A1 // aldehyde dehydrogenase 18 family, member A1 // 10q24.3 // 5832	1.81E-07	-0.352394
7899394	NM_001105556	C1orf38	NM_001105556 // C1orf38 // chromosome 1 open reading frame 38 // 1p35.3 // 9473 /// NM_	0.00214273	-0.221562
8174598	NM_000640	IL13RA2	NM_000640 // IL13RA2 // interleukin 13 receptor, alpha 2 // Xq13.1-q28 // 3598 /// ENST	0.00148832	-0.223468
8129390	---		---	0.00957142	0.194564
8095986	NM_005139	ANXA3	NM_005139 // ANXA3 // annexin A3 // 4q21.21 // 306 /// ENST00000264908 // ANXA3 // anne	0.00741138	0.195943
8034851	NM_032571	EMR3	NM_032571 // EMR3 // egf-like module containing, mucin-like, hormone receptor-like 3 //	0.00265223	-0.220299
8057797	NM_004657	SDPR	NM_004657 // SDPR // serum deprivation response // 2q32-q33 // 8436 /// ENST00000304141	0.000163912	0.270071
7914950	NM_156039	CSF3R	NM_156039 // CSF3R // colony stimulating factor 3 receptor (granulocyte) // 1p35-p34.3	0.00139436	-0.236789
8117653	---		---	0.000227334	0.271807
8152215	NM_005655	KLF10	NM_005655 // KLF10 // Kruppel-like factor 10 // 8q22.2 // 7071 /// NM_001032282 // KLF1	0.00504978	-0.209729
7954527	NM_020183	ARNTL2	NM_020183 // ARNTL2 // aryl hydrocarbon receptor nuclear translocator-like 2 // 12p12.2	6.80E-05	-0.280456

8105348	NM_001008397	GPX8	NM_001008397 // GPX8 // glutathione peroxidase 8 (putative) // 5q11.2 // 493869 /// ENS	0.00301576	-0.210256
7952268	NM_006288	THY1	NM_006288 // THY1 // Thy-1 cell surface antigen // 11q23.3 // 7070 /// ENST00000284240	0.000228672	-0.262641
8017867	NM_017565	FAM20A	NM_017565 // FAM20A // family with sequence similarity 20, member A // 17q24.2 // 54757	0.000116054	-0.290371
8178598	NM_001288	CLIC1	NM_001288 // CLIC1 // chloride intracellular channel 1 // 6p21.3 // 1192 /// ENST000004	0.00823507	-0.198362
8136645	NM_016944	TAS2R4	NM_016944 // TAS2R4 // taste receptor, type 2, member 4 // 7q31.3-q32 // 50832 /// ENST	0.00125674	0.236383
7917199	NM_024686	TTLL7	NM_024686 // TTLL7 // tubulin tyrosine ligase-like family, member 7 // 1p31.1 // 79739	0.00119944	0.23497
8086330	NM_033027	CSRNP1	NM_033027 // CSRNP1 // cysteine-serine-rich nuclear protein 1 // 3p22 // 64651 /// ENST	0.00616784	-0.207953
7959205	NM_014730	MLEC	NM_014730 // MLEC // malectin // 12q24.31 // 9761 /// ENST00000228506 // MLEC // malect	3.72E-05	-0.299364
8018449	NM_199242	UNC13D	NM_199242 // UNC13D // unc-13 homolog D (C. elegans) // 17q25.1 // 201294 /// ENST00000	0.00897751	0.184859
8081288	NM_018004	TMEM45A	NM_018004 // TMEM45A // transmembrane protein 45A // 3q12.2 // 55076 /// ENST0000032352	0.00590925	-0.196869
8150076	NM_001394	DUSP4	NM_001394 // DUSP4 // dual specificity phosphatase 4 // 8p12-p11 // 1846 /// NM_057158	0.000219243	-0.275334
8179704	NM_003897	IER3	NM_003897 // IER3 // immediate early response 3 // 6p21.3 // 8870 /// ENST00000259874 /	0.00222168	-0.226873
7994756	NM_002720	PPP4C	NM_002720 // PPP4C // protein phosphatase 4, catalytic subunit // 16p11.2 // 5531 /// E	0.00656803	-0.198714
7951217	NM_002423	MMP7	NM_002423 // MMP7 // matrix metallopeptidase 7 (matrilysin, uterine) // 11q21-q22 // 43	0.00286751	-0.216355

8161701	NM_013390	TMEM2	NM_013390 // TMEM2 // transmembrane protein 2 // 9q13-q21 // 23670 /// NM_001135820 //	0.00212514	-0.23118
7900365	NM_001136493	MFSD2A	NM_001136493 // MFSD2A // major facilitator superfamily domain containing 2A // 1p34.2	0.00554774	-0.208375
7977615	NM_198232	RNASE1	NM_198232 // RNASE1 // ribonuclease, RNase A family, 1 (pancreatic) // 14q11.2 // 6035	0.00172594	-0.234407
8126729	NM_001114086	CLIC5	NM_001114086 // CLIC5 // chloride intracellular channel 5 // 6p12.3 // 53405 /// NM_016	0.000450178	0.252354
8102592	---		---	0.000807523	0.251618
8084717	NM_173216	ST6GAL1	NM_173216 // ST6GAL1 // ST6 beta-galactosamide alpha-2,6-sialyltranferase 1 // 3q27-q28	0.00158273	-0.228237
8167973	NM_138737	HEPH	NM_138737 // HEPH // hephaestin // Xq11-q12 // 9843 /// NM_001130860 // HEPH // hephaes	0.00949127	-0.185914
8045339	---		---	0.00102199	0.240954
8141328	NR_033807	CYP3A5	NR_033807 // CYP3A5 // cytochrome P450, family 3, subfamily A, polypeptide 5 // 7q21.1	0.00027951	0.267341
7920291	NM_080388	S100A16	NM_080388 // S100A16 // S100 calcium binding protein A16 // 1q21 // 140576 /// ENST0000	0.0070458	-0.202965
8157804	NM_182487	OLFML2A	NM_182487 // OLFML2A // olfactomedin-like 2A // 9q33.3 // 169611 /// ENST00000373580 //	0.000948321	0.238726
7960865	NM_006931	SLC2A3	NM_006931 // SLC2A3 // solute carrier family 2 (facilitated glucose transporter), membe	0.00097528	-0.244726
7919382	---		---	0.00360486	0.212697
8164252	NM_170600	SH2D3C	NM_170600 // SH2D3C // SH2 domain containing 3C // 9q34.11 // 10044 /// NM_005489 // SH	0.00209554	0.224997
8024485	NM_015675	GADD45B	NM_015675 // GADD45B // growth arrest and DNA-damage-inducible, beta // 19p13.3 // 4616	0.000569978	-0.259077
8051413	NM_015475	FAM98A	NM_015475 // FAM98A // family with sequence similarity 98, member A // 2p22.3 // 25940	0.000222816	-0.262147
7989159	NM_017661	ZNF280D	NM_017661 // ZNF280D // zinc finger protein 280D // 15q21.3 // 54816 /// NM_001002843 /	0.00998413	0.18984

8171313	NM_013427	ARHGAP6	NM_013427 // ARHGAP6 // Rho GTPase activating protein 6 // Xp22.3 // 395 /// NM_006125	0.00794965	0.196624
7917875	NM_001993	F3	NM_001993 // F3 // coagulation factor III (thromboplastin, tissue factor) // 1p22-p21 /	1.34E-07	-0.389211
7999387	NM_001424	EMP2	NM_001424 // EMP2 // epithelial membrane protein 2 // 16p13.2 // 2013 /// ENST000003595	0.0022838	0.221865
8035825	NM_001001411	ZNF676	NM_001001411 // ZNF676 // zinc finger protein 676 // 19p12 // 163223 /// ENST0000039712	0.00987311	0.19416
8047563	---		---	0.00319819	0.21616
7952249	NM_004205	USP2	NM_004205 // USP2 // ubiquitin specific peptidase 2 // 11q23.3 // 9099 /// NM_171997 //	0.000623647	-0.246772
8109086	NM_000024	ADRB2	NM_000024 // ADRB2 // adrenergic, beta-2-, receptor, surface // 5q31-q32 // 154 /// ENS	2.31E-05	0.299215
8055323	NM_207363	NCKAP5	NM_207363 // NCKAP5 // NCK-associated protein 5 // 2q21.2 // 344148 /// NM_207481 // NC	0.000421902	0.256701
7936242	NM_033397	ITPRIP	NM_033397 // ITPRIP // inositol 1,4,5-triphosphate receptor interacting protein // 10q2	0.00095231	-0.252146
8098470	NM_024949	WWC2	NM_024949 // WWC2 // WW and C2 domain containing 2 // 4q35.1 // 80014 /// ENST000004037	0.00259606	0.2181
8068593	NM_005239	ETS2	NM_005239 // ETS2 // v-ets erythroblastosis virus E26 oncogene homolog 2 (avian) // 21q	0.00621111	-0.207285
8019046	NM_014740	EIF4A3	NM_014740 // EIF4A3 // eukaryotic translation initiation factor 4A3 // 17q25.3 // 9775	0.00253545	-0.228287
8150889	NM_138969	SDR16C5	NM_138969 // SDR16C5 // short chain dehydrogenase/reductase family 16C, member 5 // 8q1	0.000965564	-0.249271
8119016	NM_002754	MAPK13	NM_002754 // MAPK13 // mitogen-activated protein kinase 13 // 6p21.31 // 5603 /// ENST0	0.000106896	-0.276195

8072678	NM_002133	HMOX1	NM_002133 // HMOX1 // heme oxygenase (decycling) 1 // 22q12|22q13.1 // 3162 /// ENST000	0.00221923	-0.229948
8162313	NM_013417	IARS	NM_013417 // IARS // isoleucyl-tRNA synthetase // 9q21 // 3376 /// NM_002161 // IARS //	0.000241533	-0.262515
8079305	NM_003278	CLEC3B	NM_003278 // CLEC3B // C-type lectin domain family 3, member B // 3p22-p21.3 // 7123 //	0.00014378	0.277662
8088820	NM_012234	RYBP	NM_012234 // RYBP // RING1 and YY1 binding protein // 3p13 // 23429 /// ENST00000477973	7.78E-05	-0.294325
7919751	NM_021960	MCL1	NM_021960 // MCL1 // myeloid cell leukemia sequence 1 (BCL2-related) // 1q21 // 4170 //	0.00033395	-0.270798
8141228	NM_001134450	TMEM130	NM_001134450 // TMEM130 // transmembrane protein 130 // 7q22.1 // 222865 /// NM_152913	0.000935108	0.244045
8178435	NM_003897	IER3	NM_003897 // IER3 // immediate early response 3 // 6p21.3 // 8870 /// ENST00000259874 /	0.000522607	-0.256748
8165709	---		---	5.42E-05	0.295577
8167601	NM_001145073	USP27X	NM_001145073 // USP27X // ubiquitin specific peptidase 27, X-linked // Xp11.23 // 38985	0.00872515	-0.198014
7974047	NM_001049	SSTR1	NM_001049 // SSTR1 // somatostatin receptor 1 // 14q13 // 6751 /// ENST00000267377 // S	0.00501177	0.206925
8109120	NM_152406	AFAP1L1	NM_152406 // AFAP1L1 // actin filament associated protein 1-like 1 // 5q32 // 134265 //	0.00261884	0.22145
7994659	NM_017458	MVP	NM_017458 // MVP // major vault protein // 16p11.2 // 9961 /// NM_005115 // MVP // majo	0.00385776	-0.211029
8092594	---		---	0.00205429	0.226051
7909225	NM_001004023	DYRK3	NM_001004023 // DYRK3 // dual-specificity tyrosine-(Y)-phosphorylation regulated kinase	0.00180924	-0.235203
7952341	NM_024769	ASAM	NM_024769 // ASAM // adipocyte-specific adhesion molecule // 11q24.1 // 79827 /// ENST0	0.00213691	-0.224824
7942783	BC002752	C11orf67	BC002752 // C11orf67 // chromosome 11 open reading frame 67 // 11q14.1 // 28971 /// BC0	0.000395261	0.258476

8102328	NM_000204	CFI	NM_000204 // CFI // complement factor I // 4q25 // 3426 /// ENST00000394634 // CFI // c	0.00722993	-0.197887
8035517	NM_000095	COMP	NM_000095 // COMP // cartilage oligomeric matrix protein // 19p13.1 // 1311 /// ENST000	0.000723043	-0.244387
8007348	NM_005854	RAMP2	NM_005854 // RAMP2 // receptor (G protein-coupled) activity modifying protein 2 // 17q1	0.00273042	0.220994
7934215	NM_014767	SPOCK2	NM_014767 // SPOCK2 // sparc/osteonectin, cwcv and kazal-like domains proteoglycan (tes	0.0041555	0.211255
8074991	NM_001099781	GGT5	NM_001099781 // GGT5 // gamma-glutamyltransferase 5 // 22q11.23 // 2687 /// NM_004121 /	0.000400513	-0.247876
8011823	NM_032530	ZNF594	NM_032530 // ZNF594 // zinc finger protein 594 // 17p13 // 84622 /// ENST00000399604 //	0.000281523	0.274713
7978644	NM_020529	NFKBIA	NM_020529 // NFKBIA // nuclear factor of kappa light polypeptide gene enhancer in B-cel	0.000661152	-0.258663
8076344	NM_001018050	POLR3H	NM_001018050 // POLR3H // polymerase (RNA) III (DNA directed) polypeptide H (22.9kD) //	0.0041436	-0.213748
8102482	NM_014822	SEC24D	NM_014822 // SEC24D // SEC24 family, member D (S. cerevisiae) // 4q26 // 9871 /// ENST0	3.59E-08	-0.384068
8151788	NM_203390	RBM12B	NM_203390 // RBM12B // RNA binding motif protein 12B // 8q22.1 // 389677 /// ENST000003	0.000737969	0.258168
8157905	NM_033446	FAM125B	NM_033446 // FAM125B // family with sequence similarity 125, member B // 9q33.3 // 8985	7.77E-05	0.285861
8086627	NR_033815	ALS2CL	NR_033815 // ALS2CL // ALS2 C-terminal like // 3p21.31 // 259173 /// NM_147129 // ALS2C	0.00266878	0.213221
8020090	NM_020648	TWSG1	NM_020648 // TWSG1 // twisted gastrulation homolog 1 (Drosophila) // 18p11.3 // 57045 /	0.00474533	-0.204175
7917304	NM_018298	MCOLN3	NM_018298 // MCOLN3 // mucolipin 3 // 1p22.3 // 55283 /// ENST00000302814 // MCOLN3 //	0.00167573	0.229733
8158627	NM_014286	NCS1	NM_014286 // NCS1 // neuronal calcium sensor 1 // 9q34 // 23413 /// NM_001128826 // NCS	0.000211353	-0.259443

8114733	NM_022481	ARAP3	NM_022481 // ARAP3 // ArfGAP with RhoGAP domain, ankyrin repeat and PH domain 3 // 5q31	0.00746554	0.198483
7901054	NM_004073	PLK3	NM_004073 // PLK3 // polo-like kinase 3 // 1p34.1 // 1263 /// NM_001013632 // TCTEX1D4	0.00405185	-0.21771
8036813	NM_001005851	ZNF780B	NM_001005851 // ZNF780B // zinc finger protein 780B // 19q13.2 // 163131 /// ENST000004	0.01024	0.192683
8017688	NM_199340	LRRC37A3	NM_199340 // LRRC37A3 // leucine rich repeat containing 37, member A3 // 17q24.1 // 374	0.00875568	0.194225
7897620	NM_002631	PGD	NM_002631 // PGD // phosphogluconate dehydrogenase // 1p36.22 // 5226 /// ENST000002707	0.000287952	-0.256391
8073960	NM_001001852	PIM3	NM_001001852 // PIM3 // pim-3 oncogene // 22q13 // 415116 /// ENST00000360612 // PIM3 /	0.000214837	-0.280178
8089835	NM_007085	FSTL1	NM_007085 // FSTL1 // follistatin-like 1 // 3q13.33 // 11167 /// NR_029584 // MIR198 //	0.00106465	-0.230309
8148184	NM_032899	FAM83A	NM_032899 // FAM83A // family with sequence similarity 83, member A // 8q24.13 // 84985	0.00904722	-0.193783
7941260	NM_031904	FRMD8	NM_031904 // FRMD8 // FERM domain containing 8 // 11q13 // 83786 /// ENST00000317568 //	0.00260271	-0.22663
7933855	NM_145307	RTKN2	NM_145307 // RTKN2 // rhotekin 2 // 10q21.2 // 219790 /// ENST00000373789 // RTKN2 // r	2.94E-05	0.300322
8124848	NM_003897	IER3	NM_003897 // IER3 // immediate early response 3 // 6p21.3 // 8870 /// ENST00000259874 /	0.000194133	-0.276112
8157487	NM_002581	PAPPA	NM_002581 // PAPPA // pregnancy-associated plasma protein A, pappalysin 1 // 9q33.2 //	0.000172229	-0.265535
7988467	NM_000138	FBN1	NM_000138 // FBN1 // fibrillin 1 // 15q21.1 // 2200 /// ENST00000316623 // FBN1 // fibr	0.00897688	-0.187211
8129392	NM_001012279	C6orf174	NM_001012279 // C6orf174 // chromosome 6 open reading frame 174 // 6q22.33 // 387104 //	0.00128019	0.231007
7918900	---		---	1.44E-07	0.388813

7951485	NM_017515	SLC35F2	NM_017515 // SLC35F2 // solute carrier family 35, member F2 // 11q22.3 // 54733 /// ENS	0.00491873	-0.204135
7982339	AK097050	LOC100130857	AK097050 // LOC100130857 // hypothetical protein LOC100130857 // 15q13.3 // 100130857	0.000209197	0.273768
8172520	NM_006521	TFE3	NM_006521 // TFE3 // transcription factor binding to IGHM enhancer 3 // Xp11.22 // 7030	0.00962822	-0.196854
7965873	NM_001111283	IGF1	NM_001111283 // IGF1 // insulin-like growth factor 1 (somatomedin C) // 12q23.2 // 3479	0.00147796	-0.227829
8157800	NR_029611	MIR181A2	NR_029611 // MIR181A2 // microRNA 181a-2 // 9q33.3 // 406954	0.00668126	0.205278
8172204	NM_000898	MAOB	NM_000898 // MAOB // monoamine oxidase B // Xp11.23 // 4129 /// ENST00000378069 // MAOB	0.00344979	0.217913
8082465	NM_024768	CCDC48	NM_024768 // CCDC48 // coiled-coil domain containing 48 // 3q21.3 // 79825 /// AK022119	7.45E-08	0.376049
7939265	NM_024662	NAT10	NM_024662 // NAT10 // N-acetyltransferase 10 (GCN5-related) // 11p13 // 55226 /// NM_00	0.00233856	-0.230175
8123562	NM_001500	GMDS	NM_001500 // GMDS // GDP-mannose 4,6-dehydratase // 6p25 // 2762 /// ENST00000380815 //	0.0004715	-0.252567
7992639	NM_020705	TBC1D24	NM_020705 // TBC1D24 // TBC1 domain family, member 24 // 16p13.3 // 57465 /// ENST00000	0.00952666	-0.188941
8066619	NM_006227	PLTP	NM_006227 // PLTP // phospholipid transfer protein // 20q13.12 // 5360 /// NM_182676 //	0.00392794	-0.212879
8178676	NM_000434	NEU1	NM_000434 // NEU1 // sialidase 1 (lysosomal sialidase) // 6p21.3 // 4758 /// ENST000002	0.000116668	-0.283901
8052654	NM_020651	PELI1	NM_020651 // PELI1 // pellino homolog 1 (Drosophila) // 2p13.3 // 57162 /// ENST0000035	2.59E-05	-0.317598
8094499	---		---	0.00244335	0.216116
8064415	NM_018354	C20orf46	NM_018354 // C20orf46 // chromosome 20 open reading frame 46 // 20p13 // 55321 /// ENST	0.00401033	0.204733
7961626	NM_134431	SLCO1A2	NM_134431 // SLCO1A2 // solute carrier organic anion transporter family, member 1A2 //	0.00136252	0.227602

7997504	NM_001257	CDH13	NM_001257 // CDH13 // cadherin 13, H-cadherin (heart) // 16q23.3 // 1012 /// ENST000002	0.000291422	0.267232
8135587	NM_001233	CAV2	NM_001233 // CAV2 // caveolin 2 // 7q31.1 // 858 /// NM_198212 // CAV2 // caveolin 2 //	0.00193626	0.22927
7923659	NM_032833	PPP1R15B	NM_032833 // PPP1R15B // protein phosphatase 1, regulatory (inhibitor) subunit 15B // 1	1.24E-05	-0.325098
8150592	NM_005195	CEBPD	NM_005195 // CEBPD // CCAAT/enhancer binding protein (C/EBP), delta // 8p11.2-p11.1 //	1.80E-06	-0.356396
7918913	NM_001542	IGSF3	NM_001542 // IGSF3 // immunoglobulin superfamily, member 3 // 1p13 // 3321 /// NM_00100	0.00902327	-0.19682
7948612	NM_013402	FADS1	NM_013402 // FADS1 // fatty acid desaturase 1 // 11q12.2-q13.1 // 3992 /// ENST00000350	0.00993436	-0.193587
8027429	---		---	0.00473548	0.211962
8089467	NM_024508	ZBED2	NM_024508 // ZBED2 // zinc finger, BED-type containing 2 // 3q13.2 // 79413 /// ENST000	5.25E-05	0.287227
7902441	NM_030965	ST6GALNAC5	NM_030965 // ST6GALNAC5 // ST6 (alpha-N-acetyl-neuraminyl-2,3-beta-galactosyl-1,3)-N-ac	0.00291716	0.219659
7924686	NM_152608	C1orf55	NM_152608 // C1orf55 // chromosome 1 open reading frame 55 // 1q42.12 // 163859 /// ENS	0.000874424	-0.251474
8013042	NM_000422	KRT17	NM_000422 // KRT17 // keratin 17 // 17q21.2 // 3872 /// ENST00000311208 // KRT17 // ker	0.000813322	-0.243003
7922326	NR_029627	MIR214	NR_029627 // MIR214 // microRNA 214 // 1q24.3 // 406996 /// NR_036066 // MIR3120 // mic	1.12E-05	0.316663
8090852	NM_016201	AMOTL2	NM_016201 // AMOTL2 // angiomotin like 2 // 3q21-q22 // 51421 /// ENST00000249883 // AM	0.00039412	0.262303
7939072	NM_152636	METT5D1	NM_152636 // METT5D1 // methyltransferase 5 domain containing 1 // 11p14.1 // 196074 //	0.00281404	0.221775
7986977	NM_003257	TJP1	NM_003257 // TJP1 // tight junction protein 1 (zona occludens 1) // 15q13 // 7082 /// N	0.00274437	0.221234

7954090	NM_001423	EMP1	NM_001423 // EMP1 // epithelial membrane protein 1 // 12p12.3 // 2012 /// ENST000002569	0.000499203	-0.260055
8052622	---		---	0.00435797	-0.218507
7939492	NM_001145033	C11orf96	NM_001145033 // C11orf96 // chromosome 11 open reading frame 96 // 11p11.2 // 387763 //	1.18E-05	-0.324644
7964872	NM_001109754	PTPRB	NM_001109754 // PTPRB // protein tyrosine phosphatase, receptor type, B // 12q15-q21 //	8.80E-05	0.288714
8037913	NM_003827	NAPA	NM_003827 // NAPA // N-ethylmaleimide-sensitive factor attachment protein, alpha // 19q	0.000293578	-0.268628
7928882	NM_006829	C10orf116	NM_006829 // C10orf116 // chromosome 10 open reading frame 116 // 10q23.2 // 10974 ///	0.000534522	0.25578
8021081	NM_001128588	SLC14A1	NM_001128588 // SLC14A1 // solute carrier family 14 (urea transporter), member 1 (Kidd	0.00484525	0.206899
7938608	NM_006108	SPON1	NM_006108 // SPON1 // spondin 1, extracellular matrix protein // 11p15.2 // 10418 /// E	0.00521042	-0.205421
8022531	NM_000271	NPC1	NM_000271 // NPC1 // Niemann-Pick disease, type C1 // 18q11-q12 // 4864 /// ENST0000026	0.00241048	-0.215562
8095221	NM_001079525	PAICS	NM_001079525 // PAICS // phosphoribosylaminoimidazole carboxylase, phosphoribosylaminoi	0.000133794	-0.274076
8122334	NM_178445	CCRL1	NM_178445 // CCRL1 // chemokine (C-C motif) receptor-like 1 // 3q22 // 51554 /// NM_016	0.00698548	0.20103
8067944	NR_029480	MIRLET7C	NR_029480 // MIRLET7C // microRNA let-7c // 21q21.1 // 406885	4.99E-07	0.365904
7924526	NM_005426	TP53BP2	NM_005426 // TP53BP2 // tumor protein p53 binding protein, 2 // 1q42.1 // 7159 /// NM_0	0.00267218	-0.225673
7902317	NM_015978	TNNI3K	NM_015978 // TNNI3K // TNNI3 interacting kinase // 1p31.1 // 51086 /// NM_001112808 //	0.0026257	0.231174
8038861	NM_001245	SIGLEC6	NM_001245 // SIGLEC6 // sialic acid binding Ig-like lectin 6 // 19q13.3 // 946 /// NM_1	0.00320745	0.222105
8038899	NM_001193306	FPR1	NM_001193306 // FPR1 // formyl peptide receptor 1 // 19q13.4 // 2357 /// NM_002029 // F	0.000402446	-0.270186

8024228	NM_177401	MIDN	NM_177401 // MIDN // midnolin // 19p13.3 // 90007 /// ENST00000300952 // MIDN // midnol	5.34E-05	-0.298916
7935027	NM_004969	IDE	NM_004969 // IDE // insulin-degrading enzyme // 10q23-q25 // 3416 /// NM_001165946 // I	3.54E-05	-0.294119
8155359	NM_033655	CNTNAP3	NM_033655 // CNTNAP3 // contactin associated protein-like 3 // 9p13.1 // 79937 /// AF33	0.000496003	0.258955
8064502	NM_003091	SNRPB	NM_003091 // SNRPB // small nuclear ribonucleoprotein polypeptides B and B1 // 20p13 //	0.00344799	-0.222556
7984319	NM_002755	MAP2K1	NM_002755 // MAP2K1 // mitogen-activated protein kinase kinase 1 // 15q22.1-q22.33 // 5	0.00171037	-0.232204
8121569	AK091822	FLJ34503	AK091822 // FLJ34503 // hypothetical FLJ34503 // 6q21 // 285759	2.29E-05	0.304683
7992893	---		---	0.000522908	0.24715
8155540	NM_033655	CNTNAP3	NM_033655 // CNTNAP3 // contactin associated protein-like 3 // 9p13.1 // 79937 /// AF33	0.00435028	0.214742
8161460	NM_033655	CNTNAP3	NM_033655 // CNTNAP3 // contactin associated protein-like 3 // 9p13.1 // 79937 /// AF33	0.00435025	0.214742
8061247	NM_018993	RIN2	NM_018993 // RIN2 // Ras and Rab interactor 2 // 20p11.22 // 54453 /// ENST00000255006	0.00345511	0.217327
8131069	NM_001039966	GPER	NM_001039966 // GPER // G protein-coupled estrogen receptor 1 // 7p22.3 // 2852 /// NM_	0.00774891	0.190537
8106098	NM_005909	MAP1B	NM_005909 // MAP1B // microtubule-associated protein 1B // 5q13 // 4131 /// ENST0000029	0.0003039	-0.254368
7898448	NM_012387	PADI4	NM_012387 // PADI4 // peptidyl arginine deiminase, type IV // 1p36.13 // 23569 /// ENST	0.00911982	-0.198973
8161238	U66622	RAB1C	U66622 // RAB1C // RAB1C, member RAS oncogene family pseudogene // 9p13.2 // 441400	0.00669766	-0.202374
8096301	NM_001040058	SPP1	NM_001040058 // SPP1 // secreted phosphoprotein 1 // 4q22.1 // 6696 /// NM_000582 // SP	0.004073	-0.211507

8005707	NM_145109	MAP2K3	NM_145109 // MAP2K3 // mitogen-activated protein kinase kinase 3 // 17q11.2 // 5606 ///	6.01E-05	-0.305121
7920244	NM_002964	S100A8	NM_002964 // S100A8 // S100 calcium binding protein A8 // 1q21 // 6279 /// ENST00000368	0.00210902	-0.233281
8133670	NM_000941	POR	NM_000941 // POR // P450 (cytochrome) oxidoreductase // 7q11.2 // 5447 /// AF258341 //	0.00102158	-0.244053
8103951	NM_001995	ACSL1	NM_001995 // ACSL1 // acyl-CoA synthetase long-chain family member 1 // 4q35 // 2180 //	4.30E-07	-0.372213
7973352	NM_014045	LRP10	NM_014045 // LRP10 // low density lipoprotein receptor-related protein 10 // 14q11.2 //	0.00410628	-0.216006
8025402	NM_139314	ANGPTL4	NM_139314 // ANGPTL4 // angiopoietin-like 4 // 19p13.3 // 51129 /// NM_001039667 // ANG	0.000444353	-0.265213
8065433	NM_020531	C20orf3	NM_020531 // C20orf3 // chromosome 20 open reading frame 3 // 20p11.2 // 57136 /// ENST	0.00616591	-0.203604
8060963	NM_003081	SNAP25	NM_003081 // SNAP25 // synaptosomal-associated protein, 25kDa // 20p12-p11.2 // 6616 //	0.00599916	-0.203731
8048257	NM_015488	PNKD	NM_015488 // PNKD // paroxysmal nonkinesigenic dyskinesia // 2q35 // 25953 /// NM_02257	0.00141905	-0.237245
8127158	NM_001498	GCLC	NM_001498 // GCLC // glutamate-cysteine ligase, catalytic subunit // 6p12 // 2729 /// N	0.000689532	-0.250071
7997188	NM_005143	HP	NM_005143 // HP // haptoglobin // 16q22.1 // 3240 /// NM_001126102 // HP // haptoglobin	5.98E-05	-0.306571
8135069	NM_000602	SERPINE1	NM_000602 // SERPINE1 // serpin peptidase inhibitor, clade E (nexin, plasminogen activa	3.15E-09	-0.427326
8087790	NM_004704	RRP9	NM_004704 // RRP9 // ribosomal RNA processing 9, small subunit (SSU) processome compone	0.00951653	-0.199347
7991047	AK097109	LOC100131860	AK097109 // LOC100131860 // hypothetical protein LOC100131860 // 15q25.2 // 100131860	0.00218295	0.228659

8114787	NM_005471	GNPDA1	NM_005471 // GNPDA1 // glucosamine-6-phosphate deaminase 1 // 5q21 // 10007 /// ENST000	0.00307571	-0.223003
7949603	NM_015399	BRMS1	NM_015399 // BRMS1 // breast cancer metastasis suppressor 1 // 11q13-q13.2 // 25855 ///	0.00711099	-0.204255
8044963	---		---	0.0038837	0.216916
8070665	NM_173354	SIK1	NM_173354 // SIK1 // salt-inducible kinase 1 // 21q22.3 // 150094 /// ENST00000270162 /	0.000520293	-0.265661
7973336	NM_004995	MMP14	NM_004995 // MMP14 // matrix metallopeptidase 14 (membrane-inserted) // 14q11-q12 // 43	0.00203746	-0.227033
8074806	NM_014634	PPM1F	NM_014634 // PPM1F // protein phosphatase, Mg2+/Mn2+ dependent, 1F // 22q11.22 // 9647	0.00921409	0.193174
8116445	NM_182925	FLT4	NM_182925 // FLT4 // fms-related tyrosine kinase 4 // 5q35.3 // 2324 /// NM_002020 // F	0.00102199	0.242994
8075390	NM_174977	SEC14L4	NM_174977 // SEC14L4 // SEC14-like 4 (S. cerevisiae) // 22q12.2 // 284904 /// NM_001161	0.00473482	0.206452
7989277	NM_004998	MYO1E	NM_004998 // MYO1E // myosin IE // 15q21-q22 // 4643 /// ENST00000288235 // MYO1E // my	6.25E-07	-0.351775
8118544	---		---	0.00252501	0.212538
8165888	---		---	0.000696702	0.251222
8099471	NM_031950	FGFBP2	NM_031950 // FGFBP2 // fibroblast growth factor binding protein 2 // 4p16 // 83888 ///	0.000923668	0.246402
8018731	NM_024599	RHBDF2	NM_024599 // RHBDF2 // rhomboid 5 homolog 2 (Drosophila) // 17q25.1 // 79651 /// NM_001	0.000493385	-0.262367
8174119	NM_001011657	ZMAT1	NM_001011657 // ZMAT1 // zinc finger, matrin-type 1 // Xq21 // 84460 /// NR_036431 // Z	0.000317255	0.270388
8144378	NM_018361	AGPAT5	NM_018361 // AGPAT5 // 1-acylglycerol-3-phosphate O-acyltransferase 5 (lysophosphatidic	0.00144431	-0.233509
8102389	NM_018392	C4orf21	NM_018392 // C4orf21 // chromosome 4 open reading frame 21 // 4q25 // 55345 /// ENST000	0.00665718	0.206945

8063893	NM_007002	ADRM1	NM_007002 // ADRM1 // adhesion regulating molecule 1 // 20q13.33 // 11047 /// NM_175573	0.000158114	-0.281229
8091780	NM_001038628	B3GALNT1	NM_001038628 // B3GALNT1 // beta-1,3-N-acetylgalactosaminyltransferase 1 (globoside blo	0.00362013	0.214755
7918558	NM_004980	KCND3	NM_004980 // KCND3 // potassium voltage-gated channel, Shal-related subfamily, member 3	0.0002909	-0.259895
7897460	NM_032315	SLC25A33	NM_032315 // SLC25A33 // solute carrier family 25, member 33 // 1p36.22 // 84275 /// EN	6.33E-06	-0.339296
8131205	NM_152744	SDK1	NM_152744 // SDK1 // sidekick homolog 1, cell adhesion molecule (chicken) // 7p22.2 //	0.00477794	-0.208026
8104129	---		---	7.37E-05	0.291672
7919146	NR_003366	ANKRD20B	NR_003366 // ANKRD20B // ankyrin repeat domain 20B // 2q11.1 // 729171 /// AK090412 //	6.73E-06	0.328608
8087100	NM_003365	UQCRC1	NM_003365 // UQCRC1 // ubiquinol-cytochrome c reductase core protein I // 3p21.3 // 738	0.00477585	-0.211136
8169640	NM_001152	SLC25A5	NM_001152 // SLC25A5 // solute carrier family 25 (mitochondrial carrier; adenine nucleo	0.00650484	-0.204598
8037374	NM_002659	PLAUR	NM_002659 // PLAUR // plasminogen activator, urokinase receptor // 19q13 // 5329 /// NM	3.43E-05	-0.304341
8129649	NM_052831	C6orf192	NM_052831 // C6orf192 // chromosome 6 open reading frame 192 // 6q22.3-q23.3 // 116843	0.00572747	-0.203781
8054800	---		---	0.000372048	0.255734
8117045	NM_001143942	RBM24	NM_001143942 // RBM24 // RNA binding motif protein 24 // 6p22.3 // 221662 /// NM_153020	0.00500596	-0.208204
8043621	NM_212481	ARID5A	NM_212481 // ARID5A // AT rich interactive domain 5A (MRF1-like) // 2q11.2 // 10865 ///	0.000440254	-0.262503
8004842	NM_173728	ARHGEF15	NM_173728 // ARHGEF15 // Rho guanine nucleotide exchange factor (GEF) 15 // 17p13.1 //	0.00669151	0.198342

8063386	NM_005194	CEBPB	NM_005194 // CEBPB // CCAAT/enhancer binding protein (C/EBP), beta // 20q13.1 // 1051 /	3.03E-06	-0.349424
8005449	NM_000422	KRT17	NM_000422 // KRT17 // keratin 17 // 17q21.2 // 3872 /// ENST00000311208 // KRT17 // ker	0.00210128	-0.224896
8102532	NM_001083	PDE5A	NM_001083 // PDE5A // phosphodiesterase 5A, cGMP-specific // 4q27 // 8654 /// NM_033430	0.00161577	0.239242
8148565	NM_002066	GML	NM_002066 // GML // glycosylphosphatidylinositol anchored molecule like protein // 8q24	0.00456757	0.210492
8057620	NM_000393	COL5A2	NM_000393 // COL5A2 // collagen, type V, alpha 2 // 2q14-q32 // 1290 /// ENST0000037486	0.00167467	-0.227343
8017843	NM_001174166	SLC16A6	NM_001174166 // SLC16A6 // solute carrier family 16, member 6 (monocarboxylic acid tran	0.00012456	-0.288356
8035201	NM_015692	CPAMD8	NM_015692 // CPAMD8 // C3 and PZP-like, alpha-2-macroglobulin domain containing 8 // 19	0.00298607	0.214584
8125383	NM_004557	NOTCH4	NM_004557 // NOTCH4 // notch 4 // 6p21.3 // 4855 /// NM_022107 // GPSM3 // G-protein si	0.0102217	0.189439
8085914	NM_003615	SLC4A7	NM_003615 // SLC4A7 // solute carrier family 4, sodium bicarbonate cotransporter, membe	0.000635284	-0.247
7985268	NM_000137	FAH	NM_000137 // FAH // fumarylacetoacetate hydrolase (fumarylacetoacetase) // 15q23-q25 //	0.000754483	-0.255105
7938348	NM_003390	WEE1	NM_003390 // WEE1 // WEE1 homolog (S. pombe) // 11p15.3-p15.1 // 7465 /// NM_001143976	0.00483684	-0.208088
7900488	NR_029846	MIR30E	NR_029846 // MIR30E // microRNA 30e // 1p34.2 // 407034	3.77E-05	0.304432
7936968	NM_003474	ADAM12	NM_003474 // ADAM12 // ADAM metallopeptidase domain 12 // 10q26.3 // 8038 /// NM_021641	0.00753273	-0.192918
8056100	---		---	0.000380832	0.267883
8169000	---		---	0.00705134	0.202509

8104348	---		---	0.00127006	0.23712
8151496	NM_001033723	ZNF704	NM_001033723 // ZNF704 // zinc finger protein 704 // 8q21.13 // 619279 /// ENST00000327	8.04E-05	0.292141
7960900	NM_014358	CLEC4E	NM_014358 // CLEC4E // C-type lectin domain family 4, member E // 12p13.31 // 26253 ///	0.00178212	-0.240034
7957551	NM_003877	SOCS2	NM_003877 // SOCS2 // suppressor of cytokine signaling 2 // 12q // 8835 /// ENST0000034	9.22E-05	-0.291529
7933310	---		---	1.67E-05	0.318128
8131111	NM_001037283	EIF3B	NM_001037283 // EIF3B // eukaryotic translation initiation factor 3, subunit B // 7p22.	0.00264301	-0.229594
7971350	NM_183422	TSC22D1	NM_183422 // TSC22D1 // TSC22 domain family, member 1 // 13q14 // 8848 /// NM_006022 //	0.00337722	-0.222802
8099685	NM_018176	LGI2	NM_018176 // LGI2 // leucine-rich repeat LGI family, member 2 // 4p15.2 // 55203 /// EN	4.28E-05	-0.295773
7948088	NM_032315	SLC25A33	NM_032315 // SLC25A33 // solute carrier family 25, member 33 // 1p36.22 // 84275 /// EN	3.24E-06	-0.350226
8083445	---		---	0.000127213	0.284386
8113666	NM_020796	SEMA6A	NM_020796 // SEMA6A // sema domain, transmembrane domain (TM), and cytoplasmic domain,	0.00340501	0.218743
8063028	NM_052951	DNTTIP1	NM_052951 // DNTTIP1 // deoxynucleotidyltransferase, terminal, interacting protein 1 //	2.63E-05	-0.314497
8106743	NM_004385	VCAN	NM_004385 // VCAN // versican // 5q14.3 // 1462 /// NM_001164097 // VCAN // versican //	3.40E-06	-0.33253
8158167	NM_005564	LCN2	NM_005564 // LCN2 // lipocalin 2 // 9q34 // 3934 /// ENST00000277480 // LCN2 // lipocal	0.00492813	-0.209175
8046086	NM_203463	LASS6	NM_203463 // LASS6 // LAG1 homolog, ceramide synthase 6 // 2q24.3 // 253782 /// ENST000	2.33E-05	-0.308998
7902227	NM_001924	GADD45A	NM_001924 // GADD45A // growth arrest and DNA-damage-inducible, alpha // 1p31.2 // 1647	4.58E-11	-0.445024

8067233	NM_020182	PMEPA1	NM_020182 // PMEPA1 // prostate transmembrane protein, androgen induced 1 // 20q13.31-q	0.00320121	-0.213425
8035506	NM_004750	CRLF1	NM_004750 // CRLF1 // cytokine receptor-like factor 1 // 19p12 // 9244 /// ENST00000392	0.00042902	-0.25852
8015706	NR_024461	LOC100190938	NR_024461 // LOC100190938 // hypothetical LOC100190938 // 17q21.31 // 100190938 /// NR_	5.40E-06	0.325628
7998759	NM_001042371	PGP	NM_001042371 // PGP // phosphoglycolate phosphatase // 16p13.3 // 283871 /// ENST000003	0.00132517	-0.241567
7983650	NM_003645	SLC27A2	NM_003645 // SLC27A2 // solute carrier family 27 (fatty acid transporter), member 2 //	0.0045605	-0.208087
7929750	NM_020354	ENTPD7	NM_020354 // ENTPD7 // ectonucleoside triphosphate diphosphohydrolase 7 // --- // 57089	2.06E-07	-0.366732
7977621	NM_201540	NDRG2	NM_201540 // NDRG2 // NDRG family member 2 // 14q11.2 // 57447 /// NM_201535 // NDRG2 /	0.00656127	0.203752
7913185	---		---	0.000101688	0.28762
8074734	AK128837	LOC284861	AK128837 // LOC284861 // hypothetical LOC284861 // 22q11.21 // 284861	0.000931697	0.245438
7940582	NM_004183	BEST1	NM_004183 // BEST1 // bestrophin 1 // 11q13 // 7439 /// NM_001139443 // BEST1 // bestro	0.00446236	-0.212586
8122986	NM_016224	SNX9	NM_016224 // SNX9 // sorting nexin 9 // 6q25.1-q26 // 51429 /// ENST00000392185 // SNX9	0.000286989	-0.263102
8065136	NM_001042576	RRBP1	NM_001042576 // RRBP1 // ribosome binding protein 1 homolog 180kDa (dog) // 20p12 // 62	1.07E-05	-0.320305
7919139	AK090412	LOC375010	AK090412 // LOC375010 // ankyrin repeat domain 20 family, member A pseudogene // 1q21.1	2.45E-05	0.309708
7963410	NM_173086	KRT6C	NM_173086 // KRT6C // keratin 6C // 12q13.13 // 286887 /// NM_005555 // KRT6B // kerati	0.000211518	-0.272872
7912937	NM_007365	PADI2	NM_007365 // PADI2 // peptidyl arginine deiminase, type II // 1p36.13 // 11240 /// ENST	0.00405307	-0.216479

7953508	NM_000365	TPI1	NM_000365 // TPI1 // triosephosphate isomerase 1 // 12p13 // 7167 /// NM_001159287 // T	0.00508236	-0.212495
7905553	NM_003125	SPRR1B	NM_003125 // SPRR1B // small proline-rich protein 1B // 1q21-q22 // 6699 /// ENST000003	0.00219515	-0.229064
7900658	NM_005424	TIE1	NM_005424 // TIE1 // tyrosine kinase with immunoglobulin-like and EGF-like domains 1 //	0.00824518	0.193995
7902518	NM_017655	GIPC2	NM_017655 // GIPC2 // GIPC PDZ domain containing family, member 2 // 1p31.1 // 54810 //	0.00230812	0.231334
8002969	NM_001031804	MAF	NM_001031804 // MAF // v-maf musculoaponeurotic fibrosarcoma oncogene homolog (avian) /	0.00149508	0.241401
8111136	NM_001034850	FAM134B	NM_001034850 // FAM134B // family with sequence similarity 134, member B // 5p15.1 // 5	0.00830132	-0.198772
8082597	NM_001102608	COL6A6	NM_001102608 // COL6A6 // collagen, type VI, alpha 6 // 3q22.1 // 131873 /// ENST000003	0.00231994	0.228826
8110090	NM_022754	SFXN1	NM_022754 // SFXN1 // sideroflexin 1 // --- // 94081 /// ENST00000321442 // SFXN1 // si	0.000168858	-0.276031
7951271	NM_002421	MMP1	NM_002421 // MMP1 // matrix metallopeptidase 1 (interstitial collagenase) // 11q22.3 //	0.000127537	-0.28041
8078066	NM_152536	FGD5	NM_152536 // FGD5 // FYVE, RhoGEF and PH domain containing 5 // 3p25.1 // 152273 /// EN	0.00071903	0.244474
8165644	---		---	5.15E-05	0.307585
8077786	NM_001570	IRAK2	NM_001570 // IRAK2 // interleukin-1 receptor-associated kinase 2 // 3p25.3 // 3656 ///	0.000250099	-0.276264
8013243	NM_004169	SHMT1	NM_004169 // SHMT1 // serine hydroxymethyltransferase 1 (soluble) // 17p11.2 // 6470 //	0.00133283	-0.242743
8017711	NM_006572	GNA13	NM_006572 // GNA13 // guanine nucleotide binding protein (G protein), alpha 13 // 17q24	0.00597351	-0.209358
8097305	---		---	0.000827903	0.250539

7968004	NM_000231	SGCG	NM_000231 // SGCG // sarcoglycan, gamma (35kDa dystrophin-associated glycoprotein) // 1	2.33E-06	0.339601
8156599	---		---	9.20E-06	0.324958
8097903	NM_003264	TLR2	NM_003264 // TLR2 // toll-like receptor 2 // 4q32 // 7097 /// ENST00000260010 // TLR2 /	5.07E-07	-0.376388
8015387	NM_000422	KRT17	NM_000422 // KRT17 // keratin 17 // 17q21.2 // 3872 /// ENST00000311208 // KRT17 // ker	0.00110291	-0.238628
8126666	NM_004556	NFKBIE	NM_004556 // NFKBIE // nuclear factor of kappa light polypeptide gene enhancer in B-cel	0.00399471	-0.220245
8110392	NM_017510	TMED9	NM_017510 // TMED9 // transmembrane emp24 protein transport domain containing 9 // 5q35	0.000143373	-0.287567
8162117	NM_016548	GOLM1	NM_016548 // GOLM1 // golgi membrane protein 1 // 9q21.33 // 51280 /// NM_177937 // GOL	0.0006173	-0.249283
7924819	BC019830	PRO2012	BC019830 // PRO2012 // hypothetical protein PRO2012 // 1q42.13 // 55478	0.00320198	0.218016
7981947	NR_001295	SNORD109A	NR_001295 // SNORD109A // small nucleolar RNA, C/D box 109A // 15q11.2 // 338428 /// NR	0.000829125	0.239234
7982098	NR_001295	SNORD109A	NR_001295 // SNORD109A // small nucleolar RNA, C/D box 109A // 15q11.2 // 338428 /// NR	0.000829125	0.239234
8096733	NM_001136258	SGMS2	NM_001136258 // SGMS2 // sphingomyelin synthase 2 // 4q25 // 166929 /// NM_152621 // SG	0.00883339	-0.199103
8176133	NM_000402	G6PD	NM_000402 // G6PD // glucose-6-phosphate dehydrogenase // Xq28 // 2539 /// NM_001042351	0.00049137	-0.249969
8142343	---		---	0.00149629	0.228683
8113709	NM_002317	LOX	NM_002317 // LOX // lysyl oxidase // 5q23.2 // 4015 /// NM_001178102 // LOX // lysyl ox	1.83E-05	-0.307157
8091385	NM_000096	CP	NM_000096 // CP // ceruloplasmin (ferroxidase) // 3q23-q25 // 1356 /// ENST00000264613	0.00443689	-0.210041

7933933	NM_021800	DNAJC12	NM_021800 // DNAJC12 // DnaJ (Hsp40) homolog, subfamily C, member 12 // 10q22.1 // 5652	0.00952171	-0.190846
8006736	NM_007026	DUSP14	NM_007026 // DUSP14 // dual specificity phosphatase 14 // 17q12 // 11072 /// ENST000003	0.000123622	-0.284034
8135216	---		---	0.00554678	0.210527
8083569	NM_015508	TIPARP	NM_015508 // TIPARP // TCDD-inducible poly(ADP-ribose) polymerase // 3q25.31 // 25976 /	2.58E-05	-0.31731
8023766	NM_173630	RTTN	NM_173630 // RTTN // rotatin // 18q22.2 // 25914 /// ENST00000255674 // RTTN // rotatin	0.000702848	0.255237
8034712	NM_024825	PODNL1	NM_024825 // PODNL1 // podocan-like 1 // 19p13.12 // 79883 /// NM_001146254 // PODNL1 /	0.00012433	-0.277641
8041995	NM_003128	SPTBN1	NM_003128 // SPTBN1 // spectrin, beta, non-erythrocytic 1 // 2p21 // 6711 /// NM_178313	0.0017597	0.233711
8169709	NM_016417	GLRX5	NM_016417 // GLRX5 // glutaredoxin 5 // 14q32.13 // 51218 /// ENST00000331334 // GLRX5	0.00373615	-0.221419
7915032	NM_001038633	RSPO1	NM_001038633 // RSPO1 // R-spondin homolog (Xenopus laevis) // 1p34.3 // 284654 /// ENS	0.00184335	0.2312
8023401	NM_025214	CCDC68	NM_025214 // CCDC68 // coiled-coil domain containing 68 // 18q21 // 80323 /// NM_001143	0.00817468	0.198015
8076137	NM_015374	SUN2	NM_015374 // SUN2 // Sad1 and UNC84 domain containing 2 // 22q13.1 // 25777 /// ENST000	0.00998507	0.182752
8135955	NM_001219	CALU	NM_001219 // CALU // calumenin // 7q32.1 // 813 /// NM_001130674 // CALU // calumenin /	2.87E-05	-0.298942
8136662	NM_004668	MGAM	NM_004668 // MGAM // maltase-glucoamylase (alpha-glucosidase) // 7q34 // 8972 /// ENST0	7.91E-05	-0.298991
7973869	---		---	0.00329521	0.218811
8161648	NM_001206	KLF9	NM_001206 // KLF9 // Kruppel-like factor 9 // 9q13 // 687 /// ENST00000377126 // KLF9 /	0.00113644	-0.249108

8171248	NM_000216	KAL1	NM_000216 // KAL1 // Kallmann syndrome 1 sequence // Xp22.32 // 3730 /// ENST0000026264	0.00187742	0.229731
8112428	NM_005582	CD180	NM_005582 // CD180 // CD180 molecule // 5q12 // 4064 /// ENST00000256447 // CD180 // CD	0.00129798	-0.240377
8039842	NM_012312	KIR2DS2	NM_012312 // KIR2DS2 // killer cell immunoglobulin-like receptor, two domains, short cy	0.00970928	0.193657
8164131	NM_173690	SCAI	NM_173690 // SCAI // suppressor of cancer cell invasion // 9q33.3 // 286205 /// NM_0011	0.000228516	0.268354
8031328	NM_006737	KIR3DL2	NM_006737 // KIR3DL2 // killer cell immunoglobulin-like receptor, three domains, long c	0.00875886	0.195352
8021727	NM_018235	CNDP2	NM_018235 // CNDP2 // CNDP dipeptidase 2 (metallopeptidase M20 family) // 18q22.3 // 55	0.004968	-0.211473
7979927	NM_003814	ADAM20	NM_003814 // ADAM20 // ADAM metallopeptidase domain 20 // 14q24.1 // 8748 /// ENST00000	0.000877715	0.247629
7901426	NM_004799	ZFYVE9	NM_004799 // ZFYVE9 // zinc finger, FYVE domain containing 9 // 1p32.3 // 9372 /// NM_0	0.00248989	0.226309
8100870	NM_014243	ADAMTS3	NM_014243 // ADAMTS3 // ADAM metallopeptidase with thrombospondin type 1 motif, 3 // 4q	3.10E-05	-0.303342
7975268	NM_001172	ARG2	NM_001172 // ARG2 // arginase, type II // 14q24.1 // 384 /// NM_006370 // VTI1B // vesi	1.71E-06	-0.354725
7908793	NM_001114309	ELF3	NM_001114309 // ELF3 // E74-like factor 3 (ets domain transcription factor, epithelial-	0.00210693	-0.234173
8018864	NM_003955	SOCS3	NM_003955 // SOCS3 // suppressor of cytokine signaling 3 // 17q25.3 // 9021 /// ENST000	9.24E-05	-0.295894
7976496	NM_001085	SERPINA3	NM_001085 // SERPINA3 // serpin peptidase inhibitor, clade A (alpha-1 antiproteinase, a	5.78E-08	-0.377439
8125139	NM_000434	NEU1	NM_000434 // NEU1 // sialidase 1 (lysosomal sialidase) // 6p21.3 // 4758 /// ENST000002	0.000409085	-0.261616

8179851	NM_000434	NEU1	NM_000434 // NEU1 // sialidase 1 (lysosomal sialidase) // 6p21.3 // 4758 /// ENST000002	0.000409085	-0.261616
7907271	NM_001460	FMO2	NM_001460 // FMO2 // flavin containing monooxygenase 2 (non-functional) // 1q24.3 // 23	0.000394326	0.266525
7907531	NM_005684	GPR52	NM_005684 // GPR52 // G protein-coupled receptor 52 // 1q24 // 9293 /// ENST00000367685	0.000717414	0.250648
8139792	---		---	0.00774939	0.201164
7969640	NM_182848	CLDN10	NM_182848 // CLDN10 // claudin 10 // 13q31-q34 // 9071 /// NM_001160100 // CLDN10 // cl	7.81E-05	-0.289735
7900146	NM_025079	ZC3H12A	NM_025079 // ZC3H12A // zinc finger CCCH-type containing 12A // 1p34.3 // 80149 /// ENS	0.000325581	-0.272569
8030860	NM_001462	FPR2	NM_001462 // FPR2 // formyl peptide receptor 2 // 19q13.3-q13.4 // 2358 /// NM_00100573	0.00137275	-0.245197
7967993	NM_002010	FGF9	NM_002010 // FGF9 // fibroblast growth factor 9 (glia-activating factor) // 13q11-q12 /	0.00368639	0.215326
7916024	NM_001144832	TTC39A	NM_001144832 // TTC39A // tetratricopeptide repeat domain 39A // 1p32.3 // 22996 /// NM	0.00126775	-0.242022
7992789	NM_016639	TNFRSF12A	NM_016639 // TNFRSF12A // tumor necrosis factor receptor superfamily, member 12A // 16p	0.00637331	-0.207615
8114249	NM_004887	CXCL14	NM_004887 // CXCL14 // chemokine (C-X-C motif) ligand 14 // 5q31 // 9547 /// ENST000003	0.000173636	-0.27429
8143772	NM_002889	RARRES2	NM_002889 // RARRES2 // retinoic acid receptor responder (tazarotene induced) 2 // 7q36	0.000171503	0.281029
8150537	NM_006749	SLC20A2	NM_006749 // SLC20A2 // solute carrier family 20 (phosphate transporter), member 2 // 8	0.00416766	-0.212276
8107094	---		---	0.00916241	-0.200226
8100495	NM_002703	PPAT	NM_002703 // PPAT // phosphoribosyl pyrophosphate amidotransferase // 4q12 // 5471 ///	0.000657032	-0.257553
8053602	NM_002665	PLGLB2	NM_002665 // PLGLB2 // plasminogen-like B2 // 2p11.2 // 5342 /// NM_001032392 // PLGLB1	0.00209353	0.225194

8156199	NM_004938	DAPK1	NM_004938 // DAPK1 // death-associated protein kinase 1 // 9q34.1 // 1612 /// ENST00000	0.000449595	0.253678
8098060	NM_021634	RXFP1	NM_021634 // RXFP1 // relaxin/insulin-like family peptide receptor 1 // 4q32.1 // 59350	0.00436715	0.213642
7944164	NM_019894	TMPRSS4	NM_019894 // TMPRSS4 // transmembrane protease, serine 4 // 11q23.3 // 56649 /// NM_001	0.00755609	-0.199422
7900540	NM_173642	RIMKLA	NM_173642 // RIMKLA // ribosomal modification protein rimK-like family member A // 1p34	0.00493835	0.202819
8060940	NM_012261	C20orf103	NM_012261 // C20orf103 // chromosome 20 open reading frame 103 // 20p12 // 24141 /// EN	0.00304812	-0.214032
8168727	---		---	0.000337173	0.269264
7952004	NM_174934	SCN4B	NM_174934 // SCN4B // sodium channel, voltage-gated, type IV, beta // 11q23.3 // 6330 /	0.00347343	0.216595
7955110	AK125945	DKFZP779L1853	AK125945 // DKFZP779L1853 // hypothetical LOC643162 // 12q13.11 // 643162	8.81E-06	0.326338
8032484	NM_012458	TIMM13	NM_012458 // TIMM13 // translocase of inner mitochondrial membrane 13 homolog (yeast) /	0.00104279	-0.250034
7898112	---		---	0.000173698	0.279044
8146357	NM_005914	MCM4	NM_005914 // MCM4 // minichromosome maintenance complex component 4 // 8q11.2 // 4173 /	0.00611621	-0.204461
8153039	---		---	0.000403632	0.257452
7905938	NM_018845	RAG1AP1	NM_018845 // RAG1AP1 // recombination activating gene 1 activating protein 1 // 1q22 //	4.12E-06	-0.338957
7954653	NM_001080509	TSPAN11	NM_001080509 // TSPAN11 // tetraspanin 11 // 12p11.21 // 441631 /// ENST00000261177 //	0.00734556	-0.194967
7963851	NM_001098815	KIAA0748	NM_001098815 // KIAA0748 // KIAA0748 // 12q13.2 // 9840 /// AB018291 // KIAA0748 // KIA	0.00694507	0.207191
8028908	NM_025194	ITPKC	NM_025194 // ITPKC // inositol 1,4,5-trisphosphate 3-kinase C // 19q13.1 // 80271 /// E	1.48E-07	-0.387454

8070557	NM_001098402	ZNF295	NM_001098402 // ZNF295 // zinc finger protein 295 // 21q22.3 // 49854 /// NM_020727 //	2.40E-07	-0.37344
8155460	NM_033655	CNTNAP3	NM_033655 // CNTNAP3 // contactin associated protein-like 3 // 9p13.1 // 79937 /// AF33	0.000842036	0.248966
8015376	NM_005557	KRT16	NM_005557 // KRT16 // keratin 16 // 17q21.2 // 3868 /// ENST00000301653 // KRT16 // ker	0.00273256	-0.221404
8042503	NM_002357	MXD1	NM_002357 // MXD1 // MAX dimerization protein 1 // 2p13-p12 // 4084 /// ENST00000264444	1.85E-06	-0.357133
8067942	NR_029514	MIR99A	NR_029514 // MIR99A // microRNA 99a // 21q21.1 // 407055	1.48E-06	0.353767
8029280	NM_020406	CD177	NM_020406 // CD177 // CD177 molecule // 19q13.2 // 57126 /// ENST00000457794 // CD177 /	0.00217916	-0.231543
8052413	---		---	1.61E-05	-0.32746
8120043	NM_001024630	RUNX2	NM_001024630 // RUNX2 // runt-related transcription factor 2 // 6p21 // 860 /// NM_0010	0.000471842	-0.25504
7909954	NM_032890	DISP1	NM_032890 // DISP1 // dispatched homolog 1 (Drosophila) // 1q41 // 84976 /// ENST000002	0.00208978	0.228123
7911114	NM_018012	KIF26B	NM_018012 // KIF26B // kinesin family member 26B // 1q44 // 55083 /// ENST00000407071 /	0.000224841	-0.271707
7965226	NM_005447	RASSF9	NM_005447 // RASSF9 // Ras association (RalGDS/AF-6) domain family (N-terminal) member	0.00765212	0.204563
7953835	NM_005810	KLRG1	NM_005810 // KLRG1 // killer cell lectin-like receptor subfamily G, member 1 // 12p13.3	0.00914423	0.200121
7922610	NM_007314	ABL2	NM_007314 // ABL2 // v-abl Abelson murine leukemia viral oncogene homolog 2 // 1q25.2 /	1.90E-08	-0.406607
7964522	NM_000075	CDK4	NM_000075 // CDK4 // cyclin-dependent kinase 4 // 12q14 // 1019 /// ENST00000257904 //	0.00114118	-0.248177
8006812	NM_002795	PSMB3	NM_002795 // PSMB3 // proteasome (prosome, macropain) subunit, beta type, 3 // 17q12 //	0.00726173	-0.205015

8010897	NM_001004431	METRNL	NM_001004431 // METRNL // meteorin, glial cell differentiation regulator-like // 17q25.	0.00451397	-0.217556
7916654	NM_181712	KANK4	NM_181712 // KANK4 // KN motif and ankyrin repeat domains 4 // 1p31.3 // 163782 /// ENS	0.00234314	0.230594
8146863	NM_001128205	SULF1	NM_001128205 // SULF1 // sulfatase 1 // 8q13.1 // 23213 /// NM_015170 // SULF1 // sulfa	0.00169022	-0.227692
8043367	NM_002665	PLGLB2	NM_002665 // PLGLB2 // plasminogen-like B2 // 2p11.2 // 5342 /// NM_001032392 // PLGLB1	0.00589471	0.202059
8029521	NM_001128917	TOMM40	NM_001128917 // TOMM40 // translocase of outer mitochondrial membrane 40 homolog (yeast	0.000232804	-0.274236
8149733	NM_003842	TNFRSF10B	NM_003842 // TNFRSF10B // tumor necrosis factor receptor superfamily, member 10b // 8p2	2.45E-06	-0.34668
8133477	NM_016328	GTF2IRD1	NM_016328 // GTF2IRD1 // GTF2I repeat domain containing 1 // 7q11.23 // 9569 /// NM_005	0.00396622	-0.218764
8026631	NM_003950	F2RL3	NM_003950 // F2RL3 // coagulation factor II (thrombin) receptor-like 3 // 19p12 // 9002	0.000398808	-0.272854
8139776	---		---	0.00472408	0.213726
7942596	NM_001235	SERPINH1	NM_001235 // SERPINH1 // serpin peptidase inhibitor, clade H (heat shock protein 47), m	0.00235863	-0.230706
8050278	NM_005742	PDIA6	NM_005742 // PDIA6 // protein disulfide isomerase family A, member 6 // 2p25.1 // 10130	3.49E-06	-0.335096
8039871	NM_001083539	KIR3DS1	NM_001083539 // KIR3DS1 // killer cell immunoglobulin-like receptor, three domains, sho	0.00174723	0.233856
8077441	NM_003670	BHLHE40	NM_003670 // BHLHE40 // basic helix-loop-helix family, member e40 // 3p26 // 8553 /// E	2.47E-06	-0.351311
8061653	NM_080625	C20orf160	NM_080625 // C20orf160 // chromosome 20 open reading frame 160 // 20q11.2 // 140706 ///	7.90E-05	0.28962
8155345	NR_026801	FAM74A3	NR_026801 // FAM74A3 // family with sequence similarity 74, member A3 // 9p13.1 // 7284	0.00298401	0.219479
8078138	NM_033083	EAF1	NM_033083 // EAF1 // ELL associated factor 1 // 3p25.1 // 85403 /// ENST00000396842 //	5.02E-05	-0.297982

7987066	---		---	0.00677654	0.207181
8084206	NM_032047	B3GNT5	NM_032047 // B3GNT5 // UDP-GlcNAc:betaGal beta-1,3-N-acetylglucosaminyltransferase 5 //	0.0037717	-0.220416
8043504	NM_002371	MAL	NM_002371 // MAL // mal, T-cell differentiation protein // 2cen-q13 // 4118 /// NM_0224	0.000154753	0.28636
8140668	NM_006080	SEMA3A	NM_006080 // SEMA3A // sema domain, immunoglobulin domain (Ig), short basic domain, sec	0.000292328	-0.265907
8135774	NM_002851	PTPRZ1	NM_002851 // PTPRZ1 // protein tyrosine phosphatase, receptor-type, Z polypeptide 1 //	0.000984055	-0.245647
8066513	NM_002999	SDC4	NM_002999 // SDC4 // syndecan 4 // 20q12 // 6385 /// ENST00000372733 // SDC4 // syndeca	0.000100908	-0.288639
7906400	NM_005531	IFI16	NM_005531 // IFI16 // interferon, gamma-inducible protein 16 // 1q22 // 3428 /// ENST00	0.00082096	-0.243129
7949577	NM_020470	YIF1A	NM_020470 // YIF1A // Yip1 interacting factor homolog A (S. cerevisiae) // 11q13 // 108	0.000672064	-0.261544
8027621	NM_000175	GPI	NM_000175 // GPI // glucose-6-phosphate isomerase // 19q13.1 // 2821 /// NM_001184722 /	0.0044991	-0.213992
7981068	NM_001002236	SERPINA1	NM_001002236 // SERPINA1 // serpin peptidase inhibitor, clade A (alpha-1 antiproteinase	0.00157482	-0.234667
7986329	NM_021005	NR2F2	NM_021005 // NR2F2 // nuclear receptor subfamily 2, group F, member 2 // 15q26 // 7026	0.000664809	0.261882
8110672	NM_001005277	OR4F16	NM_001005277 // OR4F16 // olfactory receptor, family 4, subfamily F, member 16 // 1p36.	8.12E-05	0.287326
7896744	NM_001005277	OR4F16	NM_001005277 // OR4F16 // olfactory receptor, family 4, subfamily F, member 16 // 1p36.	8.12E-05	0.287326
7911345	NM_001005277	OR4F16	NM_001005277 // OR4F16 // olfactory receptor, family 4, subfamily F, member 16 // 1p36.	8.12E-05	0.287326
8113641	NM_001801	CDO1	NM_001801 // CDO1 // cysteine dioxygenase, type I // 5q23.2 // 1036 /// ENST00000250535	0.00421525	0.216467
8096116	NM_032717	AGPAT9	NM_032717 // AGPAT9 // 1-acylglycerol-3-phosphate O-acyltransferase 9 // 4q21.23 // 848	0.00435662	-0.217366

7955179	NM_032704	TUBA1C	NM_032704 // TUBA1C // tubulin, alpha 1c // 12q13.12 // 84790 /// ENST00000301072 // TU	0.00251314	-0.233122
8156373	NM_001083536	FGD3	NM_001083536 // FGD3 // FYVE, RhoGEF and PH domain containing 3 // --- // 89846 /// NM_	0.000451153	0.259115
7951030	NR_003036	SNORD6	NR_003036 // SNORD6 // small nucleolar RNA, C/D box 6 // 11q21 // 692075	0.00157637	-0.236835
8054872	NM_014553	TFCP2L1	NM_014553 // TFCP2L1 // transcription factor CP2-like 1 // 2q14 // 29842 /// ENST000002	0.00803877	-0.201003
7989501	NM_001218	CA12	NM_001218 // CA12 // carbonic anhydrase XII // 15q22 // 771 /// NM_206925 // CA12 // ca	0.00047616	-0.26669
8035787	---		---	0.00274291	0.225184
7951838	NM_003904	ZNF259	NM_003904 // ZNF259 // zinc finger protein 259 // 11q23.3 // 8882 /// ENST00000227322 /	0.00323733	-0.227073
8114300	NM_017415	KLHL3	NM_017415 // KLHL3 // kelch-like 3 (Drosophila) // 5q31 // 26249 /// ENST00000309755 //	0.0085817	0.20062
7917850	NM_004815	ARHGAP29	NM_004815 // ARHGAP29 // Rho GTPase activating protein 29 // 1p22.1 // 9411 /// ENST000	0.000302559	0.26738
8104234	NM_004237	TRIP13	NM_004237 // TRIP13 // thyroid hormone receptor interactor 13 // 5p15.33 // 9319 /// NM	0.00154195	-0.236118
8148962	NM_001005504	OR4F21	NM_001005504 // OR4F21 // olfactory receptor, family 4, subfamily F, member 21 // 8p23.	9.98E-05	0.284242
8090193	NM_020733	HEG1	NM_020733 // HEG1 // HEG homolog 1 (zebrafish) // 3q21.2 // 57493 /// ENST00000311127 /	0.00726051	0.203227
8115584	NM_024565	CCNJL	NM_024565 // CCNJL // cyclin J-like // 5q33.3 // 79616 /// ENST00000393977 // CCNJL //	0.000121125	-0.295708
8138592	AB052759	TRA2A	AB052759 // TRA2A // transformer 2 alpha homolog (Drosophila) // 7p15.3 // 29896 /// AK	0.0087539	0.196534
8079740	AY078383	STGC3	AY078383 // STGC3 // hypothetical STGC3 // 3p21 // 474171	0.000460444	0.261372
7910427	NM_004481	GALNT2	NM_004481 // GALNT2 // UDP-N-acetyl-alpha-D-galactosamine:polypeptide N-acetylgalactosa	3.23E-07	-0.369525

7921332	NM_005894	CD5L	NM_005894 // CD5L // CD5 molecule-like // 1q21-q23 // 922 /// ENST00000368174 // CD5L /	0.00224478	0.218654
8097461	NM_012118	CCRN4L	NM_012118 // CCRN4L // CCR4 carbon catabolite repression 4-like (S. cerevisiae) // 4q31	7.47E-08	-0.388576
8097955	---		---	0.00132223	0.238166
8095751	NM_015393	PARM1	NM_015393 // PARM1 // prostate androgen-regulated mucin-like protein 1 // 4q13.3-q21.3	0.0012201	-0.240894
8009517	NM_000346	SOX9	NM_000346 // SOX9 // SRY (sex determining region Y)-box 9 // 17q23 // 6662 /// ENST0000	0.00325917	-0.217114
8031311	NM_013289	KIR3DL1	NM_013289 // KIR3DL1 // killer cell immunoglobulin-like receptor, three domains, long c	0.00144651	0.237831
8111925	NM_001014279	C5orf39	NM_001014279 // C5orf39 // chromosome 5 open reading frame 39 // 5p12 // 389289 /// ENS	0.000372185	0.270607
8027674	NM_018443	ZNF302	NM_018443 // ZNF302 // zinc finger protein 302 // 19q13.11 // 55900 /// NM_001012320 //	1.23E-05	0.331077
7951259	NM_002425	MMP10	NM_002425 // MMP10 // matrix metallopeptidase 10 (stromelysin 2) // 11q22.3 // 4319 ///	4.07E-05	-0.301415
8120992	NM_015021	ZNF292	NM_015021 // ZNF292 // zinc finger protein 292 // 6q14.3 // 23036 /// ENST00000369577 /	0.00541573	0.213842
8113800	NM_001999	FBN2	NM_001999 // FBN2 // fibrillin 2 // 5q23-q31 // 2201 /// ENST00000262464 // FBN2 // fib	0.00819604	-0.199915
7999384	---		---	0.000521753	0.256906
8077160	NM_000487	ARSA	NM_000487 // ARSA // arylsulfatase A // 22q13.31-qter|22q13.33 // 410 /// NM_001085425	0.00598367	-0.213007
8067140	NM_000782	CYP24A1	NM_000782 // CYP24A1 // cytochrome P450, family 24, subfamily A, polypeptide 1 // 20q13	0.00204025	-0.229322
8026339	NM_003096	SNRPG	NM_003096 // SNRPG // small nuclear ribonucleoprotein polypeptide G // 2p13.3 // 6637 /	0.00848712	-0.202918

7981960	NR_003321	SNORD116-6	NR_003321 // SNORD116-6 // small nucleolar RNA, C/D box 116-6 // 15q11.2 // 100033418	0.00017891	0.279346
8170468	NM_005342	HMGB3	NM_005342 // HMGB3 // high-mobility group box 3 // Xq28 // 3149 /// ENST00000325307 //	0.000313041	-0.267225
8142930	NM_018718	TSGA14	NM_018718 // TSGA14 // testis specific, 14 // 7q32 // 95681 /// ENST00000223208 // TSGA	0.00669084	-0.204243
7934442	NM_001114133	SYNPO2L	NM_001114133 // SYNPO2L // synaptopodin 2-like // 10q22.2 // 79933 /// NM_024875 // SYN	0.00181632	0.235107
8021603	NM_012397	SERPINB13	NM_012397 // SERPINB13 // serpin peptidase inhibitor, clade B (ovalbumin), member 13 //	0.00274326	-0.226156
7907861	NM_004736	XPR1	NM_004736 // XPR1 // xenotropic and polytropic retrovirus receptor 1 // 1q25.1 // 9213	0.00068825	-0.255779
7981215	---		---	0.00105059	0.243121
7928429	NM_002658	PLAU	NM_002658 // PLAU // plasminogen activator, urokinase // 10q24 // 5328 /// NM_001145031	0.00131483	-0.243851
7900922	NM_004047	ATP6V0B	NM_004047 // ATP6V0B // ATPase, H+ transporting, lysosomal 21kDa, V0 subunit b // 1p32.	0.000648713	-0.25753
7954104	NM_018179	ATF7IP	NM_018179 // ATF7IP // activating transcription factor 7 interacting protein // 12p13.1	0.00890913	0.202122
7906969	---		---	1.89E-05	0.307167
8077299	NM_014461	CNTN6	NM_014461 // CNTN6 // contactin 6 // 3p26-p25 // 27255 /// ENST00000446702 // CNTN6 //	2.24E-05	0.309345
8163063	NM_003798	CTNNAL1	NM_003798 // CTNNAL1 // catenin (cadherin-associated protein), alpha-like 1 // 9q31.2 /	0.000196928	0.280721
7918634	NM_006608	PHTF1	NM_006608 // PHTF1 // putative homeodomain transcription factor 1 // 1p13 // 10745 ///	0.00456189	-0.212255
8022145	NM_173464	L3MBTL4	NM_173464 // L3MBTL4 // l(3)mbt-like 4 (Drosophila) // 18p11.31 // 91133 /// ENST000002	0.000611578	0.253787
8098214	NM_012464	TLL1	NM_012464 // TLL1 // tolloid-like 1 // 4q32-q33 // 7092 /// ENST00000061240 // TLL1 //	0.00998145	0.192243

8120468	NM_001704	BAI3	NM_001704 // BAI3 // brain-specific angiogenesis inhibitor 3 // 6q12 // 577 /// ENST000	0.00102095	0.247383
8013519	---		---	0.000231744	0.269076
7998233	NM_021259	TMEM8A	NM_021259 // TMEM8A // transmembrane protein 8A // 16p13.3 // 58986 /// ENST00000431232	0.00080993	-0.251538
7899462	NM_001048194	RCC1	NM_001048194 // RCC1 // regulator of chromosome condensation 1 // 1p36.1 // 1104 /// NR	1.41E-07	-0.390359
7905571	NM_002965	S100A9	NM_002965 // S100A9 // S100 calcium binding protein A9 // 1q21 // 6280 /// ENST00000368	0.000790072	-0.258451
8152617	NM_005328	HAS2	NM_005328 // HAS2 // hyaluronan synthase 2 // 8q24.12 // 3037 /// ENST00000303924 // HA	2.58E-05	-0.297983
8033257	NM_000064	C3	NM_000064 // C3 // complement component 3 // 19p13.3-p13.2 // 718 /// ENST00000245907 /	0.00200578	-0.235574
7916984	NR_029707	MIR186	NR_029707 // MIR186 // microRNA 186 // 1p31.1 // 406962	0.00148275	0.239371
8162631	NM_014930	ZNF510	NM_014930 // ZNF510 // zinc finger protein 510 // 9q22.33 // 22869 /// ENST00000375231	0.00102019	0.249341
7907370	NM_015569	DNM3	NM_015569 // DNM3 // dynamin 3 // 1q24.3 // 26052 /// NM_001136127 // DNM3 // dynamin 3	0.0076431	0.200761
7914563	NM_003680	YARS	NM_003680 // YARS // tyrosyl-tRNA synthetase // 1p35.1 // 8565 /// ENST00000373477 // Y	5.91E-06	-0.336666
8097586	NM_207123	GAB1	NM_207123 // GAB1 // GRB2-associated binding protein 1 // 4q31.21 // 2549 /// NM_002039	1.90E-05	0.314119
7909027	NM_001005388	NFASC	NM_001005388 // NFASC // neurofascin // 1q32.1 // 23114 /// NM_001160332 // NFASC // ne	0.00932709	0.198561
8083000	NM_001033030	FAIM	NM_001033030 // FAIM // Fas apoptotic inhibitory molecule // 3q22.3 // 55179 /// NM_001	0.00828157	-0.197807
8042310	NM_003038	SLC1A4	NM_003038 // SLC1A4 // solute carrier family 1 (glutamate/neutral amino acid transporte	0.00143697	-0.23839

8146328	---		---	0.00252482	0.225511
8162276	NM_005384	NFIL3	NM_005384 // NFIL3 // nuclear factor, interleukin 3 regulated // 9q22 // 4783 /// ENST0	3.77E-07	-0.378817
8171066	NR_028269	LOC100288778	NR_028269 // LOC100288778 // WAS protein family homolog 1 pseudogene // 12p13.33 // 100	0.00121043	0.238266
8002041	NM_004691	ATP6V0D1	NM_004691 // ATP6V0D1 // ATPase, H+ transporting, lysosomal 38kDa, V0 subunit d1 // 16q	0.000430496	-0.262074
7993148	NM_000303	PMM2	NM_000303 // PMM2 // phosphomannomutase 2 // 16p13 // 5373 /// ENST00000268261 // PMM2	0.000220542	-0.276886
8140478	NM_017439	PION	NM_017439 // PION // pigeon homolog (Drosophila) // 7q11.23 // 54103 /// ENST0000025762	0.00912896	0.191353
7973936	NM_002791	PSMA6	NM_002791 // PSMA6 // proteasome (prosome, macropain) subunit, alpha type, 6 // 14q13 /	0.00602405	-0.21099
8110631	NM_152547	BTNL9	NM_152547 // BTNL9 // butyrophilin-like 9 // 5q35.3 // 153579 /// ENST00000327705 // BT	0.00283536	0.220726
7927876	NM_030625	TET1	NM_030625 // TET1 // tet oncogene 1 // 10q21 // 80312 /// ENST00000373644 // TET1 // te	0.00771089	0.203218
8097388	NM_018078	LARP1B	NM_018078 // LARP1B // La ribonucleoprotein domain family, member 1B // 4q28.2 // 55132	0.00399607	-0.215045
8044793	NM_182915	STEAP3	NM_182915 // STEAP3 // STEAP family member 3 // 2q14.2 // 55240 /// NM_018234 // STEAP3	0.000120219	-0.284405
7981335	NM_001017963	HSP90AA1	NM_001017963 // HSP90AA1 // heat shock protein 90kDa alpha (cytosolic), class A member	0.00538133	-0.209292
7932826	NM_020848	KIAA1462	NM_020848 // KIAA1462 // KIAA1462 // 10p11.23 // 57608 /// ENST00000375377 // KIAA1462	0.0100986	0.193719
8037322	NM_014297	ETHE1	NM_014297 // ETHE1 // ethylmalonic encephalopathy 1 // 19q13.31 // 23474 /// ENST000002	0.00964128	-0.198916

8119161	NM_002648	PIM1	NM_002648 // PIM1 // pim-1 oncogene // 6p21.2 // 5292 /// ENST00000373509 // PIM1 // pi	1.08E-05	-0.329256
7919193	NR_002212	NUDT4P1	NR_002212 // NUDT4P1 // nudix (nucleoside diphosphate linked moiety X)-type motif 4 pse	0.00337442	-0.216066
8113122	---		---	0.000162126	0.27585
8174975	---		---	0.000247426	0.277346
8082408	NM_013336	SEC61A1	NM_013336 // SEC61A1 // Sec61 alpha 1 subunit (S. cerevisiae) // 3q21.3 // 29927 /// EN	4.95E-07	-0.366651
8161288	NM_033655	CNTNAP3	NM_033655 // CNTNAP3 // contactin associated protein-like 3 // 9p13.1 // 79937 /// AF33	0.000757898	0.250797
8138363	NM_015464	SOSTDC1	NM_015464 // SOSTDC1 // sclerostin domain containing 1 // 7p21.1 // 25928 /// ENST00000	0.00445932	0.216209
7971104	NM_016179	TRPC4	NM_016179 // TRPC4 // transient receptor potential cation channel, subfamily C, member	0.00383487	-0.216329
7952145	NM_006389	HYOU1	NM_006389 // HYOU1 // hypoxia up-regulated 1 // 11q23.1-q23.3 // 10525 /// NM_001130991	1.11E-05	-0.321231
8155388	NR_026801	FAM74A3	NR_026801 // FAM74A3 // family with sequence similarity 74, member A3 // 9p13.1 // 7284	0.0010927	0.242917
8152988	NM_001045556	SLA	NM_001045556 // SLA // Src-like-adaptor // 8q22.3-qter|8q24 // 6503 /// NM_006748 // SL	0.00328772	-0.224826
8077270	NM_006614	CHL1	NM_006614 // CHL1 // cell adhesion molecule with homology to L1CAM (close homolog of L1	8.12E-05	-0.292516
8092218	---		---	0.00957516	0.195311
7994280	NM_000418	IL4R	NM_000418 // IL4R // interleukin 4 receptor // 16p12.1-p11.2 // 3566 /// NM_001008699 /	1.23E-06	-0.36848
8013517	---		---	0.000472272	0.255192
8124756	NM_002714	PPP1R10	NM_002714 // PPP1R10 // protein phosphatase 1, regulatory (inhibitor) subunit 10 // 6p2	0.00763813	-0.204847
8178358	NM_002714	PPP1R10	NM_002714 // PPP1R10 // protein phosphatase 1, regulatory (inhibitor) subunit 10 // 6p2	0.00763813	-0.204847
8179664	NM_002714	PPP1R10	NM_002714 // PPP1R10 // protein phosphatase 1, regulatory (inhibitor) subunit 10 // 6p2	0.00763813	-0.204847
8136658	---		---	0.000121873	0.277834

7898809	NM_017449	EPHB2	NM_017449 // EPHB2 // EPH receptor B2 // 1p36.1-p35 // 2048 /// NM_004442 // EPHB2 // E	0.00553712	-0.206409
8020903	NM_020474	GALNT1	NM_020474 // GALNT1 // UDP-N-acetyl-alpha-D-galactosamine:polypeptide N-acetylgalactosa	0.000154912	-0.277376
8055202	---		---	0.000688847	0.251439
7956401	NM_005412	SHMT2	NM_005412 // SHMT2 // serine hydroxymethyltransferase 2 (mitochondrial) // 12q12-q14 //	4.06E-05	-0.307326
8127370	NM_152688	KHDRBS2	NM_152688 // KHDRBS2 // KH domain containing, RNA binding, signal transduction associat	0.00071337	0.253201
8120431	NM_003463	PTP4A1	NM_003463 // PTP4A1 // protein tyrosine phosphatase type IVA, member 1 // 6q12 // 7803	0.000483666	-0.265592
8069943	NM_014825	URB1	NM_014825 // URB1 // URB1 ribosome biogenesis 1 homolog (S. cerevisiae) // 21q22.11 //	9.05E-05	-0.287093
7957126	NM_014505	KCNMB4	NM_014505 // KCNMB4 // potassium large conductance calcium-activated channel, subfamily	0.00240485	0.23161
7930894	NM_005308	GRK5	NM_005308 // GRK5 // G protein-coupled receptor kinase 5 // 10q26.11 // 2869 /// ENST00	0.00389898	0.217623
7905088	NM_003517	HIST2H2AC	NM_003517 // HIST2H2AC // histone cluster 2, H2ac // 1q21.2 // 8338 /// ENST00000331380	0.000163243	0.276383
8020806	NM_017831	RNF125	NM_017831 // RNF125 // ring finger protein 125 // 18q12.1 // 54941 /// ENST00000217740	0.00212604	0.228589
7926979	---		---	7.51E-06	0.326795
7940333	NM_001098835	MS4A15	NM_001098835 // MS4A15 // membrane-spanning 4-domains, subfamily A, member 15 // 11q12.	0.00156613	0.234413
8135488	NM_001099660	LRRN3	NM_001099660 // LRRN3 // leucine rich repeat neuronal 3 // 7q31.1 // 54674 /// NM_00109	0.00118092	0.245129
7978201	NM_006156	NEDD8	NM_006156 // NEDD8 // neural precursor cell expressed, developmentally down-regulated 8	0.00312099	-0.229904

7996759	---		---	0.000331308	0.268485
8174253	NM_001142418	MORF4L2	NM_001142418 // MORF4L2 // mortality factor 4 like 2 // Xq22 // 9643 /// NM_001142422 /	0.00564505	-0.204364
7970426	---		---	0.00731491	0.204874
8141263	NM_001145715	KPNA7	NM_001145715 // KPNA7 // karyopherin alpha 7 (importin alpha 8) // 7q22.1 // 402569 ///	0.00190958	-0.232562
7922229	NM_000450	SELE	NM_000450 // SELE // selectin E // 1q22-q25 // 6401 /// ENST00000333360 // SELE // sele	4.06E-06	-0.331939
8129071	NM_002031	FRK	NM_002031 // FRK // fyn-related kinase // 6q21-q22.3 // 2444 /// ENST00000368626 // FRK	0.005147	-0.206397
8052845	NM_022173	TIA1	NM_022173 // TIA1 // TIA1 cytotoxic granule-associated RNA binding protein // 2p13 // 7	0.00804783	0.20389
7961546	NM_004447	EPS8	NM_004447 // EPS8 // epidermal growth factor receptor pathway substrate 8 // 12p12.3 //	0.000402847	-0.263061
8156848	NM_006981	NR4A3	NM_006981 // NR4A3 // nuclear receptor subfamily 4, group A, member 3 // 9q22 // 8013 /	9.02E-05	-0.293525
8074780	NM_013313	YPEL1	NM_013313 // YPEL1 // yippee-like 1 (Drosophila) // 22q11.2 // 29799 /// NM_148175 // P	0.00129228	0.236407
8043375	---		---	2.17E-05	0.309566
7982366	NM_001144757	SCG5	NM_001144757 // SCG5 // secretogranin V (7B2 protein) // 15q13-q14 // 6447 /// NM_00302	0.000107925	-0.27896
8104621	NR_027028	GUSBP1	NR_027028 // GUSBP1 // glucuronidase, beta pseudogene 1 // 5p14.3 // 728411 /// NR_0036	5.12E-06	0.332507
7924817	BC019830	PRO2012	BC019830 // PRO2012 // hypothetical protein PRO2012 // 1q42.13 // 55478	4.16E-05	0.30122
8156309	NM_006705	GADD45G	NM_006705 // GADD45G // growth arrest and DNA-damage-inducible, gamma // 9q22.1-q22.2 /	0.000450516	-0.271842
7898405	---		---	0.00342157	0.22587
7912806	---		---	0.00342157	0.22587
8012126	NM_001307	CLDN7	NM_001307 // CLDN7 // claudin 7 // 17p13 // 1366 /// NM_001185022 // CLDN7 // claudin 7	0.00563544	-0.213806

7908496	NM_005666	CFHR2	NM_005666 // CFHR2 // complement factor H-related 2 // 1q31.3 // 3080 /// ENST000003674	0.00351248	-0.213056
8173607	---		---	0.000649864	0.251541
8175195	NM_001077188	HS6ST2	NM_001077188 // HS6ST2 // heparan sulfate 6-O-sulfotransferase 2 // Xq26.2 // 90161 ///	0.00264457	-0.224572
7954559	NM_003622	PPFIBP1	NM_003622 // PPFIBP1 // PTPRF interacting protein, binding protein 1 (liprin beta 1) //	8.92E-05	0.29142
7937913	---		---	0.000853166	0.255323
8135594	NM_001753	CAV1	NM_001753 // CAV1 // caveolin 1, caveolae protein, 22kDa // 7q31.1 // 857 /// NM_001172	0.000609376	0.257511
8162283	NM_004560	ROR2	NM_004560 // ROR2 // receptor tyrosine kinase-like orphan receptor 2 // 9q22 // 4920 //	0.000169821	-0.275686
7949532	NM_005438	FOSL1	NM_005438 // FOSL1 // FOS-like antigen 1 // 11q13 // 8061 /// ENST00000312562 // FOSL1	0.00141258	-0.242894
8048782	NM_000091	COL4A3	NM_000091 // COL4A3 // collagen, type IV, alpha 3 (Goodpasture antigen) // 2q36-q37 //	0.00134332	0.242545
8109612	NM_000679	ADRA1B	NM_000679 // ADRA1B // adrenergic, alpha-1B-, receptor // 5q33.3 // 147 /// ENST0000030	0.00356151	0.216757
8131179	NM_025250	TTYH3	NM_025250 // TTYH3 // tweety homolog 3 (Drosophila) // 7p22 // 80727 /// ENST0000025879	0.00113036	-0.24182
7910186	---		---	0.00695073	0.201655
8155665	NM_021965	PGM5	NM_021965 // PGM5 // phosphoglucomutase 5 // 9q13 // 5239 /// ENST00000396396 // PGM5 /	0.00967286	0.197862
8079598	NM_016089	ZNF589	NM_016089 // ZNF589 // zinc finger protein 589 // 3p21 // 51385 /// ENST00000448461 //	0.00597483	0.200579
8146957	NM_015886	PI15	NM_015886 // PI15 // peptidase inhibitor 15 // 8q21.11 // 51050 /// ENST00000260113 //	3.71E-08	-0.38585
7936856	NM_015892	CHST15	NM_015892 // CHST15 // carbohydrate (N-acetylgalactosamine 4-sulfate 6-O) sulfotransfer	0.00340876	-0.218584
8045287	---		---	0.000103315	0.287044
7921677	NM_016382	CD244	NM_016382 // CD244 // CD244 molecule, natural killer cell receptor 2B4 // 1q23.3 // 517	0.0003269	0.266472

8158424	NM_001127244	LRRC8A	NM_001127244 // LRRC8A // leucine rich repeat containing 8 family, member A // 9q34.11	8.86E-06	-0.333881
8033809	NM_001077624	ZNF846	NM_001077624 // ZNF846 // zinc finger protein 846 // 19p13.2 // 162993 /// ENST00000397	0.00198927	0.235534
8149315	---		---	0.0005915	0.254681
7961291	NM_176885	TAS2R31	NM_176885 // TAS2R31 // taste receptor, type 2, member 31 // 12p13.2 // 259290 /// BC11	0.00900155	0.19944
8171297	NM_000381	MID1	NM_000381 // MID1 // midline 1 (Opitz/BBB syndrome) // Xp22 // 4281 /// NM_033290 // MI	0.00642852	-0.204356
7950391	NM_173582	PGM2L1	NM_173582 // PGM2L1 // phosphoglucomutase 2-like 1 // 11q13.4 // 283209 /// ENST0000029	0.00057407	-0.255327
8059720	NM_006056	NMUR1	NM_006056 // NMUR1 // neuromedin U receptor 1 // 2q37.1 // 10316 /// ENST00000305141 //	0.000394658	0.259321
7954645	NM_001080509	TSPAN11	NM_001080509 // TSPAN11 // tetraspanin 11 // 12p11.21 // 441631 /// ENST00000261177 //	0.00030005	-0.263137
8077198	NR_033856	FLJ43315	NR_033856 // FLJ43315 // asparagine synthetase pseudogene // --- // 644316 /// BC057848	0.00656249	0.208063
8029784	NM_152794	HIF3A	NM_152794 // HIF3A // hypoxia inducible factor 3, alpha subunit // 19q13.32 // 64344 //	0.00066469	0.255357
8136654	---		---	3.24E-05	0.302115
8113726	NM_000943	PPIC	NM_000943 // PPIC // peptidylprolyl isomerase C (cyclophilin C) // 5q23.2 // 5480 /// E	0.00355837	-0.214242
8133876	NM_001001548	CD36	NM_001001548 // CD36 // CD36 molecule (thrombospondin receptor) // 7q11.2 // 948 /// NM	0.00712602	0.199133
8115884	---		---	0.00117455	0.244813
8149885	NM_000680	ADRA1A	NM_000680 // ADRA1A // adrenergic, alpha-1A-, receptor // 8p21.2 // 148 /// NM_033303 /	5.87E-07	0.359608
7912706	NM_004431	EPHA2	NM_004431 // EPHA2 // EPH receptor A2 // 1p36 // 1969 /// ENST00000407976 // EPHA2 // E	0.000798929	-0.24651
8055688	NM_005168	RND3	NM_005168 // RND3 // Rho family GTPase 3 // 2q23.3 // 390 /// ENST00000375734 // RND3 /	0.0056022	-0.201493

8170882	NM_001183	ATP6AP1	NM_001183 // ATP6AP1 // ATPase, H+ transporting, lysosomal accessory protein 1 // Xq28	0.00180192	-0.226065
8112560	NR_034021	SMA5	NR_034021 // SMA5 // glucuronidase, beta pseudogene // 5q13 // 11042 /// AK289851 // SM	4.81E-06	0.334364
8154962	NM_001135004	DNAJB5	NM_001135004 // DNAJB5 // DnaJ (Hsp40) homolog, subfamily B, member 5 // 9p13.3 // 2582	8.38E-06	-0.323037
8165667	---		---	2.71E-05	0.306304
7896752	---		---	2.71E-05	0.306304
8053775	NM_032788	ZNF514	NM_032788 // ZNF514 // zinc finger protein 514 // 2q11.1 // 84874 /// ENST00000411425 /	0.00288905	0.231435
7974214	NM_172193	KLHDC1	NM_172193 // KLHDC1 // kelch domain containing 1 // 14q21.3 // 122773 /// ENST000003593	0.00299428	0.224161
7896754	AK290103	LOC100287934	AK290103 // LOC100287934 // hypothetical LOC100287934 // 1p36.33 // 100287934 /// ENST0	0.00271892	0.227393
7953892	NM_016523	KLRF1	NM_016523 // KLRF1 // killer cell lectin-like receptor subfamily F, member 1 // 12p13.3	0.00159719	0.238206
8164165	NM_005347	HSPA5	NM_005347 // HSPA5 // heat shock 70kDa protein 5 (glucose-regulated protein, 78kDa) //	5.75E-05	-0.302656
8121130	---		---	0.0072309	0.204523
8063458	NM_018431	DOK5	NM_018431 // DOK5 // docking protein 5 // 20q13.2 // 55816 /// ENST00000262593 // DOK5	0.00551461	-0.205414
7983630	NM_002009	FGF7	NM_002009 // FGF7 // fibroblast growth factor 7 // 15q21.2 // 2252 /// M60828 // FGF7 /	0.00979987	-0.192084
7913216	NM_000300	PLA2G2A	NM_000300 // PLA2G2A // phospholipase A2, group IIA (platelets, synovial fluid) // 1p35	0.00859002	-0.20103
7952451	BC040288	LOC100130428	BC040288 // LOC100130428 // IGYY565 // 11q24.2 // 100130428	0.00890759	0.198307
8105991	BT006760	GUSBP3	BT006760 // GUSBP3 // glucuronidase, beta pseudogene 3 // 5q13.2 // 653188 /// AK315849	5.07E-06	0.333907

7953321	NM_002342	LTBR	NM_002342 // LTBR // lymphotoxin beta receptor (TNFR superfamily, member 3) // 12p13 //	0.000111075	-0.292325
7985488	BC112329	FAM103A1	BC112329 // FAM103A1 // family with sequence similarity 103, member A1 // 15q25.2 // 83	0.00931241	-0.20273
7909371	NM_000573	CR1	NM_000573 // CR1 // complement component (3b/4b) receptor 1 (Knops blood group) // 1q32	4.44E-05	-0.305145
8178115	NM_001710	CFB	NM_001710 // CFB // complement factor B // 6p21.3 // 629 /// ENST00000417261 // CFB //	0.000195703	-0.280012
7934997	NM_005398	PPP1R3C	NM_005398 // PPP1R3C // protein phosphatase 1, regulatory (inhibitor) subunit 3C // 10q	0.000555216	0.265609
8004671	NM_001080424	KDM6B	NM_001080424 // KDM6B // lysine (K)-specific demethylase 6B // 17p13.1 // 23135 /// ENS	0.00174172	-0.24024
8121416	---		---	1.42E-05	0.330916
8011093	NM_152346	SLC43A2	NM_152346 // SLC43A2 // solute carrier family 43, member 2 // 17p13.3 // 124935 /// ENS	0.0030782	-0.225289
7935425	NM_015179	RRP12	NM_015179 // RRP12 // ribosomal RNA processing 12 homolog (S. cerevisiae) // 10q24.1 //	1.64E-06	-0.358624
7969058	---		---	0.00624304	0.210947
8160297	NM_001122	PLIN2	NM_001122 // PLIN2 // perilipin 2 // 9p22.1 // 123 /// ENST00000276914 // PLIN2 // peri	0.000312318	-0.272314
8091637	NM_004733	SLC33A1	NM_004733 // SLC33A1 // solute carrier family 33 (acetyl-CoA transporter), member 1 //	0.00117223	-0.23958
7921955	NM_006917	RXRG	NM_006917 // RXRG // retinoid X receptor, gamma // 1q22-q23 // 6258 /// NR_033824 // RX	0.00449296	0.216334
7909990	AK290103	LOC100287934	AK290103 // LOC100287934 // hypothetical LOC100287934 // 1p36.33 // 100287934 /// ENST0	0.00217855	0.232054
7970810	NM_003045	SLC7A1	NM_003045 // SLC7A1 // solute carrier family 7 (cationic amino acid transporter, y+ sys	1.05E-06	-0.352067
8041048	NM_005253	FOSL2	NM_005253 // FOSL2 // FOS-like antigen 2 // 2p23.3 // 2355 /// ENST00000264716 // FOSL2	2.57E-08	-0.403668

7923907	NM_000572	IL10	NM_000572 // IL10 // interleukin 10 // 1q31-q32 // 3586 /// ENST00000423557 // IL10 //	0.00242591	-0.233023
8118345	NM_001710	CFB	NM_001710 // CFB // complement factor B // 6p21.3 // 629 /// ENST00000425368 // CFB //	0.000286258	-0.272913
7914603	NM_153341	RNF19B	NM_153341 // RNF19B // ring finger protein 19B // 1p35.1 // 127544 /// NM_001127361 //	7.48E-05	-0.29902
7932407	NM_001004470	ST8SIA6	NM_001004470 // ST8SIA6 // ST8 alpha-N-acetyl-neuraminide alpha-2,8-sialyltransferase 6	6.75E-05	0.293481
7942613	NM_032564	DGAT2	NM_032564 // DGAT2 // diacylglycerol O-acyltransferase 2 // 11q13.5 // 84649 /// ENST00	0.00818582	-0.205175
8157828	NM_030978	ARPC5L	NM_030978 // ARPC5L // actin related protein 2/3 complex, subunit 5-like // 9q33.3 // 8	0.000857595	-0.258081
8112053	NM_152623	CDC20B	NM_152623 // CDC20B // cell division cycle 20 homolog B (S. cerevisiae) // 5q11.2 // 16	0.0081387	-0.200723
8169419	NM_001099922	ALG13	NM_001099922 // ALG13 // asparagine-linked glycosylation 13 homolog (S. cerevisiae) //	0.00314747	0.220401
8134030	NM_012449	STEAP1	NM_012449 // STEAP1 // six transmembrane epithelial antigen of the prostate 1 // 7q21 /	0.00419148	-0.214331
8009526	NM_001050	SSTR2	NM_001050 // SSTR2 // somatostatin receptor 2 // 17q24 // 6752 /// ENST00000357585 // S	0.00321269	-0.222646
8165703	AF284753	UIMC1	AF284753 // UIMC1 // ubiquitin interaction motif containing 1 // 5q35.2 // 51720	2.40E-06	0.34396
7911343	AF284753	UIMC1	AF284753 // UIMC1 // ubiquitin interaction motif containing 1 // 5q35.2 // 51720	2.40E-06	0.34396
8092849	NM_024524	ATP13A3	NM_024524 // ATP13A3 // ATPase type 13A3 // 3q29 // 79572 /// ENST00000256031 // ATP13A	1.22E-08	-0.398849
8086799	NM_006574	CSPG5	NM_006574 // CSPG5 // chondroitin sulfate proteoglycan 5 (neuroglycan C) // 3p21.3 // 1	0.00306908	-0.228257
8021470	NM_021127	PMAIP1	NM_021127 // PMAIP1 // phorbol-12-myristate-13-acetate-induced protein 1 // 18q21.32 //	0.00266535	-0.227721
8149161	NR_027000	LOC349196	NR_027000 // LOC349196 // hypothetical LOC349196 // 8p23.1 // 349196 /// AK094835 // LO	0.0018375	0.236272

8149210	NR_027000	LOC349196	NR_027000 // LOC349196 // hypothetical LOC349196 // 8p23.1 // 349196 /// AK094835 // LO	0.0018375	0.236272
7923332	NM_000364	TNNT2	NM_000364 // TNNT2 // troponin T type 2 (cardiac) // 1q32 // 7139 /// NM_001001430 // T	0.00125558	0.242306
8094184	NM_001135170	C1QTNF7	NM_001135170 // C1QTNF7 // C1q and tumor necrosis factor related protein 7 // 4p15.3 //	0.00642302	0.208123
8117594	NM_003521	HIST1H2BM	NM_003521 // HIST1H2BM // histone cluster 1, H2bm // 6p22.1 // 8342 /// ENST00000359465	0.00354268	-0.215128
8165676	ENST0000036138:	ND4	ENST00000361381 // ND4 // NADH dehydrogenase, subunit 4 (complex I) // --- // 4538 ///	0.000568036	0.263646
8160682	NM_022917	NOL6	NM_022917 // NOL6 // nucleolar protein family 6 (RNA-associated) // 9p13.3 // 65083 ///	2.49E-06	-0.34963
8150276	NM_001102559	PPAPDC1B	NM_001102559 // PPAPDC1B // phosphatidic acid phosphatase type 2 domain containing 1B /	1.99E-05	-0.310099
7912610	NM_001024661	PRAMEF13	NM_001024661 // PRAMEF13 // PRAME family member 13 // 1p36.21 // 400736 /// NM_00109985	0.000154414	0.280757
8126784	NM_001168357	PLA2G7	NM_001168357 // PLA2G7 // phospholipase A2, group VII (platelet-activating factor acety	1.51E-05	-0.310551
8045341	---		---	0.00263571	0.22611
8098328	NM_017423	GALNT7	NM_017423 // GALNT7 // UDP-N-acetyl-alpha-D-galactosamine:polypeptide N-acetylgalactosa	5.66E-06	-0.329065
7910387	NM_021205	RHOU	NM_021205 // RHOU // ras homolog gene family, member U // 1q42.11-q42.3 // 58480 /// EN	0.00022695	-0.284462
8052762	NM_002056	GFPT1	NM_002056 // GFPT1 // glutamine--fructose-6-phosphate transaminase 1 // 2p13 // 2673 //	9.35E-09	-0.400431
7929562	NM_014803	ZNF518A	NM_014803 // ZNF518A // zinc finger protein 518A // 10q24.1 // 9849 /// ENST00000371192	0.00685402	0.208624
7928589	NM_005729	PPIF	NM_005729 // PPIF // peptidylprolyl isomerase F // 10q22-q23 // 10105 /// ENST000002251	7.01E-07	-0.365005

7983143	NM_020759	STARD9	NM_020759 // STARD9 // StAR-related lipid transfer (START) domain containing 9 // 15q15	7.11E-06	0.324978
8048120	NM_004044	ATIC	NM_004044 // ATIC // 5-aminoimidazole-4-carboxamide ribonucleotide formyltransferase/IM	0.00890064	-0.200056
8055038	NM_017980	LIMS2	NM_017980 // LIMS2 // LIM and senescent cell antigen-like domains 2 // 2q14.3 // 55679	2.08E-05	0.311079
8027002	NM_004864	GDF15	NM_004864 // GDF15 // growth differentiation factor 15 // 19p13.11 // 9518 /// ENST0000	0.00649639	-0.204614
8145570	NM_001017420	ESCO2	NM_001017420 // ESCO2 // establishment of cohesion 1 homolog 2 (S. cerevisiae) // 8p21.	0.00746108	-0.201186
8126820	NM_153840	GPR110	NM_153840 // GPR110 // G protein-coupled receptor 110 // 6p12.3 // 266977 /// NM_025048	1.32E-05	-0.318904
8115851	NM_003714	STC2	NM_003714 // STC2 // stanniocalcin 2 // 5q35.1 // 8614 /// ENST00000265087 // STC2 // s	0.000176177	-0.289435
8049044	NM_025139	ARMC9	NM_025139 // ARMC9 // armadillo repeat containing 9 // 2q37.1 // 80210 /// ENST00000349	0.00478473	-0.211438
8027304	NM_001076678	ZNF493	NM_001076678 // ZNF493 // zinc finger protein 493 // 19p12 // 284443 /// NM_175910 // Z	4.42E-05	0.302172
8105828	NM_031966	CCNB1	NM_031966 // CCNB1 // cyclin B1 // 5q12 // 891 /// ENST00000256442 // CCNB1 // cyclin B	0.00115167	-0.244609
8015366	NM_000526	KRT14	NM_000526 // KRT14 // keratin 14 // 17q12-q21 // 3861 /// ENST00000167586 // KRT14 // k	0.00139246	-0.235897
7926531	NM_178815	ARL5B	NM_178815 // ARL5B // ADP-ribosylation factor-like 5B // 10p12.31 // 221079 /// ENST000	4.17E-05	-0.308013
8155734	NM_004816	FAM189A2	NM_004816 // FAM189A2 // family with sequence similarity 189, member A2 // 9q21.11 // 9	2.59E-06	0.347493
7981752	NR_027411	GOLGA8C	NR_027411 // GOLGA8C // golgin A8 family, member C // 15q11.2 // 729786 /// NR_033353 /	0.000316994	0.265283
8149153	NR_027000	LOC349196	NR_027000 // LOC349196 // hypothetical LOC349196 // 8p23.1 // 349196 /// AK094835 // LO	0.00143427	0.241527

8149157	NR_027000	LOC349196	NR_027000 // LOC349196 // hypothetical LOC349196 // 8p23.1 // 349196 /// AK094835 // LO	0.00143427	0.241527
8127234	NM_015548	DST	NM_015548 // DST // dystonin // 6p12.1 // 667 /// NM_001723 // DST // dystonin // 6p12.	0.00079939	0.255598
8056983	NR_026966	LOC100130691	NR_026966 // LOC100130691 // hypothetical LOC100130691 // 2q31.2 // 100130691 /// AK126	0.00296554	0.227233
8053834	NM_025190	ANKRD36B	NM_025190 // ANKRD36B // ankyrin repeat domain 36B // 2q11.2 // 57730 /// NM_001164315	0.00522723	0.206531
7966621	NM_006843	SDS	NM_006843 // SDS // serine dehydratase // 12q24.13 // 10993 /// ENST00000257549 // SDS	0.000898884	-0.251207
7903358	NM_001078	VCAM1	NM_001078 // VCAM1 // vascular cell adhesion molecule 1 // 1p32-p31 // 7412 /// NM_0806	4.68E-05	-0.292672
7973067	NM_000270	PNP	NM_000270 // PNP // purine nucleoside phosphorylase // 14q13.1 // 4860 /// ENST00000361	3.51E-05	-0.313425
8152222	NM_015878	AZIN1	NM_015878 // AZIN1 // antizyme inhibitor 1 // 8q22.3 // 51582 /// NM_148174 // AZIN1 //	0.00119506	-0.243757
8125091	NM_006295	VARS	NM_006295 // VARS // valyl-tRNA synthetase // 6p21.3 // 7407 /// ENST00000211402 // VAR	0.000227416	-0.274902
8178609	NM_006295	VARS	NM_006295 // VARS // valyl-tRNA synthetase // 6p21.3 // 7407 /// ENST00000211402 // VAR	0.000227416	-0.274902
8154727	AF091236	LOC138412	AF091236 // LOC138412 // solute carrier family 25 (mitochondrial carrier; adenine nucle	0.00416195	-0.220076
7917050	---		---	0.000551147	0.261706
8105937	BT006760	GUSBP3	BT006760 // GUSBP3 // glucuronidase, beta pseudogene 3 // 5q13.2 // 653188 /// NR_02405	1.34E-07	0.38559
8002347	NM_001605	AARS	NM_001605 // AARS // alanyl-tRNA synthetase // 16q22 // 16 /// ENST00000261772 // AARS	0.000448828	-0.269175
7984662	NM_018652	GOLGA6B	NM_018652 // GOLGA6B // golgin A6 family, member B // 15q24.1 // 55889 /// NM_001038640	0.00599525	0.207484

7946323	NM_153444	OR5P2	NM_153444 // OR5P2 // olfactory receptor, family 5, subfamily P, member 2 // 11p15.4 //	0.00346288	0.217361
8032863	NM_019107	C19orf10	NM_019107 // C19orf10 // chromosome 19 open reading frame 10 // 19p13.3 // 56005 /// EN	0.000915524	-0.253328
8003953	NM_002798	PSMB6	NM_002798 // PSMB6 // proteasome (prosome, macropain) subunit, beta type, 6 // 17p13 //	0.00140883	-0.244821
8104607	---		---	0.00623371	-0.202996
8026579	NM_024074	TMEM38A	NM_024074 // TMEM38A // transmembrane protein 38A // 19p13.11 // 79041 /// ENST00000187	0.00934185	0.196022
7930921	NM_004281	BAG3	NM_004281 // BAG3 // BCL2-associated athanogene 3 // 10q25.2-q26.2 // 9531 /// ENST0000	4.62E-09	-0.422902
8030796	NR_002804	SIGLECP3	NR_002804 // SIGLECP3 // sialic acid binding Ig-like lectin, pseudogene 3 // 19q13.3 //	0.00172297	0.244762
8147990	---		---	0.000371739	0.266089
8099246	NM_025196	GRPEL1	NM_025196 // GRPEL1 // GrpE-like 1, mitochondrial (E. coli) // 4p16 // 80273 /// ENST00	2.79E-06	-0.352212
7931081	NM_021622	PLEKHA1	NM_021622 // PLEKHA1 // pleckstrin homology domain containing, family A (phosphoinositi	0.00398611	0.219825
7898002	NM_001100631	PRAMEF22	NM_001100631 // PRAMEF22 // PRAME family member 22 // 1p36.21 // 653606 /// NM_00101369	0.00352297	0.223728
8003656	NM_001165920	SERPINF2	NM_001165920 // SERPINF2 // serpin peptidase inhibitor, clade F (alpha-2 antiplasmin, p	0.00206846	-0.239087
8043687	NM_001164315	ANKRD36	NM_001164315 // ANKRD36 // ankyrin repeat domain 36 // 2q11.2 // 375248 /// AK304740 //	0.00542024	0.208592
7926983	NM_183013	CREM	NM_183013 // CREM // cAMP responsive element modulator // 10p11.21 // 1390 /// NM_18301	5.68E-09	-0.421291
7958273	---		---	0.00141539	0.237859

8149151	NR_027000	LOC349196	NR_027000 // LOC349196 // hypothetical LOC349196 // 8p23.1 // 349196 /// AK094835 // LO	0.000731225	0.254256
8149214	NR_027000	LOC349196	NR_027000 // LOC349196 // hypothetical LOC349196 // 8p23.1 // 349196 /// AK094835 // LO	0.000731225	0.254256
8054722	NM_000576	IL1B	NM_000576 // IL1B // interleukin 1, beta // 2q14 // 3553 /// ENST00000263341 // IL1B //	0.000423732	-0.268778
8046003	NM_012198	GCA	NM_012198 // GCA // grancalcin, EF-hand calcium binding protein // 2q24.2 // 25801 ///	0.00569165	-0.213321
8112564	NR_027386	GUSBP3	NR_027386 // GUSBP3 // glucuronidase, beta pseudogene 3 // 5q13.2 // 653188 /// NR_0340	2.46E-07	0.374145
7949948	NM_022338	C11orf24	NM_022338 // C11orf24 // chromosome 11 open reading frame 24 // 11q13 // 53838 /// ENST	4.26E-05	-0.298649
7965040	NM_007350	PHLDA1	NM_007350 // PHLDA1 // pleckstrin homology-like domain, family A, member 1 // 12q15 //	5.68E-08	-0.39825
7997192	NM_020995	HPR	NM_020995 // HPR // haptoglobin-related protein // 16q22.1 // 3250 /// ENST00000405951	0.00648789	-0.210277
8167006	NM_006915	RP2	NM_006915 // RP2 // retinitis pigmentosa 2 (X-linked recessive) // Xp11.3 // 6102 /// E	0.00232454	-0.230224
8016099	NM_004247	EFTUD2	NM_004247 // EFTUD2 // elongation factor Tu GTP binding domain containing 2 // 17q21.31	0.000679826	-0.254326
8133914	NR_024549	DMTF1	NR_024549 // DMTF1 // cyclin D binding myb-like transcription factor 1 // 7q21 // 9988	0.00111124	0.246513
7922040	NM_198053	CD247	NM_198053 // CD247 // CD247 molecule // 1q22-q23 // 919 /// NM_000734 // CD247 // CD247	0.0013283	0.241514
7933672	NM_001142763	PCDH15	NM_001142763 // PCDH15 // protocadherin-related 15 // 10q21.1 // 65217 /// NM_001142764	0.000182157	0.28431
7915160	NM_022157	RRAGC	NM_022157 // RRAGC // Ras-related GTP binding C // 1p34 // 64121 /// ENST00000373001 //	0.0079282	-0.196668
8132725	NM_003364	UPP1	NM_003364 // UPP1 // uridine phosphorylase 1 // 7p12.3 // 7378 /// NM_181597 // UPP1 //	2.16E-05	-0.319853

7965110	---		---	2.33E-06	0.338891
8123598	NM_030666	SERPINB1	NM_030666 // SERPINB1 // serpin peptidase inhibitor, clade B (ovalbumin), member 1 // 6	0.000307147	-0.273356
8118455	NM_007293	C4A	NM_007293 // C4A // complement component 4A (Rodgers blood group) // 6p21.3 // 720 ///	0.000215424	-0.277972
8159900	NM_152629	GLIS3	NM_152629 // GLIS3 // GLIS family zinc finger 3 // 9p24.2 // 169792 /// NM_001042413 //	0.00364804	-0.21785
8001111	NR_002837	UBE2MP1	NR_002837 // UBE2MP1 // ubiquitin-conjugating enzyme E2M pseudogene 1 // 16p11.2 // 606	0.000322426	-0.278869
8161829	BC034033	C9orf41	BC034033 // C9orf41 // chromosome 9 open reading frame 41 // 9q21.13 // 138199 /// AK29	0.00121536	-0.245724
7997642	NM_031476	CRISPLD2	NM_031476 // CRISPLD2 // cysteine-rich secretory protein LCCL domain containing 2 // 16	1.66E-06	-0.345661
8084128	NM_133462	TTC14	NM_133462 // TTC14 // tetratricopeptide repeat domain 14 // 3q26.33 // 151613 /// NM_00	0.0042406	0.219934
8118409	NM_007293	C4A	NM_007293 // C4A // complement component 4A (Rodgers blood group) // 6p21.3 // 720 ///	0.000222402	-0.27747
8179399	NM_007293	C4A	NM_007293 // C4A // complement component 4A (Rodgers blood group) // 6p21.3 // 720 ///	0.000226541	-0.277115
7963786	NM_002205	ITGA5	NM_002205 // ITGA5 // integrin, alpha 5 (fibronectin receptor, alpha polypeptide) // 12	0.00119255	-0.246932
7908924	NM_002725	PRELP	NM_002725 // PRELP // proline/arginine-rich end leucine-rich repeat protein // 1q32 //	0.00955222	0.196977
8179351	NM_001710	CFB	NM_001710 // CFB // complement factor B // 6p21.3 // 629 /// ENST00000417261 // CFB //	0.000143985	-0.285524
8064375	NM_080725	SRXN1	NM_080725 // SRXN1 // sulfiredoxin 1 // 20p13 // 140809 /// ENST00000381962 // SRXN1 //	0.00499705	-0.211148
8165684	---		---	3.15E-05	0.308619
8095680	NM_000584	IL8	NM_000584 // IL8 // interleukin 8 // 4q13-q21 // 3576 /// ENST00000307407 // IL8 // int	2.03E-07	-0.376194

8153474	NM_003313	TSTA3	NM_003313 // TSTA3 // tissue specific transplantation antigen P35B // 8q24.3 // 7264 //	0.000143789	-0.285696
7933574	NM_001077685	AGAP7	NM_001077685 // AGAP7 // ArfGAP with GTPase domain, ankyrin repeat and PH domain 7 // 1	0.0091549	0.196827
8149216	NR_027000	LOC349196	NR_027000 // LOC349196 // hypothetical LOC349196 // 8p23.1 // 349196 /// AK094835 // LO	0.000395696	0.266118
7983145	NM_020759	STARD9	NM_020759 // STARD9 // StAR-related lipid transfer (START) domain containing 9 // 15q15	2.13E-06	0.341022
8071274	L20860	GP1BB	L20860 // GP1BB // glycoprotein Ib (platelet), beta polypeptide // 22q11.21-q11.23|22q1	0.00109836	0.246594
8020702	NM_005640	TAF4B	NM_005640 // TAF4B // TAF4b RNA polymerase II, TATA box binding protein (TBP)-associate	0.00278346	-0.226
8112469	NR_027386	GUSBP3	NR_027386 // GUSBP3 // glucuronidase, beta pseudogene 3 // 5q13.2 // 653188 /// NR_0340	5.65E-08	0.391517
8061414	---		---	0.000177026	0.279927
8022393	NM_002828	PTPN2	NM_002828 // PTPN2 // protein tyrosine phosphatase, non-receptor type 2 // 18p11.3-p11.	0.000110398	-0.290649
8053337	NR_002714	REG1P	NR_002714 // REG1P // regenerating islet-derived 1 pseudogene // 2p12 // 5969 /// D5649	0.00717809	0.21033
7962895	NM_016594	FKBP11	NM_016594 // FKBP11 // FK506 binding protein 11, 19 kDa // 12q13.12 // 51303 /// NM_001	0.00388497	-0.221066
8091723	NM_206963	RARRES1	NM_206963 // RARRES1 // retinoic acid receptor responder (tazarotene induced) 1 // 3q25	3.27E-05	-0.305181
8147988	---		---	0.000387115	0.262893
7944765	---		---	5.07E-07	0.366357
7928489	---		---	0.00129311	0.240884
8124691	AY358246	HCG8	AY358246 // HCG8 // HLA complex group 8 // 6p21.3 // 100507399	0.000421081	0.269065
8177669	NR_034021	SMA5	NR_034021 // SMA5 // glucuronidase, beta pseudogene // 5q13 // 11042 /// NR_027386 // G	9.55E-08	0.38552

8175353	NM_001017438	CT45A6	NM_001017438 // CT45A6 // cancer/testis antigen family 45, member A6 // Xq26.3 // 54146	0.00868527	0.198365
8143684	NM_004911	PDIA4	NM_004911 // PDIA4 // protein disulfide isomerase family A, member 4 // 7q35 // 9601 //	5.55E-06	-0.333814
8149165	NR_027000	LOC349196	NR_027000 // LOC349196 // hypothetical LOC349196 // 8p23.1 // 349196 /// AK094835 // LO	0.000310973	0.270437
8149167	NR_027000	LOC349196	NR_027000 // LOC349196 // hypothetical LOC349196 // 8p23.1 // 349196 /// AK094835 // LO	0.000310973	0.270437
8129037	---		---	1.09E-06	0.36178
8161964	NM_174938	FRMD3	NM_174938 // FRMD3 // FERM domain containing 3 // 9q21.32 // 257019 /// ENST00000304195	3.44E-05	0.303586
7995697	NM_017839	LPCAT2	NM_017839 // LPCAT2 // lysophosphatidylcholine acyltransferase 2 // 16q12.2 // 54947 //	0.00513212	-0.211185
8149218	NR_027000	LOC349196	NR_027000 // LOC349196 // hypothetical LOC349196 // 8p23.1 // 349196 /// AK094835 // LO	0.000346951	0.267883
8149220	NR_027000	LOC349196	NR_027000 // LOC349196 // hypothetical LOC349196 // 8p23.1 // 349196 /// AK094835 // LO	0.000346951	0.267883
8149222	NR_027000	LOC349196	NR_027000 // LOC349196 // hypothetical LOC349196 // 8p23.1 // 349196 /// AK094835 // LO	0.000346951	0.267883
8149224	NR_027000	LOC349196	NR_027000 // LOC349196 // hypothetical LOC349196 // 8p23.1 // 349196 /// AK094835 // LO	0.000346951	0.267883
8149226	NR_027000	LOC349196	NR_027000 // LOC349196 // hypothetical LOC349196 // 8p23.1 // 349196 /// AK094835 // LO	0.000346951	0.267883

8013465	NR_029393	KRT16P3	NR_029393 // KRT16P3 // keratin 16 pseudogene 3 // 17p11.2 // 644945 /// NR_029392 // K	0.00405692	-0.216538
8065416	NM_001322	CST2	NM_001322 // CST2 // cystatin SA // 20p11.21 // 1470 /// ENST00000304725 // CST2 // cys	0.000840282	-0.24824
8112918	---		---	2.53E-05	0.32362
8118613	NM_006979	SLC39A7	NM_006979 // SLC39A7 // solute carrier family 39 (zinc transporter), member 7 // 6p21.3	1.23E-05	-0.32975
8178225	NM_006979	SLC39A7	NM_006979 // SLC39A7 // solute carrier family 39 (zinc transporter), member 7 // 6p21.3	1.23E-05	-0.32975
8179525	NM_006979	SLC39A7	NM_006979 // SLC39A7 // solute carrier family 39 (zinc transporter), member 7 // 6p21.3	1.23E-05	-0.32975
7986642	NR_027407	GOLGA8DP	NR_027407 // GOLGA8DP // golgin A8 family, member D (pseudogene) // 15q11.2 // 10013297	0.000327497	0.265943
8006433	NM_002982	CCL2	NM_002982 // CCL2 // chemokine (C-C motif) ligand 2 // 17q11.2-q12 // 6347 /// ENST0000	1.09E-06	-0.353593
7939559	NM_130783	TSPAN18	NM_130783 // TSPAN18 // tetraspanin 18 // 11p11.2 // 90139 /// ENST00000340160 // TSPAN	0.002783	0.229179
8114211	---		---	9.87E-06	0.324836
8089203	NM_020654	SENP7	NM_020654 // SENP7 // SUMO1/sentrin specific peptidase 7 // 3q12 // 57337 /// NM_001077	0.000497483	0.262673
8009417	NM_002266	KPNA2	NM_002266 // KPNA2 // karyopherin alpha 2 (RAG cohort 1, importin alpha 1) // 17q24.2 /	6.75E-06	-0.327364
8048864	NM_004591	CCL20	NM_004591 // CCL20 // chemokine (C-C motif) ligand 20 // 2q33-q37 // 6364 /// NM_001130	5.09E-07	-0.371842
8058373	NM_018256	WDR12	NM_018256 // WDR12 // WD repeat domain 12 // 2q33.2 // 55759 /// ENST00000261015 // WDR	0.000306555	-0.273301
7965964	NM_032148	SLC41A2	NM_032148 // SLC41A2 // solute carrier family 41, member 2 // 12q23.3 // 84102 /// ENST	7.00E-05	-0.293395
8099721	NM_015187	SEL1L3	NM_015187 // SEL1L3 // sel-1 suppressor of lin-12-like 3 (C. elegans) // 4p15.2 // 2323	0.00130471	-0.2442
8168500	NM_000291	PGK1	NM_000291 // PGK1 // phosphoglycerate kinase 1 // Xq13 // 5230 /// ENST00000373316 // P	0.00633437	-0.207277

8099524	NM_001130834	LDB2	NM_001130834 // LDB2 // LIM domain binding 2 // 4p16 // 9079 /// NM_001290 // LDB2 // L	9.26E-05	0.292911
8093104	NM_138461	TM4SF19	NM_138461 // TM4SF19 // transmembrane 4 L six family member 19 // 3q29 // 116211 /// EN	0.000295559	-0.268944
7903719	NM_004037	AMPD2	NM_004037 // AMPD2 // adenosine monophosphate deaminase 2 // 1p13.3 // 271 /// NM_13915	0.00713292	-0.20885
7973867	---		---	4.67E-05	0.302766
7982154	NR_002824	HERC2P2	NR_002824 // HERC2P2 // hect domain and RLD 2 pseudogene 2 // 15q11.2 // 400322 /// NM_	0.00448811	0.214863
7990054	NM_018003	UACA	NM_018003 // UACA // uveal autoantigen with coiled-coil domains and ankyrin repeats //	0.000434674	0.268081
7991695	NR_027407	GOLGA8DP	NR_027407 // GOLGA8DP // golgin A8 family, member D (pseudogene) // 15q11.2 // 10013297	0.000384882	0.261769
8081343	NM_017819	RG9MTD1	NM_017819 // RG9MTD1 // RNA (guanine-9-) methyltransferase domain containing 1 // 3q12.	0.00795736	-0.205138
8116980	NM_001165032	RNF182	NM_001165032 // RNF182 // ring finger protein 182 // 6p23 // 221687 /// NM_152737 // RN	0.000138613	0.28367
8165694	---		---	3.63E-05	0.308977
7911335	---		---	3.63E-05	0.308977
8092457	NM_005787	ALG3	NM_005787 // ALG3 // asparagine-linked glycosylation 3, alpha-1,3- mannosyltransferase	0.000533989	-0.264111
8165680	---		---	1.08E-05	0.32494
8071049	AF172850	LOC51152	AF172850 // LOC51152 // melanoma antigen // --- // 51152	9.42E-06	0.323807
8117128	NM_001949	E2F3	NM_001949 // E2F3 // E2F transcription factor 3 // 6p22 // 1871 /// ENST00000346618 //	9.93E-05	-0.291106
8063382	NM_005985	SNAI1	NM_005985 // SNAI1 // snail homolog 1 (Drosophila) // 20q13.2 // 6615 /// ENST000002440	9.85E-05	-0.294494
8047006	NM_144708	ANKAR	NM_144708 // ANKAR // ankyrin and armadillo repeat containing // 2q32.2 // 150709 /// E	0.000385704	0.267839
8155214	NM_014791	MELK	NM_014791 // MELK // maternal embryonic leucine zipper kinase // 9p13.2 // 9833 /// ENS	0.00930763	-0.196895

7923426	NM_014176	UBE2T	NM_014176 // UBE2T // ubiquitin-conjugating enzyme E2T (putative) // 1q32.1 // 29089 //	3.45E-05	-0.308152
8031013	NM_001012728	DPRX	NM_001012728 // DPRX // divergent-paired related homeobox // 19q13.42 // 503834 /// ENS	0.00340328	0.219751
7986947	NR_024074	GOLGA8IP	NR_024074 // GOLGA8IP // golgin A8 family, member I (pseudogene) // 15q11.2 // 283796 /	3.09E-05	0.302139
8150186	NM_024787	RNF122	NM_024787 // RNF122 // ring finger protein 122 // 8p12 // 79845 /// ENST00000256257 //	0.00245687	-0.233694
7915084	NM_024640	YRDC	NM_024640 // YRDC // yrdC domain containing (E. coli) // 1p34.3 // 79693 /// ENST000003	2.23E-05	-0.324707
7929990	NM_015062	PPRC1	NM_015062 // PPRC1 // peroxisome proliferator-activated receptor gamma, coactivator-rel	3.61E-05	-0.310688
7963946	NM_002429	MMP19	NM_002429 // MMP19 // matrix metallopeptidase 19 // 12q14 // 4327 /// ENST00000322569 /	2.19E-05	-0.319237
7974851	NM_001530	HIF1A	NM_001530 // HIF1A // hypoxia inducible factor 1, alpha subunit (basic helix-loop-helix	3.34E-11	-0.455381
8083136	NM_001679	ATP1B3	NM_001679 // ATP1B3 // ATPase, Na+/K+ transporting, beta 3 polypeptide // 3q23 // 483 /	8.68E-05	-0.302349
8008969	NM_005994	TBX2	NM_005994 // TBX2 // T-box 2 // 17q23 // 6909 /// BC052566 // TBX2 // T-box 2 // 17q23	0.00140202	0.242825
8119661	NM_033112	RRP36	NM_033112 // RRP36 // ribosomal RNA processing 36 homolog (S. cerevisiae) // 6p21.1 //	0.00281961	-0.231315
8007397	NM_176863	PSME3	NM_176863 // PSME3 // proteasome (prosome, macropain) activator subunit 3 (PA28 gamma;	6.92E-05	-0.30382
8025488	NM_003451	ZNF177	NM_003451 // ZNF177 // zinc finger protein 177 // 19p13.2 // 7730 /// NM_001172650 // Z	6.47E-06	0.331577
8165658	ENST0000036145:	ND2	ENST00000361453 // ND2 // MTND2 // --- // 4536	4.39E-05	0.305847
8176193	NM_000132	F8	NM_000132 // F8 // coagulation factor VIII, procoagulant component // Xq28 // 2157 ///	0.00609598	0.206207

8032157	NM_014963	SBNO2	NM_014963 // SBNO2 // strawberry notch homolog 2 (Drosophila) // 19p13.3 // 22904 /// N	6.07E-07	-0.373771
8112731	NM_004101	F2RL2	NM_004101 // F2RL2 // coagulation factor II (thrombin) receptor-like 2 // 5q13 // 2151	0.00249796	-0.224754
8124469	NR_034021	SMA5	NR_034021 // SMA5 // glucuronidase, beta pseudogene // 5q13 // 11042 /// NR_027386 // G	3.78E-09	0.425791
8031358	NM_012314	KIR2DS4	NM_012314 // KIR2DS4 // killer cell immunoglobulin-like receptor, two domains, short cy	0.000339061	0.268135
8136863	NM_153345	TMEM139	NM_153345 // TMEM139 // transmembrane protein 139 // 7q34 // 135932 /// ENST00000359333	0.000134774	0.281367
7971661	NR_029485	MIR15A	NR_029485 // MIR15A // microRNA 15a // 13q14.2 // 406948	0.000266037	0.272701
7984540	NM_138555	KIF23	NM_138555 // KIF23 // kinesin family member 23 // 15q23 // 9493 /// NM_004856 // KIF23	0.00221227	-0.233161
7970381	NR_003366	ANKRD20B	NR_003366 // ANKRD20B // ankyrin repeat domain 20B // 2q11.1 // 729171 /// ENST00000417	0.00014957	0.285578
8133038	---		---	8.68E-05	0.289375
7917322	NM_032184	SYDE2	NM_032184 // SYDE2 // synapse defective 1, Rho GTPase, homolog 2 (C. elegans) // 1p22.3	0.000190785	0.275564
8093330	---		---	0.00103967	0.249871
8051998	NM_139279	MCFD2	NM_139279 // MCFD2 // multiple coagulation factor deficiency 2 // 2p21 // 90411 /// NM_	0.0054575	-0.216258
8083941	NM_018098	ECT2	NM_018098 // ECT2 // epithelial cell transforming sequence 2 oncogene // 3q26.1-q26.2 /	0.000248517	-0.272385
7974404	NM_005192	CDKN3	NM_005192 // CDKN3 // cyclin-dependent kinase inhibitor 3 // 14q22 // 1033 /// NM_00113	0.00151031	-0.237872

7976726	NM_016337	EVL	NM_016337 // EVL // Enah/Vasp-like // 14q32.2 // 51466 /// ENST00000392920 // EVL // En	0.00841802	0.191607
8115600	NM_022090	C5orf54	NM_022090 // C5orf54 // chromosome 5 open reading frame 54 // 5q33.3 // 63920 /// ENST0	0.00646751	0.207949
8027862	NM_005306	FFAR2	NM_005306 // FFAR2 // free fatty acid receptor 2 // 19q13.1 // 2867 /// ENST00000246549	0.00306087	-0.228182
7904702	NM_203458	NOTCH2NL	NM_203458 // NOTCH2NL // notch 2 N-terminal like // 1q21.2 // 388677 /// ENST0000036207	0.0047144	0.215174
8110755	NM_006598	SLC12A7	NM_006598 // SLC12A7 // solute carrier family 12 (potassium/chloride transporters), mem	1.43E-05	-0.324721
8089295	---		---	0.00108846	0.251355
8009502	NM_000891	KCNJ2	NM_000891 // KCNJ2 // potassium inwardly-rectifying channel, subfamily J, member 2 // 1	0.000143879	-0.285909
7913566	NM_000864	HTR1D	NM_000864 // HTR1D // 5-hydroxytryptamine (serotonin) receptor 1D // 1p36.3-p34.3 // 33	0.00357801	0.221773
7962829	NR_002951	SNORA2B	NR_002951 // SNORA2B // small nucleolar RNA, H/ACA box 2B // 12q13.11 // 677794	0.00215101	0.235967
7970392	---		---	0.00010656	0.297969
8111677	NM_002310	LIFR	NM_002310 // LIFR // leukemia inhibitory factor receptor alpha // 5p13-p12 // 3977 ///	0.000194005	0.281906
8092765	NM_178496	C3orf59	NM_178496 // C3orf59 // chromosome 3 open reading frame 59 // 3q29 // 151963 /// ENST00	0.00783747	-0.204812
7923965	---		---	0.0102322	0.194588
8021245	NM_005215	DCC	NM_005215 // DCC // deleted in colorectal carcinoma // 18q21.3 // 1630 /// ENST00000442	0.00431619	0.217419
8161943	BC047037	LOC644714	BC047037 // LOC644714 // hypothetical LOC644714 // 3p21.31 // 644714	0.00996148	0.196364
8135661	NM_000492	CFTR	NM_000492 // CFTR // cystic fibrosis transmembrane conductance regulator (ATP-binding c	0.000574045	-0.264719
7897803	NM_000302	PLOD1	NM_000302 // PLOD1 // procollagen-lysine 1, 2-oxoglutarate 5-dioxygenase 1 // 1p36.22 /	0.00157872	-0.240852
8167185	NM_003254	TIMP1	NM_003254 // TIMP1 // TIMP metallopeptidase inhibitor 1 // Xp11.3-p11.23 // 7076 /// EN	5.75E-06	-0.333302

8133176	NM_014504	RABGEF1	NM_014504 // RABGEF1 // RAB guanine nucleotide exchange factor (GEF) 1 // 7q11.21 // 27	0.000224728	-0.283485
8091648	NM_007107	SSR3	NM_007107 // SSR3 // signal sequence receptor, gamma (translocon-associated protein gam	0.00370038	-0.219892
8073816	NR_027033	LOC400931	NR_027033 // LOC400931 // hypothetical LOC400931 // 22q13.31 // 400931 /// ENST00000435	0.00175294	0.233022
7985317	NM_018689	KIAA1199	NM_018689 // KIAA1199 // KIAA1199 // 15q24 // 57214 /// ENST00000394685 // KIAA1199 //	0.00885355	-0.196739
8048847	NM_001135187	AGFG1	NM_001135187 // AGFG1 // ArfGAP with FG repeats 1 // 2q36.3 // 3267 /// NM_004504 // AG	0.00105687	-0.251227
7919386	---		---	0.00682123	0.19965
7899560	---		---	0.00599468	0.211962
7924682	NM_003240	LEFTY2	NM_003240 // LEFTY2 // left-right determination factor 2 // 1q42.1 // 7044 /// NM_00117	0.00346253	0.218519
8021208	NM_002396	ME2	NM_002396 // ME2 // malic enzyme 2, NAD(+)-dependent, mitochondrial // 6p25-p24|18q21 /	0.00521563	-0.207459
7933405	NM_133446	AGAP4	NM_133446 // AGAP4 // ArfGAP with GTPase domain, ankyrin repeat and PH domain 4 // 10q1	0.00894863	0.198357
8031374	NM_002000	FCAR	NM_002000 // FCAR // Fc fragment of IgA, receptor for // 19q13.2-q13.4 // 2204 /// NM_1	3.76E-05	-0.311399
8132092	NM_006774	INMT	NM_006774 // INMT // indolethylamine N-methyltransferase // 7p15.1 // 11185 /// ENST000	0.00891171	0.199762
8005549	NM_001129778	GRAPL	NM_001129778 // GRAPL // GRB2-related adaptor protein-like // 17p11.2 // 400581 /// ENS	0.00266174	0.228835
7986701	NR_002824	HERC2P2	NR_002824 // HERC2P2 // hect domain and RLD 2 pseudogene 2 // 15q11.2 // 400322 /// NR_	0.00486877	0.212799

8126750	NM_021572	ENPP5	NM_021572 // ENPP5 // ectonucleotide pyrophosphatase/phosphodiesterase 5 (putative) //	0.00899095	-0.197819
8023043	NM_024430	PSTPIP2	NM_024430 // PSTPIP2 // proline-serine-threonine phosphatase interacting protein 2 // 1	0.000253736	-0.273212
7929388	NM_016341	PLCE1	NM_016341 // PLCE1 // phospholipase C, epsilon 1 // 10q23 // 51196 /// NM_001165979 //	0.00243715	0.230905
7970864	NM_006644	HSPH1	NM_006644 // HSPH1 // heat shock 105kDa/110kDa protein 1 // 13q12.3 // 10808 /// ENST00	0.000530405	-0.2583
7982206	NR_024074	GOLGA8IP	NR_024074 // GOLGA8IP // golgin A8 family, member I (pseudogene) // 15q11.2 // 283796 /	3.68E-05	0.305886
8108981	NM_001112724	STK32A	NM_001112724 // STK32A // serine/threonine kinase 32A // 5q32 // 202374 /// NM_145001 /	0.00101769	-0.248755
8103226	NM_152680	TMEM154	NM_152680 // TMEM154 // transmembrane protein 154 // 4q31.3 // 201799 /// ENST000003043	0.00145599	-0.235199
8004497	NM_001416	EIF4A1	NM_001416 // EIF4A1 // eukaryotic translation initiation factor 4A1 // 17p13 // 1973 //	1.78E-05	-0.329288
8012475	NM_005964	MYH10	NM_005964 // MYH10 // myosin, heavy chain 10, non-muscle // 17p13 // 4628 /// ENST00000	0.00176906	0.236777
8027292	NM_133473	ZNF431	NM_133473 // ZNF431 // zinc finger protein 431 // 19p12 // 170959 /// ENST00000311048 /	0.00533085	0.214489
8015323	NM_153490	KRT13	NM_153490 // KRT13 // keratin 13 // 17q12-q21.2 // 3860 /// NM_002274 // KRT13 // kerat	0.00379582	-0.221967
8109383	NM_000827	GRIA1	NM_000827 // GRIA1 // glutamate receptor, ionotropic, AMPA 1 // 5q33|5q31.1 // 2890 ///	5.46E-07	0.368077
7905329	NM_006818	MLLT11	NM_006818 // MLLT11 // myeloid/lymphoid or mixed-lineage leukemia (trithorax homolog, D	0.000502315	-0.252665
7933427	NM_001144000	AGAP5	NM_001144000 // AGAP5 // ArfGAP with GTPase domain, ankyrin repeat and PH domain 5 // 1	0.00624273	0.207296
8045533	---		---	0.000462862	0.264039

8029580	NM_006509	RELB	NM_006509 // RELB // v-rel reticuloendotheliosis viral oncogene homolog B // 19q13.32 /	0.000139975	-0.291537
8112202	NM_006622	PLK2	NM_006622 // PLK2 // polo-like kinase 2 // 5q12.1-q13.2 // 10769 /// ENST00000274289 //	0.00462731	0.21945
7905058	NR_027002	LOC388692	NR_027002 // LOC388692 // hypothetical LOC388692 // 1q21.2 // 388692 /// ENST0000036917	0.000339593	0.271614
7954173	NM_007178	STRAP	NM_007178 // STRAP // serine/threonine kinase receptor associated protein // 12p12.3 //	0.00194929	-0.239962
7948881	NM_018093	WDR74	NM_018093 // WDR74 // WD repeat domain 74 // 11q12.3 // 54663 /// ENST00000278856 // WD	0.000624192	-0.264992
7934451	NM_001144000	AGAP5	NM_001144000 // AGAP5 // ArfGAP with GTPase domain, ankyrin repeat and PH domain 5 // 1	0.00446477	0.215562
8045171	NM_033416	IMP4	NM_033416 // IMP4 // IMP4, U3 small nucleolar ribonucleoprotein, homolog (yeast) // 2q2	0.000844928	-0.257548
8165648	AK290098	C7orf11	AK290098 // C7orf11 // chromosome 7 open reading frame 11 // 7p14.1 // 136647	4.11E-05	0.314762
7960757	NM_016546	C1RL	NM_016546 // C1RL // complement component 1, r subcomponent-like // 12p13.31 // 51279 /	0.000262803	-0.277985
8029914	NM_018485	GPR77	NM_018485 // GPR77 // G protein-coupled receptor 77 // 19q13.33 // 27202 /// ENST000002	0.00669309	-0.210081
7903777	NM_000851	GSTM5	NM_000851 // GSTM5 // glutathione S-transferase mu 5 // 1p13.3 // 2949 /// ENST00000256	0.00111685	0.250702
8105040	NM_003999	OSMR	NM_003999 // OSMR // oncostatin M receptor // 5p13.1 // 9180 /// NM_001168355 // OSMR /	1.43E-07	-0.378015
8107204	---		---	5.90E-06	0.332982
7906307	NM_018240	KIRREL	NM_018240 // KIRREL // kin of IRRE like (Drosophila) // 1q21-q25 // 55243 /// ENST00000	0.00178765	-0.237212
8094301	NM_004787	SLIT2	NM_004787 // SLIT2 // slit homolog 2 (Drosophila) // 4p15.2 // 9353 /// ENST00000504154	3.95E-05	0.307166

8131573	NM_212460	ARL4A	NM_212460 // ARL4A // ADP-ribosylation factor-like 4A // 7p21.3 // 10124 /// NM_0010371	0.000780308	0.251458
8148317	NM_002467	MYC	NM_002467 // MYC // v-myc myelocytomatosis viral oncogene homolog (avian) // 8q24.21 //	2.00E-06	-0.351726
8040753	NM_017727	TMEM214	NM_017727 // TMEM214 // transmembrane protein 214 // 2p23.3 // 54867 /// NM_001083590 /	0.00216899	-0.230923
8063000	NM_006103	WFDC2	NM_006103 // WFDC2 // WAP four-disulfide core domain 2 // 20q12-q13.2 // 10406 /// ENST	0.00465541	-0.216155
7927732	NM_032199	ARID5B	NM_032199 // ARID5B // AT rich interactive domain 5B (MRF1-like) // 10q21.2 // 84159 //	0.000323705	-0.258663
8019541	NM_024702	ZNF750	NM_024702 // ZNF750 // zinc finger protein 750 // 17q25.3 // 79755 /// ENST00000269394	0.000512081	-0.269747
8109159	NR_029686	MIR145	NR_029686 // MIR145 // microRNA 145 // 5q32 // 406937 /// NR_027180 // LOC728264 // hyp	0.000346984	0.269486
8101284	NM_006259	PRKG2	NM_006259 // PRKG2 // protein kinase, cGMP-dependent, type II // 4q13.1-q21.1 // 5593 /	5.34E-06	0.334304
8111127	NM_001102562	11-Mar	NM_001102562 // MARCH11 // membrane-associated ring finger (C3HC4) 11 // 5p15.1 // 4410	0.00242485	-0.230882
8090737	NM_153240	NPHP3	NM_153240 // NPHP3 // nephronophthisis 3 (adolescent) // 3q22.1 // 27031 /// ENST000003	0.00331283	0.224155
8083978	NM_207015	NAALADL2	NM_207015 // NAALADL2 // N-acetylated alpha-linked acidic dipeptidase-like 2 // 3q26.31	5.16E-05	-0.295942
7971191	NR_003365	SUGT1P3	NR_003365 // SUGT1P3 // suppressor of G2 allele of SKP1 (S. cerevisiae) pseudogene 3 //	0.000605603	0.263091
7930917	AK097099	GRK5	AK097099 // GRK5 // G protein-coupled receptor kinase 5 // 10q26.11 // 2869	0.00170038	0.236514
7908610	---		---	0.00107712	0.250035
8036045	NM_175872	ZNF792	NM_175872 // ZNF792 // zinc finger protein 792 // 19q13.11 // 126375 /// ENST0000040480	0.00560346	0.211679
7976567	NM_000710	BDKRB1	NM_000710 // BDKRB1 // bradykinin receptor B1 // 14q32.1-q32.2 // 623 /// ENST000002166	0.000334511	-0.270704
7916246	---		---	0.00175943	0.235271

7960124	NM_138575	PGAM5	NM_138575 // PGAM5 // phosphoglycerate mutase family member 5 // 12q24.33 // 192111 ///	3.75E-05	-0.312466
8107113	---		---	0.000240714	0.272495
7909390	NM_175710	CR1L	NM_175710 // CR1L // complement component (3b/4b) receptor 1-like // 1q32.1 // 1379 ///	0.00524891	-0.215013
8167656	NM_001005333	MAGED1	NM_001005333 // MAGED1 // melanoma antigen family D, 1 // Xp11.23 // 9500 /// NM_006986	0.00967675	-0.198376
7933331	NM_001190810	AGAP9	NM_001190810 // AGAP9 // ArfGAP with GTPase domain, ankyrin repeat and PH domain 9 // 1	0.00548138	0.21061
7939642	NM_052854	CREB3L1	NM_052854 // CREB3L1 // cAMP responsive element binding protein 3-like 1 // 11p11.2 //	0.00039881	-0.264178
8082816	NM_021203	SRPRB	NM_021203 // SRPRB // signal recognition particle receptor, B subunit // 3q22.1 // 5847	2.98E-06	-0.345486
8097449	NM_032961	PCDH10	NM_032961 // PCDH10 // protocadherin 10 // 4q28.3 // 57575 /// NM_020815 // PCDH10 // p	3.06E-05	0.312745
8113073	NM_020801	ARRDC3	NM_020801 // ARRDC3 // arrestin domain containing 3 // 5q14.3 // 57561 /// ENST00000265	0.000132124	-0.290017
8107232	---		---	0.000886851	0.254102
8173551	NM_002637	PHKA1	NM_002637 // PHKA1 // phosphorylase kinase, alpha 1 (muscle) // Xq12-q13 // 5255 /// NM	0.0095459	-0.198299
7927353	NM_001190810	AGAP9	NM_001190810 // AGAP9 // ArfGAP with GTPase domain, ankyrin repeat and PH domain 9 // 1	0.00361454	0.220483
7906954	NM_002585	PBX1	NM_002585 // PBX1 // pre-B-cell leukemia homeobox 1 // 1q23 // 5087 /// ENST00000420696	0.00565915	0.211785
8162529	---		---	3.40E-05	0.302869
7979241	NM_001202	BMP4	NM_001202 // BMP4 // bone morphogenetic protein 4 // 14q22-q23 // 652 /// NM_130851 //	0.00626377	0.211449
8140424	NM_175064	SPDYE1	NM_175064 // SPDYE1 // speedy homolog E1 (Xenopus laevis) // 7p13 // 285955 /// NR_0036	6.60E-06	0.332064
8042513	---		---	0.000101914	0.29199

8166607	---		---	0.00612684	0.212742
8146645	NM_152414	BHLHE22	NM_152414 // BHLHE22 // basic helix-loop-helix family, member e22 // 8q13 // 27319 ///	0.00957902	-0.191331
8055639	---		---	0.00792605	0.204756
7928695	NM_032333	C10orf58	NM_032333 // C10orf58 // chromosome 10 open reading frame 58 // 10q23.1 // 84293 /// NR	0.00312649	-0.224147
8153021	NM_003033	ST3GAL1	NM_003033 // ST3GAL1 // ST3 beta-galactoside alpha-2,3-sialyltransferase 1 // 8q24.22 /	3.58E-06	-0.350587
8144416	NR_027000	LOC349196	NR_027000 // LOC349196 // hypothetical LOC349196 // 8p23.1 // 349196 /// AK094835 // LO	0.00050015	0.262617
8144418	NR_027000	LOC349196	NR_027000 // LOC349196 // hypothetical LOC349196 // 8p23.1 // 349196 /// AK094835 // LO	0.00050015	0.262617
8144490	NR_027000	LOC349196	NR_027000 // LOC349196 // hypothetical LOC349196 // 8p23.1 // 349196 /// AK094835 // LO	0.00050015	0.262617
8144492	NR_027000	LOC349196	NR_027000 // LOC349196 // hypothetical LOC349196 // 8p23.1 // 349196 /// AK094835 // LO	0.00050015	0.262617
8021584	NM_002639	SERPINB5	NM_002639 // SERPINB5 // serpin peptidase inhibitor, clade B (ovalbumin), member 5 // 1	0.00271723	-0.225585
8009727	NM_001545	ICT1	NM_001545 // ICT1 // immature colon carcinoma transcript 1 // 17q25.1 // 3396 /// ENST0	0.00511947	-0.219388
7928999	NM_001102469	LIPN	NM_001102469 // LIPN // lipase, family member N // 10q23.31 // 643418 /// ENST000004044	0.00997376	-0.200291
8013521	---		---	6.92E-05	0.297705
8084880	NM_005524	HES1	NM_005524 // HES1 // hairy and enhancer of split 1, (Drosophila) // 3q28-q29 // 3280 //	0.000446998	-0.26109
8111214	XM_001715032	LOC391766	XM_001715032 // LOC391766 // putative TAF11-like protein ENSP00000332601-like // 5p15.1	0.0013972	0.240065

7912198	NM_001428	ENO1	NM_001428 // ENO1 // enolase 1, (alpha) // 1p36.2 // 2023 /// ENST00000234590 // ENO1 /	0.000163345	-0.286051
7928870	---		---	0.000439025	0.26672
8008982	NM_018488	TBX4	NM_018488 // TBX4 // T-box 4 // 17q21-q22 // 9496 /// ENST00000240335 // TBX4 // T-box	0.00140154	0.242859
8158240	NM_021109	TMSB4X	NM_021109 // TMSB4X // thymosin beta 4, X-linked // Xq21.3-q22 // 7114 /// NM_183049 //	0.00109736	0.245728
8122176	NM_003206	TCF21	NM_003206 // TCF21 // transcription factor 21 // 6pter-qter // 6943 /// NM_198392 // TC	0.00354877	0.223648
8113120	BC028919	TOB2	BC028919 // TOB2 // transducer of ERBB2, 2 // 22q13.2 // 10766	1.19E-06	0.356121
8165707	BC028919	TOB2	BC028919 // TOB2 // transducer of ERBB2, 2 // 22q13.2 // 10766	1.19E-06	0.356121
8117458	NM_001145009	BTN3A1	NM_001145009 // BTN3A1 // butyrophilin, subfamily 3, member A1 // 6p22.1 // 11119 /// N	0.000481152	0.263855
8146794	NM_024870	PREX2	NM_024870 // PREX2 // phosphatidylinositol-3,4,5-trisphosphate-dependent Rac exchange f	0.000950743	0.249089
7976556	NR_023938	C14orf132	NR_023938 // C14orf132 // chromosome 14 open reading frame 132 // 14q32.2 // 56967	0.00894745	0.20108
8102877	NM_004362	CLGN	NM_004362 // CLGN // calmegin // 4q28.3-q31.1 // 1047 /// NM_001130675 // CLGN // calme	0.00385878	-0.218903
7916219	NM_023077	C1orf163	NM_023077 // C1orf163 // chromosome 1 open reading frame 163 // 1p32.3 // 65260 /// ENS	8.12E-05	-0.299787
8096580	NM_000253	MTTP	NM_000253 // MTTP // microsomal triglyceride transfer protein // 4q24 // 4547 /// ENST0	0.000606421	-0.255778
8069511	NR_027270	C21orf81	NR_027270 // C21orf81 // ankyrin repeat domain 20 family, member A3 pseudogene // 21q11	0.000168398	0.280915
7928318	NM_138357	CCDC109A	NM_138357 // CCDC109A // coiled-coil domain containing 109A // 10q22.1 // 90550 /// ENS	0.000684113	-0.253688
7934898	NM_144590	ANKRD22	NM_144590 // ANKRD22 // ankyrin repeat domain 22 // 10q23.31 // 118932 /// ENST00000371	0.00585511	-0.20836

8008064	NM_018129	PNPO	NM_018129 // PNPO // pyridoxamine 5'-phosphate oxidase // 17q21.32 // 55163 /// ENST000	0.0013038	-0.239619
7922162	NM_006996	SLC19A2	NM_006996 // SLC19A2 // solute carrier family 19 (thiamine transporter), member 2 // 1q	1.41E-05	-0.330993
8032789	NM_017720	STAP2	NM_017720 // STAP2 // signal transducing adaptor family member 2 // 19p13.3 // 55620 //	0.00156561	-0.245207
7947423	---		---	0.00145146	0.246338
8060745	NM_175839	SMOX	NM_175839 // SMOX // spermine oxidase // 20p13 // 54498 /// NM_175842 // SMOX // spermi	0.00655667	-0.208989
8165700	---		---	1.92E-06	0.35035
8013354	---		---	0.000704524	0.257607
8174076	NM_000169	GLA	NM_000169 // GLA // galactosidase, alpha // Xq22 // 2717 /// ENST00000218516 // GLA //	0.000160496	-0.272637
8147756	NM_024812	BAALC	NM_024812 // BAALC // brain and acute leukemia, cytoplasmic // 8q22.3 // 79870 /// NM_0	0.00865184	0.196115
8128371	NM_032511	C6orf168	NM_032511 // C6orf168 // chromosome 6 open reading frame 168 // 6q16.2 // 84553 /// ENS	0.00563022	-0.211393
8173493	NM_001142797	CXCR3	NM_001142797 // CXCR3 // chemokine (C-X-C motif) receptor 3 // Xq13 // 2833 /// NM_0015	0.00703124	0.208008
8117589	NM_003536	HIST1H3H	NM_003536 // HIST1H3H // histone cluster 1, H3h // 6p22.1 // 8357 /// BC096128 // HIST1	0.00296006	-0.226978
7941563	---		---	0.00459668	0.212002
8092621	NM_017541	CRYGS	NM_017541 // CRYGS // crystallin, gamma S // 3q25-qter // 1427 /// NM_001134415 // TBCC	5.85E-05	0.300302
8099107	NM_017816	LYAR	NM_017816 // LYAR // Ly1 antibody reactive homolog (mouse) // 4p16.3 // 55646 /// NM_00	0.0060562	-0.212409
7923956	---		---	0.00167655	0.242063
8149720	NM_004430	EGR3	NM_004430 // EGR3 // early growth response 3 // 8p23-p21 // 1960 /// ENST00000317216 //	0.00227395	-0.231388
7934810	---		---	0.00225614	0.232921

7972805	NM_017817	RAB20	NM_017817 // RAB20 // RAB20, member RAS oncogene family // 13q34 // 55647 /// ENST00000	0.000451066	-0.267126
7930226	NM_014976	PDCD11	NM_014976 // PDCD11 // programmed cell death 11 // 10q24.33 // 22984 /// ENST0000036979	0.00906146	-0.201903
7977879	NM_001144932	PSMB5	NM_001144932 // PSMB5 // proteasome (prosome, macropain) subunit, beta type, 5 // 14q11	0.00966875	-0.199595
7920238	NM_005621	S100A12	NM_005621 // S100A12 // S100 calcium binding protein A12 // 1q21 // 6283 /// ENST000003	1.41E-05	-0.331154
7926105	NM_001002295	GATA3	NM_001002295 // GATA3 // GATA binding protein 3 // 10p15 // 2625 /// NM_002051 // GATA3	0.000329123	0.269266
8114898	---		---	0.000132198	0.290361
7974366	NM_000956	PTGER2	NM_000956 // PTGER2 // prostaglandin E receptor 2 (subtype EP2), 53kDa // 14q22 // 5732	0.000164051	-0.286593
8019737	NM_002266	KPNA2	NM_002266 // KPNA2 // karyopherin alpha 2 (RAG cohort 1, importin alpha 1) // 17q24.2 /	4.16E-06	-0.333206
8018082	NM_001159770	SLC39A11	NM_001159770 // SLC39A11 // solute carrier family 39 (metal ion transporter), member 11	0.0010768	-0.247409
7967025	---		---	1.49E-05	0.323579
7982271	NR_024074	GOLGA8IP	NR_024074 // GOLGA8IP // golgin A8 family, member I (pseudogene) // 15q11.2 // 283796 /	0.000157583	0.275399
8100603	NM_001812	CENPC1	NM_001812 // CENPC1 // centromere protein C 1 // 4q13.2 // 1060 /// ENST00000273853 //	5.21E-05	0.310928
8135033	NM_001164462	MUC12	NM_001164462 // MUC12 // mucin 12, cell surface associated // 7q22 // 10071 /// ENST000	0.00895261	0.198718
8149228	NR_027000	LOC349196	NR_027000 // LOC349196 // hypothetical LOC349196 // 8p23.1 // 349196 /// AK094835 // LO	0.000143115	0.285539

7927649	NM_018464	CISD1	NM_018464 // CISD1 // CDGSH iron sulfur domain 1 // 10q21.1 // 55847 /// ENST0000033392	0.000872386	-0.250663
8012349	NM_002616	PER1	NM_002616 // PER1 // period homolog 1 (Drosophila) // 17p13.1 // 5187 /// ENST000003172	0.00491192	-0.220722
7991668	NR_033351	GOLGA8F	NR_033351 // GOLGA8F // golgin A8 family, member F // 15q13.1 // 100132565 /// NR_03335	0.000105389	0.285291
7900235	NM_012090	MACF1	NM_012090 // MACF1 // microtubule-actin crosslinking factor 1 // 1p32-p31 // 23499 ///	2.37E-05	0.313831
7946567	---		---	0.00158479	0.245611
7981787	NM_001001413	GOLGA6L1	NM_001001413 // GOLGA6L1 // golgin A6 family-like 1 // 15q11.2 // 283767 /// ENST000003	0.000560805	0.260457
8025601	NM_000201	ICAM1	NM_000201 // ICAM1 // intercellular adhesion molecule 1 // 19p13.3-p13.2 // 3383 /// EN	0.000912524	-0.253819
8059111	NM_005689	ABCB6	NM_005689 // ABCB6 // ATP-binding cassette, sub-family B (MDR/TAP), member 6 // 2q36 //	0.000669977	-0.251429
7983393	NM_003104	SORD	NM_003104 // SORD // sorbitol dehydrogenase // 15q15.3 // 6652 /// NR_034039 // SORD //	0.0086925	-0.201259
8175683	NR_029638	MIR224	NR_029638 // MIR224 // microRNA 224 // Xq28 // 407009 /// U92285 // GABRE // gamma-amin	0.000790808	0.251384
7982131	NR_033353	GOLGA8G	NR_033353 // GOLGA8G // golgin A8 family, member G // 15q13.1 // 283768 /// NR_033351 /	0.000119304	0.281734
7986922	NR_033353	GOLGA8G	NR_033353 // GOLGA8G // golgin A8 family, member G // 15q13.1 // 283768 /// NR_033351 /	0.00011931	0.281733
7992867	NR_033904	FLJ39639	NR_033904 // FLJ39639 // hypothetical protein FLJ39639 // 16p13.3 // 283876	0.00380535	0.216904
8047159	---		---	0.00350893	0.22446
8046124	NM_005771	DHRS9	NM_005771 // DHRS9 // dehydrogenase/reductase (SDR family) member 9 // 2q31.1 // 10170	0.00196166	-0.233412
7951140	AY358248	LOC100131541	AY358248 // LOC100131541 // hypothetical LOC100131541 // 11q21 // 100131541	0.00257776	0.217417

8101322	AB014771	MOP-1	AB014771 // MOP-1 // MOP-1 // 4q21.22 // 643616	0.000833413	0.252472
8129637	NM_004665	VNN2	NM_004665 // VNN2 // vanin 2 // 6q23-q24 // 8875 /// NM_078488 // VNN2 // vanin 2 // 6q	0.000411162	-0.272896
7921821	NM_005099	ADAMTS4	NM_005099 // ADAMTS4 // ADAM metallopeptidase with thrombospondin type 1 motif, 4 // 1q	7.93E-08	-0.391683
7966052	NM_004075	CRY1	NM_004075 // CRY1 // cryptochrome 1 (photolyase-like) // 12q23-q24.1 // 1407 /// ENST00	3.48E-05	-0.311635
7954196	NM_145792	MGST1	NM_145792 // MGST1 // microsomal glutathione S-transferase 1 // 12p12.3-p12.1 // 4257 /	4.06E-05	-0.315767
7902102	---		---	0.00554594	0.211769
8002987	NM_020188	C16orf61	NM_020188 // C16orf61 // chromosome 16 open reading frame 61 // 16q23.2 // 56942 /// NM	0.000270648	-0.278071
7985253	NR_028330	C15orf37	NR_028330 // C15orf37 // chromosome 15 open reading frame 37 // 15q25.1 // 283687 /// N	0.000657551	-0.262574
7902104	NM_002600	PDE4B	NM_002600 // PDE4B // phosphodiesterase 4B, cAMP-specific // 1p31 // 5142 /// NM_001037	0.000544	-0.268073
8137232	NM_175571	GIMAP8	NM_175571 // GIMAP8 // GTPase, IMAP family member 8 // 7q36.1 // 155038 /// ENST0000030	0.00151671	0.240078
8175393	NM_004840	ARHGEF6	NM_004840 // ARHGEF6 // Rac/Cdc42 guanine nucleotide exchange factor (GEF) 6 // Xq26.3	5.59E-05	0.301586
8079079	NM_005385	NKTR	NM_005385 // NKTR // natural killer-tumor recognition sequence // 3p23-p21 // 4820 ///	0.00108539	0.249095
8114213	---		---	0.00275107	0.229955
8157216	NM_003358	UGCG	NM_003358 // UGCG // UDP-glucose ceramide glucosyltransferase // 9q31 // 7357 /// ENST0	1.30E-08	-0.396289
8082163	---		---	0.000891668	0.251342
7935146	NM_022451	NOC3L	NM_022451 // NOC3L // nucleolar complex associated 3 homolog (S. cerevisiae) // 10q23.3	0.00566492	-0.209777

8076365	NM_015704	PPPDE2	NM_015704 // PPPDE2 // PPPDE peptidase domain containing 2 // 22q13.2 // 27351 /// AF03	0.000602522	-0.256452
8113699	---		---	5.47E-05	0.306548
7996100	NM_170776	GPR97	NM_170776 // GPR97 // G protein-coupled receptor 97 // 16q21 // 222487 /// ENST00000333	0.000473507	-0.268208
7939590	NM_018389	SLC35C1	NM_018389 // SLC35C1 // solute carrier family 35, member C1 // 11p11.2 // 55343 /// NM_	0.00100115	-0.250741
7979196	NM_198066	GNPNAT1	NM_198066 // GNPNAT1 // glucosamine-phosphate N-acetyltransferase 1 // 14q22.1 // 64841	0.000197775	-0.274672
7924190	NM_144567	ANGEL2	NM_144567 // ANGEL2 // angel homolog 2 (Drosophila) // 1q32.3 // 90806 /// ENST00000366	0.00642994	0.211262
8066461	NM_006809	TOMM34	NM_006809 // TOMM34 // translocase of outer mitochondrial membrane 34 // --- // 10953 /	9.43E-05	-0.289253
8157976	NM_032293	GARNL3	NM_032293 // GARNL3 // GTPase activating Rap/RanGAP domain-like 3 // 9q33.3 // 84253 //	0.00245693	0.225677
7948982	NM_017878	HRASLS2	NM_017878 // HRASLS2 // HRAS-like suppressor 2 // 11q12.3 // 54979 /// ENST00000255695	0.00911721	-0.197835
7934178	NM_000281	PCBD1	NM_000281 // PCBD1 // pterin-4 alpha-carbinolamine dehydratase/dimerization cofactor of	0.00691683	-0.207119
7996569	---		---	0.00215059	0.237521
8155246	---		---	0.00496646	0.211845
8144420	NR_027000	LOC349196	NR_027000 // LOC349196 // hypothetical LOC349196 // 8p23.1 // 349196 /// AK094835 // LO	0.000386556	0.267167
8144494	NR_027000	LOC349196	NR_027000 // LOC349196 // hypothetical LOC349196 // 8p23.1 // 349196 /// AK094835 // LO	0.000386556	0.267167
8155376	NR_026801	FAM74A3	NR_026801 // FAM74A3 // family with sequence similarity 74, member A3 // 9p13.1 // 7284	0.00348838	0.221885

7958202	NM_018413	CHST11	NM_018413 // CHST11 // carbohydrate (chondroitin 4) sulfotransferase 11 // 12q // 50515	0.000201281	-0.271813
8132580	NM_006555	YKT6	NM_006555 // YKT6 // YKT6 v-SNARE homolog (S. cerevisiae) // 7p15.1 // 10652 /// ENST00	0.000102784	-0.294173
8102212	---		---	0.00546238	0.217899
7984263	NM_016395	PTPLAD1	NM_016395 // PTPLAD1 // protein tyrosine phosphatase-like A domain containing 1 // 15q2	0.0012592	-0.242862
8070341	NM_033656	BRWD1	NM_033656 // BRWD1 // bromodomain and WD repeat domain containing 1 // 21q22.2 // 54014	0.00600517	0.212692
8138721	NM_153631	HOXA3	NM_153631 // HOXA3 // homeobox A3 // 7p15.2 // 3200 /// NM_030661 // HOXA3 // homeobox	0.00543003	0.217124
8058905	NM_000634	CXCR1	NM_000634 // CXCR1 // chemokine (C-X-C motif) receptor 1 // 2q35 // 3577 /// ENST000002	0.0023505	-0.235316
7911341	---		---	3.57E-06	0.339549
7948033	---		---	0.00350075	0.223548
8175336	NM_001017436	CT45A4	NM_001017436 // CT45A4 // cancer/testis antigen family 45, member A4 // Xq26.3 // 44152	0.00257623	0.227538
7922523	---		---	6.79E-05	-0.306067
7920971	NM_144580	C1orf85	NM_144580 // C1orf85 // chromosome 1 open reading frame 85 // 1q22 // 112770 /// ENST00	0.000163655	-0.282435
8009227	NM_002805	PSMC5	NM_002805 // PSMC5 // proteasome (prosome, macropain) 26S subunit, ATPase, 5 // 17q23.3	0.00562379	-0.215265
7902913	NM_003503	CDC7	NM_003503 // CDC7 // cell division cycle 7 homolog (S. cerevisiae) // 1p22 // 8317 ///	0.00111899	-0.242293
7962516	NM_030674	SLC38A1	NM_030674 // SLC38A1 // solute carrier family 38, member 1 // 12q13.11 // 81539 /// NM_	0.00955218	-0.195379
8122426	NM_014721	PHACTR2	NM_014721 // PHACTR2 // phosphatase and actin regulator 2 // 6q24.2 // 9749 /// NM_0011	5.56E-05	0.300248
7946321	---		---	0.00591043	0.210742
7930663	NM_198496	VWA2	NM_198496 // VWA2 // von Willebrand factor A domain containing 2 // 10q25.3 // 340706 /	0.00171473	-0.237912

8165255	---		---	0.00442145	0.216793
8166355	NM_014927	CNKSR2	NM_014927 // CNKSR2 // connector enhancer of kinase suppressor of Ras 2 // Xp22.12 // 2	2.04E-05	0.319969
8059158	NM_024506	GLB1L	NM_024506 // GLB1L // galactosidase, beta 1-like // 2q35 // 79411 /// ENST00000295759 /	0.00546948	-0.211947
8050007	NM_012293	PXDN	NM_012293 // PXDN // peroxidasin homolog (Drosophila) // 2p25 // 7837 /// ENST000002528	0.00306864	-0.225315
8088911	NM_001128223	ZNF717	NM_001128223 // ZNF717 // zinc finger protein 717 // 3p12.3 // 100131827 /// ENST000004	0.000403883	0.263359
7986598	NM_001001413	GOLGA6L1	NM_001001413 // GOLGA6L1 // golgin A6 family-like 1 // 15q11.2 // 283767 /// ENST000003	0.000199431	0.278804
8064485	NM_018556	SIRPG	NM_018556 // SIRPG // signal-regulatory protein gamma // 20p13 // 55423 /// NM_00103950	0.00932848	0.202027
7986736	NM_001001413	GOLGA6L1	NM_001001413 // GOLGA6L1 // golgin A6 family-like 1 // 15q11.2 // 283767 /// ENST000003	0.000239552	0.275512
8143397	NM_015689	DENND2A	NM_015689 // DENND2A // DENN/MADD domain containing 2A // 7q34 // 27147 /// ENST0000027	0.00657304	0.207977
8002919	NM_001130089	KARS	NM_001130089 // KARS // lysyl-tRNA synthetase // 16q23.1 // 3735 /// NM_005548 // KARS	0.0018055	-0.242262
8151074	NM_002603	PDE7A	NM_002603 // PDE7A // phosphodiesterase 7A // 8q13 // 5150 /// NM_002604 // PDE7A // ph	0.000391526	0.271598
8144410	NR_027000	LOC349196	NR_027000 // LOC349196 // hypothetical LOC349196 // 8p23.1 // 349196 /// AK094835 // LO	0.000934636	0.250625
8053171	NM_032779	CCDC142	NM_032779 // CCDC142 // coiled-coil domain containing 142 // 2p13.1 // 84865 /// ENST00	0.0073018	0.201016
7991406	NM_003981	PRC1	NM_003981 // PRC1 // protein regulator of cytokinesis 1 // 15q26.1 // 9055 /// NM_19941	0.00904969	-0.198752
7897449	NM_025106	SPSB1	NM_025106 // SPSB1 // splA/ryanodine receptor domain and SOCS box containing 1 // 1p36.	1.43E-08	-0.397319

8071420	NM_000185	SERPIND1	NM_000185 // SERPIND1 // serpin peptidase inhibitor, clade D (heparin cofactor), member	0.00905285	-0.200741
8144412	NR_027000	LOC349196	NR_027000 // LOC349196 // hypothetical LOC349196 // 8p23.1 // 349196 /// AK094835 // LO	0.0004938	0.264043
8144414	NR_027000	LOC349196	NR_027000 // LOC349196 // hypothetical LOC349196 // 8p23.1 // 349196 /// AK094835 // LO	0.0004938	0.264043
8020762	NM_001944	DSG3	NM_001944 // DSG3 // desmoglein 3 // 18q12.1 // 1830 /// ENST00000257189 // DSG3 // des	0.00712477	-0.204371
8095697	NM_001511	CXCL1	NM_001511 // CXCL1 // chemokine (C-X-C motif) ligand 1 (melanoma growth stimulating act	3.83E-06	-0.33548
8108376	---		---	0.00271575	0.227745
8097118	ENST0000041541ε	FLJ45340	ENST00000415418 // FLJ45340 // hypothetical LOC402483 // 7q32.1 // 402483 /// AK097701	0.00300096	0.226425
7908388	NM_002922	RGS1	NM_002922 // RGS1 // regulator of G-protein signaling 1 // 1q31 // 5996 /// ENST0000036	0.00408168	-0.220306
8044263	NM_006267	RANBP2	NM_006267 // RANBP2 // RAN binding protein 2 // 2q12.3 // 5903 /// ENST00000283195 // R	0.00449116	-0.216039
8147112	NM_198584	CA13	NM_198584 // CA13 // carbonic anhydrase XIII // 8q21.2 // 377677 /// ENST00000321764 //	0.00600047	-0.209832
7996081	NM_201524	GPR56	NM_201524 // GPR56 // G protein-coupled receptor 56 // 16q13 // 9289 /// NM_201525 // G	0.0032922	-0.219073
8168749	NM_014467	SRPX2	NM_014467 // SRPX2 // sushi-repeat-containing protein, X-linked 2 // Xq21.33-q23 // 272	1.41E-05	-0.317376
8055294	---		---	0.000726765	0.25514
8175234	NM_001164617	GPC3	NM_001164617 // GPC3 // glypican 3 // Xq26.1 // 2719 /// NM_004484 // GPC3 // glypican	0.00111581	0.24835
8132943	NM_001762	CCT6A	NM_001762 // CCT6A // chaperonin containing TCP1, subunit 6A (zeta 1) // 7p11.2 // 908	0.00117056	-0.24538
8164428	NM_015679	TRUB2	NM_015679 // TRUB2 // TruB pseudouridine (psi) synthase homolog 2 (E. coli) // 9q34.11	9.79E-05	-0.294493

7929026	NM_001141945	ACTA2	NM_001141945 // ACTA2 // actin, alpha 2, smooth muscle, aorta // 10q23.3 // 59 /// NM_0	0.00486815	0.212098
8122058	NM_000045	ARG1	NM_000045 // ARG1 // arginase, liver // 6q23 // 383 /// ENST00000368087 // ARG1 // argi	8.92E-05	-0.299581
7986446	NM_000693	ALDH1A3	NM_000693 // ALDH1A3 // aldehyde dehydrogenase 1 family, member A3 // 15q26.3 // 220 //	5.87E-05	-0.297324
8006999	NR_033662	CSF3	NR_033662 // CSF3 // colony stimulating factor 3 (granulocyte) // 17q11.2-q12 // 1440 /	9.85E-05	-0.298527
8073015	NM_006855	KDELR3	NM_006855 // KDELR3 // KDEL (Lys-Asp-Glu-Leu) endoplasmic reticulum protein retention r	0.00129152	-0.241541
7938777	NM_005566	LDHA	NM_005566 // LDHA // lactate dehydrogenase A // 11p15.4 // 3939 /// NR_028500 // LDHA /	1.61E-07	-0.383112
7896748	---		---	0.00013201	0.287003
8152280	NM_013437	LRP12	NM_013437 // LRP12 // low density lipoprotein receptor-related protein 12 // 8q22.2 //	2.96E-05	-0.308107
7986741	NM_001001413	GOLGA6L1	NM_001001413 // GOLGA6L1 // golgin A6 family-like 1 // 15q11.2 // 283767 /// ENST000003	0.000263689	0.273701
8013671	NM_006461	SPAG5	NM_006461 // SPAG5 // sperm associated antigen 5 // 17q11.2 // 10615 /// ENST0000032176	0.000176295	-0.279318
8149361	NM_001136572	FAM90A7	NM_001136572 // FAM90A7 // family with sequence similarity 90, member A7 // 8p23.1 // 4	0.000609823	0.259854
8039896	NM_014512	KIR2DS1	NM_014512 // KIR2DS1 // killer cell immunoglobulin-like receptor, two domains, short cy	0.00388022	0.218761
7928890	NM_133447	AGAP11	NM_133447 // AGAP11 // ankyrin repeat and GTPase domain Arf GTPase activating protein 1	2.97E-05	0.308239
7904976	NM_001170755	NBPF15	NM_001170755 // NBPF15 // neuroblastoma breakpoint family, member 15 // 1q21.2 // 28456	0.0086273	0.200328
7937404	NM_173573	C11orf35	NM_173573 // C11orf35 // chromosome 11 open reading frame 35 // 11p15.5 // 256329 /// E	0.00581245	0.20489

8065758	AK096092	FLJ38773	AK096092 // FLJ38773 // hypothetical protein FLJ38773 // 20q11.22 // 284808	0.00084132	0.253405
8089299	NM_001777	CD47	NM_001777 // CD47 // CD47 molecule // 3q13.1-q13.2 // 961 /// NM_198793 // CD47 // CD47	0.00786506	0.202496
8070615	NM_018669	WDR4	NM_018669 // WDR4 // WD repeat domain 4 // 21q22.3 // 10785 /// NM_033661 // WDR4 // WD	0.00341268	-0.223055
7902127	NM_032291	SGIP1	NM_032291 // SGIP1 // SH3-domain GRB2-like (endophilin) interacting protein 1 // 1p31.3	0.00443129	0.218441
7927173	NM_145312	ZNF485	NM_145312 // ZNF485 // zinc finger protein 485 // 10q11.21 // 220992 /// ENST0000036180	0.00388603	0.219137
8104601	NM_006317	BASP1	NM_006317 // BASP1 // brain abundant, membrane attached signal protein 1 // 5p15.1 // 1	1.42E-06	-0.353013
8126588	NM_020750	XPO5	NM_020750 // XPO5 // exportin 5 // 6p21.1 // 57510 /// ENST00000265351 // XPO5 // expor	0.000573058	-0.264851
7954503	NM_004264	MED21	NM_004264 // MED21 // mediator complex subunit 21 // 12p11.23 // 9412 /// ENST000002828	0.00520853	-0.215398
8110803	NM_030782	CLPTM1L	NM_030782 // CLPTM1L // CLPTM1-like // 5p15.33 // 81037 /// ENST00000320895 // CLPTM1L	0.00935917	-0.202504
8169115	NM_198465	NRK	NM_198465 // NRK // Nik related kinase // Xq22.3 // 203447 /// BX538345 // NRK // Nik r	0.00423616	-0.217582
8023995	NM_005860	FSTL3	NM_005860 // FSTL3 // follistatin-like 3 (secreted glycoprotein) // 19p13 // 10272 ///	0.00356997	-0.225151
7909478	NM_014388	C1orf107	NM_014388 // C1orf107 // chromosome 1 open reading frame 107 // 1q32.2 // 27042 /// ENS	0.000376386	-0.265564
8162744	NM_003389	CORO2A	NM_003389 // CORO2A // coronin, actin binding protein, 2A // 9q22.3 // 7464 /// NM_0528	0.000362482	-0.266525
8106986	NM_014899	RHOBTB3	NM_014899 // RHOBTB3 // Rho-related BTB domain containing 3 // 5q15 // 22836 /// ENST00	0.00284782	-0.229661
7904478	AF172850	LOC51152	AF172850 // LOC51152 // melanoma antigen // --- // 51152	4.19E-05	0.302513

7923453	NM_006618	KDM5B	NM_006618 // KDM5B // lysine (K)-specific demethylase 5B // 1q32.1 // 10765 /// ENST000	0.0031323	-0.218217
8044391	NM_006343	MERTK	NM_006343 // MERTK // c-mer proto-oncogene tyrosine kinase // 2q14.1 // 10461 /// ENST0	1.37E-06	-0.358149
7969651	NM_006260	DNAJC3	NM_006260 // DNAJC3 // DnaJ (Hsp40) homolog, subfamily C, member 3 // 13q32.1 // 5611 /	5.41E-05	-0.297842
7933084	NM_005746	NAMPT	NM_005746 // NAMPT // nicotinamide phosphoribosyltransferase // 7q22.3 // 10135 /// ENS	3.83E-09	-0.434525
7963421	NM_005554	KRT6A	NM_005554 // KRT6A // keratin 6A // 12q12-q13 // 3853 /// ENST00000330722 // KRT6A // k	4.49E-05	-0.30576
7940717	NM_001012661	SLC3A2	NM_001012661 // SLC3A2 // solute carrier family 3 (activators of dibasic and neutral am	0.00333571	-0.226907
8085360	NM_003256	TIMP4	NM_003256 // TIMP4 // TIMP metallopeptidase inhibitor 4 // 3p25 // 7079 /// ENST0000028	0.000618169	-0.258227
8170060	NM_152582	CT45A2	NM_152582 // CT45A2 // cancer/testis antigen family 45, member A2 // Xq26.3 // 728911 /	0.00171657	0.236671
7978718	NM_006364	SEC23A	NM_006364 // SEC23A // Sec23 homolog A (S. cerevisiae) // 14q21.1 // 10484 /// ENST0000	0.00141271	-0.239513
8094870	NM_001080505	SHISA3	NM_001080505 // SHISA3 // shisa homolog 3 (Xenopus laevis) // 4p13 // 152573 /// ENST00	0.00304959	0.229541
8145122	NM_001128431	SLC39A14	NM_001128431 // SLC39A14 // solute carrier family 39 (zinc transporter), member 14 // 8	2.24E-08	-0.402649
7918048	---		---	0.00646004	0.208507
8019924	NM_006471	MYL12A	NM_006471 // MYL12A // myosin, light chain 12A, regulatory, non-sarcomeric // 18p11.31	0.0070696	0.202716
7985767	NM_022767	AEN	NM_022767 // AEN // apoptosis enhancing nuclease // 15q26.1 // 64782 /// ENST0000033281	0.00381441	-0.218213
8102988	NM_198682	GYPE	NM_198682 // GYPE // glycophorin E (MNS blood group) // 4q31.1 // 2996 /// NM_002102 //	0.000181079	0.28081
8154692	NM_000459	TEK	NM_000459 // TEK // TEK tyrosine kinase, endothelial // 9p21 // 7010 /// BC035514 // TE	0.00691844	0.207388

7988260	NM_032892	FRMD5	NM_032892 // FRMD5 // FERM domain containing 5 // 15q15.3 // 84978 /// ENST00000417257	0.000238866	-0.273882
8088369	---		---	0.00212311	0.23232
7927305	NM_001144000	AGAP5	NM_001144000 // AGAP5 // ArfGAP with GTPase domain, ankyrin repeat and PH domain 5 // 1	0.000268526	0.269458
8044473	NM_019014	POLR1B	NM_019014 // POLR1B // polymerase (RNA) I polypeptide B, 128kDa // 2q13 // 84172 /// NM	0.000762246	-0.258074
7958410	NM_007076	FICD	NM_007076 // FICD // FIC domain containing // 12q24.1 // 11153 /// ENST00000361549 // F	0.00250026	-0.230549
7946563	---		---	0.00010413	0.297694
8080084	NM_006010	MANF	NM_006010 // MANF // mesencephalic astrocyte-derived neurotrophic factor // 3p21.1 // 7	0.00769944	-0.204654
8101916	NM_152292	RG9MTD2	NM_152292 // RG9MTD2 // RNA (guanine-9-) methyltransferase domain containing 2 // 4q23	0.00480153	-0.215455
7979179	NM_014584	ERO1L	NM_014584 // ERO1L // ERO1-like (S. cerevisiae) // 14q22.1 // 30001 /// ENST00000395686	1.06E-06	-0.358057
8123644	NM_001069	TUBB2A	NM_001069 // TUBB2A // tubulin, beta 2A // 6p25 // 7280 /// ENST00000333628 // TUBB2A /	0.00012334	-0.292799
8055348	---		---	0.000254206	0.271949
8017555	NM_001433	ERN1	NM_001433 // ERN1 // endoplasmic reticulum to nucleus signaling 1 // 17q24.2 // 2081 //	0.000411411	-0.274338
8076298	---		---	0.00220652	0.229046
7904999	NM_001102663	NBPF16	NM_001102663 // NBPF16 // neuroblastoma breakpoint family, member 16 // 1q21.2 // 72893	0.00947543	0.199241
8028872	NM_001042544	LTBP4	NM_001042544 // LTBP4 // latent transforming growth factor beta binding protein 4 // 19	0.000284055	0.277589
8124518	NM_021066	HIST1H2AJ	NM_021066 // HIST1H2AJ // histone cluster 1, H2aj // 6p22.1 // 8331	0.0059339	-0.214097
8088903	---		---	0.00150849	0.241448
8129454	---		---	0.00546961	0.213063

8041644	NM_172069	PLEKHH2	NM_172069 // PLEKHH2 // pleckstrin homology domain containing, family H (with MyTH4 dom	4.15E-06	0.345542
8148280	NM_003129	SQLE	NM_003129 // SQLE // squalene epoxidase // 8q24.1 // 6713 /// ENST00000265896 // SQLE /	0.00615371	-0.208983
8096845	NM_001963	EGF	NM_001963 // EGF // epidermal growth factor // 4q25 // 1950 /// NM_001178130 // EGF //	0.00330495	-0.217473
8073422	NM_003216	TEF	NM_003216 // TEF // thyrotrophic embryonic factor // 22q13|22q13.2 // 7008 /// NM_00114	0.0011979	0.245236
8142345	NM_014705	DOCK4	NM_014705 // DOCK4 // dedicator of cytokinesis 4 // 7q31.1 // 9732 /// ENST00000437633	9.91E-06	0.327521
8142687	NM_005302	GPR37	NM_005302 // GPR37 // G protein-coupled receptor 37 (endothelin receptor type B-like) /	4.45E-05	-0.301514
8138741	NM_024014	HOXA6	NM_024014 // HOXA6 // homeobox A6 // 7p15.2 // 3203 /// ENST00000222728 // HOXA6 // hom	0.000363898	0.27178
7943347	---		---	0.00465278	0.213922
8056323	NM_018086	FIGN	NM_018086 // FIGN // fidgetin // 2q24.3 // 55137 /// ENST00000333129 // FIGN // fidgeti	0.0032008	0.227138
8149383	NR_036463	FAM90A25P	NR_036463 // FAM90A25P // family with sequence similarity 90 pseudogene // 8p23.1 // 38	0.000513915	0.263518
8037197	NM_198477	CXCL17	NM_198477 // CXCL17 // chemokine (C-X-C motif) ligand 17 // 19q13.2 // 284340 /// ENST0	0.000245538	-0.282831
8053668	NM_004836	EIF2AK3	NM_004836 // EIF2AK3 // eukaryotic translation initiation factor 2-alpha kinase 3 // 2p	2.43E-06	-0.349462
7904574	NM_001102663	NBPF16	NM_001102663 // NBPF16 // neuroblastoma breakpoint family, member 16 // 1q21.2 // 72893	0.00423699	0.218226
8096635	NM_003998	NFKB1	NM_003998 // NFKB1 // nuclear factor of kappa light polypeptide gene enhancer in B-cell	3.48E-06	-0.340445
8056060	NM_013450	BAZ2B	NM_013450 // BAZ2B // bromodomain adjacent to zinc finger domain, 2B // 2q24.2 // 29994	0.0085435	0.204676
8112070	---		---	0.00543381	0.211866

8173999	NM_212559	XKRX	NM_212559 // XKRX // XK, Kell blood group complex subunit-related, X-linked // Xq22.1 /	0.00189519	-0.241953
8165682	---		---	4.62E-06	0.338699
8149208	ENST0000042019{	OR7E125P	ENST00000420198 // OR7E125P // olfactory receptor, family 7, subfamily E, member 125 ps	0.000344491	0.267839
8167573	NM_001127345	GAGE12B	NM_001127345 // GAGE12B // G antigen 12B // Xp11.23 // 729428 /// NM_001098408 // GAGE1	0.00451027	0.21181
8099051	NM_003703	NOP14	NM_003703 // NOP14 // NOP14 nucleolar protein homolog (yeast) // 4p16.3 // 8602 /// ENS	3.88E-05	-0.315182
8091283	NM_182943	PLOD2	NM_182943 // PLOD2 // procollagen-lysine, 2-oxoglutarate 5-dioxygenase 2 // 3q24 // 535	0.000562927	-0.258434
7936871	NM_000274	OAT	NM_000274 // OAT // ornithine aminotransferase // 10q26 // 4942 /// NM_001171814 // OAT	0.000357611	-0.267034
8051030	NM_021095	SLC5A6	NM_021095 // SLC5A6 // solute carrier family 5 (sodium-dependent vitamin transporter),	1.22E-06	-0.362162
7964830	NR_033335	SNORA70G	NR_033335 // SNORA70G // small nucleolar RNA, H/ACA box 70G (retrotransposed) // 12q14	0.0026854	0.226123
7915910	NM_005764	PDZK1IP1	NM_005764 // PDZK1IP1 // PDZK1 interacting protein 1 // 1p33 // 10158 /// ENST000002943	0.00295922	-0.228487
8075160	---		---	0.00852372	0.200288
8102800	NM_014331	SLC7A11	NM_014331 // SLC7A11 // solute carrier family 7, (cationic amino acid transporter, y+ s	9.78E-06	-0.33496
8013771	NM_138463	TLCD1	NM_138463 // TLCD1 // TLC domain containing 1 // 17q11.2 // 116238 /// NM_001160407 //	0.000379147	-0.271673
8001455	---		---	0.00102618	0.248936
8103311	NM_000508	FGA	NM_000508 // FGA // fibrinogen alpha chain // 4q28 // 2243 /// NM_021871 // FGA // fibr	0.000280293	-0.279931
8112914	---		---	0.000725479	0.261702
8089038	---		---	0.000725479	0.261702
7953385	NM_002046	GAPDH	NM_002046 // GAPDH // glyceraldehyde-3-phosphate dehydrogenase // 12p13 // 2597 /// ENS	0.00538584	-0.21211

8071194	---		---	0.00165471	0.24118
8001099	NR_002827	HERC2P4	NR_002827 // HERC2P4 // hect domain and RLD 2 pseudogene 4 // 16p11.2 // 440362 /// NM_	0.00230097	0.230406
8018189	NM_181449	CD300E	NM_181449 // CD300E // CD300e molecule // 17q25.1 // 342510 /// ENST00000328630 // CD30	0.00209408	-0.239164
8142100	NM_020725	ATXN7L1	NM_020725 // ATXN7L1 // ataxin 7-like 1 // 7q22.3 // 222255 /// NM_138495 // ATXN7L1 //	0.0024336	0.235575
8027566	NM_001806	CEBPG	NM_001806 // CEBPG // CCAAT/enhancer binding protein (C/EBP), gamma // 19q13.11 // 1054	0.00481254	-0.219261
7942135	NM_018043	ANO1	NM_018043 // ANO1 // anoctamin 1, calcium activated chloride channel // 11q13.3 // 5510	0.000268627	0.270452
8096771	NM_021227	OSTC	NM_021227 // OSTC // oligosaccharyltransferase complex subunit // 4q25 // 58505 /// BC0	0.000153198	-0.288173
8075564	NM_001098527	RFPL2	NM_001098527 // RFPL2 // ret finger protein-like 2 // 22q12.3 // 10739 /// NM_001159545	0.00551633	0.210653
7943218	NM_015368	PANX1	NM_015368 // PANX1 // pannexin 1 // 11q21 // 24145 /// ENST00000227638 // PANX1 // pann	9.49E-09	-0.413759
8172531	NM_007213	PRAF2	NM_007213 // PRAF2 // PRA1 domain family, member 2 // Xp11.23 // 11230 /// ENST00000376	0.000366979	-0.265634
7929616	NM_005479	FRAT1	NM_005479 // FRAT1 // frequently rearranged in advanced T-cell lymphomas // 10q24.1 //	0.0052639	0.204975
7898012	NM_001098376	PRAMEF15	NM_001098376 // PRAMEF15 // PRAME family member 15 // 1p36.21 // 653619 /// NM_00101089	0.0034115	0.222903
8161407	NR_026801	FAM74A3	NR_026801 // FAM74A3 // family with sequence similarity 74, member A3 // 9p13.1 // 7284	0.000569455	0.259456
7985213	NM_000745	CHRNA5	NM_000745 // CHRNA5 // cholinergic receptor, nicotinic, alpha 5 // 15q24 // 1138 /// EN	0.00086987	-0.251834
8015460	NM_001096	ACLY	NM_001096 // ACLY // ATP citrate lyase // 17q21.2 // 47 /// NM_198830 // ACLY // ATP ci	4.59E-05	-0.306029
8018006	NM_080282	ABCA10	NM_080282 // ABCA10 // ATP-binding cassette, sub-family A (ABC1), member 10 // 17q24 //	6.14E-05	0.298232

7952046	NM_144765	MPZL2	NM_144765 // MPZL2 // myelin protein zero-like 2 // 11q24 // 10205 /// NM_005797 // MPZ	6.56E-05	-0.295732
8138718	NM_006735	HOXA2	NM_006735 // HOXA2 // homeobox A2 // 7p15.2 // 3199 /// ENST00000222718 // HOXA2 // hom	3.41E-05	0.313465
8078260	---		---	0.00243621	0.227934
7912808	NM_001039703	NBPF10	NM_001039703 // NBPF10 // neuroblastoma breakpoint family, member 10 // 1q21.1 // 10013	0.00133208	0.244452
8000028	NM_173475	DCUN1D3	NM_173475 // DCUN1D3 // DCN1, defective in cullin neddylation 1, domain containing 3 (S	2.85E-06	-0.347328
8024572	NM_002068	GNA15	NM_002068 // GNA15 // guanine nucleotide binding protein (G protein), alpha 15 (Gq clas	7.90E-07	-0.36987
8091715	NM_020169	LXN	NM_020169 // LXN // latexin // 3q25.32 // 56925 /// ENST00000264265 // LXN // latexin /	0.00180206	-0.236982
8174761	NR_027131	NKAPP1	NR_027131 // NKAPP1 // NFKB activating protein pseudogene 1 // Xq24 // 158801 /// BX537	0.00273445	0.228472
8001067	NR_002827	HERC2P4	NR_002827 // HERC2P4 // hect domain and RLD 2 pseudogene 4 // 16p11.2 // 440362 /// NM_	0.00123498	0.243582
8144488	NR_027000	LOC349196	NR_027000 // LOC349196 // hypothetical LOC349196 // 8p23.1 // 349196 /// AK094835 // LO	0.00331022	0.221757
7987027	ENST0000045080:	GOLGA8H	ENST00000450802 // GOLGA8H // golgin A8 family, member H // 15q13.2 // 728498 /// ENST0	0.000444501	0.266191
8076185	NM_175709	CBX7	NM_175709 // CBX7 // chromobox homolog 7 // 22q13.1 // 23492 /// ENST00000216133 // CBX	0.000612295	0.260985
8102594	NM_024873	TNIP3	NM_024873 // TNIP3 // TNFAIP3 interacting protein 3 // 4q27 // 79931 /// NM_001128843 /	0.000475649	-0.264383
8165698	---		---	6.59E-05	0.301164
7911339	---		---	6.59E-05	0.301164
7904254	NM_000701	ATP1A1	NM_000701 // ATP1A1 // ATPase, Na+/K+ transporting, alpha 1 polypeptide // 1p21 // 476	8.78E-06	-0.337779

8121861	NM_181782	NCOA7	NM_181782 // NCOA7 // nuclear receptor coactivator 7 // 6q22.32 // 135112 /// NM_001122	0.000682406	-0.259501
8021372	---		---	0.000188934	0.286147
7953697	---		---	0.000967463	-0.252508
7987114	NR_024074	GOLGA8IP	NR_024074 // GOLGA8IP // golgin A8 family, member I (pseudogene) // 15q11.2 // 283796 /	5.43E-05	0.30299
7981859	NR_024074	GOLGA8IP	NR_024074 // GOLGA8IP // golgin A8 family, member I (pseudogene) // 15q11.2 // 283796 /	1.63E-05	0.316528
8017964	NM_080284	ABCA6	NM_080284 // ABCA6 // ATP-binding cassette, sub-family A (ABC1), member 6 // 17q24.3 //	0.00115413	0.244356
8123520	BC118988	NCRNA00266	BC118988 // NCRNA00266 // non-protein coding RNA 266 // --- // 140849	0.00131103	0.237735
7919131	---		---	0.000484385	0.25681
8097262	NM_145207	SPATA5	NM_145207 // SPATA5 // spermatogenesis associated 5 // 4q28.1 // 166378 /// ENST0000027	0.000580708	0.263534
8123717	NM_006638	RPP40	NM_006638 // RPP40 // ribonuclease P/MRP 40kDa subunit // 6p25.1 // 10799 /// ENST00000	0.000481392	-0.264438
7959023	NM_001085481	MAP1LC3B2	NM_001085481 // MAP1LC3B2 // microtubule-associated protein 1 light chain 3 beta 2 // 1	0.00820535	-0.202789
8088813	NM_001126128	PROK2	NM_001126128 // PROK2 // prokineticin 2 // 3p13 // 60675 /// NM_021935 // PROK2 // prok	0.00345381	-0.227924
7928171	NM_003901	SGPL1	NM_003901 // SGPL1 // sphingosine-1-phosphate lyase 1 // 10q21 // 8879 /// ENST00000509	0.000739967	-0.256968
7982597	NM_003246	THBS1	NM_003246 // THBS1 // thrombospondin 1 // 15q15 // 7057 /// ENST00000260356 // THBS1 //	5.04E-09	-0.430122
7977018	NM_145725	TRAF3	NM_145725 // TRAF3 // TNF receptor-associated factor 3 // 14q32.32 // 7187 /// NM_14572	0.00251918	-0.230173
8108180	---		---	8.09E-06	0.328026

8169836	NM_003399	XPNPEP2	NM_003399 // XPNPEP2 // X-prolyl aminopeptidase (aminopeptidase P) 2, membrane-bound //	0.00565803	-0.207194
7933129	NR_026777	ZNF37BP	NR_026777 // ZNF37BP // zinc finger protein 37B, pseudogene // 10q11.21 // 100129482	0.00204913	0.233721
8170775	NM_006280	SSR4	NM_006280 // SSR4 // signal sequence receptor, delta (translocon-associated protein del	0.00514817	-0.21563
8094789	NM_014988	LIMCH1	NM_014988 // LIMCH1 // LIM and calponin homology domains 1 // 4p13 // 22998 /// NM_0011	4.75E-05	0.306257
7923976	NR_029518	MIR29B2	NR_029518 // MIR29B2 // microRNA 29b-2 // 1q32.2 // 407025	0.000159519	0.27399
8066247	NR_027241	LOC388796	NR_027241 // LOC388796 // hypothetical LOC388796 // 20q11.23 // 388796 /// NR_015366 //	0.00700579	-0.205762
8116245	BC063116	CLK4	BC063116 // CLK4 // CDC-like kinase 4 // 5q35 // 57396	0.00137683	0.247332
7945232	NM_139055	ADAMTS15	NM_139055 // ADAMTS15 // ADAM metallopeptidase with thrombospondin type 1 motif, 15 //	0.000883527	-0.255418
8069532	NM_006948	HSPA13	NM_006948 // HSPA13 // heat shock protein 70kDa family, member 13 // 21q11.1|21q11 // 6	0.00146835	-0.240842
8155600	---		---	0.00113599	0.242533
8155598	---		---	0.00113599	0.242533
8112881	---		---	0.00466369	0.216828
7909839	---		---	0.00452431	0.217567
8013523	---		---	0.000147018	0.285958
7983763	NM_002748	MAPK6	NM_002748 // MAPK6 // mitogen-activated protein kinase 6 // 15q21 // 5597 /// ENST00000	0.00085696	-0.249426
8144786	NM_003046	SLC7A2	NM_003046 // SLC7A2 // solute carrier family 7 (cationic amino acid transporter, y+ sys	7.64E-05	-0.297105
8161004	NM_001782	CD72	NM_001782 // CD72 // CD72 molecule // 9p13.3 // 971 /// ENST00000396757 // CD72 // CD72	0.00697639	-0.20206

7910611	NM_002245	KCNK1	NM_002245 // KCNK1 // potassium channel, subfamily K, member 1 // 1q42-q43 // 3775 ///	1.39E-07	-0.386904
7944799	NM_001001953	OR10G9	NM_001001953 // OR10G9 // olfactory receptor, family 10, subfamily G, member 9 // 11q24	0.000450774	0.267182
8108720	NM_019119	PCDHB9	NM_019119 // PCDHB9 // protocadherin beta 9 // 5q31 // 56127 /// AF217749 // PCDHB9 //	0.00449355	0.218552
8170068	NM_001017435	CT45A3	NM_001017435 // CT45A3 // cancer/testis antigen family 45, member A3 // Xq26.3 // 44151	0.00215623	0.232017
7976560	NM_000623	BDKRB2	NM_000623 // BDKRB2 // bradykinin receptor B2 // 14q32.1-q32.2 // 624 /// ENST000003060	0.00445248	-0.214918
8075569	NM_014227	SLC5A4	NM_014227 // SLC5A4 // solute carrier family 5 (low affinity glucose cotransporter), me	0.0014029	0.240436
7931977	NM_030569	ITIH5	NM_030569 // ITIH5 // inter-alpha (globulin) inhibitor H5 // 10p14 // 80760 /// NM_0328	0.00247264	0.231409
7980861	NM_024764	CATSPERB	NM_024764 // CATSPERB // cation channel, sperm-associated, beta // 14q32.12 // 79820 //	0.000394825	-0.268114
8131803	NM_000600	IL6	NM_000600 // IL6 // interleukin 6 (interferon, beta 2) // 7p21 // 3569 /// ENST00000404	3.28E-05	-0.314581
8109157	NR_029684	MIR143	NR_029684 // MIR143 // microRNA 143 // 5q32 // 406935 /// NR_027180 // LOC728264 // hyp	0.00026957	0.271068
8129120	NM_001085480	FAM162B	NM_001085480 // FAM162B // family with sequence similarity 162, member B // 6q22.1 // 2	0.00559853	0.211088
8029465	NM_005178	BCL3	NM_005178 // BCL3 // B-cell CLL/lymphoma 3 // 19q13.1-q13.2 // 602 /// BC064993 // BCL3	1.59E-06	-0.357147
8169949	NM_016542	MST4	NM_016542 // MST4 // serine/threonine protein kinase MST4 // Xq26.2 // 51765 /// NM_001	0.00616525	-0.204251
8100601	---		---	0.000643881	0.255871
7898007	NM_001013407	PRAMEF5	NM_001013407 // PRAMEF5 // PRAME family member 5 // 1p36.21 // 343068 /// NM_001098376	0.00196785	0.235229

7912575	NM_001013407	PRAMEF5	NM_001013407 // PRAMEF5 // PRAME family member 5 // 1p36.21 // 343068 /// NM_001010889	0.00196785	0.23523
8108422	---		---	0.0073289	0.202558
7977854	NM_032876	JUB	NM_032876 // JUB // jub, ajuba homolog (Xenopus laevis) // 14q11.2 // 84962 /// NM_1980	0.00694124	0.207057
8016708	NM_018509	LRRC59	NM_018509 // LRRC59 // leucine rich repeat containing 59 // 17q21.33 // 55379 /// ENST0	9.29E-07	-0.363951
8148982	AK128318	LOC389607	AK128318 // LOC389607 // hypothetical LOC389607 // 8p23.3 // 389607 /// ENST00000382819	0.00485166	0.219561
8042038	NR_002229	RPL23AP32	NR_002229 // RPL23AP32 // ribosomal protein L23a pseudogene 32 // 2p16.2 // 56969	6.04E-06	0.332712
8145244	NM_003841	TNFRSF10C	NM_003841 // TNFRSF10C // tumor necrosis factor receptor superfamily, member 10c, decoy	0.0087302	-0.19896
8051215	NM_022128	RBKS	NM_022128 // RBKS // ribokinase // 2p23.3 // 64080 /// ENST00000302188 // RBKS // ribok	0.00142481	-0.242921
7999532	NM_002094	GSPT1	NM_002094 // GSPT1 // G1 to S phase transition 1 // 16p13.1 // 2935 /// NM_001130006 //	0.000206589	-0.283006
8156897	BC008993	C9orf30	BC008993 // C9orf30 // chromosome 9 open reading frame 30 // 9q31.1 // 91283 /// AY5983	0.00394163	-0.219097
7995793	NR_001447	MT1L	NR_001447 // MT1L // metallothionein 1L (gene/pseudogene) // 16q13 // 4500 /// X97261 /	0.000564953	-0.265833
8144496	ENST0000042019{	OR7E125P	ENST00000420198 // OR7E125P // olfactory receptor, family 7, subfamily E, member 125 ps	0.000367124	0.266494
8137008	AK290098	C7orf11	AK290098 // C7orf11 // chromosome 7 open reading frame 11 // 7p14.1 // 136647	0.000742019	0.262203
7912802	BC036435	LOC100132147	BC036435 // LOC100132147 // hypothetical LOC100132147 // 1p36.13 // 100132147 /// AK125	0.00493585	0.21556

8118142	NM_000594	TNF	NM_000594 // TNF // tumor necrosis factor // 6p21.3 // 7124 /// ENST00000376122 // TNF	0.00730528	0.20829
8177983	NM_000594	TNF	NM_000594 // TNF // tumor necrosis factor // 6p21.3 // 7124 /// ENST00000376122 // TNF	0.00730528	0.20829
8179263	NM_000594	TNF	NM_000594 // TNF // tumor necrosis factor // 6p21.3 // 7124 /// ENST00000376122 // TNF	0.00730528	0.20829
7963261	AK292677	LOC494150	AK292677 // LOC494150 // prohibitin pseudogene // 12q13 // 494150	0.00813818	0.196798
7909144	AK094426	LOC284581	AK094426 // LOC284581 // hypothetical protein LOC284581 // 1q32.1 // 284581 /// ENST000	0.00135398	0.249213
7986601	---		---	0.00517808	-0.215173
7986739	---		---	0.00517808	-0.215173
7959146	---		---	0.00680649	-0.211941
7962327	NM_052885	SLC2A13	NM_052885 // SLC2A13 // solute carrier family 2 (facilitated glucose transporter), memb	4.59E-05	-0.309829
8099897	NM_003359	UGDH	NM_003359 // UGDH // UDP-glucose 6-dehydrogenase // 4p15.1 // 7358 /// NM_001184700 //	8.09E-06	-0.325238
8038967	NM_001105549	ZNF83	NM_001105549 // ZNF83 // zinc finger protein 83 // 19q13.3 // 55769 /// NM_001105551 //	0.000658005	0.258371
7953981	NM_001987	ETV6	NM_001987 // ETV6 // ets variant 6 // 12p13 // 2120 /// ENST00000266427 // ETV6 // ets	0.00036544	-0.268965
8031260	NM_153443	KIR3DL3	NM_153443 // KIR3DL3 // killer cell immunoglobulin-like receptor, three domains, long c	0.00260725	0.228917
8027760	NM_005031	FXYD1	NM_005031 // FXYD1 // FXYD domain containing ion transport regulator 1 // 19q13.1 // 53	0.00108176	0.252575
7919438	NM_001102663	NBPF16	NM_001102663 // NBPF16 // neuroblastoma breakpoint family, member 16 // 1q21.2 // 72893	0.00440391	0.217644
8090988	NM_024491	CEP70	NM_024491 // CEP70 // centrosomal protein 70kDa // 3q22.3 // 80321 /// ENST00000484888	0.00241231	-0.233569
8150960	---		---	0.010308	0.194265

8021695	NM_152721	DOK6	NM_152721 // DOK6 // docking protein 6 // 18q22.2 // 220164 /// ENST00000382713 // DOK6	0.00156096	0.241665
8153201	NM_012154	EIF2C2	NM_012154 // EIF2C2 // eukaryotic translation initiation factor 2C, 2 // 8q24 // 27161	0.00122238	-0.251022
8105302	NM_006350	FST	NM_006350 // FST // follistatin // 5q11.2 // 10468 /// NM_013409 // FST // follistatin	4.67E-05	-0.295518
8023696	NM_006919	SERPINB3	NM_006919 // SERPINB3 // serpin peptidase inhibitor, clade B (ovalbumin), member 3 // 1	0.00424099	-0.220052
7964548	NM_005371	METTL1	NM_005371 // METTL1 // methyltransferase like 1 // 12q13 // 4234 /// NM_023033 // METTL	2.73E-05	-0.31483
7971161	---		---	0.00942208	0.197808
7923034	NM_003783	B3GALT2	NM_003783 // B3GALT2 // UDP-Gal:betaGlcNAc beta 1,3-galactosyltransferase, polypeptide	0.00791926	0.206528
8020254	NM_031216	SEH1L	NM_031216 // SEH1L // SEH1-like (S. cerevisiae) // 18p11.21 // 81929 /// NM_001013437 /	1.18E-05	-0.328099
8042381	NM_020143	PNO1	NM_020143 // PNO1 // partner of NOB1 homolog (S. cerevisiae) // 2p14 // 56902 /// ENST0	2.48E-06	-0.355627
8040639	NM_000183	HADHB	NM_000183 // HADHB // hydroxyacyl-CoA dehydrogenase/3-ketoacyl-CoA thiolase/enoyl-CoA h	0.0101378	-0.198329
7982284	NR_033933	LOC653075	NR_033933 // LOC653075 // golgin A8 family, member A pseudogene // 15q13.2 // 653075 //	0.00283416	0.22214
8141342	NM_000765	CYP3A7	NM_000765 // CYP3A7 // cytochrome P450, family 3, subfamily A, polypeptide 7 // 7q21-q2	1.20E-05	0.328818
8133209	NR_003666	SPDYE7P	NR_003666 // SPDYE7P // speedy homolog E7 (Xenopus laevis), pseudogene // 7q11.23 // 44	3.68E-05	0.304679
8086595	NM_005283	XCR1	NM_005283 // XCR1 // chemokine (C motif) receptor 1 // 3p21.3-p21.1|3p21.3 // 2829 ///	0.00416525	0.223428
7990636	NR_026813	C15orf5	NR_026813 // C15orf5 // chromosome 15 open reading frame 5 // 15q23-q24 // 81698 /// AF	0.000642862	0.257739
7957890	NM_014503	UTP20	NM_014503 // UTP20 // UTP20, small subunit (SSU) processome component, homolog (yeast)	0.000324201	-0.274086

8108301	NM_005733	KIF20A	NM_005733 // KIF20A // kinesin family member 20A // 5q31 // 10112 /// NM_004661 // CDC2	0.00229564	-0.232312
8144395	NM_201402	USP17L2	NM_201402 // USP17L2 // ubiquitin specific peptidase 17-like 2 // 8p23.1 // 377630 ///	1.25E-05	0.323708
7956878	NM_007199	IRAK3	NM_007199 // IRAK3 // interleukin-1 receptor-associated kinase 3 // 12q14.3 // 11213 //	2.17E-05	-0.329439
8089759	NM_018266	TMEM39A	NM_018266 // TMEM39A // transmembrane protein 39A // 3q13.33 // 55254 /// ENST000003191	0.000186484	-0.280921
7900510	NM_001905	CTPS	NM_001905 // CTPS // CTP synthase // 1p34.1 // 1503 /// ENST00000372621 // CTPS // CTP	0.00171862	-0.243106
8094778	NM_004181	UCHL1	NM_004181 // UCHL1 // ubiquitin carboxyl-terminal esterase L1 (ubiquitin thiolesterase)	0.00551909	-0.206149
7989718	NM_016563	RASL12	NM_016563 // RASL12 // RAS-like, family 12 // 15q11.2-q22.33 // 51285 /// ENST000002200	0.00605899	0.212932
7986569	NR_036432	HERC2P3	NR_036432 // HERC2P3 // hect domain and RLD 2 pseudogene 3 // 15q11.1 // 283755 /// NR_	0.00788306	0.201894
8159006	NR_000017	SNORD36B	NR_000017 // SNORD36B // small nucleolar RNA, C/D box 36B // 9q34 // 26814	0.0100223	-0.198942
8101701	NM_152542	PPM1K	NM_152542 // PPM1K // protein phosphatase, Mg2+/Mn2+ dependent, 1K // 4q22.1 // 152926	0.000886103	0.2526
8090448	NM_003707	RUVBL1	NM_003707 // RUVBL1 // RuvB-like 1 (E. coli) // 3q21 // 8607 /// ENST00000322623 // RUV	0.00378029	-0.22035
8150204	---		---	0.000527277	-0.269809
8137485	NM_001039350	DPP6	NM_001039350 // DPP6 // dipeptidyl-peptidase 6 // 7q36.2 // 1804 /// NM_001936 // DPP6	1.20E-05	0.323736
7953735	NR_027000	LOC349196	NR_027000 // LOC349196 // hypothetical LOC349196 // 8p23.1 // 349196 /// AK094835 // LO	0.00126542	0.244732
8107706	NM_005573	LMNB1	NM_005573 // LMNB1 // lamin B1 // 5q23.2 // 4001 /// NM_001198557 // LMNB1 // lamin B1	4.65E-05	-0.309783
8144422	ENST0000042019{	OR7E125P	ENST00000420198 // OR7E125P // olfactory receptor, family 7, subfamily E, member 125 ps	0.000768063	0.25162

7972190	NM_022118	RBM26	NM_022118 // RBM26 // RNA binding motif protein 26 // 13q31.1 // 64062 /// ENST00000267	0.00975093	0.198961
8029907	NM_001736	C5AR1	NM_001736 // C5AR1 // complement component 5a receptor 1 // 19q13.3-q13.4 // 728 /// EN	0.00100554	-0.250978
7990815	NM_001100880	ST20	NM_001100880 // ST20 // suppressor of tumorigenicity 20 // 15q25.1 // 400410 /// NR_028	4.75E-05	-0.314591
7912567	NM_001013407	PRAMEF5	NM_001013407 // PRAMEF5 // PRAME family member 5 // 1p36.21 // 343068 /// NM_001010889	0.00126478	0.245615
7925622	NM_015446	AHCTF1	NM_015446 // AHCTF1 // AT hook containing transcription factor 1 // 1q44 // 25909 /// A	0.00381047	-0.219364
8023688	NM_002974	SERPINB4	NM_002974 // SERPINB4 // serpin peptidase inhibitor, clade B (ovalbumin), member 4 // 1	0.00118931	-0.247456
7919271	NM_183372	NBPF11	NM_183372 // NBPF11 // neuroblastoma breakpoint family, member 11 // 1q21.1 // 200030 /	0.00149798	0.241503
7925876	NM_002627	PFKP	NM_002627 // PFKP // phosphofructokinase, platelet // 10p15.3-p15.2 // 5214 /// ENST000	0.000533755	-0.263827
7898040	NM_001098376	PRAMEF15	NM_001098376 // PRAMEF15 // PRAME family member 15 // 1p36.21 // 653619 /// NM_00101089	0.00234569	0.231942
8133155	NM_003596	TPST1	NM_003596 // TPST1 // tyrosylprotein sulfotransferase 1 // 7q11.21 // 8460 /// ENST0000	1.43E-06	-0.350727
8008629	NM_003647	DGKE	NM_003647 // DGKE // diacylglycerol kinase, epsilon 64kDa // 17q22 // 8526 /// ENST0000	0.000944881	0.249955
8031933	NM_001009	RPS5	NM_001009 // RPS5 // ribosomal protein S5 // 19q13.4 // 6193 /// ENST00000196551 // RPS	0.00860555	-0.20545
7952795	---		---	0.00140308	0.243768
7982230	NR_024074	GOLGA8IP	NR_024074 // GOLGA8IP // golgin A8 family, member I (pseudogene) // 15q11.2 // 283796 /	0.000390205	0.265292

7951977	NM_001164836	FXYD6	NM_001164836 // FXYD6 // FXYD domain containing ion transport regulator 6 // 11q23.3 //	0.000446911	0.267841
7946328	---		---	0.00459937	0.219901
7912145	NM_001561	TNFRSF9	NM_001561 // TNFRSF9 // tumor necrosis factor receptor superfamily, member 9 // 1p36 //	0.00317391	-0.220508
8166632	NM_001128127	GK	NM_001128127 // GK // glycerol kinase // Xp21.3 // 2710 /// NM_203391 // GK // glycerol	4.99E-08	-0.402424
8023656	NM_002035	KDSR	NM_002035 // KDSR // 3-ketodihydrosphingosine reductase // 18q21.3 // 2531 /// ENST0000	0.00557095	-0.213548
8137863	---		---	0.00146374	0.243463
8105663	NM_020726	NLN	NM_020726 // NLN // neurolysin (metallopeptidase M3 family) // 5q12.3 // 57486 /// ENST	0.00257433	-0.223636
8047036	---		---	0.000697559	0.253799
8026861	NM_014256	B3GNT3	NM_014256 // B3GNT3 // UDP-GlcNAc:betaGal beta-1,3-N-acetylglucosaminyltransferase 3 //	0.00880634	-0.200642
7962000	NM_198965	PTHLH	NM_198965 // PTHLH // parathyroid hormone-like hormone // 12p12.1-p11.2 // 5744 /// NM_	0.00266154	-0.22343
8048749	NM_080671	KCNE4	NM_080671 // KCNE4 // potassium voltage-gated channel, Isk-related family, member 4 //	0.00217822	-0.229796
8115907	NM_016391	NOP16	NM_016391 // NOP16 // NOP16 nucleolar protein homolog (yeast) // 5q35.2 // 51491 /// EN	5.03E-05	-0.305379
7971780	NM_002498	NEK3	NM_002498 // NEK3 // NIMA (never in mitosis gene a)-related kinase 3 // 13q14.13 // 475	0.000577241	0.259257
8044499	NM_005415	SLC20A1	NM_005415 // SLC20A1 // solute carrier family 20 (phosphate transporter), member 1 // 2	1.86E-05	-0.31836
7942503	NM_016147	PPME1	NM_016147 // PPME1 // protein phosphatase methylesterase 1 // 11q13.4 // 51400 /// ENST	2.95E-05	-0.314791
8108099	NM_021982	SEC24A	NM_021982 // SEC24A // SEC24 family, member A (S. cerevisiae) // 5q31.1 // 10802 /// EN	1.73E-06	-0.352005

7961151	NM_007360	KLRK1	NM_007360 // KLRK1 // killer cell lectin-like receptor subfamily K, member 1 // 12p13.2	0.0081442	0.204498
8149356	NM_201402	USP17L2	NM_201402 // USP17L2 // ubiquitin specific peptidase 17-like 2 // 8p23.1 // 377630 ///	1.79E-05	0.318246
8039820	NM_153443	KIR3DL3	NM_153443 // KIR3DL3 // killer cell immunoglobulin-like receptor, three domains, long c	0.00374598	0.221225
7959834	NM_144669	GLT1D1	NM_144669 // GLT1D1 // glycosyltransferase 1 domain containing 1 // 12q24.33 // 144423	0.00579117	-0.215661
7922889	NM_006469	IVNS1ABP	NM_006469 // IVNS1ABP // influenza virus NS1A binding protein // 1q25.1-q31.1 // 10625	3.61E-06	-0.346624
8111455	NR_027026	GUSBP1	NR_027026 // GUSBP1 // glucuronidase, beta pseudogene 1 // 5p14.3 // 728411 /// NR_0035	6.67E-09	0.420803
8052331	NM_033109	PNPT1	NM_033109 // PNPT1 // polyribonucleotide nucleotidyltransferase 1 // 2p15 // 87178 ///	0.00015574	-0.286898
7930074	NM_002502	NFKB2	NM_002502 // NFKB2 // nuclear factor of kappa light polypeptide gene enhancer in B-cell	0.0004079	-0.271348
8020411	NM_006938	SNRPD1	NM_006938 // SNRPD1 // small nuclear ribonucleoprotein D1 polypeptide 16kDa // 18q11.2	0.00193192	-0.239165
7963406	NM_005555	KRT6B	NM_005555 // KRT6B // keratin 6B // 12q12-q13 // 3854 /// ENST00000252252 // KRT6B // k	0.000129655	-0.288332
8171435	NM_003662	PIR	NM_003662 // PIR // pirin (iron-binding nuclear protein) // Xp22.2 // 8544 /// NM_00101	0.00615941	-0.213431
7931155	NM_001029888	FAM24A	NM_001029888 // FAM24A // family with sequence similarity 24, member A // 10q26.13 // 1	0.00238058	0.235212
8071042	AK124122	LOC100288884	AK124122 // LOC100288884 // hypothetical LOC100288884 // 21p11.2 // 100288884 /// ENST0	0.000882245	0.246516
7906863	NM_003115	UAP1	NM_003115 // UAP1 // UDP-N-acteylglucosamine pyrophosphorylase 1 // 1q23.3 // 6675 ///	6.56E-08	-0.38818

8110265	NM_213647	FGFR4	NM_213647 // FGFR4 // fibroblast growth factor receptor 4 // 5q35.1-qter // 2264 /// NM	0.000755896	0.257426
8108399	---		---	0.00274213	0.227174
8086344	NM_001337	CX3CR1	NM_001337 // CX3CR1 // chemokine (C-X3-C motif) receptor 1 // 3p21|3p21.3 // 1524 /// N	2.31E-05	0.322816
8172254	NM_176819	CXorf36	NM_176819 // CXorf36 // chromosome X open reading frame 36 // Xp11.3 // 79742 /// NM_02	0.00292376	0.227416
8149243	NM_201402	USP17L2	NM_201402 // USP17L2 // ubiquitin specific peptidase 17-like 2 // 8p23.1 // 377630 ///	1.28E-05	0.323415
8130038	NM_001042683	SHPRH	NM_001042683 // SHPRH // SNF2 histone linker PHD RING helicase // 6q24.3 // 257218 ///	0.000843309	0.257555
8023252	NM_000985	RPL17	NM_000985 // RPL17 // ribosomal protein L17 // 18q21 // 6139 /// NM_001035006 // RPL17	0.0029807	-0.232555
7996891	NM_032830	CIRH1A	NM_032830 // CIRH1A // cirrhosis, autosomal recessive 1A (cirhin) // 16q22.1 // 84916 /	9.42E-08	-0.382877
7989315	NM_004492	GTF2A2	NM_004492 // GTF2A2 // general transcription factor IIA, 2, 12kDa // 15q22.2 // 2958 //	0.00987234	-0.202897
7983922	---		---	0.000166771	-0.287322
8165653	ENST0000036139(	ND1	ENST00000361390 // ND1 // NADH dehydrogenase, subunit 1 (complex I) // --- // 4535 ///	0.000927743	0.252521
8104504	---		---	0.000452807	0.271093
7985134	NM_005530	IDH3A	NM_005530 // IDH3A // isocitrate dehydrogenase 3 (NAD+) alpha // 15q25.1-q25.2 // 3419	4.90E-06	-0.33453
7996934	NM_016101	NIP7	NM_016101 // NIP7 // nuclear import 7 homolog (S. cerevisiae) // 16q22.1 // 51388 /// E	0.000308467	-0.267828
7919351	NM_183372	NBPF11	NM_183372 // NBPF11 // neuroblastoma breakpoint family, member 11 // 1q21.1 // 200030 /	0.00164352	0.239519
7930413	NM_004419	DUSP5	NM_004419 // DUSP5 // dual specificity phosphatase 5 // 10q25 // 1847 /// ENST000003695	0.000725639	-0.260288

7915472	NM_006516	SLC2A1	NM_006516 // SLC2A1 // solute carrier family 2 (facilitated glucose transporter), membe	0.000832663	-0.254886
7979824	NM_001130004	ACTN1	NM_001130004 // ACTN1 // actinin, alpha 1 // 14q24.1-q24.2|14q24|14q22-q24 // 87 /// NM	0.000222417	-0.27872
8156358	---		---	0.000189898	0.281456
7968351	NM_032849	C13orf33	NM_032849 // C13orf33 // chromosome 13 open reading frame 33 // 13q12.3 // 84935 /// EN	1.32E-07	-0.384007
7950885	NM_012193	FZD4	NM_012193 // FZD4 // frizzled homolog 4 (Drosophila) // 11q14.2 // 8322 /// ENST0000030	0.00141801	0.237189
8071559	NM_022044	SDF2L1	NM_022044 // SDF2L1 // stromal cell-derived factor 2-like 1 // 22q11.21 // 23753 /// EN	0.000784369	-0.253598
8073949	NM_001135101	CRELD2	NM_001135101 // CRELD2 // cysteine-rich with EGF-like domains 2 // 22q13.33 // 79174 //	0.00115466	-0.251156
7995328	---		---	0.0018086	0.241364
7964658	---		---	0.00854323	0.205231
8043500	---		---	0.000138376	0.286434
8074131	NM_001080420	SHANK3	NM_001080420 // SHANK3 // SH3 and multiple ankyrin repeat domains 3 // 22q13.3 // 85358	0.00742984	0.203124
7989195	---		---	0.00568341	0.20995
7966343	---		---	0.00910822	0.201338
7990827	ENST0000044768?	LOC100133746	ENST00000447682 // LOC100133746 // hypothetical LOC100133746 // --- // 100133746 /// AK	0.00683231	0.204764
7976642	---		---	1.47E-06	0.353941
8065738	NM_000687	AHCY	NM_000687 // AHCY // adenosylhomocysteinase // 20cen-q13.1 // 191 /// NM_001161766 // A	0.000435394	-0.272197
8075217	NM_001127	AP1B1	NM_001127 // AP1B1 // adaptor-related protein complex 1, beta 1 subunit // 22q12|22q12.	0.00261562	-0.223416
8133654	NM_001099435	SPDYE5	NM_001099435 // SPDYE5 // speedy homolog E5 (Xenopus laevis) // 7q11.23 // 442590 /// N	6.00E-05	0.29892
7990818	NM_001114735	BCL2A1	NM_001114735 // BCL2A1 // BCL2-related protein A1 // 15q24.3 // 597 /// NM_004049 // BC	0.00165589	-0.24121

7982256	NR_024074	GOLGA8IP	NR_024074 // GOLGA8IP // golgin A8 family, member I (pseudogene) // 15q11.2 // 283796 /	0.000330948	0.270855
7998823	---		---	0.00820255	0.203988
7998841	---		---	0.00820255	0.203988
8151768	NM_001198625	RUNX1T1	NM_001198625 // RUNX1T1 // runt-related transcription factor 1; translocated to, 1 (cyc	2.20E-05	0.322908
7922807	NM_015101	GLT25D2	NM_015101 // GLT25D2 // glycosyltransferase 25 domain containing 2 // 1q25 // 23127 ///	0.000375205	0.27445
8032212	AK127759	NDUFS7	AK127759 // NDUFS7 // NADH dehydrogenase (ubiquinone) Fe-S protein 7, 20kDa (NADH-coenz	0.000531408	0.254878
7981750	AK127783	LOC400968	AK127783 // LOC400968 // hypothetical LOC400968 // 15q11.2 // 400968	0.00473258	0.212938
8170971	NM_001363	DKC1	NM_001363 // DKC1 // dyskeratosis congenita 1, dyskerin // Xq28 // 1736 /// NM_00114246	0.000111915	-0.2968
8043187	NM_005911	MAT2A	NM_005911 // MAT2A // methionine adenosyltransferase II, alpha // 2p11.2 // 4144 /// EN	0.000104068	-0.292356
8112491	NR_034021	SMA5	NR_034021 // SMA5 // glucuronidase, beta pseudogene // 5q13 // 11042 /// NR_027386 // G	5.20E-08	0.397515
8045688	NM_007115	TNFAIP6	NM_007115 // TNFAIP6 // tumor necrosis factor, alpha-induced protein 6 // 2q23.3 // 713	7.17E-08	-0.392228
7990151	NM_182470	PKM2	NM_182470 // PKM2 // pyruvate kinase, muscle // 15q22 // 5315 /// NM_182471 // PKM2 //	0.00420731	-0.216347
7956443	NM_004990	MARS	NM_004990 // MARS // methionyl-tRNA synthetase // 12q13.2 // 4141 /// ENST00000262027 /	0.00182507	-0.243642
8168447	BC001220	CXorf26	BC001220 // CXorf26 // chromosome X open reading frame 26 // Xq13.3 // 51260 /// BC0518	0.0088198	-0.199777
8095562	---		---	0.00784459	0.202864
7960438	NM_020373	ANO2	NM_020373 // ANO2 // anoctamin 2 // 12p13.3 // 57101 /// ENST00000327087 // ANO2 // ano	8.28E-05	0.292929

8042356	NM_002398	MEIS1	NM_002398 // MEIS1 // Meis homeobox 1 // 2p14 // 4211 /// ENST00000272369 // MEIS1 // M	0.000230506	0.275262
7947189	NM_030771	CCDC34	NM_030771 // CCDC34 // coiled-coil domain containing 34 // 11p14.1 // 91057 /// NM_0806	0.00560629	-0.212803
8174103	NM_001128127	GK	NM_001128127 // GK // glycerol kinase // Xp21.3 // 2710 /// NM_000167 // GK // glycerol	1.44E-07	-0.3907
8011245	NM_018128	TSR1	NM_018128 // TSR1 // TSR1, 20S rRNA accumulation, homolog (S. cerevisiae) // 17p13.3 //	1.10E-05	-0.32659
8036444	NM_152360	ZNF573	NM_152360 // ZNF573 // zinc finger protein 573 // 19q13.12 // 126231 /// NM_001172689 /	0.00697896	0.208977
8007427	NM_003734	AOC3	NM_003734 // AOC3 // amine oxidase, copper containing 3 (vascular adhesion protein 1) /	0.00473769	0.21863
8128322	NM_014165	NDUFAF4	NM_014165 // NDUFAF4 // NADH dehydrogenase (ubiquinone) 1 alpha subcomplex, assembly fa	0.00147444	-0.248331
8144388	NM_001164453	FAM90A20	NM_001164453 // FAM90A20 // family with sequence similarity 90, member A20 // 8p23.1 //	0.00118739	0.245959
7928630	NM_001099692	EIF5AL1	NM_001099692 // EIF5AL1 // eukaryotic translation initiation factor 5A-like 1 // 10q22.	1.31E-06	-0.360276
8020025	---		---	0.00233714	0.230848
7976842	NR_029874	MIR382	NR_029874 // MIR382 // microRNA 382 // 14q32.31 // 494331	0.00324501	0.223755
8101957	NM_016242	EMCN	NM_016242 // EMCN // endomucin // 4q24 // 51705 /// NM_001159694 // EMCN // endomucin /	0.0041114	0.220775
8171449	NM_021804	ACE2	NM_021804 // ACE2 // angiotensin I converting enzyme (peptidyl-dipeptidase A) 2 // Xp22	5.42E-06	-0.345674
8169504	NM_007231	SLC6A14	NM_007231 // SLC6A14 // solute carrier family 6 (amino acid transporter), member 14 //	5.52E-05	-0.309765
8070141	NM_145858	CRYZL1	NM_145858 // CRYZL1 // crystallin, zeta (quinone reductase)-like 1 // 21q21.3 // 9946 /	0.00230482	0.231051

8081740	NM_001690	ATP6V1A	NM_001690 // ATP6V1A // ATPase, H+ transporting, lysosomal 70kDa, V1 subunit A // 3q13.	0.00157642	-0.233128
7979412	---		---	0.000450366	0.26527
8152828	NM_031415	GSDMC	NM_031415 // GSDMC // gasdermin C // 8q24.21 // 56169 /// ENST00000276708 // GSDMC // g	0.000139175	-0.287062
8147891	NM_177531	PKHD1L1	NM_177531 // PKHD1L1 // polycystic kidney and hepatic disease 1 (autosomal recessive)-l	0.00231176	0.236066
8112558	NR_003504	GUSBL1	NR_003504 // GUSBL1 // glucuronidase, beta-like 1 // 6p21 // 387036 /// AK289851 // SMA	6.20E-09	0.421152
8116272	NM_014244	ADAMTS2	NM_014244 // ADAMTS2 // ADAM metallopeptidase with thrombospondin type 1 motif, 2 // 5q	0.0031106	-0.225255
7923501	---		---	0.00415992	0.221285
7934278	NM_000917	P4HA1	NM_000917 // P4HA1 // prolyl 4-hydroxylase, alpha polypeptide I // 10q21.3-q23.1 // 503	0.0026906	-0.224941
8053315	NM_024993	LRRTM4	NM_024993 // LRRTM4 // leucine rich repeat transmembrane neuronal 4 // 2p12 // 80059 //	0.0032932	0.221802
8137240	NM_153236	GIMAP7	NM_153236 // GIMAP7 // GTPase, IMAP family member 7 // 7q36.1 // 168537 /// ENST0000031	0.000192238	0.283303
8062119	BC103840	MT1P3	BC103840 // MT1P3 // metallothionein 1 pseudogene 3 // 20q11.2 // 140851 /// BC103840 /	0.00050139	-0.268202
8152902	NM_001115	ADCY8	NM_001115 // ADCY8 // adenylate cyclase 8 (brain) // 8q24 // 114 /// ENST00000286355 //	0.00044755	0.27127
7983527	NM_153618	SEMA6D	NM_153618 // SEMA6D // sema domain, transmembrane domain (TM), and cytoplasmic domain,	4.77E-05	0.305961
8021653	NM_002640	SERPINB8	NM_002640 // SERPINB8 // serpin peptidase inhibitor, clade B (ovalbumin), member 8 // 1	0.000938355	-0.252223
8097647	NM_002940	ABCE1	NM_002940 // ABCE1 // ATP-binding cassette, sub-family E (OABP), member 1 // 4q31 // 60	0.000289554	-0.270531
8140454	NM_175064	SPDYE1	NM_175064 // SPDYE1 // speedy homolog E1 (Xenopus laevis) // 7p13 // 285955 /// NR_0036	2.84E-05	0.311229

7981895	NR_027407	GOLGA8DP	NR_027407 // GOLGA8DP // golgin A8 family, member D (pseudogene) // 15q11.2 // 10013297	0.0001116	0.283625
8045804	---		---	0.000364057	0.273352
8047709	NM_152526	PARD3B	NM_152526 // PARD3B // par-3 partitioning defective 3 homolog B (C. elegans) // 2q33.3	0.00421548	0.219127
7978838	NM_018139	C14orf104	NM_018139 // C14orf104 // chromosome 14 open reading frame 104 // 14q21.3 // 55172 ///	0.00651638	-0.208481
8054870	---		---	0.00705489	0.210765
7952056	NM_000732	CD3D	NM_000732 // CD3D // CD3d molecule, delta (CD3-TCR complex) // 11q23 // 915 /// NM_0010	0.00192273	0.239788
7979548	NM_145171	GPHB5	NM_145171 // GPHB5 // glycoprotein hormone beta 5 // 14q23.2 // 122876 /// ENST00000314	0.000425852	0.269609
8099967	NM_001098634	RBM47	NM_001098634 // RBM47 // RNA binding motif protein 47 // 4p14 // 54502 /// NM_019027 //	8.65E-05	-0.300364
8172280	NM_032591	SLC9A7	NM_032591 // SLC9A7 // solute carrier family 9 (sodium/hydrogen exchanger), member 7 //	0.00915517	-0.198863
7898407	BC036435	LOC100132147	BC036435 // LOC100132147 // hypothetical LOC100132147 // 1p36.13 // 100132147 /// AK125	0.000972278	0.250149
8042335	NM_003375	VDAC2	NM_003375 // VDAC2 // voltage-dependent anion channel 2 // 10q22 // 7417 /// ENST000003	0.00352066	-0.228085
8144424	NM_001164456	FAM90A13	NM_001164456 // FAM90A13 // family with sequence similarity 90, member A13 // 8p23.1 //	0.00041899	0.267726
8144440	NM_001164451	FAM90A18	NM_001164451 // FAM90A18 // family with sequence similarity 90, member A18 // 8p23.1 //	0.00041899	0.267726
8146703	NM_152765	C8orf46	NM_152765 // C8orf46 // chromosome 8 open reading frame 46 // 8q13.1 // 254778 /// ENST	0.00576063	0.213634
7949882	NM_000695	ALDH3B2	NM_000695 // ALDH3B2 // aldehyde dehydrogenase 3 family, member B2 // 11q13 // 222 ///	0.000183165	-0.283608
8121277	NM_001624	AIM1	NM_001624 // AIM1 // absent in melanoma 1 // 6q21 // 202 /// ENST00000369066 // AIM1 //	4.10E-05	-0.30936

8088560	NM_182920	ADAMTS9	NM_182920 // ADAMTS9 // ADAM metallopeptidase with thrombospondin type 1 motif, 9 // 3p	1.82E-05	-0.315363
7946275	NR_003945	GVINP1	NR_003945 // GVINP1 // GTPase, very large interferon inducible pseudogene 1 // 11p15.4	0.00136017	0.245066
8003298	NM_003486	SLC7A5	NM_003486 // SLC7A5 // solute carrier family 7 (cationic amino acid transporter, y+ sys	1.06E-07	-0.392178
7950370	---		---	0.00618463	0.204362
8000482	NM_015171	XPO6	NM_015171 // XPO6 // exportin 6 // 16p11.2 // 23214 /// ENST00000304658 // XPO6 // expo	0.00035093	-0.275566
8161415	ENST0000045698	LOC644249	ENST00000456982 // LOC644249 // hypothetical LOC644249 // 9q12 // 644249 /// ENST000004	0.00919727	0.199439
7953715	NM_015509	NECAP1	NM_015509 // NECAP1 // NECAP endocytosis associated 1 // 12p13.31 // 25977 /// NR_02426	0.000889264	-0.250153
8019796	NM_002359	MAFG	NM_002359 // MAFG // v-maf musculoaponeurotic fibrosarcoma oncogene homolog G (avian) /	0.00548221	-0.217635
8095736	NM_001657	AREG	NM_001657 // AREG // amphiregulin // 4q13-q21 // 374 /// BC009799 // AREG // amphiregul	0.000111308	-0.29413
7973871	---		---	0.00016367	0.288525
8165696	---		---	0.00016367	0.288525
7911337	---		---	0.00016367	0.288525
8080419	NM_206825	GNL3	NM_206825 // GNL3 // guanine nucleotide binding protein-like 3 (nucleolar) // 3p21.1 //	9.75E-07	-0.35801
8161375	---		---	0.000367478	0.269311
8155453	---		---	0.000367478	0.269311
8167526	NM_001098406	GAGE12J	NM_001098406 // GAGE12J // G antigen 12J // Xp11.23 // 729396 /// NM_001476 // GAGE6 //	0.00095128	0.247797
7934384	NM_001024593	ZMYND17	NM_001024593 // ZMYND17 // zinc finger, MYND-type containing 17 // 10q22.2 // 118490 //	3.83E-05	0.304731
7935910	NM_012215	MGEA5	NM_012215 // MGEA5 // meningioma expressed antigen 5 (hyaluronidase) // 10q24.1-q24.3 /	7.37E-05	0.300564

8101609	NM_197965	SLC10A6	NM_197965 // SLC10A6 // solute carrier family 10 (sodium/bile acid cotransporter family	0.00686266	-0.20737
7899615	NM_178865	SERINC2	NM_178865 // SERINC2 // serine incorporator 2 // 1p35.1 // 347735 /// ENST00000373709 /	0.000720621	-0.257148
8069676	NM_006988	ADAMTS1	NM_006988 // ADAMTS1 // ADAM metallopeptidase with thrombospondin type 1 motif, 1 // 21	0.000252968	-0.27794
8163107	NR_029506	MIR32	NR_029506 // MIR32 // microRNA 32 // 9q31.3 // 407036	0.000405763	0.263541
7912547	NM_001146344	PRAMEF11	NM_001146344 // PRAMEF11 // PRAME family member 11 // 1p36.21 // 440560 /// NM_00109837	0.00148324	0.242935
8142061	NM_019042	PUS7	NM_019042 // PUS7 // pseudouridylate synthase 7 homolog (S. cerevisiae) // 7q22.3 // 54	0.00273641	-0.229091
7927231	NM_133446	AGAP4	NM_133446 // AGAP4 // ArfGAP with GTPase domain, ankyrin repeat and PH domain 4 // 10q1	0.000111712	0.287011
8138381	NM_006408	AGR2	NM_006408 // AGR2 // anterior gradient homolog 2 (Xenopus laevis) // 7p21.3 // 10551 //	0.0049241	-0.216251
8031981	NR_003659	WASH3P	NR_003659 // WASH3P // WAS protein family homolog 3 pseudogene // 15q26.3 // 374666 ///	0.00105651	0.246493
7921076	NM_182679	GPATCH4	NM_182679 // GPATCH4 // G patch domain containing 4 // 1q22 // 54865 /// NM_015590 // G	1.67E-05	-0.323311
7928600	NM_001099692	EIF5AL1	NM_001099692 // EIF5AL1 // eukaryotic translation initiation factor 5A-like 1 // 10q22.	1.03E-06	-0.363824
8139456	NR_002952	SNORA9	NR_002952 // SNORA9 // small nucleolar RNA, H/ACA box 9 // 7p13 // 677798 /// AK096179	0.0059172	-0.213824
8142136	NM_175884	FLJ36031	NM_175884 // FLJ36031 // hypothetical protein FLJ36031 // 7q22.3 // 168455	7.37E-05	-0.293767

8040742	NM_012326	MAPRE3	NM_012326 // MAPRE3 // microtubule-associated protein, RP/EB family, member 3 // 2p23.3	0.00993779	-0.198858
7938390	NM_001124	ADM	NM_001124 // ADM // adrenomedullin // 11p15.4 // 133 /// ENST00000278175 // ADM // adre	0.002322	-0.233836
7909236	NM_004759	MAPKAPK2	NM_004759 // MAPKAPK2 // mitogen-activated protein kinase-activated protein kinase 2 //	0.000132167	-0.293577
8144428	NM_001164456	FAM90A13	NM_001164456 // FAM90A13 // family with sequence similarity 90, member A13 // 8p23.1 //	0.0004704	0.265397
8144432	NM_001164450	FAM90A8	NM_001164450 // FAM90A8 // family with sequence similarity 90, member A8 // 8p23.1 // 4	0.0004704	0.265397
8144436	NM_001164456	FAM90A13	NM_001164456 // FAM90A13 // family with sequence similarity 90, member A13 // 8p23.1 //	0.0004704	0.265397
8144444	NM_001164450	FAM90A8	NM_001164450 // FAM90A8 // family with sequence similarity 90, member A8 // 8p23.1 // 4	0.0004704	0.265397
8097148	NM_015312	KIAA1109	NM_015312 // KIAA1109 // KIAA1109 // 4q27 // 84162 /// ENST00000264501 // KIAA1109 // K	0.00556682	0.213404
8004521	NM_004870	MPDU1	NM_004870 // MPDU1 // mannose-P-dolichol utilization defect 1 // 17p13.1-p12 // 9526 //	0.000155277	-0.285409
8149204	NM_001136572	FAM90A7	NM_001136572 // FAM90A7 // family with sequence similarity 90, member A7 // 8p23.1 // 4	0.000292359	0.274683
7909601	NR_004389	SNORA16B	NR_004389 // SNORA16B // small nucleolar RNA, H/ACA box 16B // 1q32.3 // 692157	0.00274936	0.225293
8008588	NM_002126	HLF	NM_002126 // HLF // hepatic leukemia factor // 17q22 // 3131 /// ENST00000226067 // HLF	0.00111155	0.248459
8003249	NM_024735	FBXO31	NM_024735 // FBXO31 // F-box protein 31 // 16q24.2 // 79791 /// NR_024568 // FBXO31 //	0.000949515	-0.252335

8088979	NM_016206	VGLL3	NM_016206 // VGLL3 // vestigial like 3 (Drosophila) // 3p12.1 // 389136 /// ENST0000039	0.00265889	0.232403
7899737	NM_003757	EIF3I	NM_003757 // EIF3I // eukaryotic translation initiation factor 3, subunit I // 1p34.1 /	0.000264066	-0.281358
7934753	NM_001099692	EIF5AL1	NM_001099692 // EIF5AL1 // eukaryotic translation initiation factor 5A-like 1 // 10q22.	1.22E-06	-0.360734
8149148	AK122996	OR7E154P	AK122996 // OR7E154P // olfactory receptor, family 7, subfamily E, member 154 pseudogen	0.010214	0.193487
7951447	NM_152434	CWF19L2	NM_152434 // CWF19L2 // CWF19-like 2, cell cycle control (S. pombe) // 11q22.3 // 14388	0.00493201	0.217116
8076690	NM_001009880	C22orf9	NM_001009880 // C22orf9 // chromosome 22 open reading frame 9 // 22q13.31 // 23313 ///	0.00144275	-0.240198
8007134	NM_033191	KRTAP9-4	NM_033191 // KRTAP9-4 // keratin associated protein 9-4 // 17q12-q21 // 85280 /// NM_03	0.00442593	0.216525
7963534	NM_002272	KRT4	NM_002272 // KRT4 // keratin 4 // 12q12-q13 // 3851 /// ENST00000293774 // KRT4 // kera	0.00246299	0.228105
7914516	NR_026850	MTMR9LP	NR_026850 // MTMR9LP // myotubularin related protein 9-like, pseudogene // 1p35.1 // 33	0.000191726	0.273893
8167584	NM_001098409	GAGE12G	NM_001098409 // GAGE12G // G antigen 12G // Xp11.23 // 645073 /// NM_001098408 // GAGE1	0.00100514	0.245899
8141374	NM_001185	AZGP1	NM_001185 // AZGP1 // alpha-2-glycoprotein 1, zinc-binding // 7q22.1 // 563 /// ENST000	0.00267686	-0.235371
8128316	NM_001143957	GPR63	NM_001143957 // GPR63 // G protein-coupled receptor 63 // 6q16.1-q16.3 // 81491 /// NM_	0.0026744	0.230213
8043995	NM_000877	IL1R1	NM_000877 // IL1R1 // interleukin 1 receptor, type I // 2q12 // 3554 /// ENST0000023394	1.10E-06	-0.356271
8063923	NM_016354	SLCO4A1	NM_016354 // SLCO4A1 // solute carrier organic anion transporter family, member 4A1 //	3.68E-06	-0.35739
8071642	ENST0000039028!	IGLV6-57	ENST00000390285 // IGLV6-57 // immunoglobulin lambda variable 6-57 // 22q11.2 // 28778	0.00538982	-0.21535

7906475	NM_001004310	FCRL6	NM_001004310 // FCRL6 // Fc receptor-like 6 // 1q23.2 // 343413 /// ENST00000368106 //	7.50E-05	0.303228
7955906	NR_029894	MIR148B	NR_029894 // MIR148B // microRNA 148b // 12q13.13 // 442892	0.00344585	0.225293
8117079	BC047037	LOC644714	BC047037 // LOC644714 // hypothetical LOC644714 // 3p21.31 // 644714	0.00312523	0.226945
7974255	---		---	0.00686531	0.203751
7975457	---		---	0.00716368	0.203386
8116534	NM_032765	TRIM52	NM_032765 // TRIM52 // tripartite motif-containing 52 // 5q35.3 // 84851 /// ENST000003	0.000771522	0.255999
7956826	NM_015279	TBC1D30	NM_015279 // TBC1D30 // TBC1 domain family, member 30 // 12q14.3 // 23329 /// AB449914	1.94E-05	-0.323289
8161451	AK292642	LOC554249	AK292642 // LOC554249 // hypothetical LOC554249 // 9q12 // 554249 /// ENST00000377616 /	0.00210937	0.231103
8155393	AK292642	LOC554249	AK292642 // LOC554249 // hypothetical LOC554249 // 9q12 // 554249 /// ENST00000377616 /	0.00210931	0.231104
8043993	AY358263	LOC100131131	AY358263 // LOC100131131 // AHPA9419 // 2q12.1 // 100131131	0.00205814	-0.231875
7960575	NM_001033714	NOP2	NM_001033714 // NOP2 // NOP2 nucleolar protein homolog (yeast) // 12p13 // 4839 /// NM_	8.89E-06	-0.331491
8082406	---		---	0.00362635	0.229261
8128123	NM_021244	RRAGD	NM_021244 // RRAGD // Ras-related GTP binding D // 6q15-q16 // 58528 /// ENST0000036941	0.00568917	-0.203676
8069811	NM_181624	KRTAP23-1	NM_181624 // KRTAP23-1 // keratin associated protein 23-1 // 21q22.1 // 337963 /// ENST	0.00286427	0.229811
8132531	NM_175064	SPDYE1	NM_175064 // SPDYE1 // speedy homolog E1 (Xenopus laevis) // 7p13 // 285955 /// NM_0010	0.000463165	0.263135
7950578	NM_001128620	PAK1	NM_001128620 // PAK1 // p21 protein (Cdc42/Rac)-activated kinase 1 // 11q13-q14 // 5058	0.00719604	-0.205591

8095744	NM_001657	AREG	NM_001657 // AREG // amphiregulin // 4q13-q21 // 374 /// BC009799 // AREG // amphiregul	0.000100792	-0.295737
8171723	---		---	0.000398872	0.268753
8106720	---		---	0.000275056	0.265303
7963313	NM_007210	GALNT6	NM_007210 // GALNT6 // UDP-N-acetyl-alpha-D-galactosamine:polypeptide N-acetylgalactosa	0.00917098	-0.20257
7898371	ENST0000036917:	LOC644634	ENST00000369173 // LOC644634 // UPF0627 protein ENSP00000358171-like // 1q21.2 // 64463	0.00497533	0.214692
7912861	ENST0000036917:	LOC644634	ENST00000369173 // LOC644634 // UPF0627 protein ENSP00000358171-like // 1q21.2 // 64463	0.00497533	0.214692
8062603	NM_003286	TOP1	NM_003286 // TOP1 // topoisomerase (DNA) I // 20q12-q13.1 // 7150 /// ENST00000361337 /	6.80E-06	-0.334995
7907430	NM_000639	FASLG	NM_000639 // FASLG // Fas ligand (TNF superfamily, member 6) // 1q23 // 356 /// ENST000	0.00762173	0.206629
7925823	NM_012341	GTPBP4	NM_012341 // GTPBP4 // GTP binding protein 4 // 10p15-p14 // 23560 /// ENST00000360803	3.78E-06	-0.340891
8127364	NR_003660	GUSBP4	NR_003660 // GUSBP4 // glucuronidase, beta pseudogene 4 // 6p11.2 // 375513 /// BC06554	0.000202987	0.271541
8136641	NM_016943	TAS2R3	NM_016943 // TAS2R3 // taste receptor, type 2, member 3 // 7q31.3-q32 // 50831 /// ENST	0.000209267	0.27868
8032909	NM_005817	PLIN3	NM_005817 // PLIN3 // perilipin 3 // 19p13.3 // 10226 /// NM_001164189 // PLIN3 // peri	4.53E-05	-0.30781
8065762	NM_080476	PIGU	NM_080476 // PIGU // phosphatidylinositol glycan anchor biosynthesis, class U // 20q11.	0.00722121	-0.205368
8068651	NM_006198	PCP4	NM_006198 // PCP4 // Purkinje cell protein 4 // 21q22.2 // 5121 /// ENST00000328619 //	0.00255778	-0.22839
7907492	NM_014857	RABGAP1L	NM_014857 // RABGAP1L // RAB GTPase activating protein 1-like // 1q24 // 9910 /// NM_00	0.001454	0.244927
8079229	NM_145044	ZNF501	NM_145044 // ZNF501 // zinc finger protein 501 // 3p21.31 // 115560 /// ENST00000396048	0.000131081	0.29433

8161747	NM_001102420	ZFAND5	NM_001102420 // ZFAND5 // zinc finger, AN1-type domain 5 // 9q13-q21 // 7763 /// NM_001	0.00953147	-0.203045
7943998	NM_006169	NNMT	NM_006169 // NNMT // nicotinamide N-methyltransferase // 11q23.1 // 4837 /// ENST000002	7.81E-07	-0.360768
8167577	NM_001098409	GAGE12G	NM_001098409 // GAGE12G // G antigen 12G // Xp11.23 // 645073 /// NM_001098408 // GAGE1	0.000877548	0.249489
8152606	NM_021021	SNTB1	NM_021021 // SNTB1 // syntrophin, beta 1 (dystrophin-associated protein A1, 59kDa, basi	0.00238958	-0.228793
7901314	---		---	0.000691155	0.259352
8116418	NM_005110	GFPT2	NM_005110 // GFPT2 // glutamine-fructose-6-phosphate transaminase 2 // 5q34-q35 // 9945	0.000176631	-0.287119
7939056	NM_003986	BBOX1	NM_003986 // BBOX1 // butyrobetaine (gamma), 2-oxoglutarate dioxygenase (gamma-butyrobe	0.000244545	-0.277859
8173208	NM_001012968	SPIN4	NM_001012968 // SPIN4 // spindlin family, member 4 // Xq11.1 // 139886 /// ENST00000335	0.00940155	0.204107
7960794	NM_004244	CD163	NM_004244 // CD163 // CD163 molecule // 12p13.3 // 9332 /// NM_203416 // CD163 // CD163	0.00121428	-0.248158
8163729	NR_029604	MIR147	NR_029604 // MIR147 // microRNA 147 // 9q33.2 // 406939	0.000604054	0.257552
8058664	---		---	0.000224095	0.280116
8155268	NM_022490	POLR1E	NM_022490 // POLR1E // polymerase (RNA) I polypeptide E, 53kDa // 9p13.2 // 64425 /// E	0.000200481	-0.288706
7899753	NM_005356	LCK	NM_005356 // LCK // lymphocyte-specific protein tyrosine kinase // 1p34.3 // 3932 /// N	0.00300661	0.229107
8119492	NM_004053	BYSL	NM_004053 // BYSL // bystin-like // 6p21.1 // 705 /// ENST00000230340 // BYSL // bystin	3.43E-06	-0.352035
7915563	---		---	0.00118456	-0.253333
7953878	NM_001004419	CLEC2D	NM_001004419 // CLEC2D // C-type lectin domain family 2, member D // 12p13 // 29121 ///	0.00105407	0.253636

8128429	NM_005190	CCNC	NM_005190 // CCNC // cyclin C // 6q21 // 892 /// NM_001013399 // CCNC // cyclin C // 6q	0.0010256	-0.249695
8152946	NM_004519	KCNQ3	NM_004519 // KCNQ3 // potassium voltage-gated channel, KQT-like subfamily, member 3 //	0.000343357	-0.276038
7956737	NR_029661	MIRLET7I	NR_029661 // MIRLET7I // microRNA let-7i // 12q14.1 // 406891	0.00199282	0.232922
8127502	NR_026807	C6orf155	NR_026807 // C6orf155 // chromosome 6 open reading frame 155 // 6q13 // 79940	2.46E-06	0.350783
8063497	NM_001164116	CASS4	NM_001164116 // CASS4 // Cas scaffolding protein family member 4 // 20q13.31 // 57091 /	0.00527426	0.217705
7952036	NM_198275	MPZL3	NM_198275 // MPZL3 // myelin protein zero-like 3 // 11q23.3 // 196264 /// ENST000002789	0.0001202	-0.294695
7903407	NM_020978	AMY2B	NM_020978 // AMY2B // amylase, alpha 2B (pancreatic) // 1p21 // 280 /// NM_017619 // RN	0.00122255	0.244699
7899436	NM_031459	SESN2	NM_031459 // SESN2 // sestrin 2 // 1p35.3 // 83667 /// ENST00000253063 // SESN2 // sest	0.000113789	-0.291162
8131583	NM_001159767	BZW2	NM_001159767 // BZW2 // basic leucine zipper and W2 domains 2 // 7p21.1 // 28969 /// NM	1.41E-05	-0.324107
8166948	---		---	0.00803705	0.201971
7925531	NM_181690	AKT3	NM_181690 // AKT3 // v-akt murine thymoma viral oncogene homolog 3 (protein kinase B, g	0.00137715	0.246131
8108905	NM_020768	KCTD16	NM_020768 // KCTD16 // potassium channel tetramerisation domain containing 16 // 5q31.3	0.000111689	0.291734
8087881	NR_029660	MIRLET7G	NR_029660 // MIRLET7G // microRNA let-7g // 3p21.1 // 406890	0.000409537	0.267907
8047518	NM_015934	NOP58	NM_015934 // NOP58 // NOP58 ribonucleoprotein homolog (yeast) // 2q33.1 // 51602 /// EN	0.0035929	-0.219405
7928534	NM_032024	C10orf11	NM_032024 // C10orf11 // chromosome 10 open reading frame 11 // 10q22.3 // 83938 /// EN	0.00507409	0.216175
8144448	NM_001164447	FAM90A10	NM_001164447 // FAM90A10 // family with sequence similarity 90, member A10 // 8p23.1 //	0.000334609	0.272275

7995787	NM_176870	MT1M	NM_176870 // MT1M // metallothionein 1M // 16q13 // 4499 /// ENST00000379818 // MT1M //	5.29E-05	-0.313676
8089743	NM_212543	B4GALT4	NM_212543 // B4GALT4 // UDP-Gal:betaGlcNAc beta 1,4- galactosyltransferase, polypeptide	0.00279039	-0.228084
8151629	---		---	9.40E-05	0.301341
8058869	NM_022648	TNS1	NM_022648 // TNS1 // tensin 1 // 2q35-q36 // 7145 /// ENST00000171887 // TNS1 // tensin	0.0025082	0.229134
8148435	NM_003882	WISP1	NM_003882 // WISP1 // WNT1 inducible signaling pathway protein 1 // 8q24.22 // 8840 ///	1.63E-05	-0.319616
8122265	NM_006290	TNFAIP3	NM_006290 // TNFAIP3 // tumor necrosis factor, alpha-induced protein 3 // 6q23 // 7128	7.54E-06	-0.342921
8105995	NR_024054	LOC100170939	NR_024054 // LOC100170939 // glucuronidase, beta pseudogene // 5q13 // 100170939 /// BC	1.11E-08	0.415956
8098870	NM_012445	SPON2	NM_012445 // SPON2 // spondin 2, extracellular matrix protein // 4p16.3 // 10417 /// NM	0.00476796	-0.209282
8177544	NR_034021	SMA5	NR_034021 // SMA5 // glucuronidase, beta pseudogene // 5q13 // 11042 /// NR_027386 // G	2.58E-07	0.376572
8047217	NM_025147	COQ10B	NM_025147 // COQ10B // coenzyme Q10 homolog B (S. cerevisiae) // 2q33.1 // 80219 /// EN	7.12E-06	-0.330339
8103975	NR_003542	SLED1	NR_003542 // SLED1 // proteoglycan 3 pseudogene // 4q35.1 // 643036 /// AY358224 // SLE	0.00123568	-0.252946
8028756	NM_006503	PSMC4	NM_006503 // PSMC4 // proteasome (prosome, macropain) 26S subunit, ATPase, 4 // 19q13.1	0.000494426	-0.272471
7939120	NM_002901	RCN1	NM_002901 // RCN1 // reticulocalbin 1, EF-hand calcium binding domain // 11p13 // 5954	0.000491161	-0.26719
8094134	NR_027279	USP17L6P	NR_027279 // USP17L6P // ubiquitin specific peptidase 17-like 6 (pseudogene) // 4p16.1	5.73E-05	0.30526
8142120	NM_005746	NAMPT	NM_005746 // NAMPT // nicotinamide phosphoribosyltransferase // 7q22.3 // 10135 /// ENS	6.45E-09	-0.428498

8124440	NM_003534	HIST1H3G	NM_003534 // HIST1H3G // histone cluster 1, H3g // 6p21.3 // 8355	0.00193569	-0.241506
8096704	NM_001184690	NPNT	NM_001184690 // NPNT // nephronectin // 4q24 // 255743 /// NM_001033047 // NPNT // neph	0.00421755	0.220531
8088700	NM_007114	TMF1	NM_007114 // TMF1 // TATA element modulatory factor 1 // 3p21-p12 // 7110 /// ENST00000	0.002779	-0.227483
8045088	---		---	0.000128349	0.29304
8077877	AK094424	LOC100130924	AK094424 // LOC100130924 // hypothetical LOC100130924 // 3p25.2 // 100130924	0.00994368	0.201604
7953590	NR_029779	MIR200C	NR_029779 // MIR200C // microRNA 200c // 12p13.31 // 406985	3.48E-05	0.305744
8143781	NM_024711	GIMAP6	NM_024711 // GIMAP6 // GTPase, IMAP family member 6 // --- // 474344 /// NR_024115 // G	9.88E-06	0.331448
8152333	---		---	0.00769136	0.209913
8037298	NM_020406	CD177	NM_020406 // CD177 // CD177 molecule // 19q13.2 // 57126 /// ENST00000457794 // CD177 /	0.00932944	-0.201705
7995797	NM_175617	MT1E	NM_175617 // MT1E // metallothionein 1E // 16q13 // 4493 /// BC009699 // MT1E // metall	0.00308169	-0.231798
8123884	NR_026737	C6orf52	NR_026737 // C6orf52 // chromosome 6 open reading frame 52 // 6p24.1 // 347744 /// NR_0	0.00531003	-0.216215
8160968	NM_013442	STOML2	NM_013442 // STOML2 // stomatin (EPB72)-like 2 // 9p13.1 // 30968 /// ENST00000356493 /	0.000128263	-0.2968
8147206	NM_003821	RIPK2	NM_003821 // RIPK2 // receptor-interacting serine-threonine kinase 2 // 8q21 // 8767 //	3.14E-06	-0.347813
8069541	NM_022136	SAMSN1	NM_022136 // SAMSN1 // SAM domain, SH3 domain and nuclear localization signals 1 // 21q	2.24E-06	-0.354092
8126058	NM_016059	PPIL1	NM_016059 // PPIL1 // peptidylprolyl isomerase (cyclophilin)-like 1 // 6p21.1 // 51645	1.71E-05	-0.324065
7995525	NM_033119	NKD1	NM_033119 // NKD1 // naked cuticle homolog 1 (Drosophila) // 16q12 // 85407 /// ENST000	0.000808143	0.258865
8127500	NR_029504	MIR30A	NR_029504 // MIR30A // microRNA 30a // 6q13 // 407029 /// AF480569 // MIR30A // microRN	0.0032476	0.226276

7986428	AK127420	LOC400464	AK127420 // LOC400464 // hypothetical LOC400464 // 15q26.3 // 400464	0.000344389	0.26426
8101893	NM_000669	ADH1C	NM_000669 // ADH1C // alcohol dehydrogenase 1C (class I), gamma polypeptide // 4q23 //	0.00923198	0.20257
7981872	---		---	0.00549726	0.207416
8159959	---		---	0.00342567	0.226604
8125887	NM_003214	TEAD3	NM_003214 // TEAD3 // TEA domain family member 3 // 6p21.2 // 7005 /// ENST00000506863	0.00454539	-0.212992
8081853	NM_152305	POGLUT1	NM_152305 // POGLUT1 // protein O-glucosyltransferase 1 // 3q13.33 // 56983 /// NR_0242	0.00809988	-0.204432
8046099	NM_001039724	NOSTRIN	NM_001039724 // NOSTRIN // nitric oxide synthase trafficker // 2q31.1 // 115677 /// NM_	4.56E-05	0.30979
8047161	NM_001031716	OBFC2A	NM_001031716 // OBFC2A // oligonucleotide/oligosaccharide-binding fold containing 2A //	0.00470859	-0.21655
8065412	NM_001898	CST1	NM_001898 // CST1 // cystatin SN // 20p11.21 // 1469 /// ENST00000304749 // CST1 // cys	0.0027091	-0.231651
8167562	NM_001127345	GAGE12B	NM_001127345 // GAGE12B // G antigen 12B // Xp11.23 // 729428 /// NM_001098409 // GAGE1	0.00088693	0.248769
8141843	NM_006989	RASA4	NM_006989 // RASA4 // RAS p21 protein activator 4 // 7q22 // 10156 /// NM_001079877 //	0.00136151	0.243343
8144397	NM_201402	USP17L2	NM_201402 // USP17L2 // ubiquitin specific peptidase 17-like 2 // 8p23.1 // 377630 ///	2.15E-05	0.317366
8149241	NM_201402	USP17L2	NM_201402 // USP17L2 // ubiquitin specific peptidase 17-like 2 // 8p23.1 // 377630 ///	2.15E-05	0.317366
8094122	NR_027279	USP17L6P	NR_027279 // USP17L6P // ubiquitin specific peptidase 17-like 6 (pseudogene) // 4p16.1	0.0001034	0.293381
8095688	NM_002993	CXCL6	NM_002993 // CXCL6 // chemokine (C-X-C motif) ligand 6 (granulocyte chemotactic protein	0.00110227	-0.249582

8074925	NR_024448	LOC91316	NR_024448 // LOC91316 // glucuronidase, beta/immunoglobulin lambda-like polypeptide 1 p	5.83E-09	0.421839
7943413	NM_001165	BIRC3	NM_001165 // BIRC3 // baculoviral IAP repeat-containing 3 // 11q22 // 330 /// NM_182962	0.000449704	-0.261871
7995352	---		---	0.00998328	0.198472
8113344	---		---	0.00260295	0.226664
8114207	---		---	0.00260295	0.226664
8103483	---		---	0.00260295	0.226664
7933976	---		---	0.00122884	0.244087
7912374	NM_003132	SRM	NM_003132 // SRM // spermidine synthase // 1p36-p22 // 6723 /// ENST00000376957 // SRM	0.000663467	-0.25846
7905507	NM_178428	LCE2A	NM_178428 // LCE2A // late cornified envelope 2A // 1q21.3 // 353139 /// ENST0000036877	0.00620094	0.210156
7987139	NR_024074	GOLGA8IP	NR_024074 // GOLGA8IP // golgin A8 family, member I (pseudogene) // 15q11.2 // 283796 /	0.00123075	0.244032
7982350	NR_024074	GOLGA8IP	NR_024074 // GOLGA8IP // golgin A8 family, member I (pseudogene) // 15q11.2 // 283796 /	0.00123075	0.244032
8052872	NM_003236	TGFA	NM_003236 // TGFA // transforming growth factor, alpha // 2p13 // 7039 /// NM_001099691	9.11E-06	-0.33402
8150087	---		---	0.00531298	0.21265
8138728	NM_002141	HOXA4	NM_002141 // HOXA4 // homeobox A4 // 7p15.2 // 3201 /// ENST00000360046 // HOXA4 // hom	4.54E-06	0.339654
8027996	NM_172341	PSENEN	NM_172341 // PSENEN // presenilin enhancer 2 homolog (C. elegans) // 19q13.12 // 55851	0.003052	-0.228719
8083594	NM_002852	PTX3	NM_002852 // PTX3 // pentraxin 3, long // 3q25 // 5806 /// ENST00000295927 // PTX3 // p	1.59E-08	-0.411638
8159854	NM_014878	KIAA0020	NM_014878 // KIAA0020 // KIAA0020 // 9p24.2 // 9933 /// ENST00000397885 // KIAA0020 //	0.00514097	-0.21397
8085660	NM_206831	DPH3	NM_206831 // DPH3 // DPH3, KTI11 homolog (S. cerevisiae) // 3p25.1 // 285381 /// NM_001	0.0004993	-0.268248
7918533	NM_020683	ADORA3	NM_020683 // ADORA3 // adenosine A3 receptor // 1p13.2 // 140 /// NM_001081976 // ADORA	0.00247245	-0.231834

8094116	NM_001105662	USP17	NM_001105662 // USP17 // ubiquitin specific peptidase 17 // 4p15 // 391627 /// NR_02727	0.00011277	0.292356
8094118	NM_001105662	USP17	NM_001105662 // USP17 // ubiquitin specific peptidase 17 // 4p15 // 391627 /// NR_02727	0.00011277	0.292356
8094120	NM_001105662	USP17	NM_001105662 // USP17 // ubiquitin specific peptidase 17 // 4p15 // 391627 /// NR_02727	0.00011277	0.292356
8094124	NM_001105662	USP17	NM_001105662 // USP17 // ubiquitin specific peptidase 17 // 4p15 // 391627 /// NR_02727	0.00011277	0.292356
8094126	NM_001105662	USP17	NM_001105662 // USP17 // ubiquitin specific peptidase 17 // 4p15 // 391627 /// NR_02727	0.00011277	0.292356
8094128	NM_001105662	USP17	NM_001105662 // USP17 // ubiquitin specific peptidase 17 // 4p15 // 391627 /// NR_02727	0.00011277	0.292356
8094132	NM_001105662	USP17	NM_001105662 // USP17 // ubiquitin specific peptidase 17 // 4p15 // 391627 /// NR_02727	0.00011277	0.292356
8156569	NR_029664	MIR23B	NR_029664 // MIR23B // microRNA 23b // 9q22.32 // 407011 /// AF043897 // C9orf3 // chro	2.86E-07	0.378052
8161423	NR_003674	KGFLP1	NR_003674 // KGFLP1 // keratinocyte growth factor-like protein 1 // 9p11.2 // 387628 //	0.00326026	-0.225047
8161455	NR_003674	KGFLP1	NR_003674 // KGFLP1 // keratinocyte growth factor-like protein 1 // 9p11.2 // 387628 //	0.00326026	-0.225047
8155487	NR_003674	KGFLP1	NR_003674 // KGFLP1 // keratinocyte growth factor-like protein 1 // 9p11.2 // 387628 //	0.00326026	-0.225047
8169240	NM_002764	PRPS1	NM_002764 // PRPS1 // phosphoribosyl pyrophosphate synthetase 1 // Xq21.32-q24 // 5631	0.000752435	-0.255833
8175217	NM_001448	GPC4	NM_001448 // GPC4 // glypican 4 // Xq26.1 // 2239 /// ENST00000370828 // GPC4 // glypic	9.47E-06	-0.32358
8171472	NM_020665	TMEM27	NM_020665 // TMEM27 // transmembrane protein 27 // Xp22 // 57393 /// ENST00000380342 //	0.000403822	-0.271639
7900336	AK023285	MACF1	AK023285 // MACF1 // microtubule-actin crosslinking factor 1 // 1p32-p31 // 23499 /// A	0.000494908	0.258928
8154973	NM_203299	C9orf131	NM_203299 // C9orf131 // chromosome 9 open reading frame 131 // 9p13.3 // 138724 /// NM	0.00613929	0.207473

7995803	NR_036677	MT1JP	NR_036677 // MT1JP // metallothionein 1J (pseudogene) // 16q13 // 4498 /// AF348994 //	7.30E-06	-0.34385
8171823	NM_024122	APOO	NM_024122 // APOO // apolipoprotein O // Xp22.11 // 79135 /// NR_026545 // APOO // apol	0.000130615	-0.290533
8054930	NM_032390	MKI67IP	NM_032390 // MKI67IP // MKI67 (FHA domain) interacting nucleolar phosphoprotein // 2q14	4.43E-05	-0.306458
8021635	NM_001143818	SERPINB2	NM_001143818 // SERPINB2 // serpin peptidase inhibitor, clade B (ovalbumin), member 2 /	9.41E-06	-0.33489
7912582	NM_001013407	PRAMEF5	NM_001013407 // PRAMEF5 // PRAME family member 5 // 1p36.21 // 343068 /// NM_001010889	0.00267011	0.232688
8091550	NM_020776	KIAA1328	NM_020776 // KIAA1328 // KIAA1328 // 18q12.2 // 57536 /// ENST00000280020 // KIAA1328 /	0.00132129	0.248591
8149955	NM_018492	PBK	NM_018492 // PBK // PDZ binding kinase // 8p21.2 // 55872 /// ENST00000301905 // PBK //	0.00615468	-0.209703
7902738	NM_012128	CLCA4	NM_012128 // CLCA4 // chloride channel accessory 4 // 1p31-p22 // 22802 /// NR_024602 /	0.00459256	-0.219467
8146685	NM_015169	RRS1	NM_015169 // RRS1 // RRS1 ribosome biogenesis regulator homolog (S. cerevisiae) // 8q13	1.11E-05	-0.328684
7971159	AF463496	LOC445341	AF463496 // LOC445341 // TRAFs and NIK-associated protein // --- // 445341	0.0032526	0.220351
8099537	---		---	9.44E-07	0.352839
8152703	NM_058229	FBXO32	NM_058229 // FBXO32 // F-box protein 32 // 8q24.13 // 114907 /// NM_148177 // FBXO32 //	0.000574837	-0.259682
8161388	NM_001012419	ANKRD20A3	NM_001012419 // ANKRD20A3 // ankyrin repeat domain 20 family, member A3 // 9p12 // 4414	0.000709002	0.258635
8155397	NM_001012419	ANKRD20A3	NM_001012419 // ANKRD20A3 // ankyrin repeat domain 20 family, member A3 // 9p12 // 4414	0.000709002	0.258636
8062623	NM_002660	PLCG1	NM_002660 // PLCG1 // phospholipase C, gamma 1 // 20q12-q13.1 // 5335 /// NM_182811 //	0.00761325	0.206481

8156228	NM_001912	CTSL1	NM_001912 // CTSL1 // cathepsin L1 // 9q21.33 // 1514 /// NM_145918 // CTSL1 // catheps	0.0012564	-0.244872
8089145	NM_015429	ABI3BP	NM_015429 // ABI3BP // ABI family, member 3 (NESH) binding protein // 3q12 // 25890 ///	0.00494523	0.217918
7995377	---		---	0.00648541	0.208666
8034806	NM_005804	DDX39	NM_005804 // DDX39 // DEAD (Asp-Glu-Ala-Asp) box polypeptide 39 // 19p13.12 // 10212 //	0.000105938	-0.293392
8110450	NM_031266	HNRNPAB	NM_031266 // HNRNPAB // heterogeneous nuclear ribonucleoprotein A/B // 5q35.3 // 3182 /	9.18E-06	-0.337835
8125843	NM_012391	SPDEF	NM_012391 // SPDEF // SAM pointed domain containing ets transcription factor // 6p21.3	0.00236741	-0.232303
8155574	NM_032250	ANKRD20A1	NM_032250 // ANKRD20A1 // ankyrin repeat domain 20 family, member A1 // 9q13 // 84210 /	0.000696987	0.259052
7979085	NM_002863	PYGL	NM_002863 // PYGL // phosphorylase, glycogen, liver // 14q21-q22 // 5836 /// NM_0011639	1.31E-05	-0.330104
7995834	NR_003669	MT1IP	NR_003669 // MT1IP // metallothionein 1I (pseudogene) // 16q13 // 644314 /// AF348997 /	3.29E-06	-0.356295
8048717	NM_152386	SGPP2	NM_152386 // SGPP2 // sphingosine-1-phosphate phosphatase 2 // 2q36.1 // 130367 /// ENS	6.06E-08	-0.396201
7919580	AK125737	LOC440570	AK125737 // LOC440570 // hypothetical LOC440570 // 1p36.13 // 440570 /// AK125737 // LO	5.59E-05	0.30025
7919596	AK125737	LOC440570	AK125737 // LOC440570 // hypothetical LOC440570 // 1p36.13 // 440570 /// AK125737 // LO	5.59E-05	0.30025
7908003	NM_030769	NPL	NM_030769 // NPL // N-acetylneuraminate pyruvate lyase (dihydrodipicolinate synthase) /	0.0036865	-0.219917
8107897	NM_003687	PDLIM4	NM_003687 // PDLIM4 // PDZ and LIM domain 4 // 5q31.1 // 8572 /// NM_001131027 // PDLIM	2.41E-05	-0.316794
8074978	---		---	0.0072203	0.205758
8074789	---		---	0.00290215	0.226586

7970844	NM_001014380	KATNAL1	NM_001014380 // KATNAL1 // katanin p60 subunit A-like 1 // 13q12.3 // 84056 /// NM_0321	0.00437314	0.221597
8013112	NM_016084	RASD1	NM_016084 // RASD1 // RAS, dexamethasone-induced 1 // 17p11.2 // 51655 /// ENST00000225	0.00123591	-0.253405
7965064	NM_020841	OSBPL8	NM_020841 // OSBPL8 // oxysterol binding protein-like 8 // 12q14 // 114882 /// NM_00100	0.00697516	-0.207568
7942255	NM_001012503	KRTAP5-7	NM_001012503 // KRTAP5-7 // keratin associated protein 5-7 // 11q13.4 // 440050 /// NM_	0.000180606	0.280737
7925741	NM_001004695	OR2T33	NM_001004695 // OR2T33 // olfactory receptor, family 2, subfamily T, member 33 // 1q44	0.00977951	0.200342
7948354	NM_145016	GLYATL2	NM_145016 // GLYATL2 // glycine-N-acyltransferase-like 2 // 11q12.1 // 219970 /// ENST0	0.00267794	-0.230197
8097480	NM_057175	NAA15	NM_057175 // NAA15 // N(alpha)-acetyltransferase 15, NatA auxiliary subunit // 4q31.1 /	0.000324946	-0.264645
8154951	NM_002065	GLUL	NM_002065 // GLUL // glutamate-ammonia ligase // 1q31 // 2752 /// NM_001033044 // GLUL	0.00936983	-0.200202
8048976	---		---	0.00131195	0.240557
7985364	NM_172217	IL16	NM_172217 // IL16 // interleukin 16 (lymphocyte chemoattractant factor) // 15q26.3 // 3	0.000361791	0.274088
8135480	NM_012328	DNAJB9	NM_012328 // DNAJB9 // DnaJ (Hsp40) homolog, subfamily B, member 9 // 7q31|14q24.2-q24.	0.00284688	-0.232105
8135204	NM_001031618	SPDYE2	NM_001031618 // SPDYE2 // speedy homolog E2 (Xenopus laevis) // 7q22.1 // 441273 /// NM	8.24E-05	0.295997
7907486	NM_032522	ZBTB37	NM_032522 // ZBTB37 // zinc finger and BTB domain containing 37 // 1q25.1 // 84614 ///	0.00103986	0.252617
7902553	NM_006417	IFI44	NM_006417 // IFI44 // interferon-induced protein 44 // 1p31.1 // 10561 /// ENST00000370	0.00818772	0.203185
7916491	---		---	0.00814086	-0.208134

8145782	NM_032509	MAK16	NM_032509 // MAK16 // MAK16 homolog (S. cerevisiae) // 8p12 // 84549 /// NM_001102401 /	0.00443128	-0.217176
8067839	NR_003674	KGFLP1	NR_003674 // KGFLP1 // keratinocyte growth factor-like protein 1 // 9p11.2 // 387628 //	0.00599492	-0.210842
7898677	NM_004807	HS6ST1	NM_004807 // HS6ST1 // heparan sulfate 6-O-sulfotransferase 1 // 2q21 // 9394 /// ENST0	0.00597044	-0.208231
8139977	NM_001013739	STAG3L3	NM_001013739 // STAG3L3 // stromal antigen 3-like 3 // 7q11.23 // 442578 /// NM_018991	0.00186994	0.234997
7993349	NM_006985	NPIP	NM_006985 // NPIP // nuclear pore complex interacting protein // 16p13.11 // 9284 /// E	0.00912804	0.195439
8059244	NM_024536	CHPF	NM_024536 // CHPF // chondroitin polymerizing factor // 2q35 // 79586 /// NM_001195731	0.00182716	-0.239878
8167912	NM_007250	KLF8	NM_007250 // KLF8 // Kruppel-like factor 8 // Xp11.21 // 11279 /// NM_001159296 // KLF8	0.0031425	0.222057
7924096	NM_002497	NEK2	NM_002497 // NEK2 // NIMA (never in mitosis gene a)-related kinase 2 // 1q32.2-q41 // 4	0.00263632	-0.232735
8139845	---		---	0.00924182	0.200661
7938481	NM_015881	DKK3	NM_015881 // DKK3 // dickkopf homolog 3 (Xenopus laevis) // 11p15.2 // 27122 /// NM_013	0.00409357	0.224037
8078227	NM_003884	KAT2B	NM_003884 // KAT2B // K(lysine) acetyltransferase 2B // 3p24 // 8850 /// ENST0000026375	0.00419072	0.220986
7947396	NM_001326	CSTF3	NM_001326 // CSTF3 // cleavage stimulation factor, 3' pre-RNA, subunit 3, 77kDa // 11p1	0.00506509	-0.210886
7926934	---		---	0.00159132	0.24272
8135197	NM_001031618	SPDYE2	NM_001031618 // SPDYE2 // speedy homolog E2 (Xenopus laevis) // 7q22.1 // 441273 /// NM	4.87E-05	0.305408
8007931	NM_000212	ITGB3	NM_000212 // ITGB3 // integrin, beta 3 (platelet glycoprotein IIIa, antigen CD61) // 17	7.11E-07	-0.367548
7939376	NM_174902	LDLRAD3	NM_174902 // LDLRAD3 // low density lipoprotein receptor class A domain containing 3 //	0.00502784	-0.218494

7974117	NM_001017923	C14orf28	NM_001017923 // C14orf28 // chromosome 14 open reading frame 28 // 14q21.2 // 122525 //	3.27E-05	0.312766
7963689	NM_003717	NPFF	NM_003717 // NPFF // neuropeptide FF-amide peptide precursor // 12q13.13 // 8620 /// EN	0.00684455	0.205157
7912555	NM_001009611	PRAMEF4	NM_001009611 // PRAMEF4 // PRAME family member 4 // 1p36.21 // 400735 /// NM_001013407	0.00129553	0.2461
8022434	---		---	4.59E-06	0.340905
7993359	NM_006985	NPIP	NM_006985 // NPIP // nuclear pore complex interacting protein // 16p13.11 // 9284 /// E	0.00906896	0.1956
8003087	---		---	0.000993939	0.244957
8037222	NM_001816	CEACAM8	NM_001816 // CEACAM8 // carcinoembryonic antigen-related cell adhesion molecule 8 // 19	0.00448906	-0.220465
7965681	NM_153687	IKBIP	NM_153687 // IKBIP // IKBKB interacting protein // 12q23.1 // 121457 /// NM_201612 // I	0.000801651	-0.261606
8049574	NM_080678	UBE2F	NM_080678 // UBE2F // ubiquitin-conjugating enzyme E2F (putative) // 2q37.3 // 140739 /	0.0100048	-0.200735
8051197	NM_024584	CCDC121	NM_024584 // CCDC121 // coiled-coil domain containing 121 // 2p23.3 // 79635 /// NM_001	0.000654414	0.260399
7956076	NM_001798	CDK2	NM_001798 // CDK2 // cyclin-dependent kinase 2 // 12q13 // 1017 /// NM_052827 // CDK2 /	0.000336059	-0.275786
8129497	NM_001431	EPB41L2	NM_001431 // EPB41L2 // erythrocyte membrane protein band 4.1-like 2 // 6q23 // 2037 //	0.00999147	0.200311
7995783	NM_005953	MT2A	NM_005953 // MT2A // metallothionein 2A // 16q13 // 4502 /// ENST00000245185 // MT2A //	4.43E-07	-0.382151
7928291	NM_004273	CHST3	NM_004273 // CHST3 // carbohydrate (chondroitin 6) sulfotransferase 3 // 10q22.1 // 946	0.000184433	-0.282854
8130408	NM_001130700	IPCEF1	NM_001130700 // IPCEF1 // interaction protein for cytohesin exchange factors 1 // 6q25.	0.000658052	-0.257647
8167560	NM_001098406	GAGE12J	NM_001098406 // GAGE12J // G antigen 12J // Xp11.23 // 729396 /// NM_001098412 // GAGE1	0.000335819	0.268122

7915184	NM_017821	RHBDL2	NM_017821 // RHBDL2 // rhomboid, veinlet-like 2 (Drosophila) // 1p34.3 // 54933 /// ENS	0.00953724	-0.198926
8034873	NM_013447	EMR2	NM_013447 // EMR2 // egf-like module containing, mucin-like, hormone receptor-like 2 //	1.67E-05	-0.321418
8125775	AY568085	C6orf125	AY568085 // C6orf125 // chromosome 6 open reading frame 125 // 6p21.31 // 84300 /// AF0	5.19E-05	-0.308974
8100310	---		---	2.19E-05	0.321924
7948211	AB209132	SSRP1	AB209132 // SSRP1 // structure specific recognition protein 1 // 11q12 // 6749 /// ENST	0.00860678	0.200442
7989473	NM_001007595	C2CD4B	NM_001007595 // C2CD4B // C2 calcium-dependent domain containing 4B // 15q22.2 // 38812	0.00453023	-0.218047
8175023	NM_016032	ZDHHC9	NM_016032 // ZDHHC9 // zinc finger, DHHC-type containing 9 // Xq26.1 // 51114 /// NM_00	3.51E-06	-0.344434
8042144	NM_002908	REL	NM_002908 // REL // v-rel reticuloendotheliosis viral oncogene homolog (avian) // 2p13-	2.03E-05	-0.323825
8079058	---		---	0.00396722	0.224901
8013525	---		---	0.00108283	0.249399
8142102	NM_020725	ATXN7L1	NM_020725 // ATXN7L1 // ataxin 7-like 1 // 7q22.3 // 222255 /// NM_138495 // ATXN7L1 //	0.0054222	0.215812
8077815	NR_026829	C3orf42	NR_026829 // C3orf42 // chromosome 3 open reading frame 42 // 3p25.3 // 84657 /// AF280	0.00406214	0.218097
8047339	NM_014670	BZW1	NM_014670 // BZW1 // basic leucine zipper and W2 domains 1 // 2q33 // 9689 /// ENST0000	0.000241573	-0.278146
8050255	NM_024894	NOL10	NM_024894 // NOL10 // nucleolar protein 10 // 2p25.1 // 79954 /// ENST00000381685 // NO	0.0074959	-0.208704
8068024	NM_021219	JAM2	NM_021219 // JAM2 // junctional adhesion molecule 2 // 21q21.2 // 58494 /// ENST0000048	2.86E-05	0.309837
8140356	---		---	0.00108927	0.253171
7976826	NR_003219	SNORD114-26	NR_003219 // SNORD114-26 // small nucleolar RNA, C/D box 114-26 // 14q32 // 767606	0.00857928	-0.205087
8155455	AK126863	NCRNA00268	AK126863 // NCRNA00268 // non-protein coding RNA 268 // 9p11.2 // 441426	0.000478599	0.262343

7982878	NM_007236	CHP	NM_007236 // CHP // calcium binding protein P22 // 15q13.3 // 11261 /// ENST00000334660	0.00348811	-0.21729
8130556	NM_001024465	SOD2	NM_001024465 // SOD2 // superoxide dismutase 2, mitochondrial // 6q25.3 // 6648 /// NM_	1.48E-06	-0.369322
7907788	---		---	0.00329321	0.231151
7905054	---		---	0.00831164	0.199729
8091327	NM_021105	PLSCR1	NM_021105 // PLSCR1 // phospholipid scramblase 1 // 3q23 // 5359 /// ENST00000342435 //	0.00128595	-0.24645
7944006	NM_016090	RBM7	NM_016090 // RBM7 // RNA binding motif protein 7 // 11q23.1-q23.2 // 10179 /// ENST0000	0.00254487	-0.231558
8167482	NM_001477	GAGE12I	NM_001477 // GAGE12I // G antigen 12I // Xp11.23 // 26748 /// NM_001474 // GAGE4 // G a	0.000706663	0.25231
7923547	NM_001276	CHI3L1	NM_001276 // CHI3L1 // chitinase 3-like 1 (cartilage glycoprotein-39) // 1q32.1 // 1116	3.05E-05	-0.305036
8032839	NM_032108	SEMA6B	NM_032108 // SEMA6B // sema domain, transmembrane domain (TM), and cytoplasmic domain,	0.0019208	-0.2417
8161174	NM_005476	GNE	NM_005476 // GNE // glucosamine (UDP-N-acetyl)-2-epimerase/N-acetylmannosamine kinase /	0.000256056	-0.277327
8038117	NM_001352	DBP	NM_001352 // DBP // D site of albumin promoter (albumin D-box) binding protein // 19q13	0.000119499	0.294107
8094130	NM_001105662	USP17	NM_001105662 // USP17 // ubiquitin specific peptidase 17 // 4p15 // 391627 /// NR_02727	0.000114497	0.292448
8041206	NM_030915	LBH	NM_030915 // LBH // limb bud and heart development homolog (mouse) // 2p23.1 // 81606 /	6.65E-05	0.302232
8123080	NM_001009991	SYTL3	NM_001009991 // SYTL3 // synaptotagmin-like 3 // 6q25.3 // 94120 /// ENST00000360448 //	0.00177839	-0.238498

8156581	NM_001010895	C9orf102	NM_001010895 // C9orf102 // chromosome 9 open reading frame 102 // 9q22.32 // 375748 //	0.00566163	0.213903
8149330	NM_147780	CTSB	NM_147780 // CTSB // cathepsin B // 8p22 // 1508 /// NM_147781 // CTSB // cathepsin B /	0.00229794	-0.232643
7923753	NM_030952	NUAK2	NM_030952 // NUAK2 // NUAK family, SNF1-like kinase, 2 // 1q32.1 // 81788 /// ENST00000	0.000507994	-0.268701
8076403	NM_000262	NAGA	NM_000262 // NAGA // N-acetylgalactosaminidase, alpha- // 22q13-qter|22q11 // 4668 ///	0.00925045	-0.197014
8019316	NM_006907	PYCR1	NM_006907 // PYCR1 // pyrroline-5-carboxylate reductase 1 // 17q25.3 // 5831 /// NM_153	0.00911812	-0.204069
8062190	NM_003116	SPAG4	NM_003116 // SPAG4 // sperm associated antigen 4 // 20q11.21 // 6676 /// ENST0000037427	0.00700868	-0.210697
8041168	NR_002741	SNORD53	NR_002741 // SNORD53 // small nucleolar RNA, C/D box 53 // 2p23.2 // 26796	0.00699424	-0.198592
8081548	NM_015480	PVRL3	NM_015480 // PVRL3 // poliovirus receptor-related 3 // 3q13 // 25945 /// ENST0000048530	0.00907181	0.201754
7982248	---		---	0.00285685	0.231867
7999634	AK294177	NPIP	AK294177 // NPIP // nuclear pore complex interacting protein // 16p13.11 // 9284 /// AK	0.00325514	0.219417
7930498	NM_016234	ACSL5	NM_016234 // ACSL5 // acyl-CoA synthetase long-chain family member 5 // 10q25.1-q25.2 /	0.00980055	-0.198131
7944601	---		---	0.0102358	0.192633
8002937	---		---	0.00330769	0.222034
7898373	---		---	0.00119278	0.251165
8055281	NR_002826	LOC401010	NR_002826 // LOC401010 // nucleolar complex associated 2 homolog (S. cerevisiae) pseudo	0.00904451	0.198844
8121515	NM_018593	SLC16A10	NM_018593 // SLC16A10 // solute carrier family 16, member 10 (aromatic amino acid trans	1.37E-06	-0.353815
8106532	---		---	0.00204826	0.231043
8151605	AF495523	REXO1L1	AF495523 // REXO1L1 // REX1, RNA exonuclease 1 homolog (S. cerevisiae)-like 1 // 8q21.2	8.94E-05	0.30248

8151613	AF495523	REXO1L1	AF495523 // REXO1L1 // REX1, RNA exonuclease 1 homolog (S. cerevisiae)-like 1 // 8q21.2	8.94E-05	0.30248
8151617	AF495523	REXO1L1	AF495523 // REXO1L1 // REX1, RNA exonuclease 1 homolog (S. cerevisiae)-like 1 // 8q21.2	8.94E-05	0.30248
8151621	AF495523	REXO1L1	AF495523 // REXO1L1 // REX1, RNA exonuclease 1 homolog (S. cerevisiae)-like 1 // 8q21.2	8.94E-05	0.30248
8151625	AF495523	REXO1L1	AF495523 // REXO1L1 // REX1, RNA exonuclease 1 homolog (S. cerevisiae)-like 1 // 8q21.2	8.94E-05	0.30248
7939723	NM_001003676	C11orf49	NM_001003676 // C11orf49 // chromosome 11 open reading frame 49 // 11p11.2 // 79096 ///	0.00975122	-0.201966
8172787	NM_003147	SSX2	NM_003147 // SSX2 // synovial sarcoma, X breakpoint 2 // Xp11.22 // 6757 /// NM_175698	0.00194228	0.235173
8139996	NR_003664	SPDYE8P	NR_003664 // SPDYE8P // speedy homolog E8 (Xenopus laevis), pseudogene // 7q11.23 // 38	0.00147668	0.239141
7928752	NM_014394	GHITM	NM_014394 // GHITM // growth hormone inducible transmembrane protein // 10q23.1 // 2706	0.00575053	-0.214164
8161857	NM_018339	RFK	NM_018339 // RFK // riboflavin kinase // 9q21.13 // 55312 /// ENST00000376736 // RFK //	0.00191744	-0.237759
8149749	NM_003840	TNFRSF10D	NM_003840 // TNFRSF10D // tumor necrosis factor receptor superfamily, member 10d, decoy	0.0036878	-0.224821
8094340	NR_029631	MIR218-1	NR_029631 // MIR218-1 // microRNA 218-1 // 4p15.31 // 407000	3.09E-08	0.395626
7962689	NM_001017535	VDR	NM_001017535 // VDR // vitamin D (1,25-dihydroxyvitamin D3) receptor // 12q13.11 // 74	0.0018539	-0.23498
8169868	NM_006649	UTP14A	NM_006649 // UTP14A // UTP14, U3 small nucleolar ribonucleoprotein, homolog A (yeast) /	0.008554	-0.201999
8060949	NM_022096	ANKRD5	NM_022096 // ANKRD5 // ankyrin repeat domain 5 // 20pter-q11.23 // 63926 /// NM_198798	0.00345297	-0.224837
8003806	NM_014566	OR1D5	NM_014566 // OR1D5 // olfactory receptor, family 1, subfamily D, member 5 // 17p13.3 //	0.00295151	0.230402

8023154	NM_001100817	TCEB3CL	NM_001100817 // TCEB3CL // transcription elongation factor B polypeptide 3C-like // 18q	0.00912676	0.19937
7908672	NM_000299	PKP1	NM_000299 // PKP1 // plakophilin 1 (ectodermal dysplasia/skin fragility syndrome) // 1q	3.23E-05	-0.301623
8053057	---		---	0.0007705	0.253293
8017582	NM_018469	TEX2	NM_018469 // TEX2 // testis expressed 2 // 17q23.3 // 55852 /// ENST00000258991 // TEX2	0.00844808	-0.198693
7927936	NM_004728	DDX21	NM_004728 // DDX21 // DEAD (Asp-Glu-Ala-Asp) box polypeptide 21 // 10q21 // 9188 /// EN	8.37E-08	-0.392319
8133610	NM_001013739	STAG3L3	NM_001013739 // STAG3L3 // stromal antigen 3-like 3 // 7q11.23 // 442578 /// NM_018991	0.00115198	0.244718
8128850	NM_033125	SLC22A16	NM_033125 // SLC22A16 // solute carrier family 22 (organic cation/carnitine transporter	2.44E-05	-0.324374
8155414	NR_003366	ANKRD20B	NR_003366 // ANKRD20B // ankyrin repeat domain 20B // 2q11.1 // 729171 /// ENST00000456	0.0065197	0.210215
8161384	ENST0000045698:	LOC644249	ENST00000456982 // LOC644249 // hypothetical LOC644249 // 9q12 // 644249 /// ENST000004	0.00651965	0.210215
7975045	NM_005956	MTHFD1	NM_005956 // MTHFD1 // methylenetetrahydrofolate dehydrogenase (NADP+ dependent) 1, met	0.00363441	-0.220032
8104930	NM_004172	SLC1A3	NM_004172 // SLC1A3 // solute carrier family 1 (glial high affinity glutamate transport	1.17E-06	-0.35635
7983132	NM_020759	STARD9	NM_020759 // STARD9 // StAR-related lipid transfer (START) domain containing 9 // 15q15	6.40E-06	0.330875
7923582	---		---	0.000626166	-0.265641
8095362	NM_005953	MT2A	NM_005953 // MT2A // metallothionein 2A // 16q13 // 4502 /// ENST00000245185 // MT2A //	3.62E-07	-0.385605
8095728	NM_001432	EREG	NM_001432 // EREG // epiregulin // 4q13.3 // 2069 /// ENST00000244869 // EREG // epireg	0.00188852	-0.238918
8074593	NM_015672	RIMBP3	NM_015672 // RIMBP3 // RIMS binding protein 3 // 22q11.21 // 85376 /// NM_001128635 //	0.003027	0.228153

8167508	NM_001098406	GAGE12J	NM_001098406 // GAGE12J // G antigen 12J // Xp11.23 // 729396 /// NM_001098412 // GAGE1	0.000906574	0.249599
7915485	NM_006824	EBNA1BP2	NM_006824 // EBNA1BP2 // EBNA1 binding protein 2 // 1p35-p33 // 10969 /// NM_001159936	0.010183	-0.202497
8123819	NM_004280	EEF1E1	NM_004280 // EEF1E1 // eukaryotic translation elongation factor 1 epsilon 1 // 6p24.3 /	2.14E-05	-0.324202
8089701	NM_015642	ZBTB20	NM_015642 // ZBTB20 // zinc finger and BTB domain containing 20 // 3q13.2 // 26137 ///	0.0098003	0.197772
8048205	NM_000597	IGFBP2	NM_000597 // IGFBP2 // insulin-like growth factor binding protein 2, 36kDa // 2q33-q34	0.00372359	-0.216619
8001531	NM_005950	MT1G	NM_005950 // MT1G // metallothionein 1G // 16q13 // 4495 /// BC020757 // MT1G // metall	2.29E-07	-0.392678
8127989	NR_003044	SNORD50B	NR_003044 // SNORD50B // small nucleolar RNA, C/D box 50B // 6q14.3 // 692088	0.0090981	0.202959
8132070	NM_002047	GARS	NM_002047 // GARS // glycyl-tRNA synthetase // 7p15 // 2617 /// ENST00000389266 // GARS	5.38E-05	-0.307388
8156838	NM_006808	SEC61B	NM_006808 // SEC61B // Sec61 beta subunit // 9q22.32-q31.3 // 10952 /// ENST00000223641	0.000936684	-0.255326
8151609	NM_172239	REXO1L1	NM_172239 // REXO1L1 // REX1, RNA exonuclease 1 homolog (S. cerevisiae)-like 1 // 8q21.	0.000240738	0.280807
8166072	NM_021109	TMSB4X	NM_021109 // TMSB4X // thymosin beta 4, X-linked // Xq21.3-q22 // 7114 /// NM_183049 //	7.90E-05	0.297512
7931914	NM_000417	IL2RA	NM_000417 // IL2RA // interleukin 2 receptor, alpha // 10p15-p14 // 3559 /// ENST000003	0.000656185	-0.256335
8080676	NM_177966	PDE12	NM_177966 // PDE12 // phosphodiesterase 12 // 3p14.3 // 201626 /// ENST00000311180 // P	2.35E-05	-0.321914
8119088	NR_037151	CDKN1A	NR_037151 // CDKN1A // cyclin-dependent kinase inhibitor 1A (p21, Cip1) // 6p21.2 // 10	1.84E-06	-0.352654
8121112	---		---	0.0001687	0.285992
7951297	NM_002426	MMP12	NM_002426 // MMP12 // matrix metallopeptidase 12 (macrophage elastase) // 11q22.3 // 43	0.00702734	-0.207913

7978595	NM_013448	BAZ1A	NM_013448 // BAZ1A // bromodomain adjacent to zinc finger domain, 1A // 14q13.2 // 1117	1.24E-05	-0.327805
8102362	NM_052864	TIFA	NM_052864 // TIFA // TRAF-interacting protein with forkhead-associated domain // 4q25 /	0.000804239	-0.255963
8163629	NM_001244	TNFSF8	NM_001244 // TNFSF8 // tumor necrosis factor (ligand) superfamily, member 8 // 9q33 //	0.00183023	-0.237385
7933008	---		---	3.78E-05	0.30091
8157144	NM_017832	C9orf6	NM_017832 // C9orf6 // chromosome 9 open reading frame 6 // 9q31.3 // 54942 /// NM_0036	0.00986762	-0.20098
8138857	NM_024051	GGCT	NM_024051 // GGCT // gamma-glutamylcyclotransferase // 7p15-p14 // 79017 /// ENST000002	8.26E-05	-0.291152
8140211	NR_003664	SPDYE8P	NR_003664 // SPDYE8P // speedy homolog E8 (Xenopus laevis), pseudogene // 7q11.23 // 38	0.000779315	0.253942
8124648	NM_052967	MAS1L	NM_052967 // MAS1L // MAS1 oncogene-like // 6p21 // 116511 /// ENST00000377127 // MAS1L	0.0022455	0.240425
8178293	NM_052967	MAS1L	NM_052967 // MAS1L // MAS1 oncogene-like // 6p21 // 116511 /// ENST00000377127 // MAS1L	0.0022455	0.240425
8179593	NM_052967	MAS1L	NM_052967 // MAS1L // MAS1 oncogene-like // 6p21 // 116511 /// ENST00000377127 // MAS1L	0.0022455	0.240425
8098177	NM_007246	KLHL2	NM_007246 // KLHL2 // kelch-like 2, Mayven (Drosophila) // 4q21.2 // 11275 /// NM_00116	0.000413489	-0.272109
7930870	NM_020243	TOMM22	NM_020243 // TOMM22 // translocase of outer mitochondrial membrane 22 homolog (yeast) /	0.0102794	-0.202824
8003611	NM_024792	FAM57A	NM_024792 // FAM57A // family with sequence similarity 57, member A // 17p13.3 // 79850	2.00E-06	-0.351242
7997381	NM_001100624	CENPN	NM_001100624 // CENPN // centromere protein N // 16q23.2 // 55839 /// NM_001100625 // C	0.00108089	-0.248678
7985240	NM_007364	TMED3	NM_007364 // TMED3 // transmembrane emp24 protein transport domain containing 3 // 15q2	7.63E-05	-0.301571
7952522	NM_031307	PUS3	NM_031307 // PUS3 // pseudouridylate synthase 3 // 11q24.2 // 83480 /// ENST00000227474	0.0016659	-0.239051
7919560	---		---	0.00366972	0.225387

7958174	NM_003330	TXNRD1	NM_003330 // TXNRD1 // thioredoxin reductase 1 // 12q23-q24.1 // 7296 /// NM_001093771	4.56E-07	-0.372731
8112803	NM_005779	LHFPL2	NM_005779 // LHFPL2 // lipoma HMGIC fusion partner-like 2 // 5q14.1 // 10184 /// AY3099	0.00842389	-0.19682
8133582	NR_003664	SPDYE8P	NR_003664 // SPDYE8P // speedy homolog E8 (Xenopus laevis), pseudogene // 7q11.23 // 38	0.000244603	0.275774
8136727	BC030533	TRBC1	BC030533 // TRBC1 // T cell receptor beta constant 1 // 7q34 // 28639 /// AK093303 // T	0.00813917	0.206121
7963567	NM_002273	KRT8	NM_002273 // KRT8 // keratin 8 // 12q13 // 3856 /// ENST00000293308 // KRT8 // keratin	0.00958341	-0.203025
8059376	NM_006216	SERPINE2	NM_006216 // SERPINE2 // serpin peptidase inhibitor, clade E (nexin, plasminogen activa	0.000330092	-0.274904
8068180	NM_014586	HUNK	NM_014586 // HUNK // hormonally up-regulated Neu-associated kinase // 21q22.1 // 30811	0.00251325	-0.232645
7971671	NR_003923	GUCY1B2	NR_003923 // GUCY1B2 // guanylate cyclase 1, soluble, beta 2 // 13q14.3 // 2974 /// AF0	0.00773333	0.205878
7960896	ENST0000032933(	OR7E87P	ENST00000329336 // OR7E87P // olfactory receptor, family 7, subfamily E, member 87 pseu	0.000200624	0.277473
8107578	NM_152546	SRFBP1	NM_152546 // SRFBP1 // serum response factor binding protein 1 // 5q23.1 // 153443 ///	0.000299222	-0.27052
7911854	NR_033711	KIAA0495	NR_033711 // KIAA0495 // KIAA0495 // 1p36.32 // 57212 /// NR_033710 // KIAA0495 // KIAA	0.00295069	0.22704
8117470	NR_027795	BTN2A3	NR_027795 // BTN2A3 // butyrophilin, subfamily 2, member A3 // 6p22.1 // 54718 /// BC14	0.00961902	0.201505
8075886	NM_000878	IL2RB	NM_000878 // IL2RB // interleukin 2 receptor, beta // 22q13|22q13.1 // 3560 /// ENST000	0.0048192	0.218725
8116848	NM_017906	PAK1IP1	NM_017906 // PAK1IP1 // PAK1 interacting protein 1 // 6p24.2 // 55003 /// ENST000003795	0.00665141	-0.210477
7978285	NM_001198592	ADCY4	NM_001198592 // ADCY4 // adenylate cyclase 4 // 14q12 // 196883 /// NM_139247 // ADCY4	0.00890629	0.195986

7946326	NM_153445	OR5P3	NM_153445 // OR5P3 // olfactory receptor, family 5, subfamily P, member 3 // 11p15.4 //	0.00894196	0.19788
8060082	NM_001080835	PRR21	NM_001080835 // PRR21 // proline rich 21 // 2q37.3 // 643905 /// ENST00000408934 // PRR	0.00283713	0.231093
8151871	NM_057749	CCNE2	NM_057749 // CCNE2 // cyclin E2 // 8q22.1 // 9134 /// ENST00000308108 // CCNE2 // cycli	0.0079593	-0.201249
8140840	NM_024636	STEAP4	NM_024636 // STEAP4 // STEAP family member 4 // 7q21.12 // 79689 /// ENST00000380079 //	5.56E-06	-0.332373
8007803	NR_026905	C17orf69	NR_026905 // C17orf69 // chromosome 17 open reading frame 69 // 17q21.31 // 147081 ///	0.00489359	0.21719
7946559	NM_001017998	GNG10	NM_001017998 // GNG10 // guanine nucleotide binding protein (G protein), gamma 10 // 9q	0.000165636	-0.284577
7931268	NM_016567	BCCIP	NM_016567 // BCCIP // BRCA2 and CDKN1A interacting protein // 10q26.1 // 56647 /// NM_0	0.000854347	-0.257733
8051187	NM_022823	FNDC4	NM_022823 // FNDC4 // fibronectin type III domain containing 4 // 2p23.3 // 64838 /// E	0.000184363	-0.286961
7911178	NM_004895	NLRP3	NM_004895 // NLRP3 // NLR family, pyrin domain containing 3 // 1q44 // 114548 /// NM_00	5.70E-05	-0.312125
7985522	NM_207517	ADAMTSL3	NM_207517 // ADAMTSL3 // ADAMTS-like 3 // 15q25.2 // 57188 /// ENST00000286744 // ADAMT	0.0017884	0.235939
8101874	NM_000667	ADH1A	NM_000667 // ADH1A // alcohol dehydrogenase 1A (class I), alpha polypeptide // 4q23 //	0.000151079	0.289503
7898030	NM_001099852	PRAMEF20	NM_001099852 // PRAMEF20 // PRAME family member 20 // 1p36.21 // 645425 /// NM_00110011	0.00303294	0.231002
8162533	NM_001083603	PTCH1	NM_001083603 // PTCH1 // patched 1 // 9q22.3 // 5727 /// NM_001083602 // PTCH1 // patch	0.000292542	0.280262
8167027	NM_152869	RGN	NM_152869 // RGN // regucalcin (senescence marker protein-30) // Xp11.3 // 9104 /// NM_	0.000547588	0.265645
8121502	NM_138408	GTF3C6	NM_138408 // GTF3C6 // general transcription factor IIIC, polypeptide 6, alpha 35kDa //	0.000304542	-0.278033

8044574	NM_173842	IL1RN	NM_173842 // IL1RN // interleukin 1 receptor antagonist // 2q14.2 // 3557 /// NM_173841	2.28E-06	-0.344473
8056408	NM_004482	GALNT3	NM_004482 // GALNT3 // UDP-N-acetyl-alpha-D-galactosamine:polypeptide N-acetylgalactosa	0.00444125	-0.220996
8045539	NM_003937	KYNU	NM_003937 // KYNU // kynureninase (L-kynurenine hydrolase) // 2q22.2 // 8942 /// NM_001	0.00250812	-0.228525
8156523	NR_029481	MIRLET7D	NR_029481 // MIRLET7D // microRNA let-7d // 9q22.32 // 406886	9.39E-05	0.289438
7949719	NM_001040716	PC	NM_001040716 // PC // pyruvate carboxylase // 11q13.4-q13.5 // 5091 /// NM_000920 // PC	3.36E-05	-0.303544
8062211	NM_032194	RPF2	NM_032194 // RPF2 // ribosome production factor 2 homolog (S. cerevisiae) // 6q21 // 84	9.18E-05	-0.289846
8049544	NM_001137550	LRRFIP1	NM_001137550 // LRRFIP1 // leucine rich repeat (in FLII) interacting protein 1 // 2q37.	0.000393728	0.269393
7972557	NM_004951	GPR183	NM_004951 // GPR183 // G protein-coupled receptor 183 // 13q32.3 // 1880 /// ENST000003	0.000232765	-0.281336
7919556	---		---	0.00411374	0.22294
7991374	NM_002168	IDH2	NM_002168 // IDH2 // isocitrate dehydrogenase 2 (NADP+), mitochondrial // 15q26.1 // 34	1.23E-06	-0.366685
8141750	NM_001031618	SPDYE2	NM_001031618 // SPDYE2 // speedy homolog E2 (Xenopus laevis) // 7q22.1 // 441273 /// NM	0.00029707	0.275848
8155591	NR_003366	ANKRD20B	NR_003366 // ANKRD20B // ankyrin repeat domain 20B // 2q11.1 // 729171 /// ENST00000417	0.00924629	0.20187
8004184	NM_017523	XAF1	NM_017523 // XAF1 // XIAP associated factor 1 // 17p13.1 // 54739 /// NM_199139 // XAF1	0.000467261	0.265742
8134374	---		---	0.00406924	0.217302
8149345	ENST0000042069:	CTSB	ENST00000420692 // CTSB // cathepsin B // 8p22 // 1508 /// ENST00000420692 // CTSB // c	0.000326515	0.266549
7938388	---		---	0.00102833	0.251741
8073311	NM_022098	XPNPEP3	NM_022098 // XPNPEP3 // X-prolyl aminopeptidase (aminopeptidase P) 3, putative // 22q13	0.00350445	-0.227161

8114443	NM_004730	ETF1	NM_004730 // ETF1 // eukaryotic translation termination factor 1 // 5q31.1 // 2107 ///	0.00153886	-0.248375
8051583	NM_000104	CYP1B1	NM_000104 // CYP1B1 // cytochrome P450, family 1, subfamily B, polypeptide 1 // 2p21 //	1.18E-05	-0.321448
8173755	NM_004867	ITM2A	NM_004867 // ITM2A // integral membrane protein 2A // Xq13.3-Xq21.2 // 9452 /// NM_0011	0.000374283	0.268562
7898549	NM_016183	MRTO4	NM_016183 // MRTO4 // mRNA turnover 4 homolog (S. cerevisiae) // 1p36.13 // 51154 /// E	0.00129081	-0.242797
8056220	NM_015446	AHCTF1	NM_015446 // AHCTF1 // AT hook containing transcription factor 1 // 1q44 // 25909 /// A	0.00871218	-0.202742
8117685	NM_024493	ZKSCAN3	NM_024493 // ZKSCAN3 // zinc finger with KRAB and SCAN domains 3 // 6p22.1 // 80317 ///	0.00138737	0.249623
7985934	NM_020210	SEMA4B	NM_020210 // SEMA4B // sema domain, immunoglobulin domain (Ig), transmembrane domain (T	3.70E-08	-0.405191
8144526	---		---	0.00880302	0.203515
7951034	NR_002920	SNORA8	NR_002920 // SNORA8 // small nucleolar RNA, H/ACA box 8 // 11q21 // 654320 /// AK128061	0.00209541	-0.229458
7982269	---		---	0.00404189	0.222237
7987025	---		---	0.00404189	0.222237
8127180	NM_019036	HMGCLL1	NM_019036 // HMGCLL1 // 3-hydroxymethyl-3-methylglutaryl-CoA lyase-like 1 // 6p12.1 //	0.000399763	0.270958
7929012	NM_020799	STAMBPL1	NM_020799 // STAMBPL1 // STAM binding protein-like 1 // 10q23.31 // 57559 /// ENST00000	0.00743804	0.209591
8155602	NM_001012421	ANKRD20A2	NM_001012421 // ANKRD20A2 // ankyrin repeat domain 20 family, member A2 // 9p12 // 4414	0.00215126	0.236068
8061529	NM_014012	REM1	NM_014012 // REM1 // RAS (RAD and GEM)-like GTP-binding 1 // 20q11.21 // 28954 /// ENST	0.00066274	0.261385
7946569	NM_016422	RNF141	NM_016422 // RNF141 // ring finger protein 141 // 11p15.4 // 50862 /// ENST00000265981	0.0036517	-0.225773

8106193	NM_032175	UTP15	NM_032175 // UTP15 // UTP15, U3 small nucleolar ribonucleoprotein, homolog (S. cerevisi	0.00941186	-0.198275
8038904	NM_032679	ZNF577	NM_032679 // ZNF577 // zinc finger protein 577 // 19q13.41 // 84765 /// NM_001135590 //	0.000958318	0.25329
7902541	NM_006820	IFI44L	NM_006820 // IFI44L // interferon-induced protein 44-like // 1p31.1 // 10964 /// ENST00	0.00542988	0.213516
8133571	NR_003664	SPDYE8P	NR_003664 // SPDYE8P // speedy homolog E8 (Xenopus laevis), pseudogene // 7q11.23 // 38	0.000282855	0.272472
8006123	NM_001304	CPD	NM_001304 // CPD // carboxypeptidase D // 17q11.2 // 1362 /// ENST00000225719 // CPD //	1.05E-07	-0.396502
7930450	---		---	0.00107581	0.252659
8030113	NM_006666	RUVBL2	NM_006666 // RUVBL2 // RuvB-like 2 (E. coli) // 19q13.3 // 10856 /// ENST00000221413 //	0.00693496	-0.207096
7940253	NM_139249	MS4A6E	NM_139249 // MS4A6E // membrane-spanning 4-domains, subfamily A, member 6E // 11q12.2 /	1.22E-05	-0.33126
8095376	NM_005953	MT2A	NM_005953 // MT2A // metallothionein 2A // 16q13 // 4502 /// ENST00000245185 // MT2A //	9.30E-07	-0.374662
8079149	---		---	0.000458944	0.265532
8094556	NM_018290	PGM2	NM_018290 // PGM2 // phosphoglucomutase 2 // 4p14 // 55276 /// ENST00000381967 // PGM2	8.45E-05	-0.292166
8140009	NR_003664	SPDYE8P	NR_003664 // SPDYE8P // speedy homolog E8 (Xenopus laevis), pseudogene // 7q11.23 // 38	0.000249823	0.274902
8162880	NM_019051	MRPL50	NM_019051 // MRPL50 // mitochondrial ribosomal protein L50 // 9q31.1 // 54534 /// ENST0	0.000774794	-0.258777
7990452	---		---	0.000171291	0.28842
8138735	NM_019102	HOXA5	NM_019102 // HOXA5 // homeobox A5 // 7p15.2 // 3202 /// ENST00000222726 // HOXA5 // hom	4.79E-06	0.346397
8132031	NM_175887	PRR15	NM_175887 // PRR15 // proline rich 15 // 7p14.3 // 222171 /// ENST00000319694 // PRR15	0.00204525	-0.239559
8177046	---		---	0.00027036	-0.281624

8169073	NM_194324	TMSB15B	NM_194324 // TMSB15B // thymosin beta 15B // Xq22.2 // 286527 /// NR_003238 // H2BFXP /	0.000343379	0.275871
8160670	NM_004925	AQP3	NM_004925 // AQP3 // aquaporin 3 (Gill blood group) // 9p13 // 360 /// ENST00000297991	0.000233423	-0.281975
7919160	---		---	0.00233398	0.230292
7919166	---		---	0.00233398	0.230292
8026298	---		---	0.00713814	0.207181
8014035	---		---	0.00713814	0.207181
8140196	NM_001025202	STAG3L2	NM_001025202 // STAG3L2 // stromal antigen 3-like 2 // 7q11.23 // 442582 /// NM_0010137	0.00136716	0.240769
8155528	---		---	0.000967144	0.25517
7949896	ENST0000045433(	OR7E87P	ENST00000454336 // OR7E87P // olfactory receptor, family 7, subfamily E, member 87 pseu	0.000154365	0.284628
8052149	NM_014614	PSME4	NM_014614 // PSME4 // proteasome (prosome, macropain) activator subunit 4 // 2p16.2 //	1.38E-06	-0.354152
8140445	NR_003664	SPDYE8P	NR_003664 // SPDYE8P // speedy homolog E8 (Xenopus laevis), pseudogene // 7q11.23 // 38	0.00101128	0.24926
8085081	NM_016302	CRBN	NM_016302 // CRBN // cereblon // 3p26.2 // 51185 /// NM_001173482 // CRBN // cereblon /	1.94E-05	0.32591
7903753	NM_000848	GSTM2	NM_000848 // GSTM2 // glutathione S-transferase mu 2 (muscle) // 1p13.3 // 2946 /// NM_	0.00221523	0.23713
8084634	NM_016306	DNAJB11	NM_016306 // DNAJB11 // DnaJ (Hsp40) homolog, subfamily B, member 11 // 3q27.3 // 51726	0.00018038	-0.288144
8161507	---		---	0.000483926	0.267258
8136647	NM_018980	TAS2R5	NM_018980 // TAS2R5 // taste receptor, type 2, member 5 // 7q31.3-q32 // 54429 /// ENST	0.000630302	0.248906
7904364	NM_006784	WDR3	NM_006784 // WDR3 // WD repeat domain 3 // 1p12 // 10885 /// ENST00000349139 // WDR3 //	0.00130988	-0.24995
8102567	NM_018699	PRDM5	NM_018699 // PRDM5 // PR domain containing 5 // 4q25-q26 // 11107 /// ENST00000264808 /	0.000468987	0.266366

7924499	NM_003268	TLR5	NM_003268 // TLR5 // toll-like receptor 5 // 1q41-q42 // 7100 /// ENST00000366881 // TL	0.00209591	-0.241213
7960898	---		---	4.14E-05	0.304354
8017850	NM_017983	WIPI1	NM_017983 // WIPI1 // WD repeat domain, phosphoinositide interacting 1 // 17q24.2 // 55	1.26E-07	-0.393333
8159965	---		---	0.000378531	0.272837
8088371	NM_004944	DNASE1L3	NM_004944 // DNASE1L3 // deoxyribonuclease I-like 3 // 3p14.3 // 1776 /// ENST000003183	1.35E-05	0.329798
8151631	NR_003594	REXO1L2P	NR_003594 // REXO1L2P // REX1, RNA exonuclease 1 homolog (S. cerevisiae)-like 2 (pseudo	0.000343802	0.272455
8088636	---		---	0.00029131	0.272489
7938746	NM_054032	MRGPRX4	NM_054032 // MRGPRX4 // MAS-related GPR, member X4 // 11p15.1 // 117196 /// ENST0000031	0.00375118	0.223958
8028206	NM_003419	ZNF345	NM_003419 // ZNF345 // zinc finger protein 345 // 19q13.12 // 25850 /// ENST00000299672	0.0102947	0.200797
8121489	NM_001634	AMD1	NM_001634 // AMD1 // adenosylmethionine decarboxylase 1 // 6q21 // 262 /// NM_001033059	0.000883154	-0.254065
8092691	NM_001706	BCL6	NM_001706 // BCL6 // B-cell CLL/lymphoma 6 // 3q27 // 604 /// NM_001130845 // BCL6 // B	9.97E-09	-0.411202
8106403	NM_005242	F2RL1	NM_005242 // F2RL1 // coagulation factor II (thrombin) receptor-like 1 // 5q13 // 2150	0.00111305	-0.247874
7950990	NM_152313	SLC36A4	NM_152313 // SLC36A4 // solute carrier family 36 (proton/amino acid symporter), member	0.000176463	-0.282859
8016578	NM_005827	SLC35B1	NM_005827 // SLC35B1 // solute carrier family 35, member B1 // 17q21.33 // 10237 /// EN	0.000761388	-0.26312
7957052	NM_006431	CCT2	NM_006431 // CCT2 // chaperonin containing TCP1, subunit 2 (beta) // 12q15 // 10576 ///	0.000675562	-0.262454
8166469	NR_027783	SAT1	NR_027783 // SAT1 // spermidine/spermine N1-acetyltransferase 1 // Xp22.1 // 6303 /// N	2.25E-05	-0.323352
8026106	NM_004343	CALR	NM_004343 // CALR // calreticulin // 19p13.3-p13.2 // 811 /// ENST00000316448 // CALR /	0.00527457	-0.215417

7944525	---		---	0.00709555	0.199627
7946983	NM_030754	SAA2	NM_030754 // SAA2 // serum amyloid A2 // 11p15.1-p14 // 6289 /// BC020795 // SAA2 // se	0.0015283	-0.241146
8041149	NM_015131	WDR43	NM_015131 // WDR43 // WD repeat domain 43 // 2p23.2 // 23160 /// ENST00000407426 // WDR	3.95E-06	-0.337441
8146921	NM_172037	RDH10	NM_172037 // RDH10 // retinol dehydrogenase 10 (all-trans) // 8q21.11 // 157506 /// ENS	4.39E-11	-0.471178
7971813	NM_018676	THSD1	NM_018676 // THSD1 // thrombospondin, type I, domain containing 1 // 13q14.3 // 55901 /	0.00207508	0.236745
8137252	NM_130759	GIMAP1	NM_130759 // GIMAP1 // GTPase, IMAP family member 1 // 7q36.1 // 170575 /// ENST0000030	3.19E-06	0.349546
7961829	NM_001178094	BCAT1	NM_001178094 // BCAT1 // branched chain amino-acid transaminase 1, cytosolic // 12p12.1	0.001383	-0.238732
8133590	NR_003664	SPDYE8P	NR_003664 // SPDYE8P // speedy homolog E8 (Xenopus laevis), pseudogene // 7q11.23 // 38	0.000385341	0.268627
8133600	NR_003664	SPDYE8P	NR_003664 // SPDYE8P // speedy homolog E8 (Xenopus laevis), pseudogene // 7q11.23 // 38	0.000229349	0.277875
7898585	NM_182744	NBL1	NM_182744 // NBL1 // neuroblastoma, suppression of tumorigenicity 1 // 1p36.13 // 4681	0.00509918	0.220499
7950654	NM_024079	ALG8	NM_024079 // ALG8 // asparagine-linked glycosylation 8, alpha-1,3-glucosyltransferase h	0.00959566	-0.203058
8088634	---		---	0.00237358	0.23248
8008517	NM_198175	NME1	NM_198175 // NME1 // non-metastatic cells 1, protein (NM23A) expressed in // 17q21.3 //	1.05E-05	-0.33331
7962146	NM_001135811	FAM60A	NM_001135811 // FAM60A // family with sequence similarity 60, member A // 12p11 // 5851	0.000294837	-0.27518
7929550	NM_001134375	CCNJ	NM_001134375 // CCNJ // cyclin J // 10pter-q26.12 // 54619 /// NM_019084 // CCNJ // cyc	0.00864439	-0.203885
8089954	NM_001023570	IQCB1	NM_001023570 // IQCB1 // IQ motif containing B1 // 3q13.33|3q21.1 // 9657 /// NM_001023	0.00340528	-0.22597
8103644	---		---	0.00401602	0.220527

8086538	BC047037	LOC644714	BC047037 // LOC644714 // hypothetical LOC644714 // 3p21.31 // 644714	0.00276415	0.234007
8026954	NM_005027	PIK3R2	NM_005027 // PIK3R2 // phosphoinositide-3-kinase, regulatory subunit 2 (beta) // 19q13.	0.000872488	-0.255756
7929282	NM_002729	HHEX	NM_002729 // HHEX // hematopoietically expressed homeobox // 10q23.33 // 3087 /// ENST0	1.82E-05	0.319332
8017098	---		---	0.000455237	0.272712
7914648	NM_198040	PHC2	NM_198040 // PHC2 // polyhomeotic homolog 2 (Drosophila) // 1p34.3 // 1912 /// NM_00442	2.18E-05	-0.32821
7978132	NM_024658	IPO4	NM_024658 // IPO4 // importin 4 // 14q12 // 79711 /// ENST00000354464 // IPO4 // import	7.33E-07	-0.367868
8003204	NM_016095	GINS2	NM_016095 // GINS2 // GINS complex subunit 2 (Psf2 homolog) // 16q24.1 // 51659 /// ENS	0.00942767	-0.200749
8043502	---		---	5.85E-06	0.34305
8019804	NR_033770	ROCK1P1	NR_033770 // ROCK1P1 // Rho-associated, coiled-coil containing protein kinase 1 pseudog	0.000195072	0.285938
8005134	NM_000676	ADORA2B	NM_000676 // ADORA2B // adenosine A2b receptor // 17p12 // 136 /// ENST00000304222 // A	0.00725013	-0.208799
8106730	NM_022550	XRCC4	NM_022550 // XRCC4 // X-ray repair complementing defective repair in Chinese hamster ce	0.00965408	-0.196537
7988342	---		---	0.000267391	0.27723
8015037	ENST0000034851:	SMARCE1	ENST00000348513 // SMARCE1 // SWI/SNF related, matrix associated, actin dependent regul	0.00977339	0.203769
8143597	ENST0000047987(	FAM115A	ENST00000479870 // FAM115A // family with sequence similarity 115, member A // 7q35 //	0.0097193	0.201608
8142307	NM_015723	PNPLA8	NM_015723 // PNPLA8 // patatin-like phospholipase domain containing 8 // 7q31 // 50640	0.00102375	-0.254012
7900201	NM_016037	UTP11L	NM_016037 // UTP11L // UTP11-like, U3 small nucleolar ribonucleoprotein, (yeast) // 1p3	3.66E-05	-0.319598

7907702	NM_003101	SOAT1	NM_003101 // SOAT1 // sterol O-acyltransferase 1 // 1q25 // 6646 /// ENST00000367619 //	2.27E-06	-0.348724
8100001	---		---	0.00614391	0.209654
8103922	NM_004346	CASP3	NM_004346 // CASP3 // caspase 3, apoptosis-related cysteine peptidase // 4q34 // 836 //	0.000700943	-0.256403
8022927	NM_012319	SLC39A6	NM_012319 // SLC39A6 // solute carrier family 39 (zinc transporter), member 6 // 18q12.	0.00337653	-0.223187
7998405	NR_027242	LOC146336	NR_027242 // LOC146336 // hypothetical LOC146336 // 16p13.3 // 146336	0.00380747	0.228819
8175288	NM_019556	MOSPD1	NM_019556 // MOSPD1 // motile sperm domain containing 1 // Xq26.3 // 56180 /// ENST0000	0.000413944	-0.264936
8169984	NM_000194	HPRT1	NM_000194 // HPRT1 // hypoxanthine phosphoribosyltransferase 1 // Xq26.1 // 3251 /// EN	8.12E-05	-0.299448
8129482	NM_001017373	SAMD3	NM_001017373 // SAMD3 // sterile alpha motif domain containing 3 // 6q23.1 // 154075 //	0.00183402	0.241734
7908766	NM_006335	TIMM17A	NM_006335 // TIMM17A // translocase of inner mitochondrial membrane 17 homolog A (yeast	0.00232941	-0.237525
7978553	NM_138288	C14orf147	NM_138288 // C14orf147 // chromosome 14 open reading frame 147 // 14q13.1 // 171546 ///	0.00387455	-0.225895
7995895	NM_014685	HERPUD1	NM_014685 // HERPUD1 // homocysteine-inducible, endoplasmic reticulum stress-inducible,	0.00265245	-0.235334
8057677	NM_014585	SLC40A1	NM_014585 // SLC40A1 // solute carrier family 40 (iron-regulated transporter), member 1	0.000192631	0.288144
8023605	NM_176787	PIGN	NM_176787 // PIGN // phosphatidylinositol glycan anchor biosynthesis, class N // 18q21.	0.00782917	-0.207221
7904429	NR_033781	HSD3BP4	NR_033781 // HSD3BP4 // hydroxy-delta-5-steroid dehydrogenase, 3 beta, pseudogene 4 //	1.46E-06	0.360354
7944365	NM_198489	CCDC84	NM_198489 // CCDC84 // coiled-coil domain containing 84 // 11q23.3 // 338657 /// ENST00	0.00386379	0.223858

8156571	NR_029665	MIR27B	NR_029665 // MIR27B // microRNA 27b // 9q22.32 // 407019 /// AF043897 // C9orf3 // chro	1.82E-05	0.320289
8100292	NM_015030	FRYL	NM_015030 // FRYL // FRY-like // 4p11 // 285527 /// ENST00000358350 // FRYL // FRY-like	0.0028431	0.227342
8146500	NM_002350	LYN	NM_002350 // LYN // v-yes-1 Yamaguchi sarcoma viral related oncogene homolog // 8q13 //	7.54E-05	-0.300411
8122336	NM_021243	C6orf115	NM_021243 // C6orf115 // chromosome 6 open reading frame 115 // 6q24.1 // 58527 /// ENS	0.000625569	-0.254775
7925413	AF333388	MT1P2	AF333388 // MT1P2 // metallothionein 1 pseudogene 2 // 1q43 // 645745	4.25E-05	-0.317008
7943193	---		---	0.00695494	0.208193
7909628	NM_014053	FLVCR1	NM_014053 // FLVCR1 // feline leukemia virus subgroup C cellular receptor 1 // 1q32.3 /	0.000874382	-0.255279
7975459	NM_015556	SIPA1L1	NM_015556 // SIPA1L1 // signal-induced proliferation-associated 1 like 1 // 14q24.2 //	0.00609207	-0.211942
7987145	NM_001103184	FMN1	NM_001103184 // FMN1 // formin 1 // 15q13.3 // 342184 /// ENST00000334528 // FMN1 // fo	0.000840441	-0.260489
8089082	NM_080927	DCBLD2	NM_080927 // DCBLD2 // discoidin, CUB and LCCL domain containing 2 // 3q12.1|3 // 13156	0.000470103	-0.266215
7958989	NM_173542	PLBD2	NM_173542 // PLBD2 // phospholipase B domain containing 2 // 12q24.13 // 196463 /// NM_	0.00311536	-0.223395
7945260	---		---	0.00795434	0.207191
7930008	NM_004741	NOLC1	NM_004741 // NOLC1 // nucleolar and coiled-body phosphoprotein 1 // 10q24.32 // 9221 //	1.73E-06	-0.354256
7925743	NM_001004692	OR2T12	NM_001004692 // OR2T12 // olfactory receptor, family 2, subfamily T, member 12 // 1q44	0.00147455	0.245358
7942267	NM_001012710	KRTAP5-10	NM_001012710 // KRTAP5-10 // keratin associated protein 5-10 // 11q13.4 // 387273 /// E	0.00862508	0.200403
8057990	NM_001195144	ANKRD44	NM_001195144 // ANKRD44 // ankyrin repeat domain 44 // 2q33.1 // 91526 /// NM_153697 //	0.00369079	0.22554
7937876	---		---	0.00731978	0.202115

8059532	NM_020161	C2orf83	NM_020161 // C2orf83 // chromosome 2 open reading frame 83 // 2q36.3 // 56918 /// NM_00	0.000142303	0.285609
7913237	NM_018584	CAMK2N1	NM_018584 // CAMK2N1 // calcium/calmodulin-dependent protein kinase II inhibitor 1 // 1	0.000275973	0.279357
8069178	NR_027673	ADARB1	NR_027673 // ADARB1 // adenosine deaminase, RNA-specific, B1 // 21q22.3 // 104 /// NM_0	0.00526298	0.212872
7995825	NM_005949	MT1F	NM_005949 // MT1F // metallothionein 1F // 16q13 // 4494 /// ENST00000334350 // MT1F //	2.06E-05	-0.325397
8146930	NM_017866	TMEM70	NM_017866 // TMEM70 // transmembrane protein 70 // 8q21.11 // 54968 /// NM_001040613 //	4.08E-05	-0.311413
7995813	NR_027781	MT1DP	NR_027781 // MT1DP // metallothionein 1D (pseudogene) // 16q13 // 326343 /// NR_003658	7.52E-06	-0.343148
8107321	NR_015370	NCRNA00219	NR_015370 // NCRNA00219 // non-protein coding RNA 219 // 5q22.2 // 114915 /// AB048207	0.00615015	0.213551
8088958	NM_000158	GBE1	NM_000158 // GBE1 // glucan (1,4-alpha-), branching enzyme 1 // 3p12.3 // 2632 /// ENST	0.0076623	-0.204529
8071061	AK097082	psiTPTE22	AK097082 // psiTPTE22 // TPTE pseudogene // 22q11.1 // 387590	0.0019057	0.240175
7945204	NM_021978	ST14	NM_021978 // ST14 // suppression of tumorigenicity 14 (colon carcinoma) // 11q24-q25 //	0.000626094	-0.260377
8084794	NM_002182	IL1RAP	NM_002182 // IL1RAP // interleukin 1 receptor accessory protein // 3q28 // 3556 /// NM_	1.14E-05	-0.328796
8037835	NM_005628	SLC1A5	NM_005628 // SLC1A5 // solute carrier family 1 (neutral amino acid transporter), member	0.000100926	-0.29611
8064879	NM_015939	TRMT6	NM_015939 // TRMT6 // tRNA methyltransferase 6 homolog (S. cerevisiae) // 20p12.3 // 51	0.00166844	-0.245452
7954055	NM_001130415	APOLD1	NM_001130415 // APOLD1 // apolipoprotein L domain containing 1 // 12p13.1 // 81575 ///	0.000786242	-0.261477

8122440	NM_032860	LTV1	NM_032860 // LTV1 // LTV1 homolog (S. cerevisiae) // 6q24.2 // 84946 /// ENST0000036757	0.0102198	-0.197817
8149248	---		---	0.00197804	0.234016
7970999	NM_015087	SPG20	NM_015087 // SPG20 // spastic paraplegia 20 (Troyer syndrome) // 13q13.3 // 23111 /// N	0.00469647	-0.216147
8121588	NM_013352	DSE	NM_013352 // DSE // dermatan sulfate epimerase // 6q22 // 29940 /// NM_001080976 // DSE	0.000309865	-0.279032
7919390	---		---	6.74E-05	0.304485
7971690	---		---	0.0071418	0.204074
7941621	NM_005700	DPP3	NM_005700 // DPP3 // dipeptidyl-peptidase 3 // 11q12-q13.1 // 10072 /// NM_130443 // DP	2.04E-05	-0.319376
8137483	AK131514	FLJ16734	AK131514 // FLJ16734 // hypothetical LOC641928 // 7q36.2 // 641928	0.00644242	0.208068
7906995	NM_012474	UCK2	NM_012474 // UCK2 // uridine-cytidine kinase 2 // 1q23 // 7371 /// ENST00000367879 // U	0.0028556	-0.228379
8120215	NM_133367	PAQR8	NM_133367 // PAQR8 // progestin and adipoQ receptor family member VIII // 6p12.1 // 853	0.00808048	0.204883
7966026	NM_014840	NUAK1	NM_014840 // NUAK1 // NUAK family, SNF1-like kinase, 1 // 12q23.3 // 9891 /// ENST00000	0.000405701	0.271975
8075401	NM_004861	GAL3ST1	NM_004861 // GAL3ST1 // galactose-3-O-sulfotransferase 1 // 22q12.2 // 9514 /// ENST000	0.000574894	0.268136
8165705	ENST0000036168:	ND6	ENST00000361681 // ND6 // NADH dehydrogenase, subunit 6 (complex I) // --- // 4541	0.000860881	0.246751
7982829	NM_181642	SPINT1	NM_181642 // SPINT1 // serine peptidase inhibitor, Kunitz type 1 // 15q15.1 // 6692 ///	0.00276217	-0.229109
7972674	NM_138779	C13orf27	NM_138779 // C13orf27 // chromosome 13 open reading frame 27 // 13q33.1 // 93081 /// EN	0.00684896	-0.209824
7899265	NM_006142	SFN	NM_006142 // SFN // stratifin // 1p36.11 // 2810 /// ENST00000339276 // SFN // stratifi	3.73E-05	-0.311197

8061186	NM_006363	SEC23B	NM_006363 // SEC23B // Sec23 homolog B (S. cerevisiae) // 20p11.23 // 10483 /// NM_0329	4.09E-07	-0.382556
8044643	NR_026821	FAM138B	NR_026821 // FAM138B // family with sequence similarity 138, member B // 2q14.1 // 6544	6.16E-06	0.341366
8015236	NM_032524	KRTAP4-4	NM_032524 // KRTAP4-4 // keratin associated protein 4-4 // 17q12-q21 // 84616 /// ENST0	0.00212263	0.233915
8113234	NM_000439	PCSK1	NM_000439 // PCSK1 // proprotein convertase subtilisin/kexin type 1 // 5q15-q21 // 5122	0.000227305	-0.283358
8052908	NM_173535	CLEC4F	NM_173535 // CLEC4F // C-type lectin domain family 4, member F // 2p13.3 // 165530 ///	0.000710859	0.255099
8126524	NM_199184	C6orf108	NM_199184 // C6orf108 // chromosome 6 open reading frame 108 // 6p21.1 // 10591 /// NM_	0.00418035	-0.221972
8166219	NM_032796	SYAP1	NM_032796 // SYAP1 // synapse associated protein 1 // Xp22.2 // 94056 /// NR_033181 //	0.000151651	-0.295389
7931519	NM_152643	KNDC1	NM_152643 // KNDC1 // kinase non-catalytic C-lobe domain (KIND) containing 1 // 10q26.3	0.00730803	-0.207645
7951565	NM_020809	ARHGAP20	NM_020809 // ARHGAP20 // Rho GTPase activating protein 20 // 11q23.1 // 57569 /// ENST0	9.65E-05	0.301332
7952404	NM_012378	OR8B8	NM_012378 // OR8B8 // olfactory receptor, family 8, subfamily B, member 8 // 11q24.2 //	0.00786523	0.205262
8085628	NM_015199	ANKRD28	NM_015199 // ANKRD28 // ankyrin repeat domain 28 // 3p25.1 // 23243 /// NM_001195098 //	0.000443417	-0.271209
7983910	NM_020980	AQP9	NM_020980 // AQP9 // aquaporin 9 // 15q // 366 /// ENST00000219919 // AQP9 // aquaporin	0.000390603	-0.264889
7970793	NM_181785	SLC46A3	NM_181785 // SLC46A3 // solute carrier family 46, member 3 // 13q12.3 // 283537 /// NM_	0.00105111	-0.247409
8163255	---		---	4.34E-05	0.312536
8157231	---		---	0.0018675	0.232994
7922689	NM_002065	GLUL	NM_002065 // GLUL // glutamate-ammonia ligase // 1q31 // 2752 /// NM_001033044 // GLUL	0.00454375	-0.217967

8100382	NM_012110	CHIC2	NM_012110 // CHIC2 // cysteine-rich hydrophobic domain 2 // 4q11 // 26511 /// ENST00000	0.00319789	-0.227324
8021169	NM_006033	LIPG	NM_006033 // LIPG // lipase, endothelial // 18q21.1 // 9388 /// ENST00000261292 // LIPG	0.000263101	-0.277853
8104449	NM_012073	CCT5	NM_012073 // CCT5 // chaperonin containing TCP1, subunit 5 (epsilon) // 5p15.2 // 22948	4.04E-07	-0.385314
7954388	NM_016072	GOLT1B	NM_016072 // GOLT1B // golgi transport 1B // 12p12.1 // 51026 /// ENST00000229314 // GO	0.000148379	-0.284021
8045946	NM_005805	PSMD14	NM_005805 // PSMD14 // proteasome (prosome, macropain) 26S subunit, non-ATPase, 14 // 2	0.00024101	-0.285003
7919578	AK299482	GSTM2	AK299482 // GSTM2 // glutathione S-transferase mu 2 (muscle) // 1p13.3 // 2946 /// AK29	0.00169655	0.24115
7939507	NM_032592	ACCS	NM_032592 // ACCS // 1-aminocyclopropane-1-carboxylate synthase homolog (Arabidopsis)(n	0.00781991	0.205701
8123148	NM_014161	MRPL18	NM_014161 // MRPL18 // mitochondrial ribosomal protein L18 // 6q25.3 // 29074 /// ENST0	0.00595131	-0.215984
7927146	NM_018590	CSGALNACT2	NM_018590 // CSGALNACT2 // chondroitin sulfate N-acetylgalactosaminyltransferase 2 // 1	0.00572613	-0.215391
8127841	NM_015599	PGM3	NM_015599 // PGM3 // phosphoglucomutase 3 // 6q14.1-q15 // 5238 /// ENST00000513973 //	0.00649692	-0.210601
8121510	NM_032194	RPF2	NM_032194 // RPF2 // ribosome production factor 2 homolog (S. cerevisiae) // 6q21 // 84	0.0049449	-0.216436
7970655	NM_004685	MTMR6	NM_004685 // MTMR6 // myotubularin related protein 6 // 13q12 // 9107 /// ENST000003818	2.02E-06	-0.36002
7995806	NM_005946	MT1A	NM_005946 // MT1A // metallothionein 1A // 16q13 // 4489 /// ENST00000443255 // MT1A //	3.24E-06	-0.355469
8078153	---		---	0.00807216	0.19707
8143441	NM_001080392	KIAA1147	NM_001080392 // KIAA1147 // KIAA1147 // 7q34 // 57189 /// ENST00000297761 // KIAA1147 /	0.000170126	0.285039

8160441	NM_058197	CDKN2A	NM_058197 // CDKN2A // cyclin-dependent kinase inhibitor 2A (melanoma, p16, inhibits CD	0.00309854	-0.22905
8151549	NM_005536	IMPA1	NM_005536 // IMPA1 // inositol(myo)-1(or 4)-monophosphatase 1 // 8q21.13-q21.3 // 3612	0.000689353	-0.250244
8113305	NM_001270	CHD1	NM_001270 // CHD1 // chromodomain helicase DNA binding protein 1 // 5q15-q21 // 1105 //	0.000853525	-0.254056
8091537	NM_178822	IGSF10	NM_178822 // IGSF10 // immunoglobulin superfamily, member 10 // 3q25.1 // 285313 /// NM	1.59E-05	0.329923
7904572	---		---	0.000225257	0.283779
7987315	NM_005159	ACTC1	NM_005159 // ACTC1 // actin, alpha, cardiac muscle 1 // 15q11-q14 // 70 /// ENST0000029	0.00491249	-0.217852
8100308	---		---	0.000396425	0.273107
8135181	NM_152892	LRWD1	NM_152892 // LRWD1 // leucine-rich repeats and WD repeat domain containing 1 // 7q22.1	0.000523837	-0.264645
8151627	---		---	0.000432728	0.269035
7961757	NM_003034	ST8SIA1	NM_003034 // ST8SIA1 // ST8 alpha-N-acetyl-neuraminide alpha-2,8-sialyltransferase 1 //	7.70E-06	0.337665
7940182	NM_001004705	OR4D10	NM_001004705 // OR4D10 // olfactory receptor, family 4, subfamily D, member 10 // 11q12	0.000870864	0.261054
8130993	NM_020223	FAM20C	NM_020223 // FAM20C // family with sequence similarity 20, member C // 7p22.3 // 56975	1.26E-05	-0.333976
8013307	NR_026718	FOXO3B	NR_026718 // FOXO3B // forkhead box O3B pseudogene // 17p11 // 2310 /// NM_001455 // FO	0.00418823	-0.225535
8072461	NM_016733	LIMK2	NM_016733 // LIMK2 // LIM domain kinase 2 // 22q12.2 // 3985 /// NM_001031801 // LIMK2	3.44E-06	-0.352067
8058052	NM_002156	HSPD1	NM_002156 // HSPD1 // heat shock 60kDa protein 1 (chaperonin) // 2q33.1 // 3329 /// NM_	7.75E-05	-0.30196
7991581	NM_014918	CHSY1	NM_014918 // CHSY1 // chondroitin sulfate synthase 1 // 15q26.3 // 22856 /// ENST000002	3.74E-06	-0.347042
8021685	NM_001093729	CCDC102B	NM_001093729 // CCDC102B // coiled-coil domain containing 102B // 18q22.1 // 79839 ///	0.00401742	0.223744

8040655	NM_033505	EPT1	NM_033505 // EPT1 // ethanolaminephosphotransferase 1 (CDP-ethanolamine-specific) // 2p	1.85E-06	-0.360663
7992887	AK093979	ZNF75A	AK093979 // ZNF75A // zinc finger protein 75a // 16p13.11 // 7627 /// BC150306 // LOC10	0.00877218	0.205133
8166784	NM_004615	TSPAN7	NM_004615 // TSPAN7 // tetraspanin 7 // Xp11.4 // 7102 /// ENST00000378482 // TSPAN7 //	1.78E-05	0.325402
8043909	NM_002518	NPAS2	NM_002518 // NPAS2 // neuronal PAS domain protein 2 // 2q11.2 // 4862 /// ENST000003356	4.72E-05	-0.306878
8107330	NM_001127510	APC	NM_001127510 // APC // adenomatous polyposis coli // 5q21-q22 // 324 /// NM_000038 // A	0.00536551	0.213369
8114287	NM_004598	SPOCK1	NM_004598 // SPOCK1 // sparc/osteonectin, cwcv and kazal-like domains proteoglycan (tes	0.00103107	-0.253171
8142401	---		---	0.000504225	0.25967
8174474	NM_022977	ACSL4	NM_022977 // ACSL4 // acyl-CoA synthetase long-chain family member 4 // Xq22.3-q23 // 2	9.54E-05	-0.292549
8068202	NM_058187	C21orf63	NM_058187 // C21orf63 // chromosome 21 open reading frame 63 // 21q22.11 // 59271 /// E	0.00328426	-0.220932
8115814	NM_001017995	SH3PXD2B	NM_001017995 // SH3PXD2B // SH3 and PX domains 2B // 5q35.1 // 285590 /// ENST000003116	6.54E-07	-0.377522
8117368	NM_003542	HIST1H4C	NM_003542 // HIST1H4C // histone cluster 1, H4c // 6p21.3 // 8364 /// BC130558 // HIST1	0.000371476	0.27487
8072009	NR_033733	CRYBB2P1	NR_033733 // CRYBB2P1 // crystallin, beta B2 pseudogene 1 // 22q11.2-q12.1 // 1416 ///	0.00361807	0.223277
7923347	NM_005558	LAD1	NM_005558 // LAD1 // ladinin 1 // 1q25.1-q32.3 // 3898 /// ENST00000391967 // LAD1 // l	8.07E-05	-0.296163
8175098	NM_178471	GPR119	NM_178471 // GPR119 // G protein-coupled receptor 119 // Xq26.1 // 139760 /// ENST00000	0.00753636	0.210596
7951652	---		---	0.00318859	0.23238
8168968	NM_014710	GPRASP1	NM_014710 // GPRASP1 // G protein-coupled receptor associated sorting protein 1 // Xq22	0.00411963	0.222972

7902290	NM_001902	CTH	NM_001902 // CTH // cystathionase (cystathionine gamma-lyase) // 1p31.1 // 1491 /// NM_	0.00876357	-0.205789
8058552	NM_005896	IDH1	NM_005896 // IDH1 // isocitrate dehydrogenase 1 (NADP+), soluble // 2q33.3 // 3417 ///	0.00905622	-0.197986
8054766	NM_001097	ACR	NM_001097 // ACR // acrosin // 22q13-qter|22q13.33 // 49 /// ENST00000216139 // ACR //	0.000104377	0.292278
8075182	NM_005080	XBP1	NM_005080 // XBP1 // X-box binding protein 1 // 22q12.1|22q12 // 7494 /// NM_001079539	1.91E-05	-0.321907
8162531	---		---	2.96E-07	-0.38754
8092067	NM_001099645	RPL22L1	NM_001099645 // RPL22L1 // ribosomal protein L22-like 1 // 3q26.2 // 200916 /// ENST000	0.00139971	-0.247344
8042830	NR_027405	MTHFD2	NR_027405 // MTHFD2 // methylenetetrahydrofolate dehydrogenase (NADP+ dependent) 2, met	3.56E-08	-0.408244
7967452	---		---	0.0026743	0.231489
8034974	NM_024794	EPHX3	NM_024794 // EPHX3 // epoxide hydrolase 3 // 19p13.12 // 79852 /// NM_001142886 // EPHX	0.00044705	-0.269529
7944970	---		---	0.000226935	0.277827
7999614	NM_001009944	PKD1	NM_001009944 // PKD1 // polycystic kidney disease 1 (autosomal dominant) // 16p13.3 //	0.00782074	0.198055
8016414	NM_003726	SKAP1	NM_003726 // SKAP1 // src kinase associated phosphoprotein 1 // 17q21.32 // 8631 /// NM	0.00330981	0.23027
7951032	NR_003026	SNORA1	NR_003026 // SNORA1 // small nucleolar RNA, H/ACA box 1 // 11q21 // 677792	0.00452419	-0.218427
8147785	NM_015420	DCAF13	NM_015420 // DCAF13 // DDB1 and CUL4 associated factor 13 // 8q22.3 // 25879 /// ENST00	0.00161481	-0.243109
8148501	NM_032611	PTP4A3	NM_032611 // PTP4A3 // protein tyrosine phosphatase type IVA, member 3 // 8q24.3 // 111	0.00131694	-0.250377
7930559	---		---	0.00227681	0.231926
8151615	---		---	0.000459923	0.267635

8152512	NM_002546	TNFRSF11B	NM_002546 // TNFRSF11B // tumor necrosis factor receptor superfamily, member 11b // 8q2	0.000504473	-0.266053
8033754	NM_006631	ZNF266	NM_006631 // ZNF266 // zinc finger protein 266 // 19p13.2 // 10781 /// NM_198058 // ZNF	0.0061244	0.21631
8157092	NM_018112	TMEM38B	NM_018112 // TMEM38B // transmembrane protein 38B // 9q31.2 // 55151 /// ENST0000037469	1.02E-05	-0.331197
7991386	NM_006384	CIB1	NM_006384 // CIB1 // calcium and integrin binding 1 (calmyrin) // 15q25.3-q26 // 10519	0.00645547	-0.214665
7976571	NM_016472	C14orf129	NM_016472 // C14orf129 // chromosome 14 open reading frame 129 // 14q32.2 // 51527 ///	0.00491341	-0.212259
8144699	---		---	0.00128373	0.24481
8090364	AK093796	ZXDC	AK093796 // ZXDC // ZXD family zinc finger C // 3q21.3 // 79364	0.0015079	0.241508
8157270	NM_001859	SLC31A1	NM_001859 // SLC31A1 // solute carrier family 31 (copper transporters), member 1 // 9q3	0.00028309	-0.260816
8053731	AK095105	FLJ37786	AK095105 // FLJ37786 // hypothetical LOC642691 // 2p11.1 // 642691	0.000175684	0.288898
7937104	AK097584	LOC100128830	AK097584 // LOC100128830 // hypothetical protein LOC100128830 // 10q26.3 // 100128830	0.00432479	0.223246
7910416	NM_014777	URB2	NM_014777 // URB2 // URB2 ribosome biogenesis 2 homolog (S. cerevisiae) // 1q42.13 // 9	8.40E-07	-0.371917
7997633	NM_005153	USP10	NM_005153 // USP10 // ubiquitin specific peptidase 10 // 16q24.1 // 9100 /// ENST000002	9.47E-06	-0.332542
7962375	NM_153026	PRICKLE1	NM_153026 // PRICKLE1 // prickle homolog 1 (Drosophila) // 12q12 // 144165 /// NM_00114	2.98E-05	0.318209
7928491	NM_012330	MYST4	NM_012330 // MYST4 // MYST histone acetyltransferase (monocytic leukemia) 4 // 10q22.2	0.00175819	0.24415
8176730	NM_001039567	RPS4Y2	NM_001039567 // RPS4Y2 // ribosomal protein S4, Y-linked 2 // Yq11.223 // 140032 /// EN	0.0101125	-0.192003
8139125	BC030554	TARP	BC030554 // TARP // TCR gamma alternate reading frame protein // 7p15-p14 // 445347 ///	0.00604614	0.213373

7982267	---		---	0.00718077	0.20774
8101881	NM_000668	ADH1B	NM_000668 // ADH1B // alcohol dehydrogenase 1B (class I), beta polypeptide // 4q23 // 1	3.67E-05	0.318903
8100782	---		---	0.00665386	0.212294
8095005	---		---	0.000384191	0.269905
7942274	ENST0000032933(	OR7E87P	ENST00000329336 // OR7E87P // olfactory receptor, family 7, subfamily E, member 87 pseu	0.00337198	0.217438
8133721	NM_001540	HSPB1	NM_001540 // HSPB1 // heat shock 27kDa protein 1 // 7q11.23 // 3315 /// ENST00000248553	0.00279501	-0.231152
8150862	---		---	0.00310581	-0.228026
8021365	NM_005603	ATP8B1	NM_005603 // ATP8B1 // ATPase, aminophospholipid transporter, class I, type 8B, member	0.00174337	0.239224
7917779	NM_002061	GCLM	NM_002061 // GCLM // glutamate-cysteine ligase, modifier subunit // 1p22.1 // 2730 ///	8.53E-05	-0.299728
8002403	NM_138383	MTSS1L	NM_138383 // MTSS1L // metastasis suppressor 1-like // 16q22.1 // 92154 /// ENST0000033	0.00488527	-0.219698
8072015	NM_005160	ADRBK2	NM_005160 // ADRBK2 // adrenergic, beta, receptor kinase 2 // 22q11|22q12.1 // 157 ///	0.00691866	-0.208961
8073194	NM_004810	GRAP2	NM_004810 // GRAP2 // GRB2-related adaptor protein 2 // 22q13.2 // 9402 /// ENST0000034	0.00158375	0.247274
7998637	NM_016332	SEPX1	NM_016332 // SEPX1 // selenoprotein X, 1 // 16p13.3 // 51734 /// ENST00000361871 // SEP	2.51E-06	-0.361474
8160637	NM_001497	B4GALT1	NM_001497 // B4GALT1 // UDP-Gal:betaGlcNAc beta 1,4- galactosyltransferase, polypeptide	5.28E-09	-0.436936
7935627	NM_002079	GOT1	NM_002079 // GOT1 // glutamic-oxaloacetic transaminase 1, soluble (aspartate aminotrans	1.15E-05	-0.333164
7960464	NM_000552	VWF	NM_000552 // VWF // von Willebrand factor // 12p13.3 // 7450 /// ENST00000261405 // VWF	0.00768547	0.20749
7923974	NR_029832	MIR29C	NR_029832 // MIR29C // microRNA 29c // 1q32.2 // 407026	9.55E-06	0.320989
8030391	---		---	0.00188714	0.242535

7934553	NM_032772	ZNF503	NM_032772 // ZNF503 // zinc finger protein 503 // 10q22.2 // 84858 /// ENST00000372524	5.61E-06	0.341903
8019964	NM_001010000	ARHGAP28	NM_001010000 // ARHGAP28 // Rho GTPase activating protein 28 // 18p11.31 // 79822 /// E	0.00615083	0.215296
7909455	NM_025228	TRAF3IP3	NM_025228 // TRAF3IP3 // TRAF3 interacting protein 3 // 1q32 // 80342 /// ENST000003670	0.00323541	0.229685
7947270	NM_002233	KCNA4	NM_002233 // KCNA4 // potassium voltage-gated channel, shaker-related subfamily, member	0.000556425	0.262159
7917674	NM_001122821	SET	NM_001122821 // SET // SET nuclear oncogene // 9q34 // 6418 /// NM_003011 // SET // SET	0.00127225	-0.250786
8127498	NR_029598	MIR30C2	NR_029598 // MIR30C2 // microRNA 30c-2 // 6q13 // 407032	1.58E-06	0.350419
8069565	NM_001130914	BTG3	NM_001130914 // BTG3 // BTG family, member 3 // 21q21.1 // 10950 /// NM_006806 // BTG3	0.000925889	-0.257729
7906386	NM_152501	PYHIN1	NM_152501 // PYHIN1 // pyrin and HIN domain family, member 1 // 1q23.1 // 149628 /// NM	0.00190865	0.242295
8104788	NM_001145525	RAI14	NM_001145525 // RAI14 // retinoic acid induced 14 // 5p13.3-p13.2 // 26064 /// NM_00114	0.00104106	-0.250186
7966878	NM_007174	CIT	NM_007174 // CIT // citron (rho-interacting, serine/threonine kinase 21) // 12q24 // 11	0.00346894	-0.225907
8057887	NM_004226	STK17B	NM_004226 // STK17B // serine/threonine kinase 17b // 2q32.3 // 9262 /// ENST0000026395	0.00793449	-0.209178
8104680	NM_018356	C5orf22	NM_018356 // C5orf22 // chromosome 5 open reading frame 22 // 5p13.3 // 55322 /// ENST0	0.00263298	-0.232132
8043981	NM_004633	IL1R2	NM_004633 // IL1R2 // interleukin 1 receptor, type II // 2q12 // 7850 /// NM_173343 //	3.18E-09	-0.441552
8030974	NM_001195187	LOC646508	NM_001195187 // LOC646508 // family with sequence similarity 90, member A1-like // 19q1	0.00791239	0.206156
8017927	NM_080283	ABCA9	NM_080283 // ABCA9 // ATP-binding cassette, sub-family A (ABC1), member 9 // 17q24.2 //	0.00113339	0.244006
8034315	NM_001080493	ZNF823	NM_001080493 // ZNF823 // zinc finger protein 823 // 19p13.2 // 55552 /// ENST000003411	0.00272121	0.234532

7963986	NM_002870	RAB13	NM_002870 // RAB13 // RAB13, member RAS oncogene family // 1q21.2 // 5872 /// ENST00000	0.00769333	-0.208318
7969472	ENST0000031824!	LOC729420	ENST00000318245 // LOC729420 // hypothetical LOC729420 // 13q22.2 // 729420 /// AK09269	0.00484897	0.220833
7993369	---		---	0.00209173	0.240911
7903786	NM_000757	CSF1	NM_000757 // CSF1 // colony stimulating factor 1 (macrophage) // 1p21-p13 // 1435 /// N	0.00128994	-0.252136
8017885	NM_007168	ABCA8	NM_007168 // ABCA8 // ATP-binding cassette, sub-family A (ABC1), member 8 // 17q24 // 1	0.00333805	0.225729
8070701	NM_000100	CSTB	NM_000100 // CSTB // cystatin B (stefin B) // 21q22.3 // 1476 /// ENST00000291568 // CS	0.00522176	-0.207625
8100251	NM_015030	FRYL	NM_015030 // FRYL // FRY-like // 4p11 // 285527 /// ENST00000503238 // FRYL // FRY-like	0.0101247	0.200741
8102986	NM_001168235	FREM3	NM_001168235 // FREM3 // FRAS1 related extracellular matrix 3 // 4q31.21 // 166752	0.00221256	0.238164
8091546	NM_001123228	TMEM14E	NM_001123228 // TMEM14E // transmembrane protein 14E // 3q25.1 // 645843 /// ENST000004	0.00126952	0.247993
8051387	NM_032574	DPY30	NM_032574 // DPY30 // dpy-30 homolog (C. elegans) // 2p22.3 // 84661 /// ENST0000029506	0.00189875	-0.244014
8106818	---		---	0.000818921	0.25498
8116760	NM_031480	RIOK1	NM_031480 // RIOK1 // RIO kinase 1 (yeast) // 6p24.3 // 83732 /// NM_153005 // RIOK1 //	0.00110699	-0.249873
7945648	NM_001004325	KRTAP5-2	NM_001004325 // KRTAP5-2 // keratin associated protein 5-2 // 11p15.5 // 440021 /// ENS	0.0033034	0.226691
8038695	NM_139277	KLK7	NM_139277 // KLK7 // kallikrein-related peptidase 7 // 19q13.41 // 5650 /// NM_005046 /	0.00851622	0.207099
7956152	NM_006191	PA2G4	NM_006191 // PA2G4 // proliferation-associated 2G4, 38kDa // 12q13.2 // 5036 /// ENST00	0.0053152	-0.215694
7915015	NM_013285	GNL2	NM_013285 // GNL2 // guanine nucleotide binding protein-like 2 (nucleolar) // 1p34.3 //	2.00E-05	-0.323389
8136938	---		---	0.00409822	0.224643

8143599	---		---	0.00409822	0.224643
8146955	---		---	0.000103199	0.29107
8010963	---		---	0.000317674	0.275623
8151603	---		---	0.000534105	0.26552
8151607	NR_003594	REXO1L2P	NR_003594 // REXO1L2P // REX1, RNA exonuclease 1 homolog (S. cerevisiae)-like 2 (pseudo	0.000534105	0.26552
8151623	NR_003594	REXO1L2P	NR_003594 // REXO1L2P // REX1, RNA exonuclease 1 homolog (S. cerevisiae)-like 2 (pseudo	0.000534105	0.26552
7980535	NM_001037494	DYNLL1	NM_001037494 // DYNLL1 // dynein, light chain, LC8-type 1 // 12q24.23 // 8655 /// NM_00	0.00252485	-0.23384
8152255	NM_030780	SLC25A32	NM_030780 // SLC25A32 // solute carrier family 25, member 32 // 8q22.3 // 81034 /// NM_	0.000264277	-0.280471
8122365	NM_020455	GPR126	NM_020455 // GPR126 // G protein-coupled receptor 126 // 6q24.1 // 57211 /// NM_0010323	0.000502597	0.270435
7908022	NR_033302	DHX9	NR_033302 // DHX9 // DEAH (Asp-Glu-Ala-His) box polypeptide 9 // 1q25 // 1660 /// NM_00	0.00283422	-0.231807
8078971	NM_001248	ENTPD3	NM_001248 // ENTPD3 // ectonucleoside triphosphate diphosphohydrolase 3 // 3p21.3 // 95	0.000215153	-0.284349
7905492	NM_178433	LCE3B	NM_178433 // LCE3B // late cornified envelope 3B // 1q21.3 // 353143 /// ENST0000033563	0.0101103	0.200042
7953749	NM_080387	CLEC4D	NM_080387 // CLEC4D // C-type lectin domain family 4, member D // 12p13.31 // 338339 //	3.21E-06	-0.359111
7924092	NM_021194	SLC30A1	NM_021194 // SLC30A1 // solute carrier family 30 (zinc transporter), member 1 // 1q32.3	0.00847462	-0.206853
7938702	NR_026750	DKFZp686O24166	NR_026750 // DKFZp686O24166 // hypothetical protein DKFZp686O24166 // 11p15.1 // 374383	4.66E-07	-0.371301
8151411	---		---	0.00175883	0.239319
8031984	NR_026818	FAM138A	NR_026818 // FAM138A // family with sequence similarity 138, member A // 1p36.33 // 645	1.81E-05	0.325018
7911323	NR_026818	FAM138A	NR_026818 // FAM138A // family with sequence similarity 138, member A // 1p36.33 // 645	1.81E-05	0.325018

8159803	NR_026818	FAM138A	NR_026818 // FAM138A // family with sequence similarity 138, member A // 1p36.33 // 645	1.98E-05	0.324066
8151619	NR_003594	REXO1L2P	NR_003594 // REXO1L2P // REX1, RNA exonuclease 1 homolog (S. cerevisiae)-like 2 (pseudo	0.000348618	0.27341
7965769	NM_145913	SLC5A8	NM_145913 // SLC5A8 // solute carrier family 5 (iodide transporter), member 8 // 12q23.	0.00392631	-0.220809
8010766	---		---	0.000128984	0.289121
7956281	NM_007264	GPR182	NM_007264 // GPR182 // G protein-coupled receptor 182 // 12q13.3 // 11318 /// ENST00000	0.000393368	0.269168
8166769	NM_000531	OTC	NM_000531 // OTC // ornithine carbamoyltransferase // Xp21.1 // 5009 /// ENST0000003900	0.00320993	0.230464
8163731	---		---	0.00804476	0.206191
7961230	NM_003651	CSDA	NM_003651 // CSDA // cold shock domain protein A // 12p13.1 // 8531 /// NM_001145426 //	5.84E-05	-0.309295
8138862	---		---	0.00357362	0.223871
7921344	NM_012081	ELL2	NM_012081 // ELL2 // elongation factor, RNA polymerase II, 2 // 5q15 // 22936 /// ENST0	4.47E-08	-0.409908
8110106	NM_001131055	HRH2	NM_001131055 // HRH2 // histamine receptor H2 // 5q35.2 // 3274 /// NM_022304 // HRH2 /	0.000519865	-0.269182
7986637	---		---	0.00786556	-0.20682
7940857	NM_006819	STIP1	NM_006819 // STIP1 // stress-induced-phosphoprotein 1 // 11q13 // 10963 /// ENST0000030	0.000900265	-0.256033
7982356	---		---	0.00943567	0.200977
7987046	---		---	0.00943567	0.200977
7987137	---		---	0.00943567	0.200977
7971373	---		---	0.00181383	0.239495
7930915	---		---	0.00806314	0.201139
7955719	NM_001099668	HIGD1A	NM_001099668 // HIGD1A // HIG1 hypoxia inducible domain family, member 1A // 3p22.1 //	0.00773533	-0.208339

7943051	NM_005467	NAALAD2	NM_005467 // NAALAD2 // N-acetylated alpha-linked acidic dipeptidase 2 // 11q14.3-q21 /	3.50E-05	0.31969
8025478	NM_032497	ZNF559	NM_032497 // ZNF559 // zinc finger protein 559 // 19p13.2 // 84527 /// NM_001172650 //	0.00348714	0.224932
8033054	NM_000149	FUT3	NM_000149 // FUT3 // fucosyltransferase 3 (galactoside 3(4)-L-fucosyltransferase, Lewis	0.000488536	-0.271022
8131374	NM_006303	AIMP2	NM_006303 // AIMP2 // aminoacyl tRNA synthetase complex-interacting multifunctional pro	0.00076452	-0.263364
7906017	NR_034180	POU5F1P4	NR_034180 // POU5F1P4 // POU class 5 homeobox 1 pseudogene 4 // 1q22 // 645682 /// GU48	0.0013318	0.249613
8063115	NM_004994	MMP9	NM_004994 // MMP9 // matrix metallopeptidase 9 (gelatinase B, 92kDa gelatinase, 92kDa t	0.00654404	-0.21012
8103094	NM_000901	NR3C2	NM_000901 // NR3C2 // nuclear receptor subfamily 3, group C, member 2 // 4q31.1 // 4306	5.74E-05	0.308037
8143905	NM_007189	ABCF2	NM_007189 // ABCF2 // ATP-binding cassette, sub-family F (GCN20), member 2 // 7q36 // 1	0.000349604	-0.276519
8002143	NM_000229	LCAT	NM_000229 // LCAT // lecithin-cholesterol acyltransferase // 16q22.1 // 3931 /// NM_005	0.00409045	0.214479
8154305	NM_016275	SELT	NM_016275 // SELT // selenoprotein T // 3q25.1 // 51714 /// ENST00000471696 // SELT //	0.00280426	-0.23327
8130422	NM_173515	CNKSR3	NM_173515 // CNKSR3 // CNKSR family member 3 // 6q25.2 // 154043 /// ENST00000367213 //	0.000106199	-0.294292
8084064	NM_006636	MTHFD2	NM_006636 // MTHFD2 // methylenetetrahydrofolate dehydrogenase (NADP+ dependent) 2, met	4.42E-07	-0.382108
7949808	NM_004910	PITPNM1	NM_004910 // PITPNM1 // phosphatidylinositol transfer protein, membrane-associated 1 //	0.0009388	-0.256073
8114193	NM_001033503	SAR1B	NM_001033503 // SAR1B // SAR1 homolog B (S. cerevisiae) // 5q31.1 // 51128 /// NM_01610	0.000199153	-0.284799

7986503	NR_003260	C15orf51	NR_003260 // C15orf51 // dynamin 1 pseudogene // 15q26.3 // 196968 /// AK302717 // C15o	0.000228326	0.279753
7954589	NM_001029874	REP15	NM_001029874 // REP15 // RAB15 effector protein // 12p11.22 // 387849 /// ENST000003107	0.000348493	0.267465
7962479	---		---	0.00631835	0.206382
8041508	NM_012413	QPCT	NM_012413 // QPCT // glutaminyl-peptide cyclotransferase // 2p22.2 // 25797 /// ENST000	0.000963402	-0.246636
8165817	NM_001079855	GYG2	NM_001079855 // GYG2 // glycogenin 2 // Xp22.3 // 8908 /// NM_003918 // GYG2 // glycoge	0.00658375	-0.20824
7987279	NR_027410	GOLGA8B	NR_027410 // GOLGA8B // golgin A8 family, member B // 15q14 // 440270 /// NM_001023567	0.00667752	0.210024
7943827	NM_001931	DLAT	NM_001931 // DLAT // dihydrolipoamide S-acetyltransferase // 11q23.1 // 1737 /// ENST00	0.000136102	-0.293694
7956842	NM_014319	LEMD3	NM_014319 // LEMD3 // LEM domain containing 3 // 12q14 // 23592 /// NM_001167614 // LEM	0.00402821	-0.227115
8036787	NM_003890	FCGBP	NM_003890 // FCGBP // Fc fragment of IgG binding protein // 19q13.1 // 8857 /// ENST000	0.000103138	-0.297402
7938563	NM_001178	ARNTL	NM_001178 // ARNTL // aryl hydrocarbon receptor nuclear translocator-like // 11p15 // 4	0.00908511	-0.206555
8112312	NM_014473	DIMT1L	NM_014473 // DIMT1L // DIM1 dimethyladenosine transferase 1-like (S. cerevisiae) // 5q1	3.17E-05	-0.317173
8125919	NM_001145775	FKBP5	NM_001145775 // FKBP5 // FK506 binding protein 5 // 6p21.31 // 2289 /// NM_004117 // FK	0.000199232	-0.288703
8136115	NM_020704	FAM40B	NM_020704 // FAM40B // family with sequence similarity 40, member B // 7q32.1 // 57464	0.00486602	-0.216519
8017421	NM_020198	CCDC47	NM_020198 // CCDC47 // coiled-coil domain containing 47 // 17q23.3 // 57003 /// ENST000	0.00301967	-0.225451

8151471	NM_014018	MRPS28	NM_014018 // MRPS28 // mitochondrial ribosomal protein S28 // 8q21.1-q21.2 // 28957 ///	0.00984893	-0.203012
7932211	---		---	0.00919405	0.20291
8100154	NM_006587	CORIN	NM_006587 // CORIN // corin, serine peptidase // 4p13-p12 // 10699 /// ENST00000273857	0.00175478	-0.240268
7967898	---		---	0.00016075	0.286696
8028656	NM_022835	PLEKHG2	NM_022835 // PLEKHG2 // pleckstrin homology domain containing, family G (with RhoGef do	0.00114928	-0.248381
8127658	---		---	0.00653393	0.209648
7946977	NM_006512	SAA4	NM_006512 // SAA4 // serum amyloid A4, constitutive // 11p15.1-p14 // 6291 /// ENST0000	0.00229541	-0.232747
8139057	NM_014800	ELMO1	NM_014800 // ELMO1 // engulfment and cell motility 1 // 7p14.1 // 9844 /// NM_001039459	0.00924294	0.202163
7907859	---		---	0.00271297	-0.232389
7969428	NM_006002	UCHL3	NM_006002 // UCHL3 // ubiquitin carboxyl-terminal esterase L3 (ubiquitin thiolesterase)	0.00103839	-0.253345
7970513	NM_145061	SKA3	NM_145061 // SKA3 // spindle and kinetochore associated complex subunit 3 // 13q12.11 /	0.00118329	-0.24624
8135931	NR_024368	FLJ45340	NR_024368 // FLJ45340 // hypothetical LOC402483 // 7q32.1 // 402483 /// ENST00000415418	0.00177563	0.234331
8015685	NM_001991	EZH1	NM_001991 // EZH1 // enhancer of zeste homolog 1 (Drosophila) // 17q21.1-q21.3 // 2145	0.000692048	0.264502
7974363	NM_000953	PTGDR	NM_000953 // PTGDR // prostaglandin D2 receptor (DP) // 14q22.1 // 5729 /// ENST0000030	2.18E-07	0.384405
8123739	NM_016588	NRN1	NM_016588 // NRN1 // neuritin 1 // 6p25.1 // 51299 /// ENST00000244766 // NRN1 // neuri	0.00107298	0.255473
7947245	M30627	HSP90AA2	M30627 // HSP90AA2 // heat shock protein 90kDa alpha (cytosolic), class A member 2 // 1	0.00309376	-0.230228
8101624	NM_020803	KLHL8	NM_020803 // KLHL8 // kelch-like 8 (Drosophila) // 4q22.1 // 57563 /// ENST00000273963	0.0032341	-0.226004

7951246	NM_002424	MMP8	NM_002424 // MMP8 // matrix metallopeptidase 8 (neutrophil collagenase) // 11q22.3 // 4	0.000626004	-0.257549
8117225	NM_015895	GMNN	NM_015895 // GMNN // geminin, DNA replication inhibitor // 6p22.3 // 51053 /// ENST0000	0.0102382	-0.202171
8154153	NM_017913	CDC37L1	NM_017913 // CDC37L1 // cell division cycle 37 homolog (S. cerevisiae)-like 1 // 9p24.1	0.00280595	-0.231444
8065569	NM_138578	BCL2L1	NM_138578 // BCL2L1 // BCL2-like 1 // 20q11.21 // 598 /// NM_001191 // BCL2L1 // BCL2-l	0.0013637	-0.249029
8138977	NM_015283	DPY19L1	NM_015283 // DPY19L1 // dpy-19-like 1 (C. elegans) // 7p14.3-p14.2 // 23333 /// ENST000	0.00427079	-0.221327
8009913	NR_003587	MYO15B	NR_003587 // MYO15B // myosin XVB pseudogene // 17q25.1 // 80022 /// BC027875 // MYO15B	0.00748921	0.203402
7961540	NM_032918	RERG	NM_032918 // RERG // RAS-like, estrogen-regulated, growth inhibitor // 12p12.3 // 85004	0.000223845	0.279506
8069553	NM_003489	NRIP1	NM_003489 // NRIP1 // nuclear receptor interacting protein 1 // 21q11.2 // 8204 /// ENS	0.000537149	-0.265914
7927658	NM_003338	UBE2D1	NM_003338 // UBE2D1 // ubiquitin-conjugating enzyme E2D 1 (UBC4/5 homolog, yeast) // 10	0.000242869	-0.280718
7991512	NR_003260	C15orf51	NR_003260 // C15orf51 // dynamin 1 pseudogene // 15q26.3 // 196968 /// AK302717 // C15o	0.00875593	0.20264
8076515	NM_014570	ARFGAP3	NM_014570 // ARFGAP3 // ADP-ribosylation factor GTPase activating protein 3 // 22q13.2	4.74E-08	-0.408852
8081055	NM_014043	CHMP2B	NM_014043 // CHMP2B // chromatin modifying protein 2B // 3p11.2 // 25978 /// ENST000002	0.000672419	-0.260864
8082250	---		---	0.0100169	0.198483
8009875	NR_003587	MYO15B	NR_003587 // MYO15B // myosin XVB pseudogene // 17q25.1 // 80022 /// AB058686 // MYO15B	0.00959296	0.197859
7974870	NM_003082	SNAPC1	NM_003082 // SNAPC1 // small nuclear RNA activating complex, polypeptide 1, 43kDa // 14	0.000134403	-0.287967

8038824	NM_033130	SIGLEC10	NM_033130 // SIGLEC10 // sialic acid binding Ig-like lectin 10 // 19q13.3 // 89790 ///	0.00479447	-0.219836
8010287	NM_030968	C1QTNF1	NM_030968 // C1QTNF1 // C1q and tumor necrosis factor related protein 1 // 17q25.3 // 1	0.00809678	-0.201807
7938225	NM_198474	OLFML1	NM_198474 // OLFML1 // olfactomedin-like 1 // 11p15.4 // 283298 /// ENST00000329293 //	0.0076108	0.209106
8062927	NM_002638	PI3	NM_002638 // PI3 // peptidase inhibitor 3, skin-derived // 20q13.12 // 5266 /// ENST000	7.97E-05	-0.306287
8145636	NM_024567	HMBOX1	NM_024567 // HMBOX1 // homeobox containing 1 // 8p21.1 // 79618 /// NM_001135726 // HMB	0.00394336	0.223502
8036430	NM_152605	ZNF781	NM_152605 // ZNF781 // zinc finger protein 781 // 19q13.12 // 163115 /// ENST0000035858	0.00727541	0.209948
8129045	NM_001527	HDAC2	NM_001527 // HDAC2 // histone deacetylase 2 // 6q21 // 3066 /// NR_033441 // HDAC2 // h	0.00813336	-0.203721
8103535	NR_026575	GK3P	NR_026575 // GK3P // glycerol kinase 3 pseudogene // 4q32.1 // 2713 /// AK292282 // GK3	0.00269906	-0.233561
8046346	NM_003642	HAT1	NM_003642 // HAT1 // histone acetyltransferase 1 // 2q31.2-q33.1 // 8520 /// NR_027862	0.00248996	-0.232462
7962212	NM_004572	PKP2	NM_004572 // PKP2 // plakophilin 2 // 12p11 // 5318 /// NM_001005242 // PKP2 // plakoph	0.00270681	-0.233694
7901765	NM_015888	HOOK1	NM_015888 // HOOK1 // hook homolog 1 (Drosophila) // 1p32.1 // 51361 /// ENST0000037120	0.000694082	-0.259437
8088535	NM_014814	PSMD6	NM_014814 // PSMD6 // proteasome (prosome, macropain) 26S subunit, non-ATPase, 6 // 3p1	0.00939706	-0.204732
7943160	NR_002569	SCARNA9	NR_002569 // SCARNA9 // small Cajal body-specific RNA 9 // 11q21 // 619383	0.00731863	0.206981
7977933	NM_012244	SLC7A8	NM_012244 // SLC7A8 // solute carrier family 7 (amino acid transporter, L-type), member	0.000912303	-0.249795
8089723	---		---	0.00309184	0.233046

8113220	NM_012081	ELL2	NM_012081 // ELL2 // elongation factor, RNA polymerase II, 2 // 5q15 // 22936 /// ENST0	4.13E-08	-0.410249
7940479	NM_016499	TMEM216	NM_016499 // TMEM216 // transmembrane protein 216 // 11q13.1 // 51259 /// NM_001173990	0.00713186	-0.210343
8144516	NM_153332	ERI1	NM_153332 // ERI1 // exoribonuclease 1 // 8p23.1 // 90459 /// ENST00000250263 // ERI1 /	0.00684853	-0.209474
8129783	NM_003980	MAP7	NM_003980 // MAP7 // microtubule-associated protein 7 // 6q23.3 // 9053 /// NM_00119860	0.00317164	-0.229514
8015796	---		---	0.00592847	0.215626
8166096	NM_152634	TCEANC	NM_152634 // TCEANC // transcription elongation factor A (SII) N-terminal and central d	0.000194672	0.284523
8072582	---		---	0.00481243	0.218869
8144315	BC026965	ARHGEF10	BC026965 // ARHGEF10 // Rho guanine nucleotide exchange factor (GEF) 10 // 8p23 // 9639	0.00521196	0.214541
8090637	---		---	2.75E-05	0.318248
7975224	NM_004094	EIF2S1	NM_004094 // EIF2S1 // eukaryotic translation initiation factor 2, subunit 1 alpha, 35k	0.00118324	-0.245124
8177130	NM_139173	NHEDC1	NM_139173 // NHEDC1 // Na+/H+ exchanger domain containing 1 // 4q24 // 150159 /// NM_00	0.00477129	0.221125
7980940	NR_028459	ATXN3	NR_028459 // ATXN3 // ataxin 3 // 14q21 // 4287 /// NM_004993 // ATXN3 // ataxin 3 // 1	0.000394771	0.272986
8092265	NM_020409	MRPL47	NM_020409 // MRPL47 // mitochondrial ribosomal protein L47 // 3q26.33 // 57129 /// NM_1	0.000494051	-0.267438
8032834	NM_052972	LRG1	NM_052972 // LRG1 // leucine-rich alpha-2-glycoprotein 1 // 19p13.3 // 116844 /// ENST0	0.00408064	-0.225019
8042568	NR_002185	OR7E91P	NR_002185 // OR7E91P // olfactory receptor, family 7, subfamily E, member 91 pseudogene	0.000488507	0.264867
8097570	NM_032557	USP38	NM_032557 // USP38 // ubiquitin specific peptidase 38 // --- // 84640 /// ENST000003070	0.00315194	-0.226939

7939383	NM_024841	PRR5L	NM_024841 // PRR5L // proline rich 5 like // 11p13-p12 // 79899 /// NM_001160167 // PRR	0.0044862	0.220483
8051528	NM_005760	CEBPZ	NM_005760 // CEBPZ // CCAAT/enhancer binding protein (C/EBP), zeta // 2p22.2 // 10153 /	0.0084806	-0.203721
8102792	NM_019035	PCDH18	NM_019035 // PCDH18 // protocadherin 18 // 4q31 // 54510 /// ENST00000344876 // PCDH18	0.0066837	0.213831
8099912	BC008502	C4orf34	BC008502 // C4orf34 // chromosome 4 open reading frame 34 // 4p14 // 201895 /// ENST000	0.00116698	-0.253504
7961595	NM_024730	RERGL	NM_024730 // RERGL // RERG/RAS-like // 12p12.3 // 79785 /// ENST00000229002 // RERGL //	0.00848221	0.206146
8117583	NM_003509	HIST1H2AI	NM_003509 // HIST1H2AI // histone cluster 1, H2ai // 6p22.1 // 8329 /// NM_003536 // HI	0.00566663	-0.214309
7897378	NM_016831	PER3	NM_016831 // PER3 // period homolog 3 (Drosophila) // 1p36.23 // 8863 /// ENST000003619	0.00274908	0.234728
8021181	NR_003003	SCARNA17	NR_003003 // SCARNA17 // small Cajal body-specific RNA 17 // 18q21.1 // 677769	0.00783112	0.203565
8143471	NM_013252	CLEC5A	NM_013252 // CLEC5A // C-type lectin domain family 5, member A // 7q33 // 23601 /// ENS	0.005797	-0.212715
7973221	AK301287	TRAJ17	AK301287 // TRAJ17 // T cell receptor alpha joining 17 // 14q11 // 28738 /// BC035680 /	0.000574922	0.268584
8048733	NM_004457	ACSL3	NM_004457 // ACSL3 // acyl-CoA synthetase long-chain family member 3 // 2q34-q35 // 218	2.00E-05	-0.325861
8083656	NM_022736	MFSD1	NM_022736 // MFSD1 // major facilitator superfamily domain containing 1 // 3q25.32 // 6	0.00592496	-0.212313
7935707	NM_001278	CHUK	NM_001278 // CHUK // conserved helix-loop-helix ubiquitous kinase // 10q24-q25 // 1147	0.000318533	-0.273209
8151032	NM_003878	GGH	NM_003878 // GGH // gamma-glutamyl hydrolase (conjugase, folylpolygammaglutamyl hydrola	0.0017008	-0.245141
7975976	NM_012111	AHSA1	NM_012111 // AHSA1 // AHA1, activator of heat shock 90kDa protein ATPase homolog 1 (yea	0.00397563	-0.228121

7938758	NM_000331	SAA1	NM_000331 // SAA1 // serum amyloid A1 // 11p15.1 // 6288 /// NM_199161 // SAA1 // serum	0.00307997	-0.224844
7988767	NM_031226	CYP19A1	NM_031226 // CYP19A1 // cytochrome P450, family 19, subfamily A, polypeptide 1 // 15q21	0.000386664	-0.27576
7951429	NM_198439	KBTBD3	NM_198439 // KBTBD3 // kelch repeat and BTB (POZ) domain containing 3 // 11q22.3 // 143	0.000521169	0.268271
8104738	NM_006713	SUB1	NM_006713 // SUB1 // SUB1 homolog (S. cerevisiae) // 5p13.3 // 10923 /// ENST0000026507	0.00601389	-0.210324
7994541	NM_014387	LAT	NM_014387 // LAT // linker for activation of T cells // 16p11.2 // 27040 /// NM_0010149	0.0011667	0.253289
8095139	NM_024592	SRD5A3	NM_024592 // SRD5A3 // steroid 5 alpha-reductase 3 // 4q12 // 79644 /// ENST00000264228	4.14E-06	-0.352568
8144717	---		---	0.00990659	0.197465
8129456	---		---	0.00951729	0.202472
7977820	NM_001039619	PRMT5	NM_001039619 // PRMT5 // protein arginine methyltransferase 5 // 14q11.2 // 10419 /// N	0.000643943	-0.26541
8129618	NM_004666	VNN1	NM_004666 // VNN1 // vanin 1 // 6q23-q24 // 8876 /// ENST00000367928 // VNN1 // vanin 1	4.33E-06	-0.351302
7995838	NM_005952	MT1X	NM_005952 // MT1X // metallothionein 1X // 16q13 // 4501 /// ENST00000394485 // MT1X //	5.72E-07	-0.374817
8099326	NM_020041	SLC2A9	NM_020041 // SLC2A9 // solute carrier family 2 (facilitated glucose transporter), membe	0.0039493	-0.219596
8092392	NM_130446	KLHL6	NM_130446 // KLHL6 // kelch-like 6 (Drosophila) // --- // 89857 /// ENST00000341319 //	0.00259459	-0.233505
8156521	NR_029483	MIRLET7F1	NR_029483 // MIRLET7F1 // microRNA let-7f-1 // 9q22.32 // 406888	4.55E-05	0.308047
8065230	NM_006606	RBBP9	NM_006606 // RBBP9 // retinoblastoma binding protein 9 // 20p11.2 // 10741 /// ENST0000	0.00267406	-0.229792
7932985	NM_003873	NRP1	NM_003873 // NRP1 // neuropilin 1 // 10p12 // 8829 /// NM_001024628 // NRP1 // neuropil	0.000163475	0.292028

8093219	NM_203314	BDH1	NM_203314 // BDH1 // 3-hydroxybutyrate dehydrogenase, type 1 // 3q29 // 622 /// NM_0040	0.00761864	-0.20954
8114455	NM_004134	HSPA9	NM_004134 // HSPA9 // heat shock 70kDa protein 9 (mortalin) // 5q31.1 // 3313 /// ENST0	1.71E-07	-0.396774
8167163	ENST0000035741.	CXorf24	ENST00000357412 // CXorf24 // chromosome X open reading frame 24 // Xp11.23 // 203414 /	0.00825001	0.208328
8011850	NM_001212	C1QBP	NM_001212 // C1QBP // complement component 1, q subcomponent binding protein // 17p13.3	0.000513952	-0.268868
8130383	NM_019041	MTRF1L	NM_019041 // MTRF1L // mitochondrial translational release factor 1-like // 6q25-q26 //	0.00417766	-0.219453
8080973	NM_174907	PPP4R2	NM_174907 // PPP4R2 // protein phosphatase 4, regulatory subunit 2 // 3p13 // 151987 //	0.00486987	-0.217451
8119842	NM_203290	POLR1C	NM_203290 // POLR1C // polymerase (RNA) I polypeptide C, 30kDa // 6p21.1 // 9533 /// NM	4.65E-06	-0.347339
8139299	NM_001127218	POLD2	NM_001127218 // POLD2 // polymerase (DNA directed), delta 2, regulatory subunit 50kDa /	0.000434995	-0.274774
8147724	NM_001695	ATP6V1C1	NM_001695 // ATP6V1C1 // ATPase, H+ transporting, lysosomal 42kDa, V1 subunit C1 // 8q2	0.00726015	-0.206429
8066939	NM_004776	B4GALT5	NM_004776 // B4GALT5 // UDP-Gal:betaGlcNAc beta 1,4- galactosyltransferase, polypeptide	1.51E-09	-0.443193
8156761	NM_018946	NANS	NM_018946 // NANS // N-acetylneuraminic acid synthase // 9p24.1-p23 // 54187 /// ENST00	4.77E-06	-0.348906
8023159	NM_016427	TCEB3B	NM_016427 // TCEB3B // transcription elongation factor B polypeptide 3B (elongin A2) //	0.00262363	0.233584
8041170	---		---	0.00750045	-0.204201
7915787	NM_003629	PIK3R3	NM_003629 // PIK3R3 // phosphoinositide-3-kinase, regulatory subunit 3 (gamma) // 1p34.	0.00010886	-0.296821
7995829	NM_005951	MT1H	NM_005951 // MT1H // metallothionein 1H // 16q13 // 4496 /// ENST00000332374 // MT1H //	1.06E-06	-0.369292

8086451	NM_001099669	HIGD1A	NM_001099669 // HIGD1A // HIG1 hypoxia inducible domain family, member 1A // 3p22.1 //	0.00138674	-0.248202
8147447	NM_014754	PTDSS1	NM_014754 // PTDSS1 // phosphatidylserine synthase 1 // 8q22 // 9791 /// ENST0000033700	0.00852516	-0.199313
8128991	NM_001105206	LAMA4	NM_001105206 // LAMA4 // laminin, alpha 4 // 6q21 // 3910 /// NM_002290 // LAMA4 // lam	0.000598244	0.263765
8146896	---		---	0.00181542	0.241685
8067295	NM_016045	SLMO2	NM_016045 // SLMO2 // slowmo homolog 2 (Drosophila) // 20q13.32 // 51012 /// ENST000003	0.000778385	-0.260646
8124562	NM_001135215	ZNF323	NM_001135215 // ZNF323 // zinc finger protein 323 // 6p21.31|6p22.3-p22.1 // 64288 ///	4.00E-05	0.316388
7991335	NM_001150	ANPEP	NM_001150 // ANPEP // alanyl (membrane) aminopeptidase // 15q25-q26 // 290 /// ENST0000	0.00417865	-0.214773
7986509	ENST0000043652!	C15orf51	ENST00000436525 // C15orf51 // dynamin 1 pseudogene // 15q26.3 // 196968	0.00179112	0.239108
7986512	ENST0000043652!	C15orf51	ENST00000436525 // C15orf51 // dynamin 1 pseudogene // 15q26.3 // 196968	0.00179112	0.239108
7986527	---		---	0.00179112	0.239108
8042259	NM_005917	MDH1	NM_005917 // MDH1 // malate dehydrogenase 1, NAD (soluble) // 2p13.3 // 4190 /// ENST00	0.00879069	-0.204241
8076223	NR_002439	SNORD43	NR_002439 // SNORD43 // small nucleolar RNA, C/D box 43 // 22q13 // 26807	0.00252462	0.227763
8169272	NM_052936	ATG4A	NM_052936 // ATG4A // ATG4 autophagy related 4 homolog A (S. cerevisiae) // Xq22.1-q22.	0.00565635	-0.213938
8103725	---		---	2.33E-05	-0.327508
7995820	NM_005947	MT1B	NM_005947 // MT1B // metallothionein 1B // 16q13 // 4490 /// ENST00000334346 // MT1B //	4.19E-05	-0.313813
7900426	NM_022733	SMAP2	NM_022733 // SMAP2 // small ArfGAP2 // 1p35.3-p34.1 // 64744 /// ENST00000372718 // SMA	3.06E-05	-0.322847

7917771	NM_014597	DNTTIP2	NM_014597 // DNTTIP2 // deoxynucleotidyltransferase, terminal, interacting protein 2 //	0.00555505	-0.21768
7914665	---		---	0.00276465	-0.233978
7993606	---		---	0.00205605	0.23636
7933723	NM_152230	IPMK	NM_152230 // IPMK // inositol polyphosphate multikinase // 10q21.1 // 253430 /// ENST00	0.0100964	-0.203621
7951325	NM_032299	DCUN1D5	NM_032299 // DCUN1D5 // DCN1, defective in cullin neddylation 1, domain containing 5 (S	0.00117968	-0.251073
7986520	ENST0000043652!	C15orf51	ENST00000436525 // C15orf51 // dynamin 1 pseudogene // 15q26.3 // 196968	0.00118228	0.247558
8003824	NM_004937	CTNS	NM_004937 // CTNS // cystinosis, nephropathic // 17p13 // 1497 /// NM_001031681 // CTNS	0.00070601	-0.254886
8003068	NM_005792	MPHOSPH6	NM_005792 // MPHOSPH6 // M-phase phosphoprotein 6 // 16q23.3 // 10200 /// ENST000002581	0.00253795	-0.235436
8150830	NM_006330	LYPLA1	NM_006330 // LYPLA1 // lysophospholipase I // 8q11.23 // 10434 /// ENST00000316963 // L	0.00660824	-0.210193
8026490	NR_015379	UCA1	NR_015379 // UCA1 // urothelial cancer associated 1 (non-protein coding) // 19p13.12 //	0.000734095	0.26499
7988687	NM_005254	GABPB1	NM_005254 // GABPB1 // GA binding protein transcription factor, beta subunit 1 // 15q21	1.61E-06	-0.361507
7986517	ENST0000043652!	C15orf51	ENST00000436525 // C15orf51 // dynamin 1 pseudogene // 15q26.3 // 196968	0.00124774	0.246621
7986522	ENST0000043652!	C15orf51	ENST00000436525 // C15orf51 // dynamin 1 pseudogene // 15q26.3 // 196968	0.00124774	0.246621
8076826	NR_026997	C22orf34	NR_026997 // C22orf34 // chromosome 22 open reading frame 34 // 22q13.33 // 348645 ///	0.00501575	0.214341
7928308	NM_019058	DDIT4	NM_019058 // DDIT4 // DNA-damage-inducible transcript 4 // 10pter-q26.12 // 54541 /// E	0.00117047	-0.253782
8063536	NM_003222	TFAP2C	NM_003222 // TFAP2C // transcription factor AP-2 gamma (activating enhancer binding pro	0.0030989	-0.229341
8135915	NM_013332	C7orf68	NM_013332 // C7orf68 // chromosome 7 open reading frame 68 // 7q32.1 // 29923 /// NM_00	3.15E-06	-0.35241

Supplemental Table S6. Subject demographics and clinical characteristics of the validation (NJH) cohort by IIP subcategory.

Disease Group[1]	Control	All IIPs	IPF	NSIP	UF	RB-ILD
Number	39	131	111	12	8	0
Age - mean (std dev)	50.7 (19.4)	61.8(10.3)	62.8(8.8)	52.6(9.6)	62.8(21.2)	NA
Gender – % male	64	59	59	58	50	NA
Race - % Caucasian	74	88	88	75	100	NA
Smoker –						
Current	16	13	12	0	1	
Former	5	67	62	2	3	NA
Never	17	39	32	5	2	
Unknown	1	12	5	5	2	
Pack years - mean (std dev)[2]	26.3(19.5)	41.8(31.5)	43.5(31.5)	6.6(7.7)	28.3(29.8)	NA
Pre-BD FVC, %predicted - mean (std dev)	NA	65.9(17.2)	66.0(17.4)	60.3(13.4)	72.0(19.2)	NA
D_LCO, %predicted - mean (std dev)	NA	52.5(16.1)	52.8(16.3)	52.0(11.4)	48.9(20.2)	NA

Supplemental Table S7. Validation analysis of 58 genes from table 2 in the NJH replication cohort.

Transcript ID	Gene Symbol	p value FVC categorical	FC severe/mild FVC	p value DLCO categorical	FC severe/mild DLCO	p value FVC continous	PartialCorr FVC	p value DLCO continous	PartialCorr DLCO
7897449	SPSB1	0.0790135	1.15	0.055715	1.12	**0.00057677**	-0.30	**0.0170299**	-0.20
7902227	GADD45A	0.2062315	1.10	**0.0096343**	1.22	**0.00912315**	-0.22	**0.0136273**	-0.21
7915787	PIK3R3	**0.0087648**	1.33	**0.0136426**	1.21	**0.0020963**	-0.26	0.0511715	-0.16
7921344	ELL2	0.165931	1.16	0.136029	1.12	0.077596	-0.13	**0.049247**	-0.16
7921821	ADAMTS4	0.337822	1.12	**0.00153113**	1.70	0.123123	-0.11	**0.003140835**	-0.26
7922229	SELE	0.4252095	-1.06	**0.000903485**	2.01	0.4130915	-0.02	**0.00401934**	-0.25
7922610	ABL2	0.3352025	-1.04	**0.03667605**	1.12	**0.02537795**	-0.18	**0.04490485**	-0.16
7933855	RTKN2	**0.04530865**	-2.32	0.252356	-1.27	**0.0262835**	0.18	**0.02167185**	0.19
7940530	C11orf9	0.4284515	-1.03	0.1635445	1.11	0.3740775	0.03	0.4781425	0.01
7966839	VSIG10	0.492476	-1.00	0.3977515	1.02	0.330628	0.04	0.219206	0.07
7968650	C13orf36	0.110516	-1.20	0.3838115	-1.03	**0.03658055**	0.17	**0.03636145**	0.17
7974851	HIF1A	0.3753245	-1.05	**0.03206675**	1.21	0.22085	-0.07	**0.0098081**	-0.22
7976496	SERPINA3	**0.0051514**	1.81	**0.02074435**	1.41	**0.00249752**	-0.26	**0.004024195**	-0.25
7985934	SEMA4B	0.271081	1.05	0.0690115	1.09	0.201529	-0.08	**0.00327884**	-0.26
7986446	ALDH1A3	0.1609765	1.16	0.110594	1.14	0.208633	-0.08	**0.0139386**	-0.21
8006123	CPD	0.318277	1.04	**0.023567**	1.14	**0.00818305**	-0.22	**0.0078409**	-0.23
8007931	ITGB3	0.2943025	1.08	**0.03066025**	1.21	**0.02157325**	-0.19	**0.00849025**	-0.23
8035201	CPAMD8	0.0891565	-1.14	0.209808	-1.06	**0.00614675**	0.23	0.117221	0.11
8038117	DBP	0.1759325	-1.13	**0.0354854**	-1.18	0.143002	0.10	**0.0465892**	0.16
8041149	WDR43	0.307953	-1.05	0.4697685	1.01	0.0532005	-0.15	0.42267	-0.02
8041168	SNORD53	0.145538	1.12	**0.02615265**	1.16	**0.00624295**	-0.23	0.0764145	-0.14
8041206	LBH	0.220519	-1.10	0.0818715	-1.13	0.2200035	0.07	**0.003361345**	0.25
8043909	NPAS2	**0.01883625**	1.42	**0.02513055**	1.27	0.0886615	-0.13	**0.00983985**	-0.22
8043981	IL1R2	0.1063055	1.29	**0.00176277**	1.52	0.110183	-0.11	**0.00429388**	-0.25
8043995	IL1R1	**0.01268945**	1.30	**0.0087623**	1.22	**0.00656725**	-0.23	**0.00252742**	-0.26
8044391	MERTK	0.422335	-1.02	0.0564075	1.14	0.3055825	-0.05	**0.03748165**	-0.17
8057677	SLC40A1	0.2137165	-1.13	0.443101	-1.02	0.184665	0.08	0.058566	0.15
8059376	SERPINE2	0.2836455	1.10	**0.03707075**	1.24	0.4122985	-0.02	**0.004846275**	-0.24
8066939	B4GALT5	0.454471	-1.01	0.1569015	1.06	0.080988	-0.13	**0.0482084**	-0.16
8076515	ARFGAP3	0.1168495	-1.10	0.0990465	1.08	0.377461	-0.03	0.199033	-0.08

8082465	CCDC48	0.265085	-1.07	0.0709185	-1.12	0.1940115	0.08	**0.0066756**	0.23
8088560	ADAMTS9	0.1233925	1.28	0.1161065	1.20	0.092113	-0.12	0.1109955	-0.12
8089467	ZBED2	0.1812935	-1.16	**0.02672055**	-1.26	**0.03385905**	0.17	**0.00069944**	0.30
8099685	LGI2	0.0585265	1.27	0.47113	1.01	**0.0280374**	-0.18	0.116908	-0.11
8102482	SEC24D	0.263412	1.06	0.078415	1.10	0.0941595	-0.12	**0.01909805**	-0.20
8105040	OSMR	0.161192	1.12	**0.00439033**	1.25	**0.01004775**	-0.21	**0.001382375**	-0.28
8106098	MAP1B	0.1084065	1.23	**0.003847505**	1.37	0.056246	-0.15	**0.002953575**	-0.26
8106743	VCAN	0.2465375	1.12	**0.03417735**	1.25	0.1233155	-0.11	**0.02087665**	-0.19
8109086	ADRB2	0.288911	1.04	0.1328875	-1.06	0.4340195	-0.02	0.195088	0.08
8109383	GRIA1	0.3076065	-1.12	0.107938	-1.23	0.229918	0.07	**0.001521225**	0.28
8113220	ELL2	0.219427	1.12	0.149019	1.12	0.078263	-0.13	0.0656785	-0.14
8115814	SH3PXD2B	0.0974465	1.18	**0.04301335**	1.17	0.1941035	-0.08	**0.0300181**	-0.18
8117128	E2F3	0.4260345	-1.01	0.411855	1.01	0.1736825	-0.09	0.295487	-0.05
8125341	AGER	0.0780535	-1.67	0.4273655	-1.05	0.06486	0.14	0.153685	0.10
8133155	TPST1	0.455105	1.01	0.4547845	-1.01	0.400879	0.02	0.459215	-0.01
8145122	SLC39A14	0.1669075	-1.11	0.119705	1.09	0.2559935	0.06	**0.0408536**	-0.17
8146957	PI15	0.181961	1.27	**0.046174**	1.33	**0.01942805**	-0.19	**0.03754975**	-0.17
8149885	ADRA1A	0.451031	1.01	0.3027585	-1.04	0.0820815	0.13	0.064258	0.14
8155734	FAM189A2	0.39005	-1.05	0.2764585	-1.08	0.296923	0.05	**0.02640445**	0.18
8156043	PSAT1	0.055978	1.31	0.4451275	-1.02	**0.0494755**	-0.15	0.07062	-0.14
8156569	MIR23B	0.445777	1.03	**0.02206445**	-1.32	0.3691225	0.03	**0.0356101**	0.17
8157216	UGCG	0.456306	1.01	0.1381515	1.10	**0.01196025**	-0.21	0.0653325	-0.14
8162276	NFIL3	0.1424265	1.21	**0.002452705**	1.42	0.087546	-0.13	**0.00457649**	-0.25
8168749	SRPX2	**0.01117115**	1.52	**0.0043555**	1.41	**0.00387515**	-0.24	**0.00513255**	-0.24
8171248	KAL1	0.2660595	-1.15	0.0633605	-1.29	0.449832	0.01	**0.00284587**	0.26
8171427	FIGF	0.063676	-1.86	0.285387	-1.18	0.0858775	0.13	**0.0248813**	0.19
8178771	AGER	0.0810545	-1.66	0.430219	-1.05	0.0653175	0.14	0.153848	0.10
8179967	AGER	0.0767195	-1.63	0.4242645	-1.05	0.0638955	0.14	0.1480685	0.10

Supplemental Table S8. Primers for SYBRGreen quantitative RT-PCR assays.

Gene	Accession	Primer	Sequence	Length	Start	Stop	Tm	GC%
GADD45A	NM_001924.3	GADD45A F	CTGCTCAACGTCGACCCCGATA	22	454	475	58.48	59.09
		GADD45A R	CTCGCAGCAAAACGCCTGGA	20	570	551	58.2	60
		Exon junction	464/465 (forward primer) on template NM_001924.3					
SELE	NM_000450.2	SELE F	AGGTTCCTTCCTGCCAAGTGGT	22	1608	1629	57.74	54.55
		SELE R	TGTCCGAGCTGCAGAGCCAT	20	1756	1737	58.14	60
		Exon junction	1625/1626 (forward primer) on template NM_000450.2					
PI15	NM_015886.3	PI15 F	TACGCAGATGGTTTGGGCCACT	22	698	719	58.49	54.55
		PI15 R	GTAAACTGCACGTCGCCACACA	22	797	776	58.32	54.55
		Exon junction	704/705 (forward primer) on template NM_015886.3					
GAPDH	NM_002046.3	GAPDH F	TCCTGTTCGACAGTCAGCCGCA	22	39	60	60.43	59.09
		GAPDH R	GCGCCCAATACGACCAAATCCGT	23	150	128	60.12	56.52
		Exon junction	131/132 (reverse primer) on template NM_002046.3					
